# Research on the Hormonomics of Three *Lilium* Species and Their Flavonoid Diversification and Specificity

**DOI:** 10.3390/antiox14070862

**Published:** 2025-07-14

**Authors:** Xuanyu He, Jie Fang, Biwei Hong, Xueying Zhang, Linying Li, Yuqing He, Chaomin Chen, Shuang Liang, Zelong Xu, Chunlan Peng, Jirong Huang, Gaojie Hong, Qundan Lv

**Affiliations:** 1 Key Laboratory of Saline-Alkali Vegetation Ecology Restoration, Northeast Forestry University, Ministry of Education, Harbin 150040, Chinahuangjr@shnu.edu.cn (J.H.); 2 State Key Laboratory for Managing Biotic and Chemical Treats to the Quality and Safety of Agro-Products, Zhejiang Academy of Agricultural Sciences, Hangzhou 310021, Chinalilinying@zaas.ac.cn (L.L.); heyuqing@zaas.ac.cn (Y.H.);; 3 Lishui Institute of Agriculture and Forestry Sciences, Lishui 323020, China; 4 Zhejiang Bifeng Agricultural Development Co., Ltd., Lishui 323900, China; 5 Key Laboratory of Biotechnology in Plant Protection of MOA of China and Zhejiang Province, Zhejiang Academy of Agricultural Sciences, Hangzhou 310021, China; 6 Institute of Virology and Biotechnology, Zhejiang Academy of Agricultural Sciences, Hangzhou 310021, China

**Keywords:** hormonomics, lily bulbs, metabolites, flavonoids

## Abstract

Hormonomics represents an innovative approach to plant physiology and biochemistry. We utilized hormonomics to analyze the hormone profiles of three lily bulbs. The hormones specifically enriched in BiFeng7 lily show a strong response to secondary metabolism pathways, while the Diwanghuang lily profile was predominantly focused on growth. Physiological experiments demonstrated that Diwanghuang exhibited higher levels of primary nutrients, whereas BiFeng7 displayed a greater concentration of secondary metabolites and enhanced antioxidant capacity. Through untargeted metabolomic analysis, it was revealed that BiFeng7 highly enriched four flavonoid glycosides, two flavones, one flavan, one pyranoflavonoid, two isoflavonoid O-glycosides and one rotenoid. These findings provide valuable information for developing breeding strategies and cultivation practices aimed at achieving ornamental quality, nutritional value, or stress resilience outcomes. This research demonstrates the practical application of hormone profiling in plant evaluation and offers insights into the mechanisms underlying flavonoid synthesis in lilies, serving as a reference for breeding stress-resistant lily varieties.

## 1. Introduction

The pursuit of enhancing crop breeding has been a constant throughout human history. With the rapid advancement of biotechnology in recent decades, a growing array of biotechnological methods has not only significantly increased both the qualitative and quantitative yields of crops but has also sparked further enthusiasm for developing novel strategies to assess and improve crops. Plant hormonomics, a recently coined term, aims to analyze the composition, concentration changes, and interaction networks of all plant hormones in each sample, as well as their dynamic changes during physiological and pathological processes [1,2]. Akin to genomics, transcriptomics, proteomics, and metabolomics, plant hormonomics also focuses on a systems biology perspective, relies on advanced biotechnology, closely links to biological processes, and emphasizes mutual influences and feedback regulation. Contrastingly, the profile of hormonomics is more phenotypically predictable because phytohormones act as commanders, responding to environmental changes to activate a physiological cascade that redirects metabolic resources and growth [1]. This characteristic has been consistently validated by extensive studies—whether in fundamental experiments or practical applications, hormonal regulation of plant physiological states consistently yields significant outcomes [3,4,5,6,7]. For example, a study used hormonomics to map hormone distribution in rice organs and identify their interactions. By combining hormone profiles with transcriptomics, it further analyzed the link between gene expression and hormone metabolism, providing valuable insights for breeding high-yield varieties [8]. A recent study revealed that ABA and JA can enhance tomato drought resistance by mediating ERF.D2, while exogenous application of ABA also significantly improves plant drought stress tolerance [7]. As initiators and regulators of various life events, phytohormones can serve as useful biomarkers indicating the physiological state of crops, which could facilitate crop breeding and management [1]. A study established the Wheat Phytohormone Metabolic Regulation Network (WPMRN) spanning the entire growth cycle, providing groundbreaking insights for investigating cereal metabolic regulation networks and advancing wheat genetic improvement and breeding practices [9]. Therefore, in agricultural management, precise analysis of hormone profile dynamics is crucial for optimizing production management decisions and breeding practices.

In recent years, in agricultural production management, a comprehensive analysis of major phytohormones has emerged as an advanced and effective approach [9]. This achievement stems from the rapid advancement of hormone omics, which has significantly enhanced the evaluation criteria and methodologies for germplasm resources. Comprehensive hormonal profiling of elite germplasms, particularly their tissue- and cell-specific accumulation patterns, provides critical insights into the regulatory mechanisms underlying agronomic traits. These advances not only inform genetic improvement strategies for breeding programs but also facilitate the discovery of beneficial genes and metabolic pathways. Moreover, the relationship between hormonal profiles and secondary metabolite biosynthesis represents a particularly promising area of investigation. Stress-responsive hormones, such as abscisic acid and jasmonic acid, not only mediate immediate stress responses but also trigger the biosynthesis of protective compounds, including flavonoids and other antioxidants. This hormone-metabolite connection suggests that hormonomic analysis could serve as a predictive tool for assessing plant metabolic capacity and stress resilience potential, making it invaluable for identifying superior germplasm with enhanced nutritional and stress-tolerance properties.

Lilies (*Lilium* spp.), known as the king of bulbous flowers, are prized for their vibrant, fragrant blossoms and nutrient-rich bulbs, which contain various physiologically active compounds, endowing them with significant edible and medicinal value. The lily bulb serves not only as a nutritive organ but also as a propagative organ. Therefore, bulb quality is crucial for lilies. The formation and development of the bulbs are regulated by a complex network of hormones [10]. Therefore, it is of great industrial value to explore the effects of plant hormones on the genesis and development of lily bulbs [11]. In studies of bulb development, cytokinin (CK), auxin, gibberellins (GA), and abscisic acid (ABA) are often regarded as the main hormones affecting bulb development [12,13]. GA helps to increase the activity of sucrose synthetase and boost starch content during the early stage of bulb expansion. Additionally, GA can reduce the rot rate of lily scales and increase the number of small bulbs [11]. ABA is widely regarded as a stimulating hormone in bulb formation. The antagonism between ABA and GAs can regulate the accumulation of lake powder in Gladiolus gandavensis, thus affecting the development of its bulbs [14]. ABA, within a certain concentration range, can promote the formation and expansion of lily bulbs [15]. On one hand, each phytohormone has a specific physiological function; on the other hand, they always act synergistically and antagonistically, either unidirectionally or bidirectionally, to affect biosynthesis, signaling, and output processes at multiple levels, including gene expression and the formation of primary and secondary metabolites [16,17]. Therefore, understanding these hormonal dynamics is crucial for improving lily cultivation practices and developing stress-resistant varieties that can maintain quality under challenging environmental conditions.

In this study, we employed comprehensive hormonomic profiling to investigate three lily cultivars with contrasting phenotypes: Bifeng 7, a newly identified variety exhibiting exceptional stress resistance; Diwanghuang, known for superior physiological quality and growth characteristics [18]; and Annika, a high-yielding standard cultivar. Our primary objectives were to establish complete hormonal profiles using targeted mass spectrometry analysis, identify hormone signatures associated with stress resilience and metabolic capacity, correlate hormonal patterns with physiological parameters and secondary metabolite accumulation, and provide molecular insights that could guide the development of stress-tolerant lily varieties through marker-assisted breeding approaches.

## 2. Materials and Methods

### 2.1. Plant Materials

The bulbs from three different Asian lily varieties—BiFeng 7 (BF7), Diwanghuang (DWH), and Annika (ANK) were collected from the field located in Shuqiao township, Qingtian County, Lishui City, Zhejiang Province of China, which is situated in a subtropical monsoon climate zone, with geographic coordinates of 28°25′ N latitude and 120°03′ E longitude. The climate is warm and humid, with distinct four seasons. The region is characterized by sandy loam soil and is situated at an elevation of 245 m above sea level, well-distanced from industrial activities. Lily bulb samples were collected in early July, when temperatures averaged around 36 °C. At the time of sampling, the lower leaves of the aerial plant parts had withered, whereas the upper leaves remained healthy as the plant was undergoing a brief dormancy period during the summer high-temperature season. The samples were ground in liquid nitrogen and then stored in a −80 °C freezer for subsequent treatments. Three independent plant samples from each lily variety were selected for hormonomics analysis.

### 2.2. Preparation of Hormonomics Samples and Hormone Standard Curves

Phytohormones contents were detected by MetWare: http://www.metware.cn/ (accessed on 20 April 2025) based on the AB Sciex QTRAP 6500 LC-MS/MS platform (SCIEX, Marlborough, MA, USA). The above samples (50 mg) were weighed, an appropriate amount of internal standard was added, and hormones were extracted using 1 mL of methanol/water/formic acid (15:4:1, *v*/*v*/*v*). Vortex for 10 min, then centrifuge at 13,400 g/min for 5 min at 4 °C. Transfer the supernatant to a new tube for concentration. After concentration, reconstitute with 100 μL of 80% methanol/water solution, filter through a 0.22 μm filter, and transfer to an injection vial for LC-MS/MS analysis. The hormones detected were Auxins, Cytokinins (CKs), Abscisic Acid (ABA), Jasmonates (JAs), Salicylic Acid (SA), Gibberellins (GAs), Ethylene (ETH), Strigolactones (SLs), Brassinolide (BR), and other hormone precursor substances and derivatives (Please refer to the Table S1 for the specific types of hormones). The internal standards were hormone reference standard compounds, purchased from Olchemim and ISOREAG company, with a purity of ≥99%. The data acquisition system primarily consists of Ultra Performance Liquid Chromatography (UPLC) (ExionLC™ AD: https://sciex.com.cn/ (accessed on 10 March 2025)) and Tandem Mass Spectrometry (MS/MS) (QTRAP^®^ 6500+: https://sciex.com.cn/ (accessed on 10 March 2025)).

Prepare standard solutions of varying concentrations: 0.01 ng/mL, 0.05 ng/mL, 0.1 ng/mL, 0.5 ng/mL, 1 ng/mL, 5 ng/mL, 10 ng/mL, 50 ng/mL, 100 ng/mL, 200 ng/mL, and 500 ng/mL. For seven substances, including TRP, SAG, IAA-Glc, etc., prepare solutions at 20 times the above concentrations, resulting in a standard curve concentration range of 0.2–10,000 ng/mL. For Phe, prepare solutions at 30 times the above concentrations, resulting in a standard curve concentration range of 0.3–15,000 ng/mL. Obtain the mass spectrometry peak intensity data corresponding to the quantification signals of each concentration standard; plot the standard curves of different substances with the ratio of the external standard concentration to the internal standard concentration on the *x*-axis and the ratio of the external standard peak area to the internal standard peak area on the *y*-axis.

Based on the standards and the quantitative analysis of the data detected by mass spectrometry, the amount of hormone in the sample (ng/g) was calculated using the formula C × V/1000/m, where C represents the concentration value (ng/mL) obtained by substituting the ratio of the product peak area in the sample into the standard curve, V represents the volume of the solution (μL), and m represents the mass of the sample weighed (g).

### 2.3. Detection and Analysis of Plant Hormones by LC-MS

The UPLC Conditions: The analytical conditions were as follows, LC: column, Waters ACQUITY UPLC HSS T3 C18 (Milford, MA, USA; 100 mm × 2.1 mm i.d., 1.8 µm); solvent system, water with 0.04% acetic acid (A), acetonitrile with 0.04% acetic acid (B); gradient program, started at 5% B (0–1 min), increased to 95% B (1–8 min), 95% B (8–9 min), finally ramped back to 5% B (9.1–12 min); flow rate, 220.35 mL/min; temperature, 40 °C; injection volume: 2 μL [19,20].

ESI-MS/MS Conditions: Linear ion trap (LIT) and triple quadrupole (QQQ) scans were acquired on a triple quadrupole-linear ion trap mass spectrometer (QTRAP), QTRAP^®^ 6500+ LC-MS/MS System, equipped with an ESI Turbo IonSpray interface, operating in both positive and negative ion mode and controlled by Analyst 1.6.3 software (Sciex). The ESI source operation parameters were as follows: ion source, ESI+/−; source temperature 550 °C; ion spray voltage (IS) 5500 V (Positive), −4500 V (Negative); curtain gas (CUR) was set at 35 psi, respectively. Phytohormones were analyzed using scheduled multiple reaction monitoring (MRM). Data acquisitions were performed using Analyst 1.6.3 software (Sciex). Mass spectrometer parameters, including the declustering potentials (DP) and collision energies (CE) for individual MRM transitions, were conducted with further DP and CE optimization. A specific set of MRM transitions was monitored for each period according to the metabolites eluted within this period [21].

### 2.4. Measurement of Physiological Indicators

The physiological indicators were measured using the Suzhou Keming reagent kits: http://www.cominbio.com/index.html (accessed on 5 January 2025). Please refer to the reagent kit protocols for detailed methods. Among these, the determination of total protein content is performed using the Coomassie Brilliant Blue method (item number: KMSP-1-W); the determination of reducing sugar content is performed using the 3,5-Dinitrosalicylic Acid (DNS) colorimetric method (item number: HYT-1-Y); the determination of polysaccharides content is performed using the Phenol-Sulfuric Acid method (item number: ZDT-1-Y); the determination of amylose content is performed using the acid hydrolysis method (item number: DF-1-Y); the determination of total antioxidant capacity is performed using FRAP method (item number: FRAP-1-G); the determination of flavonoid content is performed using the Aluminum ion colorimetric method (item number: LHT-1-G); the determination of phenols content is performed using the Tungstomolybdic acid reduction method (item number:TP-1-G); the determination of saponins content is performed using the Vanillin-Perchloric acid chromogenic method (item number: TSG-1-Y); the determination of activity of superoxide dismutase is performed using the NBT method (item number: SOD-1-Y POD-1-Y QZQ-1-G); the determination of activity of superoxide dismutase is performed using the H_2_O_2_ oxidant method (item number: POD-1-Y); the determination of activity of superoxide dismutase is performed using the H_2_O_2_ oxidant method (item number: POD-1-Y); the determination of hydroxyl radical scavenging capacity is performed using the salicylic acid method (item number: QZQ-1-G); and the determination of superoxide anion content is performed using the hydroxylamine oxidation method (item: SA-1-G).

### 2.5. Detection Conditions for Non-Targeted Metabolomics

The non-target metabolomics was provided by the Central Laboratory of Zhejiang Academy of Agricultural Sciences, State Key Laboratory for Managing Biotic and Chemical Threats to the Quality and Safety of Agroproducts, for data profiling as well as data analysis. The analysis employed LC-MS/MS technology using the high-resolution mass spectrometer Vion IMS QTOF (Waters, USA) coupled with a BEH C18 analytical column (optimized for medium-polarity compounds). Both positive and negative ion modes were used to increase metabolite coverage. Data processing was carried out with Progenesis QI (Waters, Milford, MA, USA), including peak extraction, alignment, and compound identification. Data acquisition was controlled by UNIFI software (version 1.9). All flavonoids were screened by comparing them against a reference database.

Chromatographic Conditions: Column: Waters ACQUITY UPLC BEH C18 (1.7 µm, 2.1 mm × 100 mm). Mobile Phase: Phase A: 98% H_2_O, 2% ACN, 0.1% formic acid. Phase B: 98% ACN, 2% H_2_O, 0.1% formic acid. Flow Rate: 0.4 mL/min. Column Temperature: 40 °C. Equilibration Time: 1.5 min between each gradient run. The gradient program started at 5% B (0–0.5 min), increased to 100% B (0.5–16 min), 100% B (16–18.5 min), decreased to 5%B (18.5–18.6 min), final ramped back to 5% B (18.6–20 min).

### 2.6. Extraction and Analysis of Untargeted Metabolomics Data

Instrument Parameters and Data Acquisition Protocol: Analysis mode: Sensitivity; Capillary voltage: ESI+ 2.5 kV, ESI− 2 kV; Ion source temperature: 120 °C; Desolvation gas temperature: 500 °C; Desolvation gas flow rate: 800 L/h; Cone gas flow rate: 50 L/h. Data acquisition is performed in the data-independent acquisition MSE mode, with a mass range of 50–1000 *m*/*z*, and the acquisition cycle time is 0.3 s. Collision energy ranges from 10 eV to 45 eV, and data is collected in both positive and negative ion modes. During the acquisition process, leucine-enkephalin (positive ion mode: 556.2766 *m*/*z*; negative ion mode: 554.2620 *m*/*z*) is injected as an online mass calibration standard to maintain mass accuracy.

Data Peak Extraction and Database Search: The LC-MS raw data were deconvoluted using Waters Progenesis QI (v 3.0.3.0). The QC file was selected as the reference for peak extraction, alignment, and area normalization. The compound identification was performed by comparing against the Metlin 2019, NIST, and a self-built plant database. The isotope similarity threshold was set to >80%, with a mass deviation of the parent ion set to 10 ppm and the fragment ion mass deviation set to 20 ppm. In positive ion mode, the adducts were set to [M + H]+ and [M + Na]+, and in negative ion mode, the adducts were set to [M − H]− and [M + FA − H]−. For database search results, compounds with a total score greater than 40 and a fragmentation score greater than 20 were considered Level 3 reliable identifications, where both the parent ion spectrum and the fragment ion spectrum matched. Compounds with a fragmentation score less than 20 but a total score greater than 36 were considered Level 3 identifications, where the parent ion spectrum could be matched but the fragment ion spectrum could not be conclusively identified.

### 2.7. Data Treatment and Statistical Analysis

Mass spectrometry data were processed using Analyst 1.6.3 software. The following figures display the Total Ion Chromatogram (TIC) and Extracted Ion Chromatogram (XIC). The x-axis represents the retention time (Time, min) of the detected ions, and the y-axis represents the ion intensity (Intensity, cps) of the detected ions. Mass spectrometry data were processed using MultiQuant 3.0.3 software. By referencing the retention time and peak shape information of standard compounds, the chromatographic peaks of the target substances detected in different samples were integrated and calibrated to ensure the accuracy of both qualitative and quantitative analysis. The peak area represents the relative content of the substance in the sample.

Statistically significant differences were analyzed using a *t*-test and one-way analysis of variance (ANOVA) followed by Duncan’s multiple comparison test. All results are expressed as the mean ± Standard Error of the Mean (SEM) of at least three independent biological replicates.

## 3. Results

### 3.1. Hormonomics Data and Multivariate Analysis

**Figure 1:**
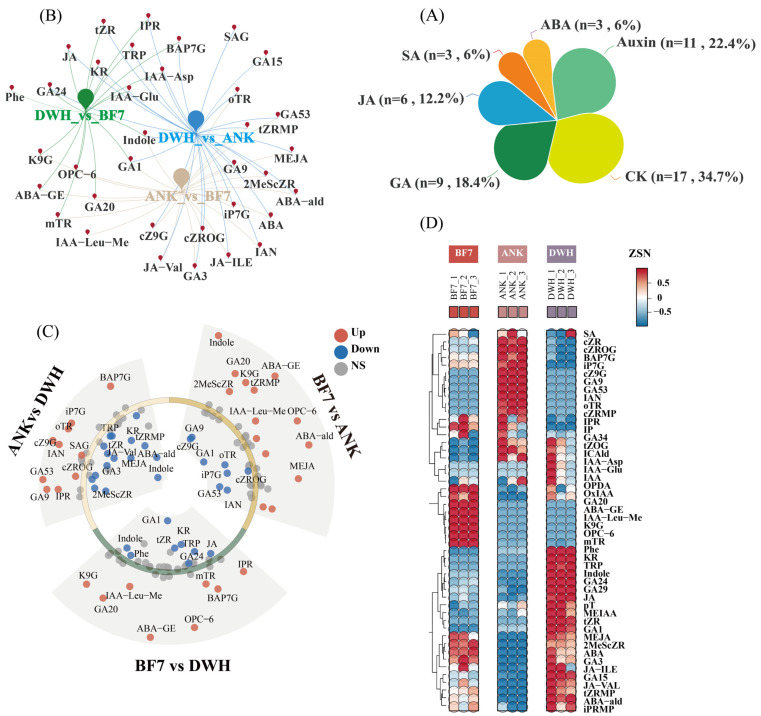
**Hormonal Profiling of Three Types of Lily Bulbs.** (**A**) The composition of hormone subclasses. A total of 48 hormones are divided into six subclasses, including three ABAs, 11 Auxins, 16 CKs, nine GAs, six Jas, and three SAs. (**B**) The Venn network of DWH vs. BF7, DWH vs. ANK and ANK vs. BF7. (**C**) Multiple differential volcano analyses illustrate the expression of differential hormones across BF7 vs. ANK, BF7 vs. DWH and ANK vs. DWH. Red dots show significantly upregulated hormones, blue dots show significantly downregulated hormones, and gray dots show these hormones that do not show significant differences between the groups. (**D**) Heatmap cluster depicting the differences in the hormones between ANK, BF7, and DWH. The color scale represents the relative abundance of each hormone, with blue indicating low levels and red indicating high levels. The rows represent different hormones, and the columns represent the three samples. Data were normalized for better comparison.

The bulb is the most important nutritional and reproductive organ of the lily, and its hormone level directly reflects the growth status. In this study, the bulbs of ANK, BF7 and DWH were selected for hormonomics determination. The total ion current (TIC) curves exhibit a high degree of overlap, with the retention time (RT) and peak intensity (PI) remaining consistent, indicating good signal stability (Figure S1). The Coefficient of Variation (CV) among each group of samples was compared, and the proportion of substances for which the CV value of the Quality Control (QC) samples was less than 0.2 and exceeded 80%, signifying a high degree of stability in the experimental data (Figure S2). This study performed a targeted assay for the identification and quantification of 109 hormones, and 49 hormones were detected (Supplementary Materials S2), including three ABAs, 11 Auxins, 17 CKs, nine GAs, six JAs, and three SAs (Figure 1A and Table 1). The inter-group metabolic disparities and intra-group sample variation were analyzed by Principal Component Analysis (PCA). PC1 exhibited a contribution rate of 52%, while PC2 showed 33%. The samples are clustered closely, suggesting data stability (Figure S3). The distinct separation among the samples implies that further in-depth analysis is needed.

### 3.2. Hormonomics Differences Among BF7, ANK, and DWH

Targeted hormonomics was used to determine the differential hormones among BF7 vs. ANK, BF7 vs. DWH, and DWH vs. ANK. The Venn-Network analysis revealed the differential distribution patterns of 48 hormones across the three lilies (Figure 1B). Compared to the other two bulbs, eight hormones showed significant differences in BF7 (yellow-green intersection points: ABA-GE, K9G, OPC-6, mTR, GA20, IAA-Leu-Me, GA1, Indole). Compared to the others, 11 hormones showed significant differences in ANK (blue-green intersection points: IPR, BAP7G, tZR, JA, KR, TRP, IAA-Asp, IAA-Glu, GA1, GA24, and Indole). Compared to the others, 17 hormones showed significant differences in DWH (yellow-blue intersection points: oTR, tZRMP, GA9, GA53, MEJA, 2MeScZR, iP7G, ABA, and ABA-ald). Through an integrative analysis combining multiple differential volcano analyses and heatmap clustering, a clearer and more explicit revelation was achieved regarding the specific classes of hormones exhibiting pronounced and statistically significant disparities among different groups (Figure 1C,D).

Through comparison, we found that ABA-GE, ABA, ABA-ald, OPC-6, GA20, MEJA, mTR, K9G, tZRMP, IAA-Leu-Me and 2MeScZR in BF7 were significantly higher than in ANK. Similarly, ABA-GE, OPC-6, GA20, K9G, IAA-Leu-Me, BAP7G, mTR, and IPR in BF7 were significantly higher than in DWH. Additionally, Phe, KR, TRP, Indole, ABA-ald, MEJA, tZRMP, KR, GA3, 2MeScZR, JA-Val, and tZR in DWH were significantly higher than in ANK. GA1, KR, Indole, tZR, TRP, JA, Phe, and GA24 in DWH were significantly higher than in BF7. That cZ9G, IAN, oTR, iP7G, GA53, GA9, BAP7G, IPR, cZROG, and SAG in ANK were significantly higher than in DWH, and that GA1, GA53, cZ9G, IAN, cZROG, iP7G, and oTR in ANK were significantly higher than in BF7. In short, the hormone profile of BF7 focuses on stress resistance, while that of DWH is more oriented towards growth and development. However, although many hormones exhibit significant disparities among diverse groups, their biological significance may be negligible due to extremely low content. For example, the content of IAA-Leu-Me, which exists only in BF7, is merely 0.03 ng/g. The content of oTR, which exists only in ANK, is only 0.16 ng/g. The content of tZR, which exists only in DWH, is only 0.08 ng/g.

**Figure 2:**
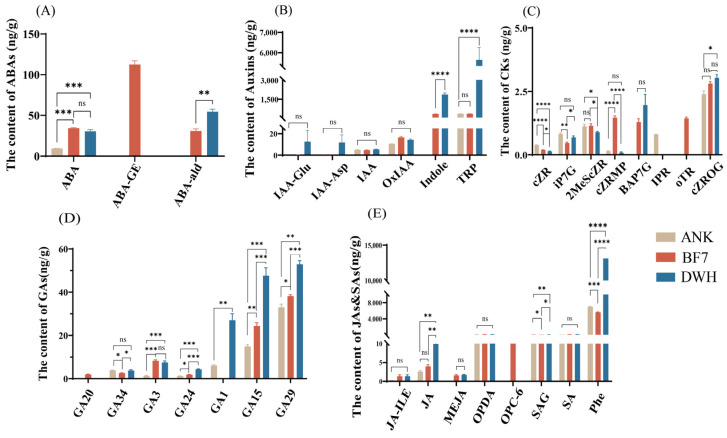
**The content of different hormones.** (**A**) The content of three types of ABA hormones (ABA, ABA-GE and ABA-ald). (**B**) The content of ten types of Auxin hormones (IAA-Asp, OxIAA, Indole, IAN, IAA, IAA-Glu, ACAld, MEIAA, TRP, and IAA-Leu-Me). (**C**) The content of seventeen types of CK hormones (KR, tZR, oTR, K9G, IP, cZR, cZROG, iPRMP, iP7G, tZOG, 2MeScZR, cZRMG, mTR, pT, BAP7G, tZRMP, and IPR). (**D**) The content of nine types of GA hormones (GA1, GA3, GA9, GA15, GA20, GA24, GA29, GA34, and GA53). (**E**) JA-ILE, JA, MEJA, OPDA, OPC-6, SAG, SA, and Phe. * *p* < 0.05, ** *p* < 0.01, *** *p* < 0.001, **** *p* < 0.0001, (Student’s *t*-test).

To more accurately identify biologically significant hormones, we further compared the absolute contents of hormones with significant differences. In the ABA class, the content of ABA-GE is 112.28 ng/g, and it exists only in BF7. ABA-ald is absent in ANK, and is significantly higher in DWH than in BF7. There is no significant difference in ABA between BF7 and DWH, but both are significantly higher than in ANK (Figure 2A). In the class of auxins, indole is absent in ANK. The content of indole in DWH is as high as 1878.69 ng/g, which is significantly higher than that in BF7 (330.92 ng/g). The content of TRP in DWH is 5639.49 ng/g, which is significantly higher than that in ANK and BF7. TRP is the precursor for indole synthesis. The high contents of TRP and indole in DWH suggest the potential growth capacity of DWH. Indole is not only an important precursor for the synthesis of the plant growth hormone IAA, but also indole and its derivatives can act as defense signal molecules to play a defensive role, which is consistent with the excellent biological qualities of DWH (Figure 2B). The hormone levels in the GA class are generally low. GA20 (1.94 ng/g) is present only in BF7. GA1 is absent in BF7 and is significantly higher in DWH than in ANK (Figure 2C). Similarly, the hormone contents in the CK class are generally low, which may be related to the fact that the bulb samples are not in a growing state (Figure 2D). In the JA and SA classes, JA is significantly higher in DWH than in BF7 and ANK. While OPC-6 only exists in BF7 (22 ng/g), and as an important intermediate in the JA synthesis pathway, it indicates the high abundance and rapid response ability of the JA pool in BF7, suggesting the high resistance of BF7 to biological stress. And SAG is significantly higher in ANK (111.52 ng/g) than in BF7 (82.74 ng/g) and DWH (49.73 ng/g), indicating that ANK may have more outstanding resistance to biotrophic pathogens. In addition, the content of phe in DWH is as high as 13,131.27 ng/g. Phe is a precursor for the synthesis of many secondary metabolites, and the high content of phe implies a high abundance of the secondary metabolite reserve pool (Figure 2E). Overall, DWH and BF7 exhibited better physiological qualities and disease resistance. In the subsequent KEGG pathway enrichment analysis, among the three comparisons, BF7 vs. ANK, BF7 vs. DWH, and DWH vs. ANK, hormones with significant differences were primarily enriched in the metabolic pathways and biosynthesis of secondary metabolites pathways, indicating that these hormones are closely related to the synthesis of secondary metabolites. This result further corroborates that DWH and BF7 possess superior physiological qualities and high resistance (Figures S4–S6).

### 3.3. Analysis of Nutrient Components and Secondary Metabolites of Bulbs

**Figure 3:**
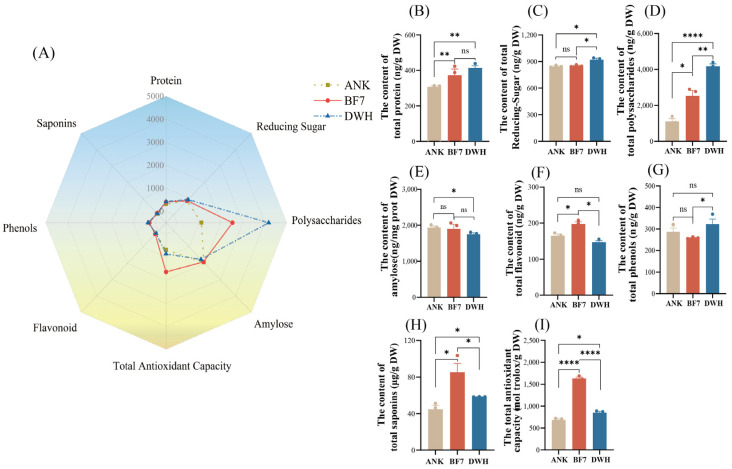
**The content of eight physiological indicators in ANK, BF7 and DWH.** (**A**) The radar chart illustrates the preferences of eight physiological indicators in ANK, BF7, and DWH. (**B**) The content of total protein in ANK, BF7, and DWH. (**C**) The content of total reducing sugar in ANK, BF7, and DWH. (**D**) The content of total polysaccharides in ANK, BF7, and DWH. (**E**) The content of amylose in ANK, BF7, and DWH. (**F**) The content of total flavonoids in ANK, BF7, and DWH. (**G**) The content of total phenols in ANK, BF7, and DWH. (**H**) The content of total saponins in ANK, BF7, and DWH. (**I**) The content of total antioxidant capacity in ANK, BF7, and DWH. Asterisks indicated the statistical significance: * *p* < 0.05, ** *p* < 0.01, **** *p* < 0.0001 (Student’s *t*-test). All the measured results are derived from biochemical experiments.

To further assess the relationship between the intrinsic hormonal profile and physiological phenotype, we measured the levels of eight physiological indicators, including total protein, total polysaccharides, total reducing sugars, amylose, total flavonoids, total phenols, total saponins, and total antioxidant capacity, in three bulbs (Figure 3A). The results show that secondary metabolites are more dominant in BF7, while nutritional compounds are more abundant in DWH. Specifically, protein, polysaccharides, and reducing sugars were highest in DWH (Figure 3B–D). The content of total flavonoids, saponins, and antioxidant capacity was significantly higher in BF7 than in DWH and ANK (Figure 3F,H,I). These findings align with the hormonomics data, demonstrating that hormone profiling accurately reflects the plant’s physiological state.

### 3.4. Untargeted Metabolomics Analysis

**Figure 4:**
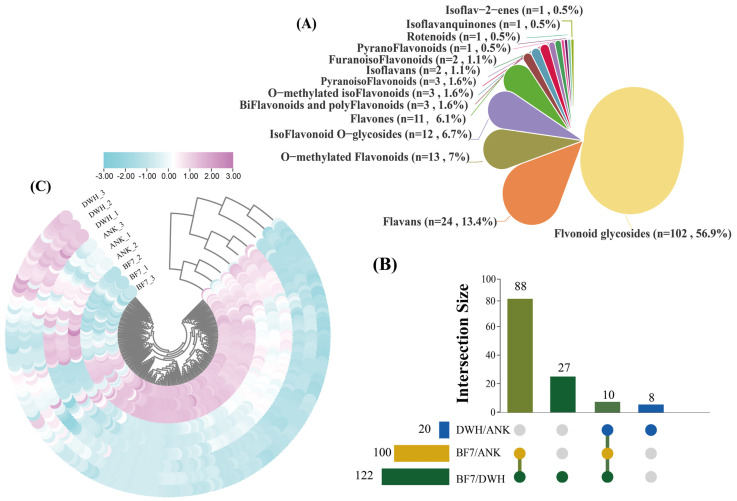
**The profiles of flavonoids in ANK, BF7 and DWH.** (**A**) Classification and quantity of flavonoids in three lilies. (**B**) The upset plot illustrates the distribution of differential flavonoids. The horizontal bar chart on the left shows the distribution of these compounds across different lily varieties. The dark green bar represents 122 flavonoids in BF7 vs. DWH, yellow represents 100 flavonoids shared in BF7 vs. ANK, and blue indicates 20 flavonoids shared in DWH vs. ANK. The vertical bar chart displays the distribution of flavonoids present in the pairwise comparisons of BF7 vs. DWH, BF7 vs. ANK, and DWH vs. ANK. Among them, 88 flavonoids are common to both BF7 vs. DWH and BF7 vs. ANK; 27 flavonoids are exclusive to BF7 vs. DWH; 10 flavonoids are present in all three groups; 8 flavonoids are unique to DWH vs. ANK. (**C**) Cluster heatmap analysis of flavonoids in BF7, ANK, and DWH. The cluster heatmap shows the enrichment of flavonoids in ANK, BF7 and DWH. The color scale represents the relative abundance of each flavonoid, with blue indicating low levels and pink indicating high levels. The rows represent different flavonoid compounds, and the columns represent the three samples. Data were normalized for better comparison.

To further investigate the differences in antioxidant capacities among the three lilies, we quantified the content of various flavonoids in the bulbs using LC-MS. Overall, the abundance of flavonoids in BF7 is notably higher than in ANK and DWH (Figure 4). Specifically, the three types of lilies contain a total of 102 flavonoid glycosides, 11 flavones, 13 O-methylated flavonoids, 24 flavans, one pyranoflavonoid, three biflavonoids and polyflavonoids, two isoflavans, 3 O-methylated isoflavonoids, 12 isoflavonoid O-glycosides, three pyranoisoflavonoids, one rotenoid, one isoflavanquinone, one isoflav-2-ene, and two furanoisoflavonoids (Figure 4A). To further identify differential compounds, differential metabolites (DEMs) were selected based on an FC ≥ 2 threshold. The results revealed that 120 flavonoids are commonly present in both BF7 and DWH, 102 flavonoids are commonly present in both BF7 and DWH, and 18 flavonoids are commonly present in both DWH and ANK (Figure 4B and Figure S7). Moreover, the enrichment level of these flavonoids differs significantly among the three bulbs, with BF7 exhibiting a notably higher abundance, which is in accordance with the results of the total flavonoid content (Figure 4C).

**Figure 5:**
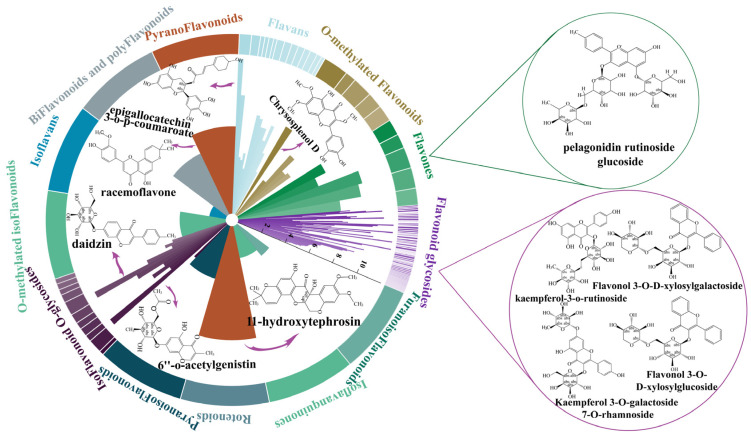
**Circular bar plots depicting the classification and distribution of specific flavonoids in BF7.** The colored segments represent various flavonoid subclasses, such as flavonoid glycosides, flavones, flavans, pyranoflavonoids, and others. The length of each bar within the segments corresponds to the relative abundance of each subclass. The inset displays the molecular structures of the flavonoids, offering a detailed view of the compounds that are enriched in BF7. Arrows indicate the classification direction within each flavonoid.

To further investigate the specificity of flavonoids in BF7, we selected 119 flavonoids from BF7 with a threshold of FC ≥ 2 and compared their abundance. Notably, there are 4 flavonoid glycosides (catechin 3-o-rutinoside, kaempferol 3-O-galactoside 7-O-rhamnoside, kaempferol 3-o-rutinoside, naringin 6″-malonic acid) specifically detected in BF7 (Table S1). Importantly, four flavonoid glycosides (kaempferol-3-o-rutinoside, flavonol 3-O-D-xylosylgalactoside, flavonol 3-O-D-xylosylglucoside and kaempferol 3-O-galactoside 7-O-rhamnoside), two flavones (pelargonidin rutinoside glucoside and velloquercetin-dimethyl ether), one flavan (epigallocatechin 3-o-p-coumaroate), one pyranoflavonoids (racemoflavone), two isoflavonoid O-glycosides (daidzin and 6″-o acetylgenistin) and one rotenoid (11-hydroxytephrosin) are highly abundant (Figure 5). Among these, kaempferol-3-o-rutinoside and kaempferol 3-O-galactoside 7-O-rhamnoside are specifically found in BF7 and exhibit remarkably high abundance, and thereby can be considered signature flavonoids of BF7.

**Figure 6:**
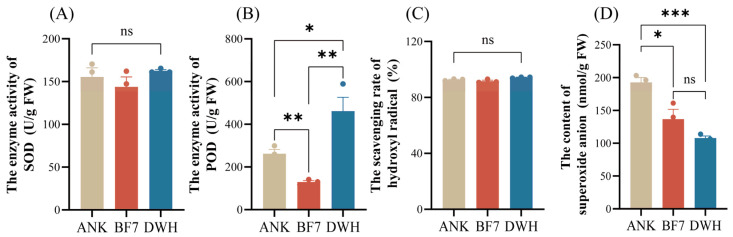
**The enzyme activities and antioxidant capacities of ANK, BF7 and DWH.** (**A**) The enzyme activity of SOD (Superoxide Dismutase), expressed as U/g FW, shows no significant difference (ns) between the groups. (**B**) The enzyme activity of POD (Peroxidase), expressed as U/g FW, significantly differs between BF7 and DWH (indicated by **), with BF7 showing higher activity. (**C**) The scavenging rate of hydroxyl radical shows no significant difference (ns) among the three groups. (**D**) The production rate of superoxide anions in BF7 and DWH did not differ significantly, but both were notably lower than that in ANK, as indicated by * and ***. Asterisks indicated the statistical significance: * *p* < 0.05, ** *p* < 0.01, *** *p* < 0.001 (Student’s *t*-test).

Additionally, to further explore the correlation between flavonoid compounds and antioxidant activity, we assessed four antioxidant indicators (Figure 6). Results showed no significant difference in SOD enzyme activity among the three lily varieties. However, POD activity was lowest in BF7, indicating that its antioxidant capacity is not attributed to protective enzymes. The production rate of superoxide anions in BF7 and DWH did not differ significantly, but both were notably lower than that in ANK. This result further indicates that the strong antioxidant capacity of BF7 may be attributed to its abundant flavonoids.

## 4. Discussion

Phytohormonomics, which involves the profiling of multiple phytohormones through targeted metabolomics, is gaining increasing attention [2,21,22]. Like other omics fields, phytohormonomics focuses on both the qualitative and quantitative analysis of hormone profiles in plant samples. As such, it serves as a critical indicator of plant developmental processes and trajectories. ANK, BF7 and DWH are three LA hybrid lily varieties. Among them, DWH is renowned for its excellent physiological quality, and BF7 was identified in the field and has drawn our attention due to its strong stress resistance. And in terms of bulb size, the order is BF7 > DWH > ANK. To elucidate the underlying reasons for the strong stress resistance of BF7, we performed a targeted analysis of the hormone profiles in the bulbs of these three lily varieties. LC-MS/MS analysis revealed distinct hormone signatures among the three lily cultivars that matched their known stress tolerance rankings. BF7 (stress-resistant) accumulated 34.3 ± 2.1 ng g^−1^ FW abscisic acid and 22.0 ± 1.5 ng g^−1^ jasmonic acid precursor, representing 2.3-fold and 1.9-fold increases over ANK, respectively (Table 1). Conversely, total gibberellins in BF7 were 48% lower than in ANK (1.9 vs. 3.6 ng g^−1^ FW). The high-yield cultivar ANK displayed the inverse pattern—lowest ABA (9.4 ± 1.2 ng g^−1^) but highest bioactive GA_1_ (6.0 ± 0.3 ng g^−1^). DWH was distinguished by elevated cytokinin levels (18.1 ± 1.3 ng g^−1^ trans-zeatin riboside), 2.1-fold higher than both BF7 and ANK. These hormone concentrations directly matched the cultivars’ field performance characteristics: BF7’s documented stress resistance, ANK’s rapid growth habit, and DWH’s superior bulb quality.

The bulbs of the three lily species were collected in early July, when the flowering period had completely ended. Typically, lily plants transition into a bulb enlargement phase post-flowering. However, the extended period of high temperatures (with daily maximums above 35 °C for five consecutive days) resulted in the above-ground stems and leaves of lilies gradually turning yellow and wither, which is a sign that the plant has ceased growth and entered dormancy [23]. This suggests that the lilies entered a brief dormancy phase triggered by high temperatures and further supported by our hormone profiling data. In this study, most auxins, CKs and GAs were detected at a lower level, reflecting a state of limited plant growth and development during this period, which is consistent with the plant’s growth status. ABA plays a key role in various important physiological processes, particularly as a crucial regulator of drought, and is involved in regulating plant growth, development, and responses to various environmental stresses [24]. In this study, ABA content in BF7 and DWH is significantly higher than in ANK, suggesting that BF7 and DWH may exhibit stronger stress resistance [25]. Importantly, ABA-GE was only found in BF7. ABA-GE is one of the most abundant conjugated forms of ABA, but it exhibits little or no biological activity on its own [26]. 

Many studies provide strong evidence that compartmentalization of ABA-GE acts as a reservoir, enabling the rapid, localized accumulation of ABA [27,28,29]. Additionally, high ABA-GE concentrations are thought to enhance plants’ stress tolerance [30,31]. The abundant ABA-GE in BF7 could form a dynamic slow-release reservoir, which continuously releases active ABA to effectively regulate stress response pathways. The specific enrichment of ABA-GE in BF7 bulbs reveals a potential stress resistance mechanism in BF7, where ABA is promptly released through the hydrolysis of ABA-GE in response to environmental stress. This mechanism is likely one of the key factors contributing to BF7’s remarkable stress tolerance under adverse conditions. The slow-release property ensures timely ABA supply while avoiding the metabolic toxicity caused by high concentrations of ABA, providing robust support for BF7’s adaptation to complex stress environments.

JA is another crucial hormone that enables plants to rapidly respond to both abiotic and biotic stresses [32]. In plants, there are two main JA biosynthesis pathways. The first pathway involves the conversion of 18:3 fatty acid to OPDA through the catalysis of LOX, AOS, and AOC. OPDA is then converted to OPC-8 in the peroxisome, followed by three rounds of β-oxidation to form JA. Additionally, OPDA can be reduced to dnOPDA, which directly forms OPC-6 and undergoes two rounds of β-oxidation to produce JA. The second pathway involves the catalysis of 16:3 fatty acid to form dnOPDA, which is then converted to OPC-6 and undergoes two rounds of β-oxidation to yield JA [32]. In this process, OPC-6 is essential. However, OPC-6 has only been found in BF7, which suggests that BF7 may possess the potential for a rapid JA response, potentially contributing to its enhanced disease resistance. ABA and JA are two key plant hormones that interact either synergistically or antagonistically to help the plant cope with environmental stress [25]. In this study, the precursor of JA is found in abundance and independently in BF7, which confers the ability to rapidly respond to stress. This could be another key factor underlying BF7’s enhanced stress resistance.

To further evaluate the correlation between hormone profiles and intrinsic physiological phenotypes, we measured eight physiological indicators, including nutrients and secondary metabolites. The profiles of these indicators are generally consistent with the hormonomics profiles. Overall, the hormone profiles of DWH and BF7 are superior, with DWH emphasizing growth and development, while BF7 focuses more on stress resistance (Figure 3). This distinction is also reflected in their physiological phenotypes. Notably, BF7 exhibits exceptional total antioxidant capacity, a composite indicator that reflects the collective performance of various antioxidants and serves as a powerful marker of stress resistance (Figure 3I). Notably, the antioxidant capacity of BF7 does not result from high activity of protective enzymes (Figure 6). Furthermore, the total flavonoid content in BF7 was significantly higher than that in ANK and DWH. (Figure 3F), suggesting that numerous flavonoids may contribute to its strong antioxidant activity. Subsequent non-targeted metabolite analysis further supported it, revealing that BF7 bulbs accumulate a significant number of flavonoids (Figure 4C). Flavonoids, a group of plant-derived secondary metabolites, are widely studied for their potent antioxidant, anti-inflammatory, and pharmacological properties. Numerous pharmacological studies have demonstrated that these flavonoids exhibit strong antioxidant activity [33,34,35,36].

Untargeted metabolomic analysis revealed four flavonoid glycosides that exhibited significant and specific enrichment in the BF7 group (Figure 5). Notably, kaempferol-3-o-rutinoside and kaempferol 3-O-galactoside 7-O-rhamnoside were uniquely enriched in BF7. Kaempferol is an important flavonoid which exhibits potent antioxidant activity with an exceptionally low IC50 value of 0.5 μM [37]. Kaempferol can directly chelate Fe^2+^ and Cu^+^ ions, thereby suppressing hydroxyl radical (·OH) generation in Fenton reactions [38], and it primarily occurs in glycosylated forms in plants [39]. Current evidence suggests that flavonoid glycosylation may confer functional advantages through two primary mechanisms: (1) enhancing aqueous solubility and chemical stability via hydrophilic sugar moieties, thereby facilitating long-distance transport and storage in planta; (2) serving as a precursor reservoir, enabling rapid release of free flavonoid aglycones (e.g., catechin) under oxidative stress via β-glucosidase-mediated hydrolysis of glycosidic bonds, thereby establishing a dynamic antioxidant defense system [36]. However, certain glycosylation modifications may further enhance antioxidant capacity. For instance, UGT78A14-catalyzed glycosylation products of kaempferol and quercetin demonstrate superior ROS scavenging activity compared to their aglycone counterparts [40]. Many studies have demonstrated that kaempferol-3-O-rutinoside possesses potent antioxidant capacity [41,42]. A study demonstrated that kaempferol-3-O-glucoside exhibited notable antioxidant activity, with an IC50 value approximately 25% of that of the potent reference antioxidant ascorbic acid [43]. Extracts of *Crassocephalum crepidioides*, *Ginkgo biloba* and soybean, all rich in kaempferol glycosides, have been demonstrated to exhibit potent antioxidant activity in vivo [44,45,46]. Furthermore, numerous additional flavonoids exhibited marked enrichment in BF7, including daidzin, epigallocatechin 3-O-p-coumaroate, 6″-O-acetylgenistin, 11-hydroxytephrosin, etc., all of which have been well-documented in numerous studies to exhibit potent antioxidant capacities. For example, epigallocatechin 3-O-p-coumaroate can extend the lifespan of *Caenorhabditis elegans* by reducing ROS levels [36]. Daidzin, an isoflavonoid O-glycoside, exhibits a strong inhibitory effect on lipid peroxidation [47,48]. Additionally, compelling evidence shows that flavonols in tomatoes can significantly reduce ROS levels, thereby improving drought and heat tolerance [49,50]. However, in our study, the activity of SOD and POD enzymes was at a relatively low level, suggesting that BF7 does not rely on the activity of protective enzymes. Instead, the flavonoid compounds that are abundantly present in BF7 may constitute an antioxidant reservoir, continuously maintaining a dynamic balance of the oxidative state. Especially the flavonoids with higher concentrations, their contribution to the total antioxidant capacity and their biological functions will be the focus of our further research.

This analysis was restricted to three lily cultivars at a single developmental stage. The hormone-phenotype relationships identified here require validation across additional genotypes and developmental stages before broad application. Field trials under variable environmental conditions are needed to confirm that the ABA/JA markers remain predictive of stress tolerance in commercial cultivation settings. The targeted LC-MS/MS approach focused on 12 major plant hormones but did not capture hormone conjugates, precursors, or catabolites that might influence the regulatory networks. Additionally, the physiological assays measured endpoint responses rather than dynamic changes, limiting insights into temporal hormone-phenotype relationships. Despite these constraints, the quantitative relationships between hormone profiles and both physiological performance and flavonoid accumulation provide a foundation for hormone-informed lily breeding strategies.

## 5. Conclusions

**Figure 7:**
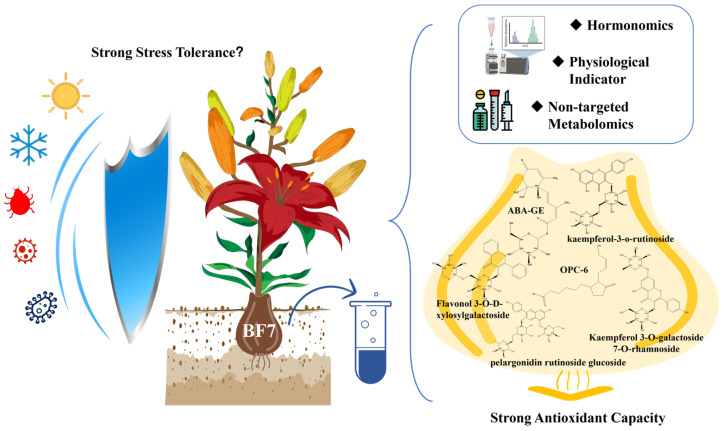
**Overview of the Experiment Diagram.** BF7, a lily variety demonstrating remarkable stress-resistant traits under field conditions. To elucidate the intrinsic mechanisms underlying its stress tolerance, this study employed targeted mass spectrometry analysis to conduct a systematic hormone profiling of BF7, Annika (a high-yielding standard cultivar) and DWH (known for superior physiological quality), aiming to identify key hormonal signatures associated with stress resistance and metabolic regulation. By correlating hormonal expression patterns with physiological parameters and secondary metabolite accumulation, this research provides molecular-level insights into the stress-resistant mechanisms of BF7, offering a theoretical foundation for marker-assisted breeding to develop stress-tolerant lily cultivars.

This work demonstrates that cultivar-specific hormonal configurations underpin both physiological performance and flavonoid accumulation in lily bulbs. Targeted LC-MS/MS profiling showed that the stress-resistant cultivar BF7 contained 3.63-fold higher abscisic acid than the high-yield standard ANK, and specific accumulation of ABA-GE and OPC-6 in BF7, suggesting the potential existence of unique secondary metabolic pathways. Subsequent physiological assays and untargeted metabolomics further confirmed the distinctive accumulation of various flavonoid compounds in BF7 bulbs, with most flavonoids existing in glycosylated forms. Taken together, our data support a mechanistic model in which an ABA/JA-rich hormonal milieu primes antioxidant defences and stimulates flavonoid biosynthesis, thereby conferring superior stress resilience (Figure 7). These results offer valuable insights into the potential use of hormones to promote plant growth and offer actionable targets for hormone-informed selection in lily improvement programs. Future studies should validate these markers across broader genetic backgrounds and developmental stages and will focus on elucidating the key molecular regulatory networks involved, with the objectives of identifying novel targets for stress-resistant breeding and providing valuable references for lily cultivar improvement.

## Figures and Tables

**Table 1:** The hormones in three lily bulbs.

Number	Name	Abbreviation	Class	The Content of BF7 (ng/g)	The Content of ANK (ng/g)	The Content of DWH (ng/g)
1	Abscisic acid	ABA	ABA	34.26	9.42	30.38
2	ABA-glucosyl ester	ABA-GE	ABA	112.28	0	0
3	Abscisic aldehyde	ABA-ald	ABA	30.81	0	54.51
4	Indole-3-acetic acid	IAA	Auxin	4.77	4.98	5.41
5	Indole-3-carboxaldehyde	ICAld	Auxin	0.33	0.62	0.54
6	Indoleacetyl glutamic acid	IAA-Glu	Auxin	0.34	0.21	12.79
7	3-Indoleacetonitrile	IAN	Auxin	0	0.21	0
8	2-oxindole-3-acetic acid	OxIAA	Auxin	16.50	10.64	14.49
9	Indole-3-acetyl-L-aspartic acid	IAA-Asp	Auxin	0.61	0.71	43.59
10	Indole	Indole	Auxin	330.92	0	1878.69
11	N6-isopentenyladenine	IP	Auxin	0.01	0.01	0.01
12	Indole-3-acetyl-L-leucine methyl ester	IAA-Leu-Me	Auxin	0.03	0	0
13	L-tryptophan	TRP	Auxin	348.04	345.31	5639.49
14	Methyl indole-3-acetate	MEIAA	Auxin	0.09	0.10	0.16
15	cis-Zeatin riboside	cZR	CK	0.16	0.23	0.13
16	N6-Isopentenyl-adenine-7-glucoside	iP7G	CK	0	0.16	0
17	trans-Zeatin-O-glucoside	tZOG	CK	0.47	0.82	0.68
18	cis-Zeatin-O-glucoside riboside	cZROG	CK	0.19	0.39	0.14
19	cis-Zeatin-9-glucoside	cZ9G	CK	0	0.8	0
20	para-Topolin	pT	CK	0.08	0.10	0.13
21	meta-Topolin riboside	mTR	CK	1.46	0.14	0.08
22	Kinetin riboside	KR	CK	0	0	0.15
23	ortho-Topolin riboside	oTR	CK	0	0.16	0
24	N6-Benzyladenine-7-glucoside	BAP7G	CK	0.09	0.14	0
25	Kinetin-9-glucoside	K9G	CK	1.43	0	0
26	2-Methylthio-cis-zeatin riboside	2MeScZR	CK	0.07	0	0.06
27	N6-isopentenyladenosine	IPR	CK	0.08	0.06	0
28	trans-Zeatin riboside	tZR	CK	0	0	0.08
29	cis-Zeatin riboside monophosphate	cZRMP	CK	1.14	1.12	0.89
30	N-6-iso-pentenyladenosine-5′-monophosphate	iPRMP	CK	2.80	2.39	3.03
31	9-Ribosyl-trans-zeatin 5′-monophosphate	tZRMP	CK	1.29	0	1.97
32	Gibberellin A1	GA1	GA	0	6.06	27.05
33	Gibberellin A3	GA3	GA	8.27	1.26	7.48
34	Gibberellin A9	GA9	GA	0	0.51	0
35	Gibberellin A15	GA15	GA	24.35	14.88	47.59
36	Gibberellin A20	GA20	GA	1.94	0	0
37	Gibberellin A24	GA24	GA	1.89	1.26	4.27
38	Gibberellin A29	GA29	GA	38.10	32.98	52.91
39	Gibberellin A34	GA34	GA	2.58	3.77	3.71
40	Gibberellin A53	GA53	GA	0	1.06	0
41	Jasmonic Acid	JA	JA	3.97	2.55	9.93
42	Methyl jasmonate	MEJA	JA	1.59	0	1.75
43	Jasmonoyl-L-isoleucine	JA-ILE	JA	1.35	0.23	1.38
44	Cis (+)-12-Oxophytodienoic acid	OPDA	JA	114.65	72.86	88.84
45	N-[(−)-Jasmonoyl]-(L)-valine	JA-Val	JA	0.02	0	0.04
46	3-oxo-2-(2-(Z)-Pentenyl) cyclopentane-1-hexanoic acid	OPC-6	JA	22.00	0	0
47	Salicylic Acid	SA	SA	98.83	107.82	99.92
48	Salicylic acid 2-O-β-Glucoside	SAG	SA	82.74	111.52	49.73
49	L-Phenylalanine	Phe	SA	5602.30	6972.02	13,131.27

## Data Availability

Data are contained within the article and Supplementary Materials.

## Supplementary Materials

The following supporting information can be downloaded at: https://www.mdpi.com/article/10.3390/antiox14070862/s1, Supplementary Materials S1: including Figures S1–S17, among them, Mass spectra of flavonoids; Supplementary Materials S2: Mass spectra of Hormones; Table S1: Hormones and flavonoids detected in ANK, BF7 and DWH.

## Abbreviations

The following abbreviations are used in this manuscript:

BF7	Bifeng 7
ANK	Anika
DWH	Diwanghuang
LC-MS	Liquid Chromatography-Mass Spectrometry
UPLC	Ultra Performance Liquid Chromatography
MS	Mass Spectrometry
LIT	Linear ion trap
ESI	Electrospray Ionization
FRAP	Ferric Reducing Antioxidant Power
NBT	Nitroblue Tetrazolium
SOD	Superoxide Dismutase
POD	Peroxidase
TIC	Total Ion Chromatogram
ANOVA	One-way analysis of variance
RT	Retention time
PI	Peak intensity
CV	Coefficient of Variation
PCA	Principal Component Analysis
ABA	Abscisic acid
ABA-GE	ABA-glucosyl ester
ABA-ald	Abscisic aldehyde
IAA	Indole-3-acetic acid
ICAld	Indole-3-carboxaldehyde
IAA-Glu	Indoleacetyl glutamic acid
IAN	3-Indoleacetonitrile
OxIAA	2-oxindole-3-acetic acid
IAA-Asp	Indole-3-acetyl-L-aspartic acid
IP	N6-isopentenyladenine
IAA-Leu-Me	Indole-3-acetyl-L-leucine methyl ester
TRP	L-tryptophan
MEIAA	Methyl indole-3-acetate
cZR	Cis-Zeatin riboside
iP7G	N6-Isopentenyl-adenine-7-glucoside
tZOG	Trans-Zeatin-O-glucoside
cZROG	Cis-Zeatin-O-glucoside riboside
cZ9G	Cis-Zeatin-9-glucoside
pT	Para-Topolin
mTR	Meta-Topolin riboside
KR	Kinetin riboside
oTR	Prtho-Topolin riboside
BAP7G	N6-Benzyladenine-7-glucoside
K9G	Kinetin-9-glucoside
2MeScZR	2-Methylthio-cis-zeatin riboside
IPR	N6-isopentenyladenosine
tZR	Trans-Zeatin riboside
cZRMP	Cis-Zeatin riboside monophosphate
iPRMP	N-6-iso-pentenyladenosine-5′-monophosphate
tZRMP	9-Ribosyl-trans-zeatin 5′-monophosphate
GA1	Gibberellin A1
GA3	Gibberellin A3
GA9	Gibberellin A9
GA15	Gibberellin A15
GA20	Gibberellin A20
GA24	Gibberellin A24
GA29	Gibberellin A29
GA34	Gibberellin A34
GA53	Gibberellin A53
JA	Jasmonic Acid
MEJA	Methyl jasmonate
JA-ILE	Jasmonoyl-L-isoleucine
OPDA	Cis (+)-12-Oxophytodienoic acid
JA-Val	N-[(-)-Jasmonoyl]-(L)-valine
OPC-6	3-oxo-2-(2-(Z)-Pentenyl) cyclopentane-1-hexanoic acid
SA	Salicylic Acid
SAG	Salicylic acid 2-O-β-Glucoside
Phe	L-Phenylalanine
