# Supplementary material for: Research on the Hormonomics of Three Lilium Species and Their Flavonoid Diversification and Specificity
Source: Antioxidants (Basel). 2025 Jul 14;14(7):862. doi: 10.3390/antiox14070862 (PMC12291843; doi:10.3390/antiox14070862)
Supplement: Supplementary file 1 [file antioxidants-14-00862-s001.zip › Supplementary materials_1.pdf]

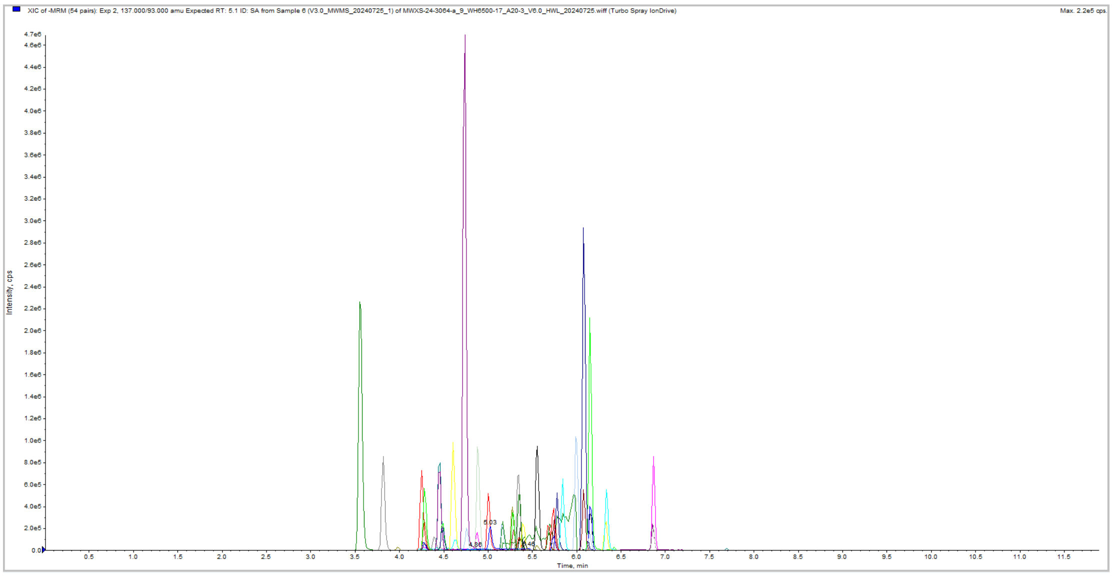

Figure S1. The total ion current (TIC) curves.

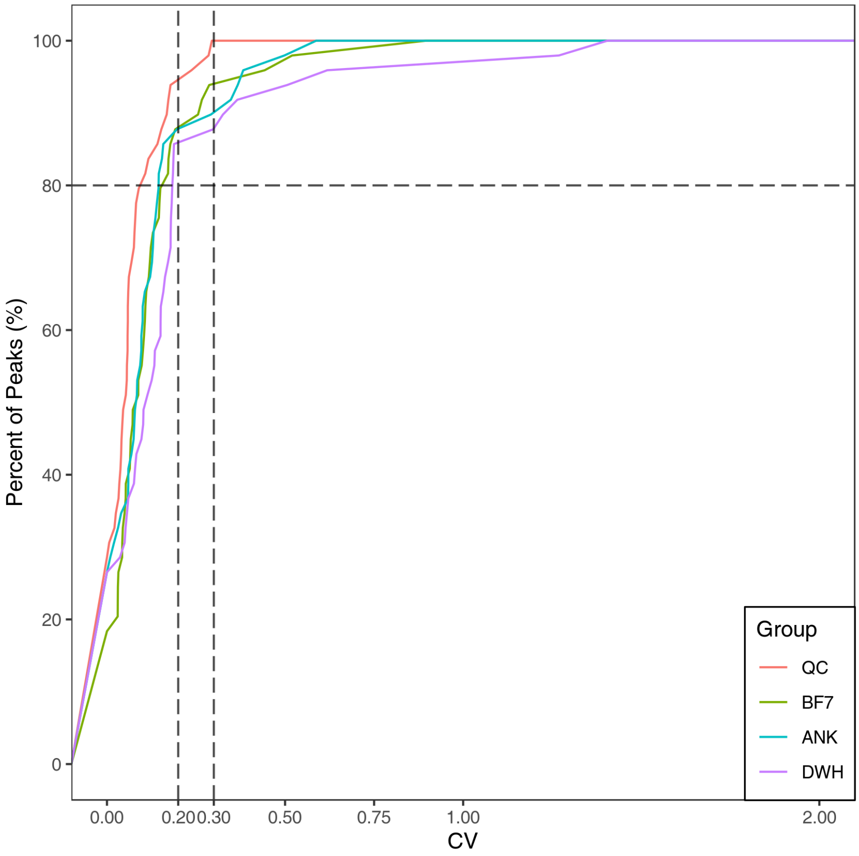

Figure S2. The Coefficient of Variation (CV) among each group of ANK, BF7 and DWH.

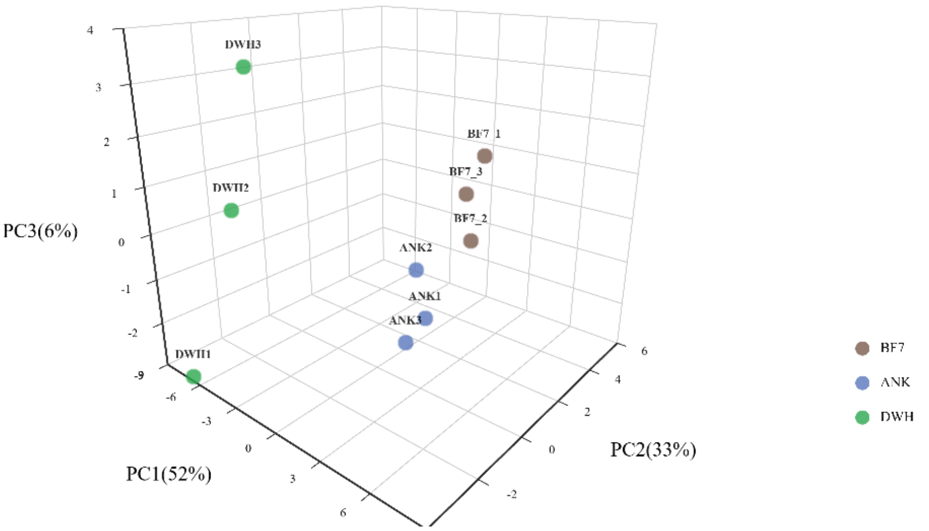

Figure S3. The principal component analysis (PCA).

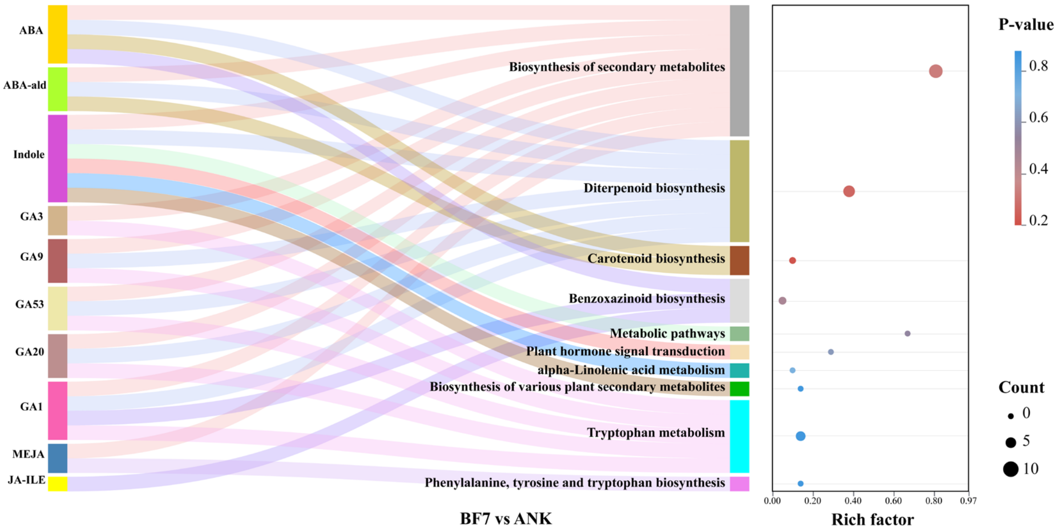

Figure S4. KEGG Sankey enrichment analysis for difference hormones of BF7 vs ANK group. The figure consists of a Sankey diagram and an enrichment bubble plot. In the Sankey diagram, the hormone names on the left side represent differential hormones in the BF7 vs ANK comparison group, while the right side displays their corresponding KEGG pathways, with the curves illustrating the association relationships. The bubble plot on the right demonstrates the enrichment levels of these pathways.

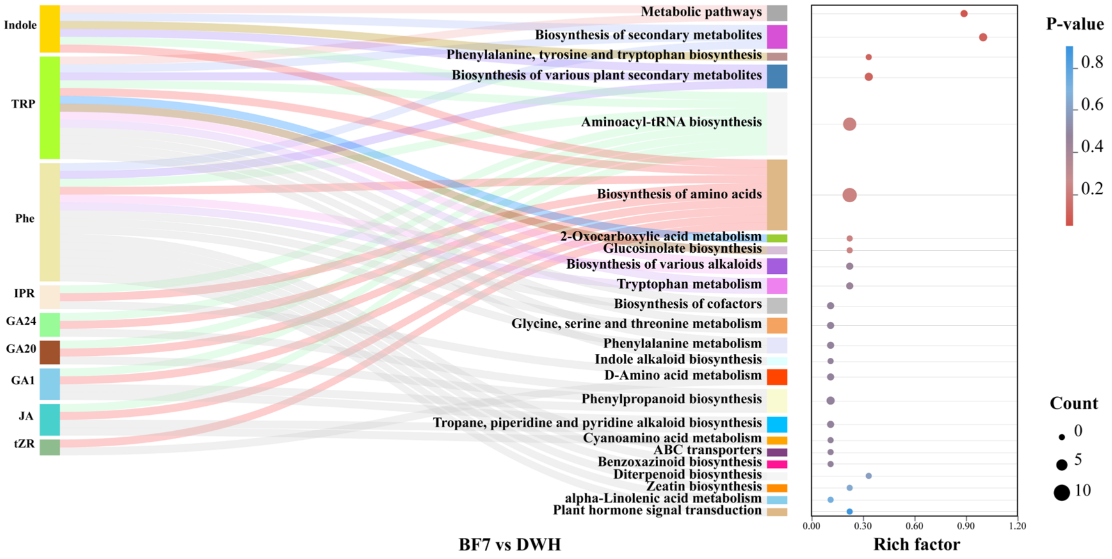

Figure S5. KEGG Sankey enrichment analysis for difference hormones of BF7 vs DWH group. The figure consists of a Sankey diagram and an enrichment bubble plot. In the Sankey diagram, the hormone names on the left side represent differential hormones in the BF7 vs DWH comparison group, while the right side displays their corresponding KEGG pathways, with the curves illustrating the association relationships. The bubble plot on the right demonstrates the enrichment levels of these pathways.

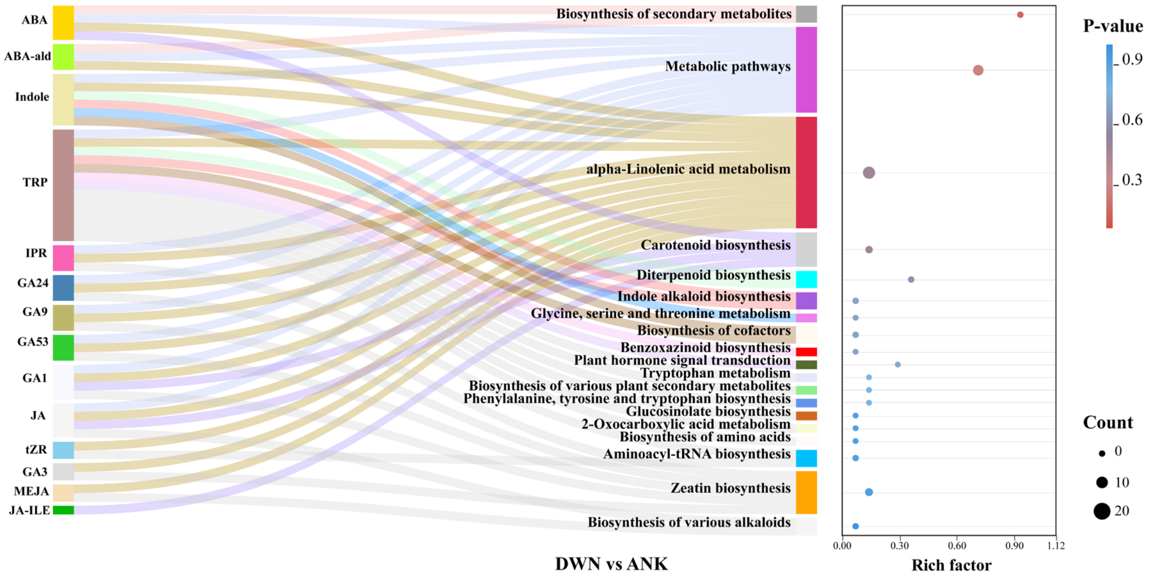

Figure S6. KEGG Sankey enrichment analysis for difference hormones of DWH vs ANK group. The figure consists of a Sankey diagram and an enrichment bubble plot. In the Sankey diagram, the hormone names on the left side represent differential hormones in the DWH vs ANK comparison group, while the right side displays their corresponding KEGG pathways, with the curves illustrating the association relationships. The bubble plot on the right demonstrates the enrichment levels of these pathways.

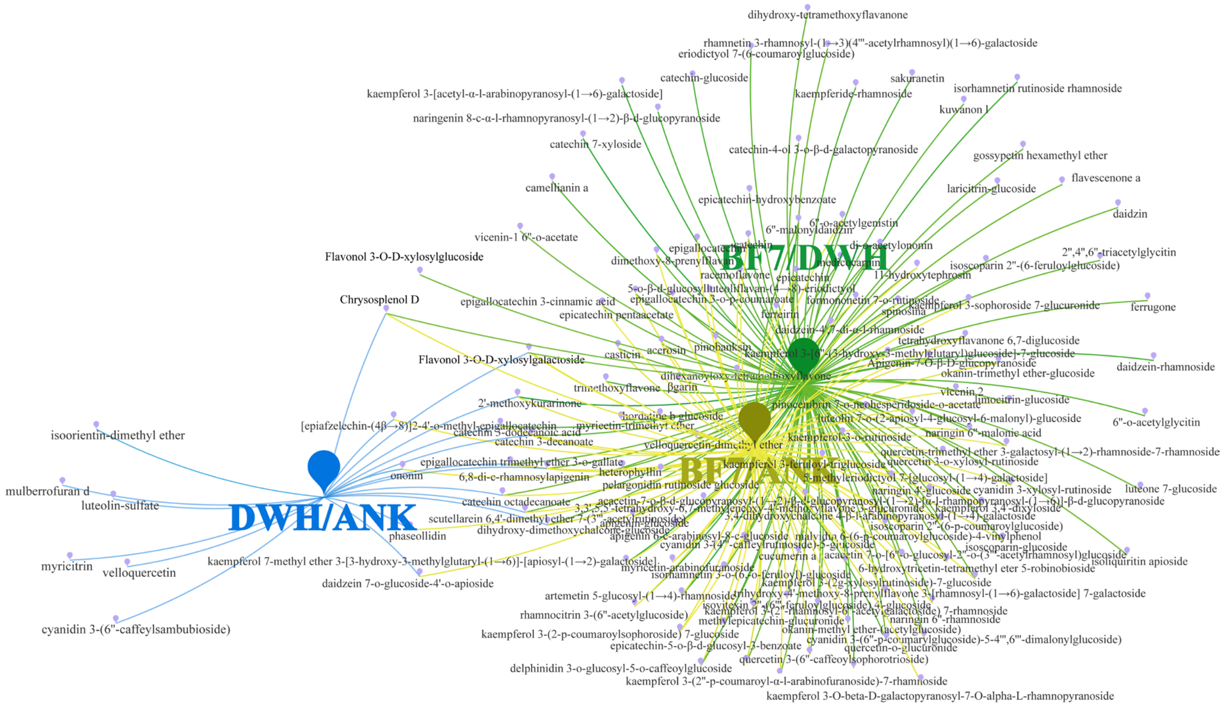

Figure S7. The Venn-network plot presents the association network of differential flavonoids across different lily varieties. The three large nodes represent the three comparison groups: BF7 vs DWH (green node), DWH vs ANK (blue node), and BF7 vs ANK (yellow node). These nodes are linked to various flavonoids, illustrating the distribution of differential flavonoids between the groups. The small purple circles represent the names of the flavonoids, and the color of the connecting lines indicates their relationships across the different comparisons. The connections allow for a clear visualization of the distribution and associations of differential flavonoids among the lily varieties.

1. 6''-o-acetylgenistin

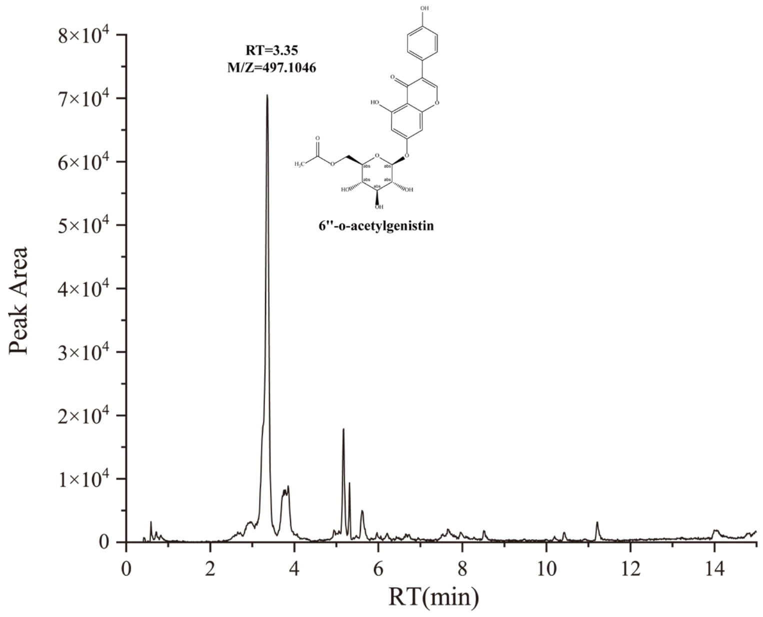

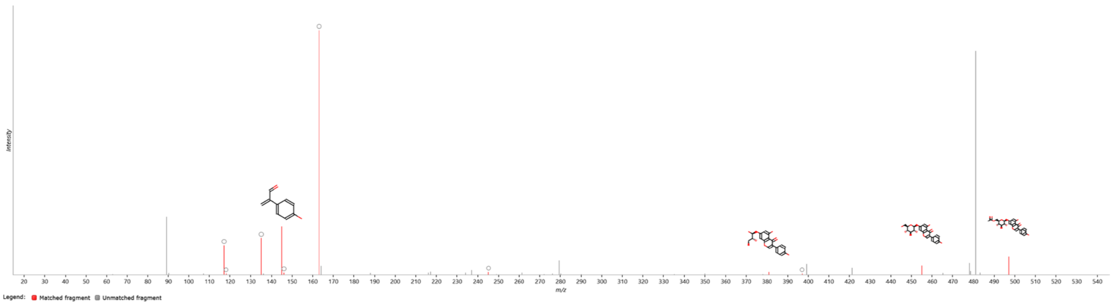

Compound ID	Description	Adducts	Formula	Score	Fragmentation score	Mass error (ppm)
NkMQrIBsXViPgdO	npass:npc224368_/_6"-o-acetylgenistin	M+Na	C23H22O11	40.2	12.1	-1.66

Figure S8. Database search of 6"-o-acetylgenistin at 3.35-min with m/z 497.1046. From top to bottom: the mass spectrum、 secondary mass spectrum and database matching results. The layout of subsequent figures follows the same format.

2. 11-hydroxytephrosin

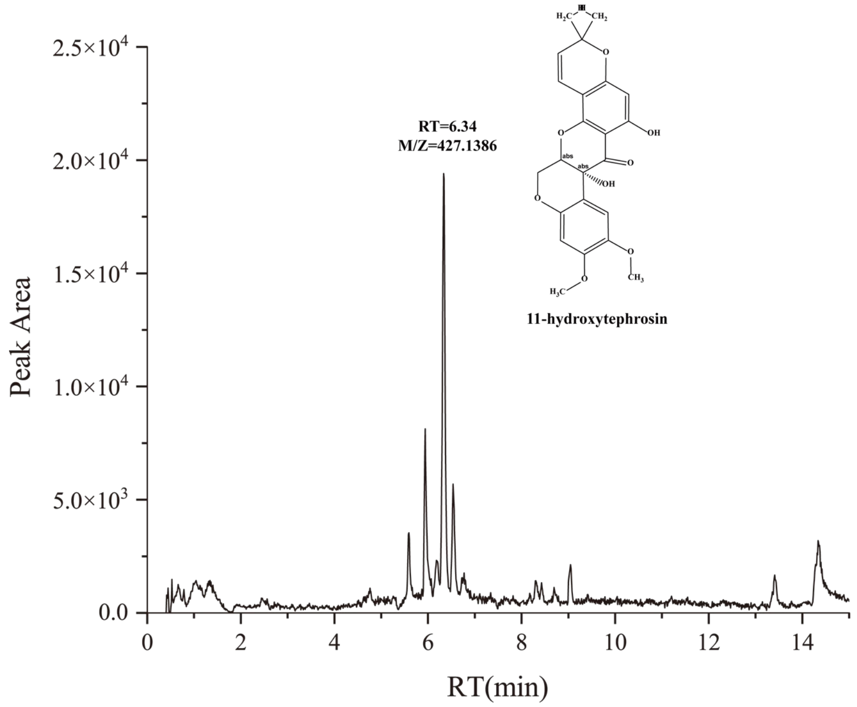

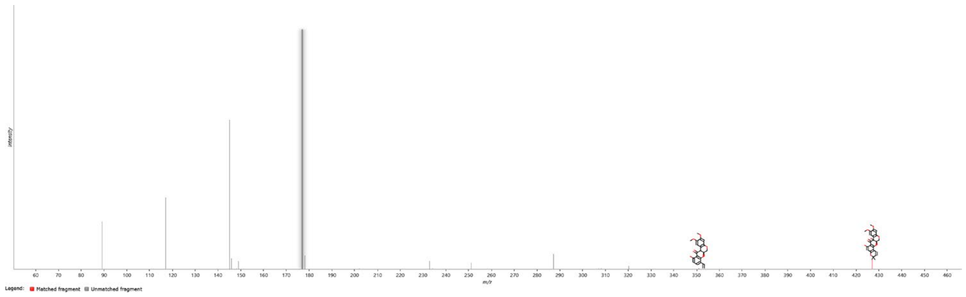

Compound ID	Description	Adducts	Formula	Score	Fragmentation score	Mass error (ppm)
Y@0tK2wRrx7jHEO	knap:c00009591_/_11-hydroxytephrosin	M+H	C23H22O[illegible]	39.5	6.8	-0.27

Figure S9. Database search of 11-hydroxytephrosin at 6.34-min with m/z 427.1386.

3. Chrysosplenol D

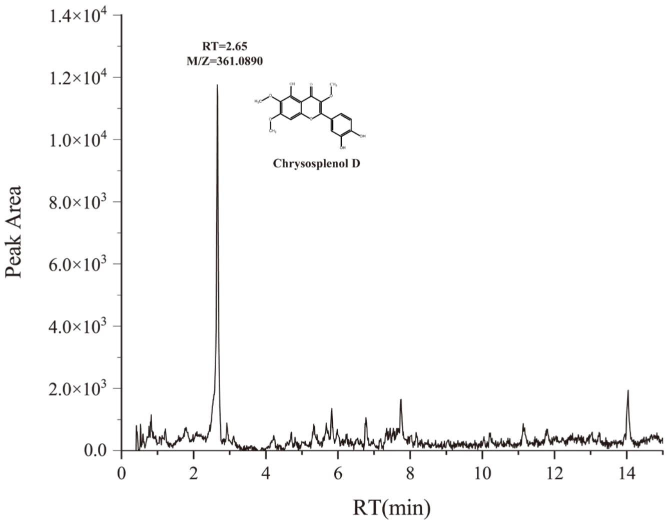

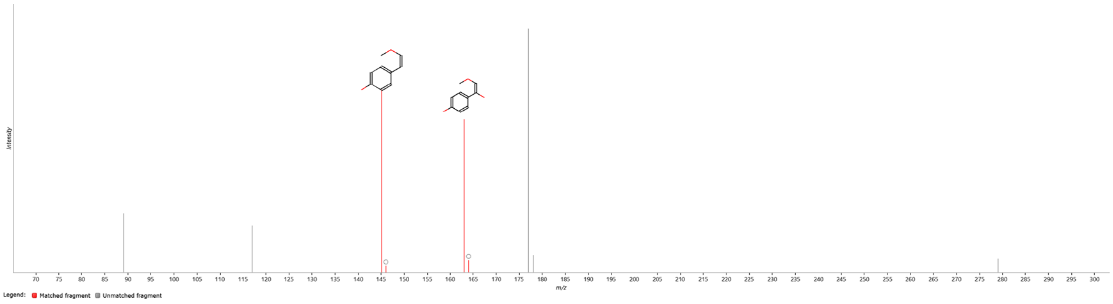

Compound ID	Description	Adducts	Formula	Score	Fragmentation score	Mass error (ppm)	Isotope similarity
xc6wMyqvj1C6Df7	Chrysosplenol D	M+H	C18H16O[illegible]	43.4	34.7	34.7	90.8910197280405

Figure S10. Database search of Chrysosplenol D at 2.65-min with m/z 361.0890.

4. daidzin

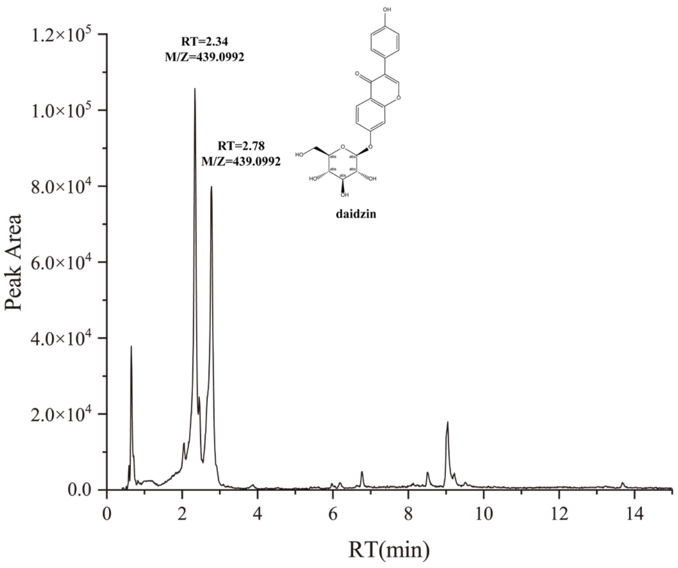

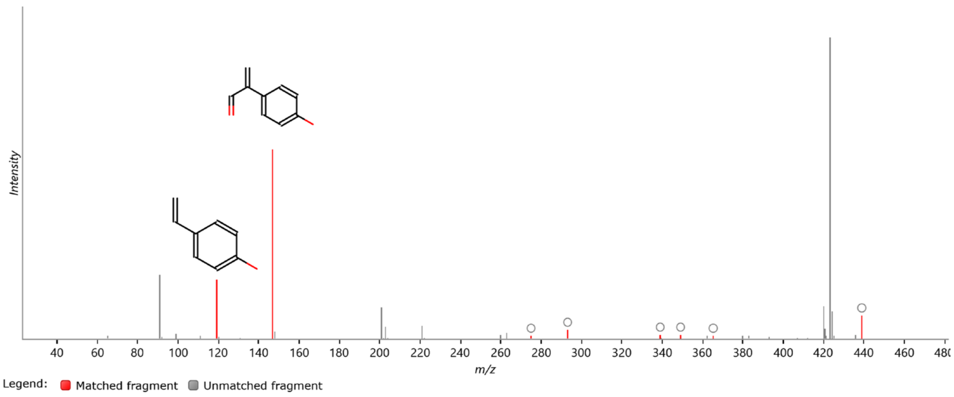

Compound ID	Description	Adducts	Formula	Score	Fragmentation score	Mass error (ppm)
LgkAgZrrHjWke17	foodb:fdb012225_/_daidzin	M+Na	C21H20O9	39.1	8.68	-1.82

Figure S11. Database search of daidzin at 2.34- and 2.78-min with m/z 439.0992.

5. epigallocatechin 3-o-p-coumaroate

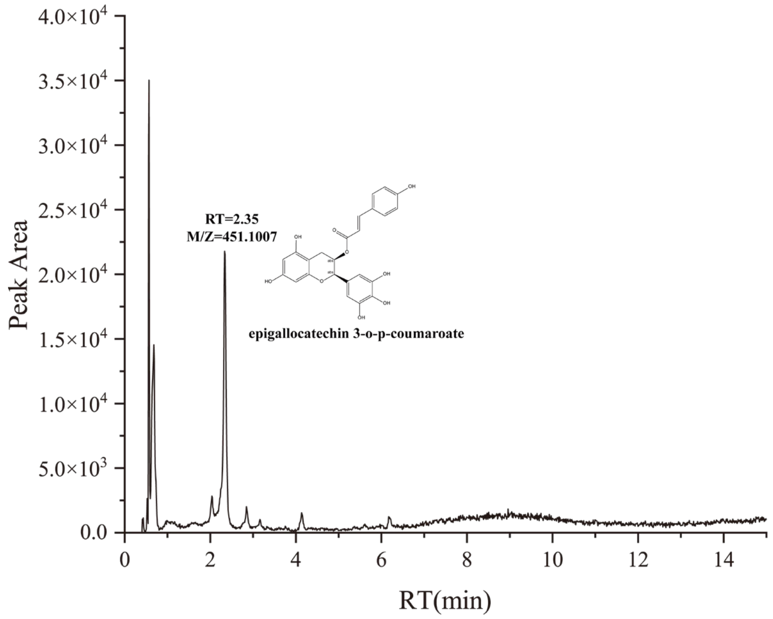

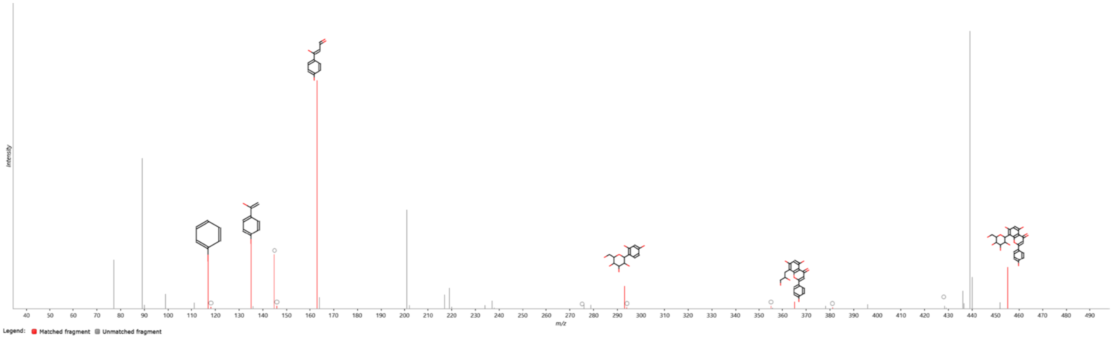

Compound ID	Description	Adducts	Formula	Score	Fragmentation score	Mass error (ppm)
sFMH7LV3OO29JY9	foodb:fdb017701_/_epigallocatechin 3-o-p-coumaroate	M-H	C24H20O[illegible]	36.8	5.48	-6.09

Figure S12. Database search of epigallocatechin 3-o-p-coumaroate at 2.35-min with m/z 451.1007.

6. Flavonol 3-O-D-xylosylgalactoside&Flavonol 3-O-D-xylosylglucoside

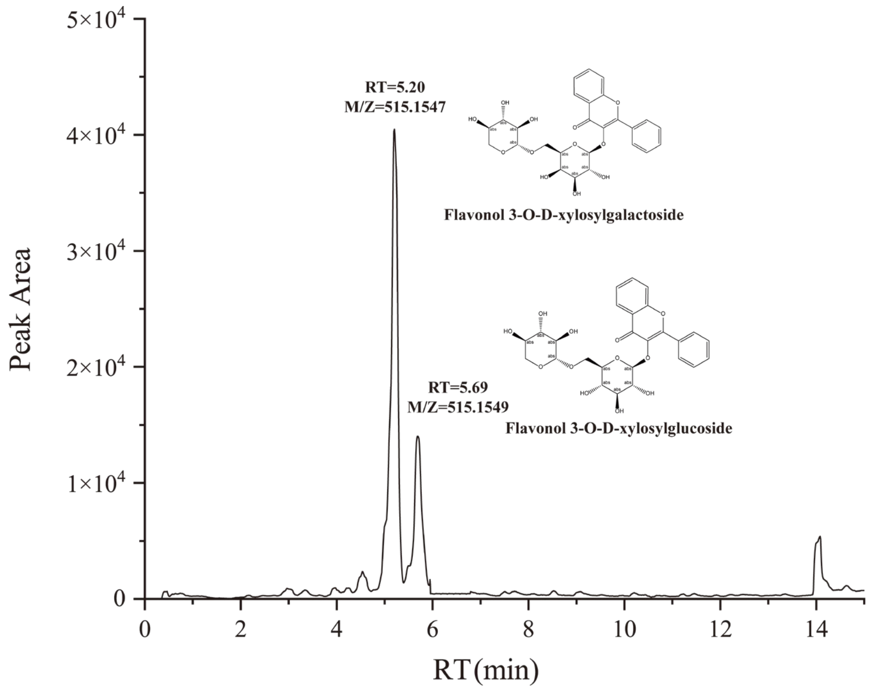

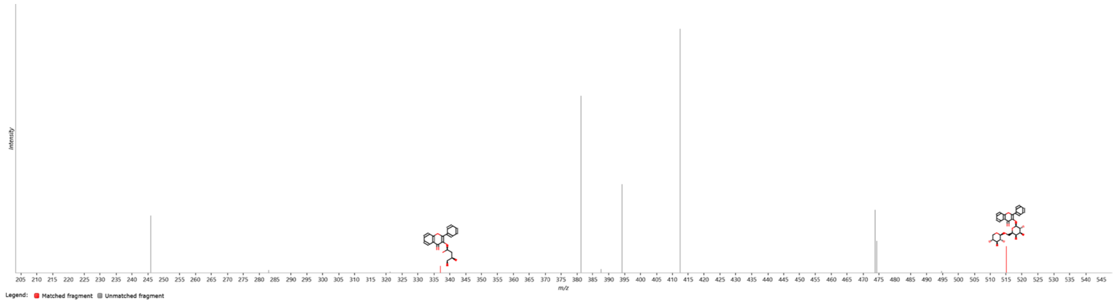

Compound ID	Description	Adducts	Formula	Score	Fragmentation score	Mass error (ppm)	Isotope similarity
jj0XRBwreRbfb-f	Flavonol 3-O-D-xylosylgalactoside	M+H-H2C	C26H28O	38.3	1.61	-0.176868891418753	90.0909809731782

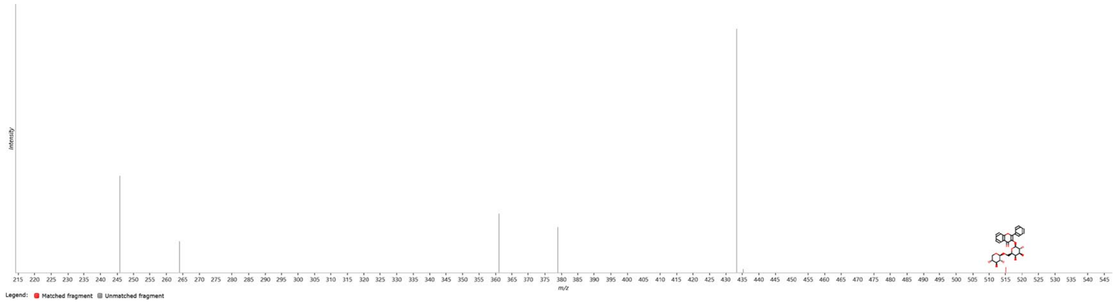

Compound ID	Description	Adducts	Formula	Score	Fragmentation score	Mass error (ppm)	Isotope similarity
wtWSRT8n3GIfl	Flavonol 3-O-D-xylosylglucoside	M+H-H2C	C26H28O	38.1	0	0.169881732633101	90.6296546656293

Figure S13. Database search of Flavonol 3-O-D-xylosylgalactoside at 5.20-min with m/z 515.1547 and Flavonol 3-O-D-xylosylglucoside at 5.69-min with m/z 515.1549.

7. Kaempferol 3-O-galactoside 7-O-rhamnoside

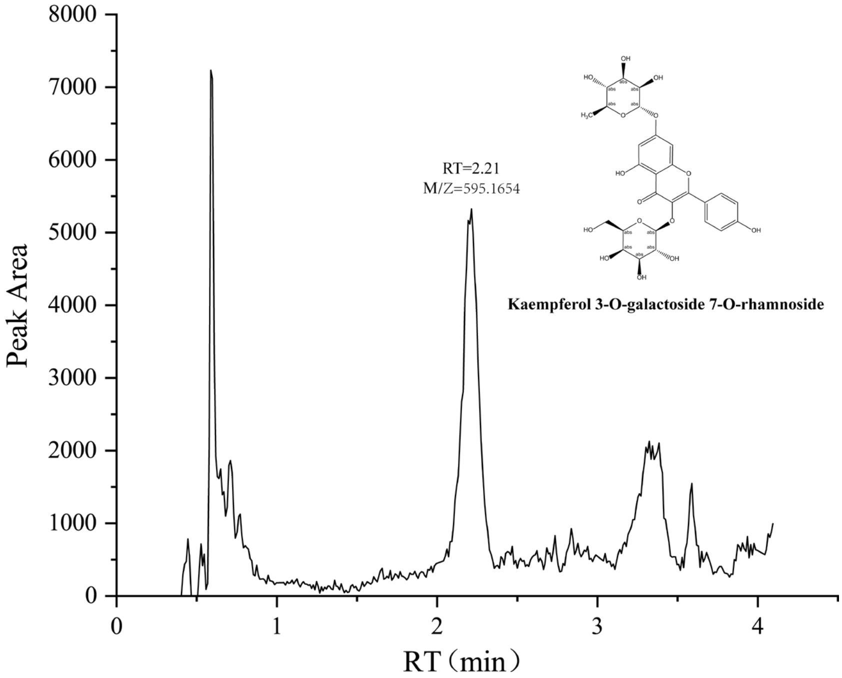

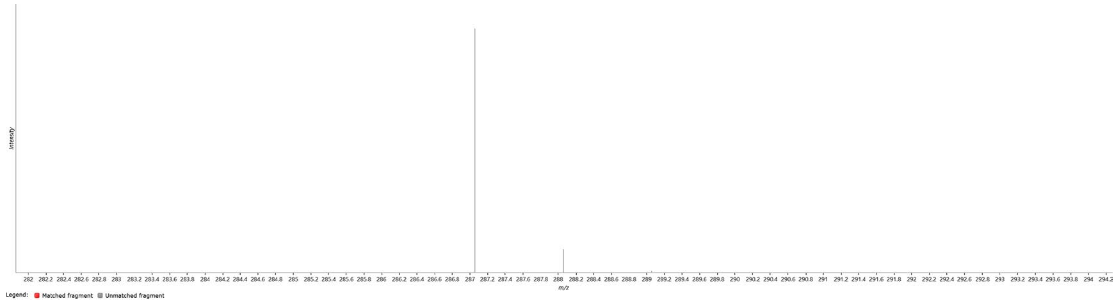

Compound ID	Description	Adducts	Formula	Retention t	Score	Fragmentation score	Mass error (ppm)	Isotope similarity
-u5k3svyl3ZL88S	Kaempferol 3-O-galactoside 7-O-rhamnoside	M+H	C27H30O15		37.5	0	-0.67	88.40099751

Figure S14. Database search of Kaempferol 3-O-galactoside 7-O-rhamnoside at 2.21-min with m/z 595.1654.

8. pelargonidin rutinoside glucoside

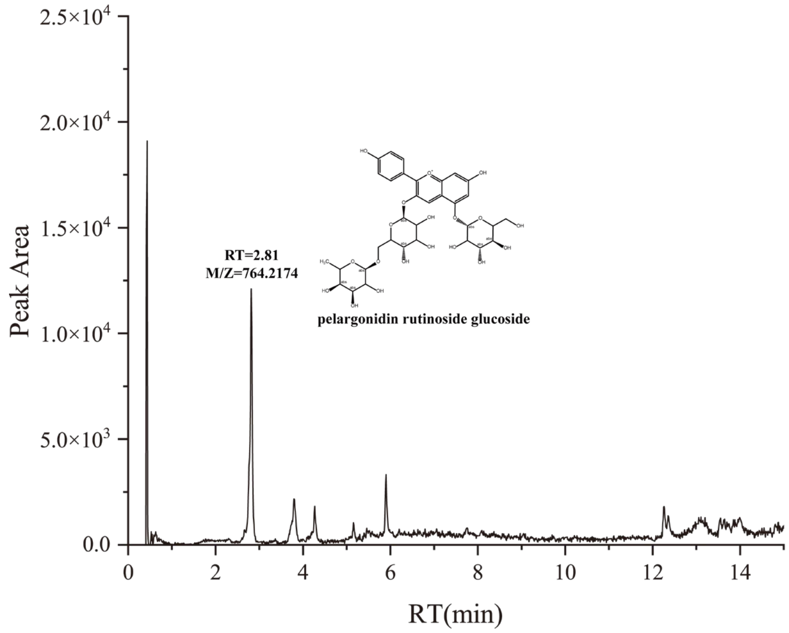

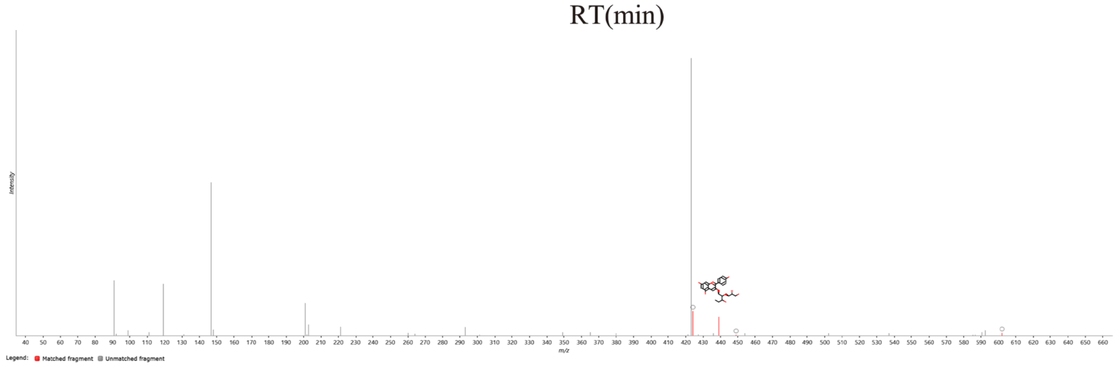

Compound ID	Description	Adducts	Formula	Score	Fragmentation score	Mass error (ppm)
@N25L5RkO2JsITh	knap:c00006644_/_pelargonidin rutinoside glucoside	M+Na	C33H41O	41	20.2	5.42

Figure S15. Database search of pelargonidin rutinoside glucoside at 2.81-min with m/z 764.2174.

9. racemoflavone

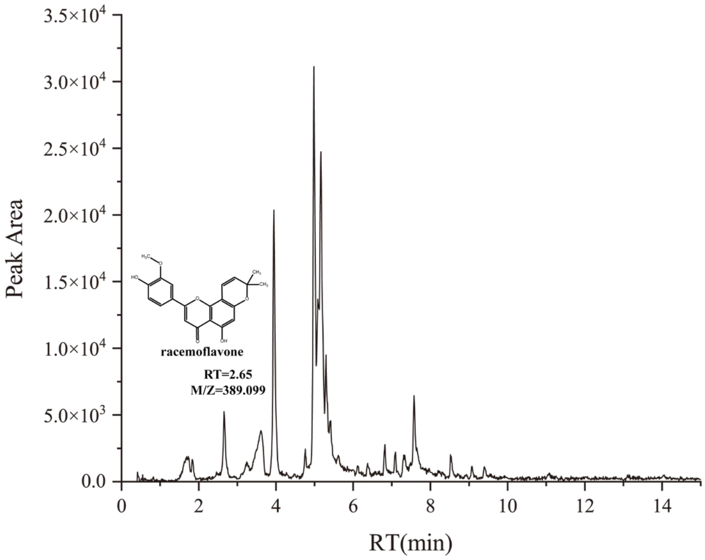

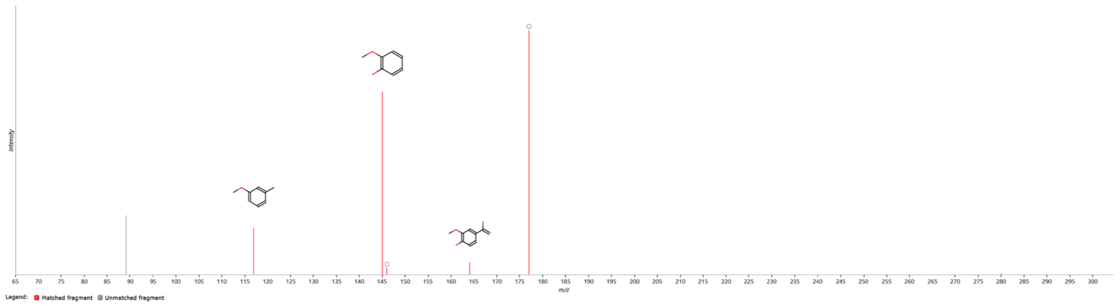

Compound ID	Description	Adducts	Formula	Score	Fragmentat	Mass error (ppm)	Isotope similarity
3ionyvJzLjlkMYC	knap:c00004058_/_racemoflavone	M+Na	C21H18O6	51.6	73.8	-1.55	86.29402966

Figure S16. Database search of racemoflavone at 2.65-min with m/z 389.0990.

10. kaempferol-3-o-rutinoside

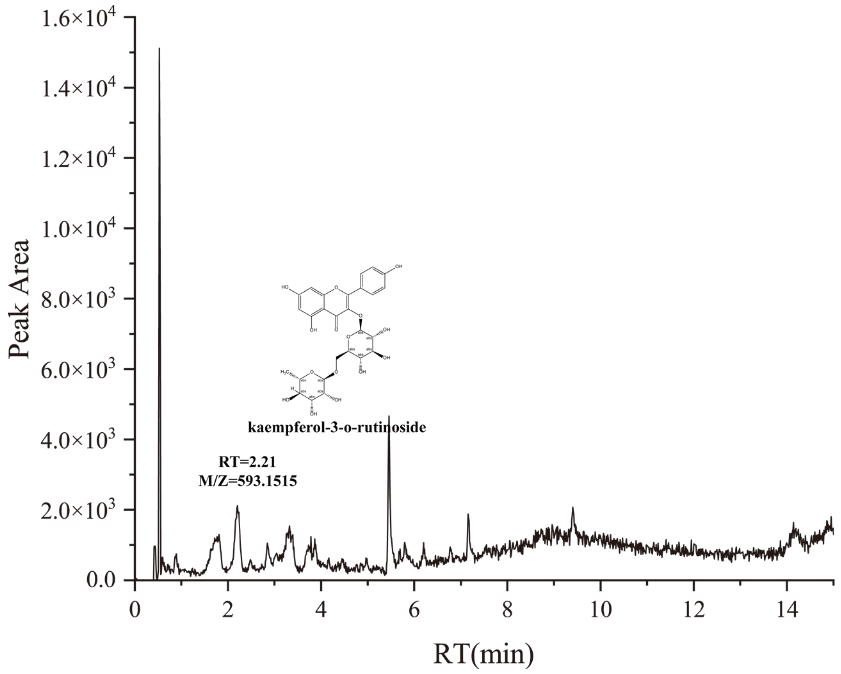

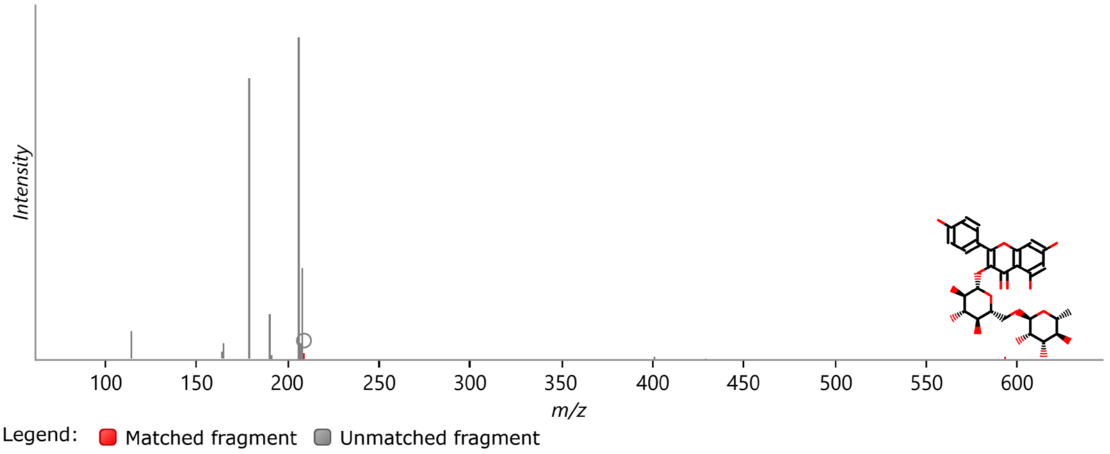

Compound ID	Description	Adducts	Formula	Score	Fragmentat	Mass error (ppm)
QQE4g-dQSOBH1PA	npass:npc173582_/_kaempferol-3-o-rutinoside	M-H	C27H30O15	38.9	2.49	0.47
TxcAc1PVzYTHAa7	kegg:c21833_/_kaempferol-3-o-rutinoside	M-H	C27H30O15	38.9	2.49	0.47
whoZt5gWwxGKHLn	knap:c00010154_/_daidzein 7-o-glucoside-4'-o-	M+FA-H	C26H28O13	38.7	0	0.51
He2J5hdX0AJh00I	foodb:fdb002630_/_vicenin 2	M-H	C27H30O15	38.6	0.998	0.47
o5XU1uCYK8HpeEF	cmaup:npc112701_/_vicenin-2	M-H	C27H30O15	38.6	0.998	0.47

Figure S17. Database search of kaempferol-3-o-rutinoside at 2.21-min with m/z 593.1515.
