# Supplementary material for: Research on the Hormonomics of Three Lilium Species and Their Flavonoid Diversification and Specificity
Source: Antioxidants (Basel). 2025 Jul 14;14(7):862. doi: 10.3390/antiox14070862 (PMC12291843; doi:10.3390/antiox14070862)

Hormone internal standard curve and Mass Spectra information

Result Table	MWXS-24-3064-a_9_WH6500-17_A20-3_V6.0_WSS_20240730	Algorithm Used	MQ4
Acquisition Method	ACC-PHs_V6.0_WH6500-17_CMY_20240521.dam	Instrument Name	Triple Quad 6500+ Low Mass
Project	N/A	Analytes QTY	109:0

Compound name: ABA-GE+Na (449.3 / 287.2)

Sample Name	Sample Type	Area (cps)	Is Area (cps)	RT (min)	S/N	Target Conc	Calculated Conc.()
STD_0.01ppb	Standard	N/A	1.776e6	N/A	N/A	0.0100	N/A
STD_0.05ppb	Standard	N/A	1.714e6	N/A	N/A	0.0500	N/A
STD_0.1ppb	Standard	N/A	1.713e6	N/A	N/A	0.1000	N/A
STD_0.5ppb	Standard	3.22e4	1.581e6	4.50	9.3	0.5000	5.359802e-1
STD_1ppb	Standard	4.44e4	1.620e6	4.51	10.9	1.0000	8.317566e-1
STD_5ppb	Standard	2.40e5	1.820e6	4.52	29.4	5.0000	5.249680e0
STD_10ppb	Standard	4.74e5	1.674e6	4.50	38.1	10.0000	1.162544e1
STD_50ppb	Standard	1.90e6	1.688e6	4.50	53.1	50.0000	4.713698e1
STD_100ppb	Standard	3.22e6	1.498e6	4.51	47.6	100.0000	9.041646e1
STD_200ppb	Standard	7.09e6	1.443e6	4.50	43.6	200.0000	2.073798e2
STD_500ppb	Standard	N/A	1.136e6	N/A	N/A	500.0000	N/A
V2.0_MW_RQC1_20240724	Quality Control	N/A	3.207e5	N/A	N/A	0.0000	N/A
Blank	Unknown	N/A	4.110e2	N/A	N/A	N/A	N/A
V3.0_MWMS_20240725_1	Unknown	1.34e6	1.002e6	4.50	38.6	N/A	5.601229e1
MWXS243064a_R1	Quality Control	2.95e5	4.433e5	4.48	5.7	0.0000	2.778105e1
MWXS243064a_R2	Quality Control	2.59e5	4.254e5	4.50	5.2	0.0000	2.544029e1
MWXS243064a_R3	Quality Control	2.53e5	4.264e5	4.48	5.3	0.0000	2.472558e1
T24186682b_a	Unknown	4.53e5	3.571e5	4.49	8.4	N/A	5.332017e1
T24186682b_b	Unknown	5.02e5	3.443e5	4.50	5.1	N/A	6.131845e1
T24186682b_c	Unknown	4.68e5	3.247e5	4.50	7.1	N/A	6.060402e1
T24186683b_a	Unknown	N/A	5.544e5	N/A	N/A	N/A	N/A
T24186683b_b	Unknown	N/A	5.030e5	N/A	N/A	N/A	N/A
T24186683b_c	Unknown	N/A	5.433e5	N/A	N/A	N/A	N/A
T24186684b_a	Unknown	N/A	4.203e5	N/A	N/A	N/A	N/A
T24186684b_b	Unknown	N/A	4.614e5	N/A	N/A	N/A	N/A
T24186684b_c	Unknown	N/A	4.338e5	N/A	N/A	N/A	N/A

Note: T24186682b represents BF7, T24186683b represents ANK, and T24186684b represents DWH, where _a/b/c denote three biological replicates, respectively. All the following tables follow the same pattern.

Compound name: ABA-GE+Na

Regression Equation: y = 0.02367 x + 0.00770 (r = 0.99215) (weighting: 1 / x^2)

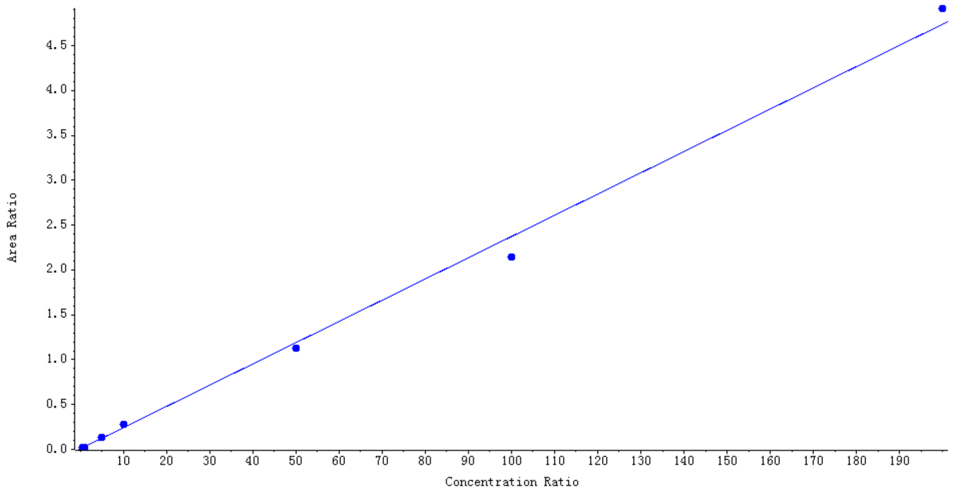

Note：Plot the standard curves for each substance with the external/internal concentration ratio (Concentration Ratio) on the x-axis and the external/internal peak area ratio (Area Ratio) on the y-axis. All the following curves follow the same pattern.

Peak Review

Blank
ABA-GE+Na AREA:N/A S/N:N/A

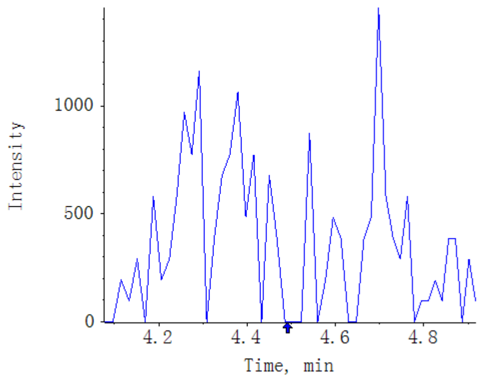

V3.0_MWMS_20240725_1
ABA-GE+Na AREA:1.34e6
S/N:38.6

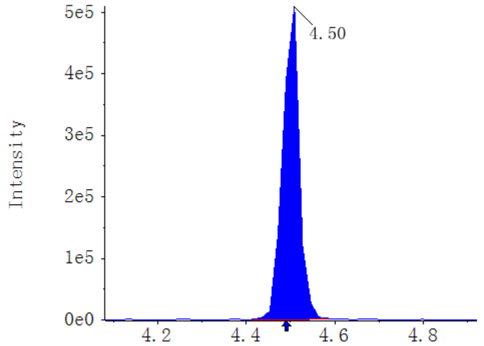

T24186682b_a
ABA-GE+Na AREA:4.53e5
S/N:8.4

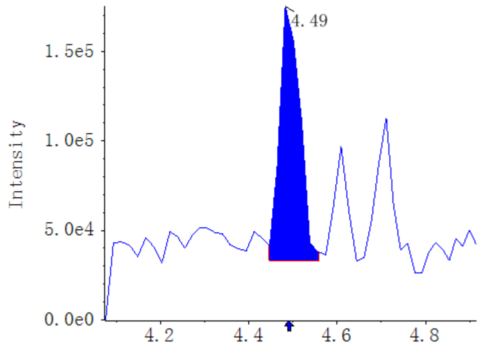

T24186682b_b
ABA-GE+Na AREA:5.02e5
S/N:5.1

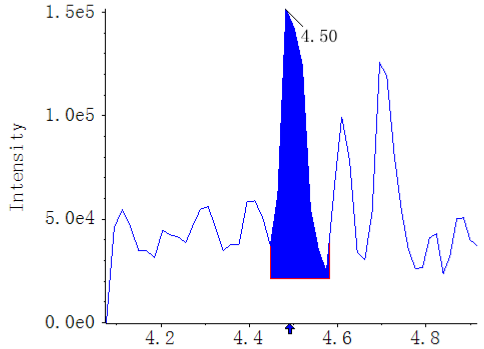

T24186682b_c
ABA-GE+Na AREA:4.68e5
S/N:7.1

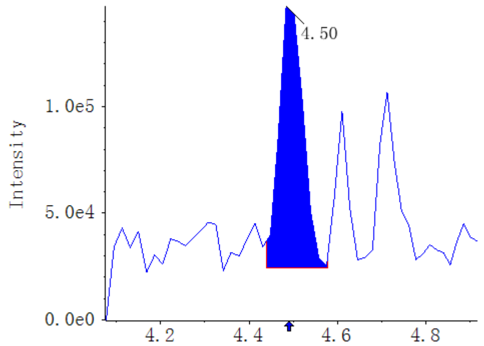

T24186683b_a
ABA-GE+Na AREA:N/A S/N:N/A

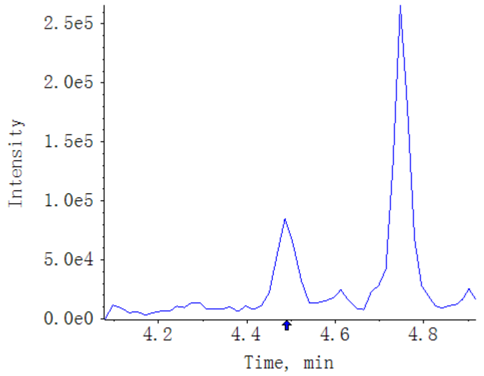

T24186683b_b
ABA-GE+Na AREA:N/A S/N:N/A

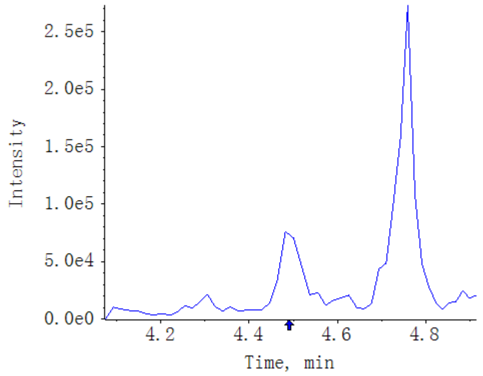

T24186683b_c
ABA-GE+Na AREA:N/A S/N:N/A

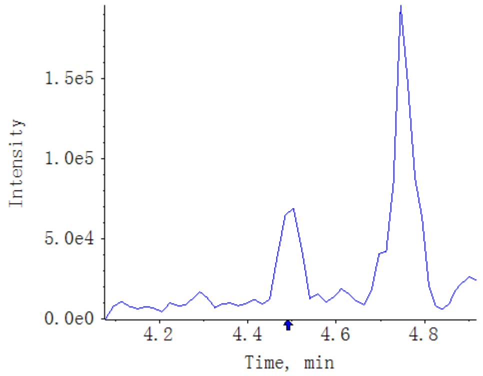

T24186684b_a
ABA-GE+Na AREA:N/A S/N:N/A

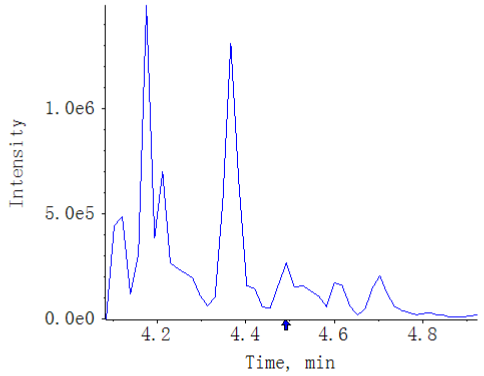

T24186684b_b
ABA-GE+Na AREA:N/A S/N:N/A

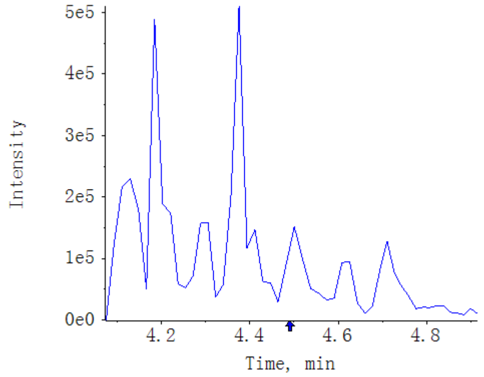

T24186684b_c
ABA-GE+Na AREA:N/A S/N:N/A

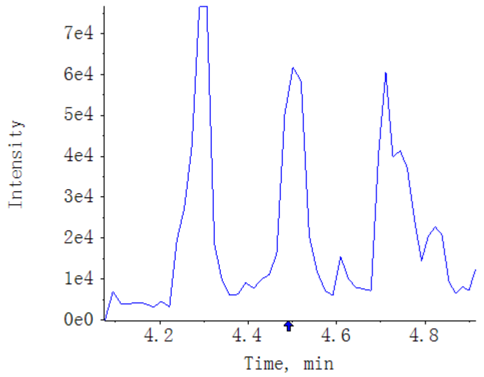

Result Table	MWXS-24-3064-a_9_WH6500-17_A20-3_V6.0_WSS_20240730	Algorithm Used	MQ4
Acquisition Method	ACC-PHs_V6.0_WH6500-17_CMY_20240521.dam	Instrument Name	Triple Quad 6500+ Low Mass
Project	N/A	Analytes QTY	109:1

Compound name: ABA-ald (249.2 / 147.0)

Sample Name	Sample Type	Area (cps)	Is Area (cps)	RT (min)	S/N	Target Conc	Calculated Conc.()
STD_0.01ppb	Standard	N/A	1.776e6	N/A	N/A	0.0100	N/A
STD_0.05ppb	Standard	N/A	1.714e6	N/A	N/A	0.0500	N/A
STD_0.1ppb	Standard	N/A	1.713e6	N/A	N/A	0.1000	N/A
STD_0.5ppb	Standard	8.46e3	1.581e6	5.53	7.6	0.5000	4.707877e-1
STD_1ppb	Standard	1.70e4	1.620e6	5.52	14.0	1.0000	1.120980e0
STD_5ppb	Standard	7.68e4	1.820e6	5.53	18.7	5.0000	5.137007e0
STD_10ppb	Standard	1.23e5	1.674e6	5.52	17.4	10.0000	9.077670e0
STD_50ppb	Standard	6.66e5	1.688e6	5.52	14.4	50.0000	4.976006e1
STD_100ppb	Standard	1.14e6	1.498e6	5.53	13.9	100.0000	9.654112e1
STD_200ppb	Standard	2.26e6	1.443e6	5.53	16.1	200.0000	1.980552e2
STD_500ppb	Standard	4.72e6	1.136e6	5.52	17.5	500.0000	5.256940e2
V2.0_MW_RQC1_20240724	Quality Control	N/A	3.207e5	N/A	N/A	0.0000	N/A
Blank	Unknown	N/A	4.110e2	N/A	N/A	N/A	N/A
V3.0_MWMS_20240725_1	Unknown	7.80e4	1.002e6	5.50	14.2	N/A	9.647313e0
MWXS243064a_R1	Quality Control	N/A	4.433e5	N/A	N/A	0.0000	N/A
MWXS243064a_R2	Quality Control	N/A	4.254e5	N/A	N/A	0.0000	N/A
MWXS243064a_R3	Quality Control	N/A	4.264e5	N/A	N/A	0.0000	N/A
T24186682b_a	Unknown	4.81e4	3.571e5	5.53	5.3	N/A	1.684464e1
T24186682b_b	Unknown	3.70e4	3.443e5	5.53	7.1	N/A	1.339497e1
T24186682b_c	Unknown	4.63e4	3.247e5	5.53	6.3	N/A	1.783293e1
T24186683b_a	Unknown	N/A	5.544e5	N/A	N/A	N/A	N/A
T24186683b_b	Unknown	N/A	5.030e5	N/A	N/A	N/A	N/A
T24186683b_c	Unknown	N/A	5.433e5	N/A	N/A	N/A	N/A
T24186684b_a	Unknown	9.98e4	4.203e5	5.54	7.1	N/A	2.987065e1
T24186684b_b	Unknown	8.91e4	4.614e5	5.55	8.8	N/A	2.424068e1
T24186684b_c	Unknown	1.00e5	4.338e5	5.54	9.0	N/A	2.907423e1

Compound name: ABA-ald
Regression Equation: y = 0.00790 x + 0.00163 (r = 0.99731) (weighting: 1 / x^2)

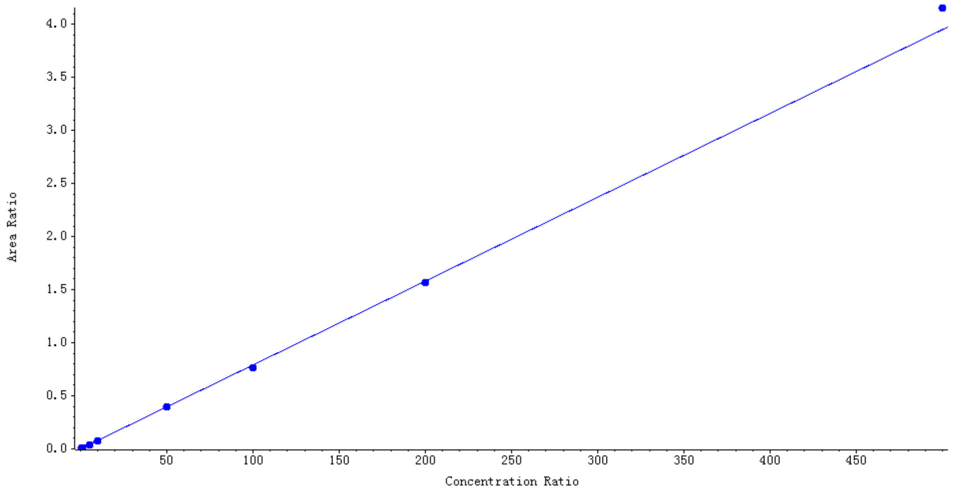

Peak Review

Blank
ABA-ald AREA:N/A S/N:N/A

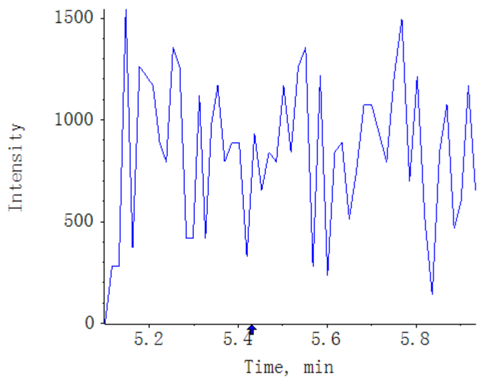

V3.0_MWMS_20240725_1
ABA-ald AREA:7.80e4 S/N:14.2

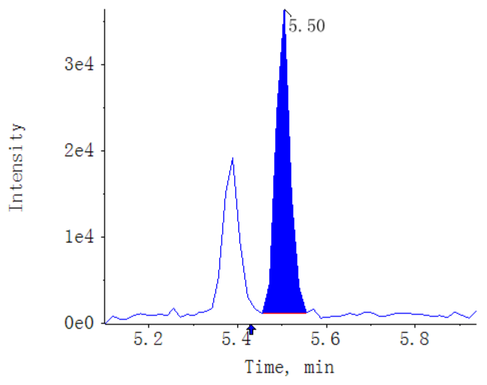

T24186682b_a
ABA-ald AREA:4.81e4 S/N:5.3

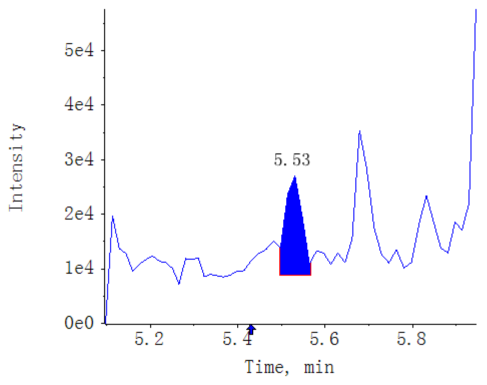

T24186682b_b
ABA-ald AREA:3.70e4 S/N:7.1

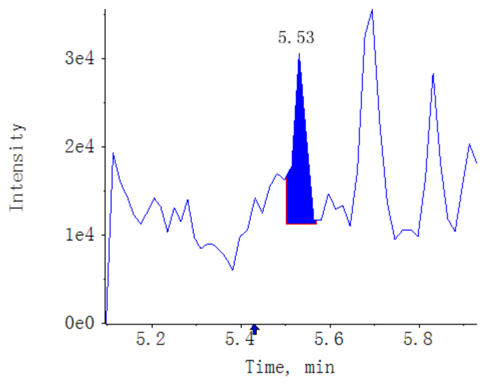

T24186682b_c
ABA-ald AREA:4.63e4 S/N:6.3

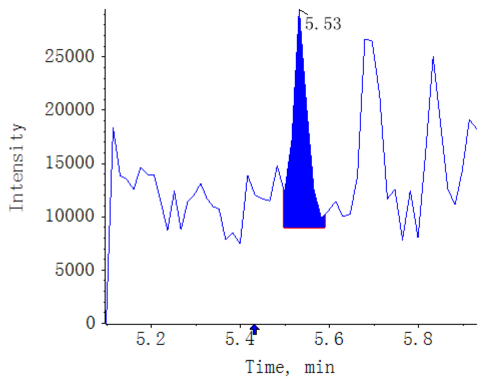

T24186683b_a
ABA-ald AREA:N/A S/N:N/A

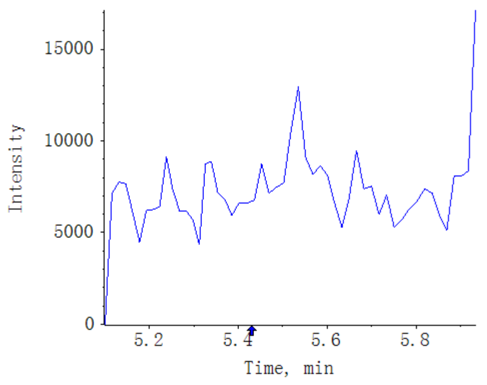

T24186683b_b
ABA-ald AREA:N/A S/N:N/A

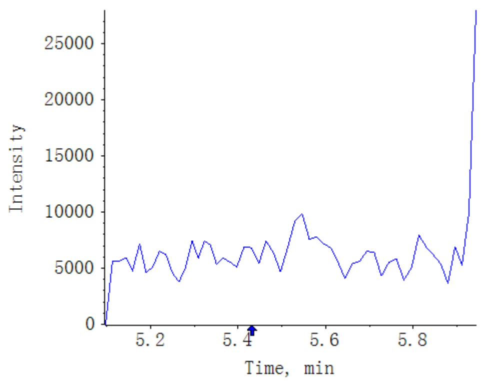

T24186683b_c
ABA-ald AREA:N/A S/N:N/A

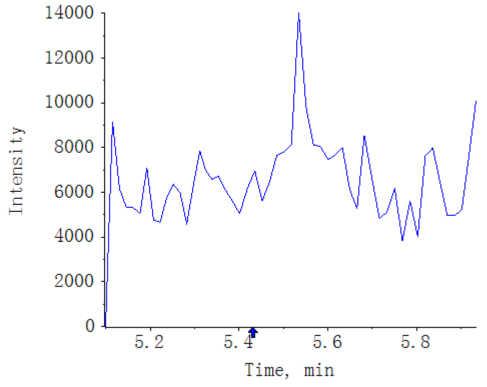

T24186684b_a
ABA-ald AREA:9.98e4 S/N:7.1

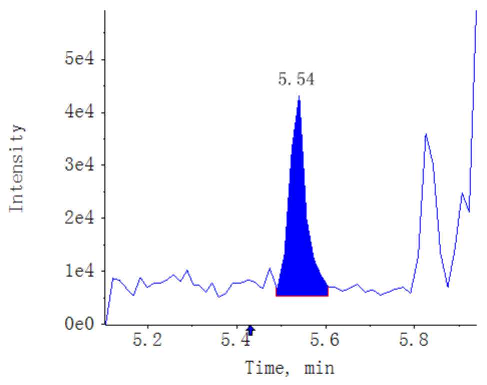

T24186684b_b
ABA-ald AREA:8.91e4 S/N:8.8

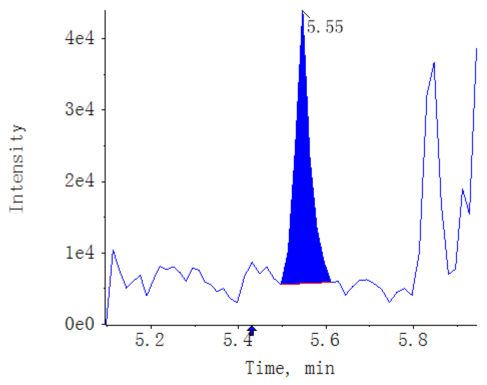

T24186684b_c
ABA-ald AREA:1.00e5 S/N:9.0

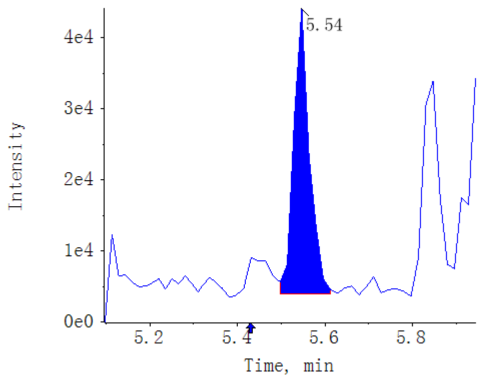

Result Table	MWXS-24-3064-a_9_WH6500-17_A20-3_V6.0_WSS_20240730	Algorithm Used	MQ4
Acquisition Method	ACC-PHs_V6.0_WH6500-17_CMY_20240521.dam	Instrument Name	Triple Quad 6500+ Low Mass
Project	N/A	Analytes QTY	109:2

Compound name: IAN (157.0 / 130.1)

Sample Name	Sample Type	Area (cps)	Is Area (cps)	RT (min)	S/N	Target Conc	Calculated Conc.()
STD_0.01ppb	Standard	4.75e3	2.044e6	5.79	8.5	0.0100	1.016603e-2
STD_0.05ppb	Standard	6.87e3	2.178e6	5.80	15.3	0.0500	4.192203e-2
STD_0.1ppb	Standard	1.10e4	2.145e6	5.81	18.8	0.1000	1.174687e-1
STD_0.5ppb	Standard	3.06e4	2.053e6	5.79	27.2	0.5000	4.917997e-1
STD_1ppb	Standard	4.94e4	2.027e6	5.80	55.0	1.0000	8.542379e-1
STD_5ppb	Standard	2.55e5	1.854e6	5.81	53.3	5.0000	5.190098e0
STD_10ppb	Standard	4.39e5	1.793e6	5.80	57.3	10.0000	9.292366e0
STD_50ppb	Standard	2.39e6	1.706e6	5.80	57.3	50.0000	5.361592e1
STD_100ppb	Standard	4.11e6	1.502e6	5.81	59.6	100.0000	1.045148e2
STD_200ppb	Standard	8.05e6	1.498e6	5.80	51.8	200.0000	2.056165e2
STD_500ppb	Standard	1.55e7	1.165e6	5.80	54.9	500.0000	5.098138e2
V2.0_MW_RQC1_20240724	Quality Control	3.88e3	4.191e5	5.76	6.9	0.0000	2.750298e-1
Blank	Unknown	N/A	3.690e3	N/A	N/A	N/A	N/A
V3.0_MWMS_20240725_1	Unknown	2.95e5	1.529e6	5.77	54.6	N/A	7.309575e0
MWXS243064a_R1	Quality Control	3.42e3	6.675e5	5.74	10.1	0.0000	1.173058e-1
MWXS243064a_R2	Quality Control	3.37e3	6.734e5	5.75	7.1	0.0000	1.126534e-1
MWXS243064a_R3	Quality Control	4.63e3	6.699e5	5.72	10.8	0.0000	1.855197e-1
T24186682b_a	Unknown	N/A	5.433e5	N/A	N/A	N/A	N/A
T24186682b_b	Unknown	N/A	5.395e5	N/A	N/A	N/A	N/A
T24186682b_c	Unknown	N/A	5.840e5	N/A	N/A	N/A	N/A
T24186683b_a	Unknown	3.10e3	7.124e5	5.75	6.1	N/A	8.751829e-2
T24186683b_b	Unknown	4.28e3	7.420e5	5.75	6.3	N/A	1.421378e-1
T24186683b_c	Unknown	3.47e3	7.798e5	5.74	5.6	N/A	9.160454e-2
T24186684b_a	Unknown	N/A	7.287e5	N/A	N/A	N/A	N/A
T24186684b_b	Unknown	N/A	8.161e5	N/A	N/A	N/A	N/A
T24186684b_c	Unknown	N/A	7.655e5	N/A	N/A	N/A	N/A

Compound name: IAN
Regression Equation: y = 0.02613 x + 0.00206 (r = 0.99503) (weighting: 1 / x^2)

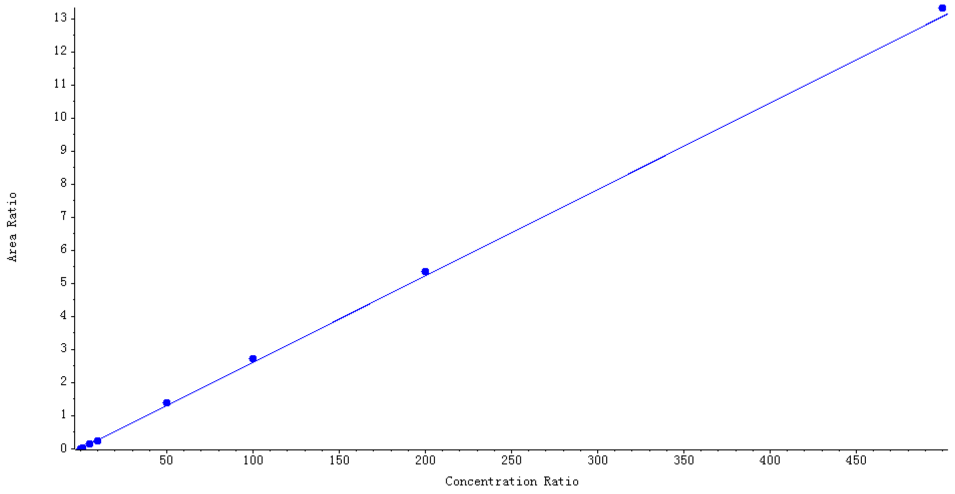

Peak Review

Blank
IAN AREA:N/A S/N:N/A

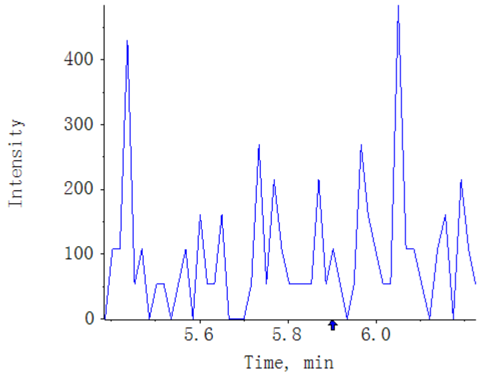

V3.0_MWMS_20240725_1
IAN AREA:2.95e5 S/N:54.6

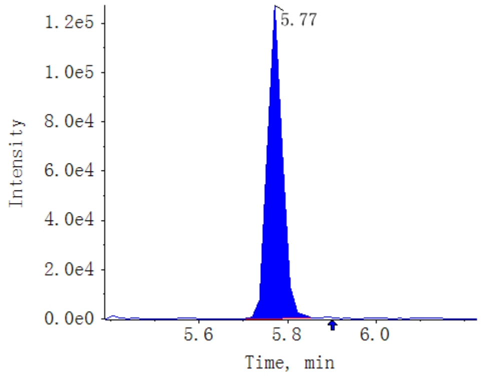

T24186682b_a
IAN AREA:N/A S/N:N/A

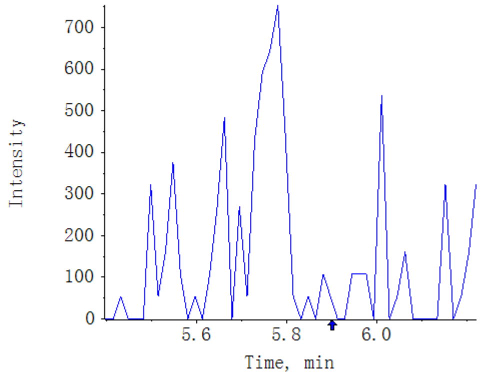

T24186682b_b
IAN AREA:N/A S/N:N/A

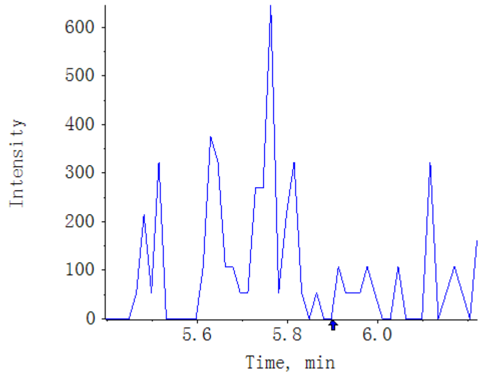

T24186682b_c
IAN AREA:N/A S/N:N/A

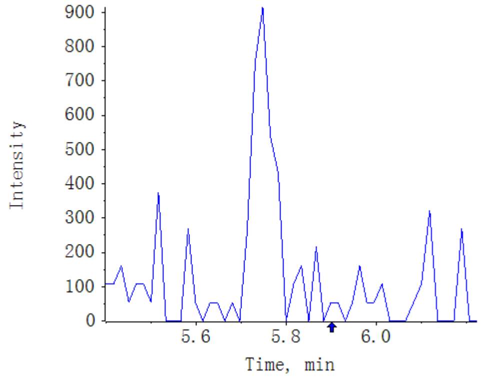

T24186683b_a
IAN AREA:3.10e3 S/N:6.1

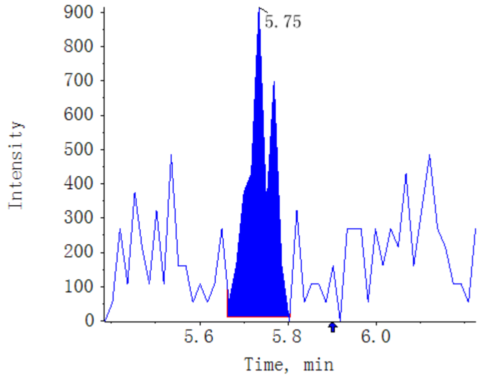

T24186683b_b
IAN AREA:4.28e3 S/N:6.3

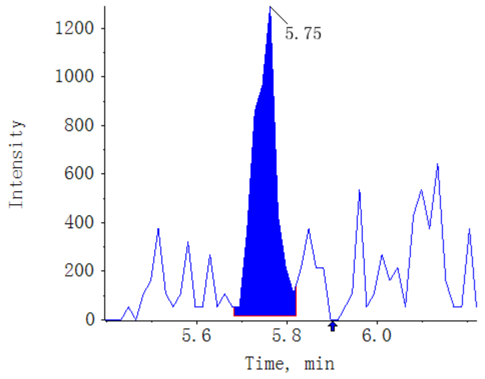

T24186683b_c
IAN AREA:3.47e3 S/N:5.6

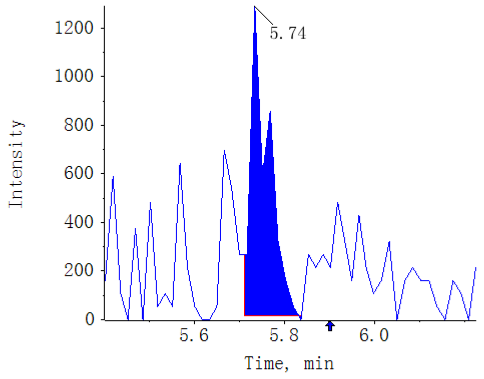

T24186684b_a
IAN AREA:N/A S/N:N/A

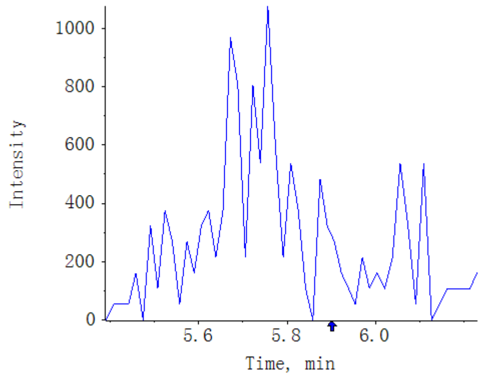

T24186684b_b
IAN AREA:N/A S/N:N/A

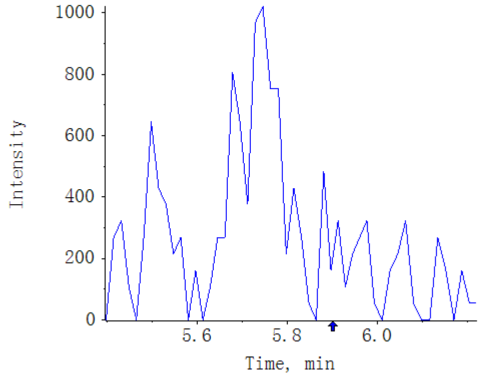

T24186684b_c
IAN AREA:N/A S/N:N/A

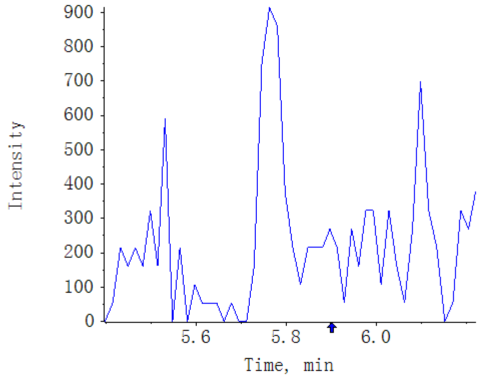

Result Table	MWXS-24-3064-a_9_WH6500-17_A20-3_V6.0_WSS_20240730	Algorithm Used	MQ4
Acquisition Method	ACC-PHs_V6.0_WH6500-17_CMY_20240521.dam	Instrument Name	Triple Quad 6500+ Low Mass
Project	N/A	Analytes QTY	109:3

Compound name: IAA-Leu-Me (302.9 / 130.1)

Sample Name	Sample Type	Area (cps)	Is Area (cps)	RT (min)	S/N	Target Conc	Calculated Conc.()
STD_0.01ppb	Standard	1.54e4	4.457e5	6.46	29.7	0.0100	9.642436e-3
STD_0.05ppb	Standard	7.56e4	4.759e5	6.46	47.9	0.0500	5.590510e-2
STD_0.1ppb	Standard	1.40e5	4.571e5	6.47	59.7	0.1000	1.107993e-1
STD_0.5ppb	Standard	6.78e5	4.822e5	6.46	72.3	0.5000	5.191680e-1
STD_1ppb	Standard	1.22e6	4.362e5	6.46	67.8	1.0000	1.039719e0
STD_5ppb	Standard	6.72e6	4.553e5	6.47	69.8	5.0000	5.485917e0
STD_10ppb	Standard	1.16e7	4.191e5	6.46	61.8	10.0000	1.029402e1
STD_50ppb	Standard	4.73e7	3.976e5	6.46	53.9	50.0000	4.422499e1
STD_100ppb	Standard	7.19e7	3.709e5	6.47	68.5	100.0000	7.205209e1
STD_200ppb	Standard	N/A	3.446e5	N/A	N/A	200.0000	N/A
STD_500ppb	Standard	N/A	2.675e5	N/A	N/A	500.0000	N/A
V2.0_MW_RQC1_20240724	Quality Control	N/A	1.104e5	N/A	N/A	0.0000	N/A
Blank	Unknown	N/A	3.683e3	N/A	N/A	N/A	N/A
V3.0_MWMS_20240725_1	Unknown	7.71e6	5.371e5	6.44	67.3	N/A	5.331558e0
MWXS243064a_R1	Quality Control	N/A	2.172e5	N/A	N/A	0.0000	N/A
MWXS243064a_R2	Quality Control	N/A	2.207e5	N/A	N/A	0.0000	N/A
MWXS243064a_R3	Quality Control	N/A	2.224e5	N/A	N/A	0.0000	N/A
T24186682b_a	Unknown	1.09e4	2.103e5	6.46	7.3	N/A	1.608293e-2
T24186682b_b	Unknown	1.02e4	1.670e5	6.45	4.3	N/A	1.947454e-2
T24186682b_c	Unknown	9.54e3	1.830e5	6.45	5.5	N/A	1.619138e-2
T24186683b_a	Unknown	N/A	2.452e5	N/A	N/A	N/A	N/A
T24186683b_b	Unknown	N/A	2.437e5	N/A	N/A	N/A	N/A
T24186683b_c	Unknown	N/A	2.504e5	N/A	N/A	N/A	N/A
T24186684b_a	Unknown	N/A	2.130e5	N/A	N/A	N/A	N/A
T24186684b_b	Unknown	N/A	2.404e5	N/A	N/A	N/A	N/A
T24186684b_c	Unknown	N/A	2.153e5	N/A	N/A	N/A	N/A

Compound name: IAA-Leu-Me
Regression Equation: y = 2.68971 x + 0.00858 (r = 0.99111) (weighting: 1 / x^2)

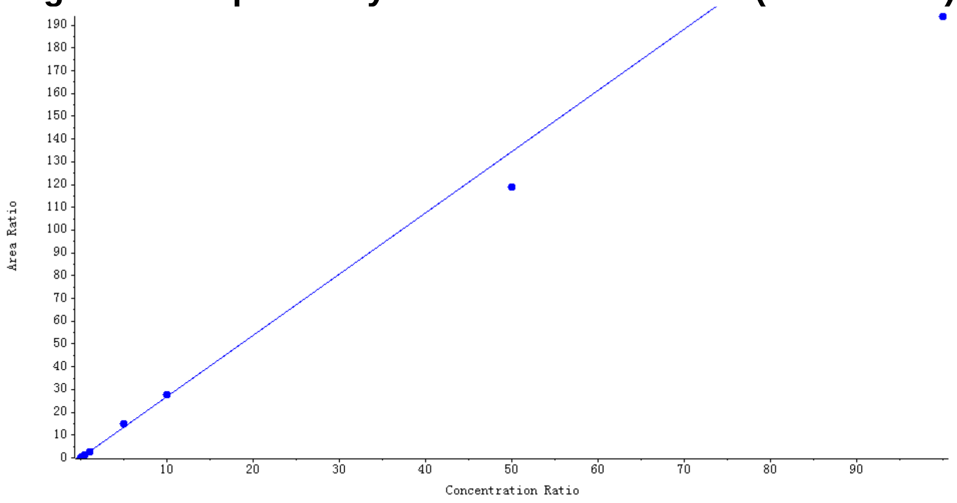

Peak Review

Blank

IAA-Leu-Me AREA:N/A S/N:N/A

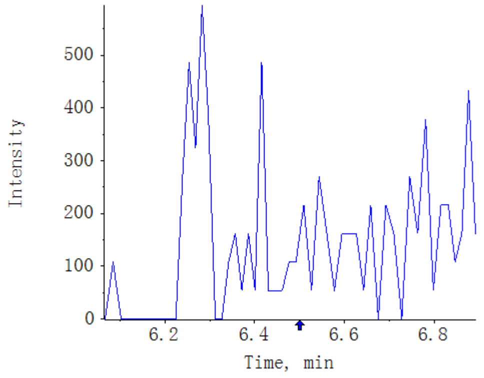

V3.0_MWMS_20240725_1

IAA-Leu-Me AREA:7.71e6 S/N:67.3

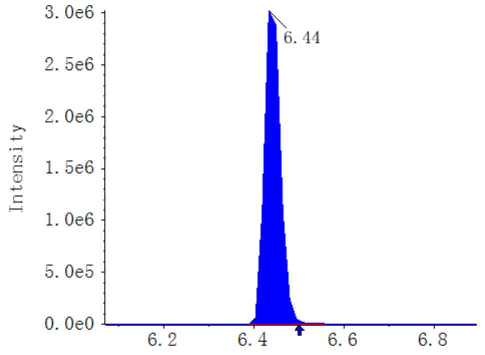

T24186682b_a

IAA-Leu-Me AREA:1.09e4 S/N:7.3

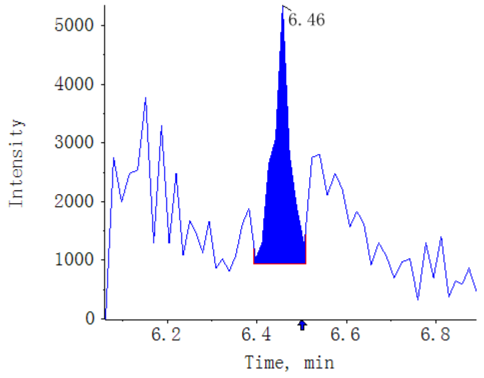

T24186682b_b

IAA-Leu-Me AREA:1.02e4 S/N:4.3

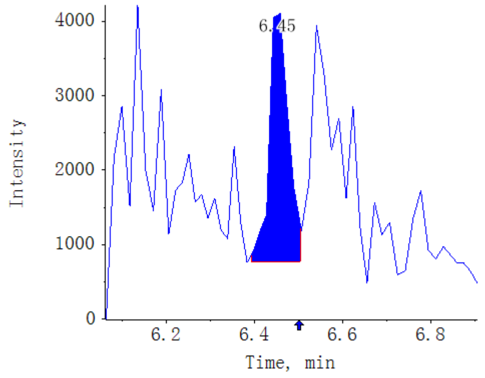

T24186682b_c

IAA-Leu-Me AREA:9.54e3 S/N:5.5

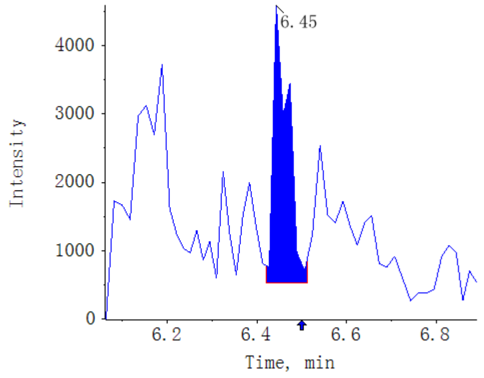

T24186683b_a

IAA-Leu-Me AREA:N/A S/N:N/A

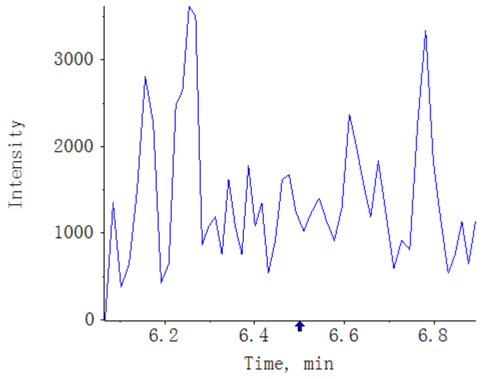

T24186683b_b

IAA-Leu-Me AREA:N/A S/N:N/A

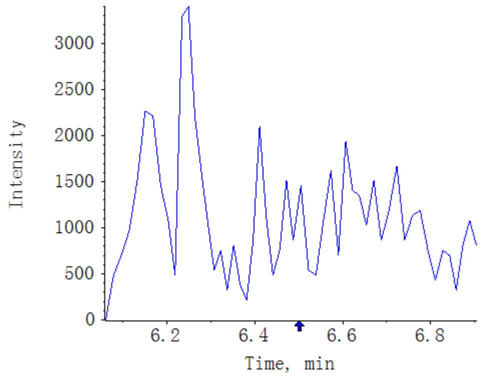

T24186683b_c

IAA-Leu-Me AREA:N/A S/N:N/A

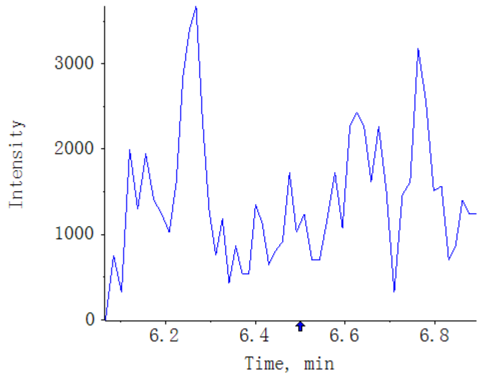

T24186684b_a

IAA-Leu-Me AREA:N/A S/N:N/A

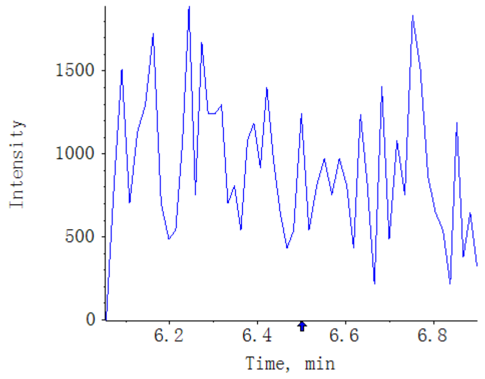

T24186684b_b

IAA-Leu-Me AREA:N/A S/N:N/A

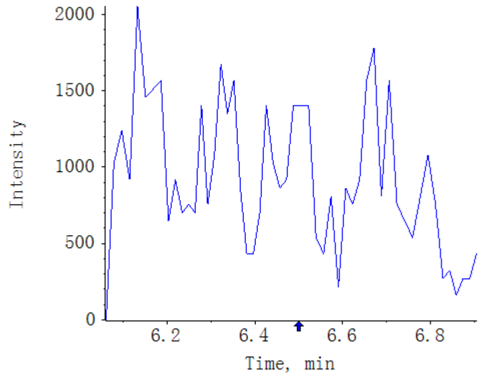

T24186684b_c

IAA-Leu-Me AREA:N/A S/N:N/A

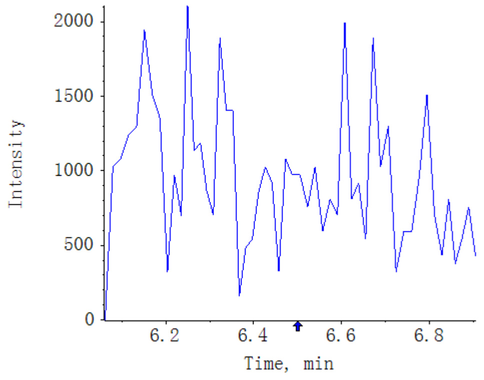

Result Table	MWXS-24-3064-a_9_WH6500-17_A20-3_V6.0_WSS_20240730	Algorithm Used	MQ4
Acquisition Method	ACC-PHs_V6.0_WH6500-17_CMY_20240521.dam	Instrument Name	Triple Quad 6500+ Low Mass
Project	N/A	Analytes QTY	109:4

Compound name: IAA-Val-Me (289.2 / 130.1)

Sample Name	Sample Type	Area (cps)	Is Area (cps)	RT (min)	S/N	Target Conc	Calculated Conc.()
STD_0.01ppb	Standard	N/A	4.457e5	N/A	N/A	0.0100	N/A
STD_0.05ppb	Standard	9.20e4	4.759e5	6.13	22.0	0.0500	4.809038e-2
STD_0.1ppb	Standard	1.56e5	4.571e5	6.14	20.2	0.1000	1.046323e-1
STD_0.5ppb	Standard	7.34e5	4.822e5	6.13	23.1	0.5000	5.528801e-1
STD_1ppb	Standard	1.25e6	4.362e5	6.13	24.6	1.0000	1.060158e0
STD_5ppb	Standard	6.91e6	4.553e5	6.14	19.7	5.0000	5.747067e0
STD_10ppb	Standard	1.15e7	4.191e5	6.13	19.1	10.0000	1.038298e1
STD_50ppb	Standard	4.63e7	3.976e5	6.13	16.6	50.0000	4.420328e1
STD_100ppb	Standard	7.36e7	3.709e5	6.14	16.1	100.0000	7.541734e1
STD_200ppb	Standard	N/A	3.446e5	N/A	N/A	200.0000	N/A
STD_500ppb	Standard	N/A	2.675e5	N/A	N/A	500.0000	N/A
V2.0_MW_RQC1_20240724	Quality Control	N/A	1.104e5	N/A	N/A	0.0000	N/A
Blank	Unknown	N/A	3.683e3	N/A	N/A	N/A	N/A
V3.0_MWMS_20240725_1	Unknown	8.15e6	5.371e5	6.10	23.1	N/A	5.741949e0
MWXS243064a_R1	Quality Control	N/A	2.172e5	N/A	N/A	0.0000	N/A
MWXS243064a_R2	Quality Control	N/A	2.207e5	N/A	N/A	0.0000	N/A
MWXS243064a_R3	Quality Control	N/A	2.224e5	N/A	N/A	0.0000	N/A
T24186682b_a	Unknown	N/A	2.103e5	N/A	N/A	N/A	N/A
T24186682b_b	Unknown	N/A	1.670e5	N/A	N/A	N/A	N/A
T24186682b_c	Unknown	N/A	1.830e5	N/A	N/A	N/A	N/A
T24186683b_a	Unknown	N/A	2.452e5	N/A	N/A	N/A	N/A
T24186683b_b	Unknown	N/A	2.437e5	N/A	N/A	N/A	N/A
T24186683b_c	Unknown	N/A	2.504e5	N/A	N/A	N/A	N/A
T24186684b_a	Unknown	N/A	2.130e5	N/A	N/A	N/A	N/A
T24186684b_b	Unknown	N/A	2.404e5	N/A	N/A	N/A	N/A
T24186684b_c	Unknown	N/A	2.153e5	N/A	N/A	N/A	N/A

Compound name: IAA-Val-Me
Regression Equation: y = 2.63055 x + 0.06686 (r = 0.99014) (weighting: 1 / x^2)

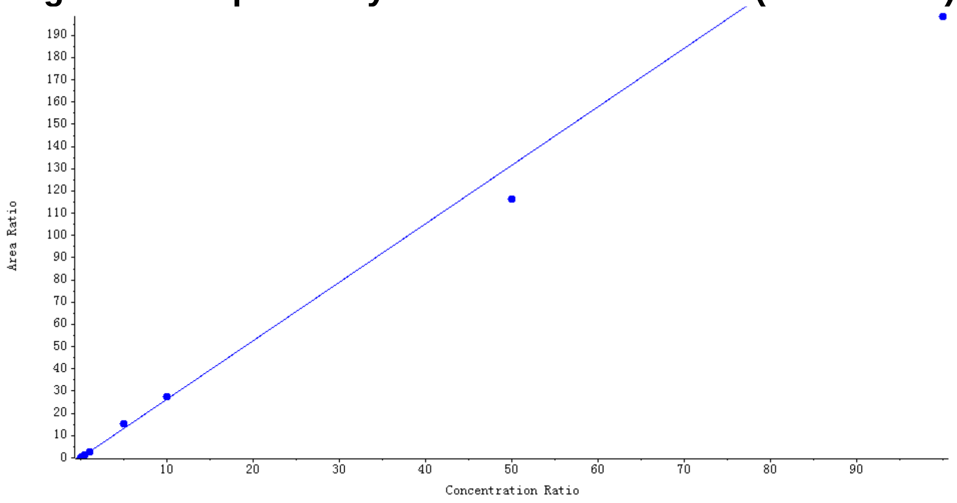

Peak Review

Blank
IAA-Val-Me AREA:N/A S/N:N/A

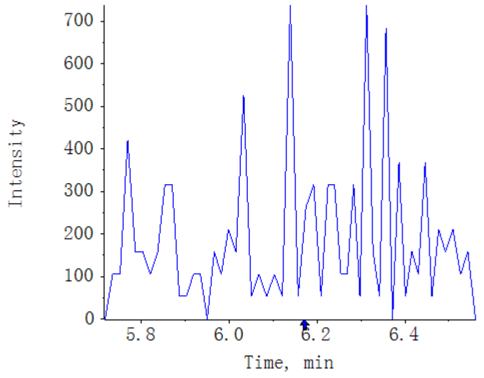

V3.0_MWMS_20240725_1
IAA-Val-Me AREA:8.15e6
S/N:23.1

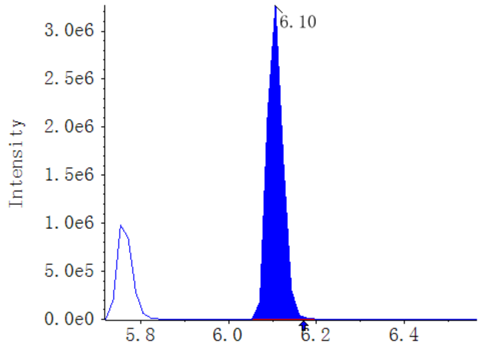

T24186682b_a
IAA-Val-Me AREA:N/A S/N:N/A

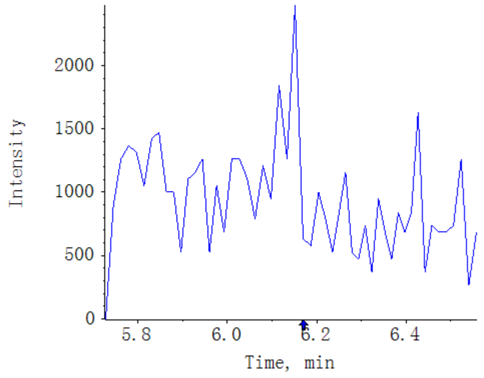

T24186682b_b
IAA-Val-Me AREA:N/A S/N:N/A

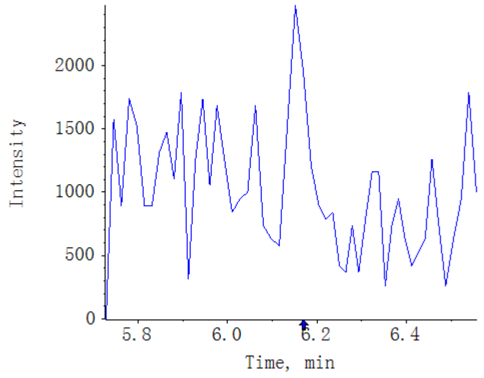

T24186682b_c
IAA-Val-Me AREA:N/A S/N:N/A

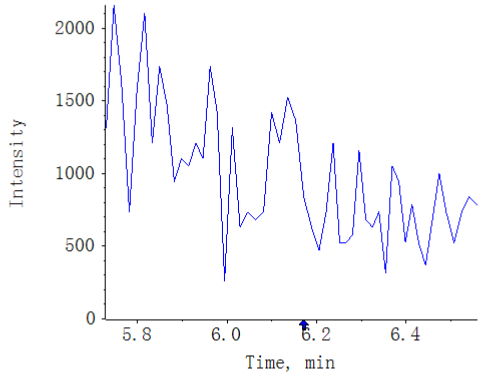

T24186683b_a
IAA-Val-Me AREA:N/A S/N:N/A

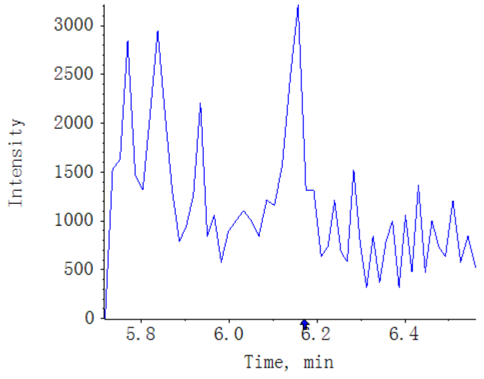

T24186683b_b
IAA-Val-Me AREA:N/A S/N:N/A

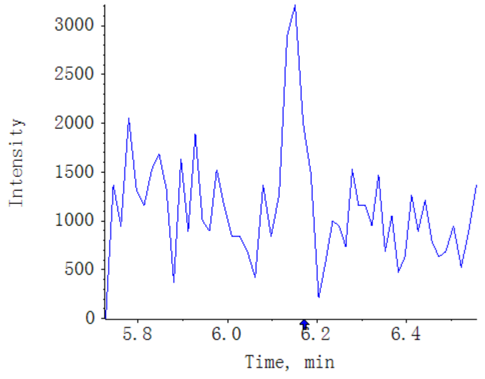

T24186683b_c
IAA-Val-Me AREA:N/A S/N:N/A

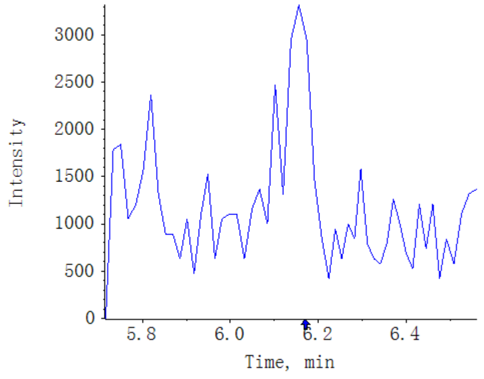

T24186684b_a
IAA-Val-Me AREA:N/A S/N:N/A

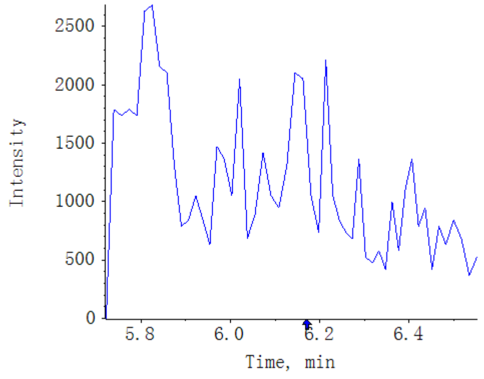

T24186684b_b
IAA-Val-Me AREA:N/A S/N:N/A

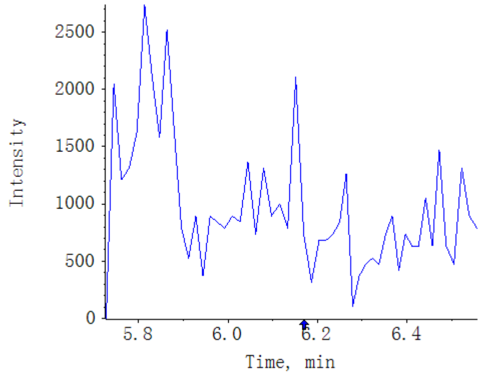

T24186684b_c
IAA-Val-Me AREA:N/A S/N:N/A

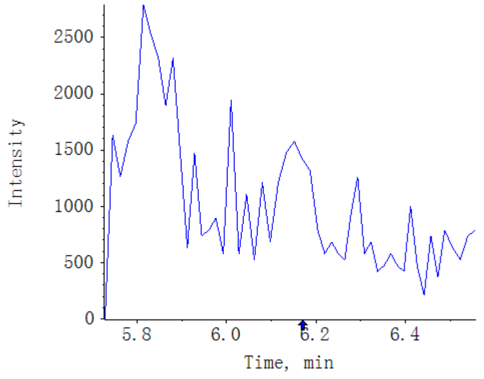

Result Table	MWXS-24-3064-a_9_WH6500-17_A20-3_V6.0_WSS_20240730	Algorithm Used	MQ4
Acquisition Method	ACC-PHs_V6.0_WH6500-17_CMY_20240521.dam	Instrument Name	Triple Quad 6500+ Low Mass
Project	N/A	Analytes QTY	109:5

Compound name: IAA-Gly (233.1 / 130.1)

Sample Name	Sample Type	Area (cps)	Is Area (cps)	RT (min)	S/N	Target Conc	Calculated Conc.()
STD_0.01ppb	Standard	N/A	4.457e5	N/A	N/A	0.0100	N/A
STD_0.05ppb	Standard	N/A	4.759e5	N/A	N/A	0.0500	N/A
STD_0.1ppb	Standard	1.08e4	4.571e5	4.43	10.6	0.1000	1.321063e-1
STD_0.5ppb	Standard	5.93e4	4.822e5	4.42	30.5	0.5000	4.694499e-1
STD_1ppb	Standard	1.12e5	4.362e5	4.42	41.6	1.0000	9.207905e-1
STD_5ppb	Standard	6.34e5	4.553e5	4.43	52.9	5.0000	4.782011e0
STD_10ppb	Standard	1.07e6	4.191e5	4.42	75.9	10.0000	8.698999e0
STD_50ppb	Standard	5.79e6	3.976e5	4.42	58.6	50.0000	4.957018e1
STD_100ppb	Standard	1.07e7	3.709e5	4.43	59.0	100.0000	9.828175e1
STD_200ppb	Standard	2.07e7	3.446e5	4.42	51.1	200.0000	2.037447e2
STD_500ppb	Standard	N/A	2.675e5	N/A	N/A	500.0000	N/A
V2.0_MW_RQC1_20240724	Quality Control	N/A	1.104e5	N/A	N/A	0.0000	N/A
Blank	Unknown	N/A	3.683e3	N/A	N/A	N/A	N/A
V3.0_MWMS_20240725_1	Unknown	8.85e5	5.371e5	4.41	30.6	N/A	5.652534e0
MWXS243064a_R1	Quality Control	N/A	2.172e5	N/A	N/A	0.0000	N/A
MWXS243064a_R2	Quality Control	N/A	2.207e5	N/A	N/A	0.0000	N/A
MWXS243064a_R3	Quality Control	N/A	2.224e5	N/A	N/A	0.0000	N/A
T24186682b_a	Unknown	N/A	2.103e5	N/A	N/A	N/A	N/A
T24186682b_b	Unknown	N/A	1.670e5	N/A	N/A	N/A	N/A
T24186682b_c	Unknown	N/A	1.830e5	N/A	N/A	N/A	N/A
T24186683b_a	Unknown	N/A	2.452e5	N/A	N/A	N/A	N/A
T24186683b_b	Unknown	N/A	2.437e5	N/A	N/A	N/A	N/A
T24186683b_c	Unknown	N/A	2.504e5	N/A	N/A	N/A	N/A
T24186684b_a	Unknown	N/A	2.130e5	N/A	N/A	N/A	N/A
T24186684b_b	Unknown	N/A	2.404e5	N/A	N/A	N/A	N/A
T24186684b_c	Unknown	N/A	2.153e5	N/A	N/A	N/A	N/A

Compound name: IAA-Gly
Regression Equation: y = 0.29425 x + -0.01523 (r = 0.99958) (weighting: 1 / x)

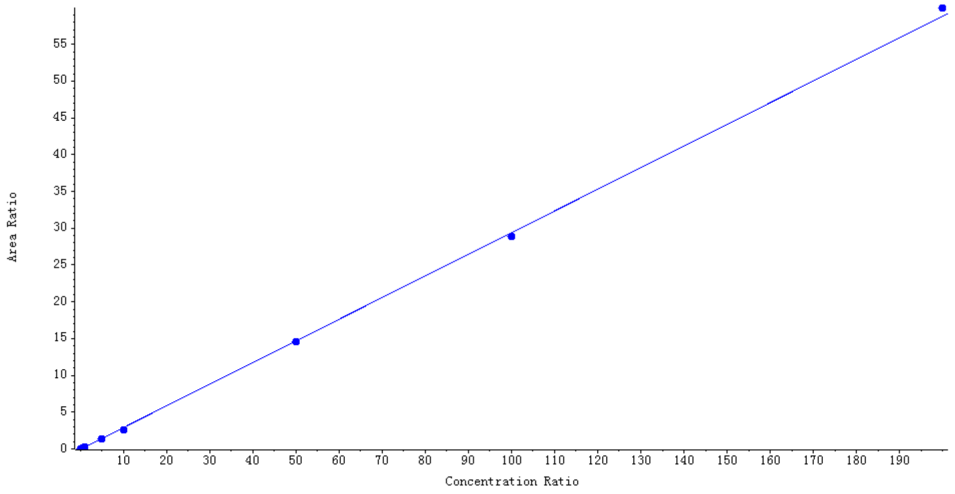

Peak Review

Blank
IAA-Gly AREA:N/A S/N:N/A

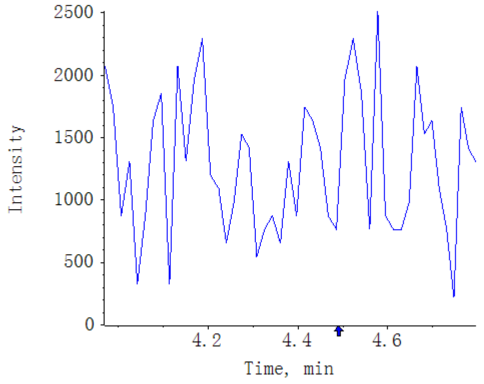

V3.0_MWMS_20240725_1
IAA-Gly AREA:8.85e5 S/N:30.6

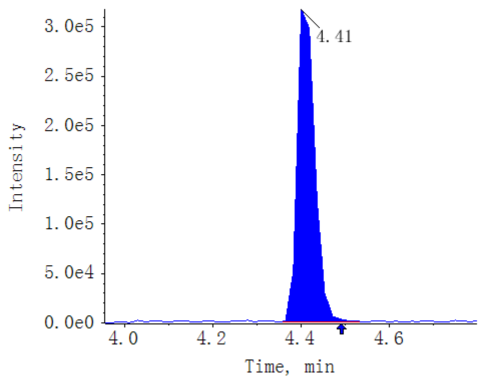

T24186682b_a
IAA-Gly AREA:N/A S/N:N/A

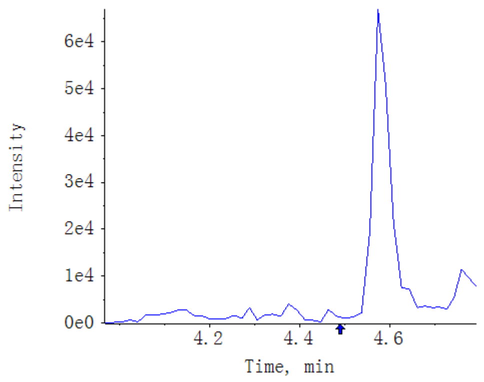

T24186682b_b
IAA-Gly AREA:N/A S/N:N/A

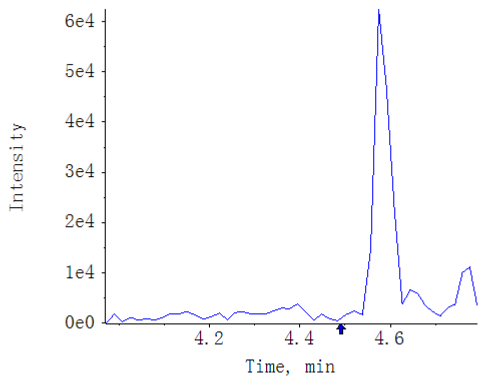

T24186682b_c
IAA-Gly AREA:N/A S/N:N/A

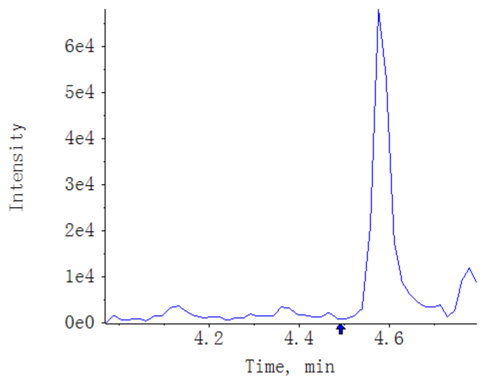

T24186683b_a
IAA-Gly AREA:N/A S/N:N/A

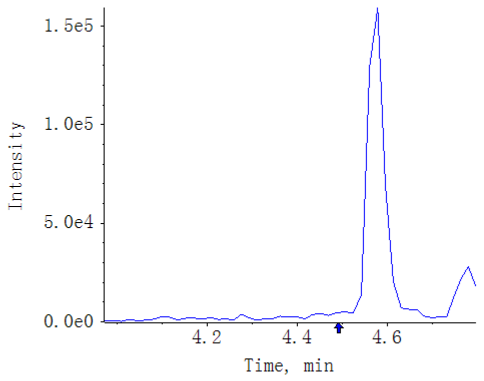

T24186683b_b
IAA-Gly AREA:N/A S/N:N/A

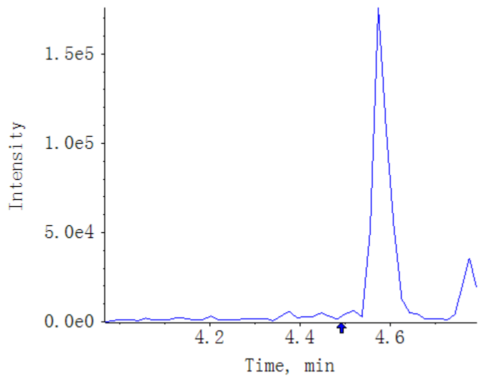

T24186683b_c
IAA-Gly AREA:N/A S/N:N/A

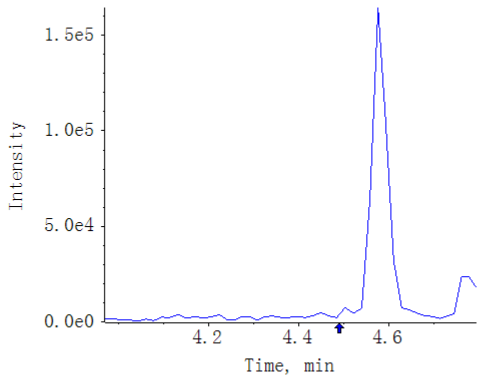

T24186684b_a
IAA-Gly AREA:N/A S/N:N/A

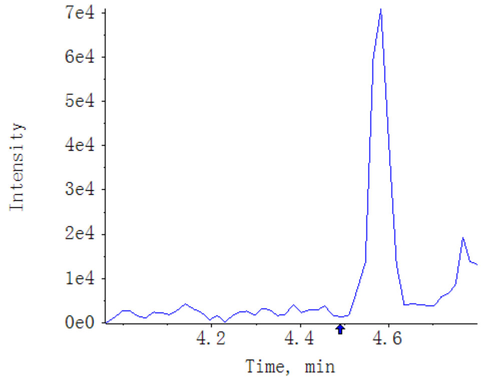

T24186684b_b
IAA-Gly AREA:N/A S/N:N/A

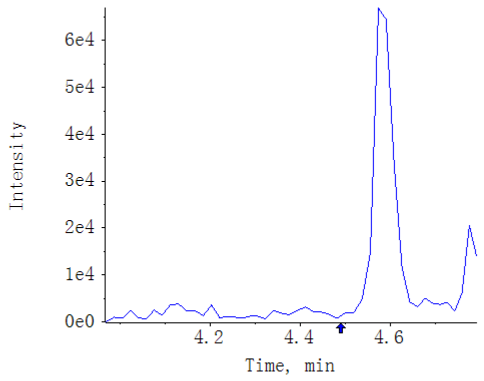

T24186684b_c
IAA-Gly AREA:N/A S/N:N/A

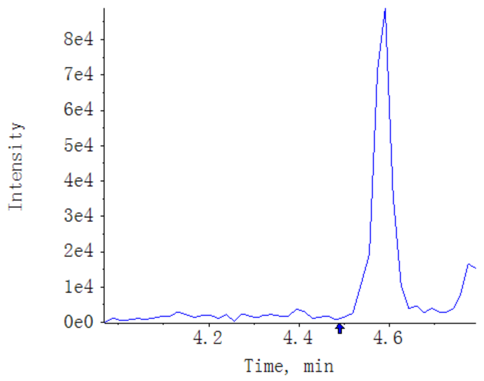

Result Table	MWXS-24-3064-a_9_WH6500-17_A20-3_V6.0_WSS_20240730	Algorithm Used	MQ4
Acquisition Method	ACC-PHs_V6.0_WH6500-17_CMY_20240521.dam	Instrument Name	Triple Quad 6500+ Low Mass
Project	N/A	Analytes QTY	109:6

Compound name: OxIAA (192.1 / 146.1)

Sample Name	Sample Type	Area (cps)	Is Area (cps)	RT (min)	S/N	Target Conc	Calculated Conc.()
STD_0.01ppb	Standard	N/A	1.463e7	N/A	N/A	0.0100	N/A
STD_0.05ppb	Standard	N/A	1.538e7	N/A	N/A	0.0500	N/A
STD_0.1ppb	Standard	1.88e4	1.466e7	4.23	4.0	0.1000	1.096908e-1
STD_0.5ppb	Standard	6.23e4	1.438e7	4.22	14.5	0.5000	4.400234e-1
STD_1ppb	Standard	1.28e5	1.446e7	4.22	22.1	1.0000	9.267067e-1
STD_5ppb	Standard	6.99e5	1.392e7	4.23	46.7	5.0000	5.407499e0
STD_10ppb	Standard	1.23e6	1.335e7	4.22	49.3	10.0000	9.923645e0
STD_50ppb	Standard	6.67e6	1.326e7	4.22	45.9	50.0000	5.435889e1
STD_100ppb	Standard	1.16e7	1.370e7	4.23	57.7	100.0000	9.162569e1
STD_200ppb	Standard	2.50e7	1.327e7	4.22	73.5	200.0000	2.038079e2
STD_500ppb	Standard	N/A	9.829e6	N/A	N/A	500.0000	N/A
V2.0_MW_RQC1_20240724	Quality Control	N/A	2.323e6	N/A	N/A	0.0000	N/A
Blank	Unknown	N/A	3.627e3	N/A	N/A	N/A	N/A
V3.0_MWMS_20240725_1	Unknown	9.51e5	1.365e7	4.22	46.0	N/A	7.516534e0
MWXS243064a_R1	Quality Control	1.63e5	1.937e6	4.20	9.2	0.0000	9.081641e0
MWXS243064a_R2	Quality Control	1.68e5	2.063e6	4.21	7.2	0.0000	8.779664e0
MWXS243064a_R3	Quality Control	1.53e5	2.038e6	4.19	7.3	0.0000	8.103833e0
T24186682b_a	Unknown	1.02e5	1.307e6	4.20	8.3	N/A	8.378658e0
T24186682b_b	Unknown	9.15e4	1.262e6	4.20	7.5	N/A	7.820084e0
T24186682b_c	Unknown	1.12e5	1.267e6	4.20	7.1	N/A	9.549486e0
T24186683b_a	Unknown	1.97e5	3.941e6	4.20	5.2	N/A	5.385896e0
T24186683b_b	Unknown	2.08e5	4.032e6	4.20	6.2	N/A	5.554221e0
T24186683b_c	Unknown	1.95e5	3.923e6	4.21	7.3	N/A	5.363744e0
T24186684b_a	Unknown	2.20e5	2.919e6	4.20	4.1	N/A	8.140041e0
T24186684b_b	Unknown	1.86e5	2.979e6	4.22	7.6	N/A	6.719132e0
T24186684b_c	Unknown	2.05e5	3.050e6	4.21	8.6	N/A	7.262764e0

Compound name: OxIAA
Regression Equation: y = 0.00924 x + 2.66649e-4 (r = 0.99834) (weighting: 1 / x)

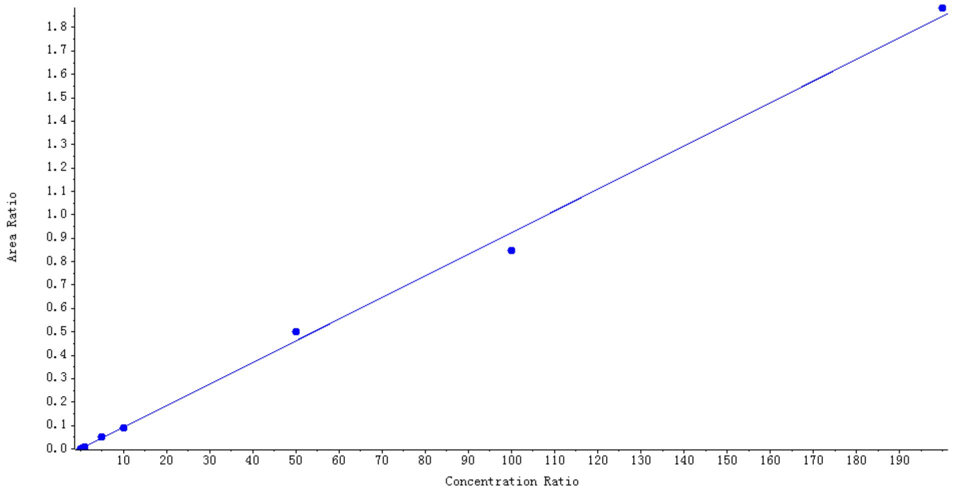

Peak Review

Blank

OxIAA AREA:N/A S/N:N/A

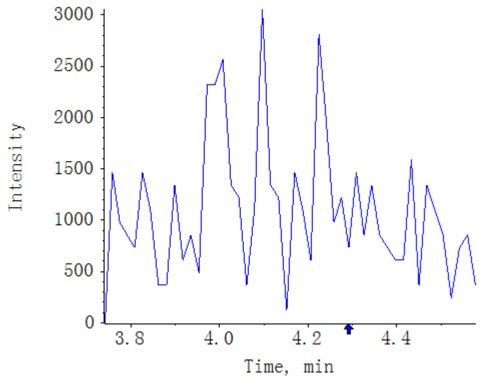

V3.0_MWMS_20240725_1

OxIAA AREA:9.51e5 S/N:46.0

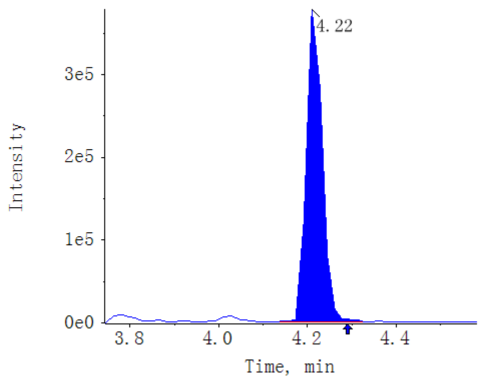

T24186682b_a

OxIAA AREA:1.02e5 S/N:8.3

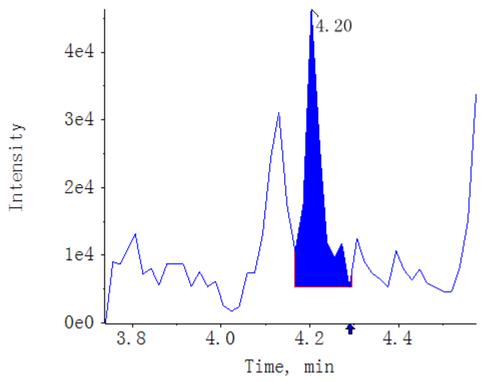

T24186682b_b

OxIAA AREA:9.15e4 S/N:7.5

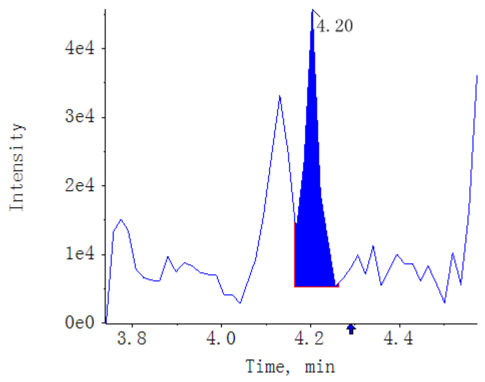

T24186682b_c

OxIAA AREA:1.12e5 S/N:7.1

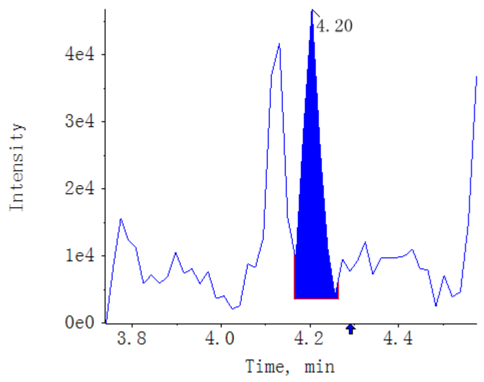

T24186683b_a

OxIAA AREA:1.97e5 S/N:5.2

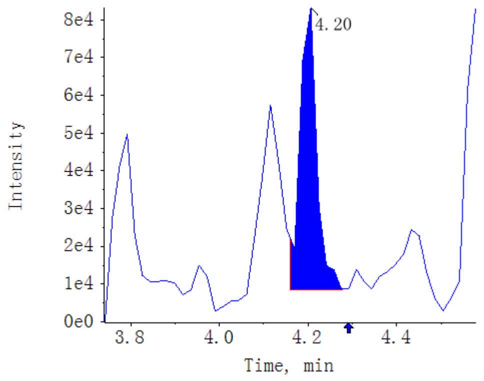

T24186683b_b

OxIAA AREA:2.08e5 S/N:6.2

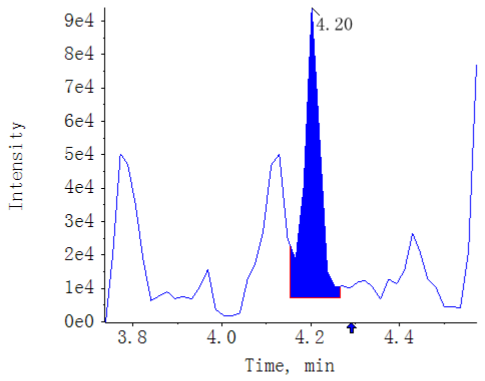

T24186683b_c

OxIAA AREA:1.95e5 S/N:7.3

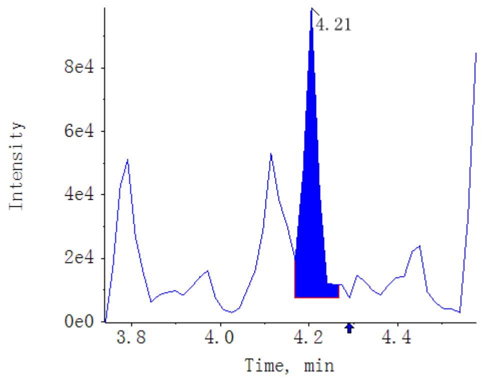

T24186684b_a

OxIAA AREA:2.20e5 S/N:4.1

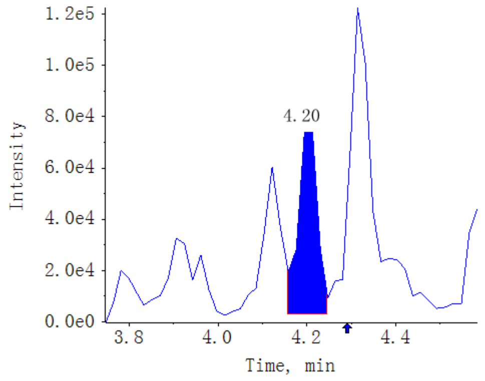

T24186684b_b

OxIAA AREA:1.86e5 S/N:7.6

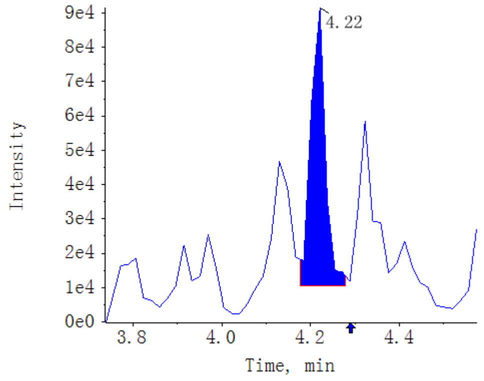

T24186684b_c

OxIAA AREA:2.05e5 S/N:8.6

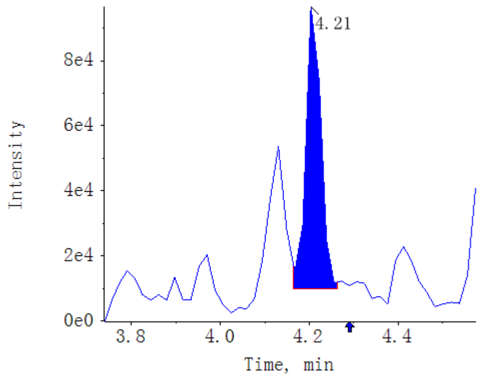

Result Table	MWXS-24-3064-a_9_WH6500-17_A20-3_V6.0_WSS_20240730	Algorithm Used	MQ4
Acquisition Method	ACC-PHs_V6.0_WH6500-17_CMY_20240521.dam	Instrument Name	Triple Quad 6500+ Low Mass
Project	N/A	Analytes QTY	109:7

Compound name: IAA-Leu (289.2 / 130.0)

Sample Name	Sample Type	Area (cps)	Is Area (cps)	RT (min)	S/N	Target Conc	Calculated Conc.()
STD_0.01ppb	Standard	N/A	4.457e5	N/A	N/A	0.0100	N/A
STD_0.05ppb	Standard	1.85e4	4.759e5	5.78	3.3	0.0500	7.379421e-2
STD_0.1ppb	Standard	3.71e4	4.571e5	5.79	2.8	0.1000	1.172803e-1
STD_0.5ppb	Standard	1.83e5	4.822e5	5.78	5.0	0.5000	4.247260e-1
STD_1ppb	Standard	3.24e5	4.362e5	5.79	5.6	1.0000	7.989024e-1
STD_5ppb	Standard	1.96e6	4.553e5	5.80	5.8	5.0000	4.471337e0
STD_10ppb	Standard	3.48e6	4.191e5	5.79	4.3	10.0000	8.592625e0
STD_50ppb	Standard	1.85e7	3.976e5	5.78	6.6	50.0000	4.803522e1
STD_100ppb	Standard	3.40e7	3.709e5	5.79	7.2	100.0000	9.455082e1
STD_200ppb	Standard	6.96e7	3.446e5	5.79	7.9	200.0000	2.080152e2
STD_500ppb	Standard	1.30e8	2.675e5	5.79	6.3	500.0000	5.015701e2
V2.0_MW_RQC1_20240724	Quality Control	N/A	1.104e5	N/A	N/A	0.0000	N/A
Blank	Unknown	N/A	3.683e3	N/A	N/A	N/A	N/A
V3.0_MWMS_20240725_1	Unknown	2.52e6	5.371e5	5.76	6.7	N/A	4.872993e0
MWXS243064a_R1	Quality Control	N/A	2.172e5	N/A	N/A	0.0000	N/A
MWXS243064a_R2	Quality Control	N/A	2.207e5	N/A	N/A	0.0000	N/A
MWXS243064a_R3	Quality Control	N/A	2.224e5	N/A	N/A	0.0000	N/A
T24186682b_a	Unknown	N/A	2.103e5	N/A	N/A	N/A	N/A
T24186682b_b	Unknown	N/A	1.670e5	N/A	N/A	N/A	N/A
T24186682b_c	Unknown	N/A	1.830e5	N/A	N/A	N/A	N/A
T24186683b_a	Unknown	N/A	2.452e5	N/A	N/A	N/A	N/A
T24186683b_b	Unknown	N/A	2.437e5	N/A	N/A	N/A	N/A
T24186683b_c	Unknown	N/A	2.504e5	N/A	N/A	N/A	N/A
T24186684b_a	Unknown	N/A	2.130e5	N/A	N/A	N/A	N/A
T24186684b_b	Unknown	N/A	2.404e5	N/A	N/A	N/A	N/A
T24186684b_c	Unknown	N/A	2.153e5	N/A	N/A	N/A	N/A

Compound name: IAA-Leu
Regression Equation: y = 0.97099 x + -0.03275 (r = 0.99941) (weighting: 1 / x)

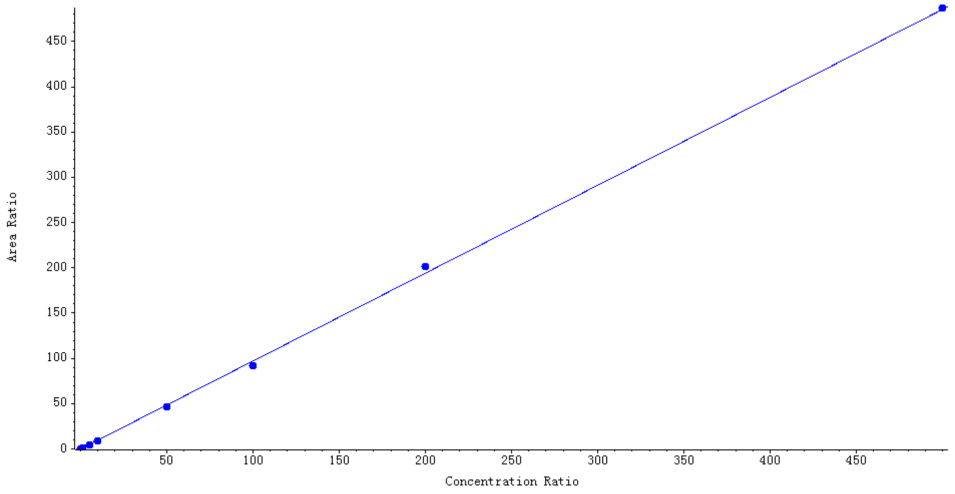

Peak Review

Blank
IAA-Leu AREA:N/A S/N:N/A

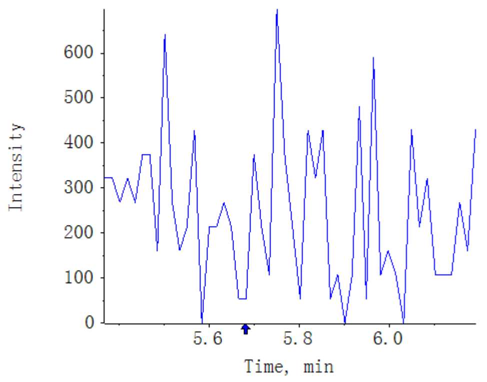

V3.0_MWMS_20240725_1
IAA-Leu AREA:2.52e6 S/N:6.7

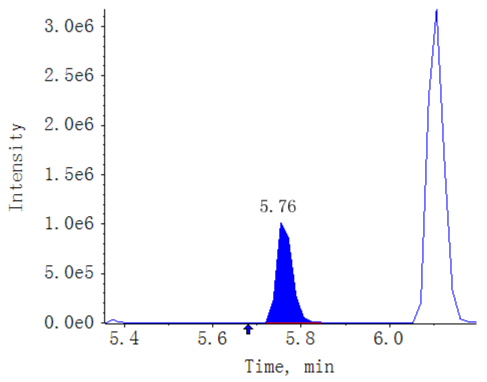

T24186682b_a
IAA-Leu AREA:N/A S/N:N/A

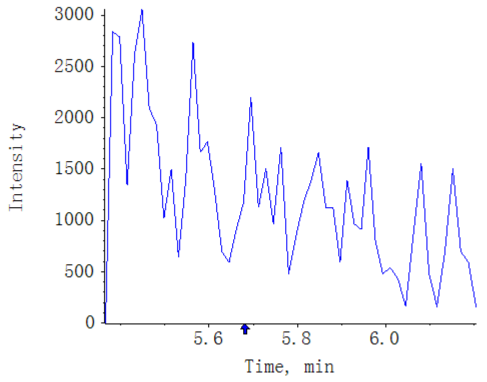

T24186682b_b
IAA-Leu AREA:N/A S/N:N/A

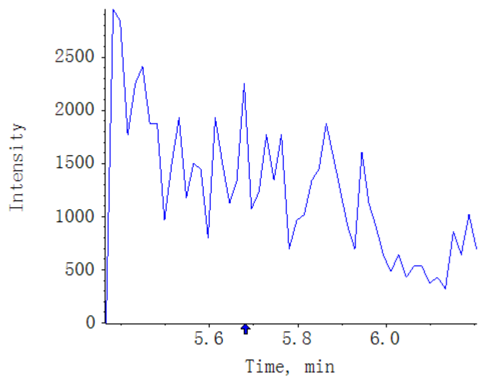

T24186682b_c
IAA-Leu AREA:N/A S/N:N/A

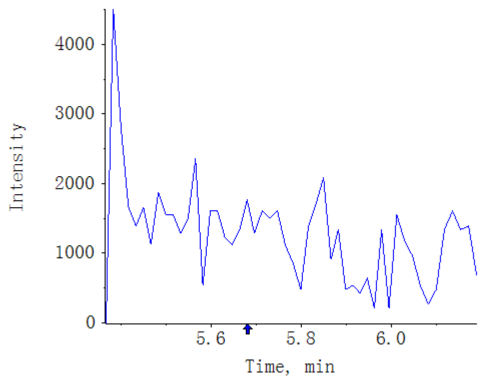

T24186683b_a
IAA-Leu AREA:N/A S/N:N/A

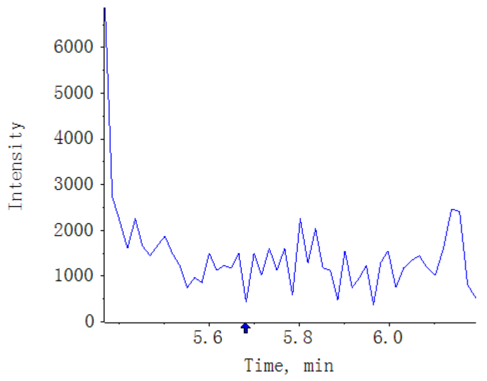

T24186683b_b
IAA-Leu AREA:N/A S/N:N/A

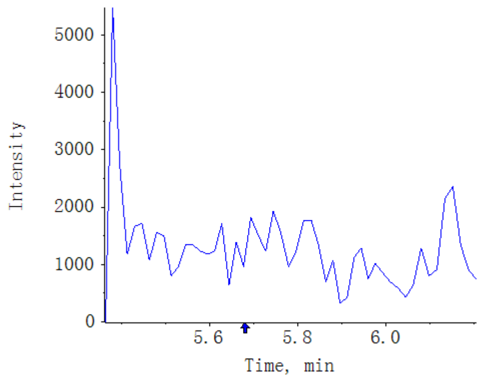

T24186683b_c
IAA-Leu AREA:N/A S/N:N/A

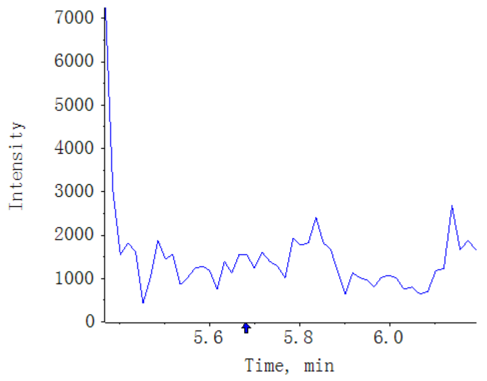

T24186684b_a
IAA-Leu AREA:N/A S/N:N/A

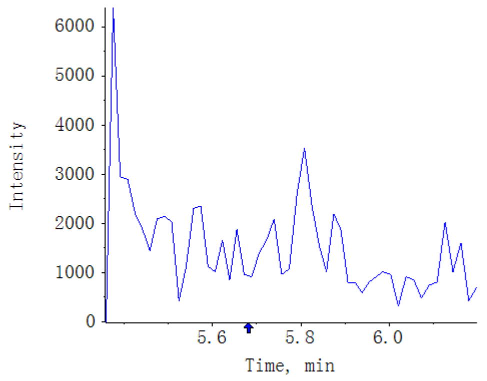

T24186684b_b
IAA-Leu AREA:N/A S/N:N/A

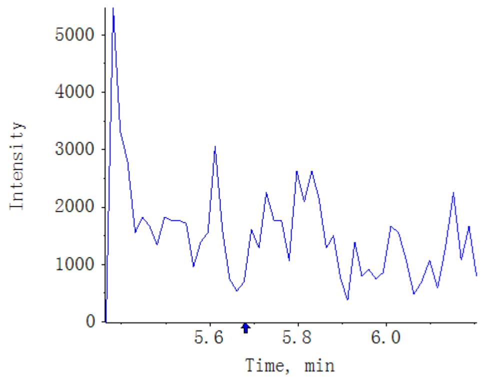

T24186684b_c
IAA-Leu AREA:N/A S/N:N/A

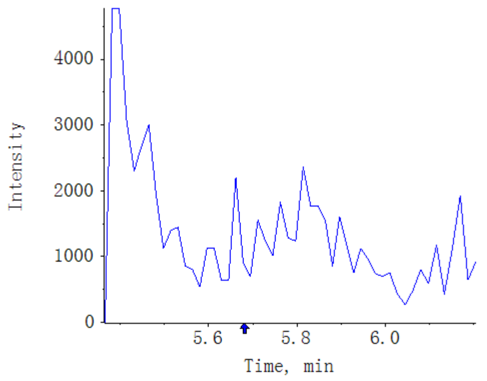

Result Table	MWXS-24-3064-a_9_WH6500-17_A20-3_V6.0_WSS_20240730	Algorithm Used	MQ4
Acquisition Method	ACC-PHs_V6.0_WH6500-17_CMY_20240521.dam	Instrument Name	Triple Quad 6500+ Low Mass
Project	N/A	Analytes QTY	109:8

Compound name: IAA-Val (275.1 / 130.0)

Sample Name	Sample Type	Area (cps)	Is Area (cps)	RT (min)	S/N	Target Conc	Calculated Conc.()
STD_0.01ppb	Standard	2.88e3	4.457e5	5.42	6.6	0.0100	9.873619e-3
STD_0.05ppb	Standard	1.79e4	4.759e5	5.42	33.0	0.0500	5.585789e-2
STD_0.1ppb	Standard	2.90e4	4.571e5	5.43	46.3	0.1000	9.363998e-2
STD_0.5ppb	Standard	1.39e5	4.822e5	5.42	54.0	0.5000	4.244598e-1
STD_1ppb	Standard	2.55e5	4.362e5	5.42	60.8	1.0000	8.607170e-1
STD_5ppb	Standard	1.54e6	4.553e5	5.43	68.3	5.0000	4.970830e0
STD_10ppb	Standard	2.70e6	4.191e5	5.42	54.2	10.0000	9.486004e0
STD_50ppb	Standard	1.53e7	3.976e5	5.41	62.5	50.0000	5.668177e1
STD_100ppb	Standard	2.96e7	3.709e5	5.42	55.8	100.0000	1.173042e2
STD_200ppb	Standard	N/A	3.446e5	N/A	N/A	200.0000	N/A
STD_500ppb	Standard	N/A	2.675e5	N/A	N/A	500.0000	N/A
V2.0_MW_RQC1_20240724	Quality Control	N/A	1.104e5	N/A	N/A	0.0000	N/A
Blank	Unknown	N/A	3.683e3	N/A	N/A	N/A	N/A
V3.0_MWMS_20240725_1	Unknown	2.15e6	5.371e5	5.40	58.3	N/A	5.894333e0
MWXS243064a_R1	Quality Control	N/A	2.172e5	N/A	N/A	0.0000	N/A
MWXS243064a_R2	Quality Control	N/A	2.207e5	N/A	N/A	0.0000	N/A
MWXS243064a_R3	Quality Control	N/A	2.224e5	N/A	N/A	0.0000	N/A
T24186682b_a	Unknown	N/A	2.103e5	N/A	N/A	N/A	N/A
T24186682b_b	Unknown	N/A	1.670e5	N/A	N/A	N/A	N/A
T24186682b_c	Unknown	N/A	1.830e5	N/A	N/A	N/A	N/A
T24186683b_a	Unknown	N/A	2.452e5	N/A	N/A	N/A	N/A
T24186683b_b	Unknown	N/A	2.437e5	N/A	N/A	N/A	N/A
T24186683b_c	Unknown	N/A	2.504e5	N/A	N/A	N/A	N/A
T24186684b_a	Unknown	N/A	2.130e5	N/A	N/A	N/A	N/A
T24186684b_b	Unknown	N/A	2.404e5	N/A	N/A	N/A	N/A
T24186684b_c	Unknown	N/A	2.153e5	N/A	N/A	N/A	N/A

Compound name: IAA-Val
Regression Equation: y = 0.67926 x + -2.35914e-4 (r = 0.99252) (weighting: 1 / x^2)

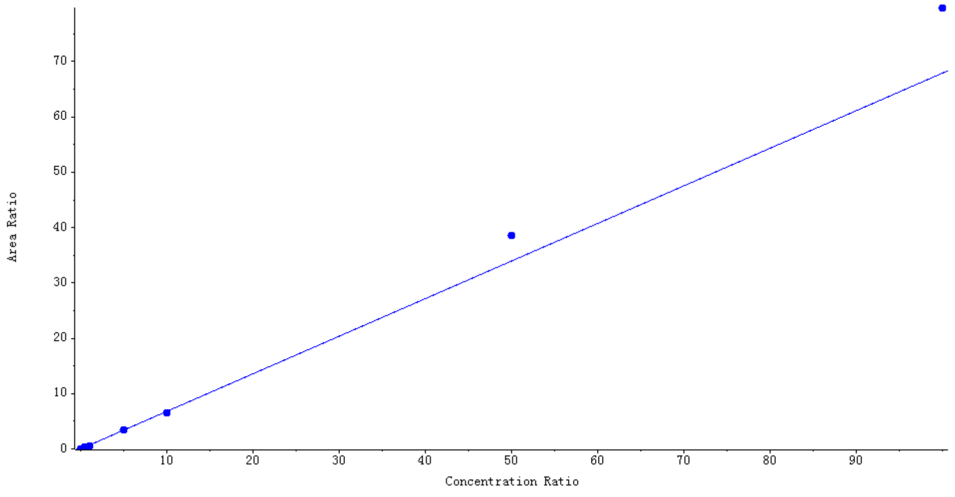

Peak Review

Blank
IAA-Val AREA:N/A S/N:N/A

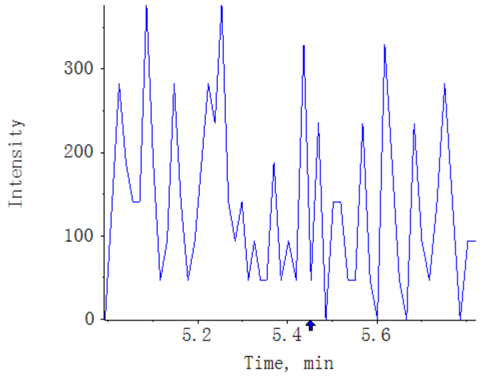

V3.0_MWMS_20240725_1
IAA-Val AREA:2.15e6 S/N:58.3

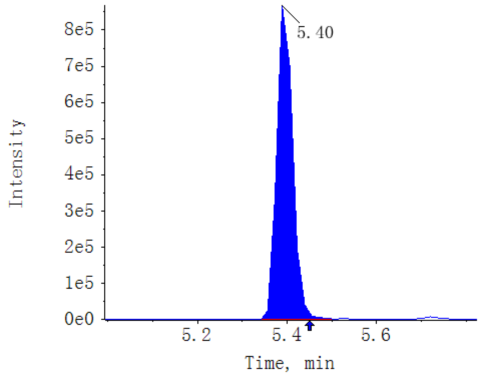

T24186682b_a
IAA-Val AREA:N/A S/N:N/A

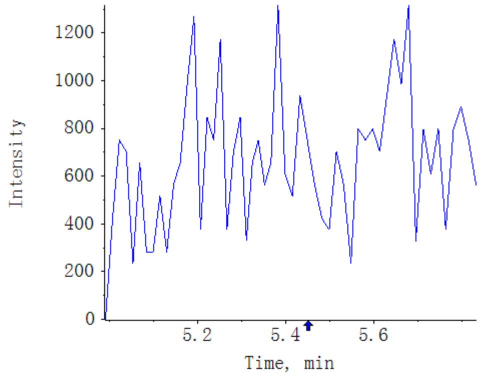

T24186682b_b
IAA-Val AREA:N/A S/N:N/A

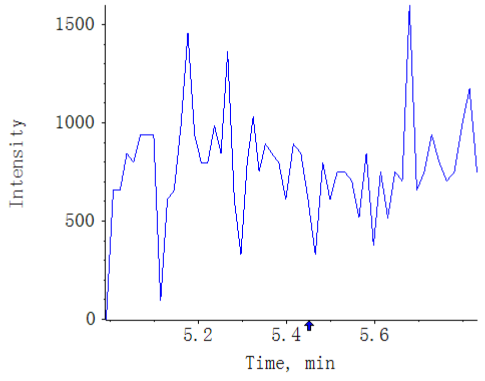

T24186682b_c
IAA-Val AREA:N/A S/N:N/A

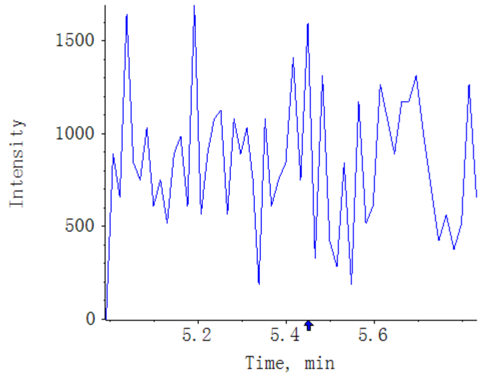

T24186683b_a
IAA-Val AREA:N/A S/N:N/A

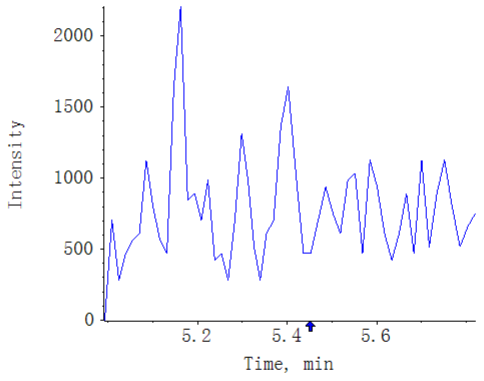

T24186683b_b
IAA-Val AREA:N/A S/N:N/A

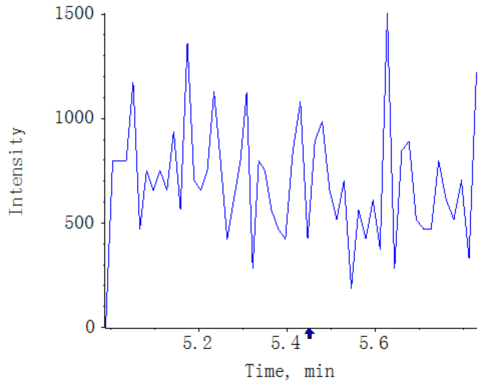

T24186683b_c
IAA-Val AREA:N/A S/N:N/A

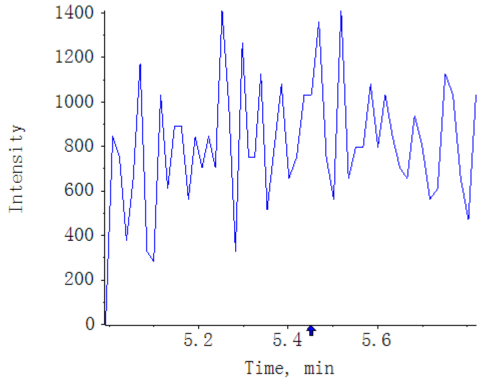

T24186684b_a
IAA-Val AREA:N/A S/N:N/A

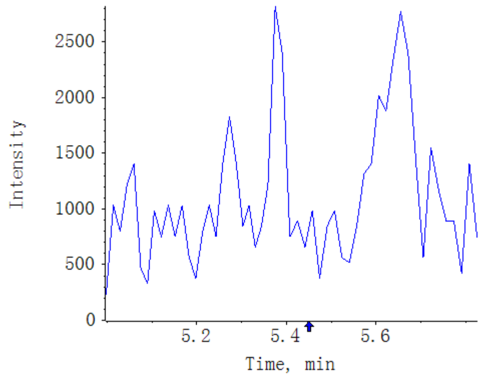

T24186684b_b
IAA-Val AREA:N/A S/N:N/A

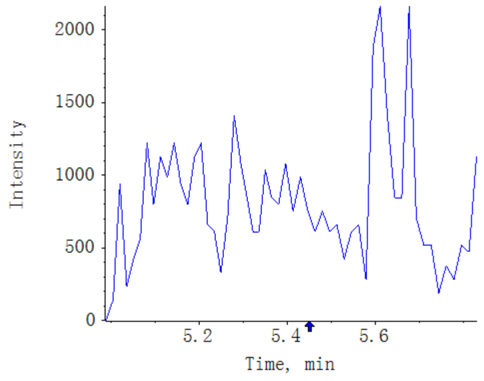

T24186684b_c
IAA-Val AREA:N/A S/N:N/A

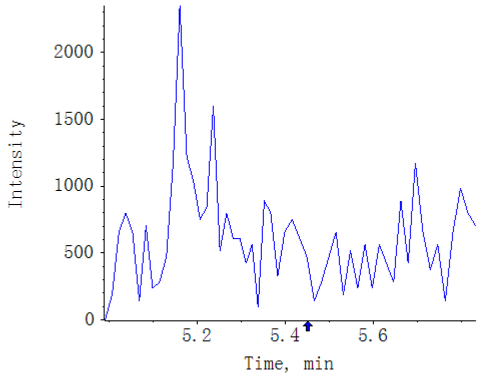

Result Table	MWXS-24-3064-a_9_WH6500-17_A20-3_V6.0_WSS_20240730	Algorithm Used	MQ4
Acquisition Method	ACC-PHs_V6.0_WH6500-17_CMY_20240521.dam	Instrument Name	Triple Quad 6500+ Low Mass
Project	N/A	Analytes QTY	109:9

Compound name: IAA-Phe-Me (337.0 / 130.2)

Sample Name	Sample Type	Area (cps)	Is Area (cps)	RT (min)	S/N	Target Conc	Calculated Conc.()
STD_0.01ppb	Standard	7.46e3	4.457e5	6.51	16.6	0.0100	9.833003e-3
STD_0.05ppb	Standard	4.34e4	4.759e5	6.50	32.3	0.0500	5.196544e-2
STD_0.1ppb	Standard	8.75e4	4.571e5	6.52	36.8	0.1000	1.087072e-1
STD_0.5ppb	Standard	4.27e5	4.822e5	6.51	77.2	0.5000	5.006959e-1
STD_1ppb	Standard	7.68e5	4.362e5	6.51	62.0	1.0000	9.963030e-1
STD_5ppb	Standard	4.38e6	4.553e5	6.52	70.1	5.0000	5.438095e0
STD_10ppb	Standard	7.44e6	4.191e5	6.51	65.7	10.0000	1.004542e1
STD_50ppb	Standard	2.81e7	3.976e5	6.50	50.6	50.0000	4.002316e1
STD_100ppb	Standard	N/A	3.709e5	N/A	N/A	100.0000	N/A
STD_200ppb	Standard	N/A	3.446e5	N/A	N/A	200.0000	N/A
STD_500ppb	Standard	N/A	2.675e5	N/A	N/A	500.0000	N/A
V2.0_MW_RQC1_20240724	Quality Control	N/A	1.104e5	N/A	N/A	0.0000	N/A
Blank	Unknown	N/A	3.683e3	N/A	N/A	N/A	N/A
V3.0_MWMS_20240725_1	Unknown	4.67e6	5.371e5	6.49	93.7	N/A	4.919856e0
MWXS243064a_R1	Quality Control	N/A	2.172e5	N/A	N/A	0.0000	N/A
MWXS243064a_R2	Quality Control	N/A	2.207e5	N/A	N/A	0.0000	N/A
MWXS243064a_R3	Quality Control	N/A	2.224e5	N/A	N/A	0.0000	N/A
T24186682b_a	Unknown	N/A	2.103e5	N/A	N/A	N/A	N/A
T24186682b_b	Unknown	N/A	1.670e5	N/A	N/A	N/A	N/A
T24186682b_c	Unknown	N/A	1.830e5	N/A	N/A	N/A	N/A
T24186683b_a	Unknown	N/A	2.452e5	N/A	N/A	N/A	N/A
T24186683b_b	Unknown	N/A	2.437e5	N/A	N/A	N/A	N/A
T24186683b_c	Unknown	N/A	2.504e5	N/A	N/A	N/A	N/A
T24186684b_a	Unknown	N/A	2.130e5	N/A	N/A	N/A	N/A
T24186684b_b	Unknown	N/A	2.404e5	N/A	N/A	N/A	N/A
T24186684b_c	Unknown	N/A	2.153e5	N/A	N/A	N/A	N/A

Compound name: IAA-Phe-Me
Regression Equation: y = 1.76793 x + -6.53239e-4 (r = 0.99552) (weighting: 1 / x^2)

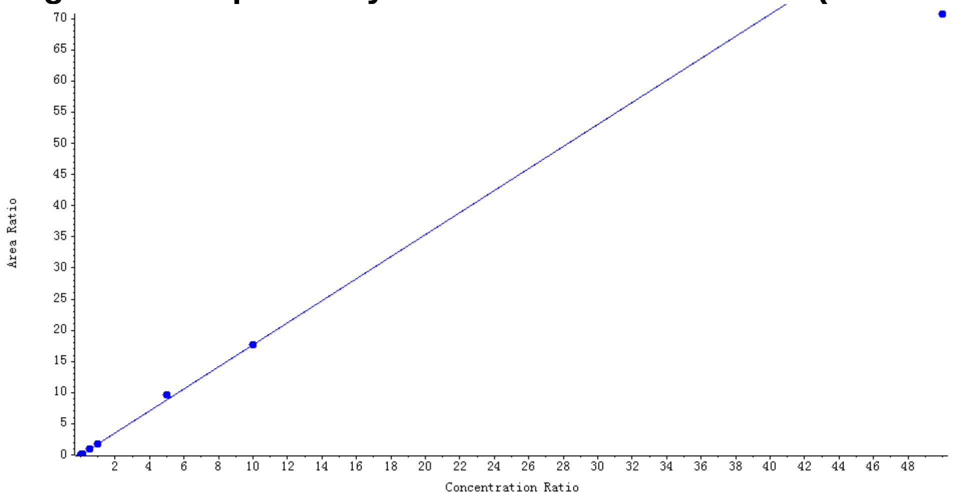

Peak Review

Blank

IAA-Phe-Me AREA:N/A S/N:N/A

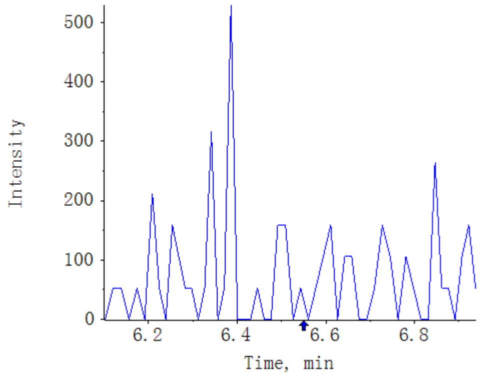

V3.0_MWMS_20240725_1

IAA-Phe-Me AREA:4.67e6 S/N:93.7

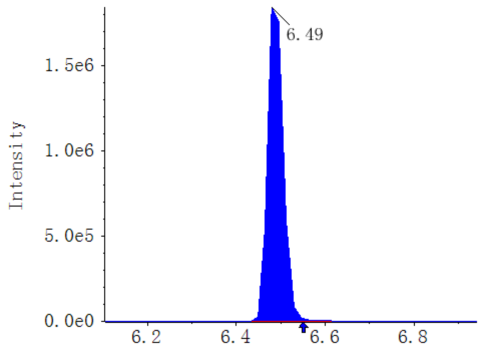

T24186682b_a

IAA-Phe-Me AREA:N/A S/N:N/A

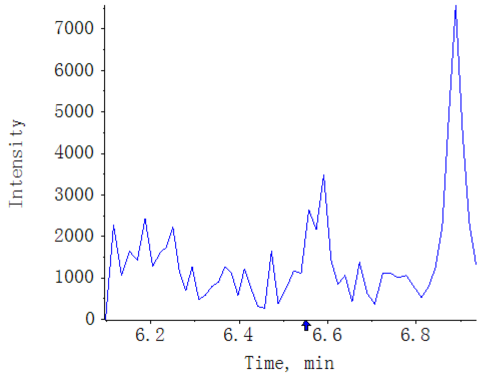

T24186682b_b

IAA-Phe-Me AREA:N/A S/N:N/A

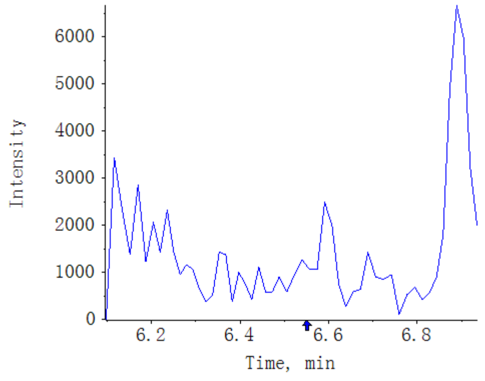

T24186682b_c

IAA-Phe-Me AREA:N/A S/N:N/A

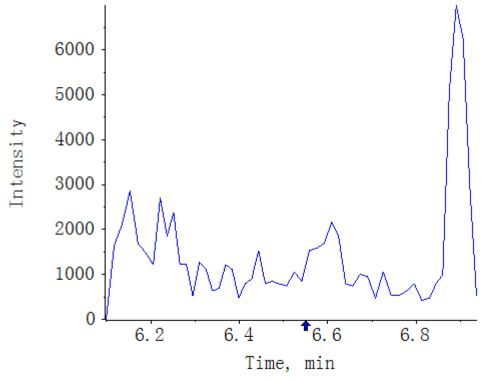

T24186683b_a

IAA-Phe-Me AREA:N/A S/N:N/A

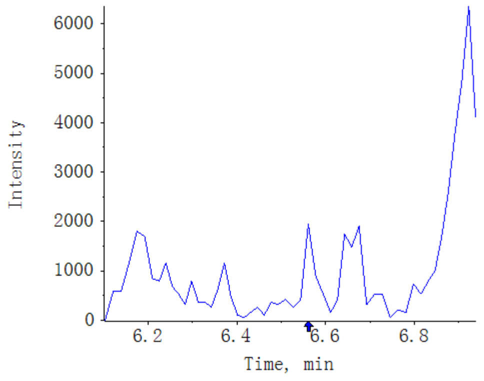

T24186683b_b

IAA-Phe-Me AREA:N/A S/N:N/A

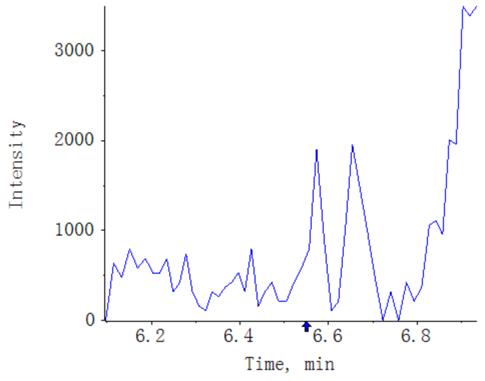

T24186683b_c

IAA-Phe-Me AREA:N/A S/N:N/A

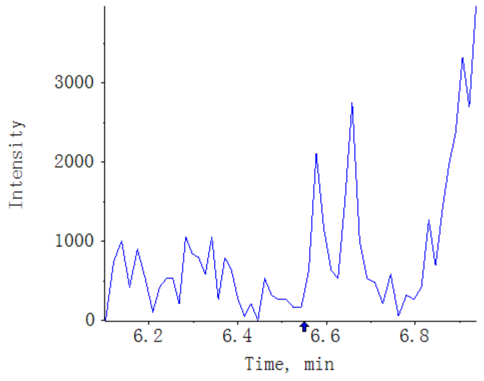

T24186684b_a

IAA-Phe-Me AREA:N/A S/N:N/A

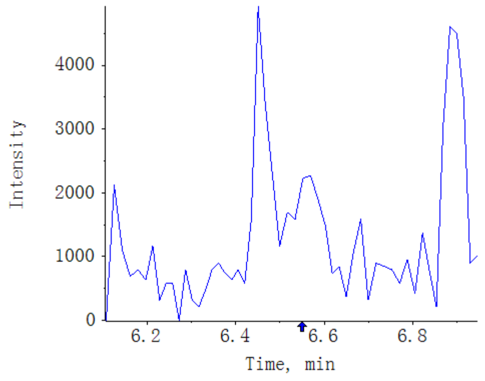

T24186684b_b

IAA-Phe-Me AREA:N/A S/N:N/A

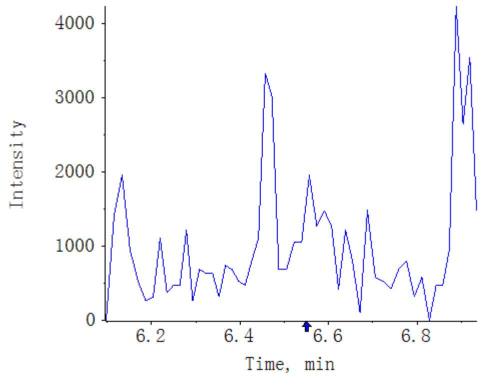

T24186684b_c

IAA-Phe-Me AREA:N/A S/N:N/A

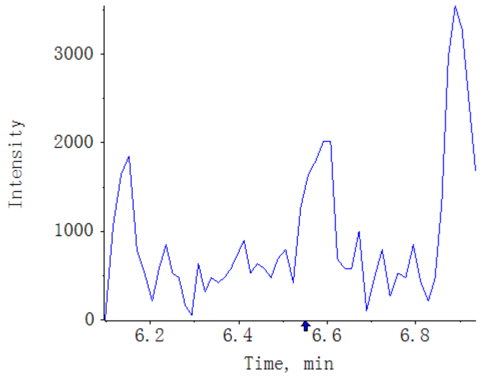

Result Table	MWXS-24-3064-a_9_WH6500-17_A20-3_V6.0_WSS_20240730	Algorithm Used	MQ4
Acquisition Method	ACC-PHs_V6.0_WH6500-17_CMY_20240521.dam	Instrument Name	Triple Quad 6500+ Low Mass
Project	N/A	Analytes QTY	109:10

Compound name: IAM (175.1 / 130.1)

Sample Name	Sample Type	Area (cps)	Is Area (cps)	RT (min)	S/N	Target Conc	Calculated Conc.()
STD_0.01ppb	Standard	N/A	1.428e6	N/A	N/A	0.0100	N/A
STD_0.05ppb	Standard	2.82e4	1.369e6	4.33	7.7	0.0500	5.402676e-2
STD_0.1ppb	Standard	4.56e4	1.463e6	4.33	15.2	0.1000	8.516211e-2
STD_0.5ppb	Standard	2.05e5	1.236e6	4.32	34.6	0.5000	4.803961e-1
STD_1ppb	Standard	3.87e5	1.201e6	4.32	39.9	1.0000	9.403266e-1
STD_5ppb	Standard	1.91e6	1.047e6	4.33	45.5	5.0000	5.360488e0
STD_10ppb	Standard	3.23e6	1.000e6	4.32	43.2	10.0000	9.491006e0
STD_50ppb	Standard	1.40e7	7.656e5	4.32	50.4	50.0000	5.372242e1
STD_100ppb	Standard	2.32e7	6.846e5	4.33	39.7	100.0000	9.954842e1
STD_200ppb	Standard	4.22e7	5.732e5	4.32	50.0	200.0000	2.167000e2
STD_500ppb	Standard	8.47e7	5.024e5	4.32	50.1	500.0000	4.960470e2
V2.0_MW_RQC1_20240724	Quality Control	N/A	2.415e5	N/A	N/A	0.0000	N/A
Blank	Unknown	N/A	4.437e3	N/A	N/A	N/A	N/A
V3.0_MWMS_20240725_1	Unknown	2.17e6	1.067e6	4.32	30.7	N/A	5.984684e0
MWXS243064a_R1	Quality Control	N/A	3.904e5	N/A	N/A	0.0000	N/A
MWXS243064a_R2	Quality Control	N/A	4.074e5	N/A	N/A	0.0000	N/A
MWXS243064a_R3	Quality Control	N/A	4.359e5	N/A	N/A	0.0000	N/A
T24186682b_a	Unknown	N/A	4.121e5	N/A	N/A	N/A	N/A
T24186682b_b	Unknown	N/A	4.393e5	N/A	N/A	N/A	N/A
T24186682b_c	Unknown	N/A	3.635e5	N/A	N/A	N/A	N/A
T24186683b_a	Unknown	N/A	4.505e5	N/A	N/A	N/A	N/A
T24186683b_b	Unknown	N/A	4.569e5	N/A	N/A	N/A	N/A
T24186683b_c	Unknown	N/A	4.754e5	N/A	N/A	N/A	N/A
T24186684b_a	Unknown	N/A	4.133e5	N/A	N/A	N/A	N/A
T24186684b_b	Unknown	N/A	4.404e5	N/A	N/A	N/A	N/A
T24186684b_c	Unknown	N/A	4.001e5	N/A	N/A	N/A	N/A

Compound name: IAM
Regression Equation: y = 0.33995 x + 0.00223 (r = 0.99656) (weighting: 1 / x^2)

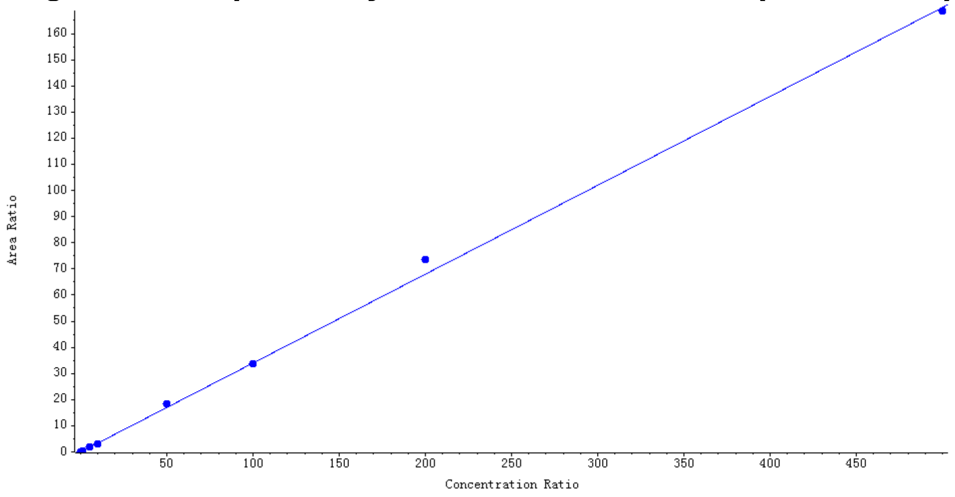

Peak Review

Blank
IAM AREA:N/A S/N:N/A

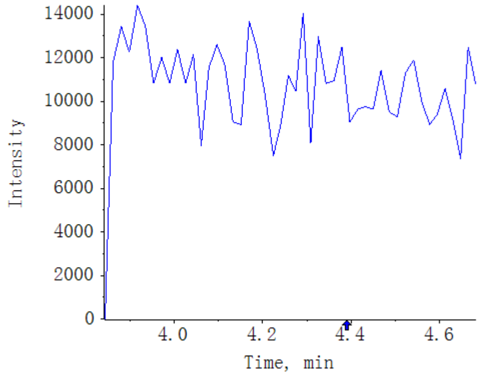

V3.0_MWMS_20240725_1
IAM AREA:2.17e6 S/N:30.7

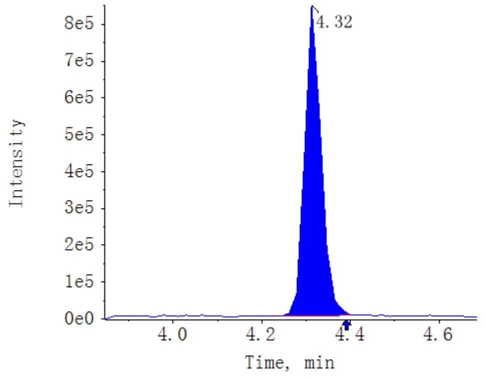

T24186682b_a
IAM AREA:N/A S/N:N/A

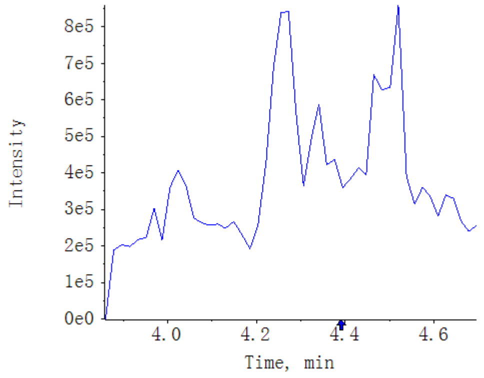

T24186682b_b
IAM AREA:N/A S/N:N/A

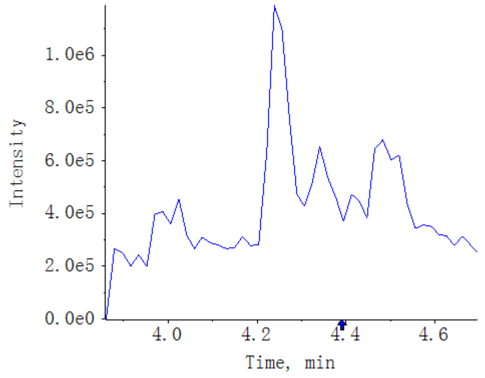

T24186682b_c
IAM AREA:N/A S/N:N/A

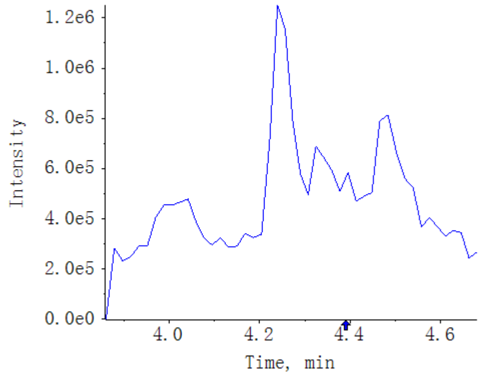

T24186683b_a
IAM AREA:N/A S/N:N/A

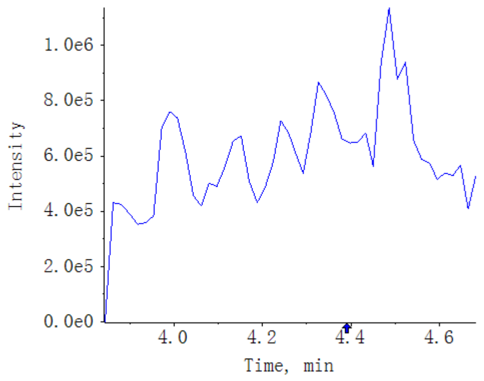

T24186683b_b
IAM AREA:N/A S/N:N/A

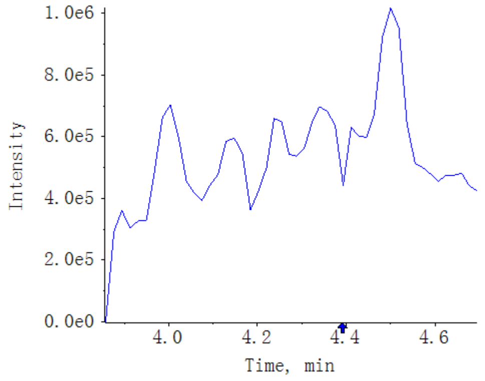

T24186683b_c
IAM AREA:N/A S/N:N/A

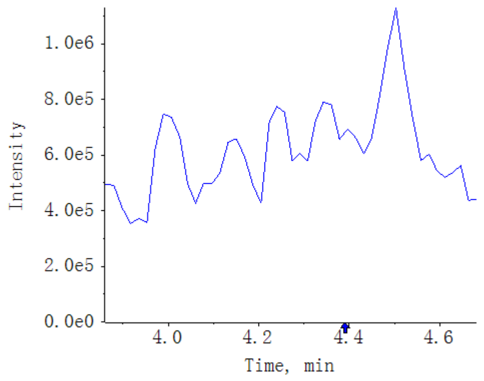

T24186684b_a
IAM AREA:N/A S/N:N/A

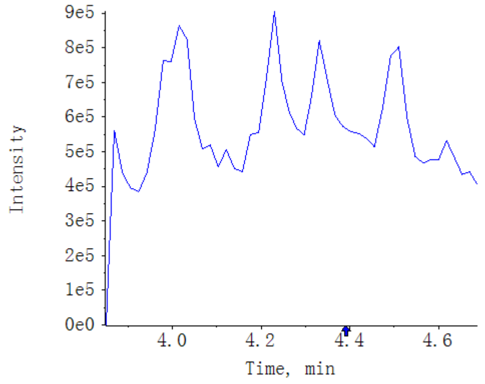

T24186684b_b
IAM AREA:N/A S/N:N/A

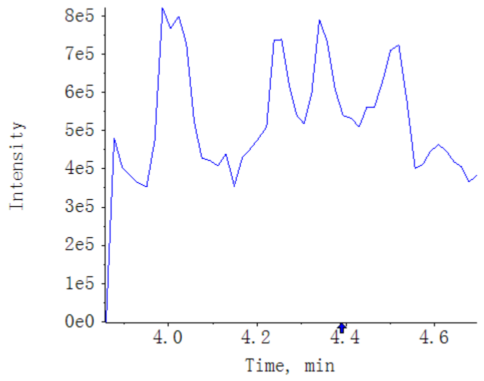

T24186684b_c
IAM AREA:N/A S/N:N/A

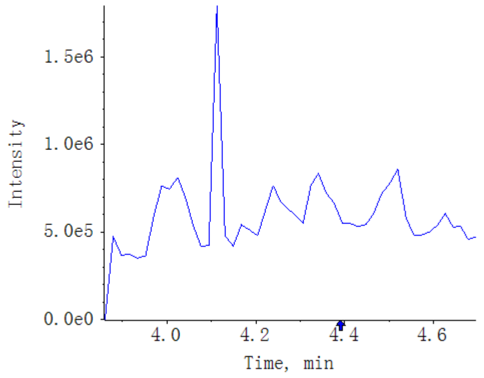

Result Table	MWXS-24-3064-a_9_WH6500-17_A20-3_V6.0_WSS_20240730	Algorithm Used	MQ4
Acquisition Method	ACC-PHs_V6.0_WH6500-17_CMY_20240521.dam	Instrument Name	Triple Quad 6500+ Low Mass
Project	N/A	Analytes QTY	109:11

Compound name: TRA (161.1 / 144.1)

Sample Name	Sample Type	Area (cps)	Is Area (cps)	RT (min)	S/N	Target Conc	Calculated Conc.()
STD_0.01ppb	Standard	N/A	1.534e6	N/A	N/A	0.0100	N/A
STD_0.05ppb	Standard	1.34e4	1.769e6	3.64	7.1	0.0500	7.204467e-2
STD_0.1ppb	Standard	2.43e4	1.783e6	3.65	8.4	0.1000	1.132279e-1
STD_0.5ppb	Standard	8.73e4	1.410e6	3.64	18.9	0.5000	4.426903e-1
STD_1ppb	Standard	1.67e5	1.448e6	3.64	24.3	1.0000	8.091584e-1
STD_5ppb	Standard	1.01e6	1.440e6	3.65	27.8	5.0000	4.814402e0
STD_10ppb	Standard	1.74e6	1.372e6	3.64	25.1	10.0000	8.656667e0
STD_50ppb	Standard	8.10e6	1.121e6	3.64	29.0	50.0000	4.931040e1
STD_100ppb	Standard	1.43e7	1.080e6	3.64	27.3	100.0000	9.020962e1
STD_200ppb	Standard	2.86e7	9.910e5	3.64	28.4	200.0000	1.969980e2
STD_500ppb	Standard	6.80e7	9.008e5	3.64	30.2	500.0000	5.152238e2
V2.0_MW_RQC1_20240724	Quality Control	4.09e5	6.482e5	3.65	161.7	0.0000	4.318812e0
Blank	Unknown	N/A	3.159e3	N/A	N/A	N/A	N/A
V3.0_MWMS_20240725_1	Unknown	9.10e5	9.644e5	3.65	460.7	N/A	6.458038e0
MWXS243064a_R1	Quality Control	N/A	7.282e5	N/A	N/A	0.0000	N/A
MWXS243064a_R2	Quality Control	N/A	7.565e5	N/A	N/A	0.0000	N/A
MWXS243064a_R3	Quality Control	N/A	7.725e5	N/A	N/A	0.0000	N/A
T24186682b_a	Unknown	N/A	7.382e5	N/A	N/A	N/A	N/A
T24186682b_b	Unknown	N/A	7.882e5	N/A	N/A	N/A	N/A
T24186682b_c	Unknown	N/A	7.476e5	N/A	N/A	N/A	N/A
T24186683b_a	Unknown	N/A	7.865e5	N/A	N/A	N/A	N/A
T24186683b_b	Unknown	N/A	7.983e5	N/A	N/A	N/A	N/A
T24186683b_c	Unknown	N/A	7.056e5	N/A	N/A	N/A	N/A
T24186684b_a	Unknown	N/A	7.244e5	N/A	N/A	N/A	N/A
T24186684b_b	Unknown	N/A	8.341e5	N/A	N/A	N/A	N/A
T24186684b_c	Unknown	N/A	7.332e5	N/A	N/A	N/A	N/A

Compound name: TRA
Regression Equation: y = 0.14662 x + -0.00299 (r = 0.99901) (weighting: 1 / x)

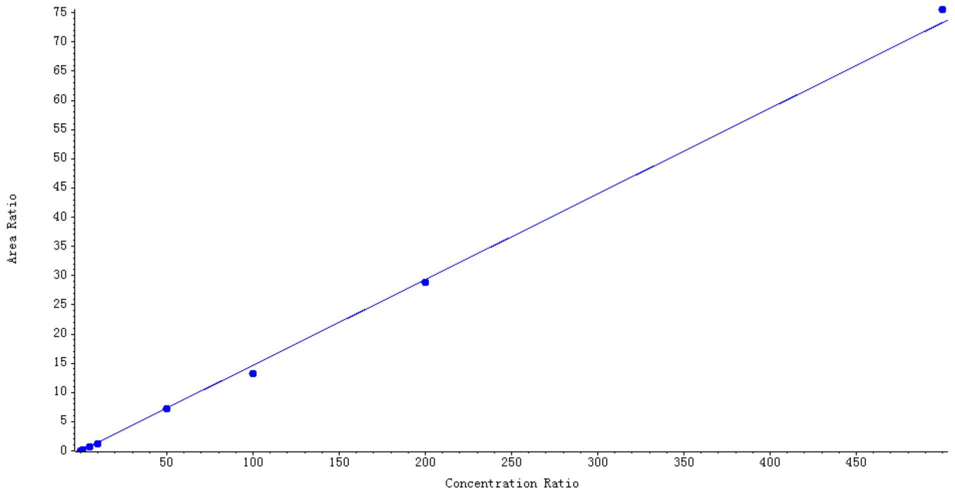

Peak Review

Blank
TRA AREA:N/A S/N:N/A

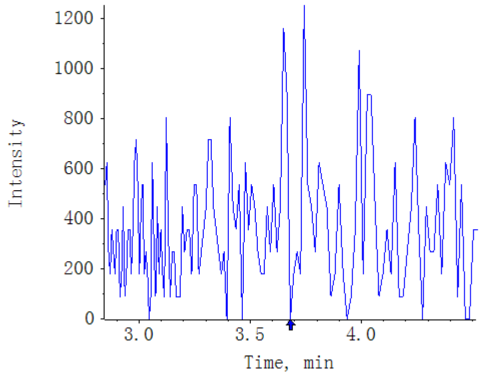

V3.0_MWMS_20240725_1
TRA AREA:9.10e5 S/N:460.7

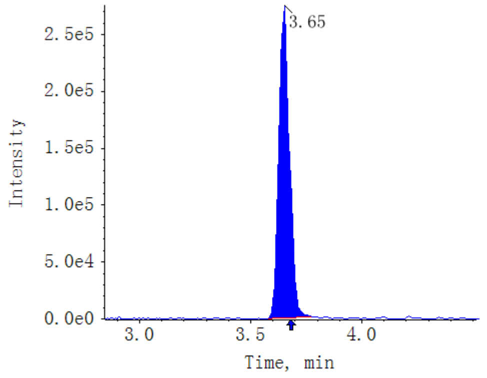

T24186682b_a
TRA AREA:N/A S/N:N/A

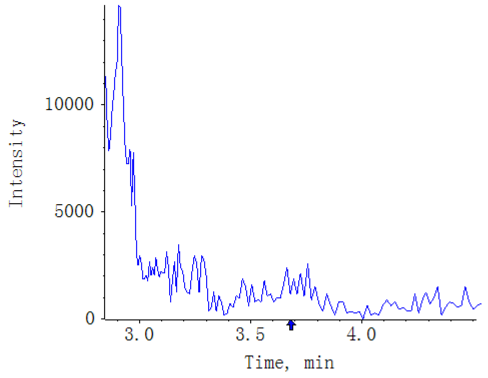

T24186682b_b
TRA AREA:N/A S/N:N/A

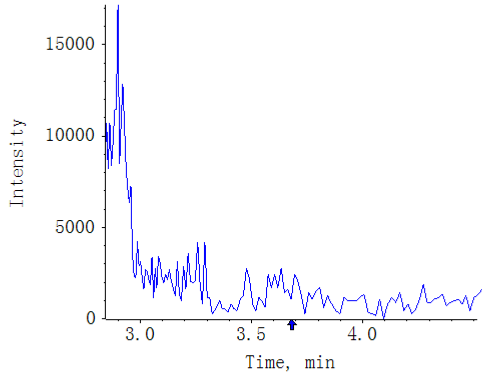

T24186682b_c
TRA AREA:N/A S/N:N/A

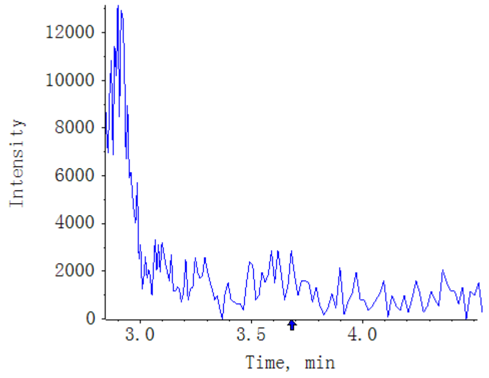

T24186683b_a
TRA AREA:N/A S/N:N/A

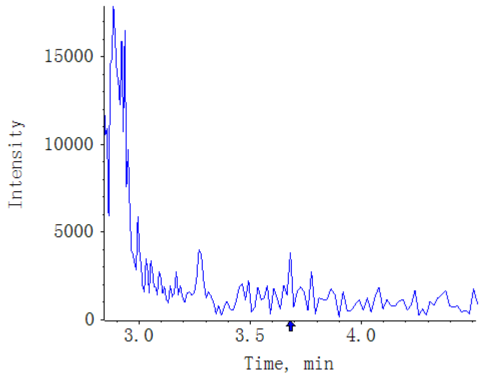

T24186683b_b
TRA AREA:N/A S/N:N/A

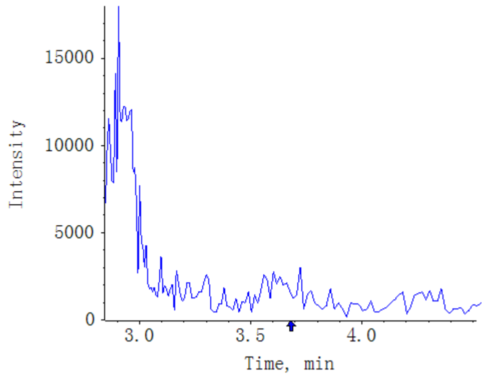

T24186683b_c
TRA AREA:N/A S/N:N/A

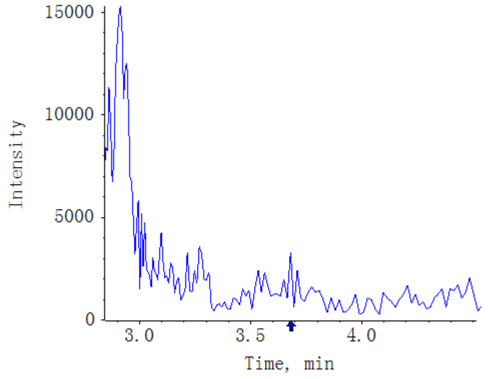

T24186684b_a
TRA AREA:N/A S/N:N/A

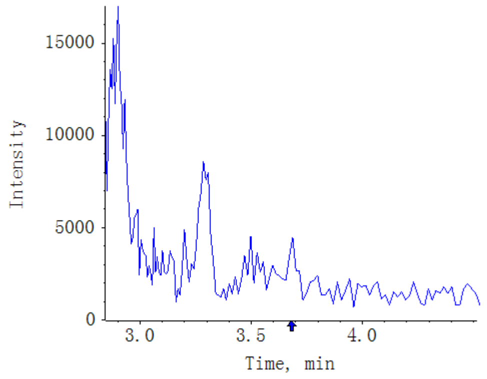

T24186684b_b
TRA AREA:N/A S/N:N/A

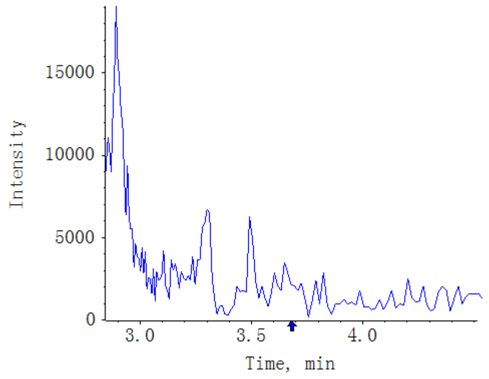

T24186684b_c
TRA AREA:N/A S/N:N/A

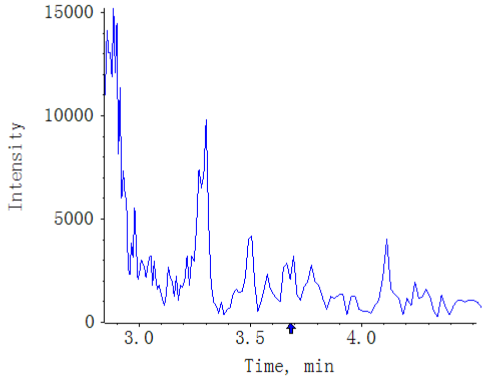

Result Table	MWXS-24-3064-a_9_WH6500-17_A20-3_V6.0_WSS_20240730	Algorithm Used	MQ4
Acquisition Method	ACC-PHs_V6.0_WH6500-17_CMY_20240521.dam	Instrument Name	Triple Quad 6500+ Low Mass
Project	N/A	Analytes QTY	109:12

Compound name: IA (188.1 / 170.1)

Sample Name	Sample Type	Area (cps)	Is Area (cps)	RT (min)	S/N	Target Conc	Calculated Conc.()
STD_0.01ppb	Standard	N/A	1.463e7	N/A	N/A	0.0100	N/A
STD_0.05ppb	Standard	1.54e4	1.538e7	5.28	10.8	0.0500	7.389112e-2
STD_0.1ppb	Standard	1.87e4	1.466e7	5.28	10.6	0.1000	9.530903e-2
STD_0.5ppb	Standard	8.98e4	1.438e7	5.27	39.5	0.5000	4.870747e-1
STD_1ppb	Standard	1.55e5	1.446e7	5.28	38.6	1.0000	8.391360e-1
STD_5ppb	Standard	8.65e5	1.392e7	5.29	58.6	5.0000	4.888966e0
STD_10ppb	Standard	1.56e6	1.335e7	5.28	42.6	10.0000	9.231178e0
STD_50ppb	Standard	8.42e6	1.326e7	5.27	50.6	50.0000	5.002590e1
STD_100ppb	Standard	1.52e7	1.370e7	5.28	45.1	100.0000	8.750670e1
STD_200ppb	Standard	3.10e7	1.327e7	5.28	62.9	200.0000	1.841008e2
STD_500ppb	Standard	6.60e7	9.829e6	5.28	69.3	500.0000	5.294011e2
V2.0_MW_RQC1_20240724	Quality Control	N/A	2.323e6	N/A	N/A	0.0000	N/A
Blank	Unknown	N/A	3.627e3	N/A	N/A	N/A	N/A
V3.0_MWMS_20240725_1	Unknown	1.01e6	1.365e7	5.25	52.1	N/A	5.837715e0
MWXS243064a_R1	Quality Control	N/A	1.937e6	N/A	N/A	0.0000	N/A
MWXS243064a_R2	Quality Control	N/A	2.063e6	N/A	N/A	0.0000	N/A
MWXS243064a_R3	Quality Control	N/A	2.038e6	N/A	N/A	0.0000	N/A
T24186682b_a	Unknown	N/A	1.307e6	N/A	N/A	N/A	N/A
T24186682b_b	Unknown	N/A	1.262e6	N/A	N/A	N/A	N/A
T24186682b_c	Unknown	N/A	1.267e6	N/A	N/A	N/A	N/A
T24186683b_a	Unknown	N/A	3.941e6	N/A	N/A	N/A	N/A
T24186683b_b	Unknown	N/A	4.032e6	N/A	N/A	N/A	N/A
T24186683b_c	Unknown	N/A	3.923e6	N/A	N/A	N/A	N/A
T24186684b_a	Unknown	N/A	2.919e6	N/A	N/A	N/A	N/A
T24186684b_b	Unknown	N/A	2.979e6	N/A	N/A	N/A	N/A
T24186684b_c	Unknown	N/A	3.050e6	N/A	N/A	N/A	N/A

Compound name: IA
Regression Equation: y = 0.01269 x + 6.46384e-5 (r = 0.99732) (weighting: 1 / x)

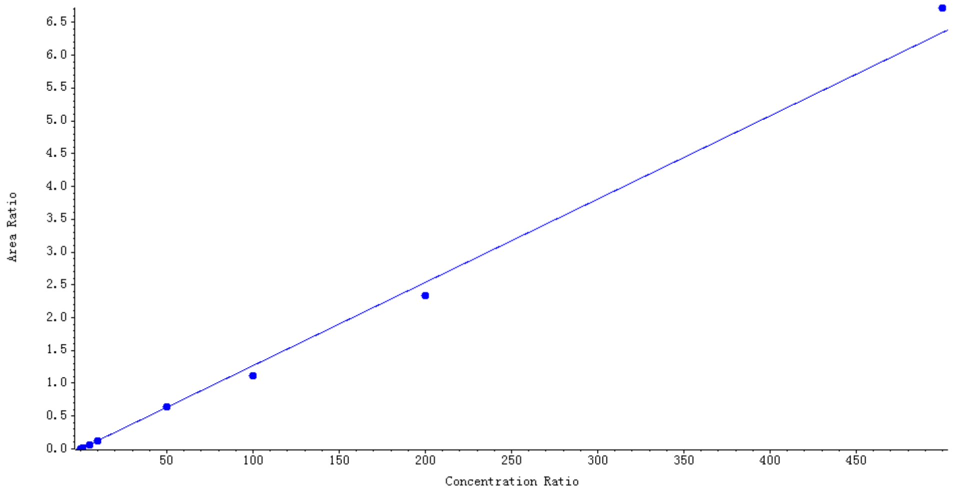

Peak Review

Blank
IA AREA:N/A S/N:N/A

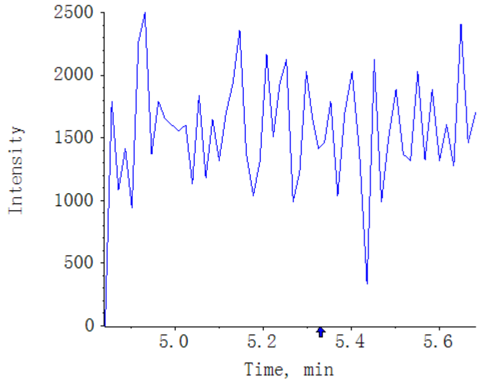

V3.0_MWMS_20240725_1
IA AREA:1.01e6 S/N:52.1

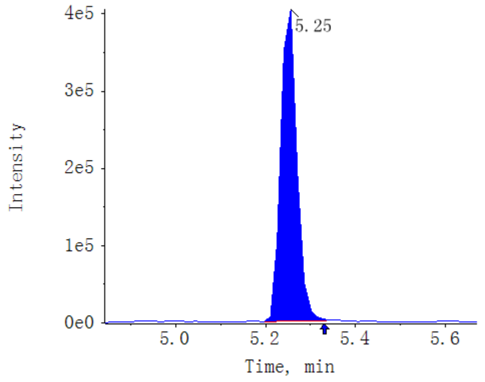

T24186682b_a
IA AREA:N/A S/N:N/A

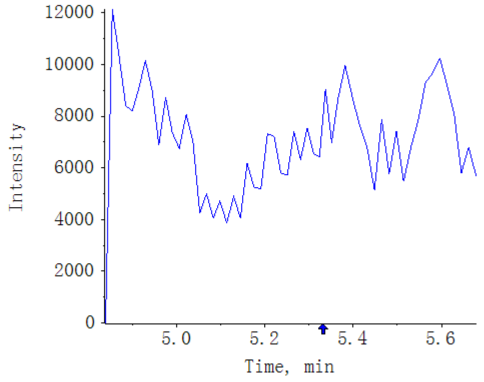

T24186682b_b
IA AREA:N/A S/N:N/A

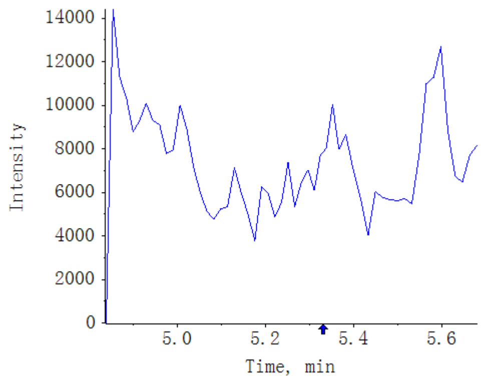

T24186682b_c
IA AREA:N/A S/N:N/A

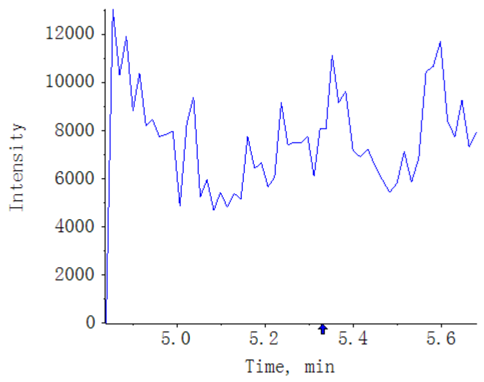

T24186683b_a
IA AREA:N/A S/N:N/A

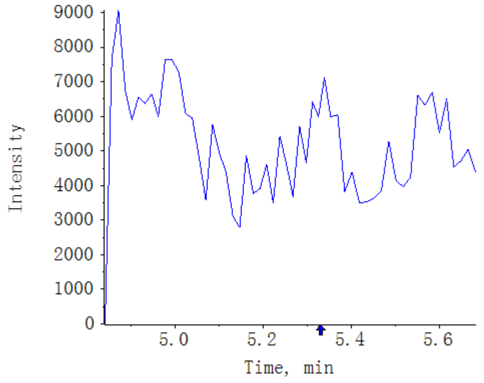

T24186683b_b
IA AREA:N/A S/N:N/A

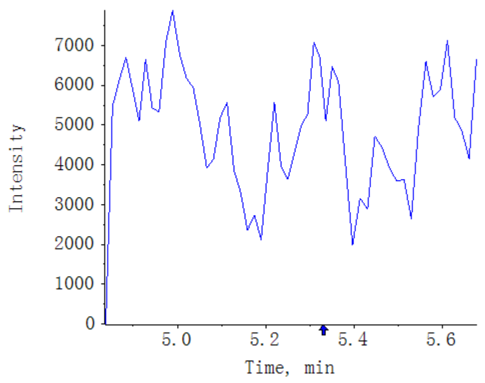

T24186683b_c
IA AREA:N/A S/N:N/A

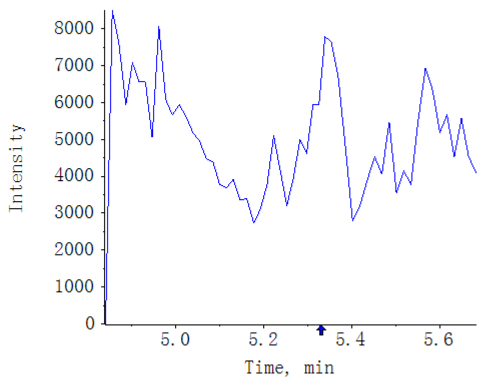

T24186684b_a
IA AREA:N/A S/N:N/A

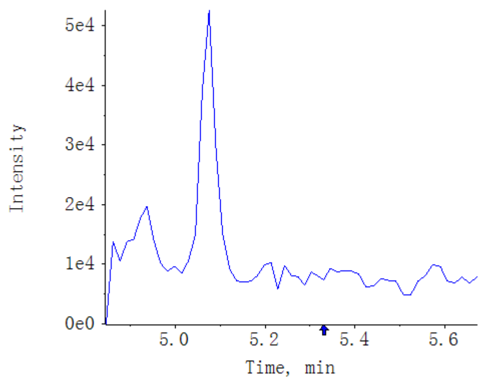

T24186684b_b
IA AREA:N/A S/N:N/A

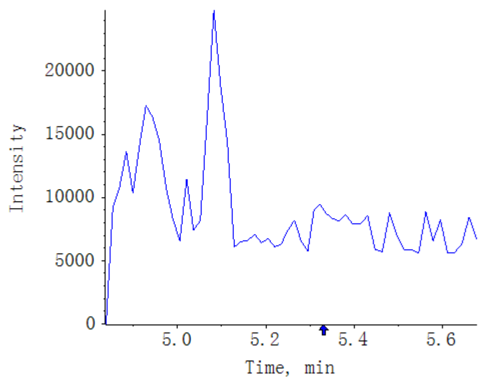

T24186684b_c
IA AREA:N/A S/N:N/A

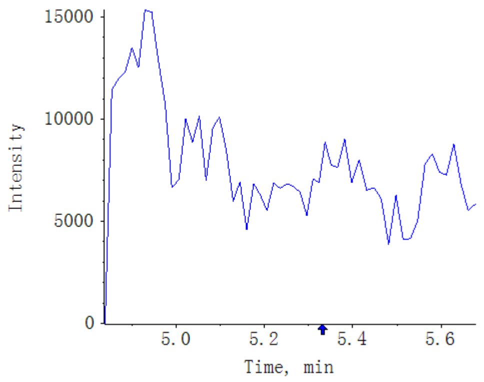

Result Table	MWXS-24-3064-a_9_WH6500-17_A20-3_V6.0_WSS_20240730	Algorithm Used	MQ4
Acquisition Method	ACC-PHs_V6.0_WH6500-17_CMY_20240521.dam	Instrument Name	Triple Quad 6500+ Low Mass
Project	N/A	Analytes QTY	109:13

Compound name: IAA-Ala (247.1 / 130.1)

Sample Name	Sample Type	Area (cps)	Is Area (cps)	RT (min)	S/N	Target Conc	Calculated Conc.()
STD_0.01ppb	Standard	N/A	9.585e5	N/A	N/A	0.0100	N/A
STD_0.05ppb	Standard	1.19e4	1.005e6	4.72	14.5	0.0500	7.419927e-2
STD_0.1ppb	Standard	1.88e4	1.006e6	4.74	18.7	0.1000	1.066554e-1
STD_0.5ppb	Standard	8.49e4	9.144e5	4.72	46.3	0.5000	4.606234e-1
STD_1ppb	Standard	1.58e5	9.133e5	4.73	43.7	1.0000	8.433922e-1
STD_5ppb	Standard	8.53e5	9.379e5	4.74	48.3	5.0000	4.360365e0
STD_10ppb	Standard	1.61e6	8.988e5	4.73	48.5	10.0000	8.566667e0
STD_50ppb	Standard	8.34e6	8.221e5	4.72	46.0	50.0000	4.844792e1
STD_100ppb	Standard	1.56e7	7.937e5	4.74	51.5	100.0000	9.363452e1
STD_200ppb	Standard	3.27e7	7.421e5	4.73	46.2	200.0000	2.101557e2
STD_500ppb	Standard	N/A	6.587e5	N/A	N/A	500.0000	N/A
V2.0_MW_RQC1_20240724	Quality Control	N/A	1.859e5	N/A	N/A	0.0000	N/A
Blank	Unknown	N/A	1.994e3	N/A	N/A	N/A	N/A
V3.0_MWMS_20240725_1	Unknown	1.28e6	1.067e6	4.72	45.4	N/A	5.750486e0
MWXS243064a_R1	Quality Control	N/A	3.954e5	N/A	N/A	0.0000	N/A
MWXS243064a_R2	Quality Control	N/A	4.267e5	N/A	N/A	0.0000	N/A
MWXS243064a_R3	Quality Control	N/A	3.851e5	N/A	N/A	0.0000	N/A
T24186682b_a	Unknown	N/A	4.183e5	N/A	N/A	N/A	N/A
T24186682b_b	Unknown	N/A	3.609e5	N/A	N/A	N/A	N/A
T24186682b_c	Unknown	N/A	3.933e5	N/A	N/A	N/A	N/A
T24186683b_a	Unknown	N/A	4.544e5	N/A	N/A	N/A	N/A
T24186683b_b	Unknown	N/A	4.851e5	N/A	N/A	N/A	N/A
T24186683b_c	Unknown	N/A	4.599e5	N/A	N/A	N/A	N/A
T24186684b_a	Unknown	N/A	4.216e5	N/A	N/A	N/A	N/A
T24186684b_b	Unknown	N/A	4.540e5	N/A	N/A	N/A	N/A
T24186684b_c	Unknown	N/A	4.275e5	N/A	N/A	N/A	N/A

Compound name: IAA-Ala
Regression Equation: y = 0.20948 x + -0.00369 (r = 0.99823) (weighting: 1 / x)

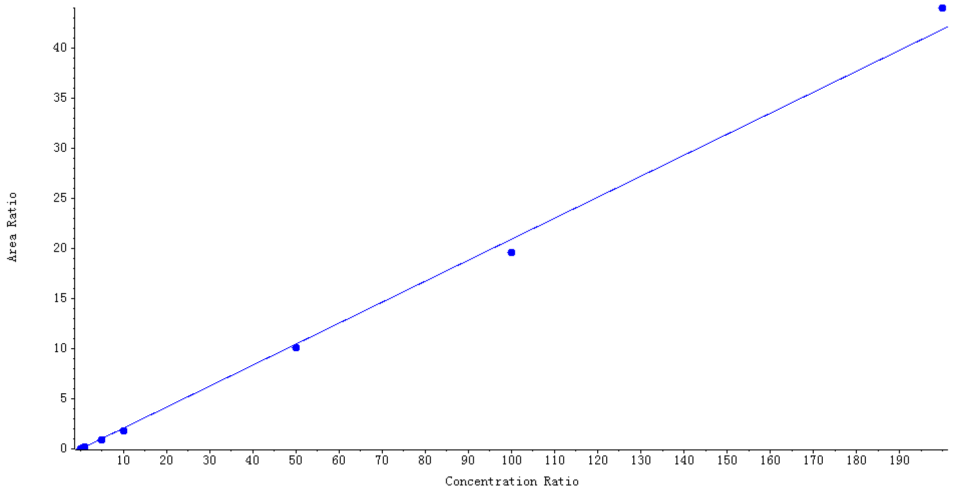

Peak Review

Blank

IAA-Ala AREA:N/A S/N:N/A

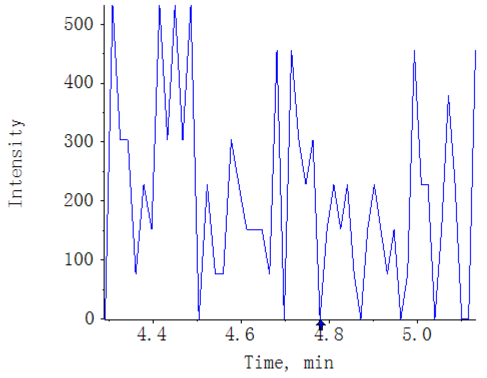

V3.0_MWMS_20240725_1

IAA-Ala AREA:1.28e6 S/N:45.4

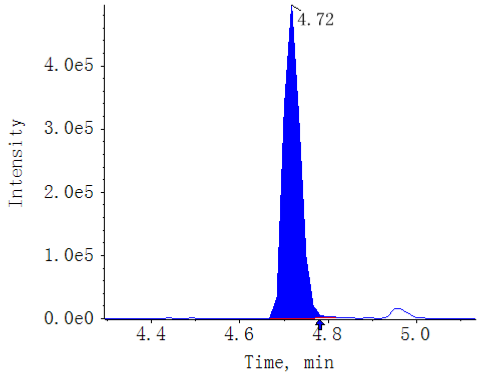

T24186682b_a

IAA-Ala AREA:N/A S/N:N/A

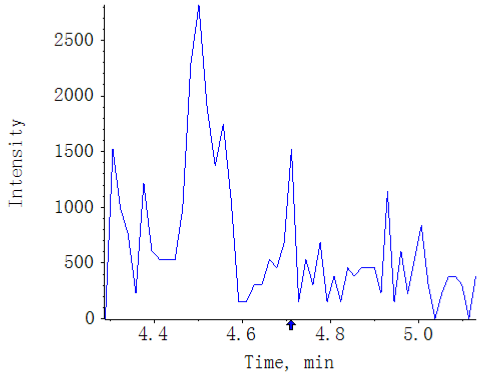

T24186682b_b

IAA-Ala AREA:N/A S/N:N/A

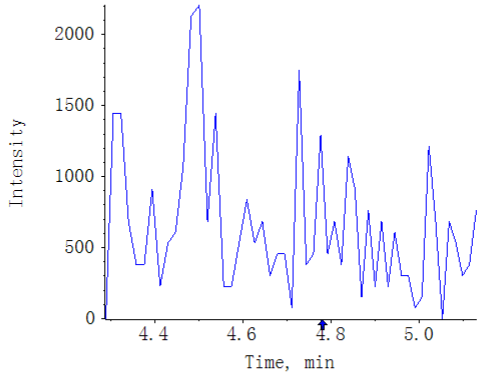

T24186682b_c

IAA-Ala AREA:N/A S/N:N/A

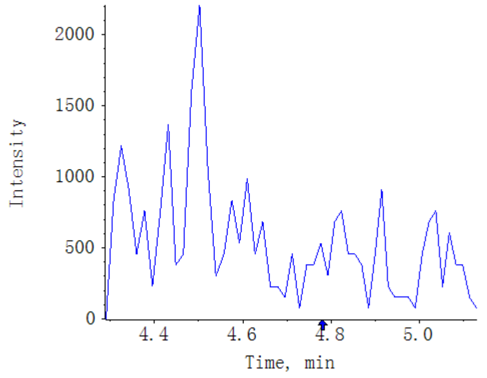

T24186683b_a

IAA-Ala AREA:N/A S/N:N/A

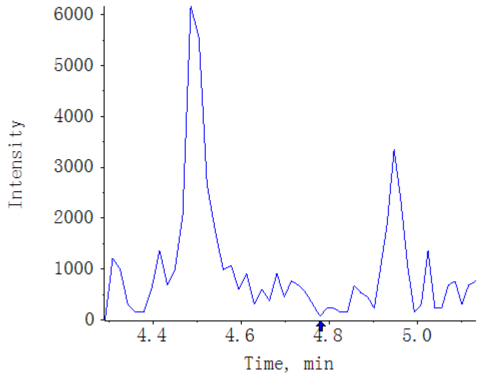

T24186683b_b

IAA-Ala AREA:N/A S/N:N/A

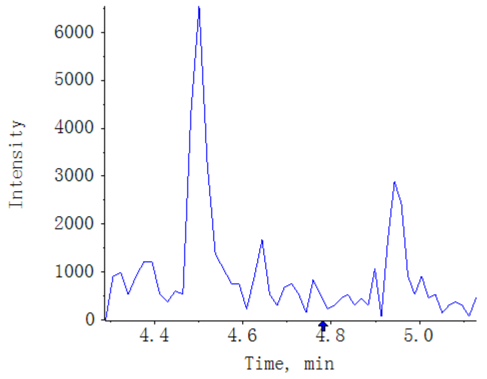

T24186683b_c

IAA-Ala AREA:N/A S/N:N/A

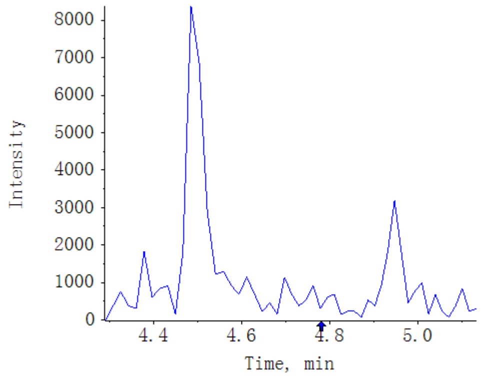

T24186684b_a

IAA-Ala AREA:N/A S/N:N/A

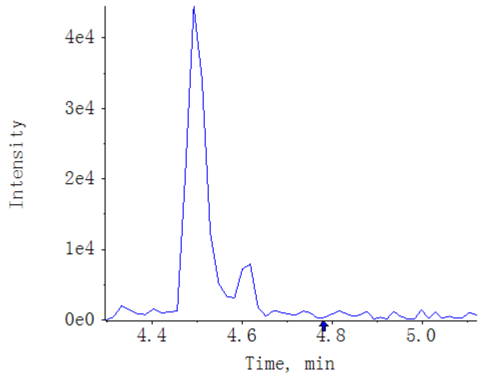

T24186684b_b

IAA-Ala AREA:N/A S/N:N/A

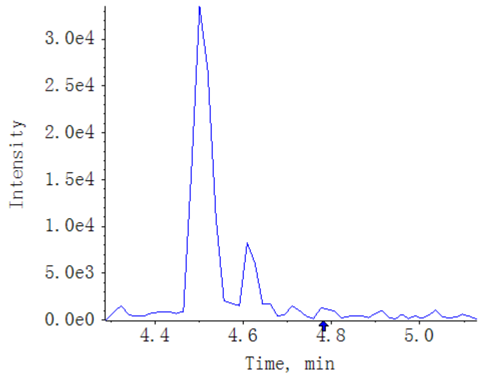

T24186684b_c

IAA-Ala AREA:N/A S/N:N/A

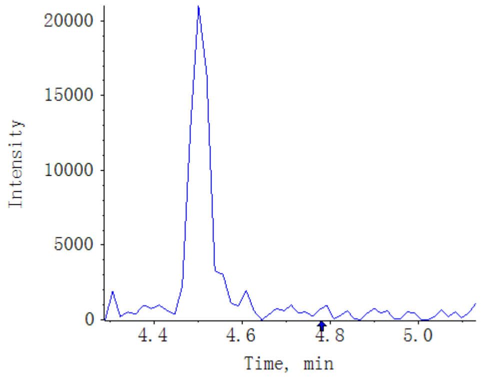

Result Table	MWXS-24-3064-a_9_WH6500-17_A20-3_V6.0_WSS_20240730	Algorithm Used	MQ4
Acquisition Method	ACC-PHs_V6.0_WH6500-17_CMY_20240521.dam	Instrument Name	Triple Quad 6500+ Low Mass
Project	N/A	Analytes QTY	109:14

Compound name: TRP (205.1 / 188.1)

Sample Name	Sample Type	Area (cps)	Is Area (cps)	RT (min)	S/N	Target Conc	Calculated Conc.()
STD_0.01ppb	Standard	N/A	6.185e5	N/A	N/A	0.2000	N/A
STD_0.05ppb	Standard	N/A	6.574e5	N/A	N/A	1.0000	N/A
STD_0.1ppb	Standard	1.47e4	6.581e5	3.25	24.4	2.0000	2.340581e0
STD_0.5ppb	Standard	3.68e4	5.613e5	3.25	67.1	10.0000	9.778168e0
STD_1ppb	Standard	6.72e4	5.806e5	3.24	128.8	20.0000	1.839432e1
STD_5ppb	Standard	3.31e5	5.534e5	3.26	548.5	100.0000	1.013267e2
STD_10ppb	Standard	5.76e5	5.236e5	3.25	702.9	200.0000	1.876257e2
STD_50ppb	Standard	3.11e6	5.248e5	3.24	3176.2	1000.0000	1.017347e3
STD_100ppb	Standard	5.81e6	5.270e5	3.25	5782.9	2000.0000	1.893445e3
STD_200ppb	Standard	1.17e7	4.958e5	3.24	8458.3	4000.0000	4.043580e3
STD_500ppb	Standard	2.48e7	4.236e5	3.24	13374.4	10000.0000	1.005816e4
V2.0_MW_RQC1_20240724	Quality Control	7.51e6	5.051e5	3.31	4361.0	0.0000	2.554827e3
Blank	Unknown	N/A	1.319e4	N/A	N/A	N/A	N/A
V3.0_MWMS_20240725_1	Unknown	4.84e5	6.073e5	3.31	684.7	N/A	1.355261e2
MWXS243064a_R1	Quality Control	3.83e6	6.170e5	3.28	1961.2	0.0000	1.065230e3
MWXS243064a_R2	Quality Control	3.93e6	6.373e5	3.29	3088.8	0.0000	1.059370e3
MWXS243064a_R3	Quality Control	3.93e6	6.126e5	3.27	1555.2	0.0000	1.101129e3
T24186682b_a	Unknown	7.08e5	6.402e5	3.29	748.5	N/A	1.884881e2
T24186682b_b	Unknown	6.83e5	6.471e5	3.29	596.2	N/A	1.799003e2
T24186682b_c	Unknown	8.32e5	8.121e5	3.30	828.8	N/A	1.746604e2
T24186683b_a	Unknown	7.52e5	6.467e5	3.29	647.8	N/A	1.985202e2
T24186683b_b	Unknown	6.63e5	6.747e5	3.30	378.9	N/A	1.673089e2
T24186683b_c	Unknown	7.05e5	7.310e5	3.30	416.3	N/A	1.642712e2
T24186684b_a	Unknown	1.09e7	6.033e5	3.29	5639.6	N/A	3.114903e3
T24186684b_b	Unknown	9.11e6	6.968e5	3.30	6048.9	N/A	2.245412e3
T24186684b_c	Unknown	1.12e7	5.910e5	3.29	6566.6	N/A	3.245484e3

Compound name: TRP
Regression Equation: y = 0.00582 x + 0.00866 (r = 0.99977) (weighting: 1 / x)

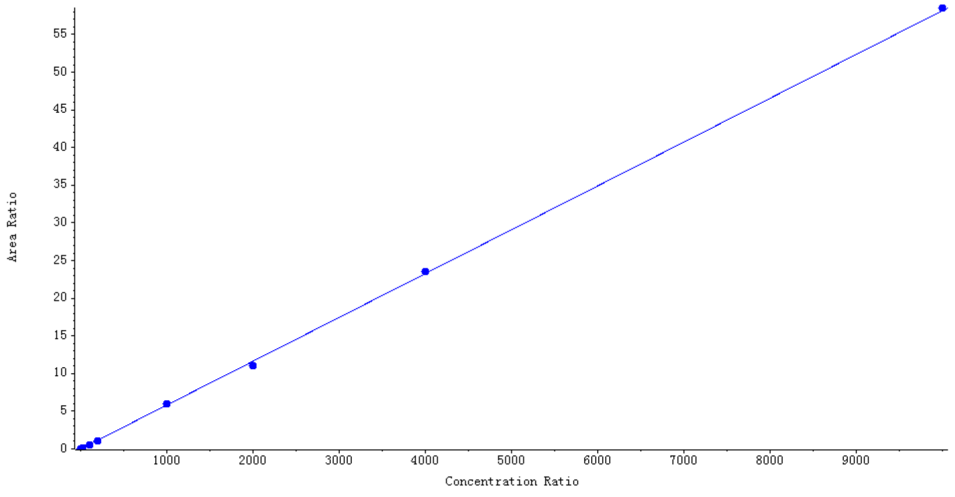

Peak Review

Blank

TRP AREA:N/A S/N:N/A

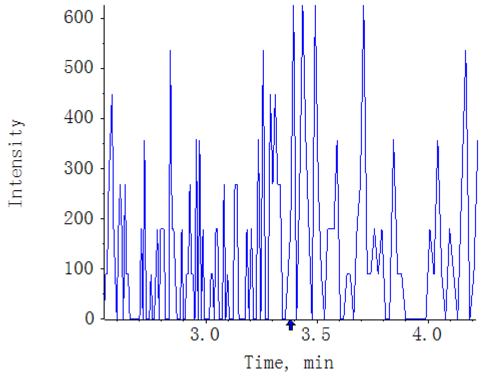

V3.0_MWMS_20240725_1

TRP AREA:4.84e5 S/N:684.7

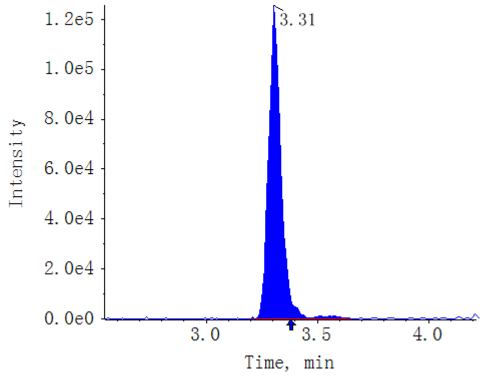

T24186682b_a

TRP AREA:7.08e5 S/N:748.5

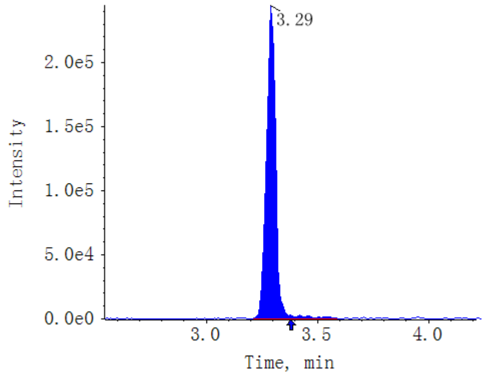

T24186682b_b

TRP AREA:6.83e5 S/N:596.2

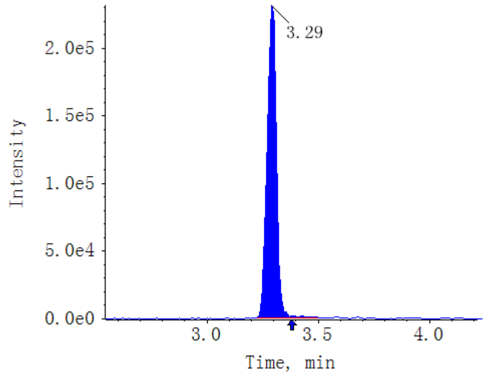

T24186682b_c

TRP AREA:8.32e5 S/N:828.8

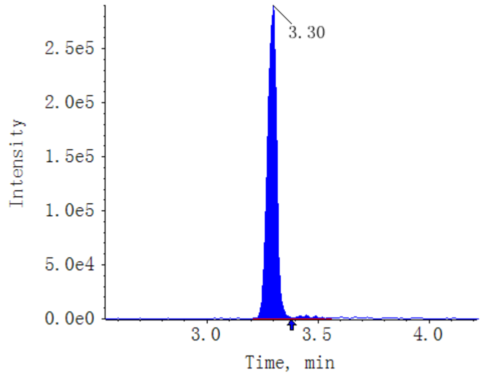

T24186683b_a

TRP AREA:7.52e5 S/N:647.8

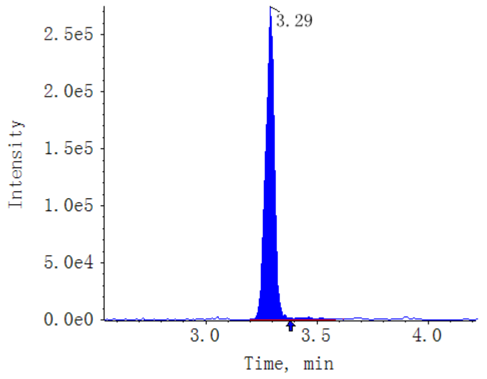

T24186683b_b

TRP AREA:6.63e5 S/N:378.9

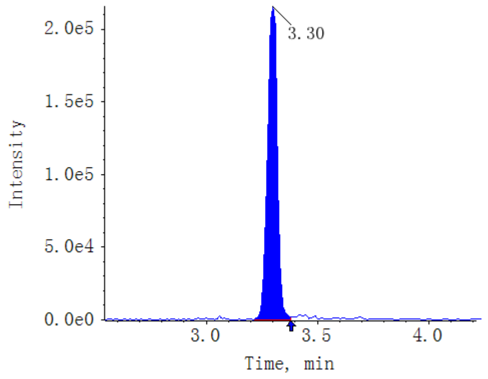

T24186683b_c

TRP AREA:7.05e5 S/N:416.3

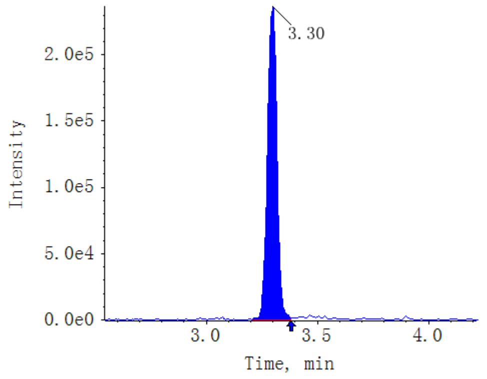

T24186684b_a

TRP AREA:1.09e7 S/N:5639.6

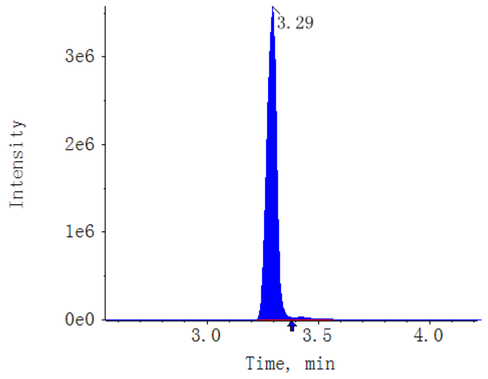

T24186684b_b

TRP AREA:9.11e6 S/N:6048.9

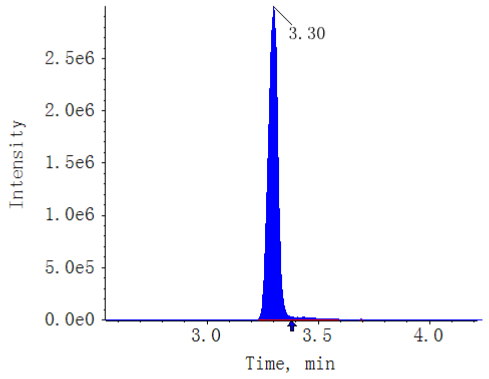

T24186684b_c

TRP AREA:1.12e7 S/N:6566.6

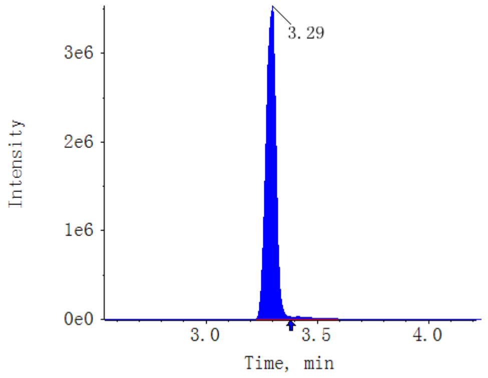

Result Table	MWXS-24-3064-a_9_WH6500-17_A20-3_V6.0_WSS_20240730	Algorithm Used	MQ4
Acquisition Method	ACC-PHs_V6.0_WH6500-17_CMY_20240521.dam	Instrument Name	Triple Quad 6500+ Low Mass
Project	N/A	Analytes QTY	109:15

Compound name: IAA (176.1 / 130.1)

Sample Name	Sample Type	Area (cps)	Is Area (cps)	RT (min)	S/N	Target Conc	Calculated Conc.()
STD_0.01ppb	Standard	6.19e3	1.463e7	5.04	5.3	0.0100	1.051687e-2
STD_0.05ppb	Standard	1.31e4	1.538e7	5.04	10.6	0.0500	5.088375e-2
STD_0.1ppb	Standard	2.26e4	1.466e7	5.05	17.9	0.1000	1.155261e-1
STD_0.5ppb	Standard	8.12e4	1.438e7	5.04	34.7	0.5000	4.998724e-1
STD_1ppb	Standard	1.37e5	1.446e7	5.04	36.9	1.0000	8.616467e-1
STD_5ppb	Standard	7.76e5	1.392e7	5.05	51.0	5.0000	5.195327e0
STD_10ppb	Standard	1.30e6	1.335e7	5.04	69.8	10.0000	9.080466e0
STD_50ppb	Standard	7.13e6	1.326e7	5.04	51.3	50.0000	5.037943e1
STD_100ppb	Standard	1.36e7	1.370e7	5.05	65.4	100.0000	9.326045e1
STD_200ppb	Standard	2.89e7	1.327e7	5.04	55.9	200.0000	2.040898e2
STD_500ppb	Standard	5.28e7	9.829e6	5.04	49.0	500.0000	5.031161e2
V2.0_MW_RQC1_20240724	Quality Control	2.45e5	2.323e6	5.01	10.8	0.0000	9.837834e0
Blank	Unknown	N/A	3.627e3	N/A	N/A	N/A	N/A
V3.0_MWMS_20240725_1	Unknown	9.17e5	1.365e7	5.02	60.9	N/A	6.271557e0
MWXS243064a_R1	Quality Control	6.18e4	1.937e6	5.01	5.8	0.0000	2.963368e0
MWXS243064a_R2	Quality Control	5.83e4	2.063e6	5.02	7.9	0.0000	2.617719e0
MWXS243064a_R3	Quality Control	5.68e4	2.038e6	5.00	6.7	0.0000	2.583292e0
T24186682b_a	Unknown	3.08e4	1.307e6	5.02	6.2	N/A	2.176049e0
T24186682b_b	Unknown	3.67e4	1.262e6	5.02	6.0	N/A	2.699753e0
T24186682b_c	Unknown	3.52e4	1.267e6	5.02	5.2	N/A	2.573968e0
T24186683b_a	Unknown	9.20e4	3.941e6	5.01	9.6	N/A	2.158249e0
T24186683b_b	Unknown	1.13e5	4.032e6	5.01	11.2	N/A	2.607475e0
T24186683b_c	Unknown	1.20e5	3.923e6	5.01	15.0	N/A	2.847346e0
T24186684b_a	Unknown	1.01e5	2.919e6	5.01	10.2	N/A	3.229113e0
T24186684b_b	Unknown	8.29e4	2.979e6	5.02	11.6	N/A	2.580034e0
T24186684b_c	Unknown	8.06e4	3.050e6	5.02	8.8	N/A	2.449027e0

Compound name: IAA
Regression Equation: y = 0.01067 x + 3.11167e-4 (r = 0.99961) (weighting: 1 / x)

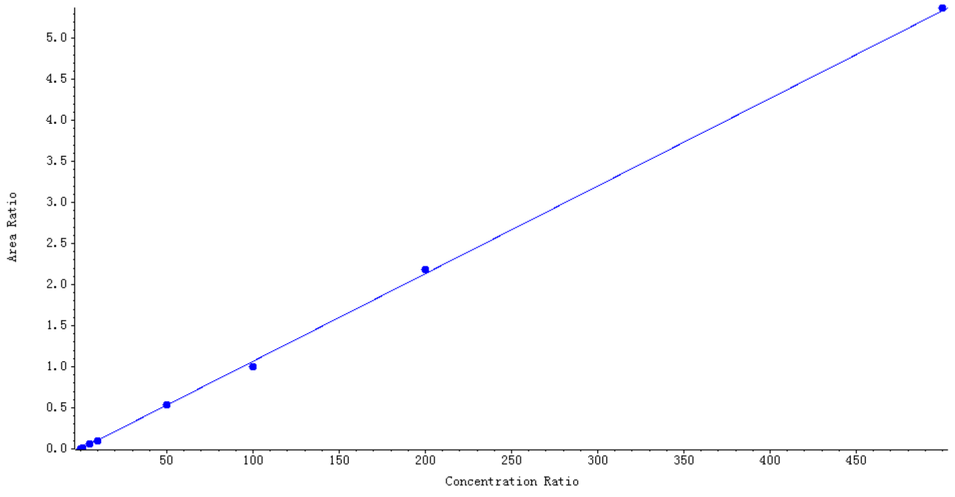

Peak Review

Blank

IAA AREA:N/A S/N:N/A

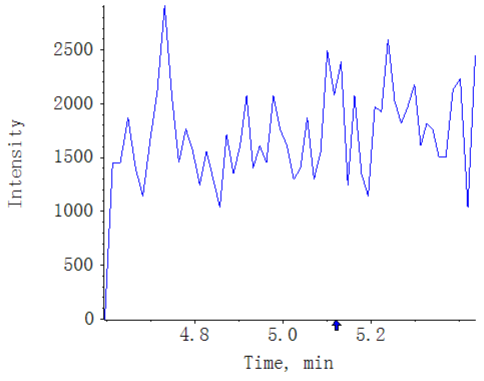

V3.0_MWMS_20240725_1

IAA AREA:9.17e5 S/N:60.9

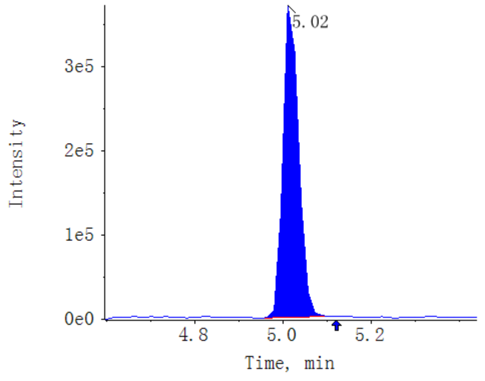

T24186682b_a

IAA AREA:3.08e4 S/N:6.2

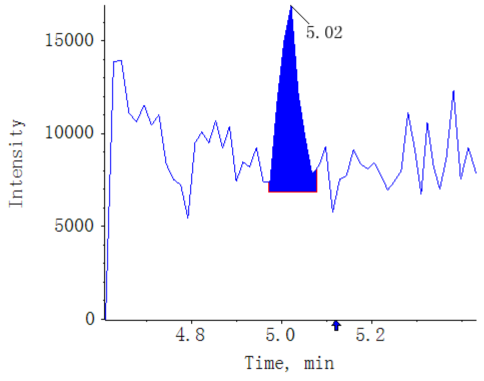

T24186682b_b

IAA AREA:3.67e4 S/N:6.0

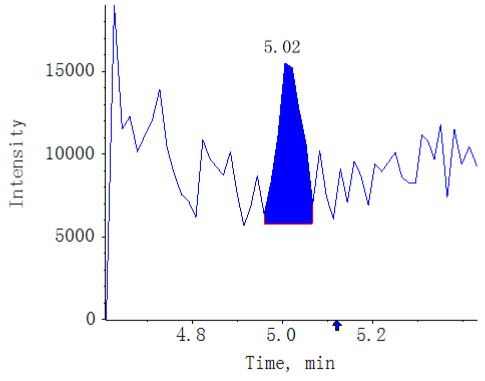

T24186682b_c

IAA AREA:3.52e4 S/N:5.2

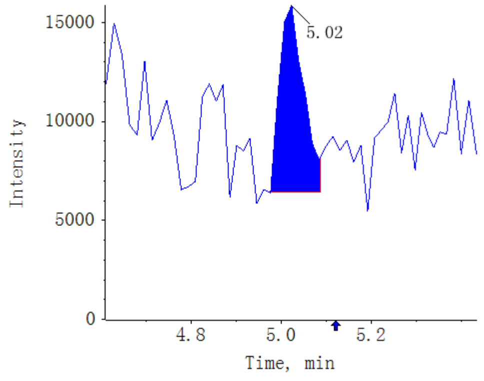

T24186683b_a

IAA AREA:9.20e4 S/N:9.6

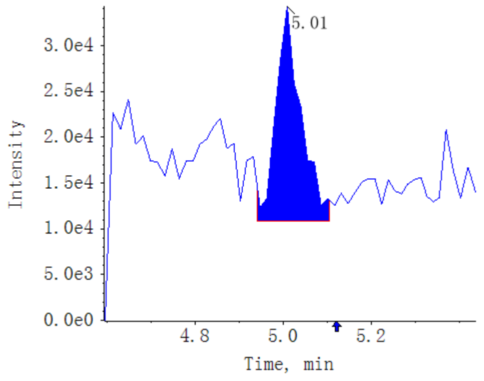

T24186683b_b

IAA AREA:1.13e5 S/N:11.2

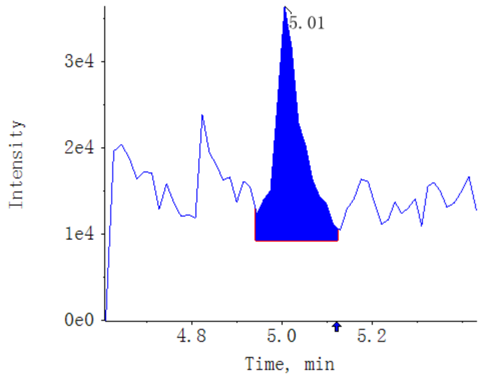

T24186683b_c

IAA AREA:1.20e5 S/N:15.0

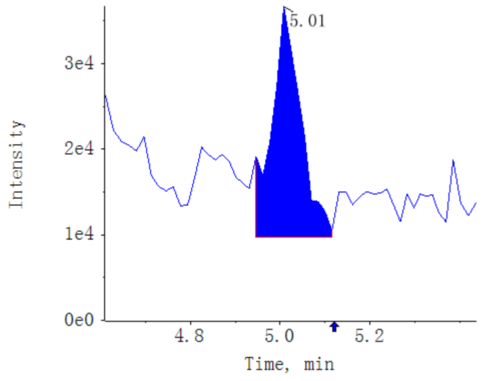

T24186684b_a

IAA AREA:1.01e5 S/N:10.2

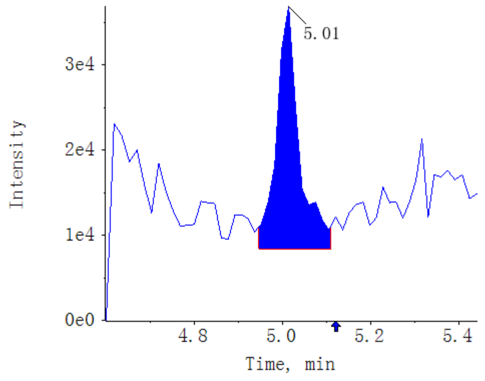

T24186684b_b

IAA AREA:8.29e4 S/N:11.6

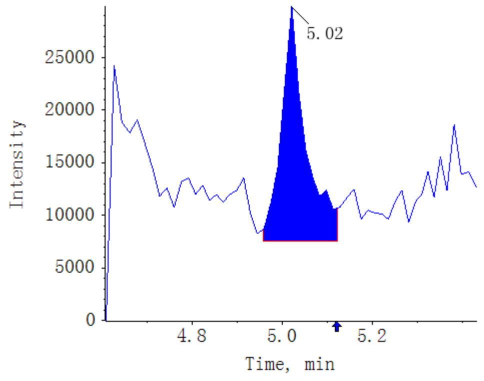

T24186684b_c

IAA AREA:8.06e4 S/N:8.8

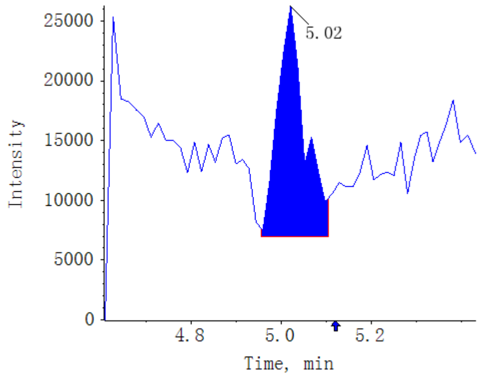

Result Table	MWXS-24-3064-a_9_WH6500-17_A20-3_V6.0_WSS_20240730	Algorithm Used	MQ4
Acquisition Method	ACC-PHs_V6.0_WH6500-17_CMY_20240521.dam	Instrument Name	Triple Quad 6500+ Low Mass
Project	N/A	Analytes QTY	109:16

Compound name: IBA (204.3 / 130.2)

Sample Name	Sample Type	Area (cps)	Is Area (cps)	RT (min)	S/N	Target Conc	Calculated Conc.()
STD_0.01ppb	Standard	N/A	3.231e6	N/A	N/A	0.0100	N/A
STD_0.05ppb	Standard	N/A	3.439e6	N/A	N/A	0.0500	N/A
STD_0.1ppb	Standard	1.31e5	3.359e6	5.79	36.5	0.1000	1.017595e-1
STD_0.5ppb	Standard	1.49e5	3.169e6	5.78	38.9	0.5000	4.960766e-1
STD_1ppb	Standard	1.82e5	3.362e6	5.78	45.6	1.0000	8.322536e-1
STD_5ppb	Standard	4.53e5	3.171e6	5.80	58.7	5.0000	5.135044e0
STD_10ppb	Standard	7.30e5	2.960e6	5.78	66.6	10.0000	1.017530e1
STD_50ppb	Standard	3.16e6	2.969e6	5.78	62.7	50.0000	4.992074e1
STD_100ppb	Standard	5.58e6	2.654e6	5.79	67.8	100.0000	1.002809e2
STD_200ppb	Standard	1.10e7	2.561e6	5.79	50.6	200.0000	2.074344e2
STD_500ppb	Standard	2.10e7	1.889e6	5.78	46.5	500.0000	5.375318e2
V2.0_MW_RQC1_20240724	Quality Control	N/A	4.525e5	N/A	N/A	0.0000	N/A
Blank	Unknown	N/A	1.025e3	N/A	N/A	N/A	N/A
V3.0_MWMS_20240725_1	Unknown	5.59e5	3.589e6	5.76	59.0	N/A	5.762447e0
MWXS243064a_R1	Quality Control	N/A	1.315e6	N/A	N/A	0.0000	N/A
MWXS243064a_R2	Quality Control	N/A	1.322e6	N/A	N/A	0.0000	N/A
MWXS243064a_R3	Quality Control	N/A	1.295e6	N/A	N/A	0.0000	N/A
T24186682b_a	Unknown	N/A	1.023e6	N/A	N/A	N/A	N/A
T24186682b_b	Unknown	N/A	1.021e6	N/A	N/A	N/A	N/A
T24186682b_c	Unknown	N/A	1.137e6	N/A	N/A	N/A	N/A
T24186683b_a	Unknown	N/A	1.473e6	N/A	N/A	N/A	N/A
T24186683b_b	Unknown	N/A	1.588e6	N/A	N/A	N/A	N/A
T24186683b_c	Unknown	N/A	1.587e6	N/A	N/A	N/A	N/A
T24186684b_a	Unknown	N/A	1.342e6	N/A	N/A	N/A	N/A
T24186684b_b	Unknown	N/A	1.508e6	N/A	N/A	N/A	N/A
T24186684b_c	Unknown	N/A	1.578e6	N/A	N/A	N/A	N/A

Compound name: IBA
Regression Equation: y = 0.02060 x + 0.03694 (r = 0.99751) (weighting: 1 / x^2)

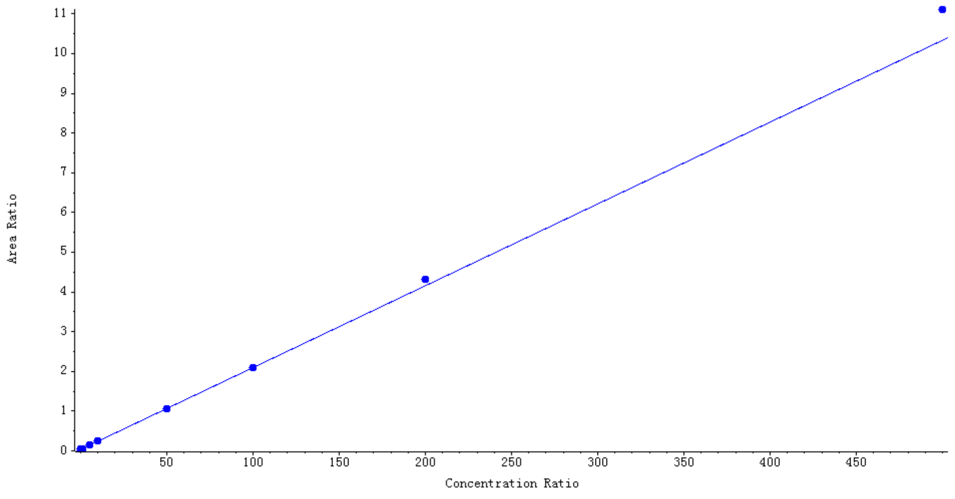

Peak Review

Blank
IBA AREA:N/A S/N:N/A

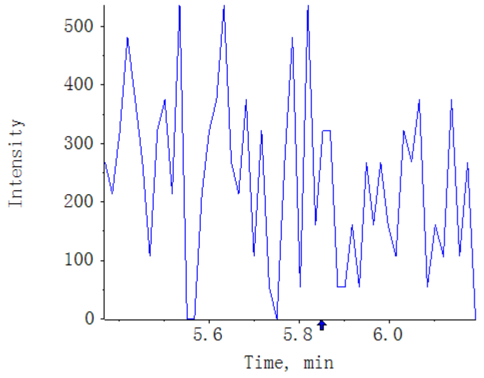

V3.0_MWMS_20240725_1
IBA AREA:5.59e5 S/N:59.0

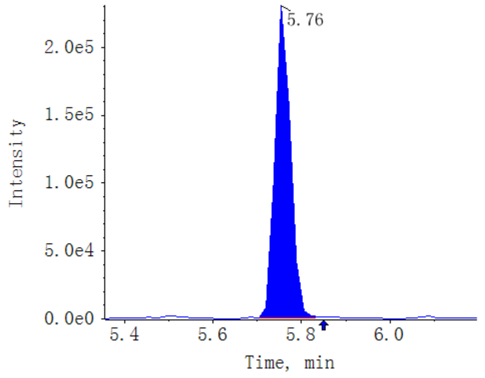

T24186682b_a
IBA AREA:N/A S/N:N/A

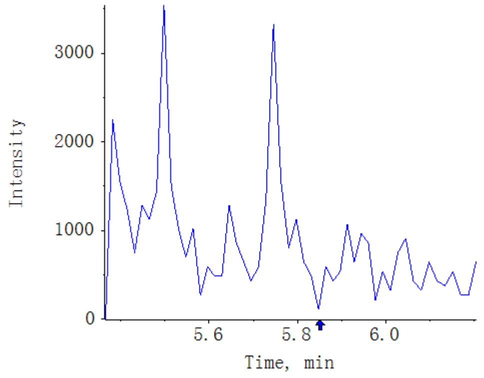

T24186682b_b
IBA AREA:N/A S/N:N/A

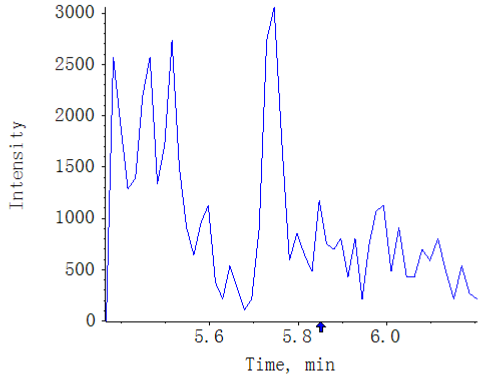

T24186682b_c
IBA AREA:N/A S/N:N/A

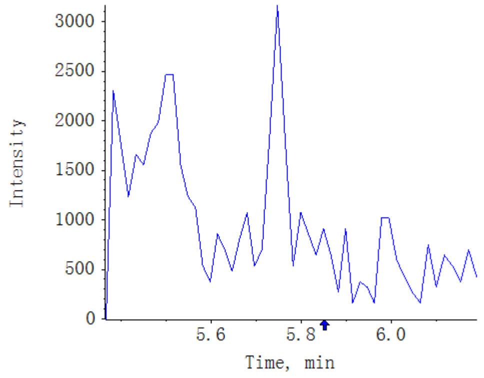

T24186683b_a
IBA AREA:N/A S/N:N/A

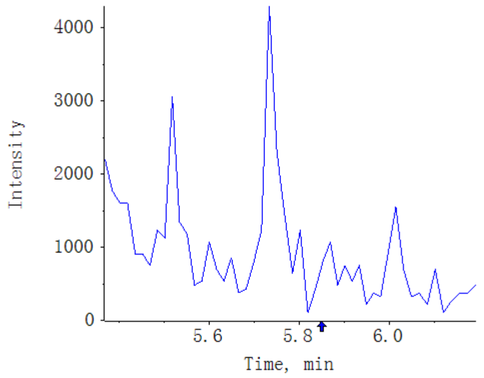

T24186683b_b
IBA AREA:N/A S/N:N/A

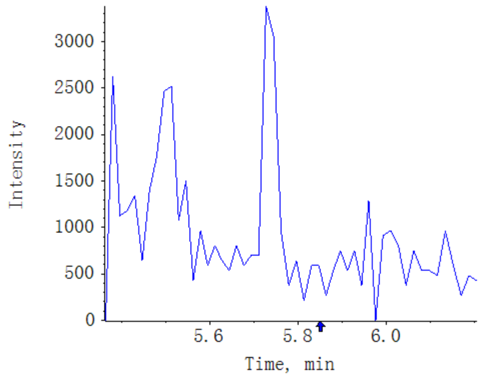

T24186683b_c
IBA AREA:N/A S/N:N/A

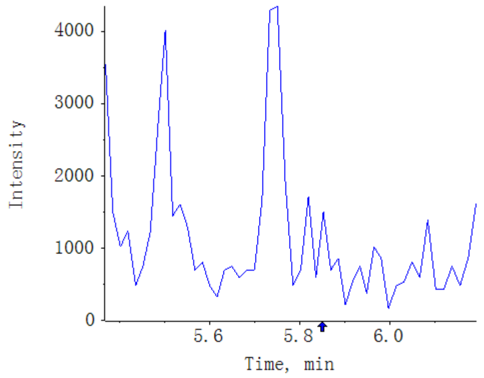

T24186684b_a
IBA AREA:N/A S/N:N/A

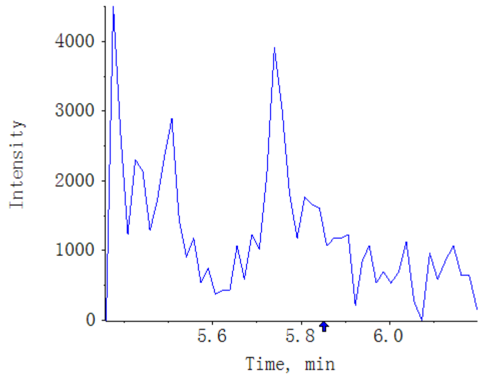

T24186684b_b
IBA AREA:N/A S/N:N/A

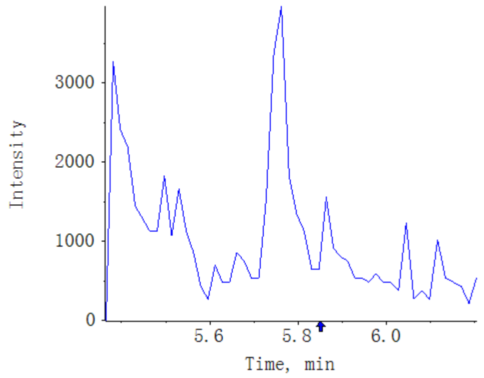

T24186684b_c
IBA AREA:N/A S/N:N/A

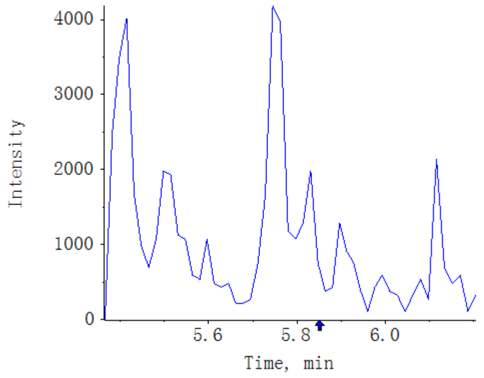

Result Table	MWXS-24-3064-a_9_WH6500-17_A20-3_V6.0_WSS_20240730	Algorithm Used	MQ4
Acquisition Method	ACC-PHs_V6.0_WH6500-17_CMY_20240521.dam	Instrument Name	Triple Quad 6500+ Low Mass
Project	N/A	Analytes QTY	109:17

Compound name: ICAld (146.1 / 91.0)

Sample Name	Sample Type	Area (cps)	Is Area (cps)	RT (min)	S/N	Target Conc	Calculated Conc.()
STD_0.01ppb	Standard	1.48e4	4.929e6	4.90	11.9	0.0100	1.018277e-2
STD_0.05ppb	Standard	2.27e4	5.184e6	4.90	20.5	0.0500	4.173434e-2
STD_0.1ppb	Standard	3.81e4	5.085e6	4.92	28.7	0.1000	1.128997e-1
STD_0.5ppb	Standard	1.28e5	4.743e6	4.91	51.1	0.5000	5.580967e-1
STD_1ppb	Standard	2.05e5	4.719e6	4.91	43.1	1.0000	9.353638e-1
STD_5ppb	Standard	1.12e6	4.577e6	4.92	60.4	5.0000	5.537270e0
STD_10ppb	Standard	1.96e6	4.478e6	4.91	52.7	10.0000	9.967999e0
STD_50ppb	Standard	9.43e6	4.148e6	4.90	41.5	50.0000	5.187290e1
STD_100ppb	Standard	1.70e7	4.035e6	4.91	41.9	100.0000	9.626451e1
STD_200ppb	Standard	3.33e7	4.409e6	4.91	45.5	200.0000	1.724250e2
STD_500ppb	Standard	N/A	5.079e6	N/A	N/A	500.0000	N/A
V2.0_MW_RQC1_20240724	Quality Control	5.59e5	1.597e6	4.89	44.4	0.0000	7.941173e0
Blank	Unknown	N/A	1.281e3	N/A	N/A	N/A	N/A
V3.0_MWMS_20240725_1	Unknown	1.17e6	4.002e6	4.89	41.7	N/A	6.625685e0
MWXS243064a_R1	Quality Control	2.98e4	2.505e6	4.88	8.4	0.0000	2.138421e-1
MWXS243064a_R2	Quality Control	3.07e4	2.649e6	4.88	9.5	0.0000	2.063845e-1
MWXS243064a_R3	Quality Control	2.90e4	2.604e6	4.87	8.4	0.0000	1.962235e-1
T24186682b_a	Unknown	2.48e4	2.839e6	4.88	7.4	N/A	1.411998e-1
T24186682b_b	Unknown	3.16e4	2.776e6	4.88	7.4	N/A	2.018256e-1
T24186682b_c	Unknown	2.65e4	2.710e6	4.88	9.6	N/A	1.647998e-1
T24186683b_a	Unknown	4.68e4	2.643e6	4.88	10.8	N/A	3.467694e-1
T24186683b_b	Unknown	3.55e4	2.532e6	4.88	11.2	N/A	2.620418e-1
T24186683b_c	Unknown	4.74e4	2.706e6	4.88	11.2	N/A	3.418842e-1
T24186684b_a	Unknown	4.28e4	2.478e6	4.88	6.5	N/A	3.366170e-1
T24186684b_b	Unknown	3.52e4	2.573e6	4.89	6.4	N/A	2.543053e-1
T24186684b_c	Unknown	3.22e4	2.508e6	4.89	6.7	N/A	2.348401e-1

Compound name: ICAld
Regression Equation: y = 0.04375 x + 0.00255 (r = 0.99431) (weighting: 1 / x^2)

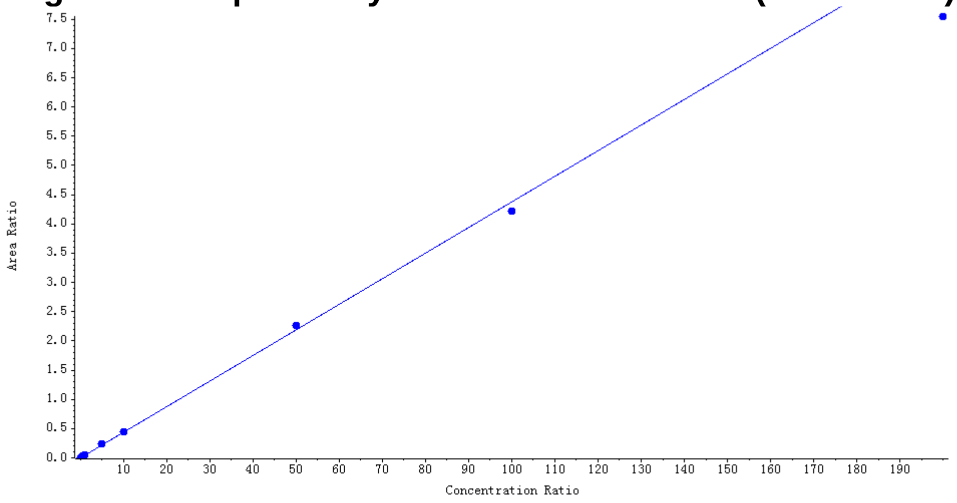

Peak Review

Blank
ICAld AREA:N/A S/N:N/A

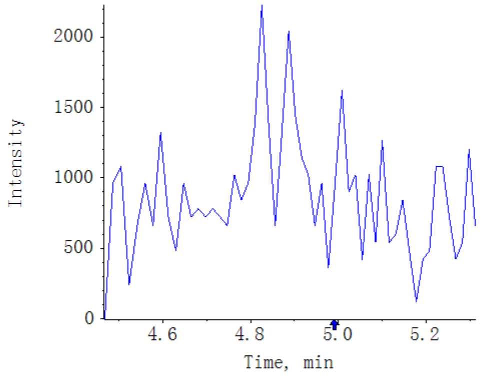

V3.0_MWMS_20240725_1
ICAld AREA:1.17e6 S/N:41.7

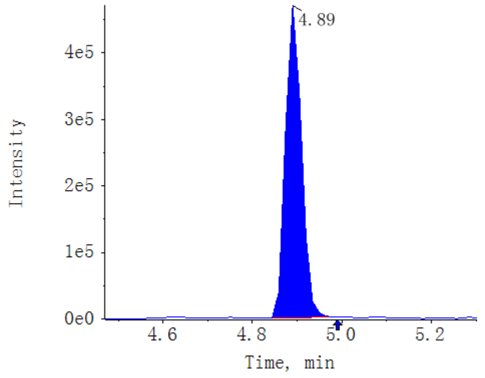

T24186682b_a
ICAld AREA:2.48e4 S/N:7.4

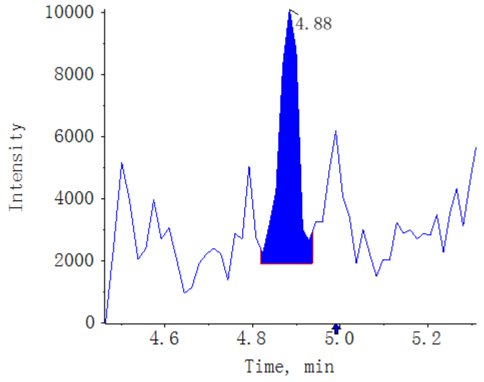

T24186682b_b
ICAld AREA:3.16e4 S/N:7.4

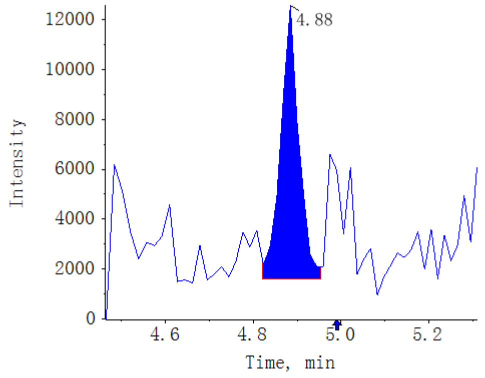

T24186682b_c
ICAld AREA:2.65e4 S/N:9.6

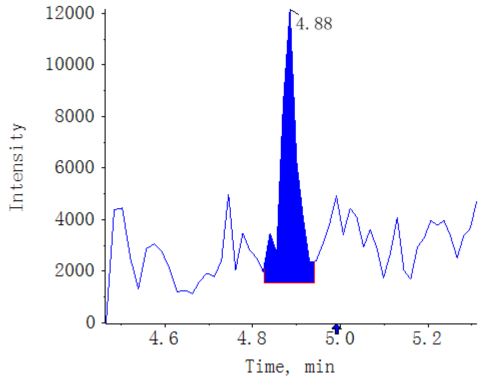

T24186683b_a
ICAld AREA:4.68e4 S/N:10.8

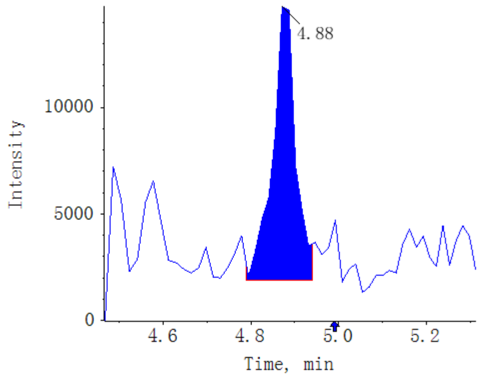

T24186683b_b
ICAld AREA:3.55e4 S/N:11.2

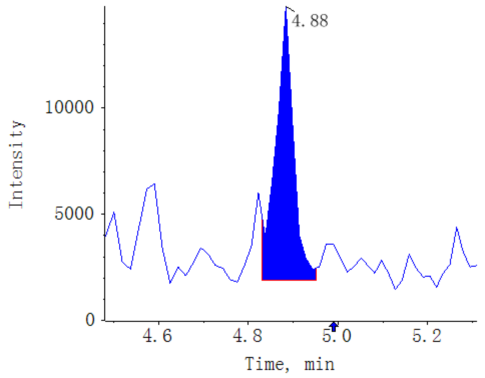

T24186683b_c
ICAld AREA:4.74e4 S/N:11.2

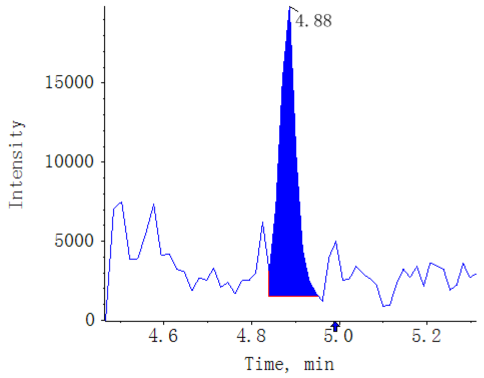

T24186684b_a
ICAld AREA:4.28e4 S/N:6.5

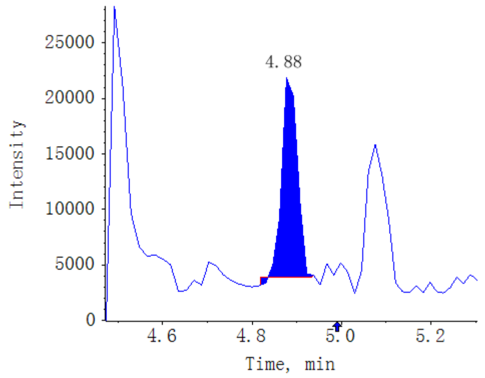

T24186684b_b
ICAld AREA:3.52e4 S/N:6.4

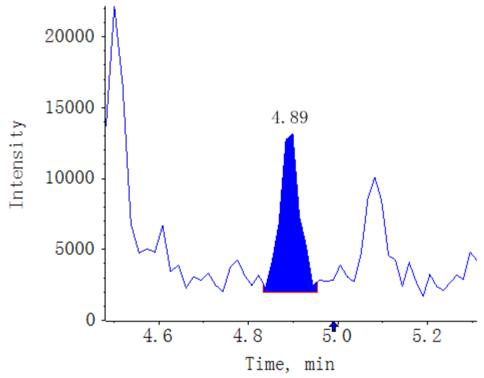

T24186684b_c
ICAld AREA:3.22e4 S/N:6.7

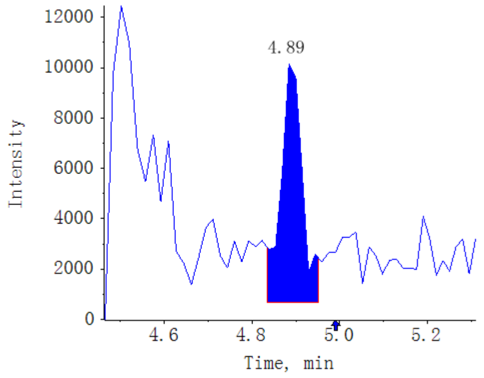

Result Table	MWXS-24-3064-a_9_WH6500-17_A20-3_V6.0_WSS_20240730	Algorithm Used	MQ4
Acquisition Method	ACC-PHs_V6.0_WH6500-17_CMY_20240521.dam	Instrument Name	Triple Quad 6500+ Low Mass
Project	N/A	Analytes QTY	109:18

Compound name: MEIAA (190.2 / 130.0)

Sample Name	Sample Type	Area (cps)	Is Area (cps)	RT (min)	S/N	Target Conc	Calculated Conc.()
STD_0.01ppb	Standard	8.89e3	1.076e7	6.08	13.9	0.0100	9.898638e-3
STD_0.05ppb	Standard	4.17e4	1.069e7	6.09	35.2	0.0500	4.997865e-2
STD_0.1ppb	Standard	8.97e4	1.076e7	6.10	58.3	0.1000	1.078790e-1
STD_0.5ppb	Standard	4.27e5	9.698e6	6.09	96.8	0.5000	5.736948e-1
STD_1ppb	Standard	6.85e5	9.537e6	6.09	73.2	1.0000	9.362956e-1
STD_5ppb	Standard	3.95e6	9.817e6	6.11	81.3	5.0000	5.249139e0
STD_10ppb	Standard	6.70e6	9.338e6	6.09	68.4	10.0000	9.362456e0
STD_50ppb	Standard	3.44e7	8.645e6	6.09	74.6	50.0000	5.185695e1
STD_100ppb	Standard	6.00e7	8.273e6	6.10	60.2	100.0000	9.459628e1
STD_200ppb	Standard	1.10e8	8.128e6	6.09	72.7	200.0000	1.757825e2
STD_500ppb	Standard	N/A	6.706e6	N/A	N/A	500.0000	N/A
V2.0_MW_RQC1_20240724	Quality Control	1.27e5	3.700e6	6.06	37.5	0.0000	4.464205e-1
Blank	Unknown	N/A	1.351e3	N/A	N/A	N/A	N/A
V3.0_MWMS_20240725_1	Unknown	3.96e6	8.473e6	6.06	90.7	N/A	6.095006e0
MWXS243064a_R1	Quality Control	1.40e4	4.541e6	6.05	12.1	0.0000	3.944896e-2
MWXS243064a_R2	Quality Control	1.41e4	4.530e6	6.06	12.2	0.0000	3.970851e-2
MWXS243064a_R3	Quality Control	1.66e4	4.486e6	6.04	11.0	0.0000	4.743709e-2
T24186682b_a	Unknown	1.45e4	3.979e6	6.05	9.1	N/A	4.673395e-2
T24186682b_b	Unknown	1.35e4	3.876e6	6.05	9.6	N/A	4.464006e-2
T24186682b_c	Unknown	1.62e4	4.176e6	6.06	9.2	N/A	4.970687e-2
T24186683b_a	Unknown	2.04e4	5.046e6	6.06	8.8	N/A	5.181160e-2
T24186683b_b	Unknown	1.98e4	5.488e6	6.05	13.0	N/A	4.619457e-2
T24186683b_c	Unknown	2.44e4	5.291e6	6.05	19.0	N/A	5.934511e-2
T24186684b_a	Unknown	3.75e4	4.710e6	6.05	18.2	N/A	1.029314e-1
T24186684b_b	Unknown	2.72e4	4.327e6	6.06	17.9	N/A	8.122042e-2
T24186684b_c	Unknown	2.28e4	4.214e6	6.07	9.9	N/A	6.966405e-2

Compound name: MEIAA
Regression Equation: y = 0.07666 x + 6.79737e-5 (r = 0.99655) (weighting: 1 / x^2)

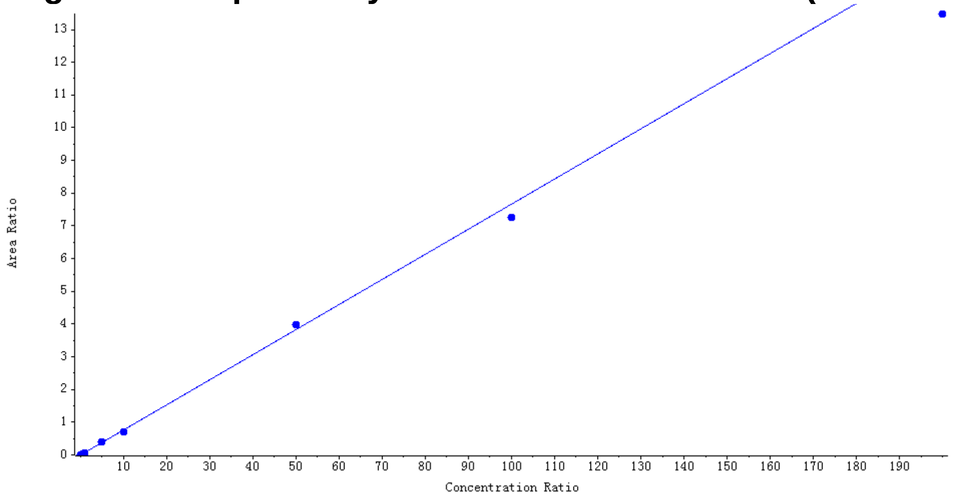

Peak Review

Blank
MEIAA AREA:N/A S/N:N/A

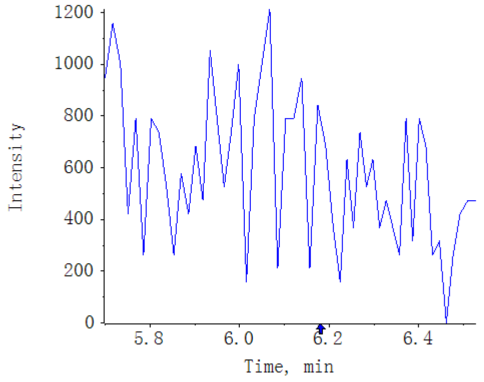

V3.0_MWMS_20240725_1
MEIAA AREA:3.96e6 S/N:90.7

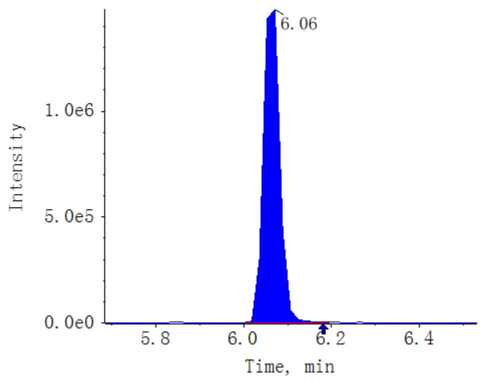

T24186682b_a
MEIAA AREA:1.45e4 S/N:9.1

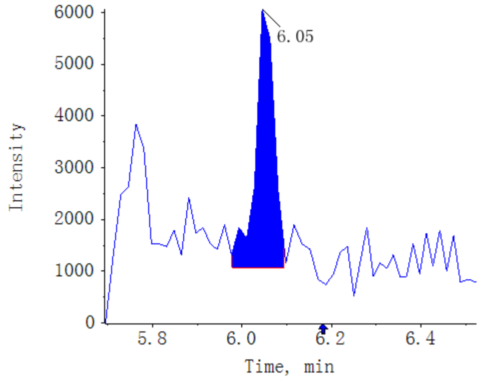

T24186682b_b
MEIAA AREA:1.35e4 S/N:9.6

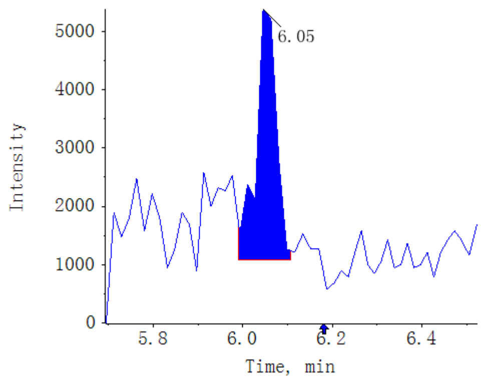

T24186682b_c
MEIAA AREA:1.62e4 S/N:9.2

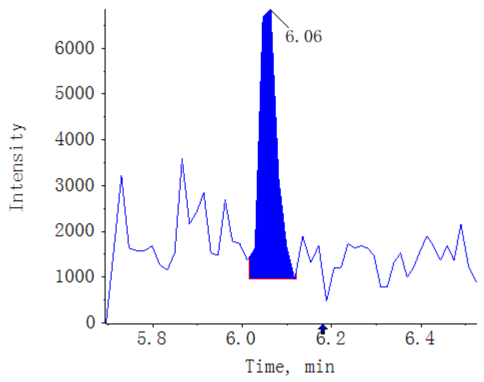

T24186683b_a
MEIAA AREA:2.04e4 S/N:8.8

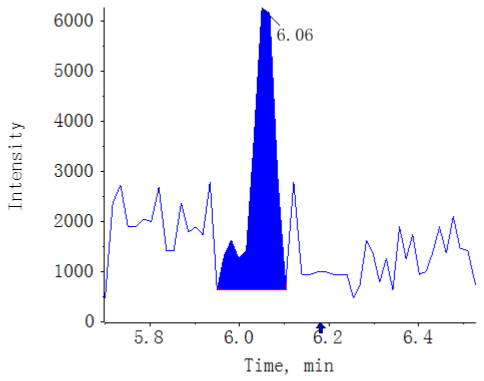

T24186683b_b
MEIAA AREA:1.98e4 S/N:13.0

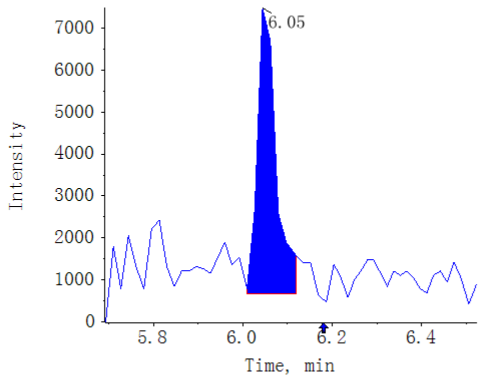

T24186683b_c
MEIAA AREA:2.44e4 S/N:19.0

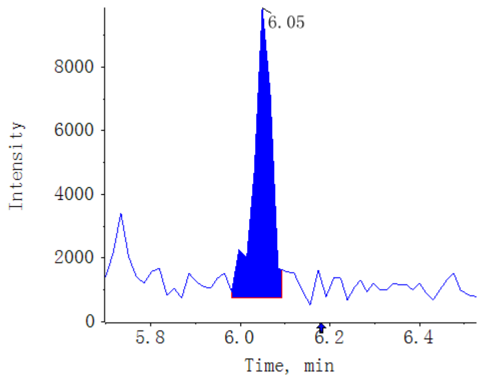

T24186684b_a
MEIAA AREA:3.75e4 S/N:18.2

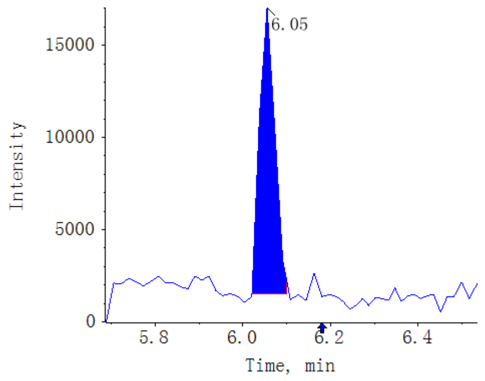

T24186684b_b
MEIAA AREA:2.72e4 S/N:17.9

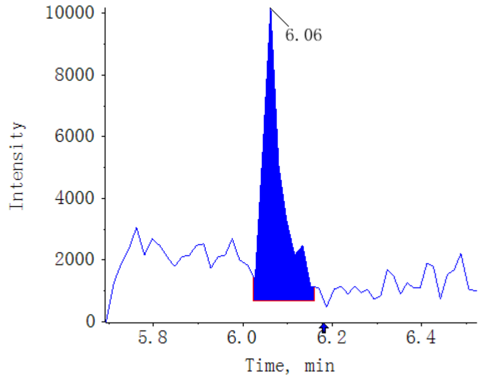

T24186684b_c
MEIAA AREA:2.28e4 S/N:9.9

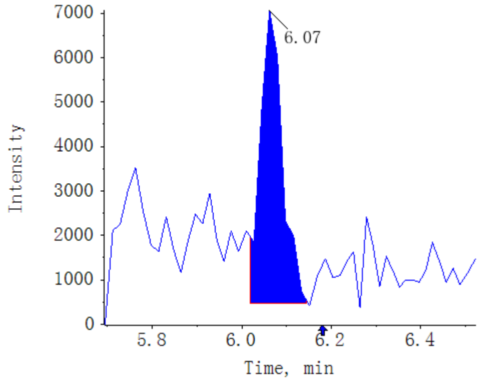

Result Table	MWXS-24-3064-a_9_WH6500-17_A20-3_V6.0_WSS_20240730	Algorithm Used	MQ4
Acquisition Method	ACC-PHs_V6.0_WH6500-17_CMY_20240521.dam	Instrument Name	Triple Quad 6500+ Low Mass
Project	N/A	Analytes QTY	109:19

Compound name: IPA (190.0 / 130.0)

Sample Name	Sample Type	Area (cps)	Is Area (cps)	RT (min)	S/N	Target Conc	Calculated Conc.()
STD_0.01ppb	Standard	N/A	3.231e6	N/A	N/A	0.0100	N/A
STD_0.05ppb	Standard	9.71e3	3.439e6	5.46	11.0	0.0500	5.520545e-2
STD_0.1ppb	Standard	1.31e4	3.359e6	5.48	17.2	0.1000	8.191475e-2
STD_0.5ppb	Standard	6.01e4	3.169e6	5.47	31.9	0.5000	4.579395e-1
STD_1ppb	Standard	1.23e5	3.362e6	5.47	49.7	1.0000	8.968367e-1
STD_5ppb	Standard	6.33e5	3.171e6	5.48	66.5	5.0000	4.971204e0
STD_10ppb	Standard	1.14e6	2.960e6	5.47	66.3	10.0000	9.591965e0
STD_50ppb	Standard	6.34e6	2.969e6	5.46	61.7	50.0000	5.327534e1
STD_100ppb	Standard	1.16e7	2.654e6	5.48	73.6	100.0000	1.089102e2
STD_200ppb	Standard	2.37e7	2.561e6	5.47	66.7	200.0000	2.311964e2
STD_500ppb	Standard	N/A	1.889e6	N/A	N/A	500.0000	N/A
V2.0_MW_RQC1_20240724	Quality Control	N/A	4.525e5	N/A	N/A	0.0000	N/A
Blank	Unknown	N/A	1.025e3	N/A	N/A	N/A	N/A
V3.0_MWMS_20240725_1	Unknown	7.73e5	3.589e6	5.44	64.9	N/A	5.362418e0
MWXS243064a_R1	Quality Control	N/A	1.315e6	N/A	N/A	0.0000	N/A
MWXS243064a_R2	Quality Control	N/A	1.322e6	N/A	N/A	0.0000	N/A
MWXS243064a_R3	Quality Control	N/A	1.295e6	N/A	N/A	0.0000	N/A
T24186682b_a	Unknown	N/A	1.023e6	N/A	N/A	N/A	N/A
T24186682b_b	Unknown	N/A	1.021e6	N/A	N/A	N/A	N/A
T24186682b_c	Unknown	N/A	1.137e6	N/A	N/A	N/A	N/A
T24186683b_a	Unknown	N/A	1.473e6	N/A	N/A	N/A	N/A
T24186683b_b	Unknown	N/A	1.588e6	N/A	N/A	N/A	N/A
T24186683b_c	Unknown	N/A	1.587e6	N/A	N/A	N/A	N/A
T24186684b_a	Unknown	N/A	1.342e6	N/A	N/A	N/A	N/A
T24186684b_b	Unknown	N/A	1.508e6	N/A	N/A	N/A	N/A
T24186684b_c	Unknown	N/A	1.578e6	N/A	N/A	N/A	N/A

Compound name: IPA
Regression Equation: y = 0.04006 x + 6.10834e-4 (r = 0.99276) (weighting: 1 / x^2)

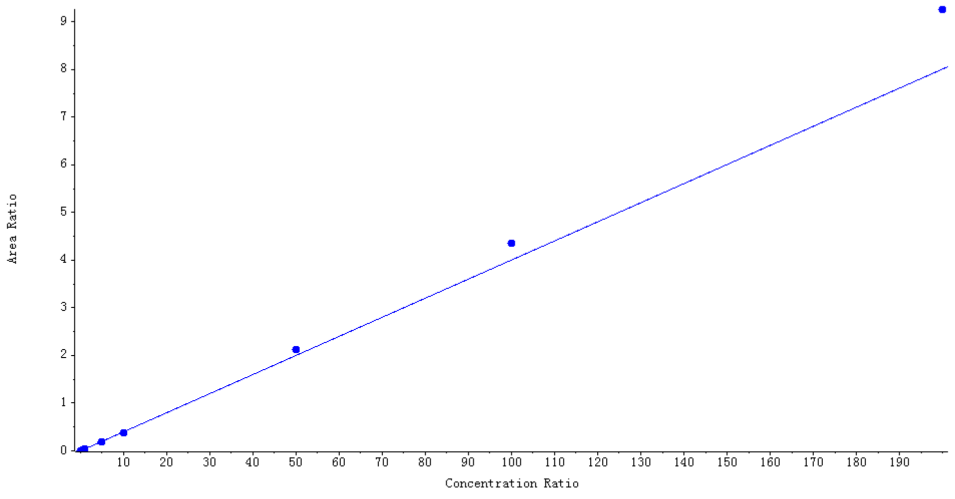

Peak Review

Blank

IPA AREA:N/A S/N:N/A

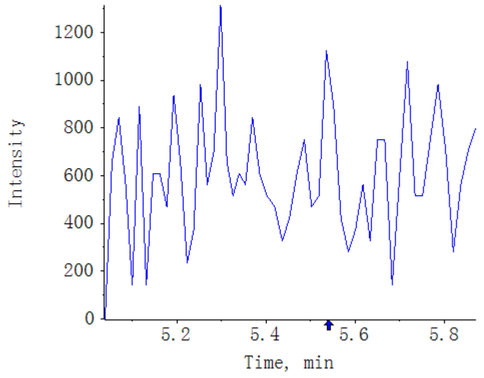

V3.0_MWMS_20240725_1

IPA AREA:7.73e5 S/N:64.9

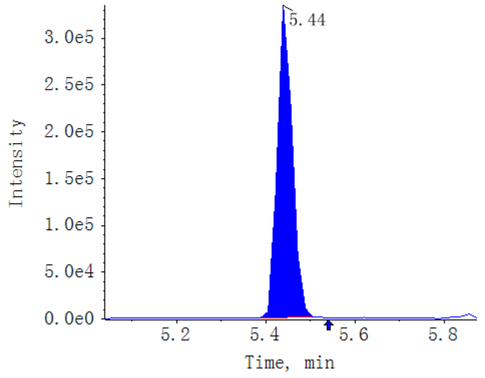

T24186682b_a

IPA AREA:N/A S/N:N/A

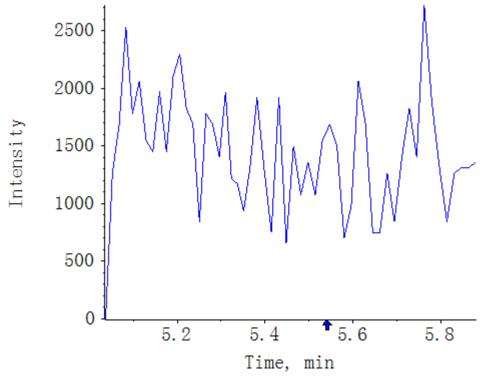

T24186682b_b

IPA AREA:N/A S/N:N/A

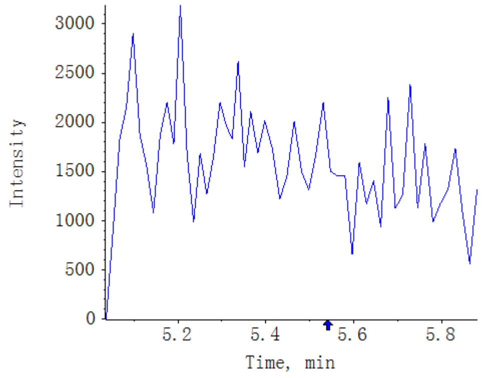

T24186682b_c

IPA AREA:N/A S/N:N/A

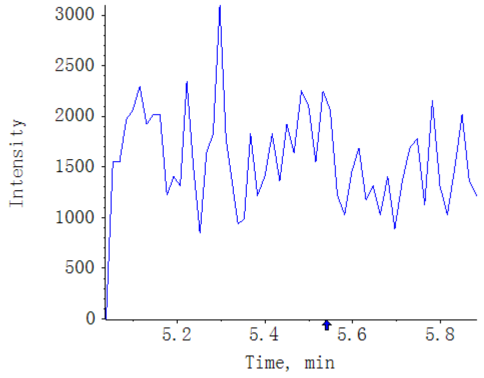

T24186683b_a

IPA AREA:N/A S/N:N/A

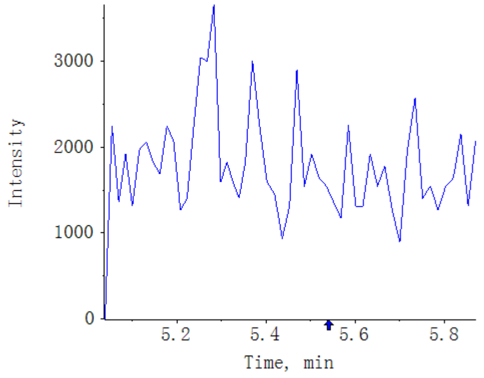

T24186683b_b

IPA AREA:N/A S/N:N/A

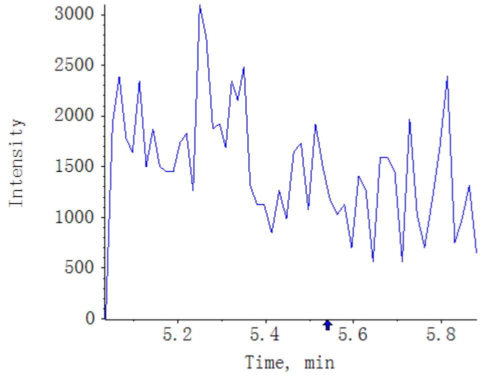

T24186683b_c

IPA AREA:N/A S/N:N/A

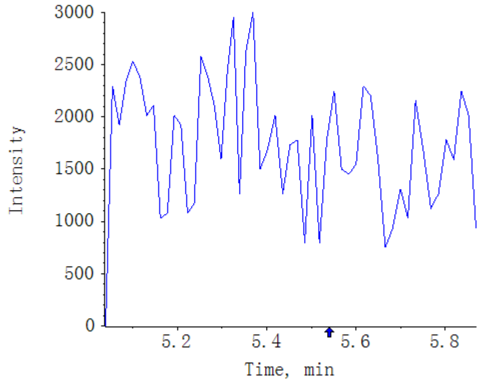

T24186684b_a

IPA AREA:N/A S/N:N/A

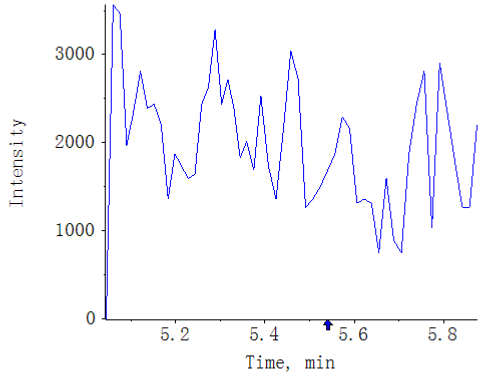

T24186684b_b

IPA AREA:N/A S/N:N/A

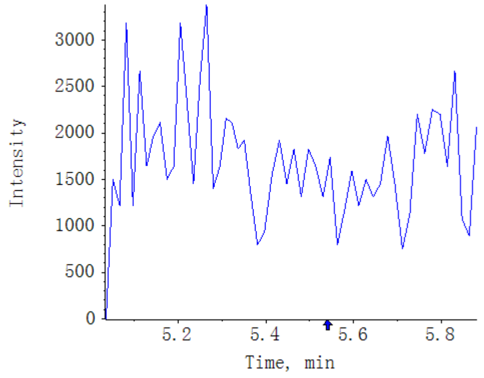

T24186684b_c

IPA AREA:N/A S/N:N/A

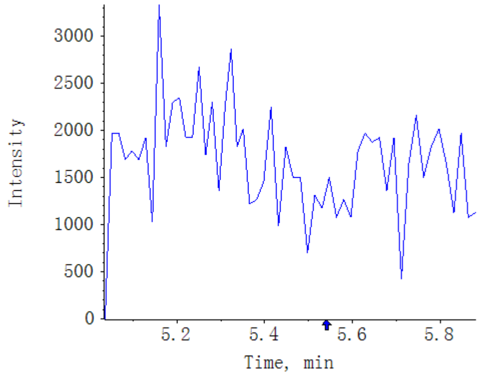

Result Table	MWXS-24-3064-a_9_WH6500-17_A20-3_V6.0_WSS_20240730	Algorithm Used	MQ4
Acquisition Method	ACC-PHs_V6.0_WH6500-17_CMY_20240521.dam	Instrument Name	Triple Quad 6500+ Low Mass
Project	N/A	Analytes QTY	109:20

Compound name: Indole (118.1 / 91.0)

Sample Name	Sample Type	Area (cps)	Is Area (cps)	RT (min)	S/N	Target Conc	Calculated Conc.()
STD_0.01ppb	Standard	N/A	1.463e7	N/A	N/A	0.0100	N/A
STD_0.05ppb	Standard	3.02e4	1.538e7	3.31	25.0	0.0500	4.364042e-2
STD_0.1ppb	Standard	3.20e4	1.466e7	3.32	20.6	0.1000	1.254173e-1
STD_0.5ppb	Standard	4.69e4	1.438e7	3.30	16.6	0.5000	5.257424e-1
STD_1ppb	Standard	6.25e4	1.446e7	3.28	12.0	1.0000	9.184229e-1
STD_5ppb	Standard	2.07e5	1.392e7	3.25	33.2	5.0000	4.821435e0
STD_10ppb	Standard	3.41e5	1.335e7	3.24	34.3	10.0000	8.782581e0
STD_50ppb	Standard	1.88e6	1.326e7	3.24	33.3	50.0000	5.185477e1
STD_100ppb	Standard	3.41e6	1.370e7	3.24	35.3	100.0000	9.161559e1
STD_200ppb	Standard	6.96e6	1.327e7	3.24	35.8	200.0000	1.937970e2
STD_500ppb	Standard	1.51e7	9.829e6	3.23	30.7	500.0000	5.691648e2
V2.0_MW_RQC1_20240724	Quality Control	6.08e6	2.323e6	3.30	514.1	0.0000	9.709760e2
Blank	Unknown	N/A	3.627e3	N/A	N/A	N/A	N/A
V3.0_MWMS_20240725_1	Unknown	3.46e5	1.365e7	3.30	78.9	N/A	8.724631e0
MWXS243064a_R1	Quality Control	2.96e6	1.937e6	3.28	464.4	0.0000	5.659379e2
MWXS243064a_R2	Quality Control	2.92e6	2.063e6	3.29	453.7	0.0000	5.249633e2
MWXS243064a_R3	Quality Control	2.94e6	2.038e6	3.27	538.5	0.0000	5.339768e2
T24186682b_a	Unknown	5.84e5	1.307e6	3.29	150.0	N/A	1.648997e2
T24186682b_b	Unknown	5.65e5	1.262e6	3.29	148.2	N/A	1.655429e2
T24186682b_c	Unknown	6.37e5	1.267e6	3.29	168.3	N/A	1.860185e2
T24186683b_a	Unknown	N/A	3.941e6	N/A	N/A	N/A	N/A
T24186683b_b	Unknown	N/A	4.032e6	N/A	N/A	N/A	N/A
T24186683b_c	Unknown	N/A	3.923e6	N/A	N/A	N/A	N/A
T24186684b_a	Unknown	8.13e6	2.919e6	3.29	677.3	N/A	1.032203e3
T24186684b_b	Unknown	6.62e6	2.979e6	3.29	639.3	N/A	8.234107e2
T24186684b_c	Unknown	8.32e6	3.050e6	3.29	915.0	N/A	1.011310e3

Compound name: Indole
Regression Equation: y = 0.00270 x + 0.00185 (r = 0.99148) (weighting: 1 / x^2)

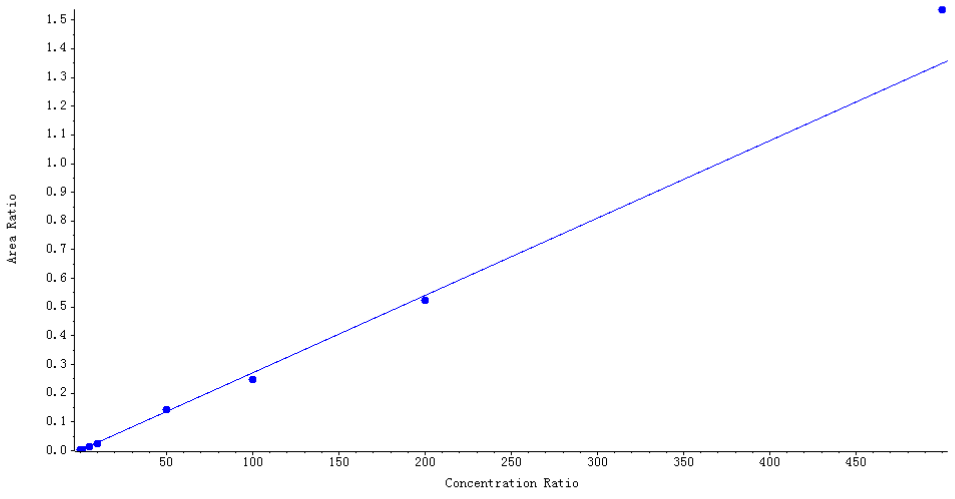

Peak Review

Blank
Indole AREA:N/A S/N:N/A

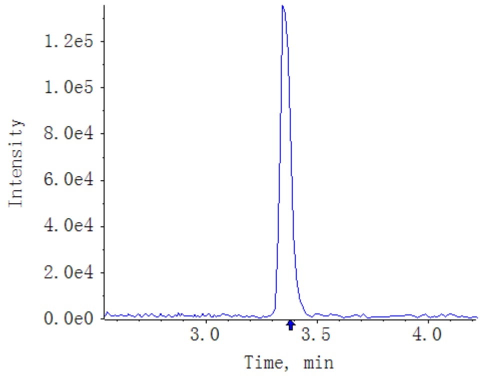

V3.0_MWMS_20240725_1
Indole AREA:3.46e5 S/N:78.9

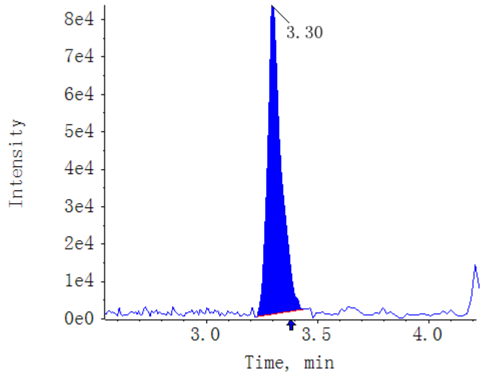

T24186682b_a
Indole AREA:5.84e5 S/N:150.0

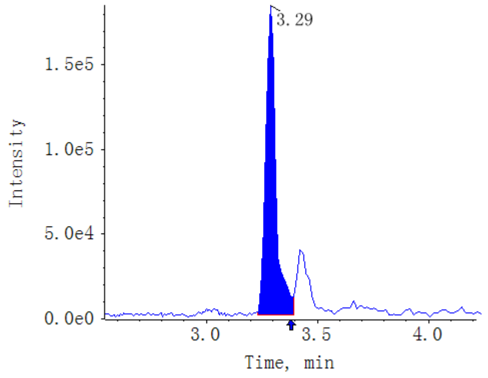

T24186682b_b
Indole AREA:5.65e5 S/N:148.2

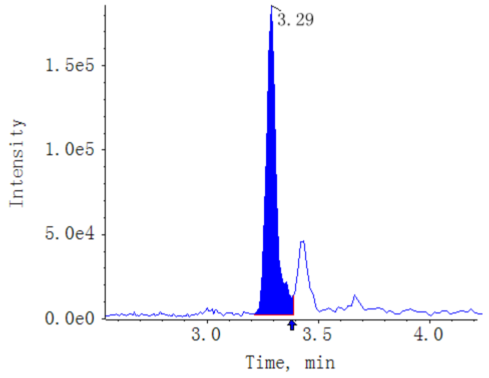

T24186682b_c
Indole AREA:6.37e5 S/N:168.3

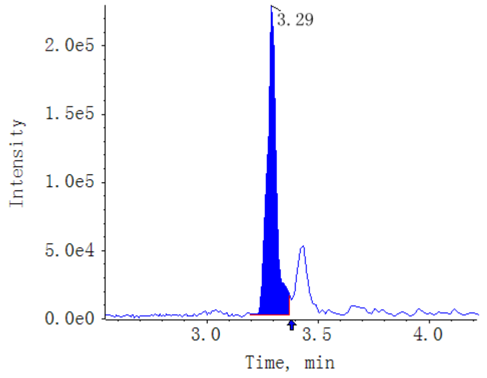

T24186683b_a
Indole AREA:N/A S/N:N/A

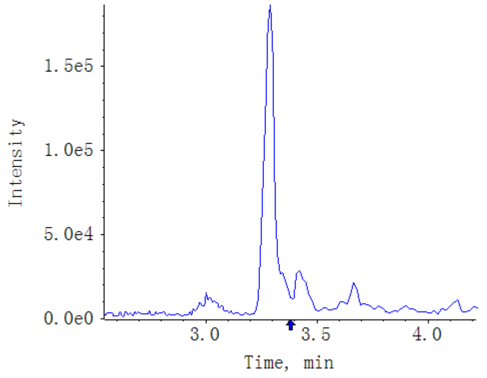

T24186683b_b
Indole AREA:N/A S/N:N/A

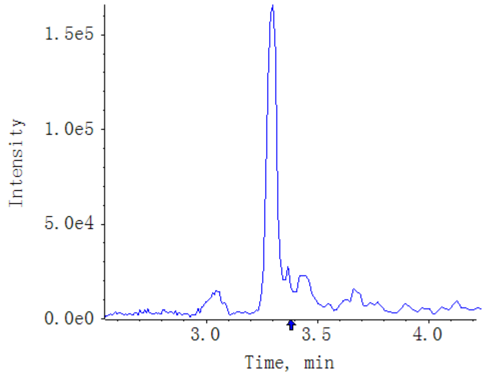

T24186683b_c
Indole AREA:N/A S/N:N/A

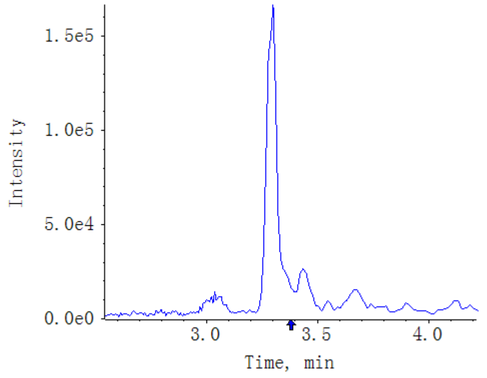

T24186684b_a
Indole AREA:8.13e6 S/N:677.3

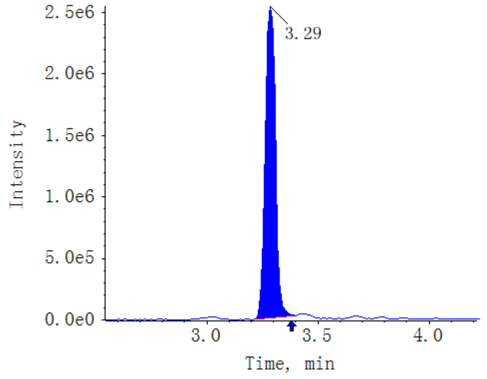

T24186684b_b
Indole AREA:6.62e6 S/N:639.3

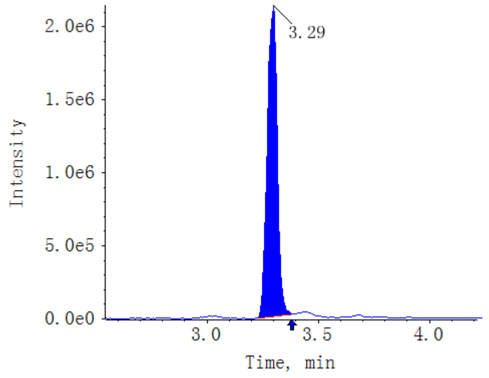

T24186684b_c
Indole AREA:8.32e6 S/N:915.0

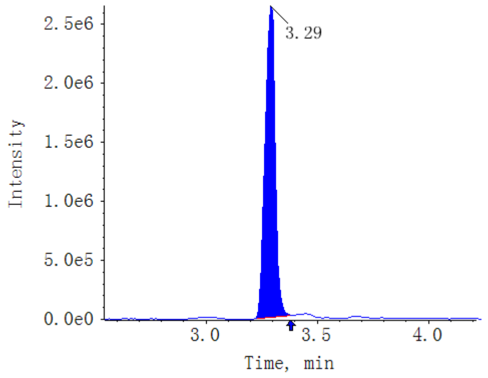

Result Table	MWXS-24-3064-a_9_WH6500-17_A20-3_V6.0_WSS_20240730	Algorithm Used	MQ4
Acquisition Method	ACC-PHs_V6.0_WH6500-17_CMY_20240521.dam	Instrument Name	Triple Quad 6500+ Low Mass
Project	N/A	Analytes QTY	109:21

Compound name: IAA-Glc+Na (360.1 / 198.2)

Sample Name	Sample Type	Area (cps)	Is Area (cps)	RT (min)	S/N	Target Conc	Calculated Conc.()
STD_0.01ppb	Standard	N/A	1.463e7	N/A	N/A	0.2000	N/A
STD_0.05ppb	Standard	1.27e4	1.538e7	4.29	15.5	1.0000	9.646164e-1
STD_0.1ppb	Standard	1.88e4	1.466e7	4.31	19.9	2.0000	2.057715e0
STD_0.5ppb	Standard	7.57e4	1.438e7	4.29	30.6	10.0000	1.156568e1
STD_1ppb	Standard	1.38e5	1.446e7	4.30	45.1	20.0000	2.178478e1
STD_5ppb	Standard	6.40e5	1.392e7	4.31	36.1	100.0000	1.087228e2
STD_10ppb	Standard	1.18e6	1.335e7	4.29	48.0	200.0000	2.096814e2
STD_50ppb	Standard	4.55e6	1.326e7	4.29	48.8	1000.0000	8.183312e2
STD_100ppb	Standard	9.26e6	1.370e7	4.31	65.8	2000.0000	1.613505e3
STD_200ppb	Standard	N/A	1.327e7	N/A	N/A	4000.0000	N/A
STD_500ppb	Standard	N/A	9.829e6	N/A	N/A	10000.0000	N/A
V2.0_MW_RQC1_20240724	Quality Control	2.75e4	2.323e6	4.23	5.9	0.0000	2.724270e1
Blank	Unknown	N/A	3.627e3	N/A	N/A	N/A	N/A
V3.0_MWMS_20240725_1	Unknown	3.74e6	1.365e7	4.29	27.5	N/A	6.535235e2
MWXS243064a_R1	Quality Control	N/A	1.937e6	N/A	N/A	0.0000	N/A
MWXS243064a_R2	Quality Control	N/A	2.063e6	N/A	N/A	0.0000	N/A
MWXS243064a_R3	Quality Control	N/A	2.038e6	N/A	N/A	0.0000	N/A
T24186682b_a	Unknown	N/A	1.307e6	N/A	N/A	N/A	N/A
T24186682b_b	Unknown	N/A	1.262e6	N/A	N/A	N/A	N/A
T24186682b_c	Unknown	N/A	1.267e6	N/A	N/A	N/A	N/A
T24186683b_a	Unknown	N/A	3.941e6	N/A	N/A	N/A	N/A
T24186683b_b	Unknown	N/A	4.032e6	N/A	N/A	N/A	N/A
T24186683b_c	Unknown	N/A	3.923e6	N/A	N/A	N/A	N/A
T24186684b_a	Unknown	N/A	2.919e6	N/A	N/A	N/A	N/A
T24186684b_b	Unknown	N/A	2.979e6	N/A	N/A	N/A	N/A
T24186684b_c	Unknown	N/A	3.050e6	N/A	N/A	N/A	N/A

Compound name: IAA-Glc+Na
Regression Equation: y = 4.18815e-4 x + 4.21164e-4 (r = 0.99024) (weighting: 1 / x^2)

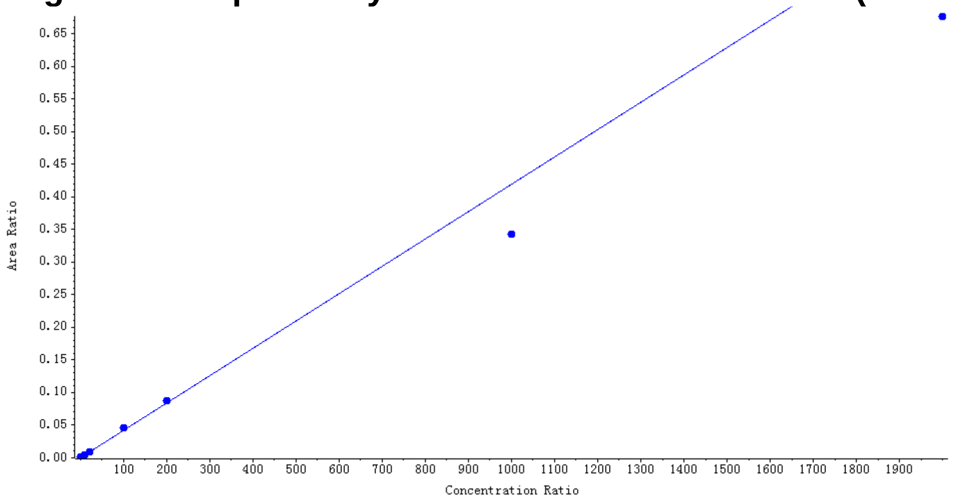

Peak Review

Blank

IAA-Glc+Na AREA:N/A S/N:N/A

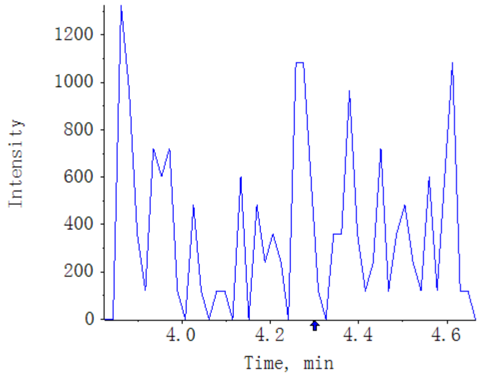

V3.0_MWMS_20240725_1

IAA-Glc+Na AREA:3.74e6 S/N:27.5

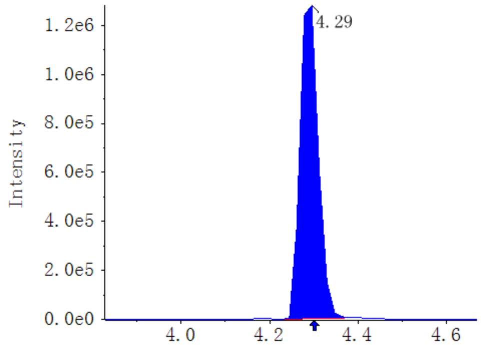

T24186682b_a

IAA-Glc+Na AREA:N/A S/N:N/A

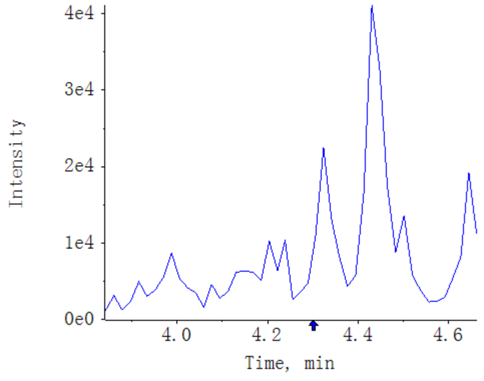

T24186682b_b

IAA-Glc+Na AREA:N/A S/N:N/A

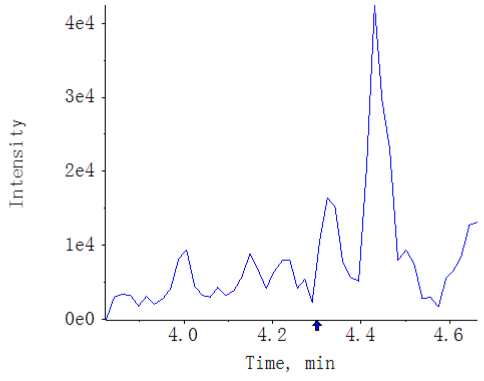

T24186682b_c

IAA-Glc+Na AREA:N/A S/N:N/A

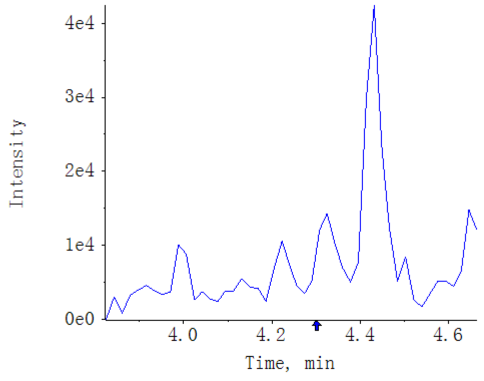

T24186683b_a

IAA-Glc+Na AREA:N/A S/N:N/A

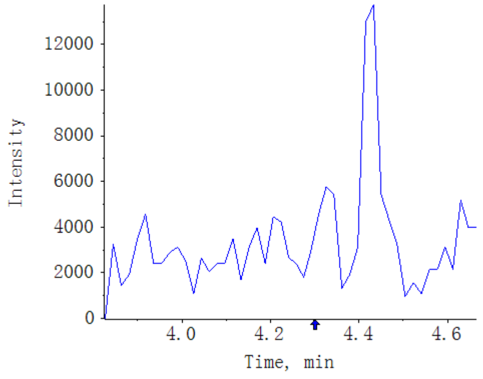

T24186683b_b

IAA-Glc+Na AREA:N/A S/N:N/A

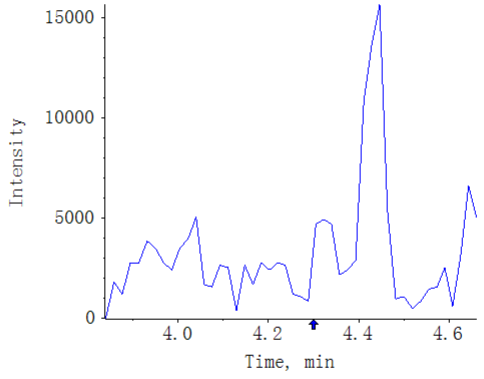

T24186683b_c

IAA-Glc+Na AREA:N/A S/N:N/A

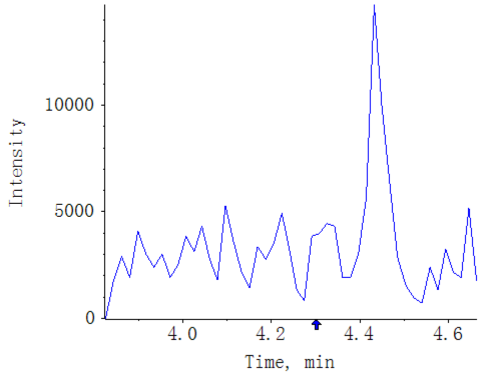

T24186684b_a

IAA-Glc+Na AREA:N/A S/N:N/A

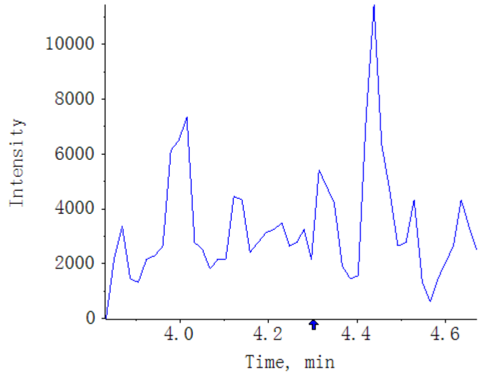

T24186684b_b

IAA-Glc+Na AREA:N/A S/N:N/A

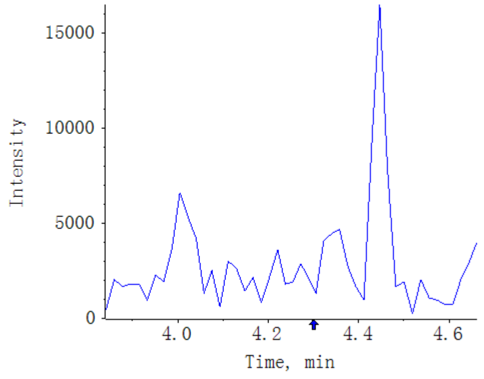

T24186684b_c

IAA-Glc+Na AREA:N/A S/N:N/A

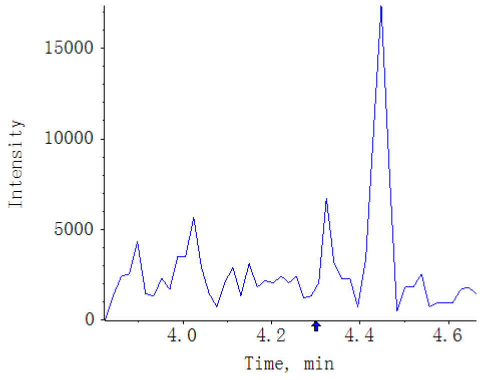

Result Table	MWXS-24-3064-a_9_WH6500-17_A20-3_V6.0_WSS_20240730	Algorithm Used	MQ4
Acquisition Method	ACC-PHs_V6.0_WH6500-17_CMY_20240521.dam	Instrument Name	Triple Quad 6500+ Low Mass
Project	N/A	Analytes QTY	109:22

Compound name: DHZR (354.0 / 222.0)

Sample Name	Sample Type	Area (cps)	Is Area (cps)	RT (min)	S/N	Target Conc	Calculated Conc.()
STD_0.01ppb	Standard	8.42e3	9.801e6	3.63	10.6	0.0100	9.712396e-3
STD_0.05ppb	Standard	5.51e4	1.037e7	3.63	46.1	0.0500	5.752027e-2
STD_0.1ppb	Standard	9.47e4	1.061e7	3.64	46.4	0.1000	9.635114e-2
STD_0.5ppb	Standard	4.53e5	8.670e6	3.63	53.8	0.5000	5.613109e-1
STD_1ppb	Standard	8.05e5	8.868e6	3.63	45.1	1.0000	9.758523e-1
STD_5ppb	Standard	4.13e6	8.482e6	3.65	70.8	5.0000	5.232764e0
STD_10ppb	Standard	7.32e6	7.694e6	3.63	71.4	10.0000	1.022228e1
STD_50ppb	Standard	3.46e7	6.996e6	3.63	66.4	50.0000	5.308781e1
STD_100ppb	Standard	5.87e7	6.577e6	3.64	61.0	100.0000	9.587445e1
STD_200ppb	Standard	1.05e8	5.831e6	3.63	37.4	200.0000	1.941356e2
STD_500ppb	Standard	1.74e8	4.950e6	3.64	37.0	500.0000	3.782049e2
V2.0_MW_RQC1_20240724	Quality Control	N/A	6.829e6	N/A	N/A	0.0000	N/A
Blank	Unknown	N/A	6.826e2	N/A	N/A	N/A	N/A
V3.0_MWMS_20240725_1	Unknown	7.96e6	1.549e7	3.64	42.3	N/A	5.517735e0
MWXS243064a_R1	Quality Control	N/A	3.825e6	N/A	N/A	0.0000	N/A
MWXS243064a_R2	Quality Control	N/A	3.773e6	N/A	N/A	0.0000	N/A
MWXS243064a_R3	Quality Control	N/A	3.826e6	N/A	N/A	0.0000	N/A
T24186682b_a	Unknown	N/A	3.711e6	N/A	N/A	N/A	N/A
T24186682b_b	Unknown	N/A	3.614e6	N/A	N/A	N/A	N/A
T24186682b_c	Unknown	N/A	3.762e6	N/A	N/A	N/A	N/A
T24186683b_a	Unknown	N/A	3.543e6	N/A	N/A	N/A	N/A
T24186683b_b	Unknown	N/A	3.969e6	N/A	N/A	N/A	N/A
T24186683b_c	Unknown	N/A	3.767e6	N/A	N/A	N/A	N/A
T24186684b_a	Unknown	N/A	3.638e6	N/A	N/A	N/A	N/A
T24186684b_b	Unknown	N/A	3.787e6	N/A	N/A	N/A	N/A
T24186684b_c	Unknown	N/A	3.698e6	N/A	N/A	N/A	N/A

Compound name: DHZR
Regression Equation: y = 0.09310 x + -4.52651e-5 (r = 0.99421) (weighting: 1 / x^2)

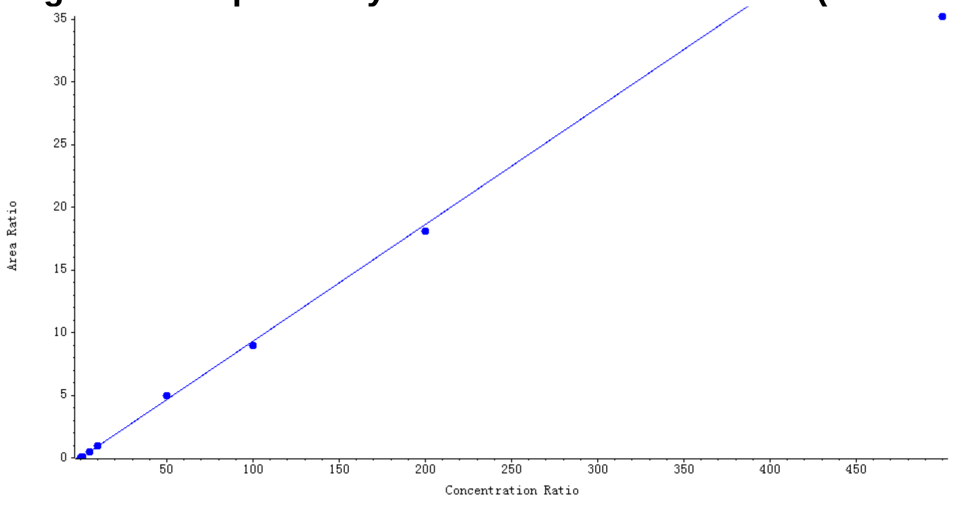

Peak Review

Blank
DHZR AREA:N/A S/N:N/A

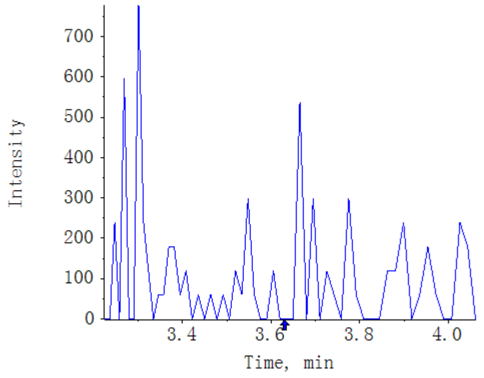

V3.0_MWMS_20240725_1
DHZR AREA:7.96e6 S/N:42.3

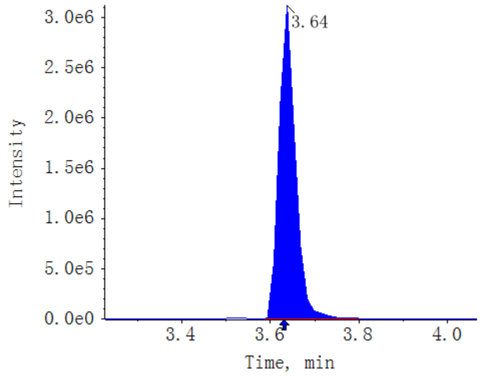

T24186682b_a
DHZR AREA:N/A S/N:N/A

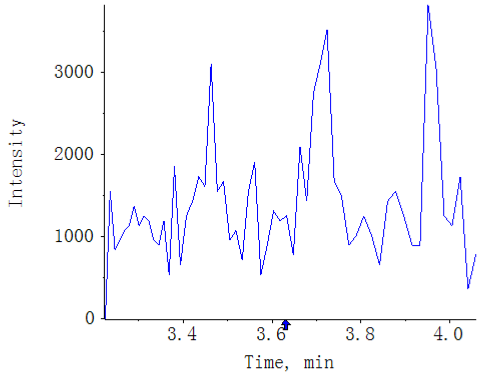

T24186682b_b
DHZR AREA:N/A S/N:N/A

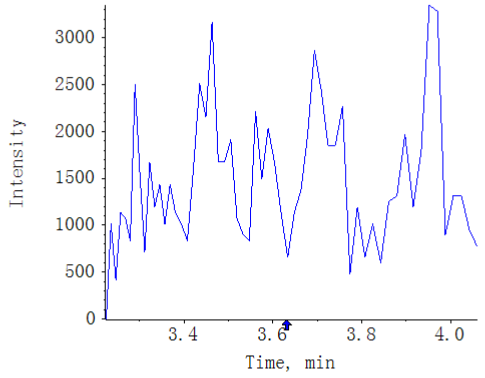

T24186682b_c
DHZR AREA:N/A S/N:N/A

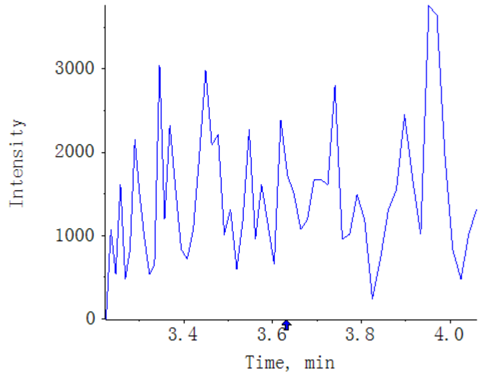

T24186683b_a
DHZR AREA:N/A S/N:N/A

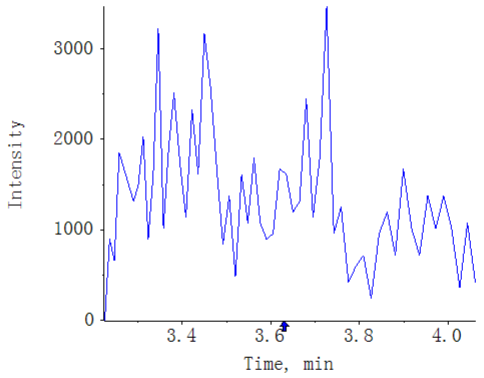

T24186683b_b
DHZR AREA:N/A S/N:N/A

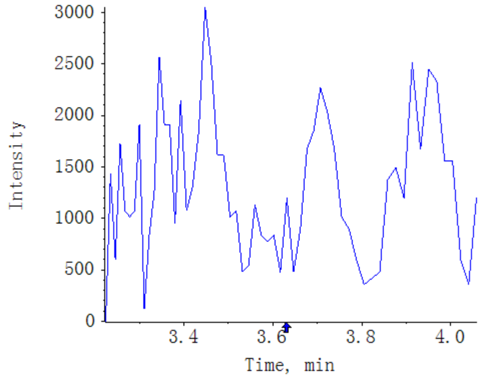

T24186683b_c
DHZR AREA:N/A S/N:N/A

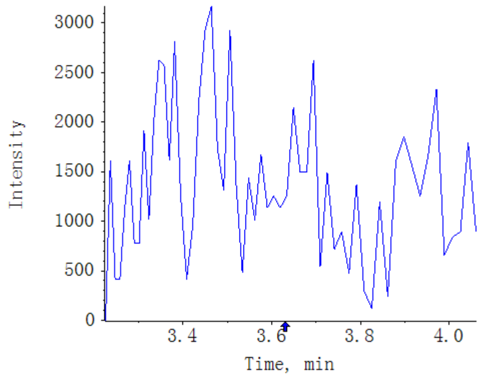

T24186684b_a
DHZR AREA:N/A S/N:N/A

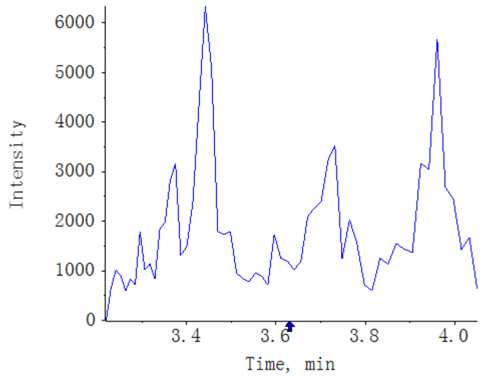

T24186684b_b
DHZR AREA:N/A S/N:N/A

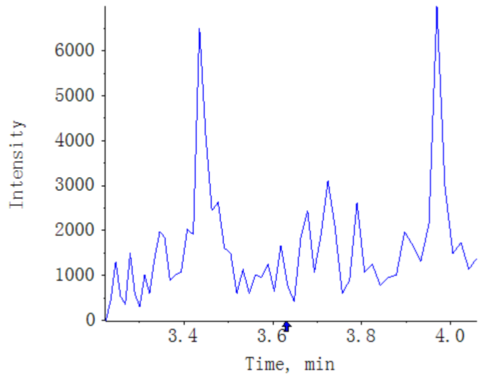

T24186684b_c
DHZR AREA:N/A S/N:N/A

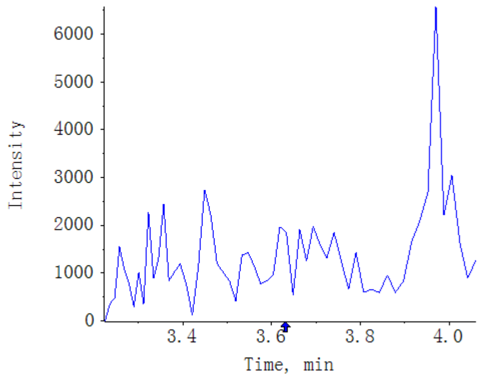

Result Table	MWXS-24-3064-a_9_WH6500-17_A20-3_V6.0_WSS_20240730	Algorithm Used	MQ4
Acquisition Method	ACC-PHs_V6.0_WH6500-17_CMY_20240521.dam	Instrument Name	Triple Quad 6500+ Low Mass
Project	N/A	Analytes QTY	109:23

Compound name: cZR (352.1 / 220.1)

Sample Name	Sample Type	Area (cps)	Is Area (cps)	RT (min)	S/N	Target Conc	Calculated Conc.()
STD_0.01ppb	Standard	1.98e4	6.534e6	3.72	10.6	0.0100	1.005313e-2
STD_0.05ppb	Standard	7.06e4	7.105e6	3.71	12.6	0.0500	4.862571e-2
STD_0.1ppb	Standard	1.30e5	6.945e6	3.72	16.8	0.1000	9.761124e-2
STD_0.5ppb	Standard	6.02e5	5.879e6	3.71	15.0	0.5000	5.650520e-1
STD_1ppb	Standard	1.01e6	5.871e6	3.71	17.1	1.0000	9.513779e-1
STD_5ppb	Standard	5.65e6	5.223e6	3.73	19.2	5.0000	6.037402e0
STD_10ppb	Standard	9.59e6	5.065e6	3.71	14.7	10.0000	1.057632e1
STD_50ppb	Standard	4.52e7	4.847e6	3.71	18.0	50.0000	5.209929e1
STD_100ppb	Standard	7.18e7	4.595e6	3.72	17.2	100.0000	8.733033e1
STD_200ppb	Standard	1.19e8	4.227e6	3.71	12.9	200.0000	1.568355e2
STD_500ppb	Standard	N/A	3.238e6	N/A	N/A	500.0000	N/A
V2.0_MW_RQC1_20240724	Quality Control	1.38e5	5.942e6	3.71	30.9	0.0000	1.229367e-1
Blank	Unknown	N/A	2.845e3	N/A	N/A	N/A	N/A
V3.0_MWMS_20240725_1	Unknown	1.10e7	1.146e7	3.71	14.0	N/A	5.358366e0
MWXS243064a_R1	Quality Control	1.09e5	6.293e6	3.69	17.1	0.0000	8.945375e-2
MWXS243064a_R2	Quality Control	1.01e5	6.413e6	3.70	18.7	0.0000	8.141607e-2
MWXS243064a_R3	Quality Control	1.13e5	6.529e6	3.69	19.8	0.0000	9.022144e-2
T24186682b_a	Unknown	1.01e5	6.437e6	3.70	17.6	N/A	8.095856e-2
T24186682b_b	Unknown	1.00e5	6.323e6	3.70	16.0	N/A	8.162665e-2
T24186682b_c	Unknown	1.13e5	6.686e6	3.70	13.2	N/A	8.786058e-2
T24186683b_a	Unknown	1.56e5	6.369e6	3.70	41.3	N/A	1.303769e-1
T24186683b_b	Unknown	1.36e5	6.536e6	3.70	25.2	N/A	1.092501e-1
T24186683b_c	Unknown	1.55e5	6.791e6	3.71	27.0	N/A	1.207475e-1
T24186684b_a	Unknown	8.52e4	5.743e6	3.70	9.5	N/A	7.595268e-2
T24186684b_b	Unknown	8.27e4	6.364e6	3.71	14.4	N/A	6.574936e-2
T24186684b_c	Unknown	6.77e4	6.178e6	3.71	10.2	N/A	5.430722e-2

Compound name: cZR
Regression Equation: y = 0.17898 x + 0.00123 (r = 0.99218) (weighting: 1 / x^2)

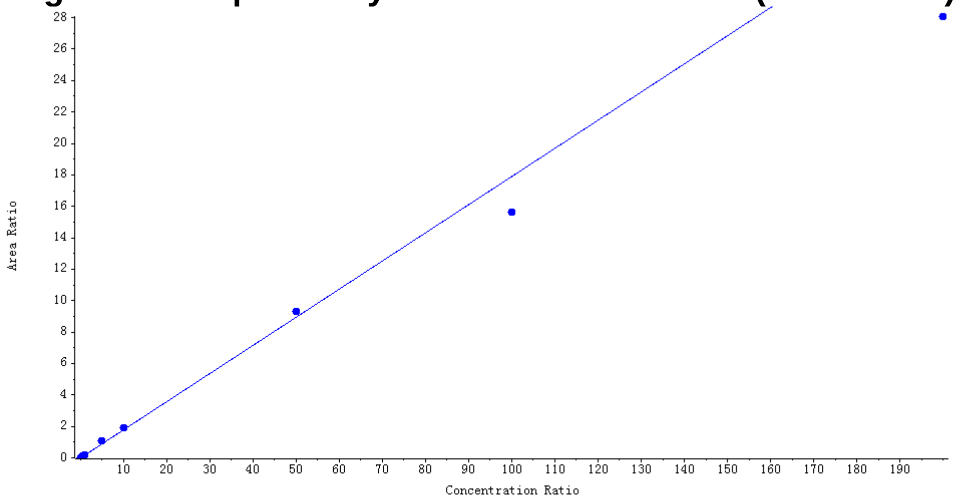

Peak Review

Blank
cZR AREA:N/A S/N:N/A

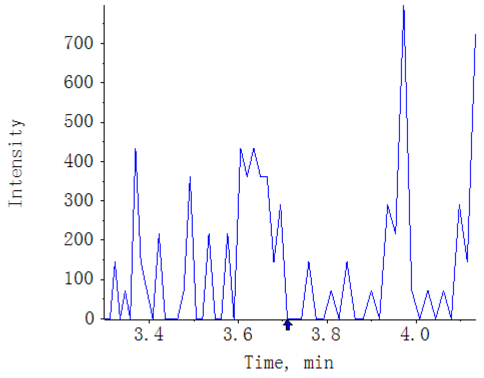

V3.0_MWMS_20240725_1
cZR AREA:1.10e7 S/N:14.0

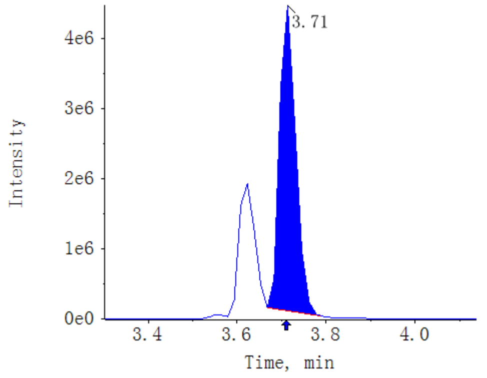

T24186682b_a
cZR AREA:1.01e5 S/N:17.6

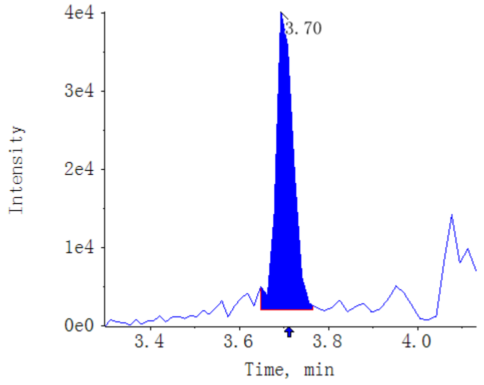

T24186682b_b
cZR AREA:1.00e5 S/N:16.0

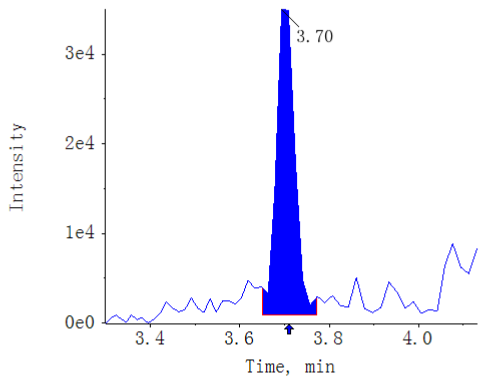

T24186682b_c
cZR AREA:1.13e5 S/N:13.2

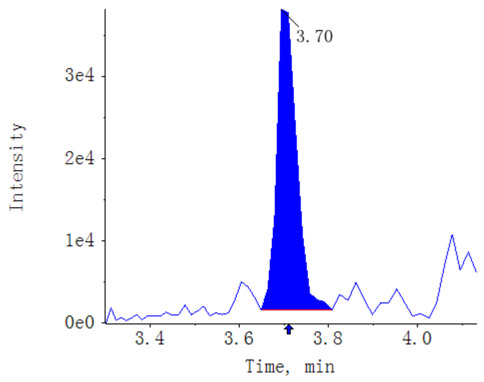

T24186683b_a
cZR AREA:1.56e5 S/N:41.3

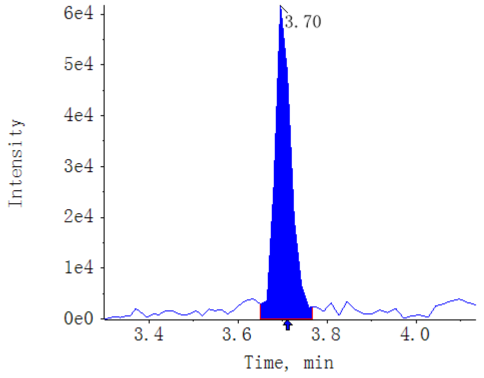

T24186683b_b
cZR AREA:1.36e5 S/N:25.2

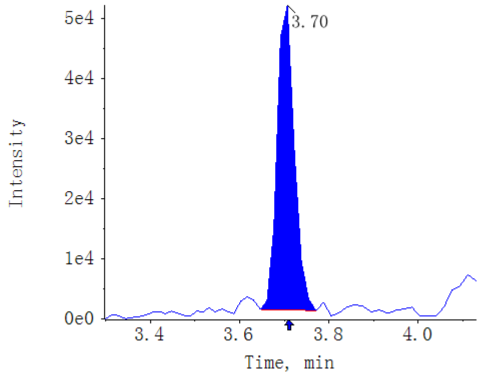

T24186683b_c
cZR AREA:1.55e5 S/N:27.0

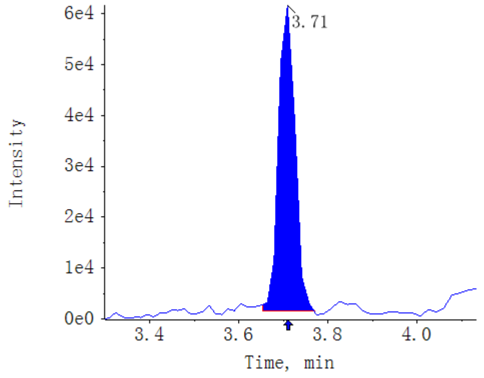

T24186684b_a
cZR AREA:8.52e4 S/N:9.5

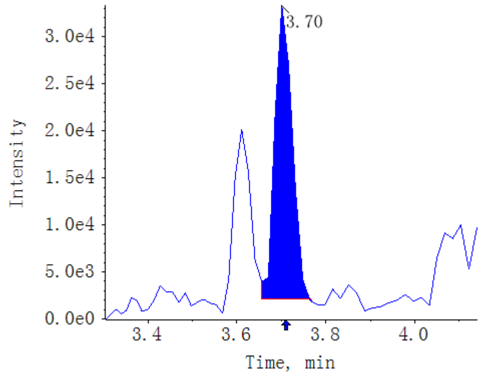

T24186684b_b
cZR AREA:8.27e4 S/N:14.4

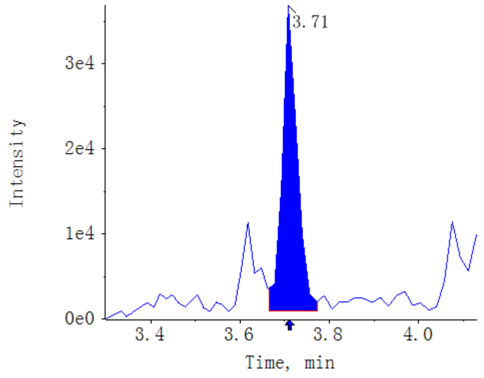

T24186684b_c
cZR AREA:6.77e4 S/N:10.2

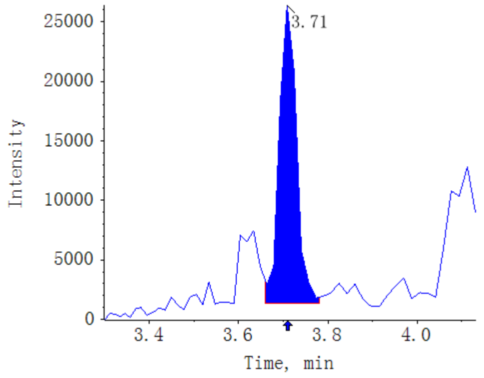

Result Table	MWXS-24-3064-a_9_WH6500-17_A20-3_V6.0_WSS_20240730	Algorithm Used	MQ4
Acquisition Method	ACC-PHs_V6.0_WH6500-17_CMY_20240521.dam	Instrument Name	Triple Quad 6500+ Low Mass
Project	N/A	Analytes QTY	109:24

Compound name: pT9G (404.1 / 242.1)

Sample Name	Sample Type	Area (cps)	Is Area (cps)	RT (min)	S/N	Target Conc	Calculated Conc.()
STD_0.01ppb	Standard	N/A	3.065e6	N/A	N/A	0.0100	N/A
STD_0.05ppb	Standard	2.59e4	3.030e6	3.58	10.6	0.0500	3.985055e-2
STD_0.1ppb	Standard	4.72e4	3.037e6	3.59	7.0	0.1000	9.129790e-2
STD_0.5ppb	Standard	2.35e5	2.659e6	3.58	7.6	0.5000	6.263032e-1
STD_1ppb	Standard	3.87e5	2.814e6	3.58	8.8	1.0000	9.858398e-1
STD_5ppb	Standard	1.91e6	2.801e6	3.59	7.9	5.0000	4.986988e0
STD_10ppb	Standard	3.46e6	2.618e6	3.58	8.7	10.0000	9.690009e0
STD_50ppb	Standard	1.84e7	2.412e6	3.58	8.3	50.0000	5.611320e1
STD_100ppb	Standard	3.00e7	2.237e6	3.59	10.7	100.0000	9.846379e1
STD_200ppb	Standard	5.59e7	2.096e6	3.58	9.3	200.0000	1.956527e2
STD_500ppb	Standard	N/A	1.785e6	N/A	N/A	500.0000	N/A
V2.0_MW_RQC1_20240724	Quality Control	N/A	1.511e6	N/A	N/A	0.0000	N/A
Blank	Unknown	N/A	9.253e2	N/A	N/A	N/A	N/A
V3.0_MWMS_20240725_1	Unknown	4.01e6	4.177e6	3.59	10.2	N/A	7.017675e0
MWXS243064a_R1	Quality Control	N/A	1.316e6	N/A	N/A	0.0000	N/A
MWXS243064a_R2	Quality Control	N/A	1.226e6	N/A	N/A	0.0000	N/A
MWXS243064a_R3	Quality Control	N/A	1.274e6	N/A	N/A	0.0000	N/A
T24186682b_a	Unknown	N/A	1.337e6	N/A	N/A	N/A	N/A
T24186682b_b	Unknown	N/A	1.303e6	N/A	N/A	N/A	N/A
T24186682b_c	Unknown	N/A	1.287e6	N/A	N/A	N/A	N/A
T24186683b_a	Unknown	N/A	1.219e6	N/A	N/A	N/A	N/A
T24186683b_b	Unknown	N/A	1.264e6	N/A	N/A	N/A	N/A
T24186683b_c	Unknown	N/A	1.317e6	N/A	N/A	N/A	N/A
T24186684b_a	Unknown	N/A	1.242e6	N/A	N/A	N/A	N/A
T24186684b_b	Unknown	N/A	1.186e6	N/A	N/A	N/A	N/A
T24186684b_c	Unknown	N/A	1.175e6	N/A	N/A	N/A	N/A

Compound name: pT9G
Regression Equation: y = 0.13622 x + 0.00312 (r = 0.99875) (weighting: 1 / x)

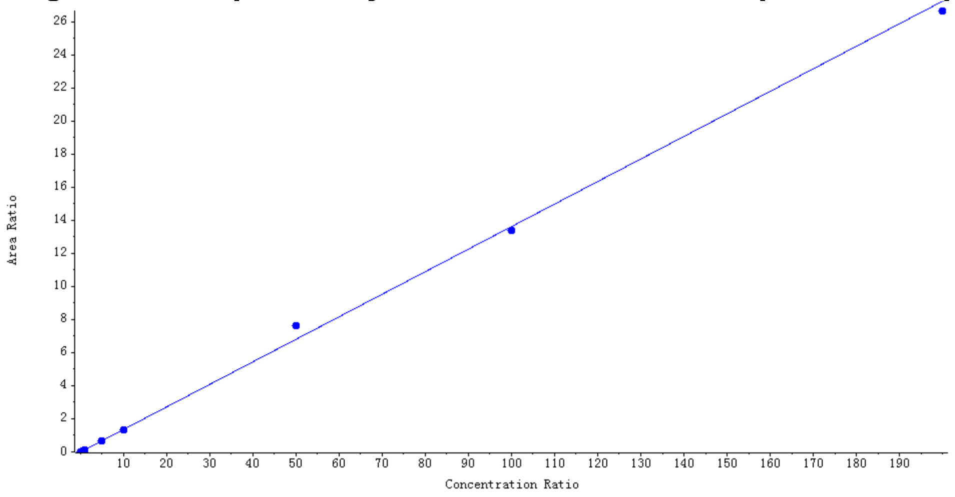

Peak Review

Blank
pT9G AREA:N/A S/N:N/A

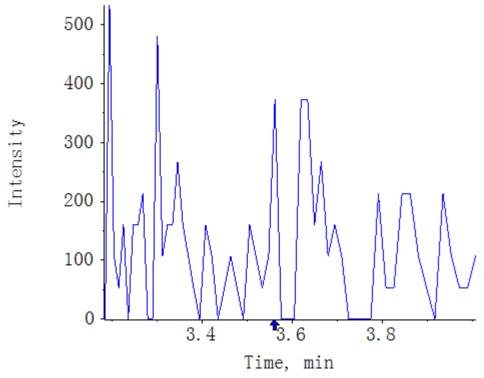

V3.0_MWMS_20240725_1
pT9G AREA:4.01e6 S/N:10.2

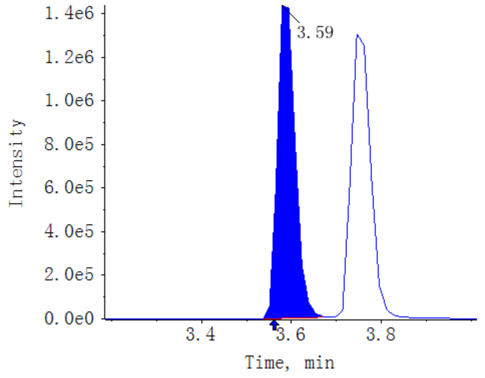

T24186682b_a
pT9G AREA:N/A S/N:N/A

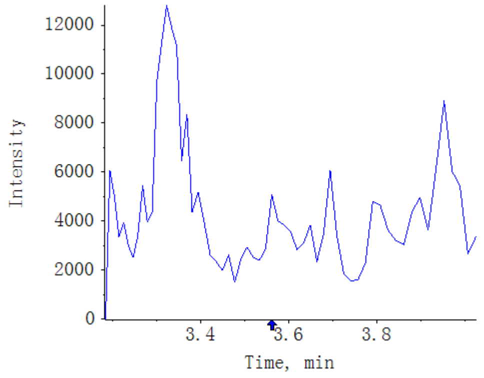

T24186682b_b
pT9G AREA:N/A S/N:N/A

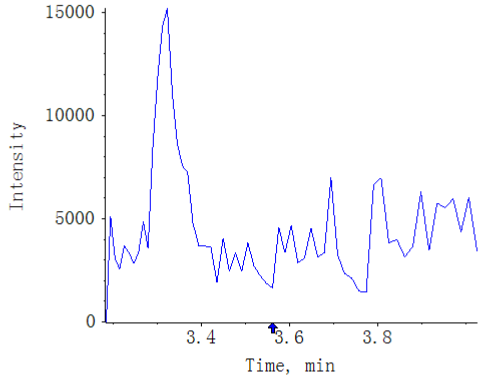

T24186682b_c
pT9G AREA:N/A S/N:N/A

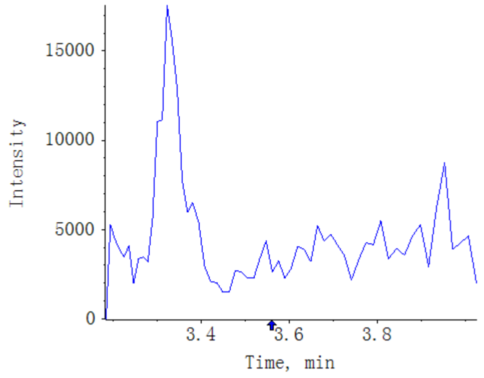

T24186683b_a
pT9G AREA:N/A S/N:N/A

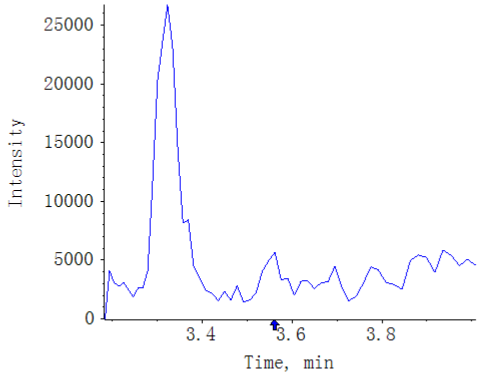

T24186683b_b
pT9G AREA:N/A S/N:N/A

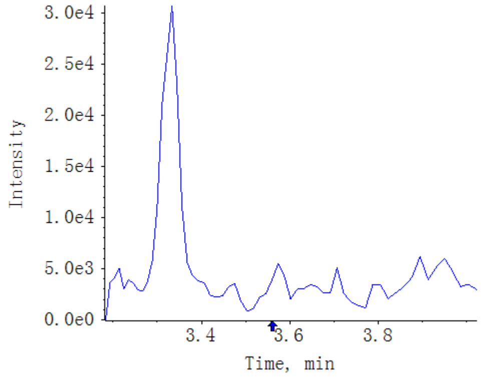

T24186683b_c
pT9G AREA:N/A S/N:N/A

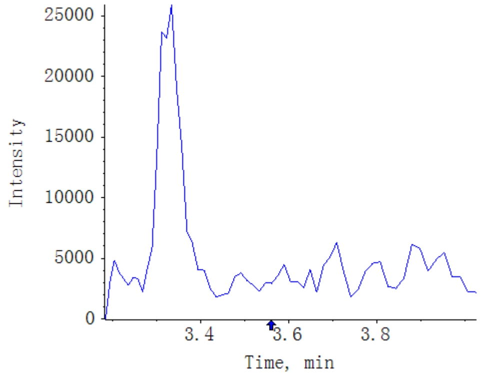

T24186684b_a
pT9G AREA:N/A S/N:N/A

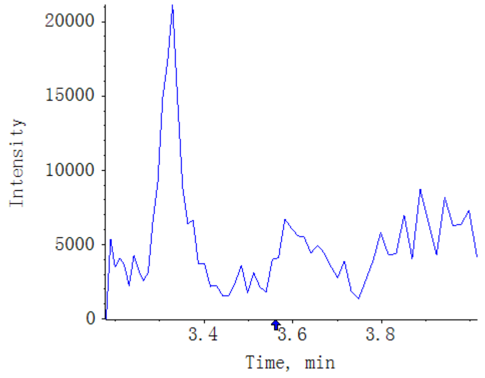

T24186684b_b
pT9G AREA:N/A S/N:N/A

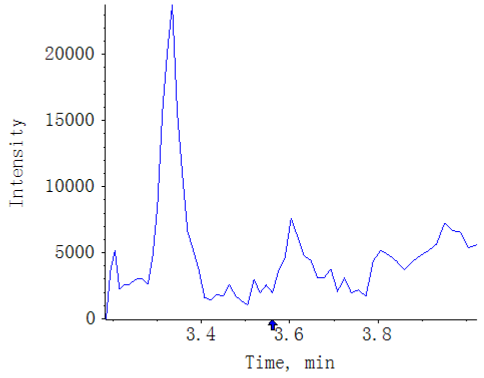

T24186684b_c
pT9G AREA:N/A S/N:N/A

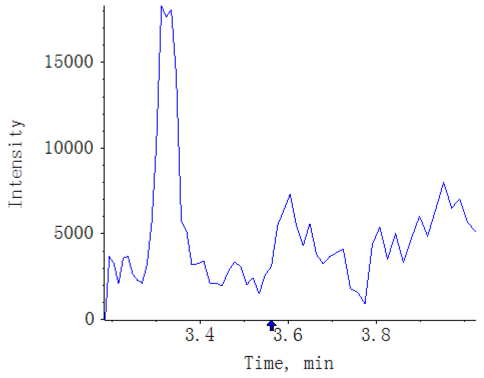

Result Table	MWXS-24-3064-a_9_WH6500-17_A20-3_V6.0_WSS_20240730	Algorithm Used	MQ4
Acquisition Method	ACC-PHs_V6.0_WH6500-17_CMY_20240521.dam	Instrument Name	Triple Quad 6500+ Low Mass
Project	N/A	Analytes QTY	109:25

Compound name: 2CltZ (254.3 / 170.2)

Sample Name	Sample Type	Area (cps)	Is Area (cps)	RT (min)	S/N	Target Conc	Calculated Conc.()
STD_0.01ppb	Standard	3.65e3	2.440e6	4.47	8.9	0.0100	1.184438e-2
STD_0.05ppb	Standard	1.80e4	2.593e6	4.46	15.5	0.0500	5.015782e-2
STD_0.1ppb	Standard	3.13e4	2.495e6	4.47	26.5	0.1000	8.942381e-2
STD_0.5ppb	Standard	1.52e5	2.073e6	4.46	53.1	0.5000	5.157284e-1
STD_1ppb	Standard	2.32e5	2.115e6	4.46	47.9	1.0000	7.716257e-1
STD_5ppb	Standard	1.39e6	1.775e6	4.47	53.2	5.0000	5.484962e0
STD_10ppb	Standard	2.57e6	1.678e6	4.46	54.6	10.0000	1.074501e1
STD_50ppb	Standard	1.28e7	1.824e6	4.45	51.3	50.0000	4.937428e1
STD_100ppb	Standard	2.39e7	1.835e6	4.47	59.2	100.0000	9.160419e1
STD_200ppb	Standard	4.89e7	1.650e6	4.46	71.0	200.0000	2.080128e2
STD_500ppb	Standard	N/A	1.698e6	N/A	N/A	500.0000	N/A
V2.0_MW_RQC1_20240724	Quality Control	N/A	3.188e6	N/A	N/A	0.0000	N/A
Blank	Unknown	N/A	3.390e3	N/A	N/A	N/A	N/A
V3.0_MWMS_20240725_1	Unknown	2.46e6	3.932e6	4.45	38.1	N/A	4.390958e0
MWXS243064a_R1	Quality Control	N/A	2.990e6	N/A	N/A	0.0000	N/A
MWXS243064a_R2	Quality Control	N/A	3.099e6	N/A	N/A	0.0000	N/A
MWXS243064a_R3	Quality Control	N/A	3.088e6	N/A	N/A	0.0000	N/A
T24186682b_a	Unknown	N/A	3.106e6	N/A	N/A	N/A	N/A
T24186682b_b	Unknown	N/A	3.036e6	N/A	N/A	N/A	N/A
T24186682b_c	Unknown	N/A	3.135e6	N/A	N/A	N/A	N/A
T24186683b_a	Unknown	N/A	2.973e6	N/A	N/A	N/A	N/A
T24186683b_b	Unknown	N/A	2.971e6	N/A	N/A	N/A	N/A
T24186683b_c	Unknown	N/A	3.009e6	N/A	N/A	N/A	N/A
T24186684b_a	Unknown	N/A	2.782e6	N/A	N/A	N/A	N/A
T24186684b_b	Unknown	N/A	3.028e6	N/A	N/A	N/A	N/A
T24186684b_c	Unknown	N/A	2.906e6	N/A	N/A	N/A	N/A

Compound name: 2CltZ
Regression Equation: y = 0.14233 x + -1.90530e-4 (r = 0.99838) (weighting: 1 / x)

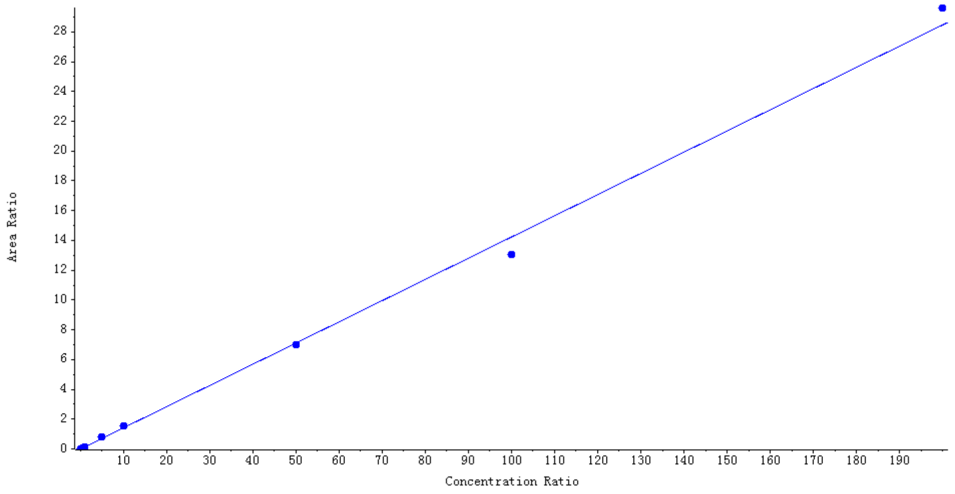

Peak Review

Blank
2CltZ AREA:N/A S/N:N/A

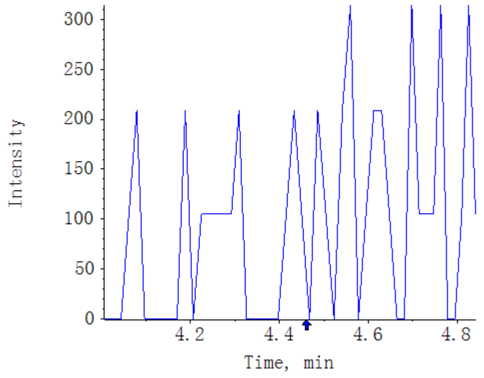

V3.0_MWMS_20240725_1
2CltZ AREA:2.46e6 S/N:38.1

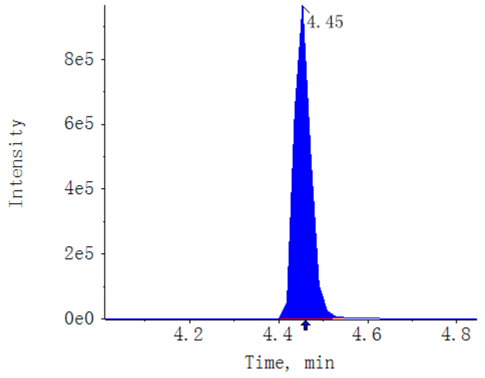

T24186682b_a
2CltZ AREA:N/A S/N:N/A

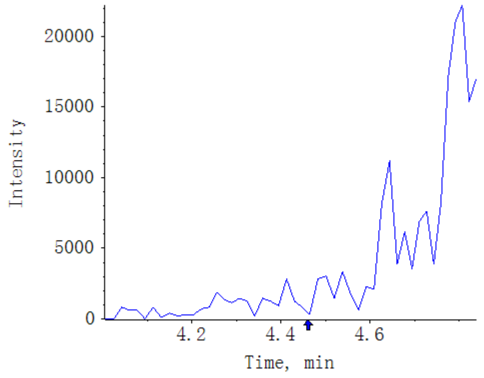

T24186682b_b
2CltZ AREA:N/A S/N:N/A

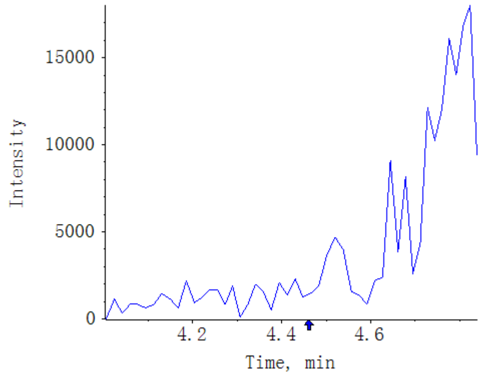

T24186682b_c
2CltZ AREA:N/A S/N:N/A

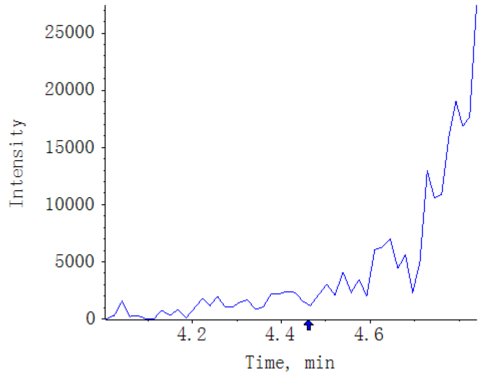

T24186683b_a
2CltZ AREA:N/A S/N:N/A

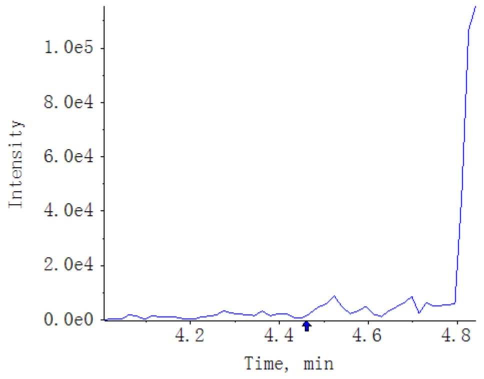

T24186683b_b
2CltZ AREA:N/A S/N:N/A

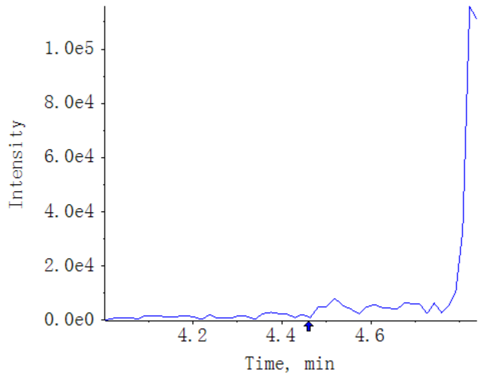

T24186683b_c
2CltZ AREA:N/A S/N:N/A

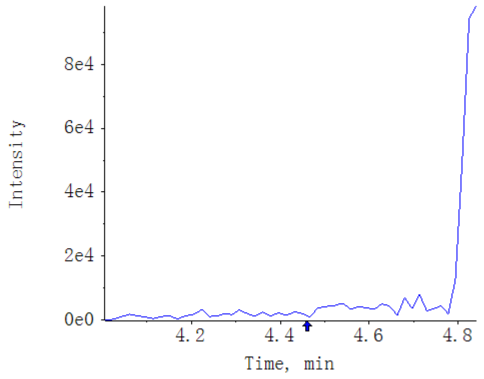

T24186684b_a
2CltZ AREA:N/A S/N:N/A

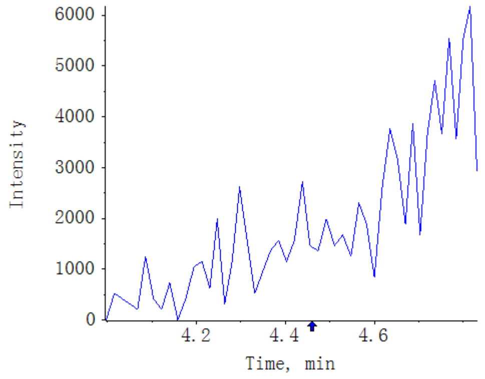

T24186684b_b
2CltZ AREA:N/A S/N:N/A

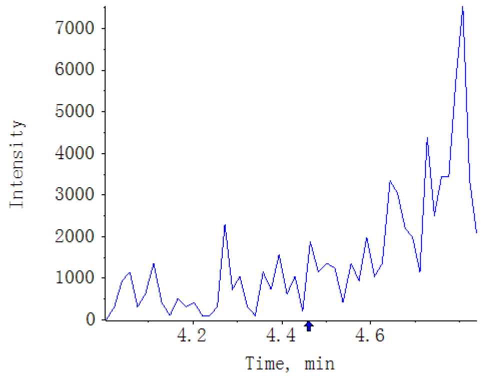

T24186684b_c
2CltZ AREA:N/A S/N:N/A

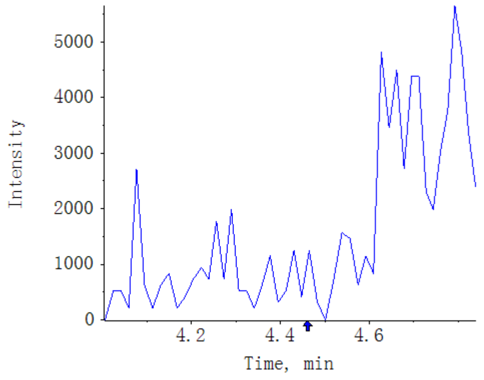

**Result Table**	MWXS-24-3064-a_9_WH6500-17_A20-3_V6.0_WSS_20240730	**Algorithm Used**	MQ4
**Acquisition Method**	ACC-PHs_V6.0_WH6500-17_CMY_20240521.dam	**Instrument Name**	Triple Quad 6500+ Low Mass
**Project**	N/A	**Analytes QTY**	109:26

Compound name: pT (242.1 / 136.2)

Sample Name	Sample Type	Area (cps)	Is Area (cps)	RT (min)	S/N	Target Conc	Calculated Conc.()
STD_0.01ppb	Standard	4.36e3	5.126e6	3.57	6.5	0.0100	9.872363e-3
STD_0.05ppb	Standard	1.89e4	5.373e6	3.57	16.8	0.0500	5.052835e-2
STD_0.1ppb	Standard	3.94e4	5.288e6	3.59	21.4	0.1000	1.105414e-1
STD_0.5ppb	Standard	1.61e5	4.766e6	3.58	26.4	0.5000	5.118190e-1
STD_1ppb	Standard	3.02e5	4.828e6	3.58	26.0	1.0000	9.517285e-1
STD_5ppb	Standard	1.61e6	4.592e6	3.59	35.3	5.0000	5.343940e0
STD_10ppb	Standard	2.90e6	4.488e6	3.57	37.4	10.0000	9.878049e0
STD_50ppb	Standard	1.37e7	4.108e6	3.57	36.3	50.0000	5.107137e1
STD_100ppb	Standard	2.42e7	4.042e6	3.58	41.0	100.0000	9.142364e1
STD_200ppb	Standard	4.97e7	3.907e6	3.57	29.7	200.0000	1.941408e2
STD_500ppb	Standard	1.12e8	3.576e6	3.57	31.6	500.0000	4.792277e2
V2.0_MW_RQC1_20240724	Quality Control	N/A	2.523e6	N/A	N/A	0.0000	N/A
Blank	Unknown	N/A	9.419e2	N/A	N/A	N/A	N/A
V3.0_MWMS_20240725_1	Unknown	3.11e6	6.790e6	3.59	30.4	N/A	6.987415e0
MWXS243064a_R1	Quality Control	1.35e4	3.675e6	3.61	5.3	0.0000	5.282532e-2
MWXS243064a_R2	Quality Control	1.39e4	3.717e6	3.63	6.1	0.0000	5.398916e-2
MWXS243064a_R3	Quality Control	1.22e4	3.772e6	3.61	7.9	0.0000	4.625665e-2
T24186682b_a	Unknown	7.17e3	3.706e6	3.61	6.6	N/A	2.642774e-2
T24186682b_b	Unknown	1.18e4	3.576e6	3.62	8.5	N/A	4.714821e-2
T24186682b_c	Unknown	1.17e4	3.670e6	3.62	6.1	N/A	4.554366e-2
T24186683b_a	Unknown	1.52e4	4.075e6	3.62	5.4	N/A	5.386481e-2
T24186683b_b	Unknown	1.34e4	4.132e6	3.63	6.1	N/A	4.650528e-2
T24186683b_c	Unknown	1.60e4	4.123e6	3.64	3.9	N/A	5.628911e-2
T24186684b_a	Unknown	1.75e4	3.376e6	3.62	6.6	N/A	7.588607e-2
T24186684b_b	Unknown	1.67e4	3.611e6	3.64	5.0	N/A	6.741680e-2
T24186684b_c	Unknown	1.40e4	3.357e6	3.63	6.0	N/A	6.068079e-2

Compound name: pT
Regression Equation: y = 0.06549 x + 2.04188e-4 (r = 0.99842) (weighting: 1 / x^2)

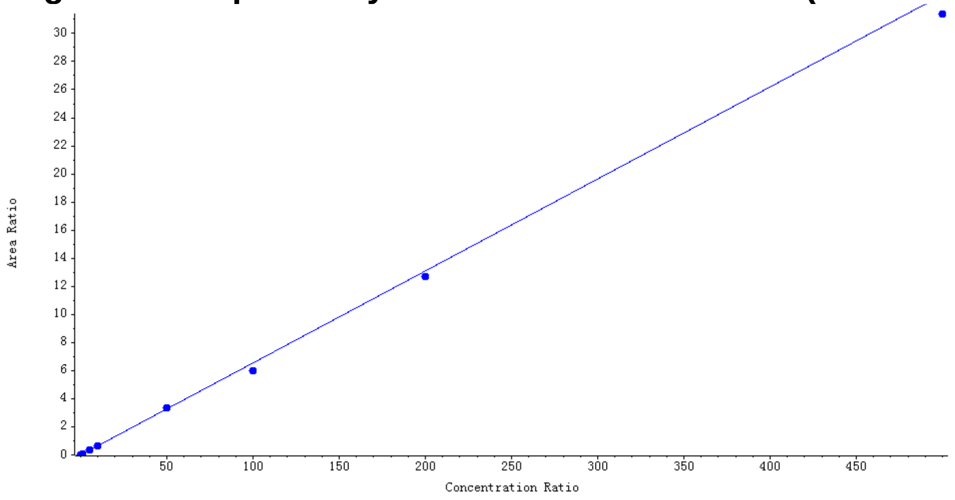

Peak Review

Blank
pT AREA:N/A S/N:N/A

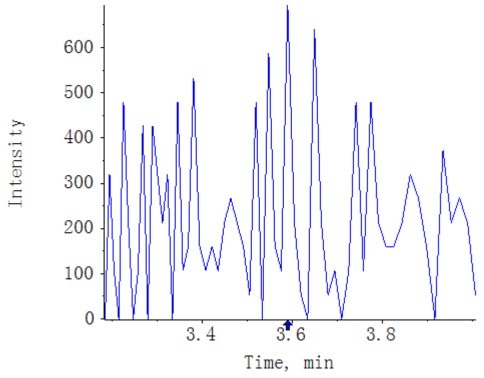

V3.0_MWMS_20240725_1
pT AREA:3.11e6 S/N:30.4

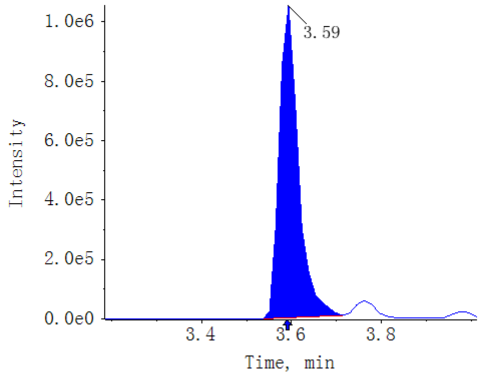

T24186682b_a
pT AREA:7.17e3 S/N:6.6

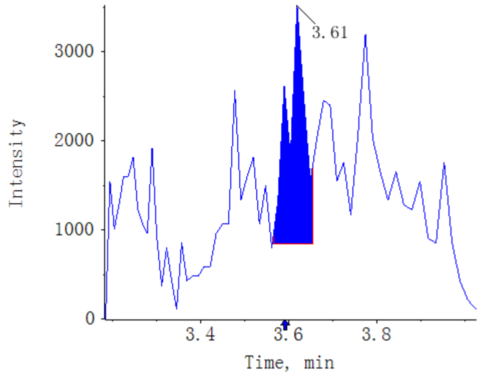

T24186682b_b
pT AREA:1.18e4 S/N:8.5

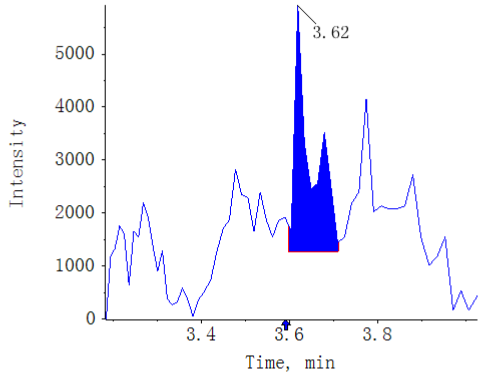

T24186682b_c
pT AREA:1.17e4 S/N:6.1

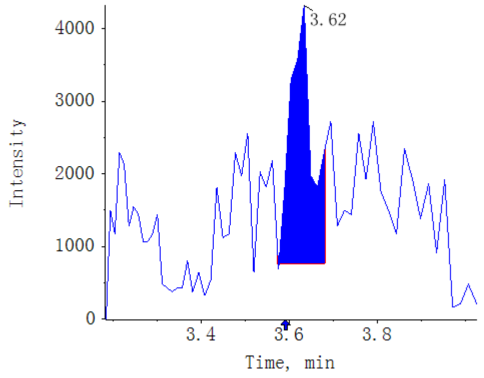

T24186683b_a
pT AREA:1.52e4 S/N:5.4

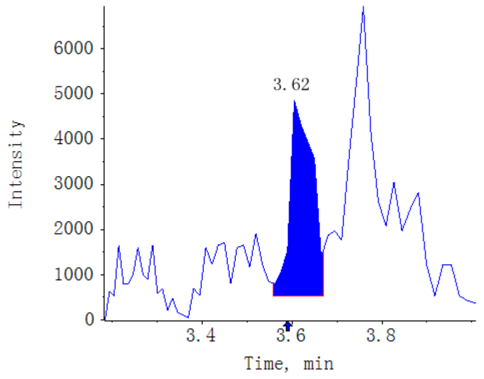

T24186683b_b
pT AREA:1.34e4 S/N:6.1

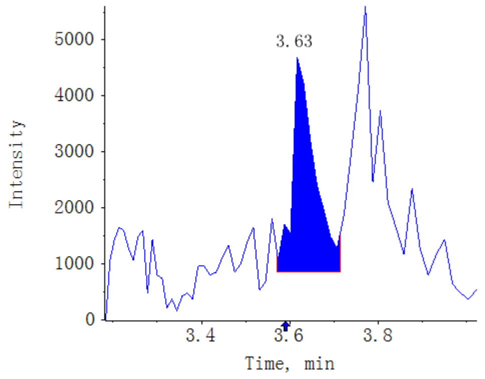

T24186683b_c
pT AREA:1.60e4 S/N:3.9

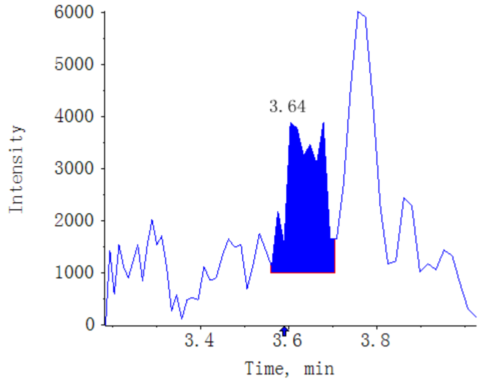

T24186684b_a
pT AREA:1.75e4 S/N:6.6

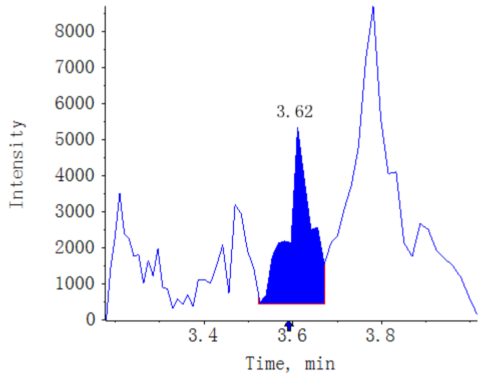

T24186684b_b
pT AREA:1.67e4 S/N:5.0

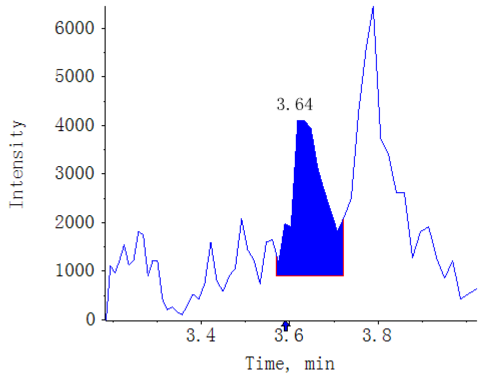

T24186684b_c
pT AREA:1.40e4 S/N:6.0

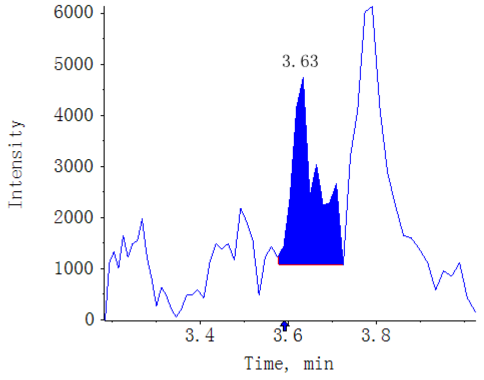

Result Table	MWXS-24-3064-a_9_WH6500-17_A20-3_V6.0_WSS_20240730	Algorithm Used	MQ4
Acquisition Method	ACC-PHs_V6.0_WH6500-17_CMY_20240521.dam	Instrument Name	Triple Quad 6500+ Low Mass
Project	N/A	Analytes QTY	109:27

Compound name: mT (242.1 / 107.1)

Sample Name	Sample Type	Area (cps)	Is Area (cps)	RT (min)	S/N	Target Conc	Calculated Conc.()
STD_0.01ppb	Standard	9.46e3	5.126e6	3.76	10.8	0.0100	9.913873e-3
STD_0.05ppb	Standard	2.95e4	5.373e6	3.76	16.5	0.0500	5.074008e-2
STD_0.1ppb	Standard	5.33e4	5.288e6	3.76	16.4	0.1000	1.021290e-1
STD_0.5ppb	Standard	2.49e5	4.766e6	3.75	14.9	0.5000	5.759005e-1
STD_1ppb	Standard	4.47e5	4.828e6	3.75	15.3	1.0000	1.028159e0
STD_5ppb	Standard	2.23e6	4.592e6	3.77	20.1	5.0000	5.441210e0
STD_10ppb	Standard	4.16e6	4.488e6	3.75	18.0	10.0000	1.037072e1
STD_50ppb	Standard	1.93e7	4.108e6	3.75	14.4	50.0000	5.259531e1
STD_100ppb	Standard	3.27e7	4.042e6	3.76	17.9	100.0000	9.077271e1
STD_200ppb	Standard	6.25e7	3.907e6	3.76	12.6	200.0000	1.794438e2
STD_500ppb	Standard	1.29e8	3.576e6	3.76	12.9	500.0000	4.051971e2
V2.0_MW_RQC1_20240724	Quality Control	N/A	2.523e6	N/A	N/A	0.0000	N/A
Blank	Unknown	N/A	9.419e2	N/A	N/A	N/A	N/A
V3.0_MWMS_20240725_1	Unknown	4.55e6	6.790e6	3.76	14.7	N/A	7.506757e0
MWXS243064a_R1	Quality Control	N/A	3.675e6	N/A	N/A	0.0000	N/A
MWXS243064a_R2	Quality Control	N/A	3.717e6	N/A	N/A	0.0000	N/A
MWXS243064a_R3	Quality Control	N/A	3.772e6	N/A	N/A	0.0000	N/A
T24186682b_a	Unknown	N/A	3.706e6	N/A	N/A	N/A	N/A
T24186682b_b	Unknown	N/A	3.576e6	N/A	N/A	N/A	N/A
T24186682b_c	Unknown	N/A	3.670e6	N/A	N/A	N/A	N/A
T24186683b_a	Unknown	N/A	4.075e6	N/A	N/A	N/A	N/A
T24186683b_b	Unknown	N/A	4.132e6	N/A	N/A	N/A	N/A
T24186683b_c	Unknown	N/A	4.123e6	N/A	N/A	N/A	N/A
T24186684b_a	Unknown	N/A	3.376e6	N/A	N/A	N/A	N/A
T24186684b_b	Unknown	N/A	3.611e6	N/A	N/A	N/A	N/A
T24186684b_c	Unknown	N/A	3.357e6	N/A	N/A	N/A	N/A

Compound name: mT
Regression Equation: y = 0.08921 x + 9.60075e-4 (r = 0.99512) (weighting: 1 / x^2)

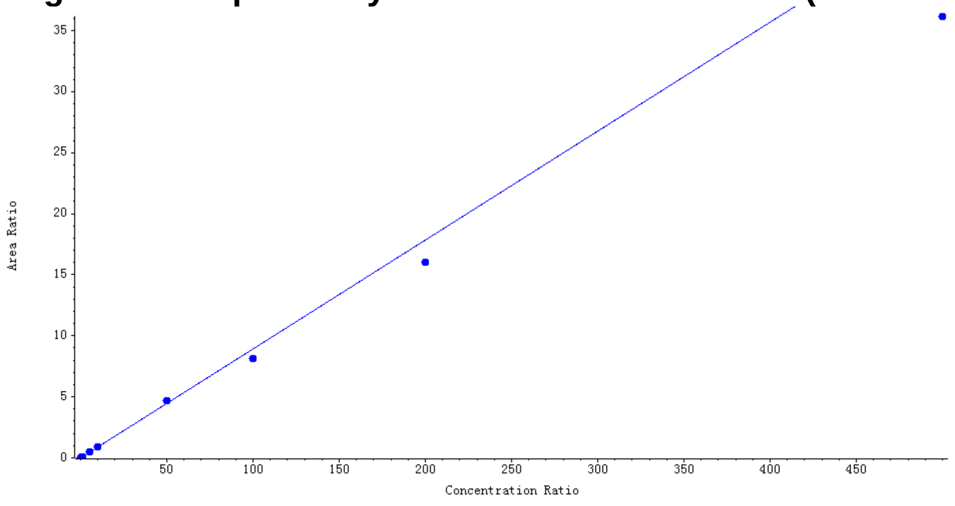

Peak Review

Blank

mT AREA:N/A S/N:N/A

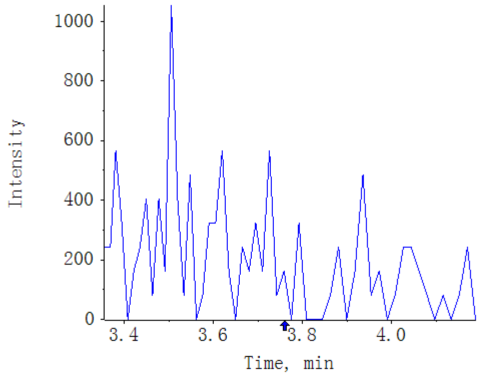

V3.0_MWMS_20240725_1

mT AREA:4.55e6 S/N:14.7

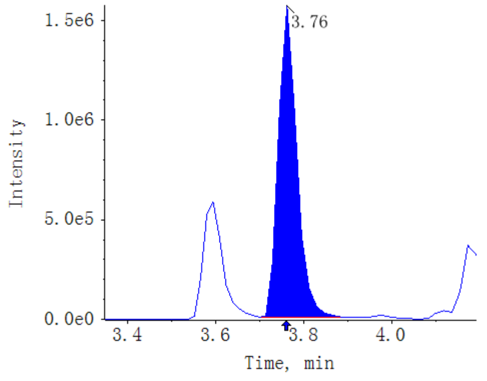

T24186682b_a

mT AREA:N/A S/N:N/A

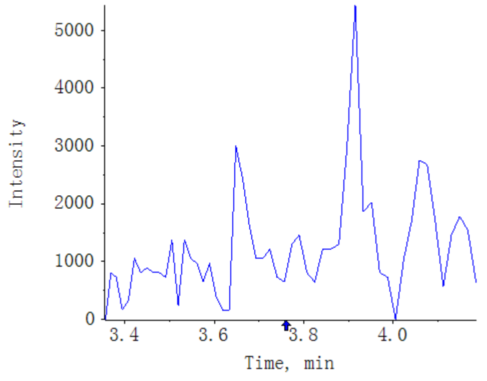

T24186682b_b

mT AREA:N/A S/N:N/A

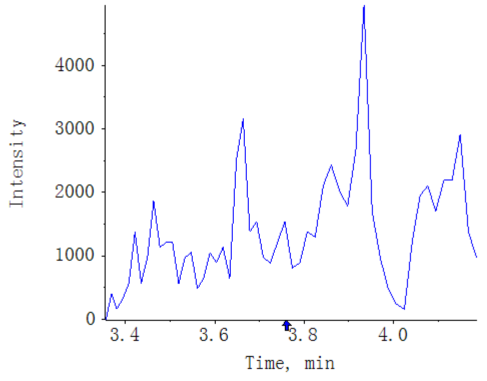

T24186682b_c

mT AREA:N/A S/N:N/A

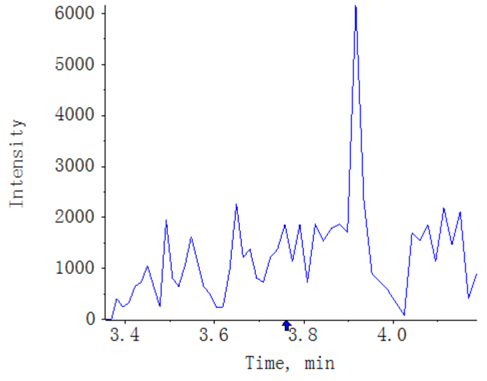

T24186683b_a

mT AREA:N/A S/N:N/A

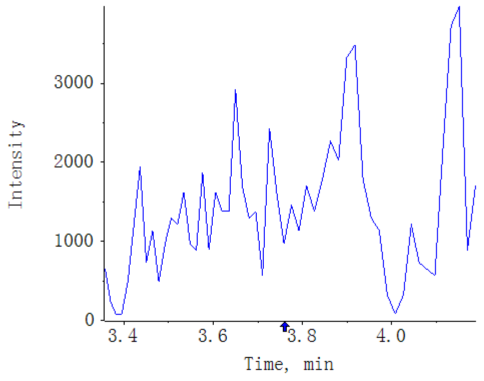

T24186683b_b

mT AREA:N/A S/N:N/A

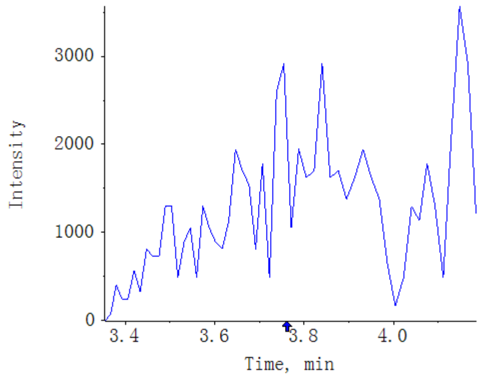

T24186683b_c

mT AREA:N/A S/N:N/A

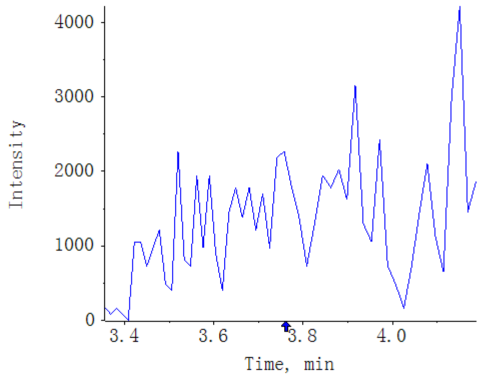

T24186684b_a

mT AREA:N/A S/N:N/A

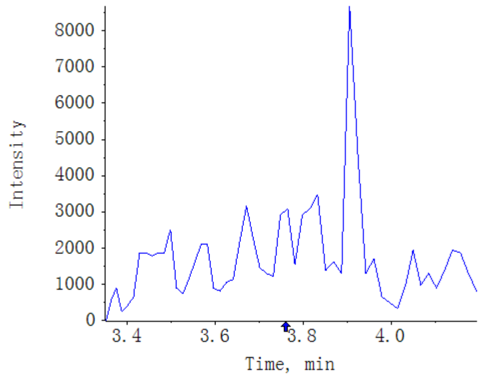

T24186684b_b

mT AREA:N/A S/N:N/A

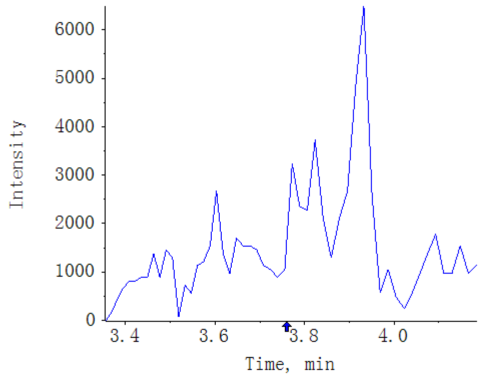

T24186684b_c

mT AREA:N/A S/N:N/A

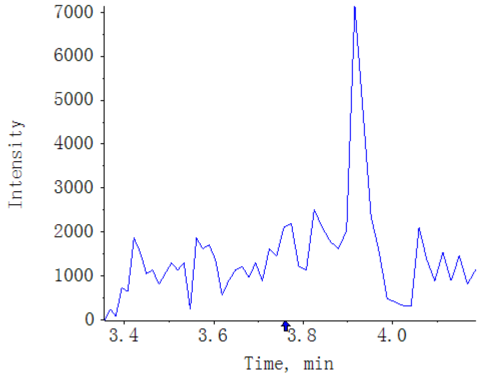

Result Table	MWXS-24-3064-a_9_WH6500-17_A20-3_V6.0_WSS_20240730	Algorithm Used	MQ4
Acquisition Method	ACC-PHs_V6.0_WH6500-17_CMY_20240521.dam	Instrument Name	Triple Quad 6500+ Low Mass
Project	N/A	Analytes QTY	109:28

Compound name: mTR (374.1 / 242.1)

Sample Name	Sample Type	Area (cps)	Is Area (cps)	RT (min)	S/N	Target Conc	Calculated Conc.()
STD_0.01ppb	Standard	N/A	3.065e6	N/A	N/A	0.0100	N/A
STD_0.05ppb	Standard	4.55e4	3.030e6	4.13	8.4	0.0500	4.803276e-2
STD_0.1ppb	Standard	8.45e4	3.037e6	4.13	8.7	0.1000	1.053651e-1
STD_0.5ppb	Standard	3.62e5	2.659e6	4.12	10.1	0.5000	5.895992e-1
STD_1ppb	Standard	5.71e5	2.814e6	4.13	8.9	1.0000	8.890560e-1
STD_5ppb	Standard	3.22e6	2.801e6	4.14	9.4	5.0000	5.118570e0
STD_10ppb	Standard	5.81e6	2.618e6	4.13	9.3	10.0000	9.916104e0
STD_50ppb	Standard	2.70e7	2.412e6	4.12	9.5	50.0000	5.002988e1
STD_100ppb	Standard	4.51e7	2.237e6	4.13	7.7	100.0000	9.015172e1
STD_200ppb	Standard	N/A	2.096e6	N/A	N/A	200.0000	N/A
STD_500ppb	Standard	N/A	1.785e6	N/A	N/A	500.0000	N/A
V2.0_MW_RQC1_20240724	Quality Control	N/A	1.511e6	N/A	N/A	0.0000	N/A
Blank	Unknown	N/A	9.253e2	N/A	N/A	N/A	N/A
V3.0_MWMS_20240725_1	Unknown	5.40e6	4.177e6	4.11	6.7	N/A	5.769043e0
MWXS243064a_R1	Quality Control	1.01e5	1.316e6	4.09	21.4	0.0000	3.252526e-1
MWXS243064a_R2	Quality Control	7.50e4	1.226e6	4.09	12.9	0.0000	2.547150e-1
MWXS243064a_R3	Quality Control	7.17e4	1.274e6	4.08	12.9	0.0000	2.327040e-1
T24186682b_a	Unknown	2.41e5	1.337e6	4.10	28.5	N/A	7.881055e-1
T24186682b_b	Unknown	2.41e5	1.303e6	4.09	26.6	N/A	8.076978e-1
T24186682b_c	Unknown	2.03e5	1.287e6	4.10	18.2	N/A	6.861705e-1
T24186683b_a	Unknown	2.70e4	1.219e6	4.09	5.7	N/A	7.995575e-2
T24186683b_b	Unknown	2.26e4	1.264e6	4.09	3.9	N/A	6.076593e-2
T24186683b_c	Unknown	2.90e4	1.317e6	4.10	5.6	N/A	7.935767e-2
T24186684b_a	Unknown	1.74e4	1.242e6	4.09	7.1	N/A	4.346425e-2
T24186684b_b	Unknown	9.33e3	1.186e6	4.09	5.5	N/A	1.601330e-2
T24186684b_c	Unknown	2.29e4	1.175e6	4.10	5.0	N/A	6.796221e-2

Compound name: mTR
Regression Equation: y = 0.22349 x + 0.00429 (r = 0.99494) (weighting: 1 / x^2)

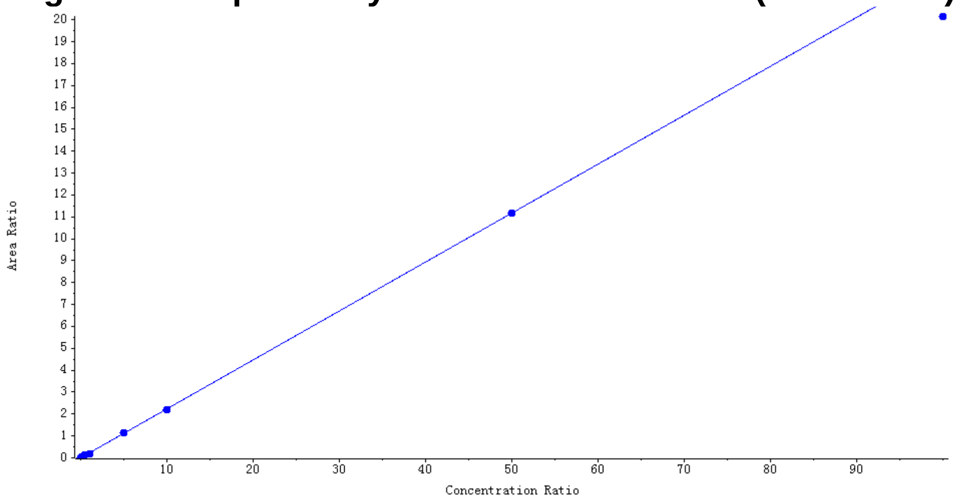

Peak Review

Blank
mTR AREA:N/A S/N:N/A

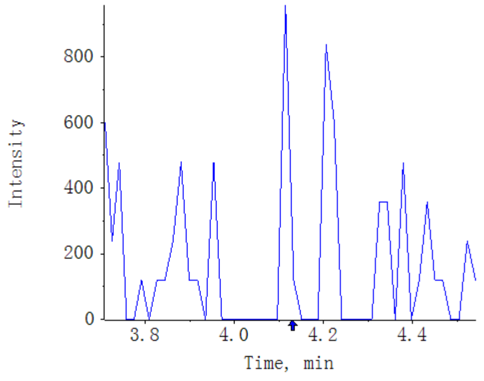

V3.0_MWMS_20240725_1
mTR AREA:5.40e6 S/N:6.7

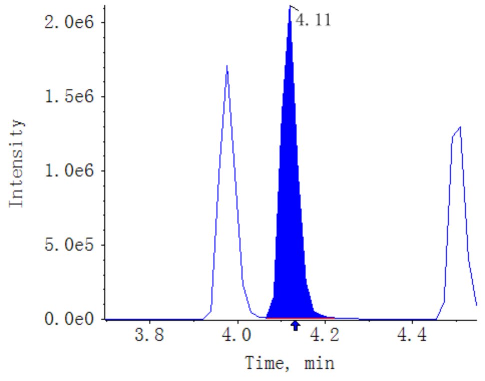

T24186682b_a
mTR AREA:2.41e5 S/N:28.5

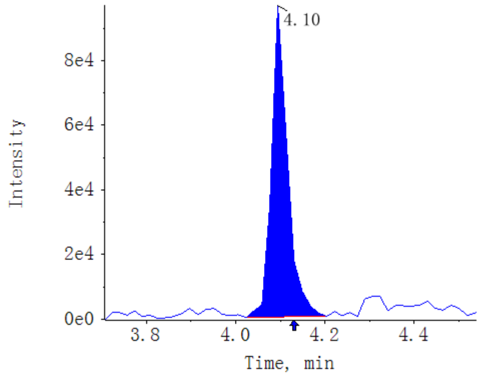

T24186682b_b
mTR AREA:2.41e5 S/N:26.6

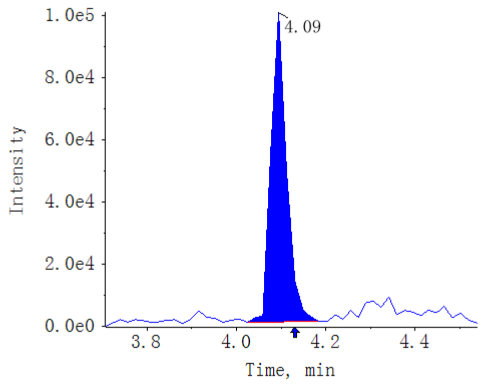

T24186682b_c
mTR AREA:2.03e5 S/N:18.2

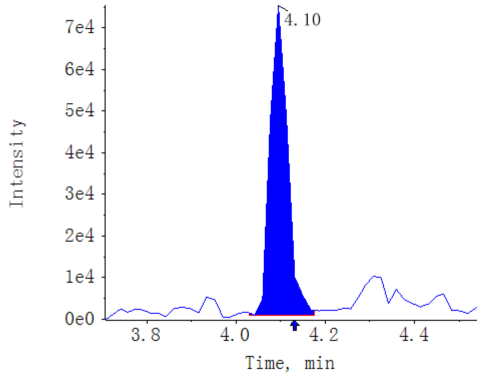

T24186683b_a
mTR AREA:2.70e4 S/N:5.7

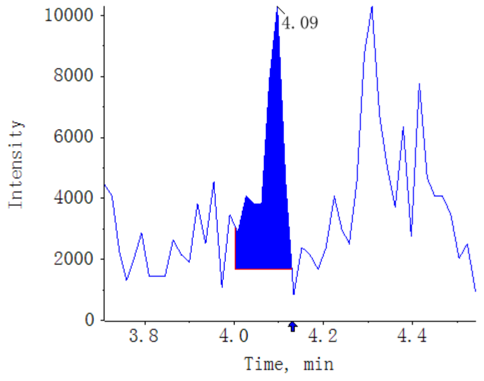

T24186683b_b
mTR AREA:2.26e4 S/N:3.9

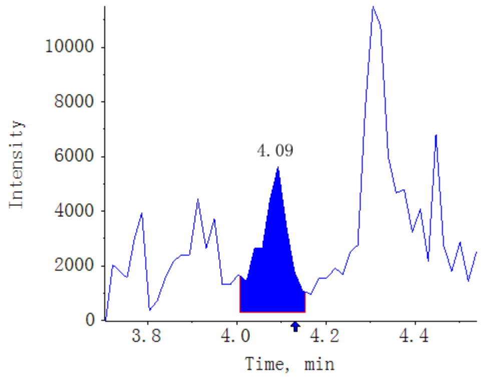

T24186683b_c
mTR AREA:2.90e4 S/N:5.6

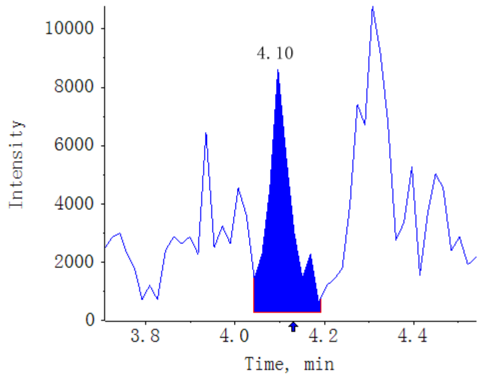

T24186684b_a
mTR AREA:1.74e4 S/N:7.1

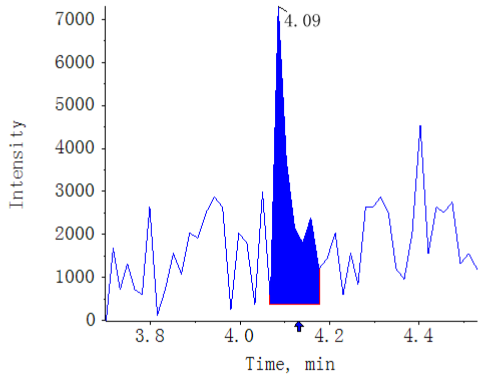

T24186684b_b
mTR AREA:9.33e3 S/N:5.5

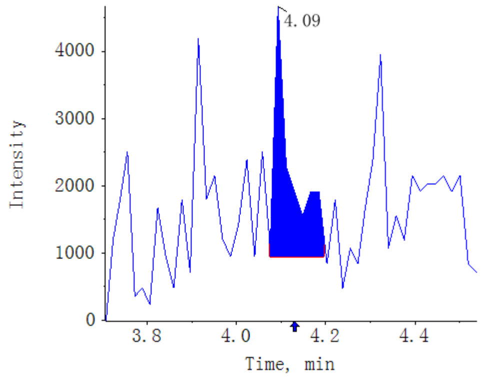

T24186684b_c
mTR AREA:2.29e4 S/N:5.0

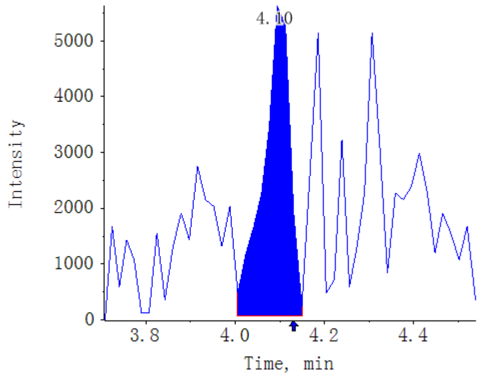

**Result Table**	MWXS-24-3064-a_9_WH6500-17_A20-3_V6.0_WSS_20240730	**Algorithm Used**	MQ4
**Acquisition Method**	ACC-PHs_V6.0_WH6500-17_CMY_20240521.dam	**Instrument Name**	Triple Quad 6500+ Low Mass
**Project**	N/A	**Analytes QTY**	109:29

Compound name: oT (242.1 / 136.1)

Sample Name	Sample Type	Area (cps)	Is Area (cps)	RT (min)	S/N	Target Conc	Calculated Conc.()
STD_0.01ppb	Standard	8.19e3	5.126e6	4.19	6.1	0.0100	9.756190e-3
STD_0.05ppb	Standard	4.20e4	5.373e6	4.18	21.5	0.0500	5.691587e-2
STD_0.1ppb	Standard	6.82e4	5.288e6	4.19	33.0	0.1000	9.546308e-2
STD_0.5ppb	Standard	3.27e5	4.766e6	4.18	38.9	0.5000	5.184512e-1
STD_1ppb	Standard	6.49e5	4.828e6	4.18	45.5	1.0000	1.017042e0
STD_5ppb	Standard	3.52e6	4.592e6	4.19	39.8	5.0000	5.815800e0
STD_10ppb	Standard	6.04e6	4.488e6	4.18	41.2	10.0000	1.019900e1
STD_50ppb	Standard	2.89e7	4.108e6	4.18	38.6	50.0000	5.337298e1
STD_100ppb	Standard	5.05e7	4.042e6	4.19	47.0	100.0000	9.478427e1
STD_200ppb	Standard	9.57e7	3.907e6	4.18	38.0	200.0000	1.857810e2
STD_500ppb	Standard	1.77e8	3.576e6	4.18	30.4	500.0000	3.751101e2
V2.0_MW_RQC1_20240724	Quality Control	N/A	2.523e6	N/A	N/A	0.0000	N/A
Blank	Unknown	N/A	9.419e2	N/A	N/A	N/A	N/A
V3.0_MWMS_20240725_1	Unknown	6.42e6	6.790e6	4.18	31.8	N/A	7.165071e0
MWXS243064a_R1	Quality Control	N/A	3.675e6	N/A	N/A	0.0000	N/A
MWXS243064a_R2	Quality Control	N/A	3.717e6	N/A	N/A	0.0000	N/A
MWXS243064a_R3	Quality Control	N/A	3.772e6	N/A	N/A	0.0000	N/A
T24186682b_a	Unknown	N/A	3.706e6	N/A	N/A	N/A	N/A
T24186682b_b	Unknown	N/A	3.576e6	N/A	N/A	N/A	N/A
T24186682b_c	Unknown	N/A	3.670e6	N/A	N/A	N/A	N/A
T24186683b_a	Unknown	N/A	4.075e6	N/A	N/A	N/A	N/A
T24186683b_b	Unknown	N/A	4.132e6	N/A	N/A	N/A	N/A
T24186683b_c	Unknown	N/A	4.123e6	N/A	N/A	N/A	N/A
T24186684b_a	Unknown	N/A	3.376e6	N/A	N/A	N/A	N/A
T24186684b_b	Unknown	N/A	3.611e6	N/A	N/A	N/A	N/A
T24186684b_c	Unknown	N/A	3.357e6	N/A	N/A	N/A	N/A

Compound name: oT
Regression Equation: $y = 0.13189 x + 3.11797e-4$ ($r = 0.99334$) (weighting: $1 / x^2$)

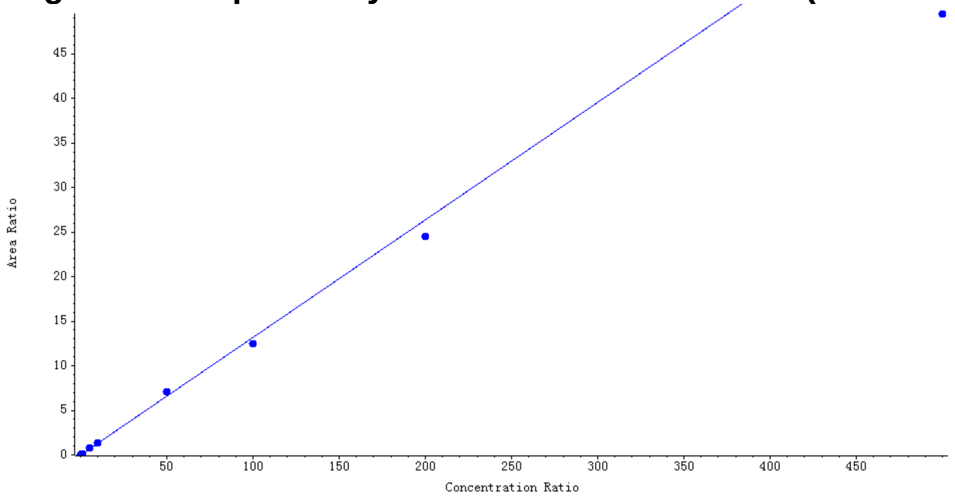

Peak Review

Blank
oT AREA:N/A S/N:N/A

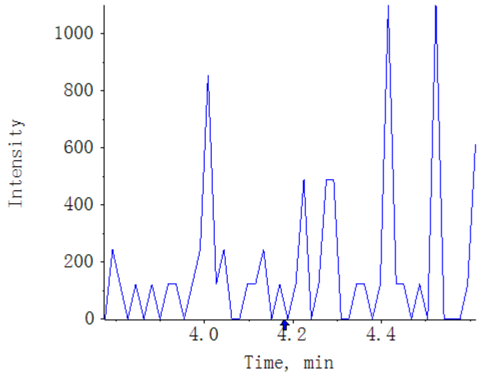

V3.0_MWMS_20240725_1
oT AREA:6.42e6 S/N:31.8

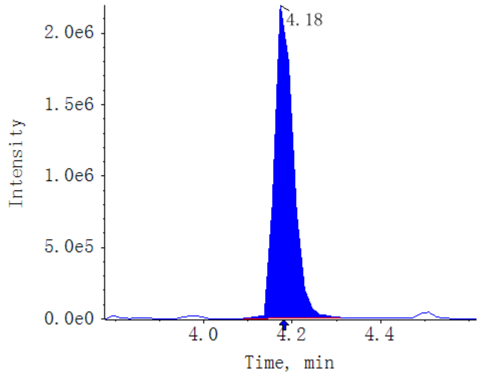

T24186682b_a
oT AREA:N/A S/N:N/A

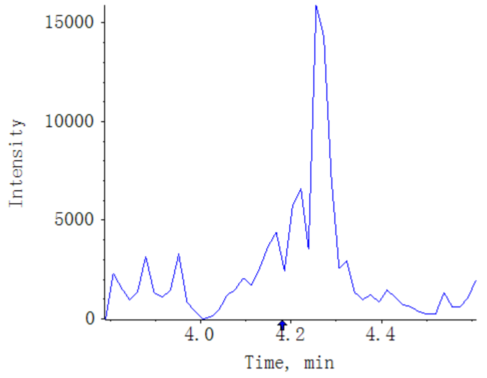

T24186682b_b
oT AREA:N/A S/N:N/A

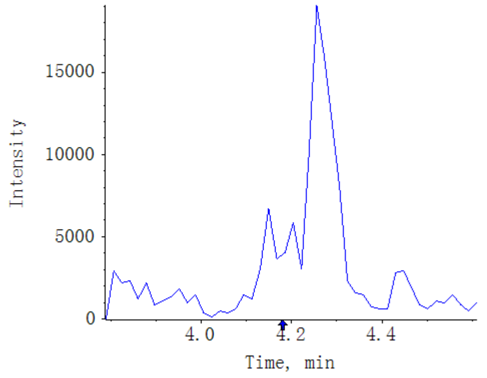

T24186682b_c
oT AREA:N/A S/N:N/A

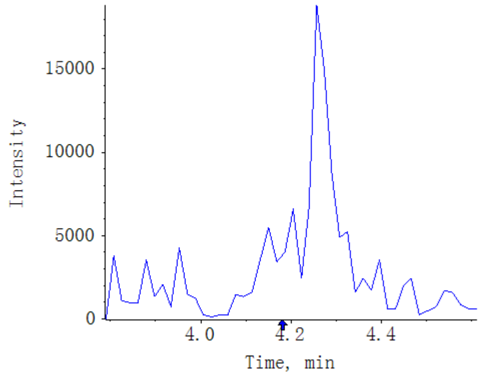

T24186683b_a
oT AREA:N/A S/N:N/A

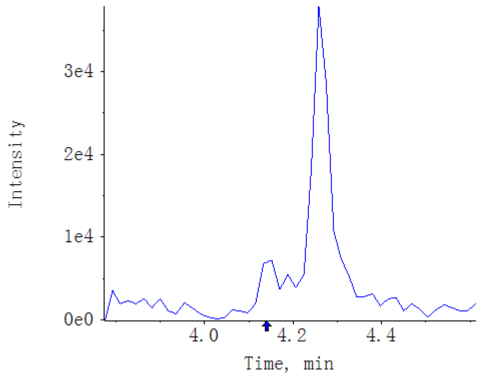

T24186683b_b
oT AREA:N/A S/N:N/A

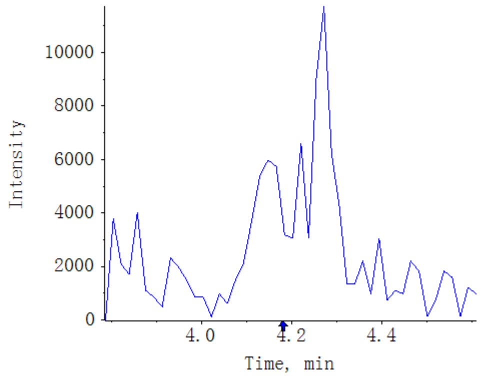

T24186683b_c
oT AREA:N/A S/N:N/A

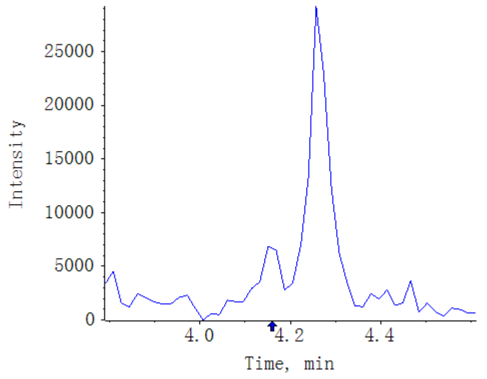

T24186684b_a
oT AREA:N/A S/N:N/A

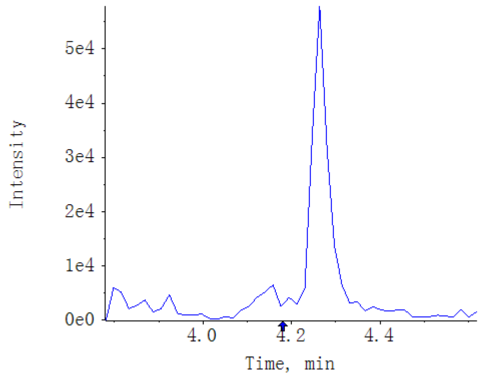

T24186684b_b
oT AREA:N/A S/N:N/A

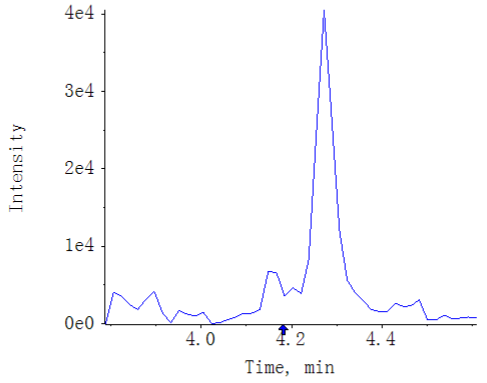

T24186684b_c
oT AREA:N/A S/N:N/A

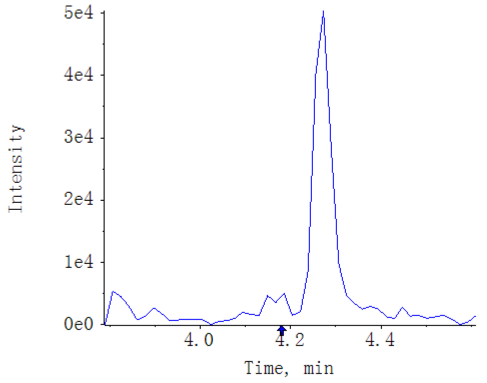

Result Table	MWXS-24-3064-a_9_WH6500-17_A20-3_V6.0_WSS_20240730	Algorithm Used	MQ4
Acquisition Method	ACC-PHs_V6.0_WH6500-17_CMY_20240521.dam	Instrument Name	Triple Quad 6500+ Low Mass
Project	N/A	Analytes QTY	109:30

Compound name: BAP (226.1 / 91.1)

Sample Name	Sample Type	Area (cps)	Is Area (cps)	RT (min)	S/N	Target Conc	Calculated Conc.()
STD_0.01ppb	Standard	1.61e4	5.126e6	4.35	13.1	0.0100	1.012085e-2
STD_0.05ppb	Standard	4.50e4	5.373e6	4.35	17.4	0.0500	4.740270e-2
STD_0.1ppb	Standard	8.04e4	5.288e6	4.37	29.6	0.1000	9.614653e-2
STD_0.5ppb	Standard	3.81e5	4.766e6	4.35	33.3	0.5000	5.577289e-1
STD_1ppb	Standard	6.63e5	4.828e6	4.35	37.8	1.0000	9.677559e-1
STD_5ppb	Standard	3.48e6	4.592e6	4.37	39.6	5.0000	5.392187e0
STD_10ppb	Standard	6.23e6	4.488e6	4.35	38.7	10.0000	9.901588e0
STD_50ppb	Standard	3.26e7	4.108e6	4.35	42.4	50.0000	5.665278e1
STD_100ppb	Standard	5.76e7	4.042e6	4.36	42.8	100.0000	1.017634e2
STD_200ppb	Standard	1.10e8	3.907e6	4.35	36.7	200.0000	2.003125e2
STD_500ppb	Standard	1.94e8	3.576e6	4.35	26.9	500.0000	3.871668e2
V2.0_MW_RQC1_20240724	Quality Control	N/A	2.523e6	N/A	N/A	0.0000	N/A
Blank	Unknown	N/A	9.419e2	N/A	N/A	N/A	N/A
V3.0_MWMS_20240725_1	Unknown	5.92e6	6.790e6	4.35	34.9	N/A	6.211822e0
MWXS243064a_R1	Quality Control	N/A	3.675e6	N/A	N/A	0.0000	N/A
MWXS243064a_R2	Quality Control	N/A	3.717e6	N/A	N/A	0.0000	N/A
MWXS243064a_R3	Quality Control	N/A	3.772e6	N/A	N/A	0.0000	N/A
T24186682b_a	Unknown	N/A	3.706e6	N/A	N/A	N/A	N/A
T24186682b_b	Unknown	N/A	3.576e6	N/A	N/A	N/A	N/A
T24186682b_c	Unknown	N/A	3.670e6	N/A	N/A	N/A	N/A
T24186683b_a	Unknown	N/A	4.075e6	N/A	N/A	N/A	N/A
T24186683b_b	Unknown	N/A	4.132e6	N/A	N/A	N/A	N/A
T24186683b_c	Unknown	N/A	4.123e6	N/A	N/A	N/A	N/A
T24186684b_a	Unknown	N/A	3.376e6	N/A	N/A	N/A	N/A
T24186684b_b	Unknown	N/A	3.611e6	N/A	N/A	N/A	N/A
T24186684b_c	Unknown	N/A	3.357e6	N/A	N/A	N/A	N/A

Compound name: BAP
Regression Equation: y = 0.14011 x + 0.00173 (r = 0.99499) (weighting: 1 / x^2)

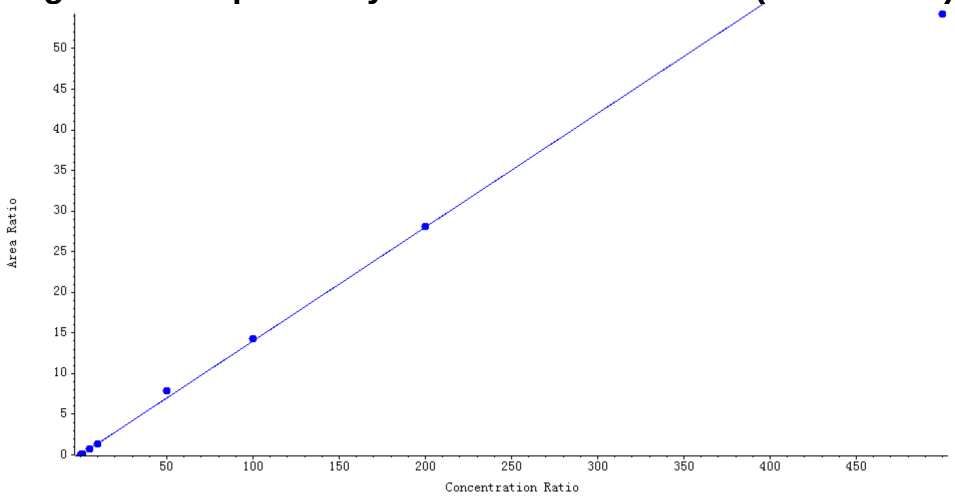

Peak Review

Blank

BAP AREA:N/A S/N:N/A

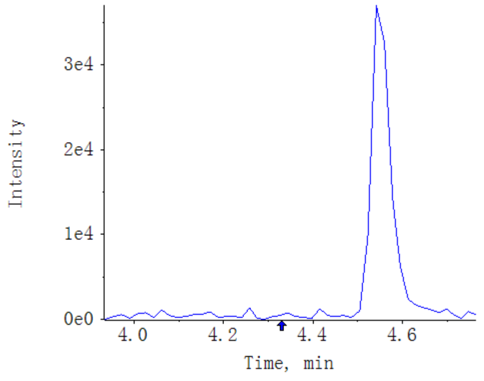

V3.0_MWMS_20240725_1

BAP AREA:5.92e6 S/N:34.9

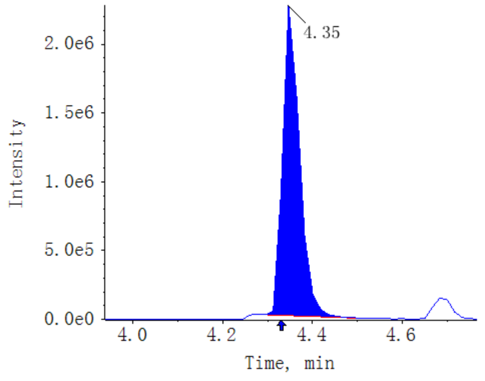

T24186682b_a

BAP AREA:N/A S/N:N/A

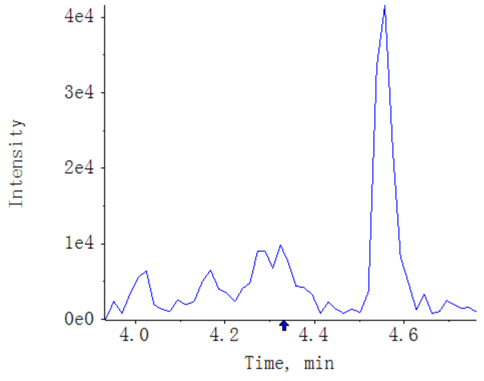

T24186682b_b

BAP AREA:N/A S/N:N/A

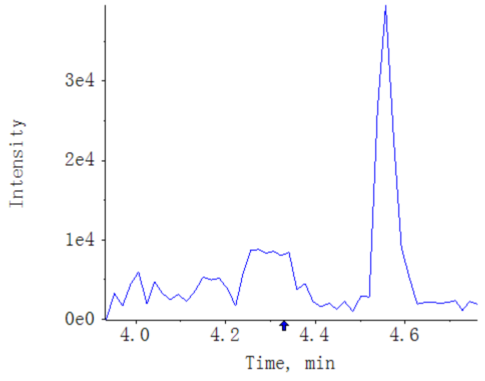

T24186682b_c

BAP AREA:N/A S/N:N/A

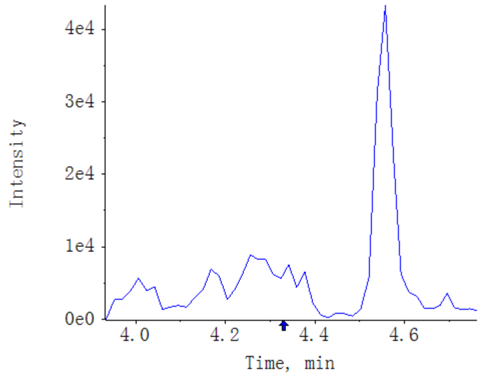

T24186683b_a

BAP AREA:N/A S/N:N/A

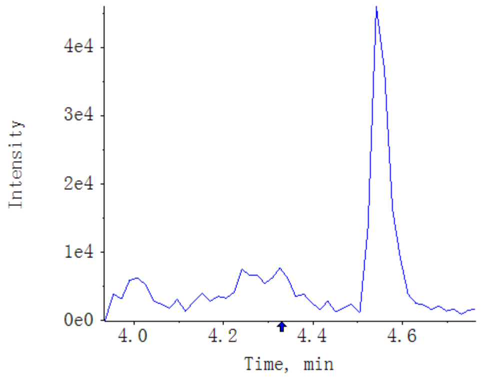

T24186683b_b

BAP AREA:N/A S/N:N/A

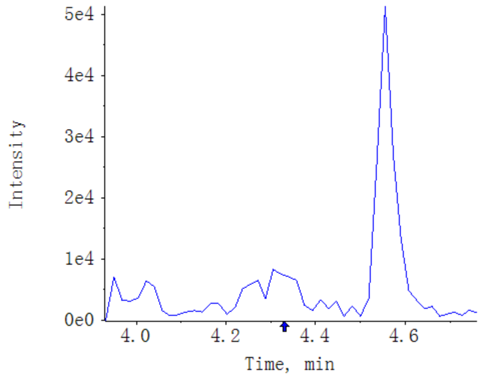

T24186683b_c

BAP AREA:N/A S/N:N/A

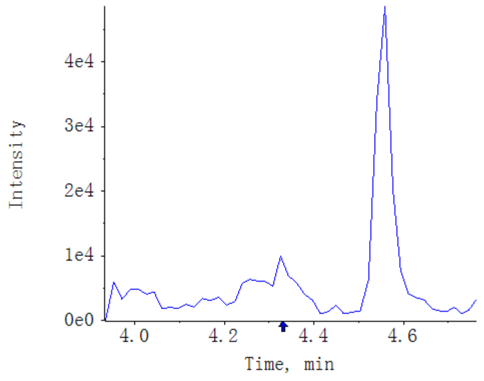

T24186684b_a

BAP AREA:N/A S/N:N/A

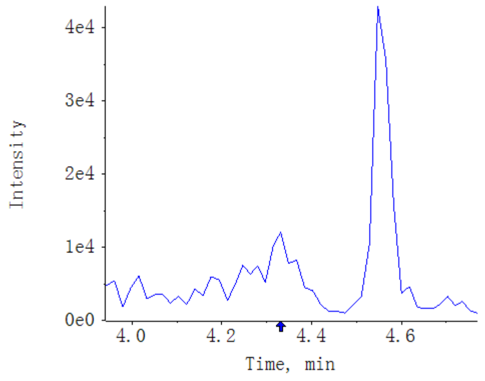

T24186684b_b

BAP AREA:N/A S/N:N/A

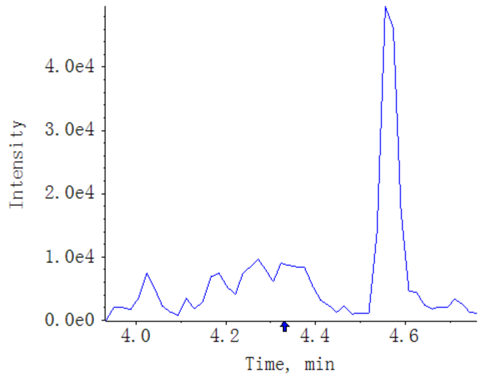

T24186684b_c

BAP AREA:N/A S/N:N/A

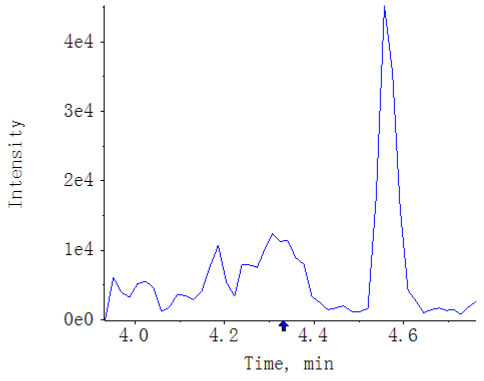

Result Table	MWXS-24-3064-a_9_WH6500-17_A20-3_V6.0_WSS_20240730	Algorithm Used	MQ4
Acquisition Method	ACC-PHs_V6.0_WH6500-17_CMY_20240521.dam	Instrument Name	Triple Quad 6500+ Low Mass
Project	N/A	Analytes QTY	109:31

Compound name: BAPR (358.1 / 226.1)

Sample Name	Sample Type	Area (cps)	Is Area (cps)	RT (min)	S/N	Target Conc	Calculated Conc.()
STD_0.01ppb	Standard	8.10e3	3.065e6	4.70	13.9	0.0100	9.534348e-3
STD_0.05ppb	Standard	7.53e4	3.030e6	4.69	44.4	0.0500	5.913101e-2
STD_0.1ppb	Standard	1.41e5	3.037e6	4.71	46.2	0.1000	1.075097e-1
STD_0.5ppb	Standard	6.72e5	2.659e6	4.70	47.2	0.5000	5.683037e-1
STD_1ppb	Standard	1.21e6	2.814e6	4.70	54.8	1.0000	9.604596e-1
STD_5ppb	Standard	6.89e6	2.801e6	4.71	56.7	5.0000	5.493056e0
STD_10ppb	Standard	1.21e7	2.618e6	4.70	65.0	10.0000	1.031881e1
STD_50ppb	Standard	5.19e7	2.412e6	4.69	51.9	50.0000	4.806357e1
STD_100ppb	Standard	8.69e7	2.237e6	4.71	65.5	100.0000	8.669481e1
STD_200ppb	Standard	1.38e8	2.096e6	4.70	36.0	200.0000	1.466139e2
STD_500ppb	Standard	N/A	1.785e6	N/A	N/A	500.0000	N/A
V2.0_MW_RQC1_20240724	Quality Control	7.45e3	1.511e6	4.62	5.8	0.0000	1.463459e-2
Blank	Unknown	N/A	9.253e2	N/A	N/A	N/A	N/A
V3.0_MWMS_20240725_1	Unknown	9.94e6	4.177e6	4.69	67.6	N/A	5.318035e0
MWXS243064a_R1	Quality Control	N/A	1.316e6	N/A	N/A	0.0000	N/A
MWXS243064a_R2	Quality Control	N/A	1.226e6	N/A	N/A	0.0000	N/A
MWXS243064a_R3	Quality Control	N/A	1.274e6	N/A	N/A	0.0000	N/A
T24186682b_a	Unknown	N/A	1.337e6	N/A	N/A	N/A	N/A
T24186682b_b	Unknown	N/A	1.303e6	N/A	N/A	N/A	N/A
T24186682b_c	Unknown	N/A	1.287e6	N/A	N/A	N/A	N/A
T24186683b_a	Unknown	N/A	1.219e6	N/A	N/A	N/A	N/A
T24186683b_b	Unknown	N/A	1.264e6	N/A	N/A	N/A	N/A
T24186683b_c	Unknown	N/A	1.317e6	N/A	N/A	N/A	N/A
T24186684b_a	Unknown	N/A	1.242e6	N/A	N/A	N/A	N/A
T24186684b_b	Unknown	N/A	1.186e6	N/A	N/A	N/A	N/A
T24186684b_c	Unknown	N/A	1.175e6	N/A	N/A	N/A	N/A

Compound name: BAPR
Regression Equation: y = 0.44794 x + -0.00163 (r = 0.99036) (weighting: 1 / x^2)

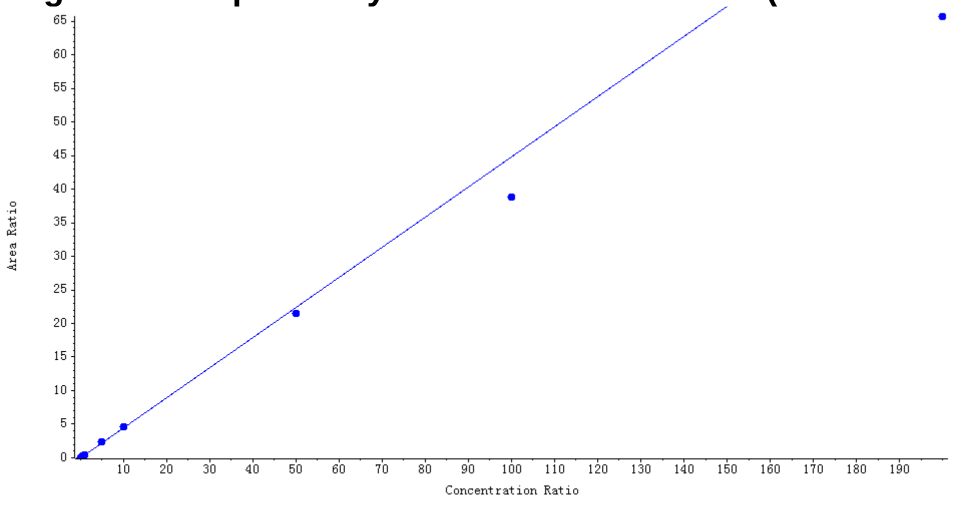

Peak Review

Blank
BAPR AREA:N/A S/N:N/A

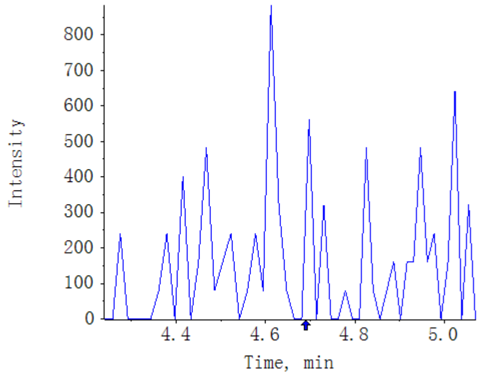

V3.0_MWMS_20240725_1
BAPR AREA:9.94e6 S/N:67.6

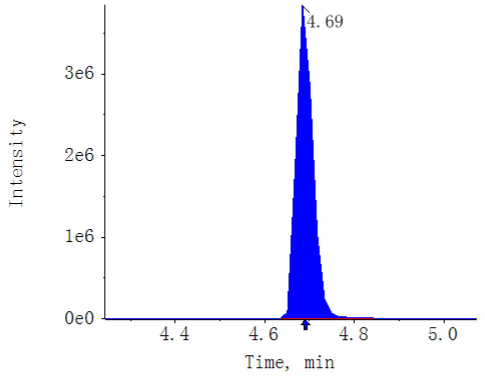

T24186682b_a
BAPR AREA:N/A S/N:N/A

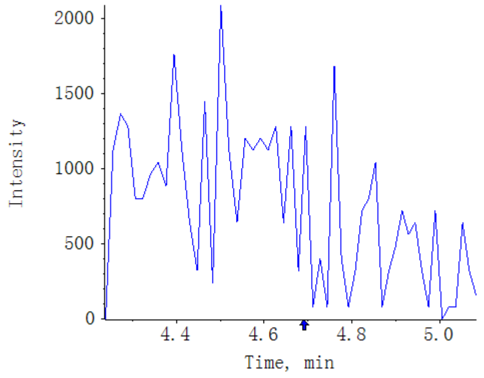

T24186682b_b
BAPR AREA:N/A S/N:N/A

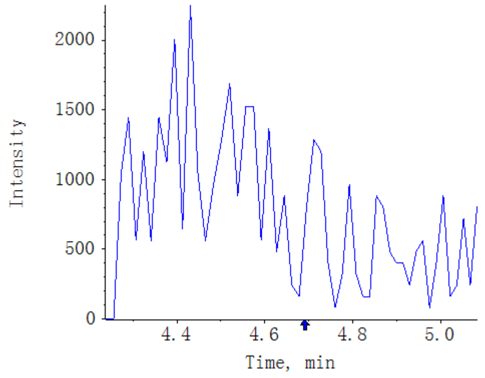

T24186682b_c
BAPR AREA:N/A S/N:N/A

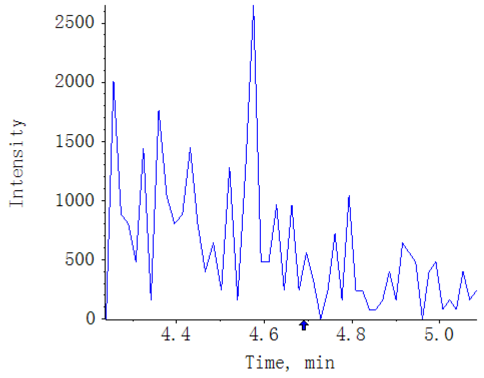

T24186683b_a
BAPR AREA:N/A S/N:N/A

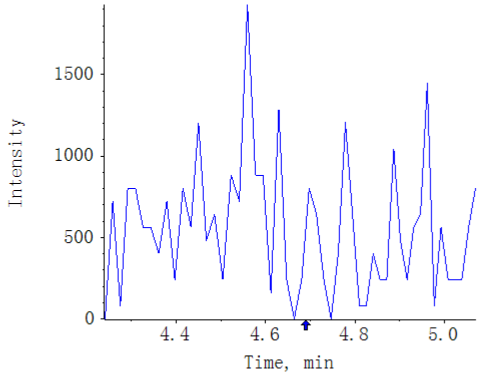

T24186683b_b
BAPR AREA:N/A S/N:N/A

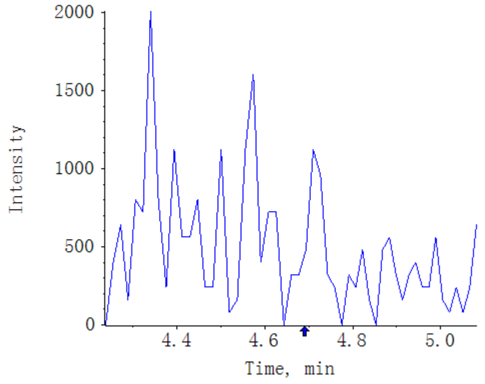

T24186683b_c
BAPR AREA:N/A S/N:N/A

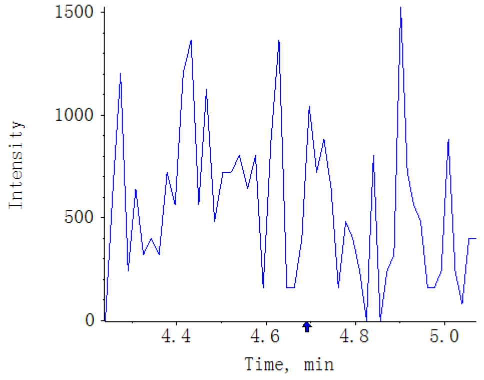

T24186684b_a
BAPR AREA:N/A S/N:N/A

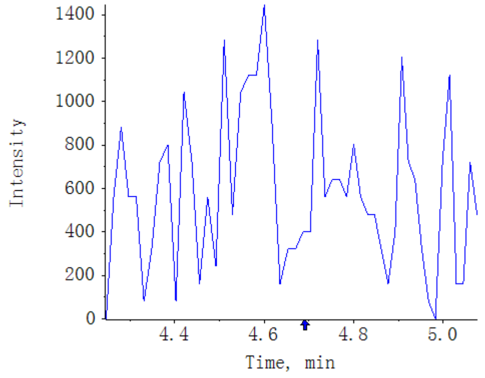

T24186684b_b
BAPR AREA:N/A S/N:N/A

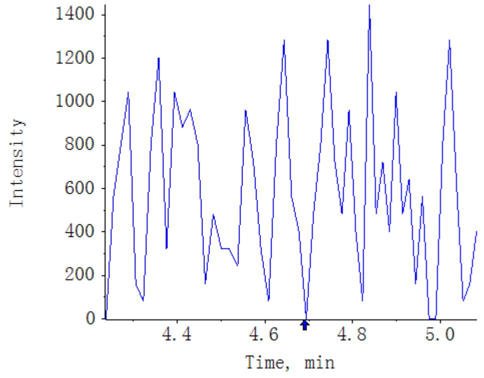

T24186684b_c
BAPR AREA:N/A S/N:N/A

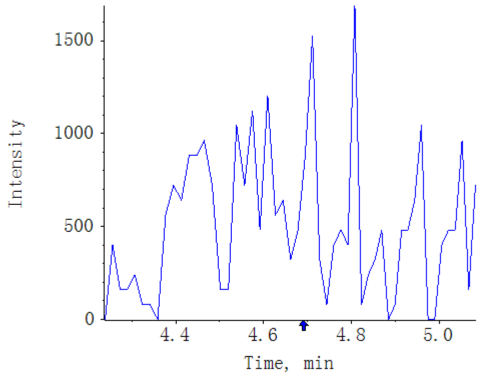

Result Table	MWXS-24-3064-a_9_WH6500-17_A20-3_V6.0_WSS_20240730	Algorithm Used	MQ4
Acquisition Method	ACC-PHs_V6.0_WH6500-17_CMY_20240521.dam	Instrument Name	Triple Quad 6500+ Low Mass
Project	N/A	Analytes QTY	109:32

Compound name: K (216.0 / 81.0)

Sample Name	Sample Type	Area (cps)	Is Area (cps)	RT (min)	S/N	Target Conc	Calculated Conc.()
STD_0.01ppb	Standard	6.76e3	5.126e6	3.82	8.5	0.0100	9.733970e-3
STD_0.05ppb	Standard	2.66e4	5.373e6	3.83	19.8	0.0500	5.611316e-2
STD_0.1ppb	Standard	4.41e4	5.288e6	3.83	26.1	0.1000	9.920024e-2
STD_0.5ppb	Standard	2.16e5	4.766e6	3.82	41.9	0.5000	5.706165e-1
STD_1ppb	Standard	3.81e5	4.828e6	3.82	46.4	1.0000	9.986092e-1
STD_5ppb	Standard	1.98e6	4.592e6	3.83	40.2	5.0000	5.492226e0
STD_10ppb	Standard	3.36e6	4.488e6	3.82	38.4	10.0000	9.532578e0
STD_50ppb	Standard	1.67e7	4.108e6	3.81	36.6	50.0000	5.171438e1
STD_100ppb	Standard	2.80e7	4.042e6	3.83	45.1	100.0000	8.823623e1
STD_200ppb	Standard	5.73e7	3.907e6	3.82	50.6	200.0000	1.867554e2
STD_500ppb	Standard	1.22e8	3.576e6	3.82	53.6	500.0000	4.351825e2
V2.0_MW_RQC1_20240724	Quality Control	N/A	2.523e6	N/A	N/A	0.0000	N/A
Blank	Unknown	N/A	9.419e2	N/A	N/A	N/A	N/A
V3.0_MWMS_20240725_1	Unknown	3.29e6	6.790e6	3.83	39.5	N/A	6.172991e0
MWXS243064a_R1	Quality Control	N/A	3.675e6	N/A	N/A	0.0000	N/A
MWXS243064a_R2	Quality Control	N/A	3.717e6	N/A	N/A	0.0000	N/A
MWXS243064a_R3	Quality Control	N/A	3.772e6	N/A	N/A	0.0000	N/A
T24186682b_a	Unknown	N/A	3.706e6	N/A	N/A	N/A	N/A
T24186682b_b	Unknown	N/A	3.576e6	N/A	N/A	N/A	N/A
T24186682b_c	Unknown	N/A	3.670e6	N/A	N/A	N/A	N/A
T24186683b_a	Unknown	N/A	4.075e6	N/A	N/A	N/A	N/A
T24186683b_b	Unknown	N/A	4.132e6	N/A	N/A	N/A	N/A
T24186683b_c	Unknown	N/A	4.123e6	N/A	N/A	N/A	N/A
T24186684b_a	Unknown	N/A	3.376e6	N/A	N/A	N/A	N/A
T24186684b_b	Unknown	N/A	3.611e6	N/A	N/A	N/A	N/A
T24186684b_c	Unknown	N/A	3.357e6	N/A	N/A	N/A	N/A

Compound name: K
Regression Equation: y = 0.07848 x + 5.54008e-4 (r = 0.99553) (weighting: 1 / x^2)

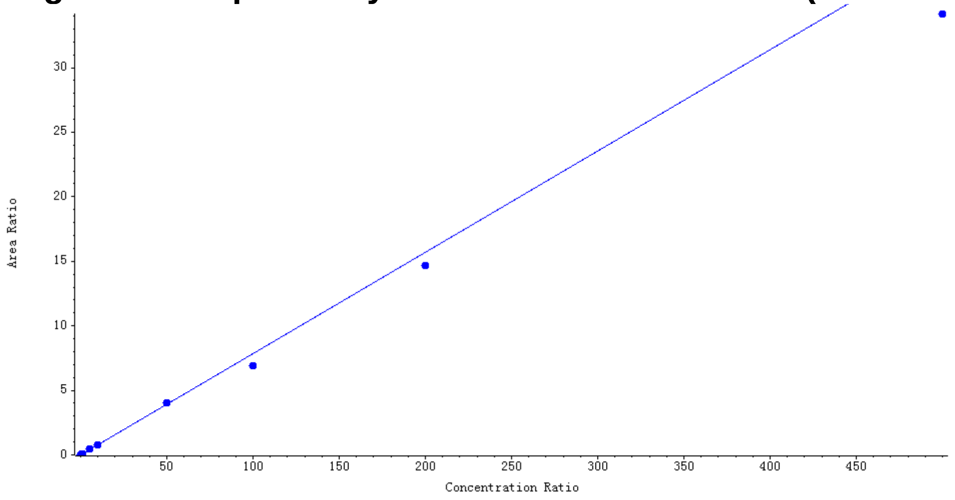

Peak Review

Blank

K AREA:N/A S/N:N/A

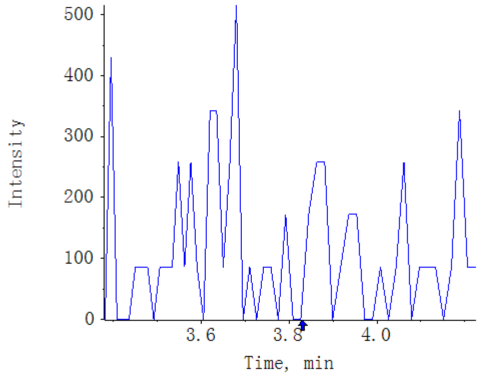

V3.0_MWMS_20240725_1

K AREA:3.29e6 S/N:39.5

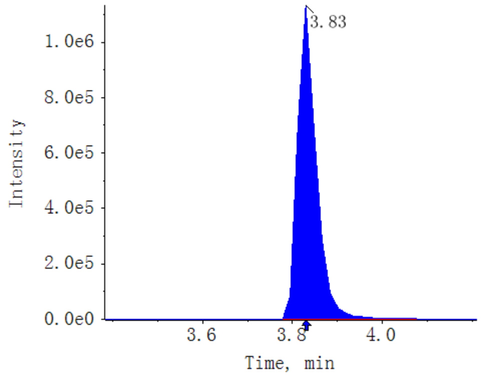

T24186682b_a

K AREA:N/A S/N:N/A

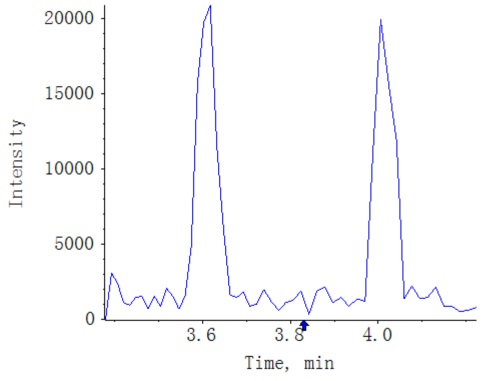

T24186682b_b

K AREA:N/A S/N:N/A

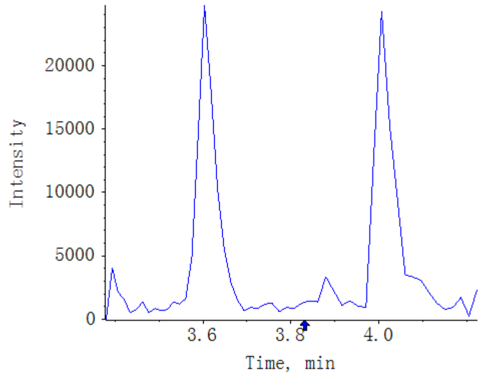

T24186682b_c

K AREA:N/A S/N:N/A

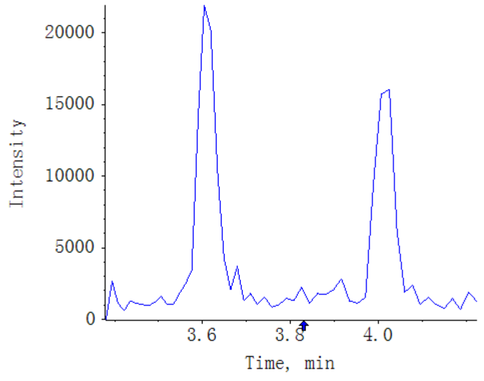

T24186683b_a

K AREA:N/A S/N:N/A

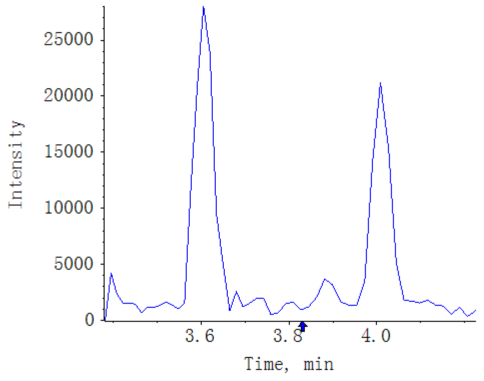

T24186683b_b

K AREA:N/A S/N:N/A

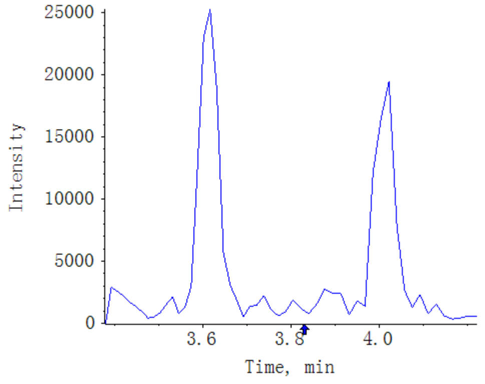

T24186683b_c

K AREA:N/A S/N:N/A

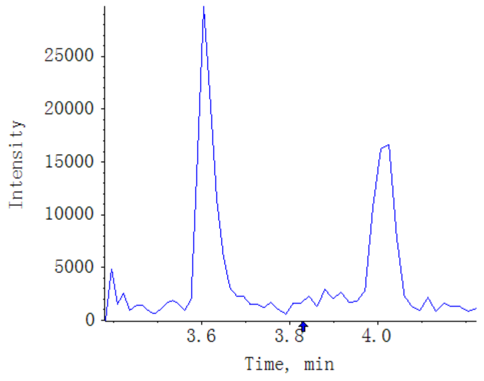

T24186684b_a

K AREA:N/A S/N:N/A

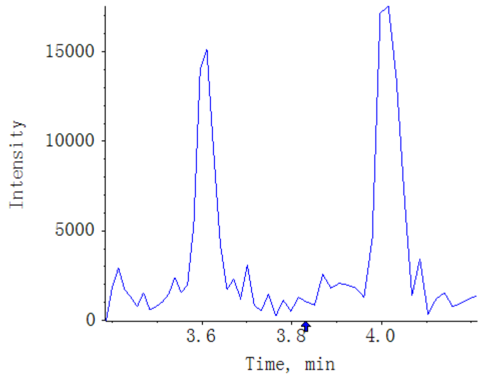

T24186684b_b

K AREA:N/A S/N:N/A

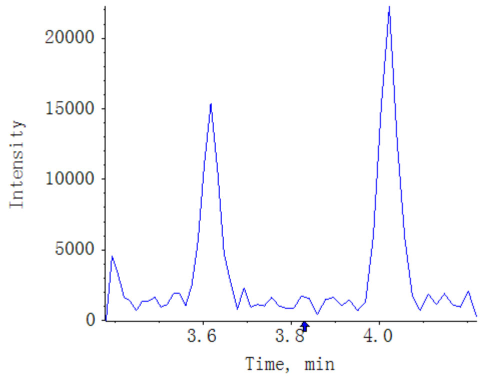

T24186684b_c

K AREA:N/A S/N:N/A

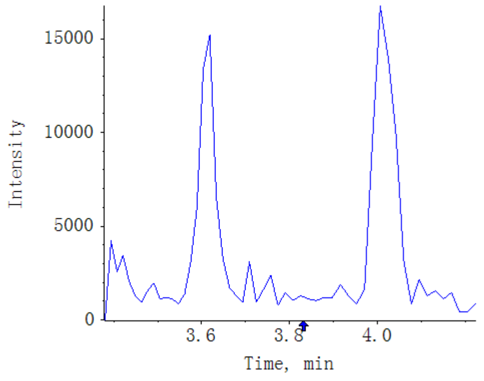

**Result Table**	MWXS-24-3064-a_9_WH6500-17_A20-3_V6.0_WSS_20240730	**Algorithm Used**	MQ4
**Acquisition Method**	ACC-PHs_V6.0_WH6500-17_CMY_20240521.dam	**Instrument Name**	Triple Quad 6500+ Low Mass
**Project**	N/A	**Analytes QTY**	109:33

Compound name: KR (348.1 / 216.2)

Sample Name	Sample Type	Area (cps)	Is Area (cps)	RT (min)	S/N	Target Conc	Calculated Conc.()
STD_0.01ppb	Standard	8.96e3	3.065e6	4.25	13.6	0.0100	9.401185e-3
STD_0.05ppb	Standard	4.95e4	3.030e6	4.26	21.9	0.0500	6.230400e-2
STD_0.1ppb	Standard	8.66e4	3.037e6	4.27	43.0	0.1000	1.103421e-1
STD_0.5ppb	Standard	3.59e5	2.659e6	4.26	68.1	0.5000	5.301192e-1
STD_1ppb	Standard	6.51e5	2.814e6	4.26	68.1	1.0000	9.092871e-1
STD_5ppb	Standard	3.65e6	2.801e6	4.27	67.7	5.0000	5.140221e0
STD_10ppb	Standard	6.60e6	2.618e6	4.26	81.4	10.0000	9.935536e0
STD_50ppb	Standard	2.96e7	2.412e6	4.25	63.7	50.0000	4.837241e1
STD_100ppb	Standard	5.11e7	2.237e6	4.26	43.0	100.0000	9.005996e1
STD_200ppb	Standard	9.05e7	2.096e6	4.26	43.9	200.0000	1.702419e2
STD_500ppb	Standard	N/A	1.785e6	N/A	N/A	500.0000	N/A
V2.0_MW_RQC1_20240724	Quality Control	N/A	1.511e6	N/A	N/A	0.0000	N/A
Blank	Unknown	N/A	9.253e2	N/A	N/A	N/A	N/A
V3.0_MWMS_20240725_1	Unknown	6.77e6	4.177e6	4.25	45.2	N/A	6.388760e0
MWXS243064a_R1	Quality Control	N/A	1.316e6	N/A	N/A	0.0000	N/A
MWXS243064a_R2	Quality Control	N/A	1.226e6	N/A	N/A	0.0000	N/A
MWXS243064a_R3	Quality Control	N/A	1.274e6	N/A	N/A	0.0000	N/A
T24186682b_a	Unknown	N/A	1.337e6	N/A	N/A	N/A	N/A
T24186682b_b	Unknown	N/A	1.303e6	N/A	N/A	N/A	N/A
T24186682b_c	Unknown	N/A	1.287e6	N/A	N/A	N/A	N/A
T24186683b_a	Unknown	N/A	1.219e6	N/A	N/A	N/A	N/A
T24186683b_b	Unknown	N/A	1.264e6	N/A	N/A	N/A	N/A
T24186683b_c	Unknown	N/A	1.317e6	N/A	N/A	N/A	N/A
T24186684b_a	Unknown	2.58e4	1.242e6	4.29	5.8	N/A	7.990657e-2
T24186684b_b	Unknown	2.21e4	1.186e6	4.30	3.6	N/A	7.126609e-2
T24186684b_c	Unknown	2.37e4	1.175e6	4.30	6.8	N/A	7.749825e-2

Compound name: KR
Regression Equation: y = 0.25370 x + 5.38996e-4 (r = 0.99282) (weighting: 1 / x^2)

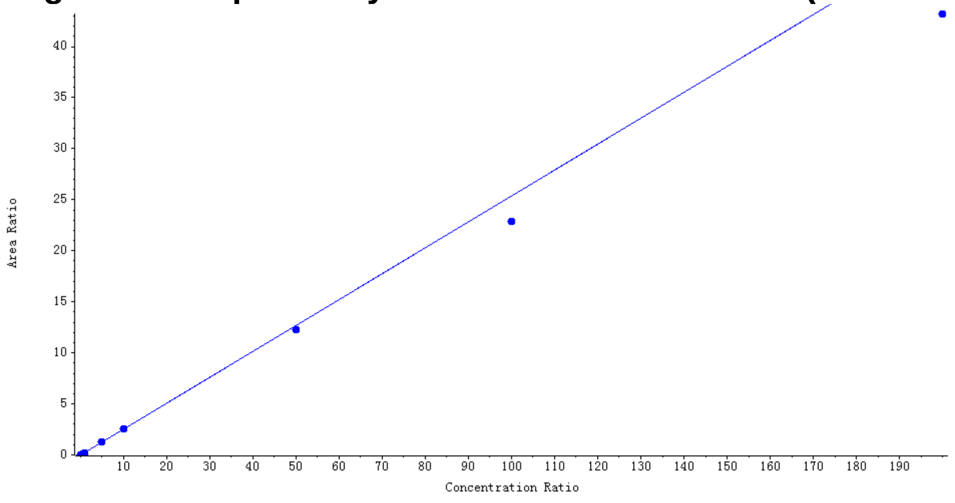

Peak Review

Blank
KR AREA:N/A S/N:N/A

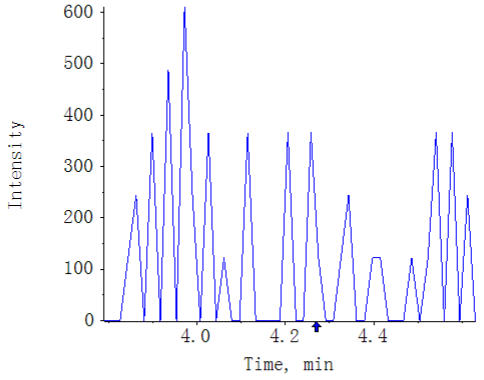

V3.0_MWMS_20240725_1
KR AREA:6.77e6 S/N:45.2

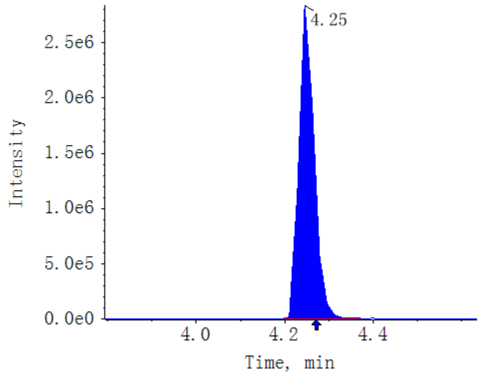

T24186682b_a
KR AREA:N/A S/N:N/A

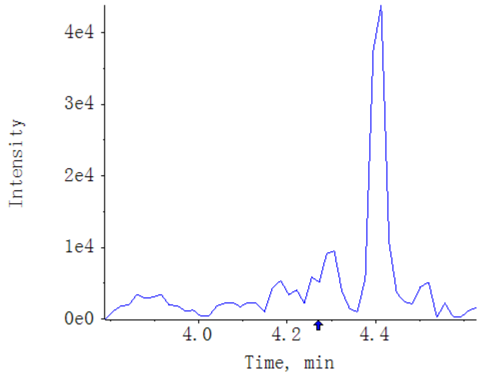

T24186682b_b
KR AREA:N/A S/N:N/A

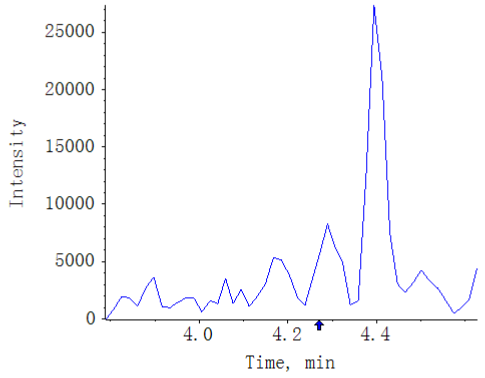

T24186682b_c
KR AREA:N/A S/N:N/A

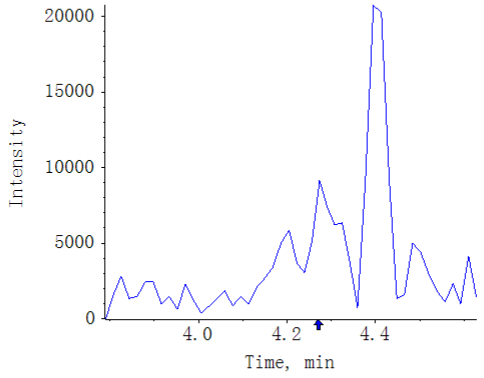

T24186683b_a
KR AREA:N/A S/N:N/A

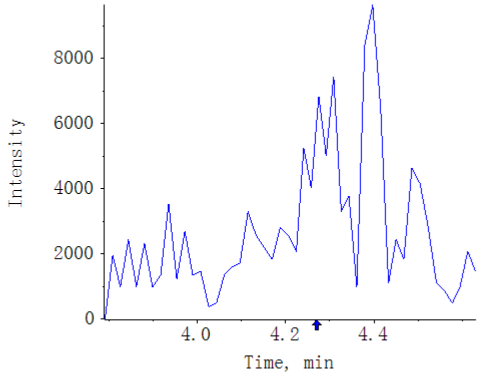

T24186683b_b
KR AREA:N/A S/N:N/A

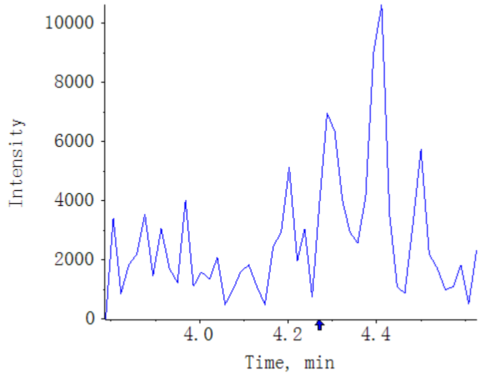

T24186683b_c
KR AREA:N/A S/N:N/A

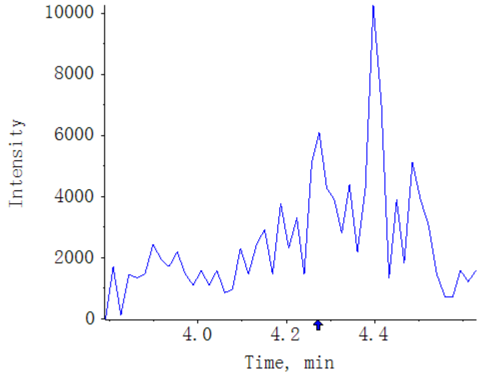

T24186684b_a
KR AREA:2.58e4 S/N:5.8

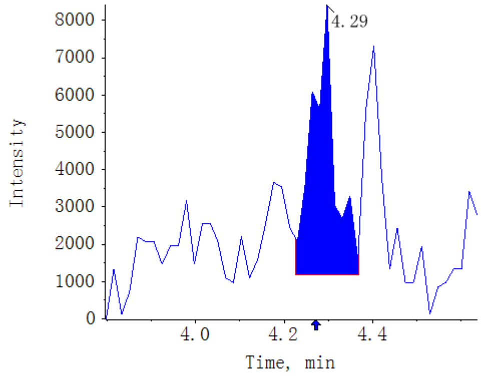

T24186684b_b
KR AREA:2.21e4 S/N:3.6

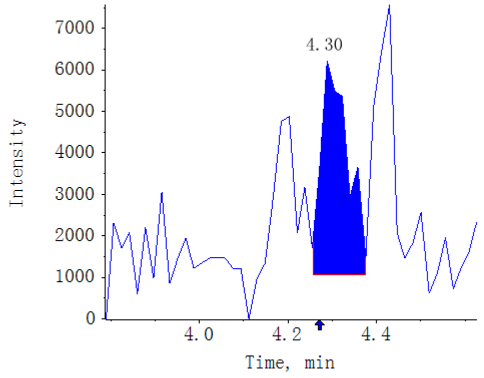

T24186684b_c
KR AREA:2.37e4 S/N:6.8

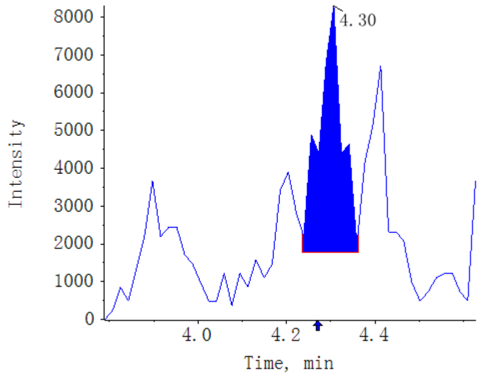

Result Table	MWXS-24-3064-a_9_WH6500-17_A20-3_V6.0_WSS_20240730	Algorithm Used	MQ4
Acquisition Method	ACC-PHs_V6.0_WH6500-17_CMY_20240521.dam	Instrument Name	Triple Quad 6500+ Low Mass
Project	N/A	Analytes QTY	109:34

Compound name: pTR (374.1 / 242.2)

Sample Name	Sample Type	Area (cps)	Is Area (cps)	RT (min)	S/N	Target Conc	Calculated Conc.()
STD_0.01ppb	Standard	N/A	3.065e6	N/A	N/A	0.0100	N/A
STD_0.05ppb	Standard	2.27e4	3.030e6	3.98	5.4	0.0500	5.054024e-2
STD_0.1ppb	Standard	4.35e4	3.037e6	3.99	6.1	0.1000	9.628480e-2
STD_0.5ppb	Standard	2.19e5	2.659e6	3.98	9.1	0.5000	5.522836e-1
STD_1ppb	Standard	4.01e5	2.814e6	3.98	9.2	1.0000	9.576345e-1
STD_5ppb	Standard	2.00e6	2.801e6	3.99	9.6	5.0000	4.797827e0
STD_10ppb	Standard	3.68e6	2.618e6	3.98	7.7	10.0000	9.438348e0
STD_50ppb	Standard	1.98e7	2.412e6	3.98	11.8	50.0000	5.520683e1
STD_100ppb	Standard	3.42e7	2.237e6	3.99	6.7	100.0000	1.027376e2
STD_200ppb	Standard	6.54e7	2.096e6	3.98	9.0	200.0000	2.094913e2
STD_500ppb	Standard	1.17e8	1.785e6	3.98	8.8	500.0000	4.408880e2
V2.0_MW_RQC1_20240724	Quality Control	N/A	1.511e6	N/A	N/A	0.0000	N/A
Blank	Unknown	N/A	9.253e2	N/A	N/A	N/A	N/A
V3.0_MWMS_20240725_1	Unknown	4.68e6	4.177e6	3.97	8.5	N/A	7.517915e0
MWXS243064a_R1	Quality Control	N/A	1.316e6	N/A	N/A	0.0000	N/A
MWXS243064a_R2	Quality Control	N/A	1.226e6	N/A	N/A	0.0000	N/A
MWXS243064a_R3	Quality Control	N/A	1.274e6	N/A	N/A	0.0000	N/A
T24186682b_a	Unknown	N/A	1.337e6	N/A	N/A	N/A	N/A
T24186682b_b	Unknown	N/A	1.303e6	N/A	N/A	N/A	N/A
T24186682b_c	Unknown	N/A	1.287e6	N/A	N/A	N/A	N/A
T24186683b_a	Unknown	N/A	1.219e6	N/A	N/A	N/A	N/A
T24186683b_b	Unknown	N/A	1.264e6	N/A	N/A	N/A	N/A
T24186683b_c	Unknown	N/A	1.317e6	N/A	N/A	N/A	N/A
T24186684b_a	Unknown	N/A	1.242e6	N/A	N/A	N/A	N/A
T24186684b_b	Unknown	N/A	1.186e6	N/A	N/A	N/A	N/A
T24186684b_c	Unknown	N/A	1.175e6	N/A	N/A	N/A	N/A

Compound name: pTR
Regression Equation: y = 0.14898 x + -2.56016e-5 (r = 0.99701) (weighting: 1 / x^2)

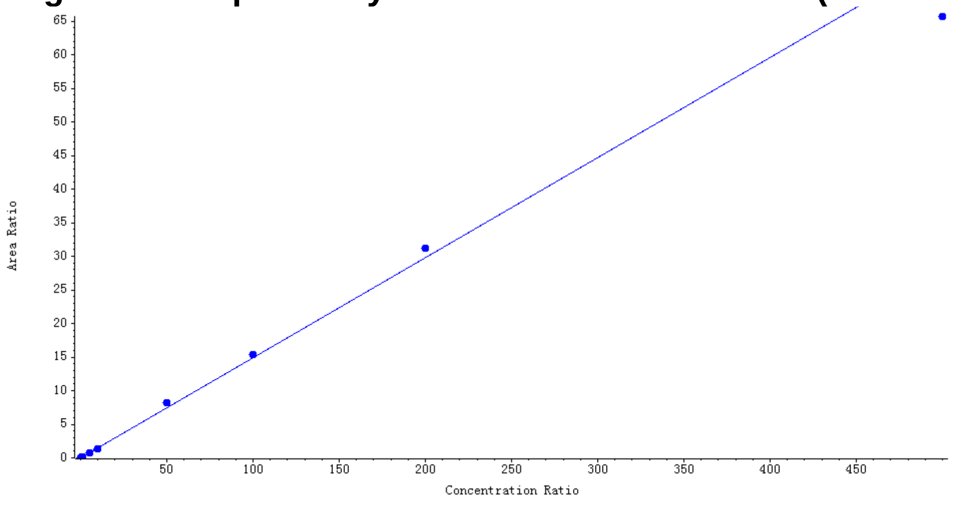

Peak Review

Blank
pTR AREA:N/A S/N:N/A

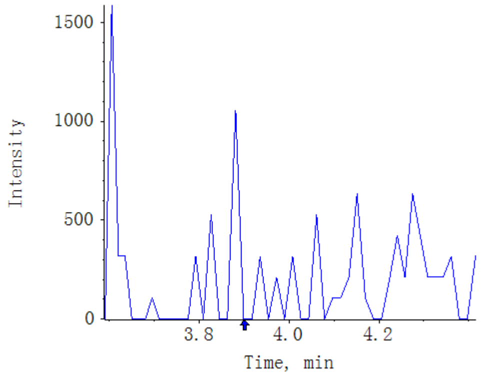

V3.0_MWMS_20240725_1
pTR AREA:4.68e6 S/N:8.5

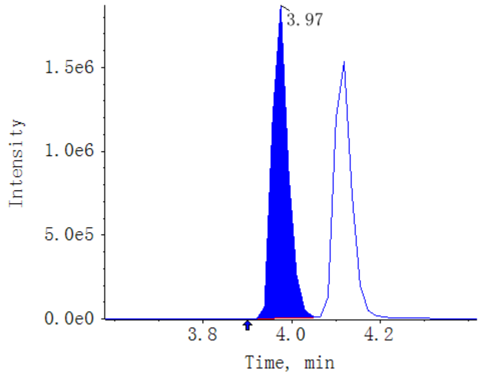

T24186682b_a
pTR AREA:N/A S/N:N/A

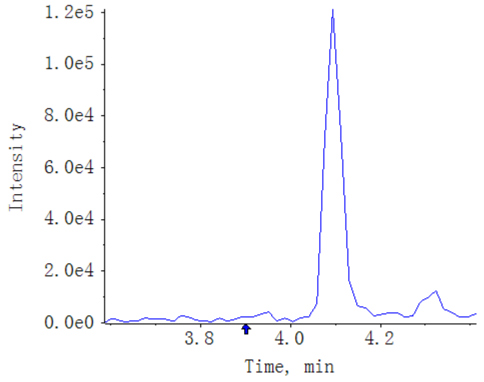

T24186682b_b
pTR AREA:N/A S/N:N/A

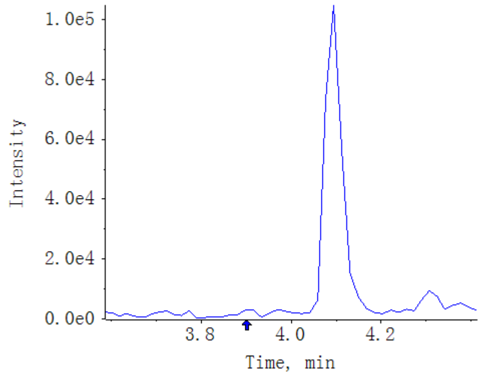

T24186682b_c
pTR AREA:N/A S/N:N/A

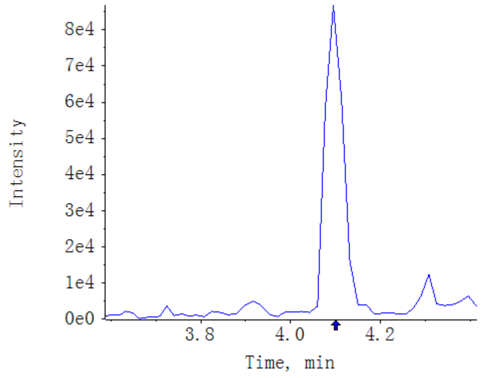

T24186683b_a
pTR AREA:N/A S/N:N/A

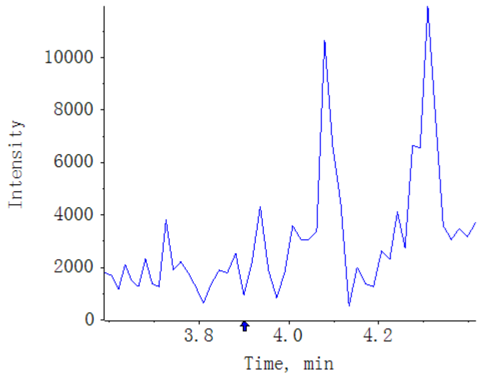

T24186683b_b
pTR AREA:N/A S/N:N/A

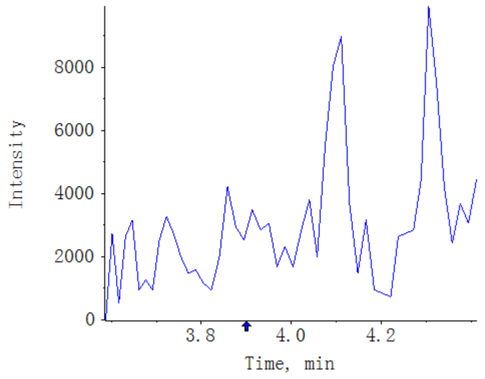

T24186683b_c
pTR AREA:N/A S/N:N/A

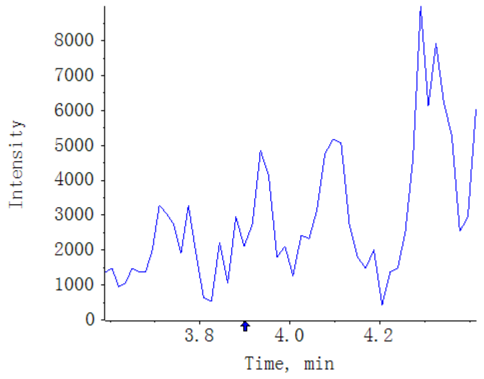

T24186684b_a
pTR AREA:N/A S/N:N/A

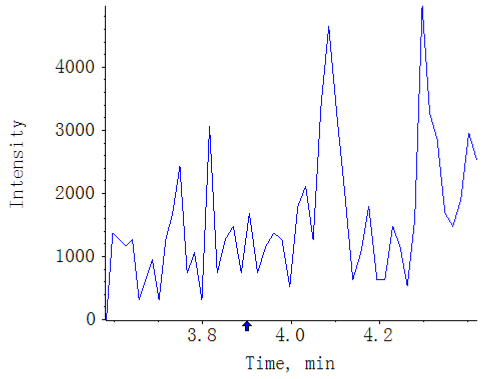

T24186684b_b
pTR AREA:N/A S/N:N/A

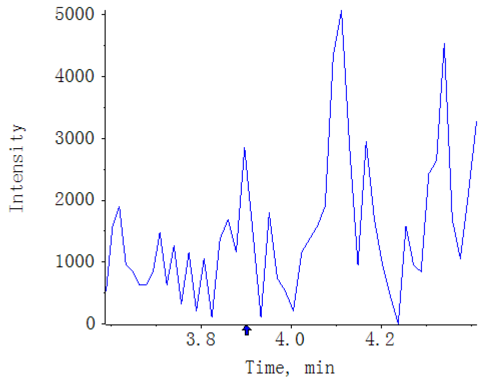

T24186684b_c
pTR AREA:N/A S/N:N/A

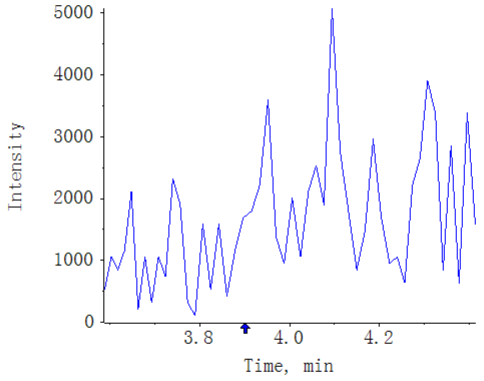

Result Table	MWXS-24-3064-a_9_WH6500-17_A20-3_V6.0_WSS_20240730	Algorithm Used	MQ4
Acquisition Method	ACC-PHs_V6.0_WH6500-17_CMY_20240521.dam	Instrument Name	Triple Quad 6500+ Low Mass
Project	N/A	Analytes QTY	109:35

Compound name: oTR (374.1 / 136.0)

Sample Name	Sample Type	Area (cps)	Is Area (cps)	RT (min)	S/N	Target Conc	Calculated Conc.()
STD_0.01ppb	Standard	6.03e3	3.065e6	4.52	10.5	0.0100	9.621164e-3
STD_0.05ppb	Standard	3.11e4	3.030e6	4.51	25.1	0.0500	5.690075e-2
STD_0.1ppb	Standard	5.98e4	3.037e6	4.52	33.6	0.1000	1.107083e-1
STD_0.5ppb	Standard	2.40e5	2.659e6	4.51	46.0	0.5000	5.135180e-1
STD_1ppb	Standard	4.45e5	2.814e6	4.51	41.4	1.0000	8.998841e-1
STD_5ppb	Standard	2.50e6	2.801e6	4.53	47.0	5.0000	5.091620e0
STD_10ppb	Standard	4.56e6	2.618e6	4.51	57.0	10.0000	9.926799e0
STD_50ppb	Standard	2.22e7	2.412e6	4.51	56.4	50.0000	5.255093e1
STD_100ppb	Standard	3.85e7	2.237e6	4.52	67.0	100.0000	9.796688e1
STD_200ppb	Standard	7.03e7	2.096e6	4.51	63.8	200.0000	1.912660e2
STD_500ppb	Standard	1.36e8	1.785e6	4.51	51.1	500.0000	4.339219e2
V2.0_MW_RQC1_20240724	Quality Control	N/A	1.511e6	N/A	N/A	0.0000	N/A
Blank	Unknown	N/A	9.253e2	N/A	N/A	N/A	N/A
V3.0_MWMS_20240725_1	Unknown	4.30e6	4.177e6	4.50	30.5	N/A	5.865438e0
MWXS243064a_R1	Quality Control	8.69e3	1.316e6	4.52	3.7	0.0000	3.605157e-2
MWXS243064a_R2	Quality Control	8.95e3	1.226e6	4.53	8.5	0.0000	4.003026e-2
MWXS243064a_R3	Quality Control	1.28e4	1.274e6	4.52	6.1	0.0000	5.578278e-2
T24186682b_a	Unknown	N/A	1.337e6	N/A	N/A	N/A	N/A
T24186682b_b	Unknown	N/A	1.303e6	N/A	N/A	N/A	N/A
T24186682b_c	Unknown	N/A	1.287e6	N/A	N/A	N/A	N/A
T24186683b_a	Unknown	1.38e4	1.219e6	4.51	7.4	N/A	6.285733e-2
T24186683b_b	Unknown	2.54e4	1.264e6	4.52	18.3	N/A	1.131057e-1
T24186683b_c	Unknown	1.51e4	1.317e6	4.52	8.5	N/A	6.368702e-2
T24186684b_a	Unknown	N/A	1.242e6	N/A	N/A	N/A	N/A
T24186684b_b	Unknown	N/A	1.186e6	N/A	N/A	N/A	N/A
T24186684b_c	Unknown	N/A	1.175e6	N/A	N/A	N/A	N/A

Compound name: oTR
Regression Equation: y = 0.17543 x + 2.80424e-4 (r = 0.99650) (weighting: 1 / x^2)

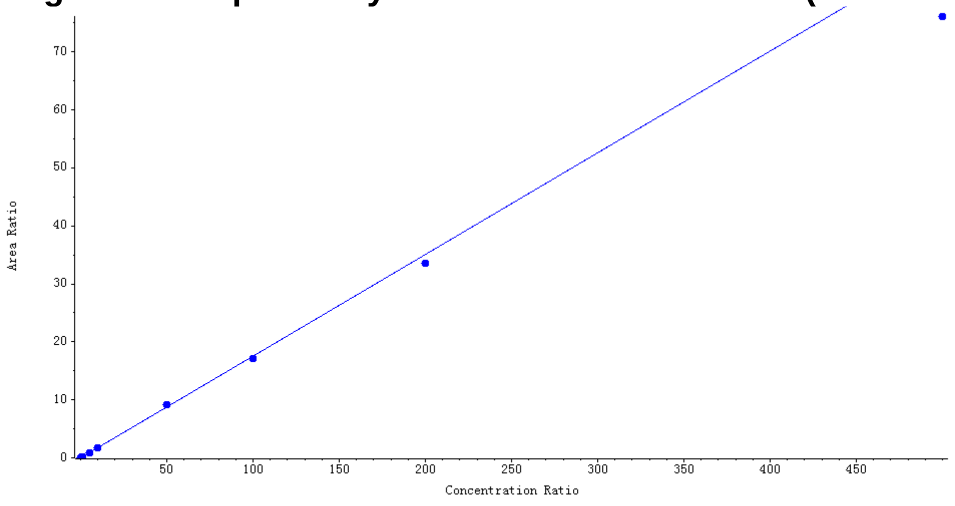

Peak Review

Blank
oTR AREA:N/A S/N:N/A

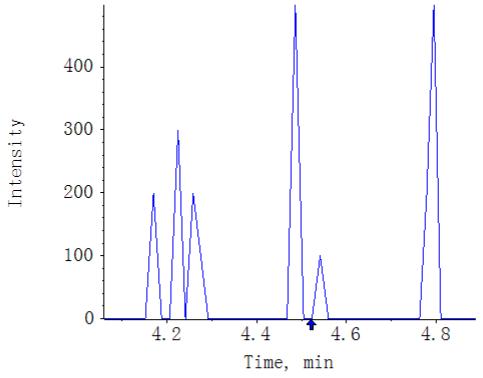

V3.0_MWMS_20240725_1
oTR AREA:4.30e6 S/N:30.5

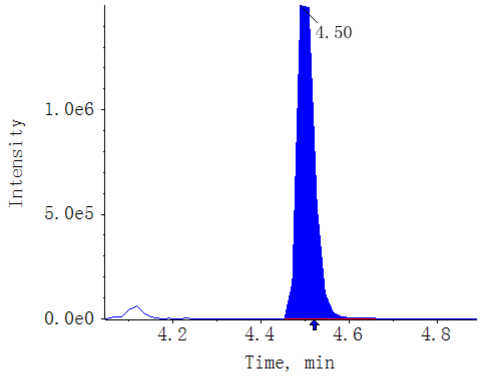

T24186682b_a
oTR AREA:N/A S/N:N/A

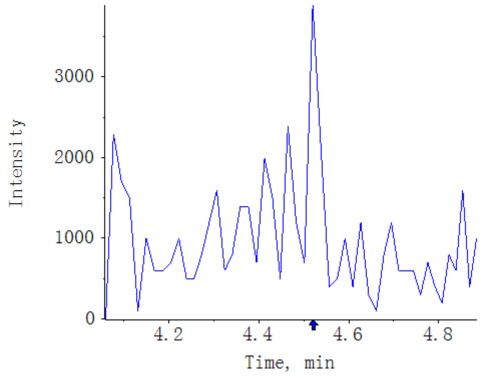

T24186682b_b
oTR AREA:N/A S/N:N/A

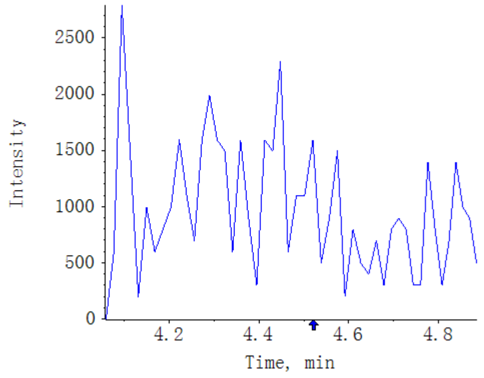

T24186682b_c
oTR AREA:N/A S/N:N/A

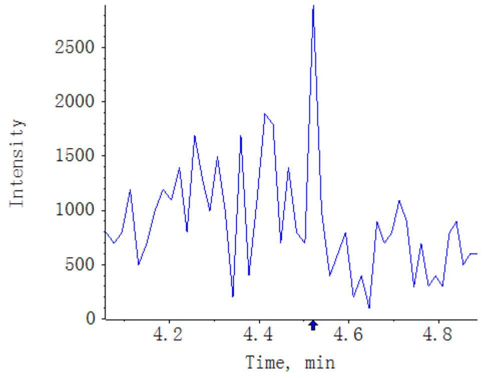

T24186683b_a
oTR AREA:1.38e4 S/N:7.4

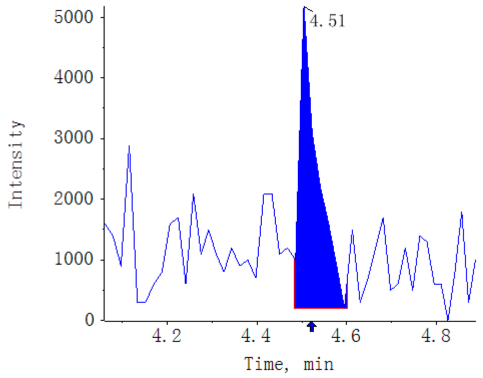

T24186683b_b
oTR AREA:2.54e4 S/N:18.3

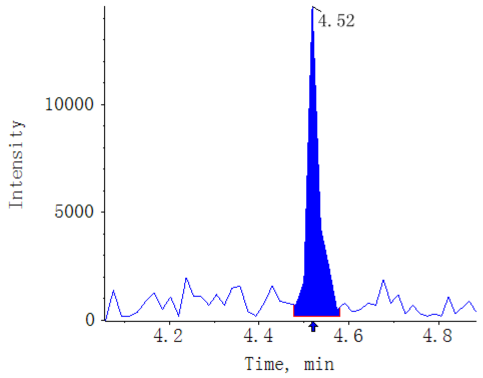

T24186683b_c
oTR AREA:1.51e4 S/N:8.5

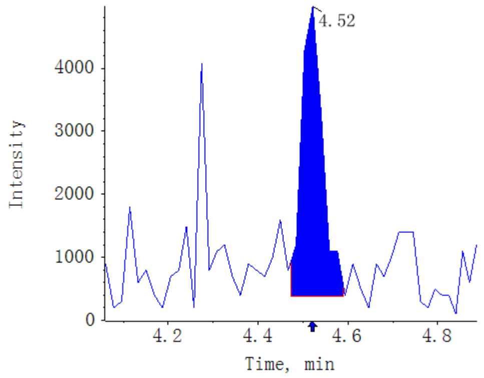

T24186684b_a
oTR AREA:N/A S/N:N/A

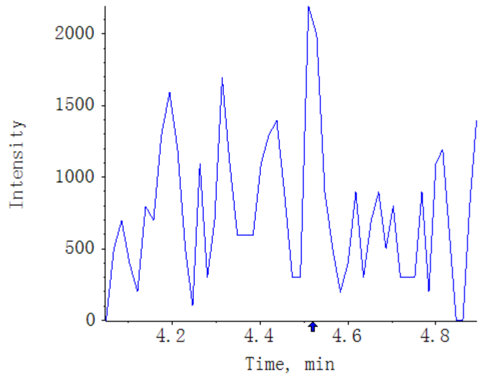

T24186684b_b
oTR AREA:N/A S/N:N/A

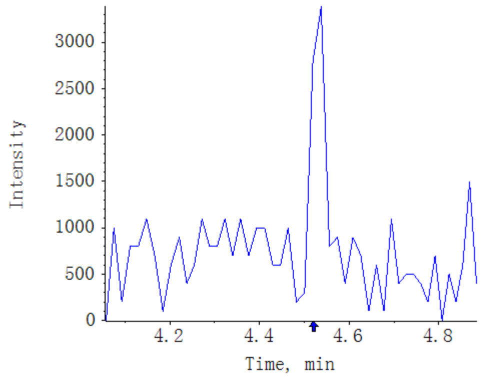

T24186684b_c
oTR AREA:N/A S/N:N/A

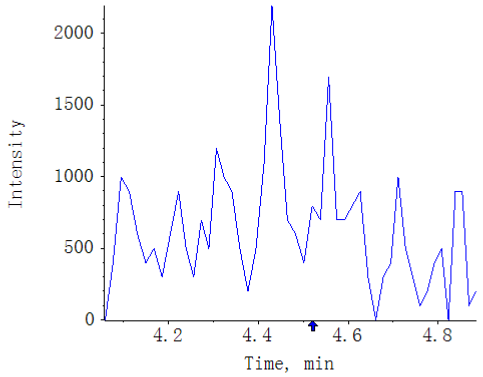

Result Table	MWXS-24-3064-a_9_WH6500-17_A20-3_V6.0_WSS_20240730	Algorithm Used	MQ4
Acquisition Method	ACC-PHs_V6.0_WH6500-17_CMY_20240521.dam	Instrument Name	Triple Quad 6500+ Low Mass
Project	N/A	Analytes QTY	109:36

Compound name: cZ9G (382.1 / 220.1)

Sample Name	Sample Type	Area (cps)	Is Area (cps)	RT (min)	S/N	Target Conc	Calculated Conc.()
STD_0.01ppb	Standard	9.79e3	2.149e6	3.23	18.9	0.0100	1.004172e-2
STD_0.05ppb	Standard	5.00e4	2.298e6	3.24	73.4	0.0500	5.125818e-2
STD_0.1ppb	Standard	8.81e4	2.309e6	3.25	129.4	0.1000	9.040868e-2
STD_0.5ppb	Standard	4.34e5	1.950e6	3.24	309.6	0.5000	5.316829e-1
STD_1ppb	Standard	7.36e5	1.990e6	3.24	409.1	1.0000	8.846600e-1
STD_5ppb	Standard	3.94e6	1.697e6	3.25	1047.8	5.0000	5.555223e0
STD_10ppb	Standard	7.04e6	1.640e6	3.24	979.1	10.0000	1.026992e1
STD_50ppb	Standard	3.97e7	1.686e6	3.24	1085.8	50.0000	5.633777e1
STD_100ppb	Standard	6.99e7	1.658e6	3.24	1153.0	100.0000	1.008714e2
STD_200ppb	Standard	1.37e8	1.623e6	3.24	1299.7	200.0000	2.023404e2
STD_500ppb	Standard	2.54e8	1.461e6	3.24	925.2	500.0000	4.166720e2
V2.0_MW_RQC1_20240724	Quality Control	5.00e6	3.581e6	3.10	475.0	0.0000	3.342747e0
Blank	Unknown	N/A	1.057e3	N/A	N/A	N/A	N/A
V3.0_MWMS_20240725_1	Unknown	8.14e6	2.638e6	3.29	1729.5	N/A	7.378196e0
MWXS243064a_R1	Quality Control	1.59e5	3.197e6	3.29	53.7	0.0000	1.179677e-1
MWXS243064a_R2	Quality Control	1.77e5	3.194e6	3.30	62.8	0.0000	1.315261e-1
MWXS243064a_R3	Quality Control	1.71e5	3.190e6	3.28	60.2	0.0000	1.277340e-1
T24186682b_a	Unknown	N/A	3.389e6	N/A	N/A	N/A	N/A
T24186682b_b	Unknown	N/A	3.111e6	N/A	N/A	N/A	N/A
T24186682b_c	Unknown	N/A	3.361e6	N/A	N/A	N/A	N/A
T24186683b_a	Unknown	5.46e5	3.077e6	3.30	163.4	N/A	4.239864e-1
T24186683b_b	Unknown	5.16e5	3.037e6	3.30	147.1	N/A	4.060576e-1
T24186683b_c	Unknown	5.03e5	3.008e6	3.30	140.1	N/A	3.992813e-1
T24186684b_a	Unknown	N/A	3.004e6	N/A	N/A	N/A	N/A
T24186684b_b	Unknown	N/A	3.134e6	N/A	N/A	N/A	N/A
T24186684b_c	Unknown	N/A	3.051e6	N/A	N/A	N/A	N/A

Compound name: cZ9G
Regression Equation: y = 0.41793 x + 3.57756e-4 (r = 0.99550) (weighting: 1 / x^2)

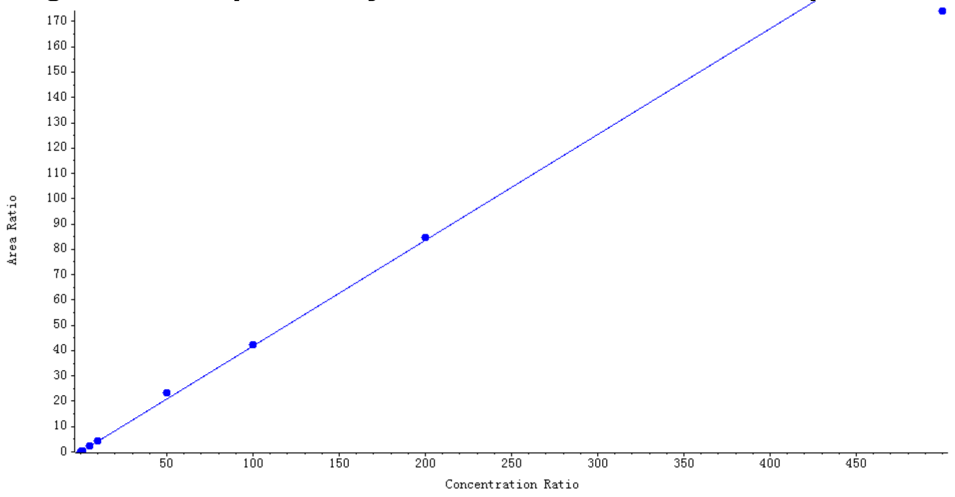

Peak Review

Blank
cZ9G AREA:N/A S/N:N/A

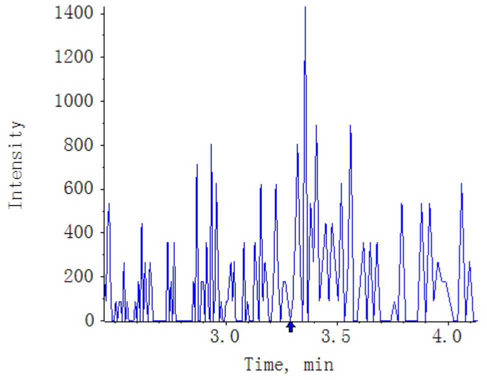

V3.0_MWMS_20240725_1
cZ9G AREA:8.14e6 S/N:1729.5

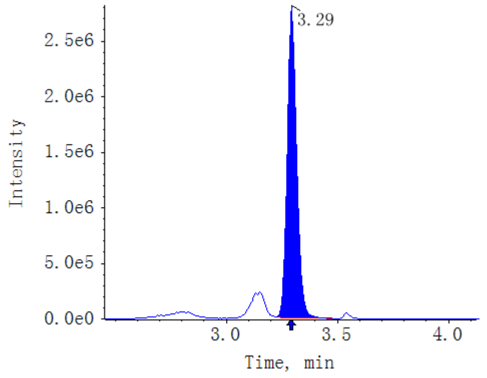

T24186682b_a
cZ9G AREA:N/A S/N:N/A

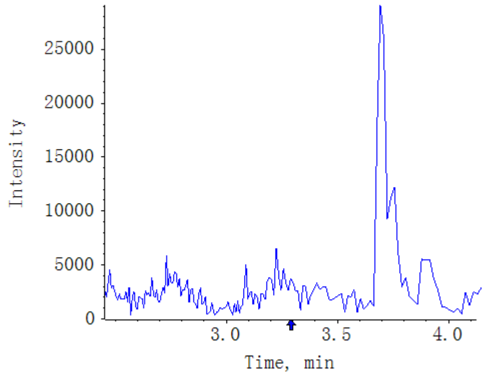

T24186682b_b
cZ9G AREA:N/A S/N:N/A

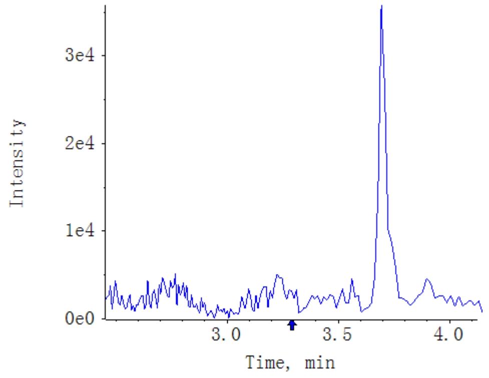

T24186682b_c
cZ9G AREA:N/A S/N:N/A

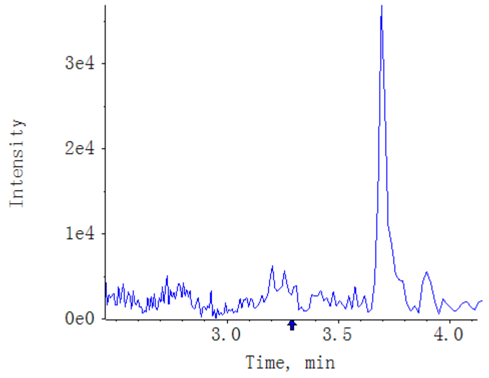

T24186683b_a
cZ9G AREA:5.46e5 S/N:163.4

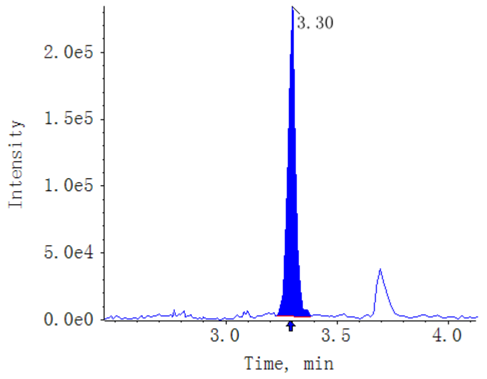

T24186683b_b
cZ9G AREA:5.16e5 S/N:147.1

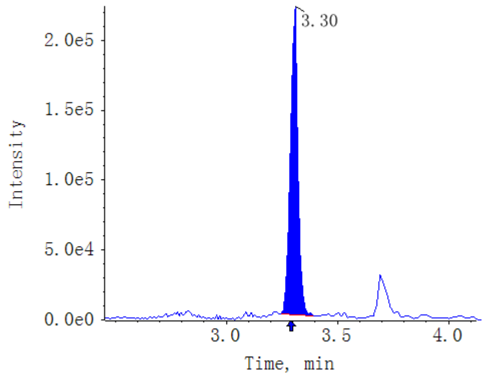

T24186683b_c
cZ9G AREA:5.03e5 S/N:140.1

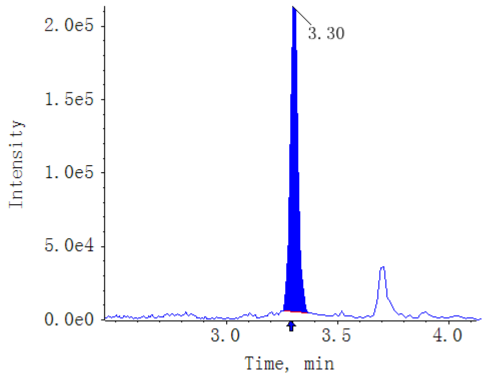

T24186684b_a
cZ9G AREA:N/A S/N:N/A

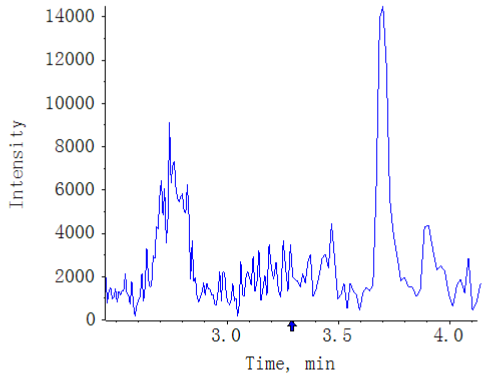

T24186684b_b
cZ9G AREA:N/A S/N:N/A

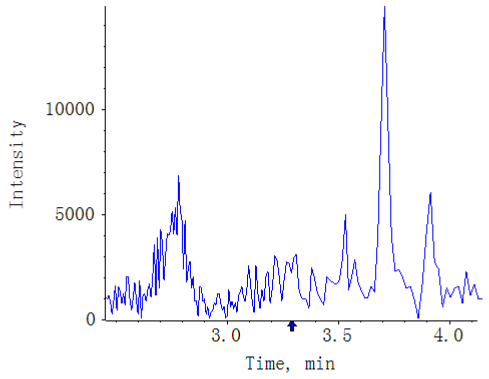

T24186684b_c
cZ9G AREA:N/A S/N:N/A

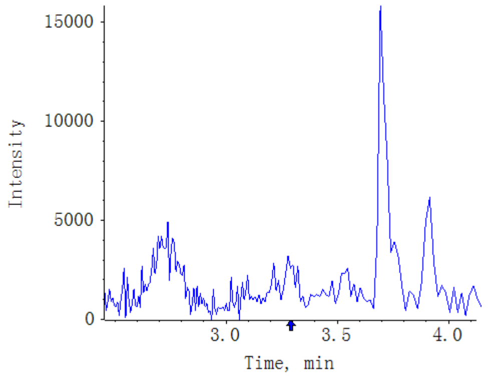

Result Table	MWXS-24-3064-a_9_WH6500-17_A20-3_V6.0_WSS_20240730	Algorithm Used	MQ4
Acquisition Method	ACC-PHs_V6.0_WH6500-17_CMY_20240521.dam	Instrument Name	Triple Quad 6500+ Low Mass
Project	N/A	Analytes QTY	109:37

Compound name: iP9G (366.1 / 204.1)

Sample Name	Sample Type	Area (cps)	Is Area (cps)	RT (min)	S/N	Target Conc	Calculated Conc.()
STD_0.01ppb	Standard	1.30e4	3.065e6	4.12	11.3	0.0100	9.873250e-3
STD_0.05ppb	Standard	4.15e4	3.030e6	4.12	7.2	0.0500	5.154403e-2
STD_0.1ppb	Standard	7.70e4	3.037e6	4.13	9.5	0.1000	1.030257e-1
STD_0.5ppb	Standard	3.68e5	2.659e6	4.12	9.3	0.5000	6.024143e-1
STD_1ppb	Standard	5.92e5	2.814e6	4.12	7.4	1.0000	9.196223e-1
STD_5ppb	Standard	3.45e6	2.801e6	4.13	6.7	5.0000	5.428211e0
STD_10ppb	Standard	5.99e6	2.618e6	4.12	8.2	10.0000	1.007323e1
STD_50ppb	Standard	2.96e7	2.412e6	4.11	8.0	50.0000	5.404036e1
STD_100ppb	Standard	4.76e7	2.237e6	4.13	7.3	100.0000	9.383557e1
STD_200ppb	Standard	8.71e7	2.096e6	4.12	6.9	200.0000	1.833193e2
STD_500ppb	Standard	1.62e8	1.785e6	4.12	6.5	500.0000	3.991808e2
V2.0_MW_RQC1_20240724	Quality Control	N/A	1.511e6	N/A	N/A	0.0000	N/A
Blank	Unknown	N/A	9.253e2	N/A	N/A	N/A	N/A
V3.0_MWMS_20240725_1	Unknown	4.97e6	4.177e6	4.12	6.7	N/A	5.238613e0
MWXS243064a_R1	Quality Control	N/A	1.316e6	N/A	N/A	0.0000	N/A
MWXS243064a_R2	Quality Control	N/A	1.226e6	N/A	N/A	0.0000	N/A
MWXS243064a_R3	Quality Control	N/A	1.274e6	N/A	N/A	0.0000	N/A
T24186682b_a	Unknown	N/A	1.337e6	N/A	N/A	N/A	N/A
T24186682b_b	Unknown	N/A	1.303e6	N/A	N/A	N/A	N/A
T24186682b_c	Unknown	N/A	1.287e6	N/A	N/A	N/A	N/A
T24186683b_a	Unknown	N/A	1.219e6	N/A	N/A	N/A	N/A
T24186683b_b	Unknown	N/A	1.264e6	N/A	N/A	N/A	N/A
T24186683b_c	Unknown	N/A	1.317e6	N/A	N/A	N/A	N/A
T24186684b_a	Unknown	N/A	1.242e6	N/A	N/A	N/A	N/A
T24186684b_b	Unknown	N/A	1.186e6	N/A	N/A	N/A	N/A
T24186684b_c	Unknown	N/A	1.175e6	N/A	N/A	N/A	N/A

Compound name: iP9G
Regression Equation: y = 0.22677 x + 0.00199 (r = 0.99384) (weighting: 1 / x^2)

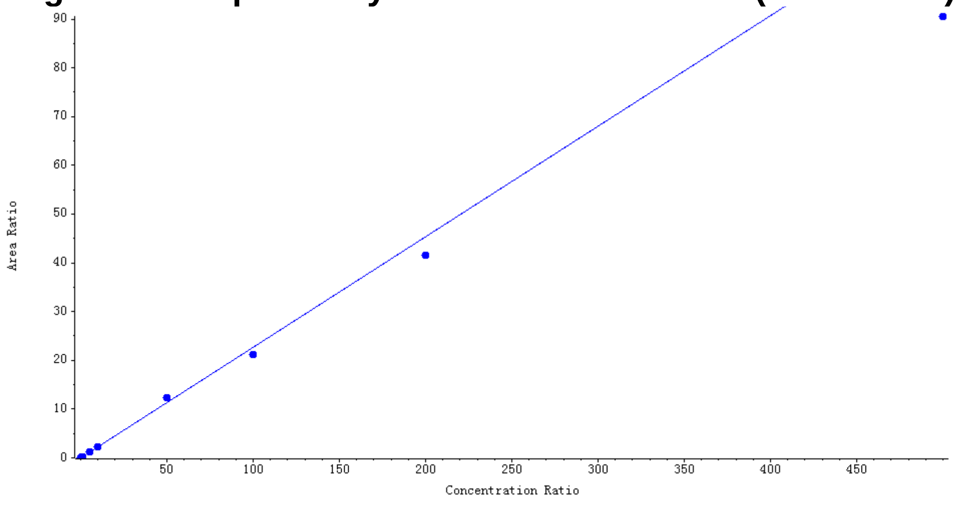

Peak Review

Blank

iP9G AREA:N/A S/N:N/A

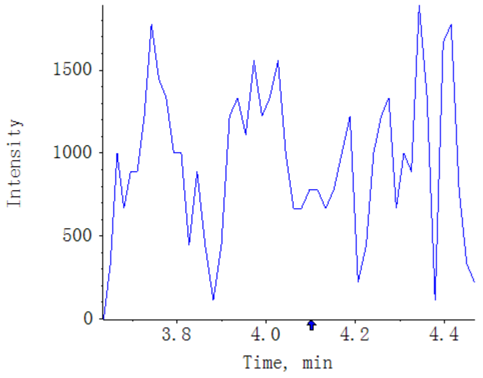

V3.0_MWMS_20240725_1

iP9G AREA:4.97e6 S/N:6.7

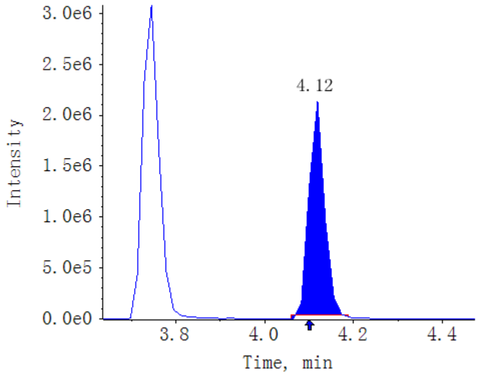

T24186682b_a

iP9G AREA:N/A S/N:N/A

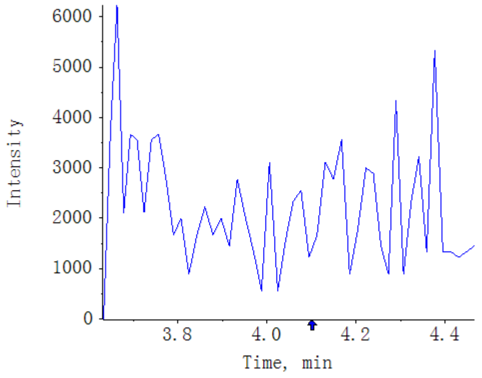

T24186682b_b

iP9G AREA:N/A S/N:N/A

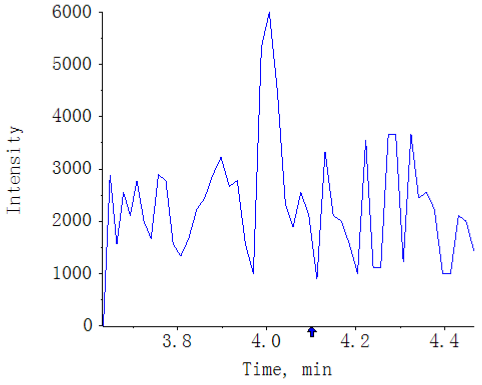

T24186682b_c

iP9G AREA:N/A S/N:N/A

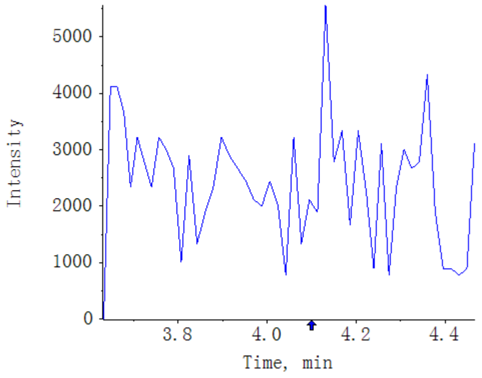

T24186683b_a

iP9G AREA:N/A S/N:N/A

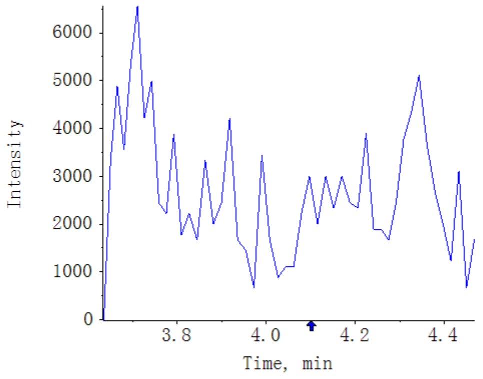

T24186683b_b

iP9G AREA:N/A S/N:N/A

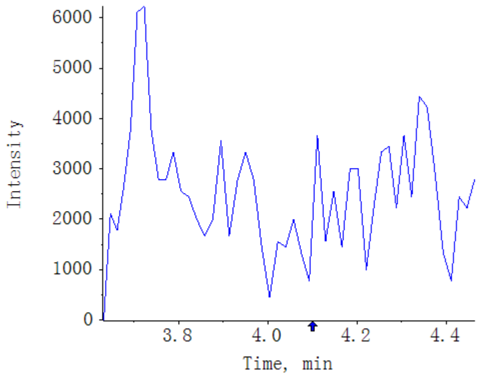

T24186683b_c

iP9G AREA:N/A S/N:N/A

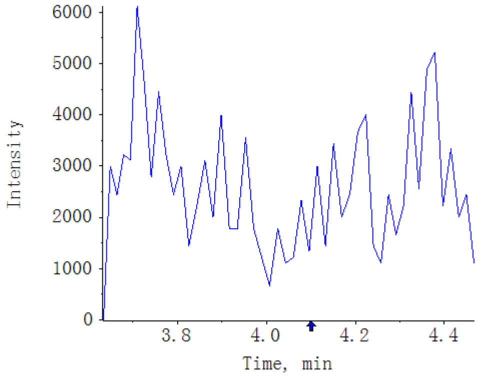

T24186684b_a

iP9G AREA:N/A S/N:N/A

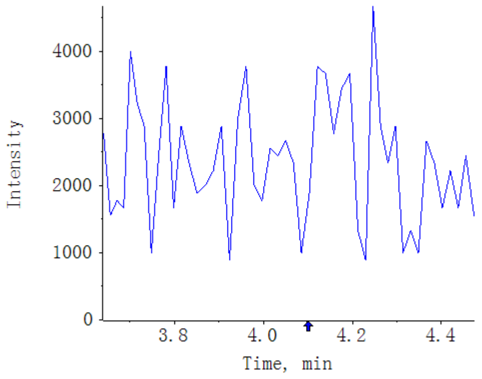

T24186684b_b

iP9G AREA:N/A S/N:N/A

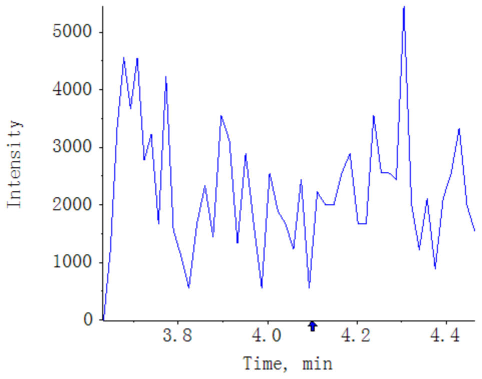

T24186684b_c

iP9G AREA:N/A S/N:N/A

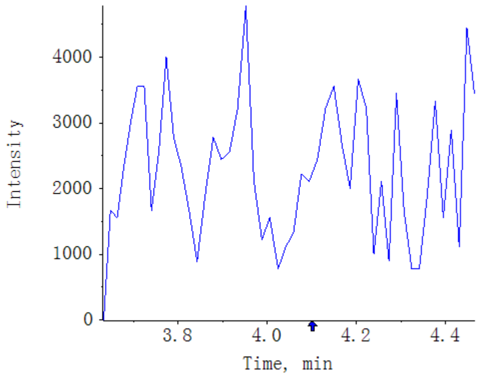

Result Table	MWXS-24-3064-a_9_WH6500-17_A20-3_V6.0_WSS_20240730	Algorithm Used	MQ4
Acquisition Method	ACC-PHs_V6.0_WH6500-17_CMY_20240521.dam	Instrument Name	Triple Quad 6500+ Low Mass
Project	N/A	Analytes QTY	109:38

Compound name: iP7G (366.1 / 204.2)

Sample Name	Sample Type	Area (cps)	Is Area (cps)	RT (min)	S/N	Target Conc	Calculated Conc.()
STD_0.01ppb	Standard	9.64e3	3.065e6	3.75	9.1	0.0100	9.687110e-3
STD_0.05ppb	Standard	5.74e4	3.030e6	3.74	13.1	0.0500	5.363909e-2
STD_0.1ppb	Standard	1.25e5	3.037e6	3.75	17.6	0.1000	1.155150e-1
STD_0.5ppb	Standard	5.38e5	2.659e6	3.74	14.3	0.5000	5.644474e-1
STD_1ppb	Standard	8.86e5	2.814e6	3.74	14.9	1.0000	8.779561e-1
STD_5ppb	Standard	4.83e6	2.801e6	3.76	18.9	5.0000	4.802148e0
STD_10ppb	Standard	8.84e6	2.618e6	3.74	15.1	10.0000	9.400591e0
STD_50ppb	Standard	4.54e7	2.412e6	3.74	13.9	50.0000	5.237855e1
STD_100ppb	Standard	7.61e7	2.237e6	3.75	15.7	100.0000	9.470949e1
STD_200ppb	Standard	1.36e8	2.096e6	3.74	12.2	200.0000	1.802704e2
STD_500ppb	Standard	N/A	1.785e6	N/A	N/A	500.0000	N/A
V2.0_MW_RQC1_20240724	Quality Control	9.53e4	1.511e6	3.69	8.8	0.0000	1.765161e-1
Blank	Unknown	N/A	9.253e2	N/A	N/A	N/A	N/A
V3.0_MWMS_20240725_1	Unknown	8.86e6	4.177e6	3.74	13.2	N/A	5.905800e0
MWXS243064a_R1	Quality Control	N/A	1.316e6	N/A	N/A	0.0000	N/A
MWXS243064a_R2	Quality Control	N/A	1.226e6	N/A	N/A	0.0000	N/A
MWXS243064a_R3	Quality Control	N/A	1.274e6	N/A	N/A	0.0000	N/A
T24186682b_a	Unknown	N/A	1.337e6	N/A	N/A	N/A	N/A
T24186682b_b	Unknown	N/A	1.303e6	N/A	N/A	N/A	N/A
T24186682b_c	Unknown	N/A	1.287e6	N/A	N/A	N/A	N/A
T24186683b_a	Unknown	3.89e4	1.219e6	3.71	7.5	N/A	8.974012e-2
T24186683b_b	Unknown	3.58e4	1.264e6	3.71	6.8	N/A	7.968817e-2
T24186683b_c	Unknown	3.60e4	1.317e6	3.73	6.1	N/A	7.706537e-2
T24186684b_a	Unknown	N/A	1.242e6	N/A	N/A	N/A	N/A
T24186684b_b	Unknown	N/A	1.186e6	N/A	N/A	N/A	N/A
T24186684b_c	Unknown	N/A	1.175e6	N/A	N/A	N/A	N/A

Compound name: iP7G
Regression Equation: y = 0.35918 x + -3.33877e-4 (r = 0.99511) (weighting: 1 / x^2)

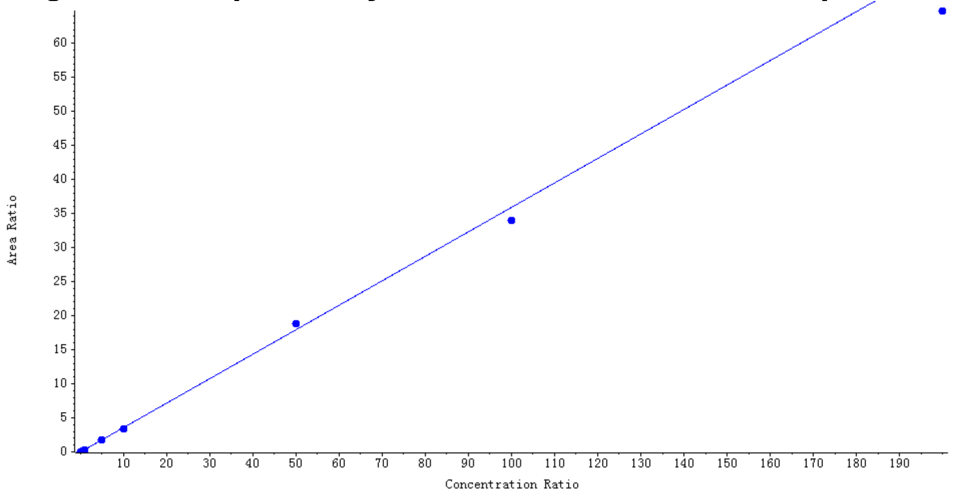

Peak Review

Blank
iP7G AREA:N/A S/N:N/A

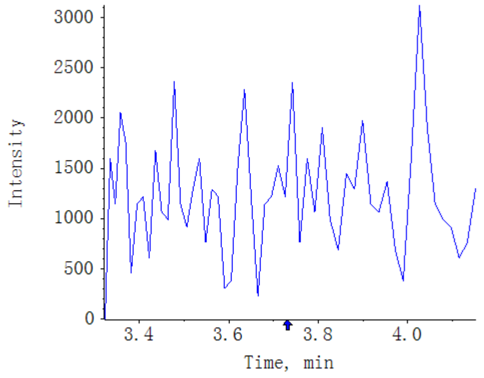

V3.0_MWMS_20240725_1
iP7G AREA:8.86e6 S/N:13.2

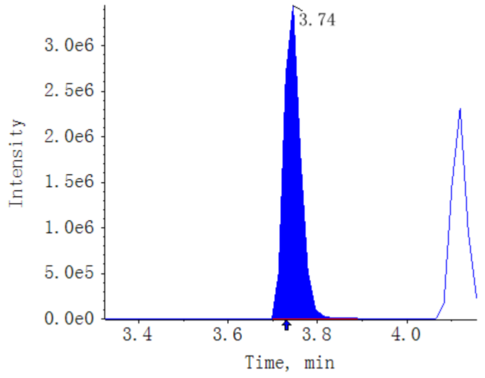

T24186682b_a
iP7G AREA:N/A S/N:N/A

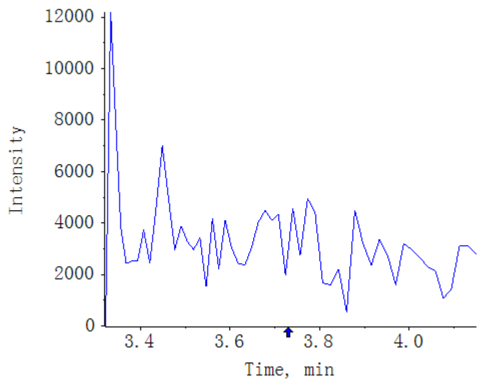

T24186682b_b
iP7G AREA:N/A S/N:N/A

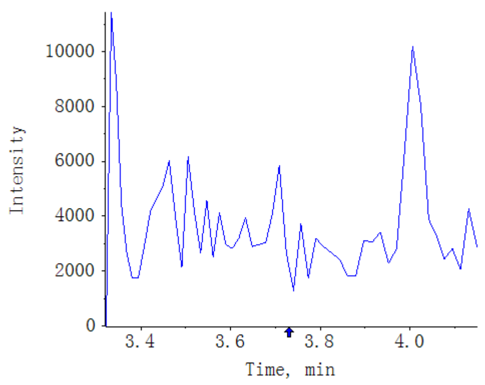

T24186682b_c
iP7G AREA:N/A S/N:N/A

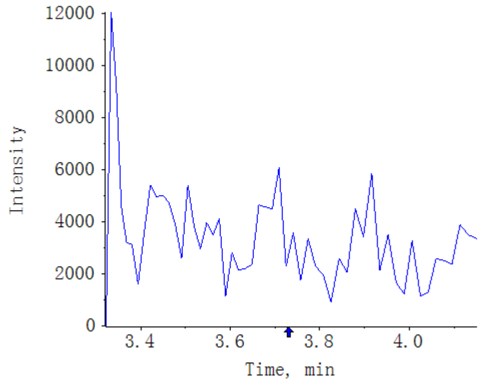

T24186683b_a
iP7G AREA:3.89e4 S/N:7.5

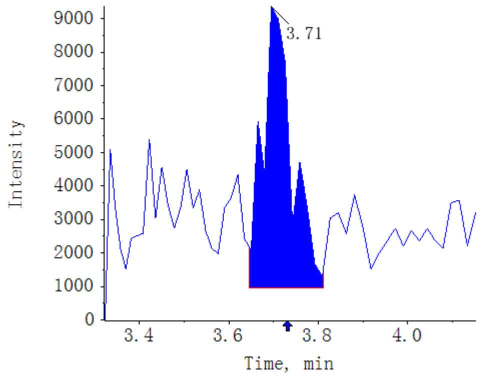

T24186683b_b
iP7G AREA:3.58e4 S/N:6.8

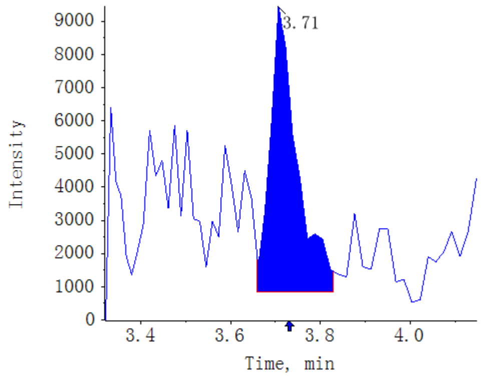

T24186683b_c
iP7G AREA:3.60e4 S/N:6.1

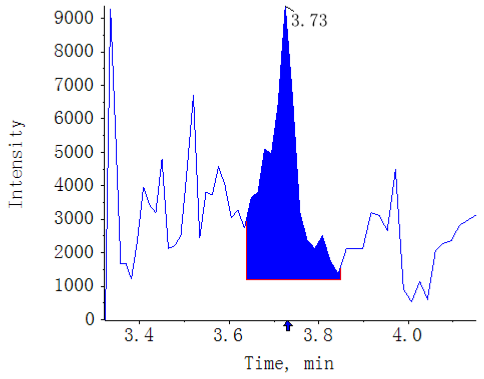

T24186684b_a
iP7G AREA:N/A S/N:N/A

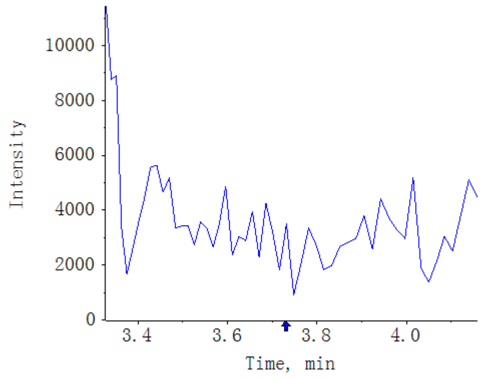

T24186684b_b
iP7G AREA:N/A S/N:N/A

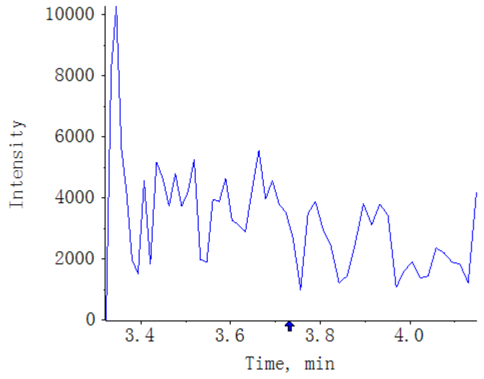

T24186684b_c
iP7G AREA:N/A S/N:N/A

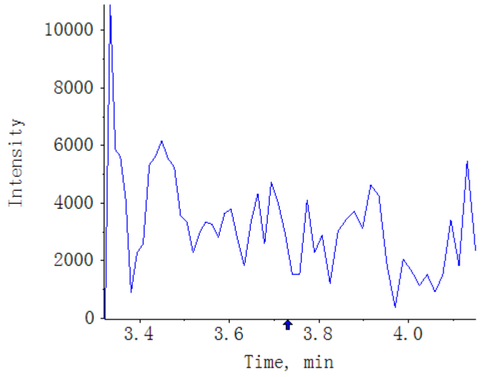

Result Table	MWXS-24-3064-a_9_WH6500-17_A20-3_V6.0_WSS_20240730	Algorithm Used	MQ4
Acquisition Method	ACC-PHs_V6.0_WH6500-17_CMY_20240521.dam	Instrument Name	Triple Quad 6500+ Low Mass
Project	N/A	Analytes QTY	109:39

Compound name: tZOG (382.1 / 220.2)

Sample Name	Sample Type	Area (cps)	Is Area (cps)	RT (min)	S/N	Target Conc	Calculated Conc.()
STD_0.01ppb	Standard	N/A	1.546e6	N/A	N/A	0.0100	N/A
STD_0.05ppb	Standard	8.76e3	1.581e6	2.36	9.3	0.0500	6.734993e-2
STD_0.1ppb	Standard	1.54e4	1.618e6	2.39	12.9	0.1000	1.118586e-1
STD_0.5ppb	Standard	5.72e4	1.352e6	2.39	25.8	0.5000	4.777939e-1
STD_1ppb	Standard	1.05e5	1.385e6	2.36	37.4	1.0000	8.495433e-1
STD_5ppb	Standard	5.27e5	1.227e6	2.38	60.0	5.0000	4.788994e0
STD_10ppb	Standard	9.58e5	1.204e6	2.39	77.0	10.0000	8.874070e0
STD_50ppb	Standard	5.14e6	1.243e6	2.37	91.2	50.0000	4.608333e1
STD_100ppb	Standard	9.98e6	1.246e6	2.38	100.2	100.0000	8.930718e1
STD_200ppb	Standard	2.42e7	1.273e6	2.37	144.8	200.0000	2.123869e2
STD_500ppb	Standard	5.48e7	1.212e6	2.37	155.8	500.0000	5.037030e2
V2.0_MW_RQC1_20240724	Quality Control	1.78e5	1.973e6	2.81	11.4	0.0000	1.013305e0
Blank	Unknown	N/A	1.128e3	N/A	N/A	N/A	N/A
V3.0_MWMS_20240725_1	Unknown	7.31e5	1.465e6	2.80	63.8	N/A	5.570803e0
MWXS243064a_R1	Quality Control	5.43e4	1.869e6	2.74	6.9	0.0000	3.292795e-1
MWXS243064a_R2	Quality Control	4.91e4	1.799e6	2.73	7.4	0.0000	3.098863e-1
MWXS243064a_R3	Quality Control	5.24e4	1.915e6	2.71	5.8	0.0000	3.107052e-1
T24186682b_a	Unknown	4.37e4	1.983e6	2.76	6.0	N/A	2.515182e-1
T24186682b_b	Unknown	4.51e4	1.875e6	2.75	5.9	N/A	2.734596e-1
T24186682b_c	Unknown	3.49e4	1.962e6	2.77	5.4	N/A	2.040607e-1
T24186683b_a	Unknown	6.38e4	1.671e6	2.76	5.6	N/A	4.311695e-1
T24186683b_b	Unknown	6.71e4	1.677e6	2.83	9.5	N/A	4.517354e-1
T24186683b_c	Unknown	5.46e4	1.667e6	2.77	8.4	N/A	3.705524e-1
T24186684b_a	Unknown	6.18e4	1.717e6	2.76	12.7	N/A	4.065167e-1
T24186684b_b	Unknown	5.22e4	1.710e6	2.77	9.5	N/A	3.459575e-1
T24186684b_c	Unknown	4.51e4	1.760e6	2.74	7.2	N/A	2.913183e-1

Compound name: tZOG
Regression Equation: y = 0.08969 x + -5.01177e-4 (r = 0.99861) (weighting: 1 / x)

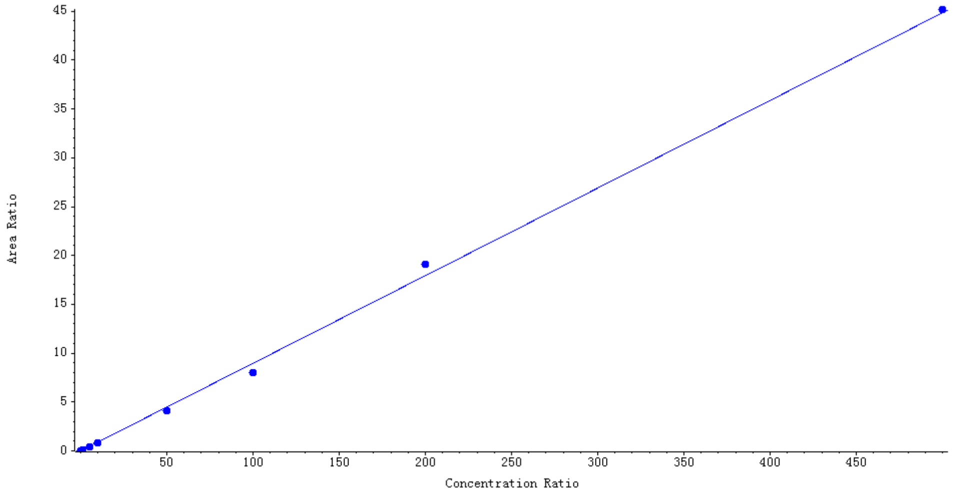

Peak Review

Blank

tZOG AREA:N/A S/N:N/A

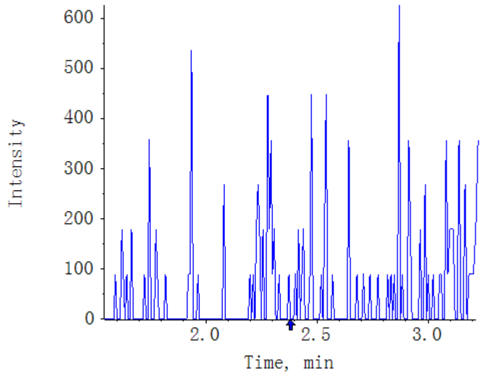

V3.0_MWMS_20240725_1

tZOG AREA:7.31e5 S/N:63.8

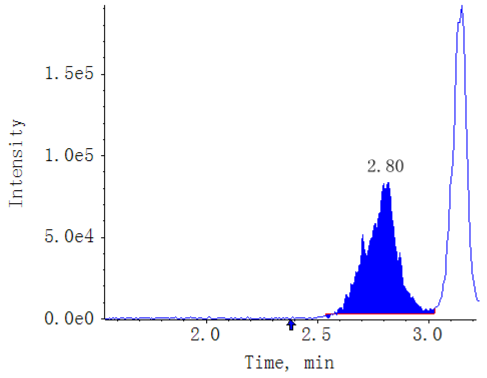

T24186682b_a

tZOG AREA:4.37e4 S/N:6.0

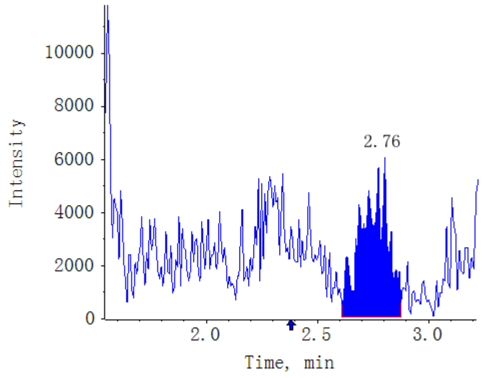

T24186682b_b

tZOG AREA:4.51e4 S/N:5.9

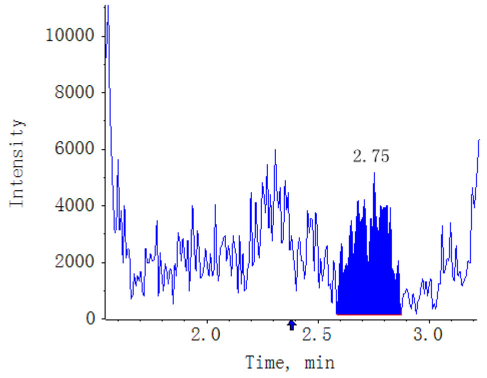

T24186682b_c

tZOG AREA:3.49e4 S/N:5.4

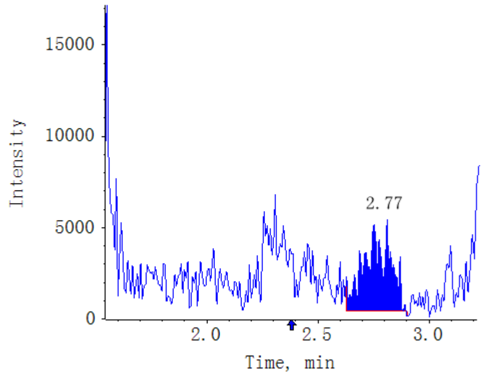

T24186683b_a

tZOG AREA:6.38e4 S/N:5.6

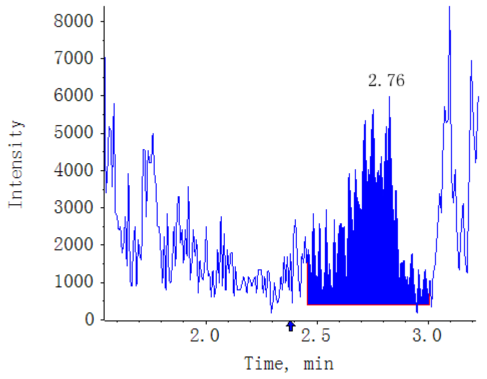

T24186683b_b

tZOG AREA:6.71e4 S/N:9.5

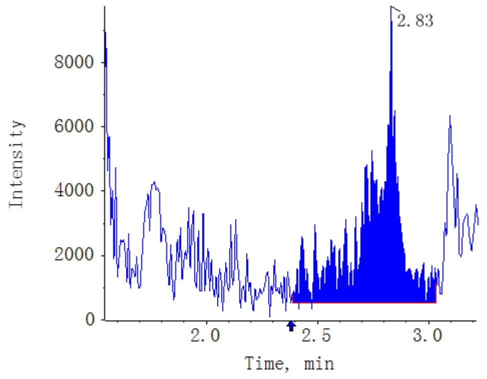

T24186683b_c

tZOG AREA:5.46e4 S/N:8.4

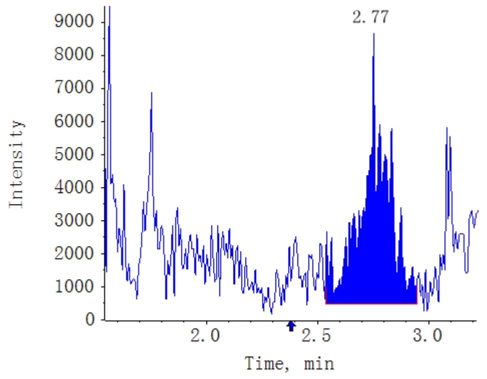

T24186684b_a

tZOG AREA:6.18e4 S/N:12.7

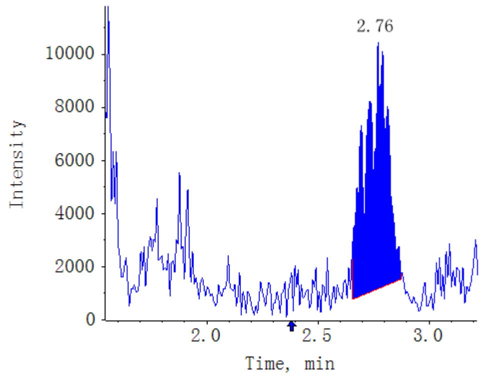

T24186684b_b

tZOG AREA:5.22e4 S/N:9.5

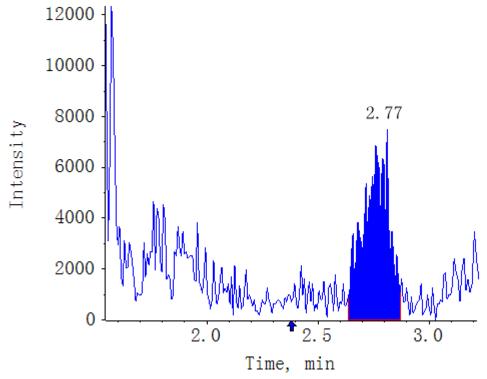

T24186684b_c

tZOG AREA:4.51e4 S/N:7.2

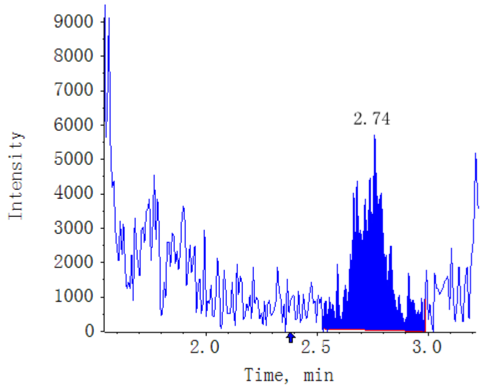

Result Table	MWXS-24-3064-a_9_WH6500-17_A20-3_V6.0_WSS_20240730	Algorithm Used	MQ4
Acquisition Method	ACC-PHs_V6.0_WH6500-17_CMY_20240521.dam	Instrument Name	Triple Quad 6500+ Low Mass
Project	N/A	Analytes QTY	109:40

Compound name: DHZROG (516.1 / 222.1)

Sample Name	Sample Type	Area (cps)	Is Area (cps)	RT (min)	S/N	Target Conc	Calculated Conc.()
STD_0.01ppb	Standard	4.29e3	1.404e6	3.52	33.5	0.0100	1.119699e-2
STD_0.05ppb	Standard	2.73e4	1.422e6	3.52	57.7	0.0500	5.797082e-2
STD_0.1ppb	Standard	4.53e4	1.429e6	3.53	88.2	0.1000	9.419762e-2
STD_0.5ppb	Standard	2.23e5	1.275e6	3.52	62.1	0.5000	5.094877e-1
STD_1ppb	Standard	3.79e5	1.231e6	3.52	71.8	1.0000	8.948885e-1
STD_5ppb	Standard	1.96e6	1.217e6	3.54	76.1	5.0000	4.663948e0
STD_10ppb	Standard	3.87e6	1.257e6	3.52	86.7	10.0000	8.922066e0
STD_50ppb	Standard	2.50e7	1.407e6	3.52	71.3	50.0000	5.144113e1
STD_100ppb	Standard	4.74e7	1.346e6	3.53	89.2	100.0000	1.021800e2
STD_200ppb	Standard	9.08e7	1.329e6	3.53	70.3	200.0000	1.978851e2
STD_500ppb	Standard	N/A	1.185e6	N/A	N/A	500.0000	N/A
V2.0_MW_RQC1_20240724	Quality Control	2.46e4	2.861e6	3.53	13.5	0.0000	2.730061e-2
Blank	Unknown	N/A	2.118e2	N/A	N/A	N/A	N/A
V3.0_MWMS_20240725_1	Unknown	3.83e6	2.160e6	3.52	69.0	N/A	5.141254e0
MWXS243064a_R1	Quality Control	N/A	1.061e6	N/A	N/A	0.0000	N/A
MWXS243064a_R2	Quality Control	N/A	1.065e6	N/A	N/A	0.0000	N/A
MWXS243064a_R3	Quality Control	N/A	1.061e6	N/A	N/A	0.0000	N/A
T24186682b_a	Unknown	N/A	1.263e6	N/A	N/A	N/A	N/A
T24186682b_b	Unknown	N/A	1.159e6	N/A	N/A	N/A	N/A
T24186682b_c	Unknown	N/A	1.192e6	N/A	N/A	N/A	N/A
T24186683b_a	Unknown	N/A	1.029e6	N/A	N/A	N/A	N/A
T24186683b_b	Unknown	N/A	1.049e6	N/A	N/A	N/A	N/A
T24186683b_c	Unknown	N/A	1.129e6	N/A	N/A	N/A	N/A
T24186684b_a	Unknown	N/A	1.242e6	N/A	N/A	N/A	N/A
T24186684b_b	Unknown	N/A	1.132e6	N/A	N/A	N/A	N/A
T24186684b_c	Unknown	N/A	1.051e6	N/A	N/A	N/A	N/A

Compound name: DHZROG
Regression Equation: y = 0.34507 x + -8.05828e-4 (r = 0.99964) (weighting: 1 / x)

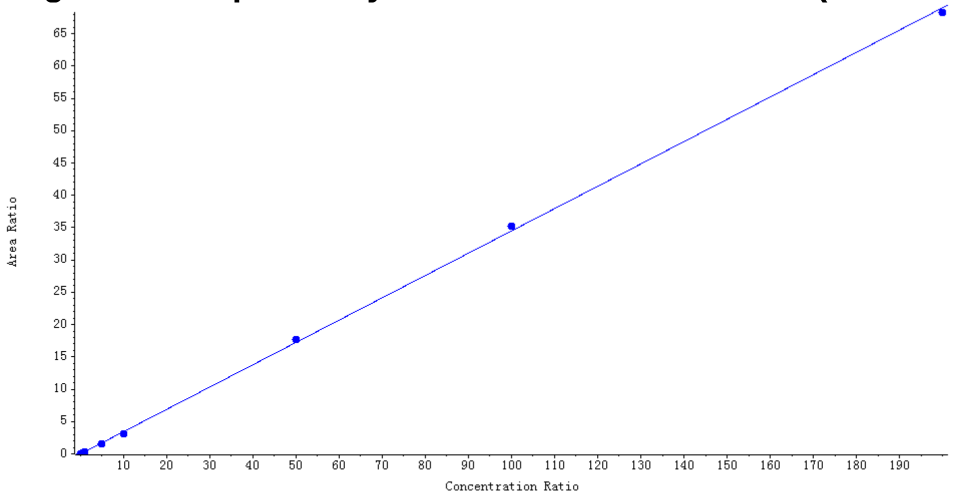

Peak Review

Blank
DHZROG AREA:N/A S/N:N/A

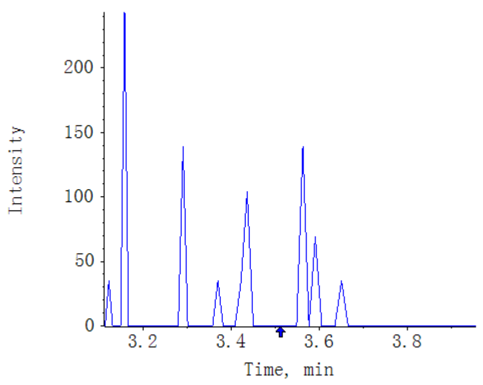

V3.0_MWMS_20240725_1
DHZROG AREA:3.83e6 S/N:69.0

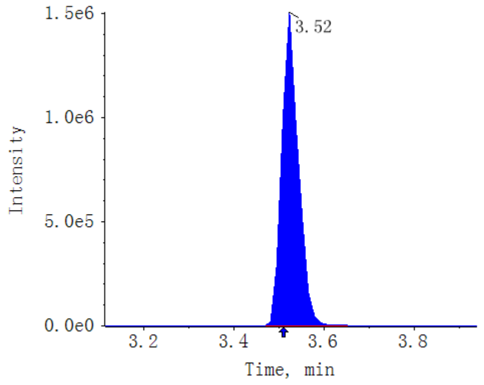

T24186682b_a
DHZROG AREA:N/A S/N:N/A

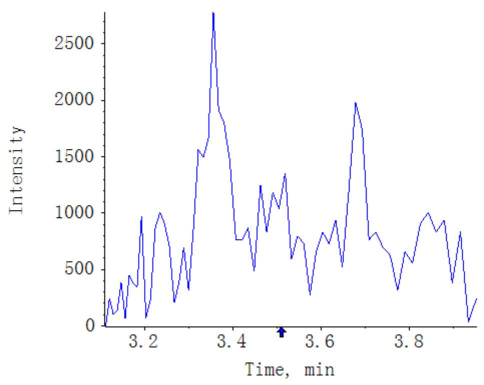

T24186682b_b
DHZROG AREA:N/A S/N:N/A

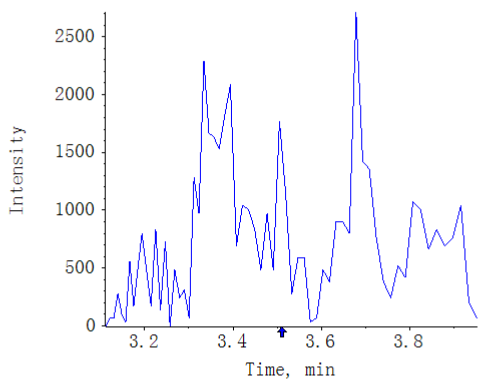

T24186682b_c
DHZROG AREA:N/A S/N:N/A

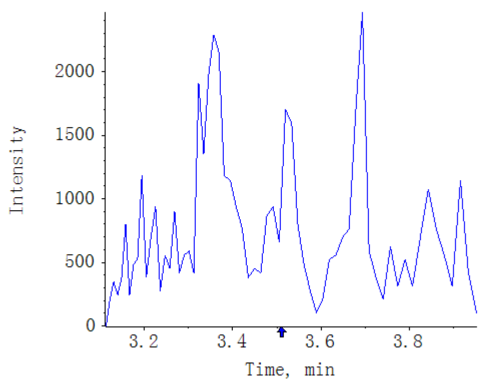

T24186683b_a
DHZROG AREA:N/A S/N:N/A

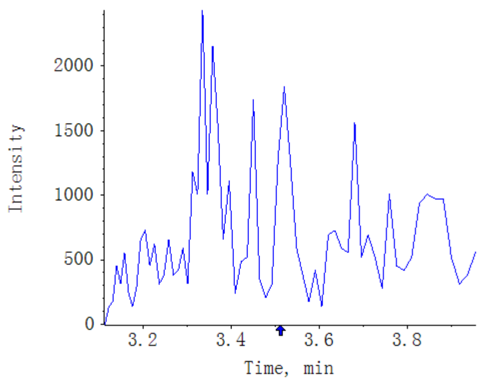

T24186683b_b
DHZROG AREA:N/A S/N:N/A

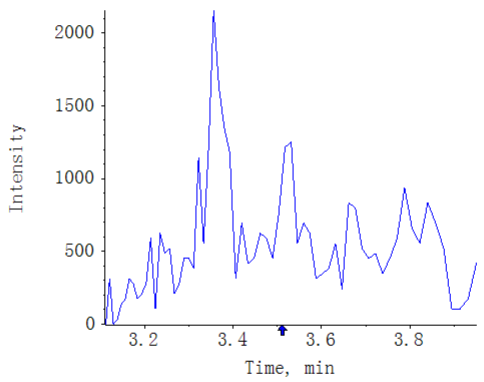

T24186683b_c
DHZROG AREA:N/A S/N:N/A

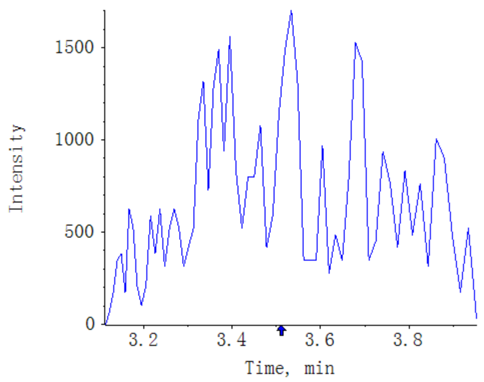

T24186684b_a
DHZROG AREA:N/A S/N:N/A

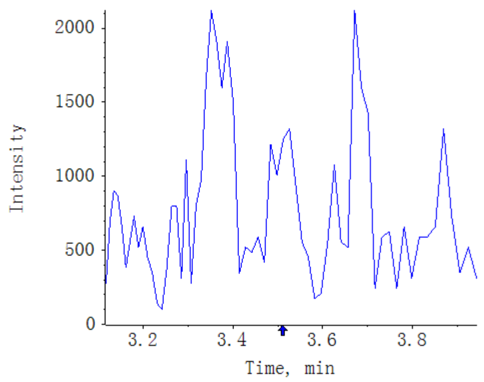

T24186684b_b
DHZROG AREA:N/A S/N:N/A

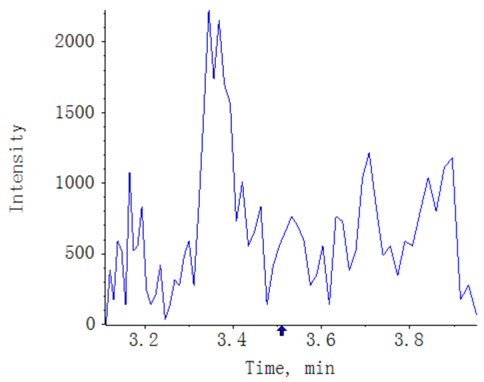

T24186684b_c
DHZROG AREA:N/A S/N:N/A

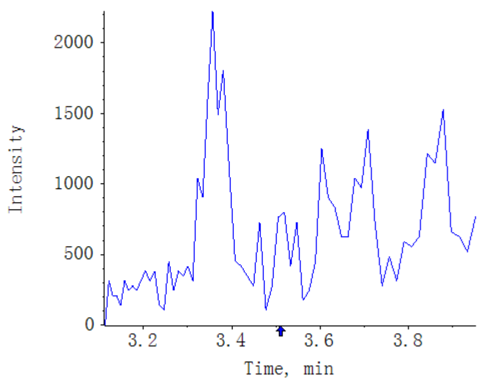

Result Table	MWXS-24-3064-a_9_WH6500-17_A20-3_V6.0_WSS_20240730	Algorithm Used	MQ4
Acquisition Method	ACC-PHs_V6.0_WH6500-17_CMY_20240521.dam	Instrument Name	Triple Quad 6500+ Low Mass
Project	N/A	Analytes QTY	109:41

Compound name: cZROG (514.1 / 382.0)

Sample Name	Sample Type	Area (cps)	Is Area (cps)	RT (min)	S/N	Target Conc	Calculated Conc.()
STD_0.01ppb	Standard	2.96e3	6.534e6	3.55	9.0	0.0100	1.015095e-2
STD_0.05ppb	Standard	1.98e4	7.105e6	3.55	26.2	0.0500	4.776405e-2
STD_0.1ppb	Standard	4.05e4	6.945e6	3.56	31.1	0.1000	9.678576e-2
STD_0.5ppb	Standard	1.66e5	5.879e6	3.54	62.9	0.5000	4.585818e-1
STD_1ppb	Standard	3.16e5	5.871e6	3.55	76.9	1.0000	8.699315e-1
STD_5ppb	Standard	1.57e6	5.223e6	3.55	85.0	5.0000	4.859520e0
STD_10ppb	Standard	3.20e6	5.065e6	3.54	107.2	10.0000	1.019504e1
STD_50ppb	Standard	1.93e7	4.847e6	3.54	90.7	50.0000	6.416316e1
STD_100ppb	Standard	N/A	4.595e6	N/A	N/A	100.0000	N/A
STD_200ppb	Standard	N/A	4.227e6	N/A	N/A	200.0000	N/A
STD_500ppb	Standard	N/A	3.238e6	N/A	N/A	500.0000	N/A
V2.0_MW_RQC1_20240724	Quality Control	1.88e6	5.942e6	3.54	106.4	0.0000	5.106583e0
Blank	Unknown	N/A	2.845e3	N/A	N/A	N/A	N/A
V3.0_MWMS_20240725_1	Unknown	2.90e6	1.146e7	3.54	85.0	N/A	4.087653e0
MWXS243064a_R1	Quality Control	4.29e4	6.293e6	3.53	13.1	0.0000	1.126558e-1
MWXS243064a_R2	Quality Control	4.55e4	6.413e6	3.53	13.0	0.0000	1.171149e-1
MWXS243064a_R3	Quality Control	4.75e4	6.529e6	3.52	11.2	0.0000	1.201770e-1
T24186682b_a	Unknown	4.17e4	6.437e6	3.53	15.0	N/A	1.071266e-1
T24186682b_b	Unknown	3.80e4	6.323e6	3.53	10.8	N/A	9.956828e-2
T24186682b_c	Unknown	3.76e4	6.686e6	3.54	9.4	N/A	9.350297e-2
T24186683b_a	Unknown	7.84e4	6.369e6	3.53	16.4	N/A	2.011002e-1
T24186683b_b	Unknown	7.55e4	6.536e6	3.53	11.7	N/A	1.890261e-1
T24186683b_c	Unknown	8.41e4	6.791e6	3.53	12.5	N/A	2.022851e-1
T24186684b_a	Unknown	2.89e4	5.743e6	3.53	10.0	N/A	8.396082e-2
T24186684b_b	Unknown	2.15e4	6.364e6	3.54	10.0	N/A	5.737690e-2
T24186684b_c	Unknown	2.40e4	6.178e6	3.54	12.7	N/A	6.538489e-2

Compound name: cZROG
Regression Equation: y = 0.06207 x + -1.77091e-4 (r = 0.99151) (weighting: 1 / x^2)

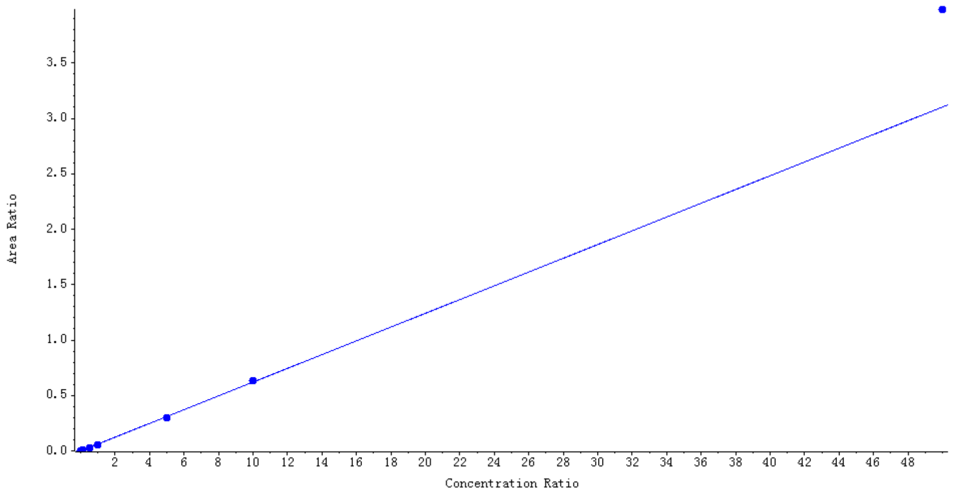

Peak Review

Blank
cZROG AREA:N/A S/N:N/A

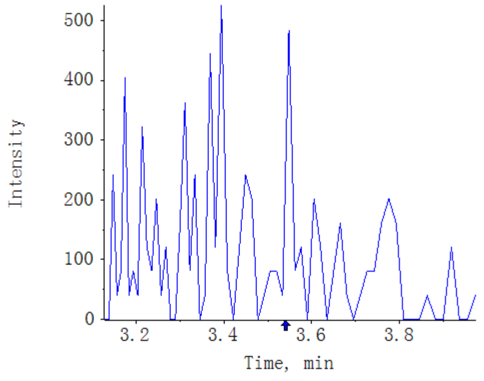

V3.0_MWMS_20240725_1
cZROG AREA:2.90e6 S/N:85.0

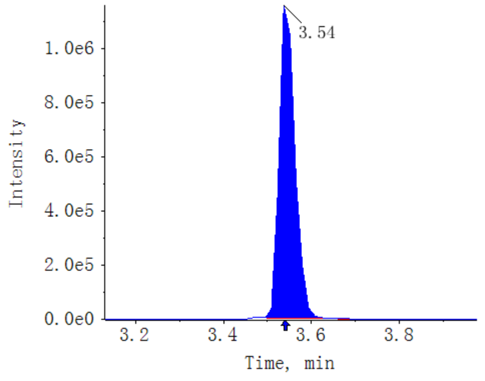

T24186682b_a
cZROG AREA:4.17e4 S/N:15.0

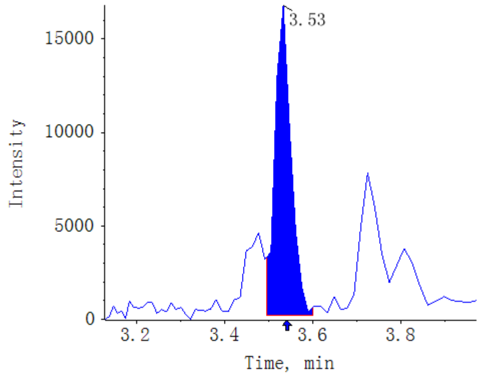

T24186682b_b
cZROG AREA:3.80e4 S/N:10.8

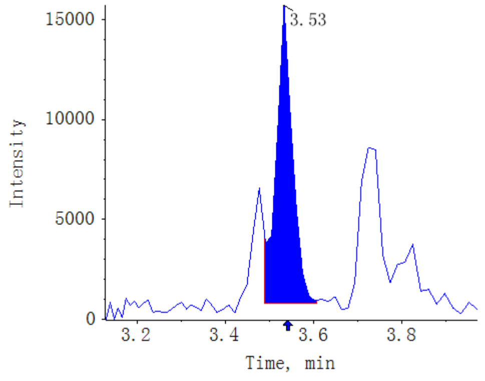

T24186682b_c
cZROG AREA:3.76e4 S/N:9.4

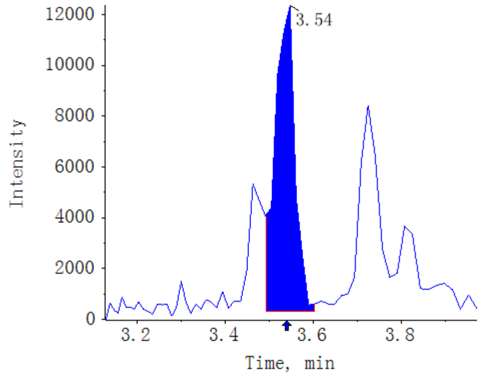

T24186683b_a
cZROG AREA:7.84e4 S/N:16.4

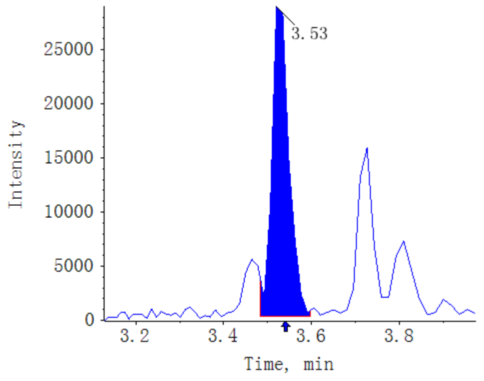

T24186683b_b
cZROG AREA:7.55e4 S/N:11.7

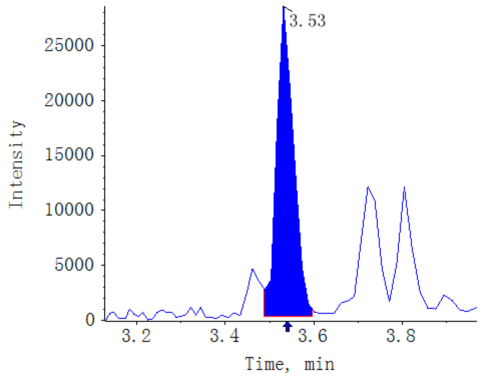

T24186683b_c
cZROG AREA:8.41e4 S/N:12.5

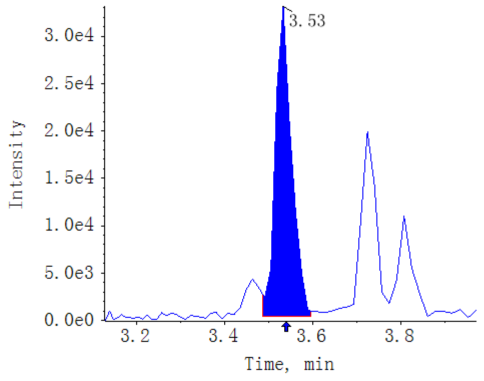

T24186684b_a
cZROG AREA:2.89e4 S/N:10.0

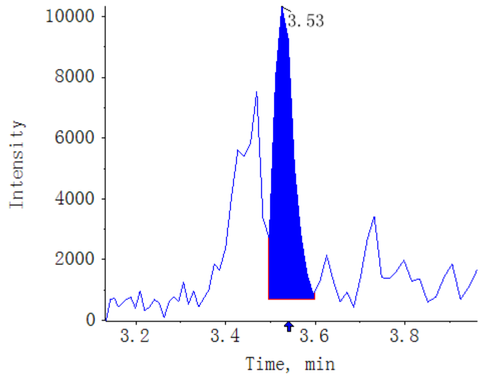

T24186684b_b
cZROG AREA:2.15e4 S/N:10.0

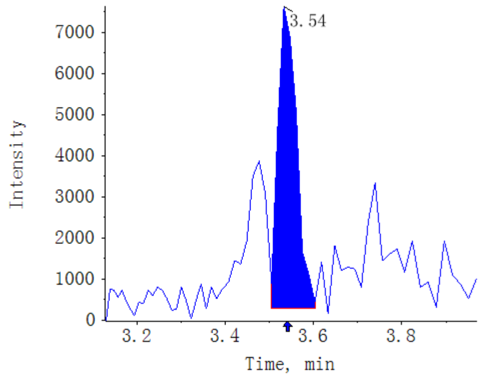

T24186684b_c
cZROG AREA:2.40e4 S/N:12.7

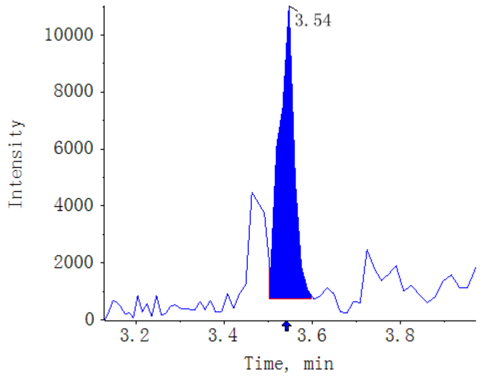

Result Table	MWXS-24-3064-a_9_WH6500-17_A20-3_V6.0_WSS_20240730	Algorithm Used	MQ4
Acquisition Method	ACC-PHs_V6.0_WH6500-17_CMY_20240521.dam	Instrument Name	Triple Quad 6500+ Low Mass
Project	N/A	Analytes QTY	109:42

Compound name: mT9G (404.1 / 242.3)

Sample Name	Sample Type	Area (cps)	Is Area (cps)	RT (min)	S/N	Target Conc	Calculated Conc.()
STD_0.01ppb	Standard	5.59e3	3.065e6	3.75	4.5	0.0100	9.498431e-3
STD_0.05ppb	Standard	2.76e4	3.030e6	3.75	6.0	0.0500	5.849531e-2
STD_0.1ppb	Standard	5.24e4	3.037e6	3.76	7.7	0.1000	1.135913e-1
STD_0.5ppb	Standard	2.26e5	2.659e6	3.75	8.0	0.5000	5.705905e-1
STD_1ppb	Standard	4.15e5	2.814e6	3.75	7.9	1.0000	9.903731e-1
STD_5ppb	Standard	2.06e6	2.801e6	3.76	6.9	5.0000	4.962104e0
STD_10ppb	Standard	3.54e6	2.618e6	3.75	6.9	10.0000	9.111796e0
STD_50ppb	Standard	1.61e7	2.412e6	3.74	6.7	50.0000	4.503095e1
STD_100ppb	Standard	2.68e7	2.237e6	3.76	6.7	100.0000	8.085646e1
STD_200ppb	Standard	N/A	2.096e6	N/A	N/A	200.0000	N/A
STD_500ppb	Standard	N/A	1.785e6	N/A	N/A	500.0000	N/A
V2.0_MW_RQC1_20240724	Quality Control	N/A	1.511e6	N/A	N/A	0.0000	N/A
Blank	Unknown	N/A	9.253e2	N/A	N/A	N/A	N/A
V3.0_MWMS_20240725_1	Unknown	3.50e6	4.177e6	3.75	7.2	N/A	5.644529e0
MWXS243064a_R1	Quality Control	N/A	1.316e6	N/A	N/A	0.0000	N/A
MWXS243064a_R2	Quality Control	N/A	1.226e6	N/A	N/A	0.0000	N/A
MWXS243064a_R3	Quality Control	N/A	1.274e6	N/A	N/A	0.0000	N/A
T24186682b_a	Unknown	N/A	1.337e6	N/A	N/A	N/A	N/A
T24186682b_b	Unknown	N/A	1.303e6	N/A	N/A	N/A	N/A
T24186682b_c	Unknown	N/A	1.287e6	N/A	N/A	N/A	N/A
T24186683b_a	Unknown	N/A	1.219e6	N/A	N/A	N/A	N/A
T24186683b_b	Unknown	N/A	1.264e6	N/A	N/A	N/A	N/A
T24186683b_c	Unknown	N/A	1.317e6	N/A	N/A	N/A	N/A
T24186684b_a	Unknown	N/A	1.242e6	N/A	N/A	N/A	N/A
T24186684b_b	Unknown	N/A	1.186e6	N/A	N/A	N/A	N/A
T24186684b_c	Unknown	N/A	1.175e6	N/A	N/A	N/A	N/A

Compound name: mT9G
Regression Equation: y = 0.14839 x + 4.15515e-4 (r = 0.99160) (weighting: 1 / x^2)

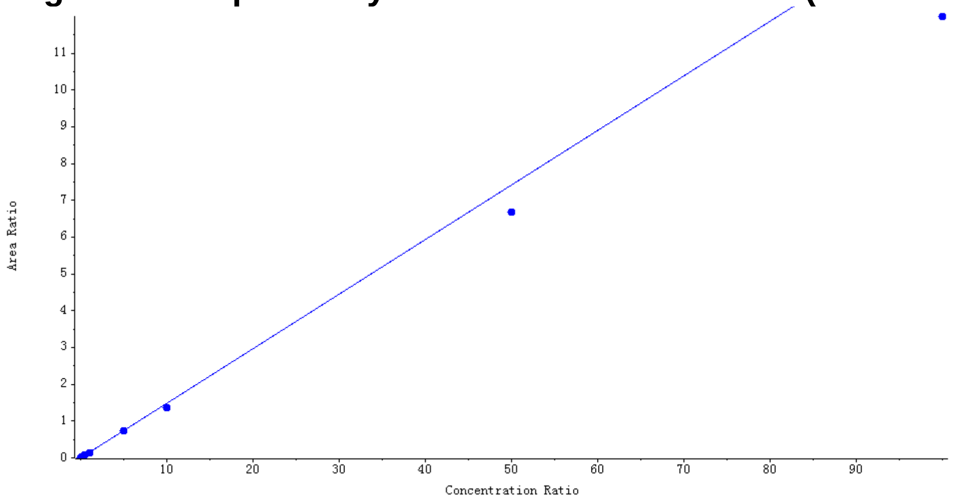

Peak Review

Blank
mT9G AREA:N/A S/N:N/A

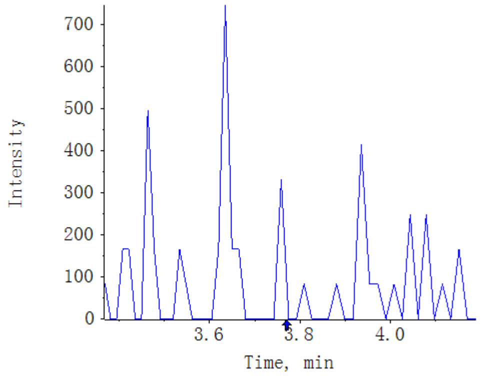

V3.0_MWMS_20240725_1
mT9G AREA:3.50e6 S/N:7.2

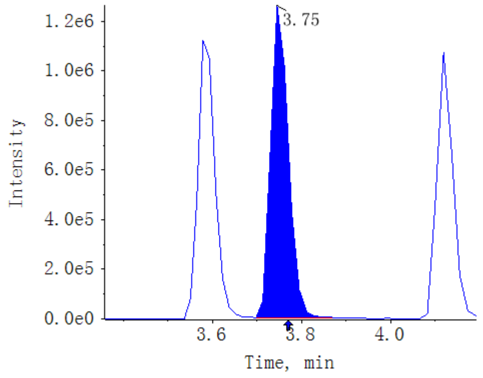

T24186682b_a
mT9G AREA:N/A S/N:N/A

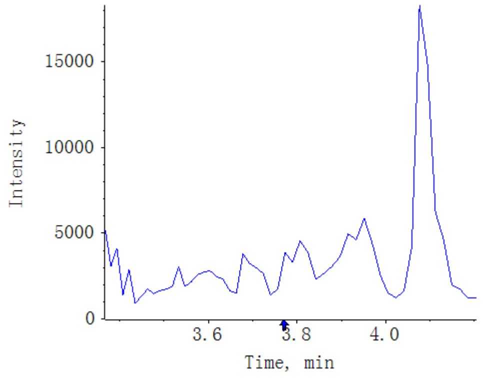

T24186682b_b
mT9G AREA:N/A S/N:N/A

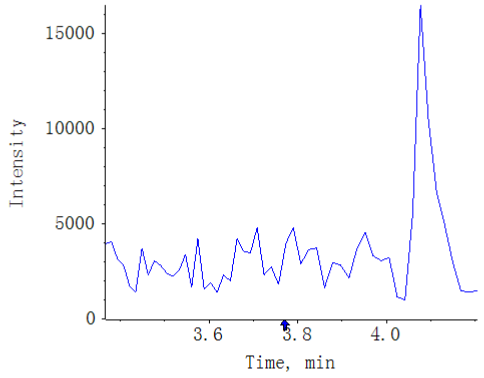

T24186682b_c
mT9G AREA:N/A S/N:N/A

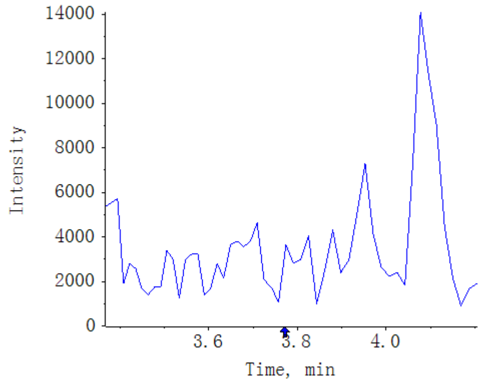

T24186683b_a
mT9G AREA:N/A S/N:N/A

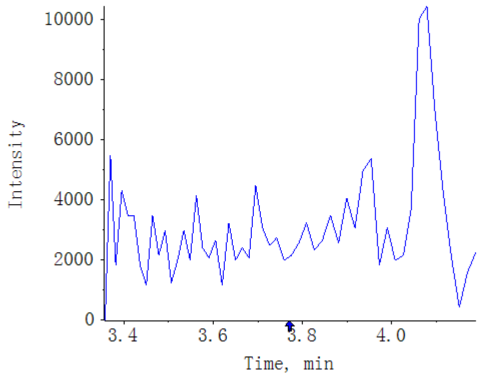

T24186683b_b
mT9G AREA:N/A S/N:N/A

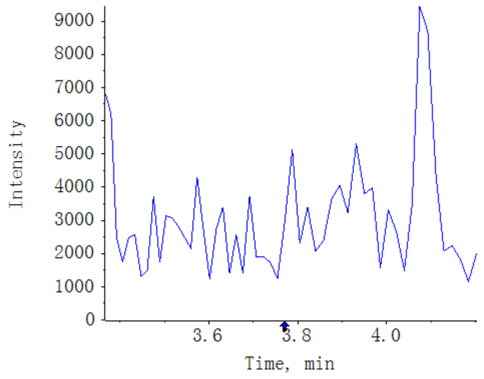

T24186683b_c
mT9G AREA:N/A S/N:N/A

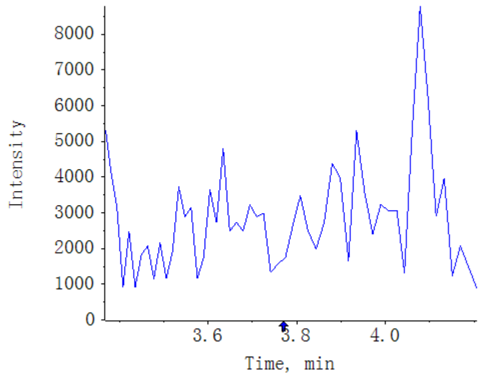

T24186684b_a
mT9G AREA:N/A S/N:N/A

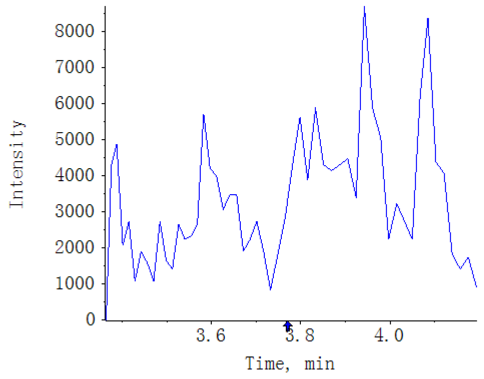

T24186684b_b
mT9G AREA:N/A S/N:N/A

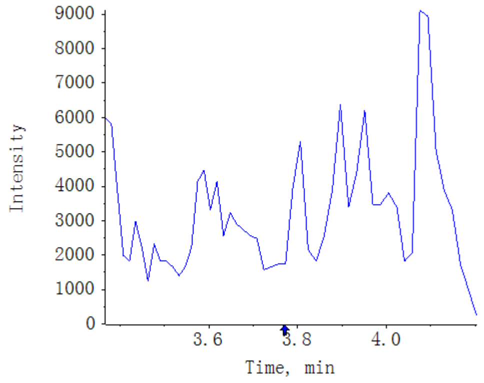

T24186684b_c
mT9G AREA:N/A S/N:N/A

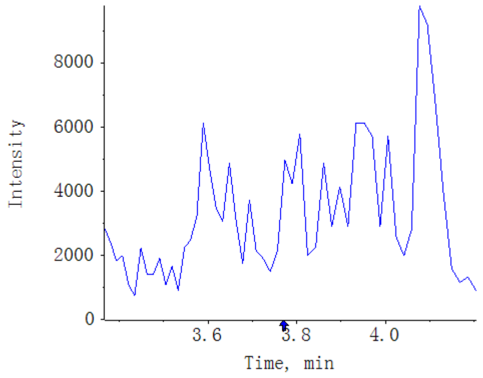

Result Table	MWXS-24-3064-a_9_WH6500-17_A20-3_V6.0_WSS_20240730	Algorithm Used	MQ4
Acquisition Method	ACC-PHs_V6.0_WH6500-17_CMY_20240521.dam	Instrument Name	Triple Quad 6500+ Low Mass
Project	N/A	Analytes QTY	109:43

Compound name: oT9G (404.0 / 136.2)

Sample Name	Sample Type	Area (cps)	Is Area (cps)	RT (min)	S/N	Target Conc	Calculated Conc.()
STD_0.01ppb	Standard	2.92e3	3.065e6	4.12	18.0	0.0100	9.203438e-3
STD_0.05ppb	Standard	2.08e4	3.030e6	4.13	28.2	0.0500	6.568318e-2
STD_0.1ppb	Standard	3.67e4	3.037e6	4.14	42.3	0.1000	1.153850e-1
STD_0.5ppb	Standard	1.56e5	2.659e6	4.13	38.5	0.5000	5.611357e-1
STD_1ppb	Standard	2.72e5	2.814e6	4.13	52.7	1.0000	9.201190e-1
STD_5ppb	Standard	1.45e6	2.801e6	4.14	51.2	5.0000	4.923395e0
STD_10ppb	Standard	2.63e6	2.618e6	4.13	62.3	10.0000	9.559811e0
STD_50ppb	Standard	1.18e7	2.412e6	4.13	56.2	50.0000	4.651313e1
STD_100ppb	Standard	2.04e7	2.237e6	4.14	46.8	100.0000	8.679999e1
STD_200ppb	Standard	3.97e7	2.096e6	4.13	53.7	200.0000	1.805410e2
STD_500ppb	Standard	8.69e7	1.785e6	4.13	51.2	500.0000	4.640625e2
V2.0_MW_RQC1_20240724	Quality Control	N/A	1.511e6	N/A	N/A	0.0000	N/A
Blank	Unknown	N/A	9.253e2	N/A	N/A	N/A	N/A
V3.0_MWMS_20240725_1	Unknown	2.08e6	4.177e6	4.12	43.7	N/A	4.748916e0
MWXS243064a_R1	Quality Control	N/A	1.316e6	N/A	N/A	0.0000	N/A
MWXS243064a_R2	Quality Control	N/A	1.226e6	N/A	N/A	0.0000	N/A
MWXS243064a_R3	Quality Control	N/A	1.274e6	N/A	N/A	0.0000	N/A
T24186682b_a	Unknown	N/A	1.337e6	N/A	N/A	N/A	N/A
T24186682b_b	Unknown	N/A	1.303e6	N/A	N/A	N/A	N/A
T24186682b_c	Unknown	N/A	1.287e6	N/A	N/A	N/A	N/A
T24186683b_a	Unknown	N/A	1.219e6	N/A	N/A	N/A	N/A
T24186683b_b	Unknown	N/A	1.264e6	N/A	N/A	N/A	N/A
T24186683b_c	Unknown	N/A	1.317e6	N/A	N/A	N/A	N/A
T24186684b_a	Unknown	N/A	1.242e6	N/A	N/A	N/A	N/A
T24186684b_b	Unknown	N/A	1.186e6	N/A	N/A	N/A	N/A
T24186684b_c	Unknown	N/A	1.175e6	N/A	N/A	N/A	N/A

Compound name: oT9G
Regression Equation: y = 0.10488 x + -1.22383e-5 (r = 0.99001) (weighting: 1 / x^2)

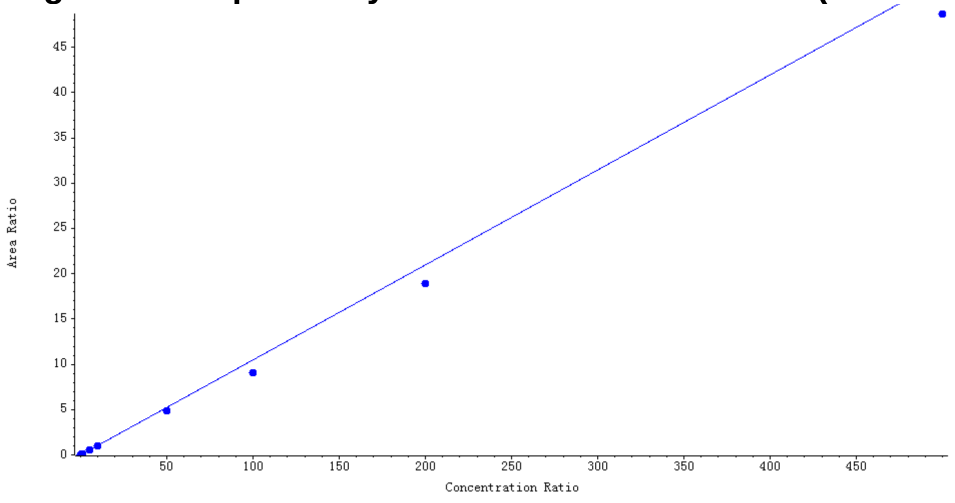

Peak Review

Blank
oT9G AREA:N/A S/N:N/A

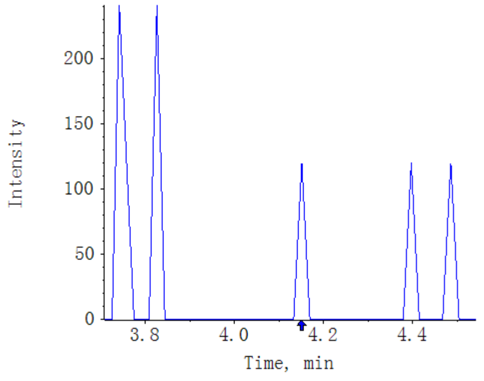

V3.0_MWMS_20240725_1
oT9G AREA:2.08e6 S/N:43.7

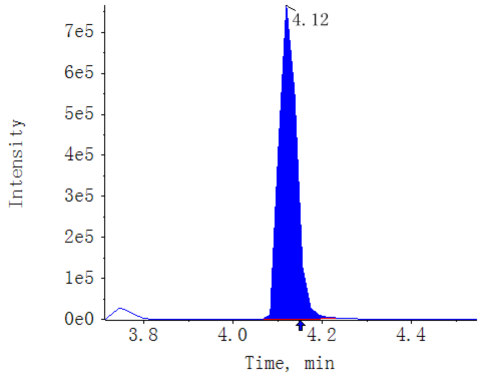

T24186682b_a
oT9G AREA:N/A S/N:N/A

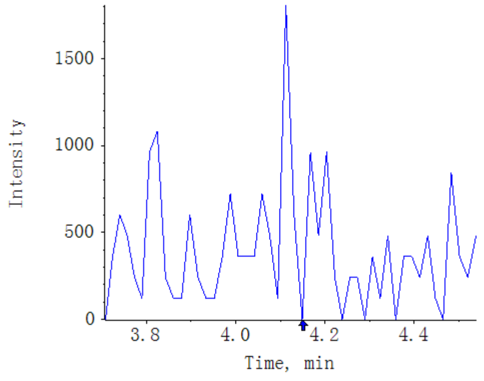

T24186682b_b
oT9G AREA:N/A S/N:N/A

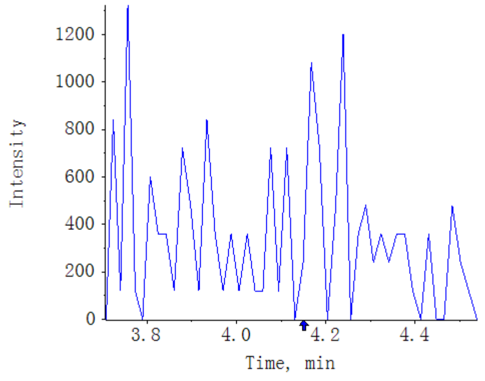

T24186682b_c
oT9G AREA:N/A S/N:N/A

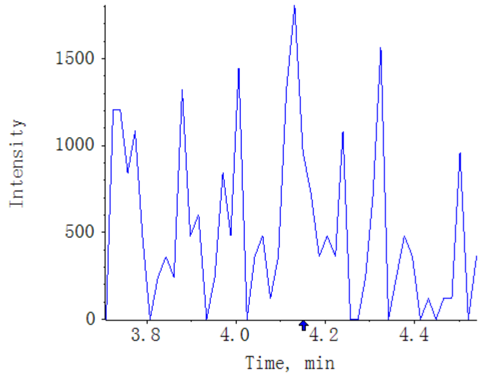

T24186683b_a
oT9G AREA:N/A S/N:N/A

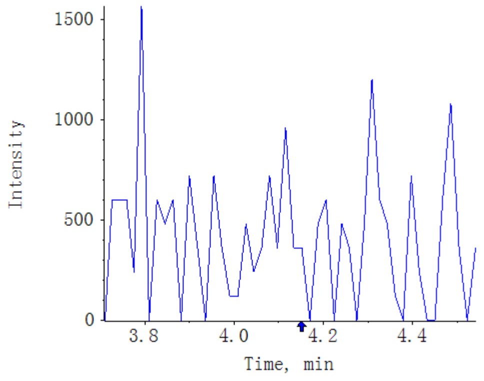

T24186683b_b
oT9G AREA:N/A S/N:N/A

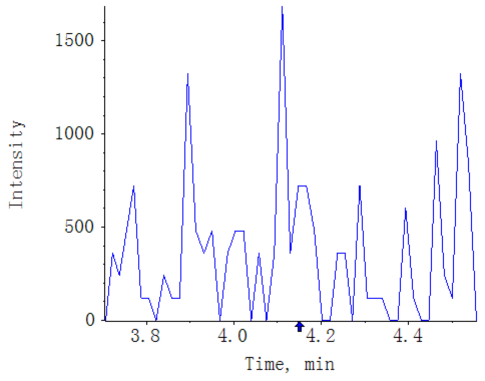

T24186683b_c
oT9G AREA:N/A S/N:N/A

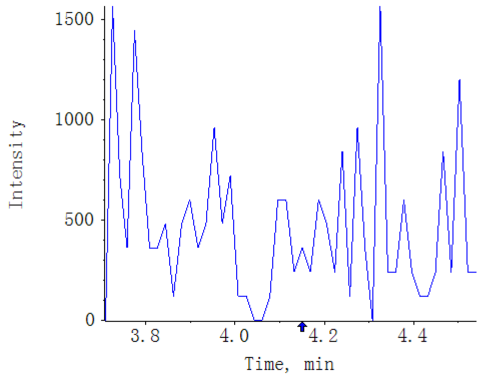

T24186684b_a
oT9G AREA:N/A S/N:N/A

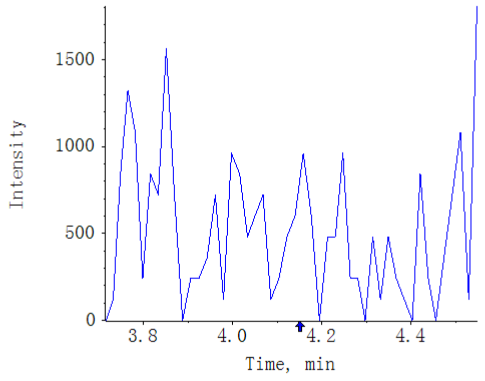

T24186684b_b
oT9G AREA:N/A S/N:N/A

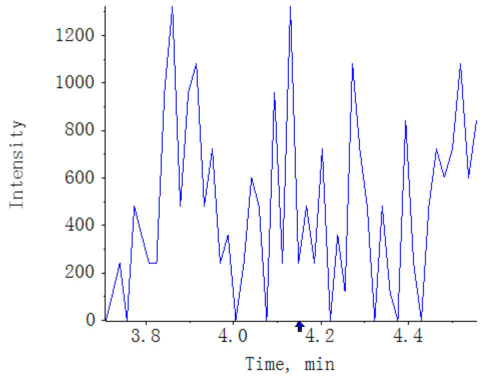

T24186684b_c
oT9G AREA:N/A S/N:N/A

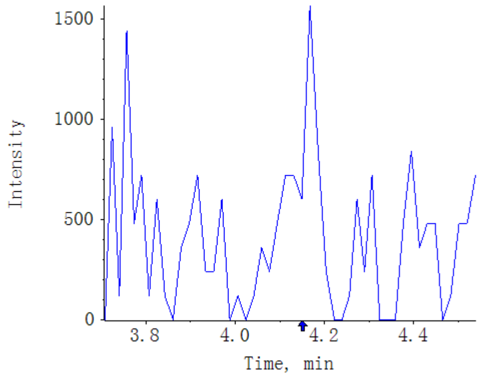

Result Table	MWXS-24-3064-a_9_WH6500-17_A20-3_V6.0_WSS_20240730	Algorithm Used	MQ4
Acquisition Method	ACC-PHs_V6.0_WH6500-17_CMY_20240521.dam	Instrument Name	Triple Quad 6500+ Low Mass
Project	N/A	Analytes QTY	109:44

Compound name: BAP9G (388.1 / 226.1)

Sample Name	Sample Type	Area (cps)	Is Area (cps)	RT (min)	S/N	Target Conc	Calculated Conc.()
STD_0.01ppb	Standard	1.06e4	3.065e6	4.28	6.8	0.0100	9.902165e-3
STD_0.05ppb	Standard	5.52e4	3.030e6	4.28	14.7	0.0500	5.157620e-2
STD_0.1ppb	Standard	1.10e5	3.037e6	4.29	14.8	0.1000	1.023173e-1
STD_0.5ppb	Standard	5.15e5	2.659e6	4.28	18.1	0.5000	5.478434e-1
STD_1ppb	Standard	9.28e5	2.814e6	4.28	27.3	1.0000	9.328121e-1
STD_5ppb	Standard	4.79e6	2.801e6	4.29	15.7	5.0000	4.835425e0
STD_10ppb	Standard	9.12e6	2.618e6	4.28	24.9	10.0000	9.849108e0
STD_50ppb	Standard	4.32e7	2.412e6	4.28	21.7	50.0000	5.069994e1
STD_100ppb	Standard	7.85e7	2.237e6	4.29	18.4	100.0000	9.926539e1
STD_200ppb	Standard	1.43e8	2.096e6	4.29	17.7	200.0000	1.935878e2
STD_500ppb	Standard	N/A	1.785e6	N/A	N/A	500.0000	N/A
V2.0_MW_RQC1_20240724	Quality Control	N/A	1.511e6	N/A	N/A	0.0000	N/A
Blank	Unknown	N/A	9.253e2	N/A	N/A	N/A	N/A
V3.0_MWMS_20240725_1	Unknown	6.59e6	4.177e6	4.28	7.9	N/A	4.461093e0
MWXS243064a_R1	Quality Control	N/A	1.316e6	N/A	N/A	0.0000	N/A
MWXS243064a_R2	Quality Control	N/A	1.226e6	N/A	N/A	0.0000	N/A
MWXS243064a_R3	Quality Control	N/A	1.274e6	N/A	N/A	0.0000	N/A
T24186682b_a	Unknown	N/A	1.337e6	N/A	N/A	N/A	N/A
T24186682b_b	Unknown	N/A	1.303e6	N/A	N/A	N/A	N/A
T24186682b_c	Unknown	N/A	1.287e6	N/A	N/A	N/A	N/A
T24186683b_a	Unknown	N/A	1.219e6	N/A	N/A	N/A	N/A
T24186683b_b	Unknown	N/A	1.264e6	N/A	N/A	N/A	N/A
T24186683b_c	Unknown	N/A	1.317e6	N/A	N/A	N/A	N/A
T24186684b_a	Unknown	N/A	1.242e6	N/A	N/A	N/A	N/A
T24186684b_b	Unknown	N/A	1.186e6	N/A	N/A	N/A	N/A
T24186684b_c	Unknown	N/A	1.175e6	N/A	N/A	N/A	N/A

Compound name: BAP9G
Regression Equation: y = 0.35352 x + -2.99609e-5 (r = 0.99893) (weighting: 1 / x^2)

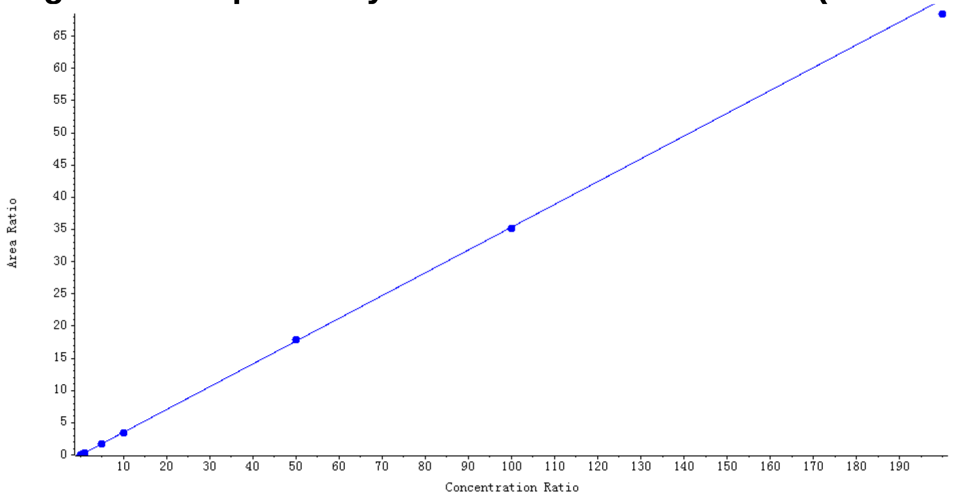

Peak Review

Blank
BAP9G AREA:N/A S/N:N/A

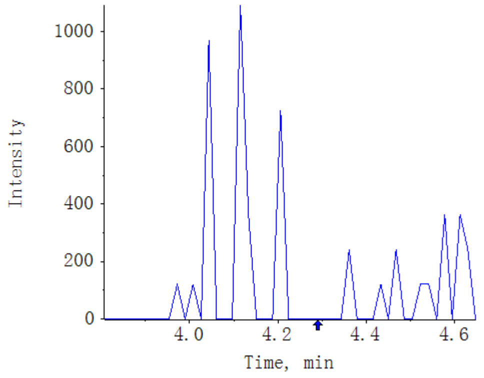

V3.0_MWMS_20240725_1
BAP9G AREA:6.59e6 S/N:7.9

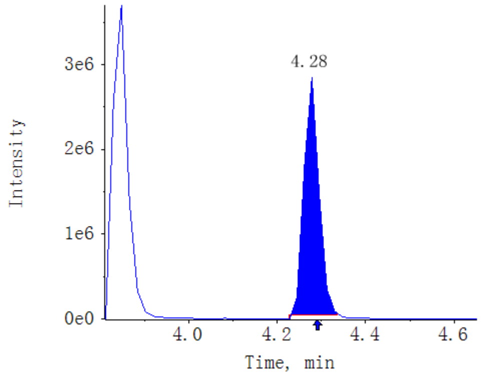

T24186682b_a
BAP9G AREA:N/A S/N:N/A

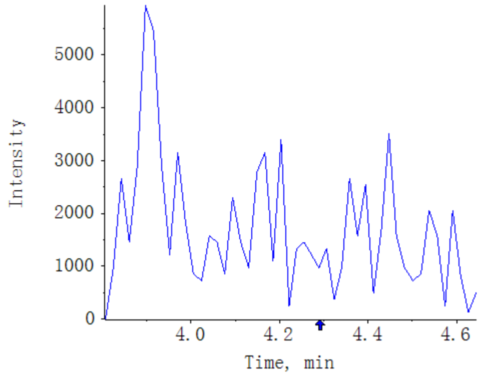

T24186682b_b
BAP9G AREA:N/A S/N:N/A

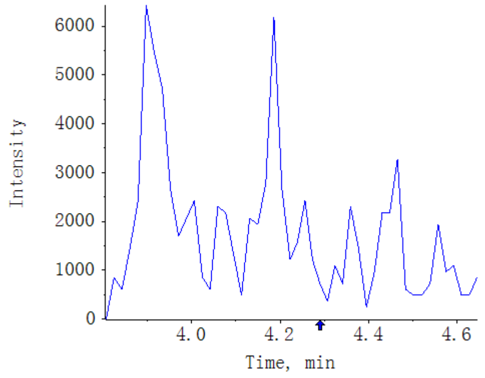

T24186682b_c
BAP9G AREA:N/A S/N:N/A

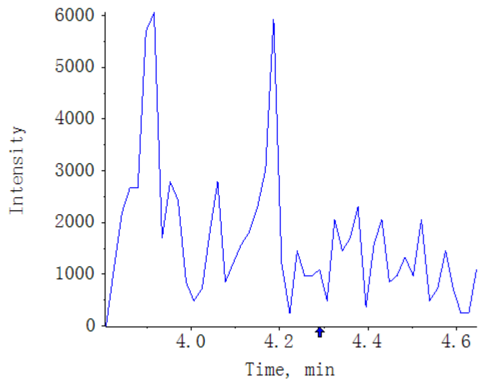

T24186683b_a
BAP9G AREA:N/A S/N:N/A

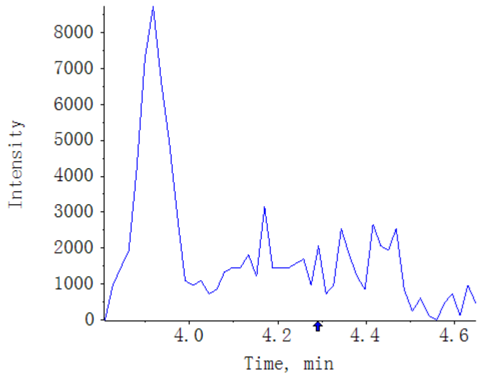

T24186683b_b
BAP9G AREA:N/A S/N:N/A

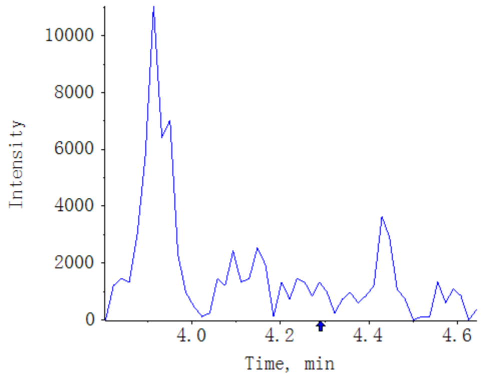

T24186683b_c
BAP9G AREA:N/A S/N:N/A

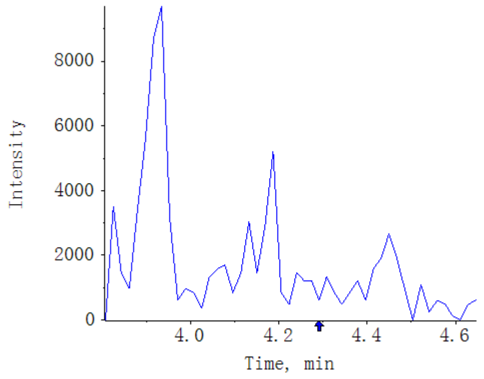

T24186684b_a
BAP9G AREA:N/A S/N:N/A

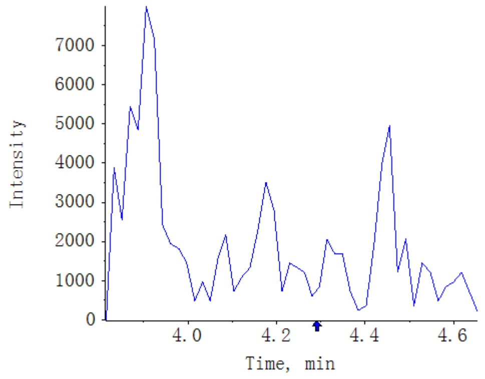

T24186684b_b
BAP9G AREA:N/A S/N:N/A

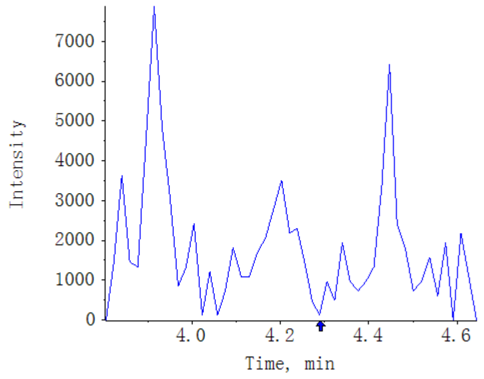

T24186684b_c
BAP9G AREA:N/A S/N:N/A

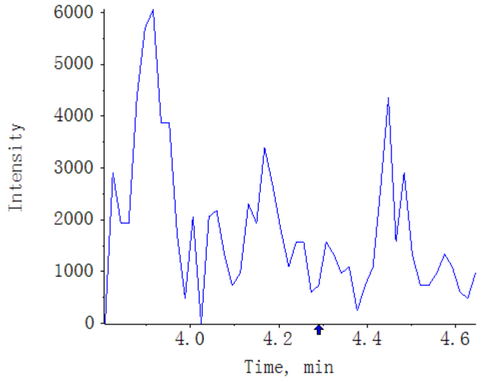

Result Table	MWXS-24-3064-a_9_WH6500-17_A20-3_V6.0_WSS_20240730	Algorithm Used	MQ4
Acquisition Method	ACC-PHs_V6.0_WH6500-17_CMY_20240521.dam	Instrument Name	Triple Quad 6500+ Low Mass
Project	N/A	Analytes QTY	109:45

Compound name: BAP7G (388.1 / 226.2)

Sample Name	Sample Type	Area (cps)	Is Area (cps)	RT (min)	S/N	Target Conc	Calculated Conc.()
STD_0.01ppb	Standard	1.27e4	3.065e6	3.84	14.6	0.0100	9.865886e-3
STD_0.05ppb	Standard	7.13e4	3.030e6	3.84	37.5	0.0500	4.965750e-2
STD_0.1ppb	Standard	1.65e5	3.037e6	3.86	66.9	0.1000	1.131229e-1
STD_0.5ppb	Standard	7.40e5	2.659e6	3.84	66.5	0.5000	5.726906e-1
STD_1ppb	Standard	1.21e6	2.814e6	3.84	51.1	1.0000	8.872523e-1
STD_5ppb	Standard	6.64e6	2.801e6	3.86	70.3	5.0000	4.867924e0
STD_10ppb	Standard	1.19e7	2.618e6	3.85	70.2	10.0000	9.335910e0
STD_50ppb	Standard	6.03e7	2.412e6	3.84	61.0	50.0000	5.134722e1
STD_100ppb	Standard	1.01e8	2.237e6	3.85	62.2	100.0000	9.222789e1
STD_200ppb	Standard	N/A	2.096e6	N/A	N/A	200.0000	N/A
STD_500ppb	Standard	N/A	1.785e6	N/A	N/A	500.0000	N/A
V2.0_MW_RQC1_20240724	Quality Control	N/A	1.511e6	N/A	N/A	0.0000	N/A
Blank	Unknown	N/A	9.253e2	N/A	N/A	N/A	N/A
V3.0_MWMS_20240725_1	Unknown	1.04e7	4.177e6	3.84	24.6	N/A	5.115087e0
MWXS243064a_R1	Quality Control	3.66e4	1.316e6	3.91	4.3	0.0000	5.839102e-2
MWXS243064a_R2	Quality Control	3.47e4	1.226e6	3.90	7.6	0.0000	5.951065e-2
MWXS243064a_R3	Quality Control	3.83e4	1.274e6	3.90	6.0	0.0000	6.310709e-2
T24186682b_a	Unknown	3.25e4	1.337e6	3.92	5.0	N/A	5.125434e-2
T24186682b_b	Unknown	3.04e4	1.303e6	3.91	5.2	N/A	4.929144e-2
T24186682b_c	Unknown	2.83e4	1.287e6	3.91	4.5	N/A	4.652198e-2
T24186683b_a	Unknown	3.89e4	1.219e6	3.91	5.9	N/A	6.681853e-2
T24186683b_b	Unknown	4.57e4	1.264e6	3.92	6.9	N/A	7.550866e-2
T24186683b_c	Unknown	4.86e4	1.317e6	3.92	6.6	N/A	7.714930e-2
T24186684b_a	Unknown	N/A	1.242e6	N/A	N/A	N/A	N/A
T24186684b_b	Unknown	N/A	1.186e6	N/A	N/A	N/A	N/A
T24186684b_c	Unknown	N/A	1.175e6	N/A	N/A	N/A	N/A

Compound name: BAP7G
Regression Equation: y = 0.48723 x + -6.59694e-4 (r = 0.99571) (weighting: 1 / x^2)

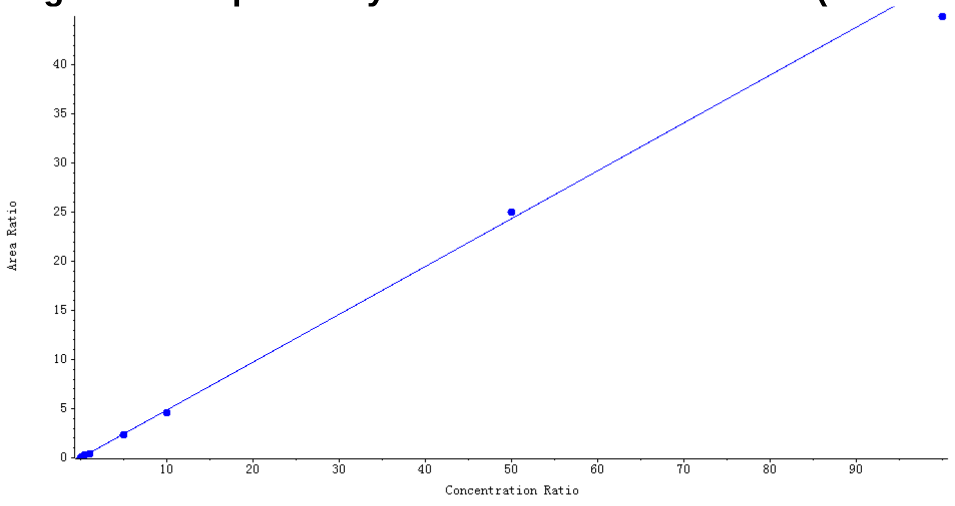

Peak Review

Blank
BAP7G AREA:N/A S/N:N/A

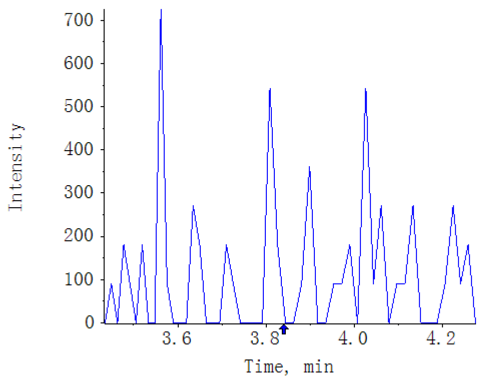

V3.0_MWMS_20240725_1
BAP7G AREA:1.04e7 S/N:24.6

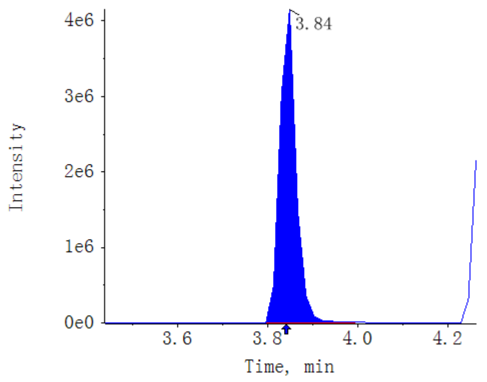

T24186682b_a
BAP7G AREA:3.25e4 S/N:5.0

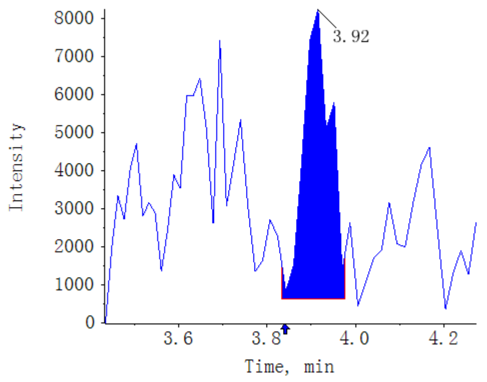

T24186682b_b
BAP7G AREA:3.04e4 S/N:5.2

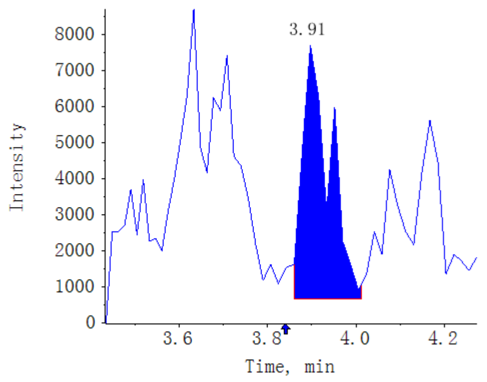

T24186682b_c
BAP7G AREA:2.83e4 S/N:4.5

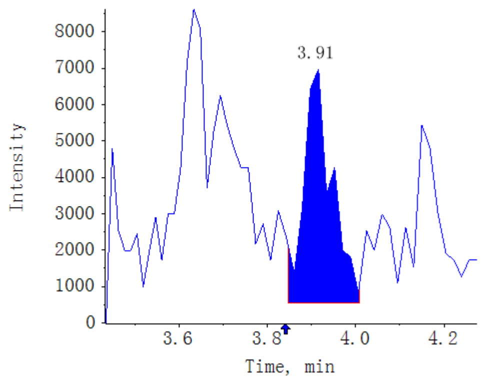

T24186683b_a
BAP7G AREA:3.89e4 S/N:5.9

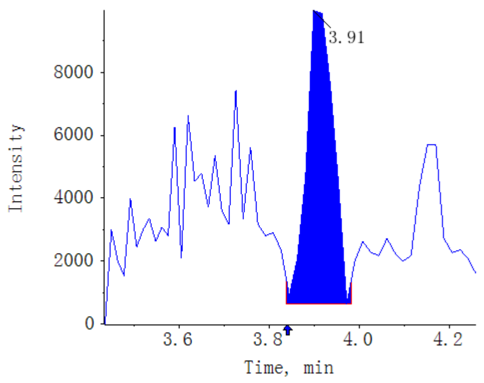

T24186683b_b
BAP7G AREA:4.57e4 S/N:6.9

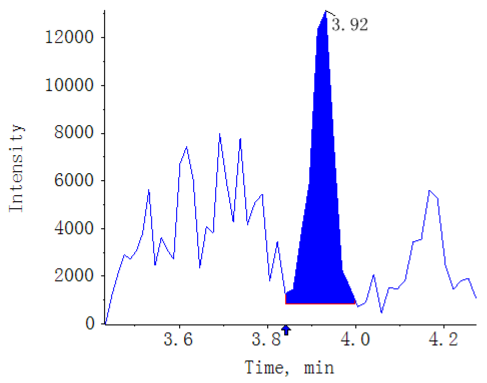

T24186683b_c
BAP7G AREA:4.86e4 S/N:6.6

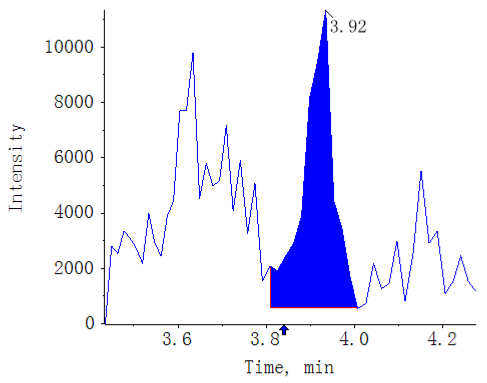

T24186684b_a
BAP7G AREA:N/A S/N:N/A

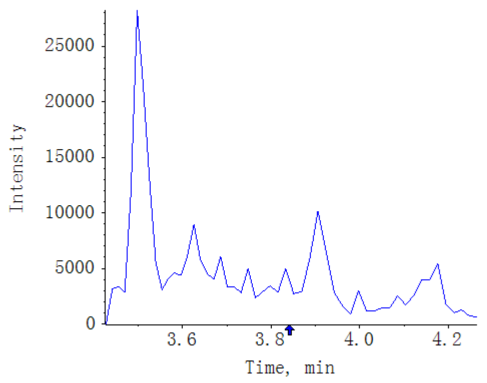

T24186684b_b
BAP7G AREA:N/A S/N:N/A

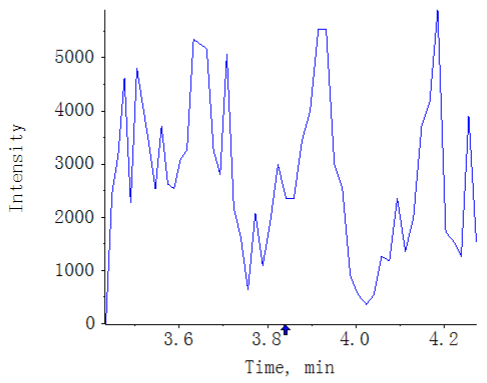

T24186684b_c
BAP7G AREA:N/A S/N:N/A

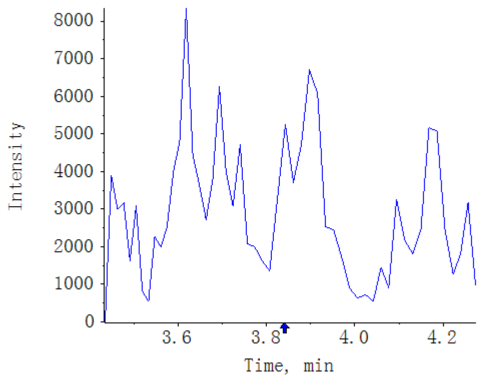

Result Table	MWXS-24-3064-a_9_WH6500-17_A20-3_V6.0_WSS_20240730	Algorithm Used	MQ4
Acquisition Method	ACC-PHs_V6.0_WH6500-17_CMY_20240521.dam	Instrument Name	Triple Quad 6500+ Low Mass
Project	N/A	Analytes QTY	109:46

Compound name: K9G (378.2 / 216.1)

Sample Name	Sample Type	Area (cps)	Is Area (cps)	RT (min)	S/N	Target Conc	Calculated Conc.()
STD_0.01ppb	Standard	5.82e3	3.065e6	3.82	10.6	0.0100	9.673076e-3
STD_0.05ppb	Standard	3.52e4	3.030e6	3.81	24.7	0.0500	5.422684e-2
STD_0.1ppb	Standard	7.44e4	3.037e6	3.82	39.9	0.1000	1.132354e-1
STD_0.5ppb	Standard	3.37e5	2.659e6	3.81	43.7	0.5000	5.817861e-1
STD_1ppb	Standard	5.75e5	2.814e6	3.81	47.3	1.0000	9.361980e-1
STD_5ppb	Standard	3.07e6	2.801e6	3.83	56.0	5.0000	5.014087e0
STD_10ppb	Standard	5.35e6	2.618e6	3.81	59.8	10.0000	9.352534e0
STD_50ppb	Standard	2.57e7	2.412e6	3.81	52.2	50.0000	4.880588e1
STD_100ppb	Standard	3.92e7	2.237e6	3.82	55.1	100.0000	8.018434e1
STD_200ppb	Standard	N/A	2.096e6	N/A	N/A	200.0000	N/A
STD_500ppb	Standard	N/A	1.785e6	N/A	N/A	500.0000	N/A
V2.0_MW_RQC1_20240724	Quality Control	5.25e4	1.511e6	3.82	9.2	0.0000	1.600759e-1
Blank	Unknown	N/A	9.253e2	N/A	N/A	N/A	N/A
V3.0_MWMS_20240725_1	Unknown	6.42e6	4.177e6	3.82	75.4	N/A	7.043862e0
MWXS243064a_R1	Quality Control	N/A	1.316e6	N/A	N/A	0.0000	N/A
MWXS243064a_R2	Quality Control	N/A	1.226e6	N/A	N/A	0.0000	N/A
MWXS243064a_R3	Quality Control	N/A	1.274e6	N/A	N/A	0.0000	N/A
T24186682b_a	Unknown	2.31e5	1.337e6	3.75	5.4	N/A	7.916766e-1
T24186682b_b	Unknown	2.11e5	1.303e6	3.73	5.6	N/A	7.420215e-1
T24186682b_c	Unknown	1.97e5	1.287e6	3.74	4.8	N/A	7.006659e-1
T24186683b_a	Unknown	N/A	1.219e6	N/A	N/A	N/A	N/A
T24186683b_b	Unknown	N/A	1.264e6	N/A	N/A	N/A	N/A
T24186683b_c	Unknown	N/A	1.317e6	N/A	N/A	N/A	N/A
T24186684b_a	Unknown	N/A	1.242e6	N/A	N/A	N/A	N/A
T24186684b_b	Unknown	N/A	1.186e6	N/A	N/A	N/A	N/A
T24186684b_c	Unknown	N/A	1.175e6	N/A	N/A	N/A	N/A

Compound name: K9G
Regression Equation: y = 0.21838 x + -2.14647e-4 (r = 0.99319) (weighting: 1 / x^2)

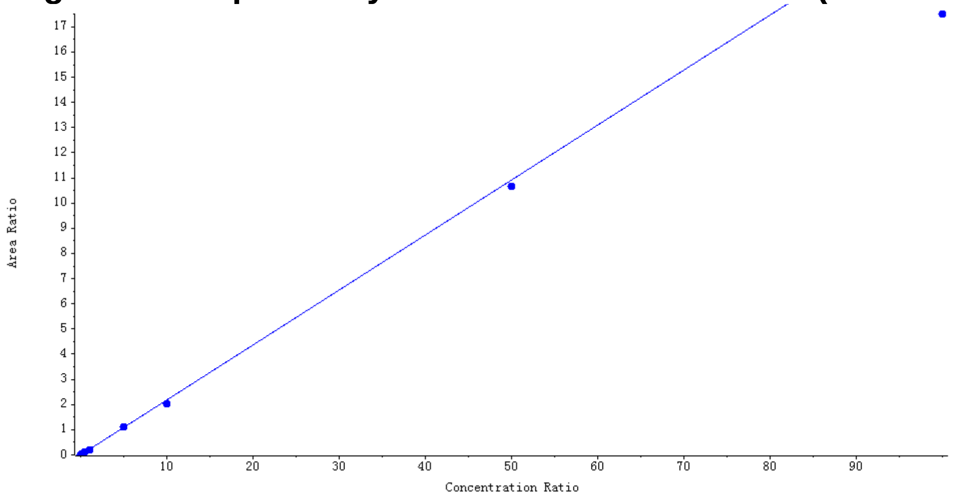

Peak Review

Blank
K9G AREA:N/A S/N:N/A

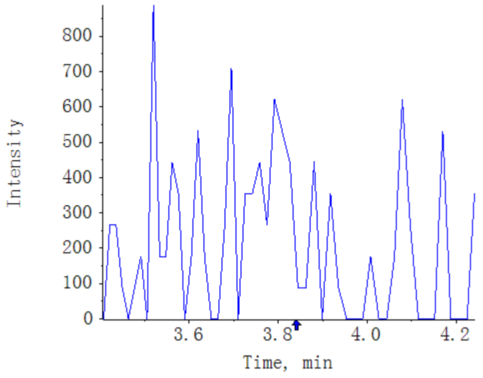

V3.0_MWMS_20240725_1
K9G AREA:6.42e6 S/N:75.4

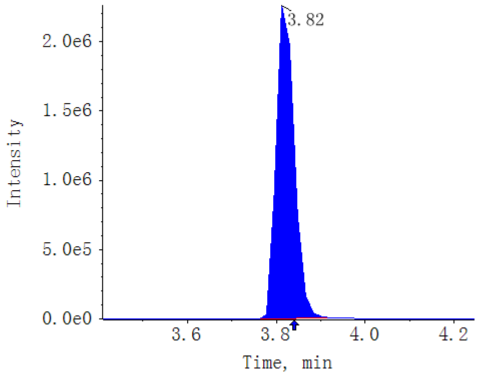

T24186682b_a
K9G AREA:2.31e5 S/N:5.4

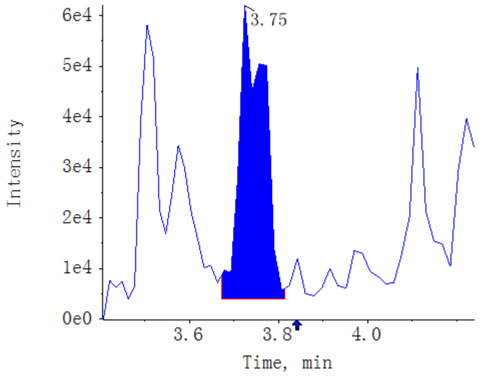

T24186682b_b
K9G AREA:2.11e5 S/N:5.6

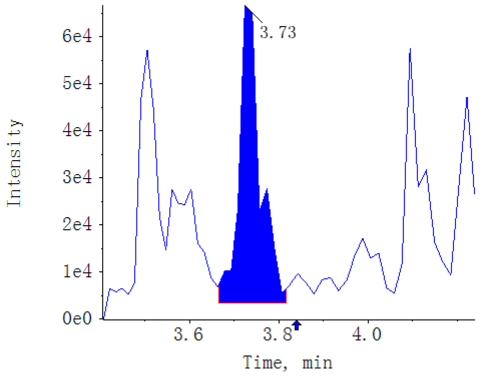

T24186682b_c
K9G AREA:1.97e5 S/N:4.8

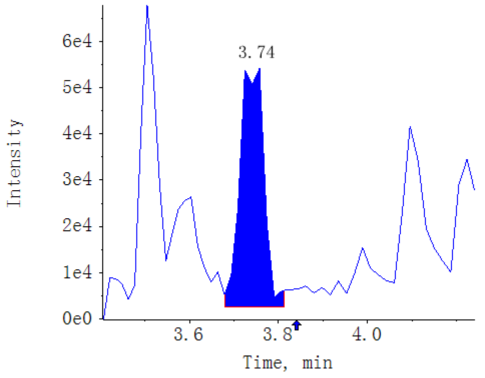

T24186683b_a
K9G AREA:N/A S/N:N/A

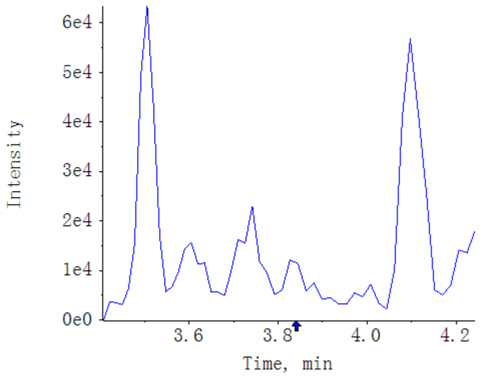

T24186683b_b
K9G AREA:N/A S/N:N/A

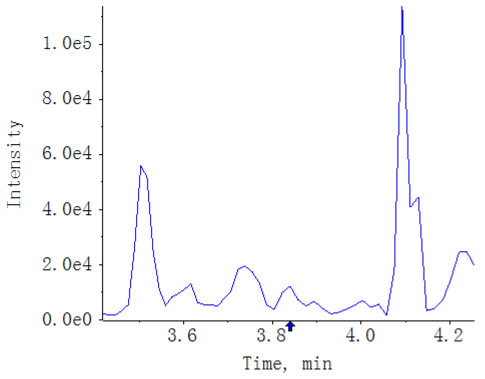

T24186683b_c
K9G AREA:N/A S/N:N/A

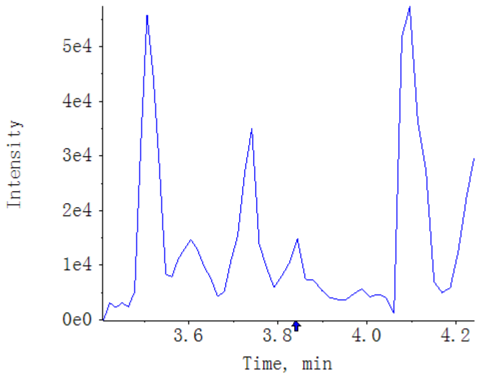

T24186684b_a
K9G AREA:N/A S/N:N/A

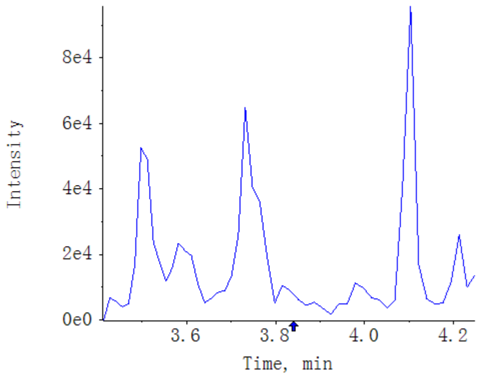

T24186684b_b
K9G AREA:N/A S/N:N/A

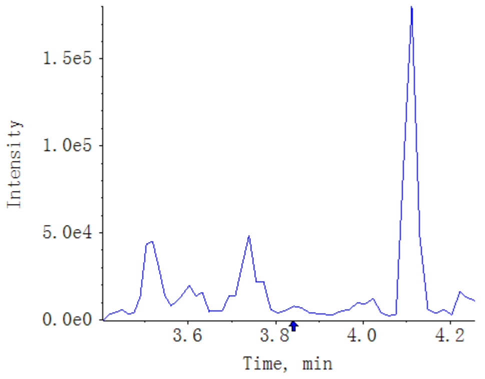

T24186684b_c
K9G AREA:N/A S/N:N/A

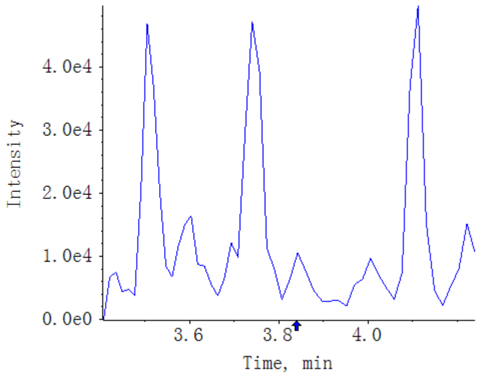

Result Table	MWXS-24-3064-a_9_WH6500-17_A20-3_V6.0_WSS_20240730	Algorithm Used	MQ4
Acquisition Method	ACC-PHs_V6.0_WH6500-17_CMY_20240521.dam	Instrument Name	Triple Quad 6500+ Low Mass
Project	N/A	Analytes QTY	109:47

Compound name: 2MeSiP (250.1 / 182.1)

Sample Name	Sample Type	Area (cps)	Is Area (cps)	RT (min)	S/N	Target Conc	Calculated Conc.()
STD_0.01ppb	Standard	2.31e3	1.557e7	5.90	10.6	0.0100	9.555072e-3
STD_0.05ppb	Standard	1.60e4	1.530e7	5.90	33.2	0.0500	6.266794e-2
STD_0.1ppb	Standard	2.64e4	1.672e7	5.92	42.2	0.1000	9.426658e-2
STD_0.5ppb	Standard	1.26e5	1.486e7	5.91	62.3	0.5000	5.000440e-1
STD_1ppb	Standard	2.30e5	1.459e7	5.91	33.2	1.0000	9.341372e-1
STD_5ppb	Standard	1.34e6	1.406e7	5.92	38.3	5.0000	5.655513e0
STD_10ppb	Standard	2.32e6	1.434e7	5.91	44.8	10.0000	9.580437e0
STD_50ppb	Standard	1.13e7	1.335e7	5.91	52.8	50.0000	4.992923e1
STD_100ppb	Standard	1.99e7	1.245e7	5.92	42.7	100.0000	9.441574e1
STD_200ppb	Standard	3.85e7	1.164e7	5.91	48.1	200.0000	1.954883e2
STD_500ppb	Standard	8.22e7	1.074e7	5.91	36.9	500.0000	4.524566e2
V2.0_MW_RQC1_20240724	Quality Control	N/A	6.875e6	N/A	N/A	0.0000	N/A
Blank	Unknown	N/A	1.497e4	N/A	N/A	N/A	N/A
V3.0_MWMS_20240725_1	Unknown	1.66e6	1.210e7	5.89	41.9	N/A	8.115469e0
MWXS243064a_R1	Quality Control	N/A	1.200e7	N/A	N/A	0.0000	N/A
MWXS243064a_R2	Quality Control	N/A	1.264e7	N/A	N/A	0.0000	N/A
MWXS243064a_R3	Quality Control	N/A	1.208e7	N/A	N/A	0.0000	N/A
T24186682b_a	Unknown	N/A	1.223e7	N/A	N/A	N/A	N/A
T24186682b_b	Unknown	N/A	1.220e7	N/A	N/A	N/A	N/A
T24186682b_c	Unknown	N/A	1.298e7	N/A	N/A	N/A	N/A
T24186683b_a	Unknown	N/A	1.222e7	N/A	N/A	N/A	N/A
T24186683b_b	Unknown	N/A	1.240e7	N/A	N/A	N/A	N/A
T24186683b_c	Unknown	N/A	1.286e7	N/A	N/A	N/A	N/A
T24186684b_a	Unknown	N/A	1.216e7	N/A	N/A	N/A	N/A
T24186684b_b	Unknown	N/A	1.270e7	N/A	N/A	N/A	N/A
T24186684b_c	Unknown	N/A	1.186e7	N/A	N/A	N/A	N/A

Compound name: 2MeSiP
Regression Equation: y = 0.01692 x + -1.34245e-5 (r = 0.99438) (weighting: 1 / x^2)

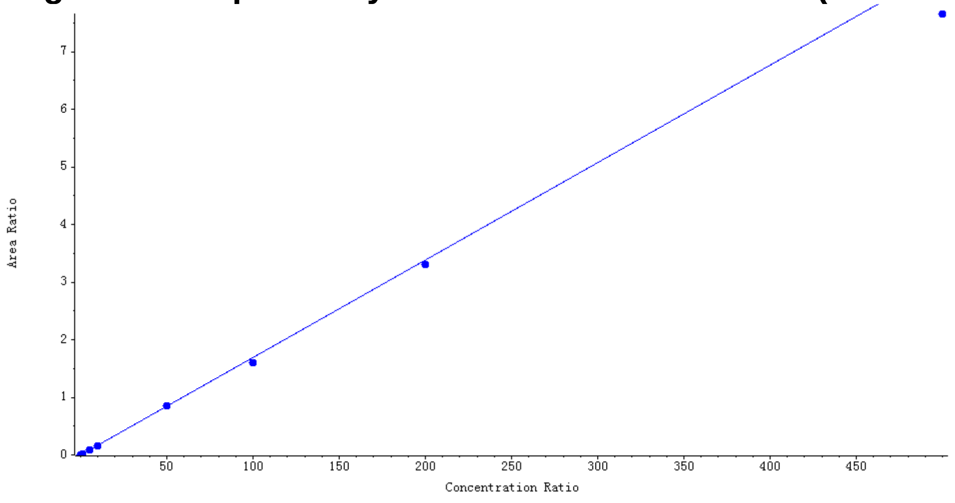

Peak Review

Blank

2MeSiP AREA:N/A S/N:N/A

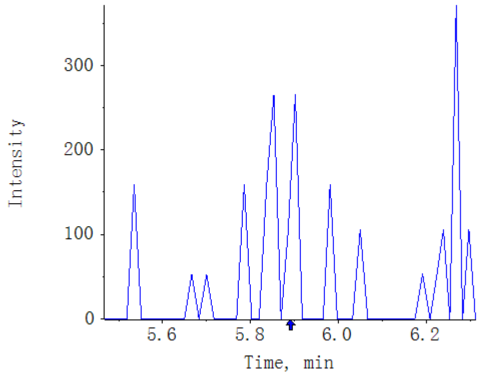

V3.0_MWMS_20240725_1

2MeSiP AREA:1.66e6 S/N:41.9

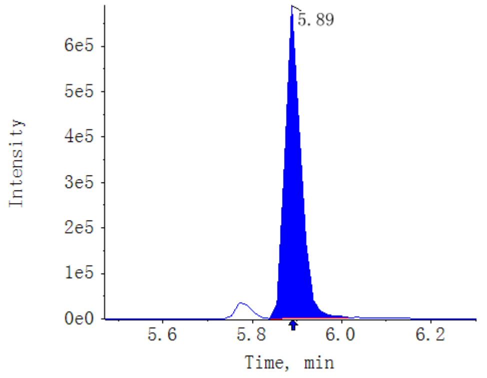

T24186682b_a

2MeSiP AREA:N/A S/N:N/A

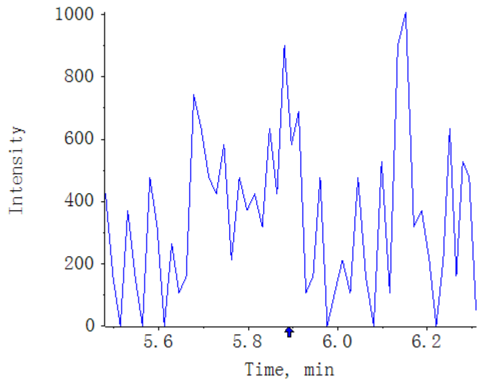

T24186682b_b

2MeSiP AREA:N/A S/N:N/A

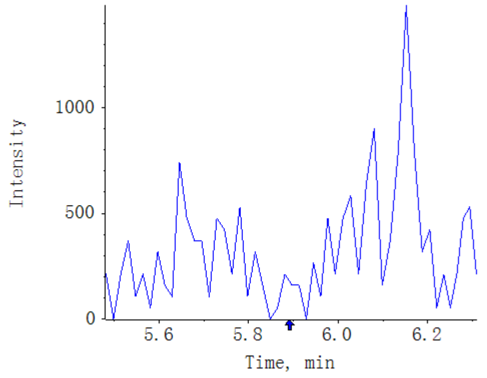

T24186682b_c

2MeSiP AREA:N/A S/N:N/A

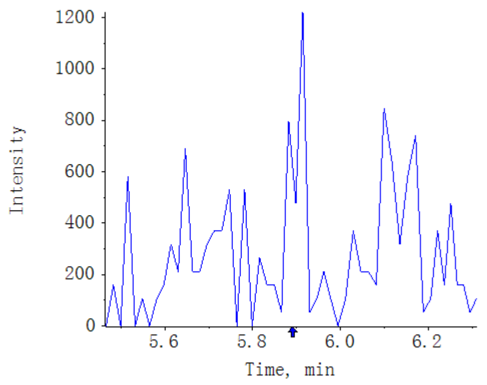

T24186683b_a

2MeSiP AREA:N/A S/N:N/A

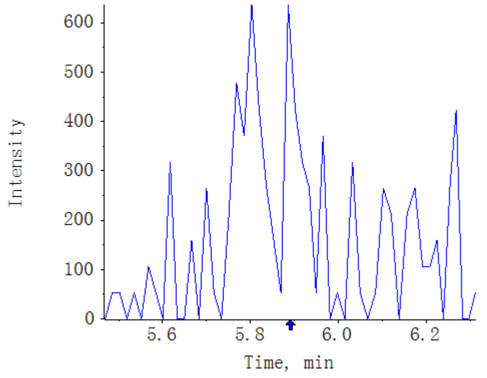

T24186683b_b

2MeSiP AREA:N/A S/N:N/A

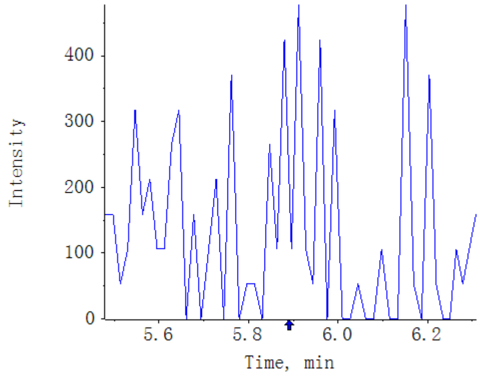

T24186683b_c

2MeSiP AREA:N/A S/N:N/A

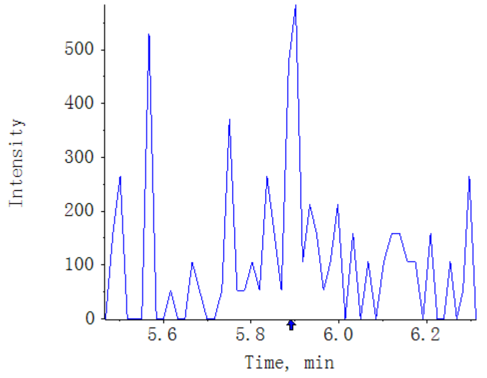

T24186684b_a

2MeSiP AREA:N/A S/N:N/A

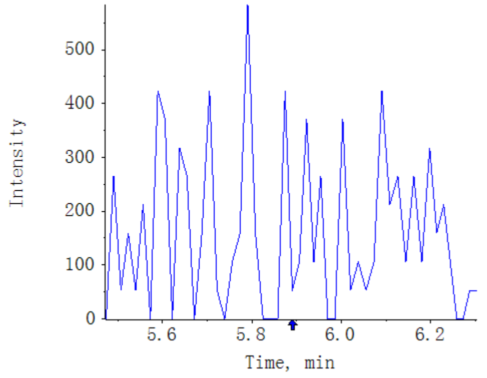

T24186684b_b

2MeSiP AREA:N/A S/N:N/A

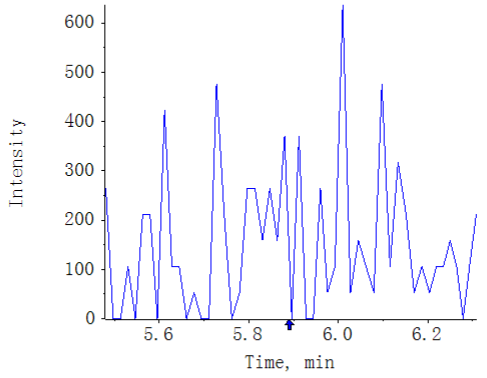

T24186684b_c

2MeSiP AREA:N/A S/N:N/A

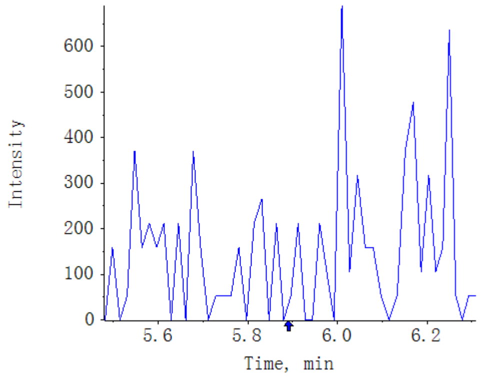

Result Table	MWXS-24-3064-a_9_WH6500-17_A20-3_V6.0_WSS_20240730	Algorithm Used	MQ4
Acquisition Method	ACC-PHs_V6.0_WH6500-17_CMY_20240521.dam	Instrument Name	Triple Quad 6500+ Low Mass
Project	N/A	Analytes QTY	109:48

Compound name: 2MeScZ (266.2 / 182.2)

Sample Name	Sample Type	Area (cps)	Is Area (cps)	RT (min)	S/N	Target Conc	Calculated Conc.()
STD_0.01ppb	Standard	N/A	6.534e6	N/A	N/A	0.0100	N/A
STD_0.05ppb	Standard	8.90e3	7.105e6	4.56	28.4	0.0500	4.910369e-2
STD_0.1ppb	Standard	1.96e4	6.945e6	4.57	22.9	0.1000	1.039022e-1
STD_0.5ppb	Standard	8.44e4	5.879e6	4.56	41.1	0.5000	5.052769e-1
STD_1ppb	Standard	1.51e5	5.871e6	4.56	37.3	1.0000	9.019796e-1
STD_5ppb	Standard	9.01e5	5.223e6	4.58	61.4	5.0000	6.010441e0
STD_10ppb	Standard	1.55e6	5.065e6	4.56	41.7	10.0000	1.067727e1
STD_50ppb	Standard	6.78e6	4.847e6	4.56	52.1	50.0000	4.872176e1
STD_100ppb	Standard	1.18e7	4.595e6	4.57	47.5	100.0000	8.914646e1
STD_200ppb	Standard	2.24e7	4.227e6	4.56	44.4	200.0000	1.841081e2
STD_500ppb	Standard	4.70e7	3.238e6	4.56	45.3	500.0000	5.050577e2
V2.0_MW_RQC1_20240724	Quality Control	N/A	5.942e6	N/A	N/A	0.0000	N/A
Blank	Unknown	N/A	2.845e3	N/A	N/A	N/A	N/A
V3.0_MWMS_20240725_1	Unknown	1.43e6	1.146e7	4.56	30.0	N/A	4.337293e0
MWXS243064a_R1	Quality Control	N/A	6.293e6	N/A	N/A	0.0000	N/A
MWXS243064a_R2	Quality Control	N/A	6.413e6	N/A	N/A	0.0000	N/A
MWXS243064a_R3	Quality Control	N/A	6.529e6	N/A	N/A	0.0000	N/A
T24186682b_a	Unknown	N/A	6.437e6	N/A	N/A	N/A	N/A
T24186682b_b	Unknown	N/A	6.323e6	N/A	N/A	N/A	N/A
T24186682b_c	Unknown	N/A	6.686e6	N/A	N/A	N/A	N/A
T24186683b_a	Unknown	N/A	6.369e6	N/A	N/A	N/A	N/A
T24186683b_b	Unknown	N/A	6.536e6	N/A	N/A	N/A	N/A
T24186683b_c	Unknown	N/A	6.791e6	N/A	N/A	N/A	N/A
T24186684b_a	Unknown	N/A	5.743e6	N/A	N/A	N/A	N/A
T24186684b_b	Unknown	N/A	6.364e6	N/A	N/A	N/A	N/A
T24186684b_c	Unknown	N/A	6.178e6	N/A	N/A	N/A	N/A

Compound name: 2MeScZ
Regression Equation: y = 0.02873 x + -1.58492e-4 (r = 0.99517) (weighting: 1 / x^2)

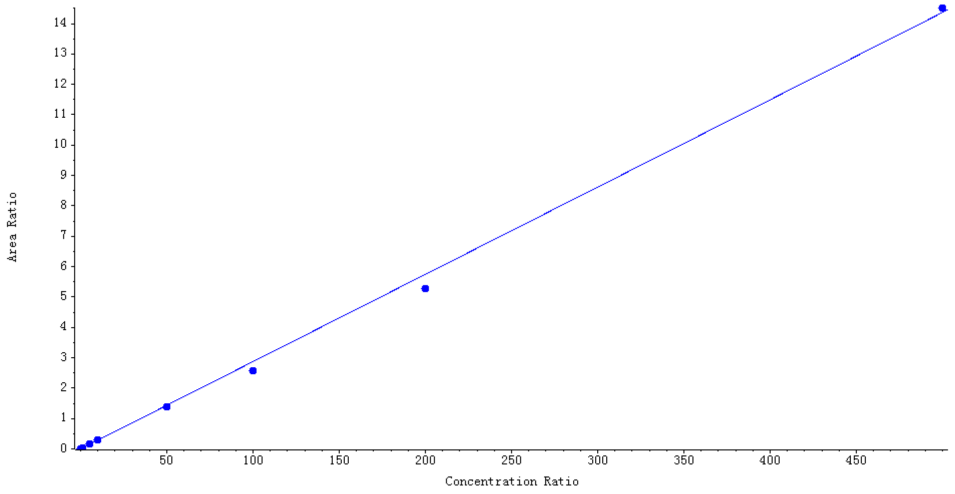

Peak Review

Blank
2MeScZ AREA:N/A S/N:N/A

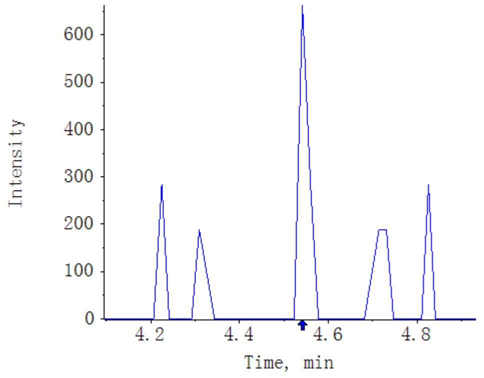

V3.0_MWMS_20240725_1
2MeScZ AREA:1.43e6 S/N:30.0

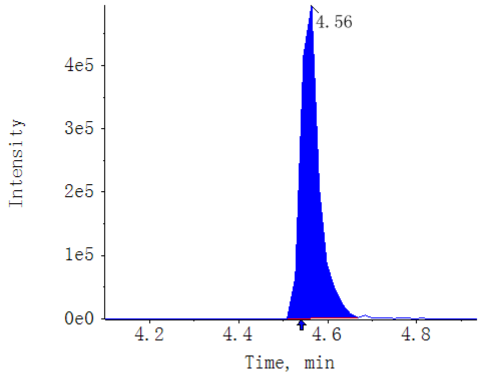

T24186682b_a
2MeScZ AREA:N/A S/N:N/A

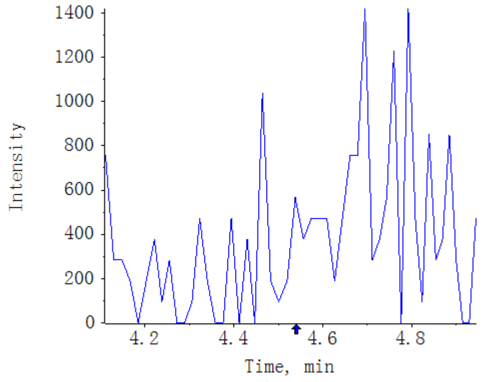

T24186682b_b
2MeScZ AREA:N/A S/N:N/A

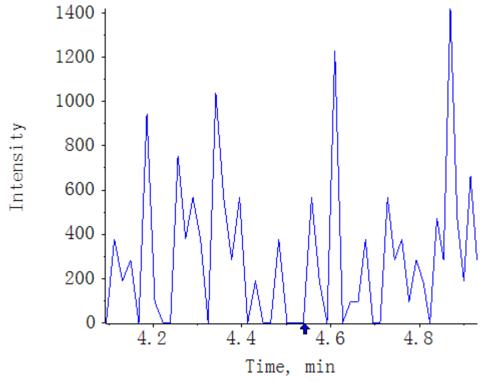

T24186682b_c
2MeScZ AREA:N/A S/N:N/A

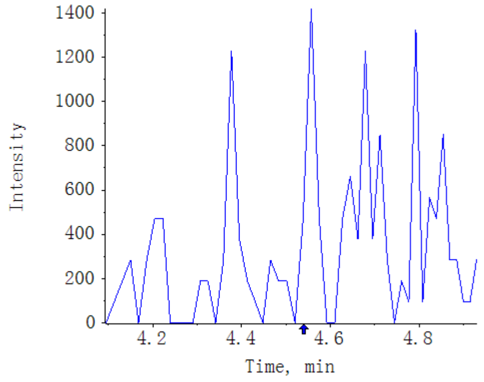

T24186683b_a
2MeScZ AREA:N/A S/N:N/A

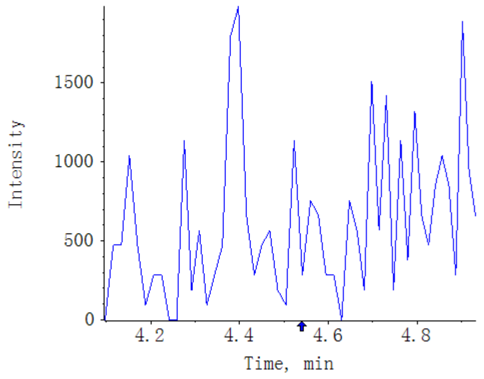

T24186683b_b
2MeScZ AREA:N/A S/N:N/A

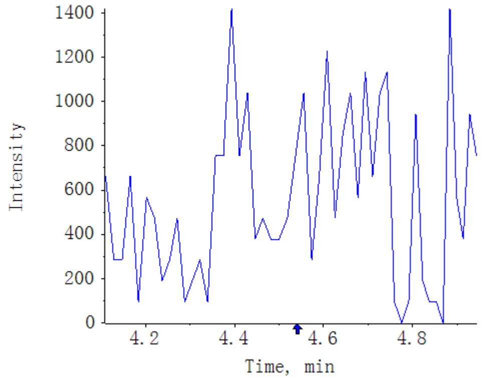

T24186683b_c
2MeScZ AREA:N/A S/N:N/A

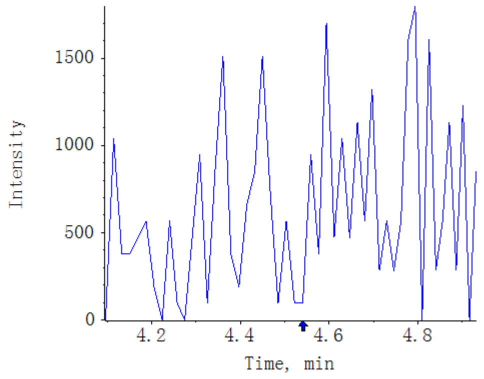

T24186684b_a
2MeScZ AREA:N/A S/N:N/A

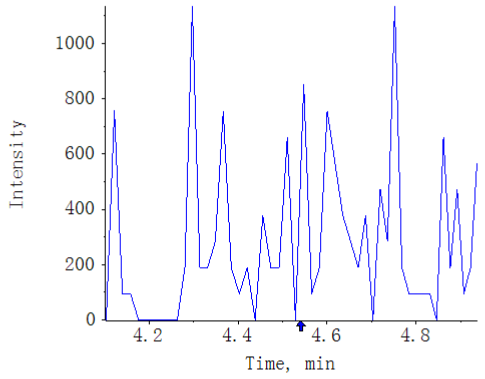

T24186684b_b
2MeScZ AREA:N/A S/N:N/A

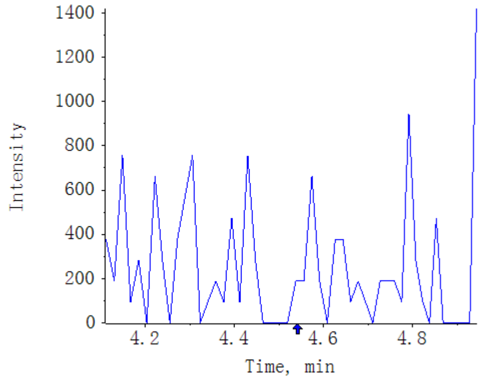

T24186684b_c
2MeScZ AREA:N/A S/N:N/A

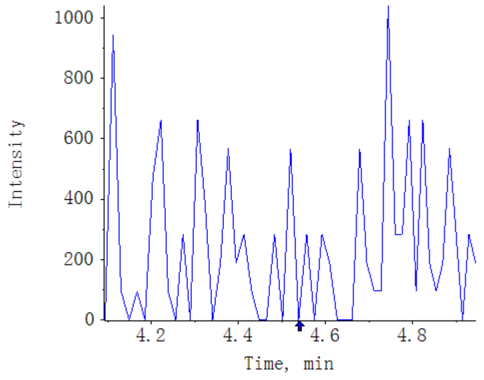

Result Table	MWXS-24-3064-a_9_WH6500-17_A20-3_V6.0_WSS_20240730	Algorithm Used	MQ4
Acquisition Method	ACC-PHs_V6.0_WH6500-17_CMY_20240521.dam	Instrument Name	Triple Quad 6500+ Low Mass
Project	N/A	Analytes QTY	109:49

Compound name: 2MeScZR (398.0 / 266.1)

Sample Name	Sample Type	Area (cps)	Is Area (cps)	RT (min)	S/N	Target Conc	Calculated Conc.()
STD_0.01ppb	Standard	3.45e4	1.360e7	4.62	21.9	0.0100	9.825701e-3
STD_0.05ppb	Standard	1.20e5	1.429e7	4.62	49.6	0.0500	5.236729e-2
STD_0.1ppb	Standard	2.14e5	1.337e7	4.63	46.0	0.1000	1.071117e-1
STD_0.5ppb	Standard	9.39e5	1.198e7	4.62	62.1	0.5000	5.585082e-1
STD_1ppb	Standard	1.47e6	1.273e7	4.63	71.8	1.0000	8.267153e-1
STD_5ppb	Standard	9.21e6	1.179e7	4.63	65.5	5.0000	5.638265e0
STD_10ppb	Standard	1.56e7	1.134e7	4.62	67.0	10.0000	9.953362e0
STD_50ppb	Standard	6.51e7	9.254e6	4.61	48.7	50.0000	5.084523e1
STD_100ppb	Standard	1.02e8	7.887e6	4.63	55.7	100.0000	9.359087e1
STD_200ppb	Standard	1.57e8	6.442e6	4.62	38.8	200.0000	1.758866e2
STD_500ppb	Standard	N/A	5.536e6	N/A	N/A	500.0000	N/A
V2.0_MW_RQC1_20240724	Quality Control	8.79e4	3.128e6	4.61	27.9	0.0000	1.947183e-1
Blank	Unknown	N/A	1.529e3	N/A	N/A	N/A	N/A
V3.0_MWMS_20240725_1	Unknown	1.06e7	1.554e7	4.61	64.8	N/A	4.917915e0
MWXS243064a_R1	Quality Control	N/A	3.227e6	N/A	N/A	0.0000	N/A
MWXS243064a_R2	Quality Control	N/A	3.229e6	N/A	N/A	0.0000	N/A
MWXS243064a_R3	Quality Control	N/A	3.206e6	N/A	N/A	0.0000	N/A
T24186682b_a	Unknown	1.92e4	3.150e6	4.59	6.9	N/A	3.547192e-2
T24186682b_b	Unknown	1.86e4	3.173e6	4.60	5.8	N/A	3.387681e-2
T24186682b_c	Unknown	2.12e4	3.249e6	4.60	3.8	N/A	3.852359e-2
T24186683b_a	Unknown	N/A	3.447e6	N/A	N/A	N/A	N/A
T24186683b_b	Unknown	N/A	3.437e6	N/A	N/A	N/A	N/A
T24186683b_c	Unknown	N/A	3.620e6	N/A	N/A	N/A	N/A
T24186684b_a	Unknown	1.58e4	2.869e6	4.61	5.0	N/A	3.118479e-2
T24186684b_b	Unknown	1.70e4	3.108e6	4.62	5.3	N/A	3.097635e-2
T24186684b_c	Unknown	1.59e4	3.185e6	4.63	4.7	N/A	2.755095e-2

Compound name: 2MeScZR
Regression Equation: y = 0.13834 x + 0.00118 (r = 0.99483) (weighting: 1 / x^2)

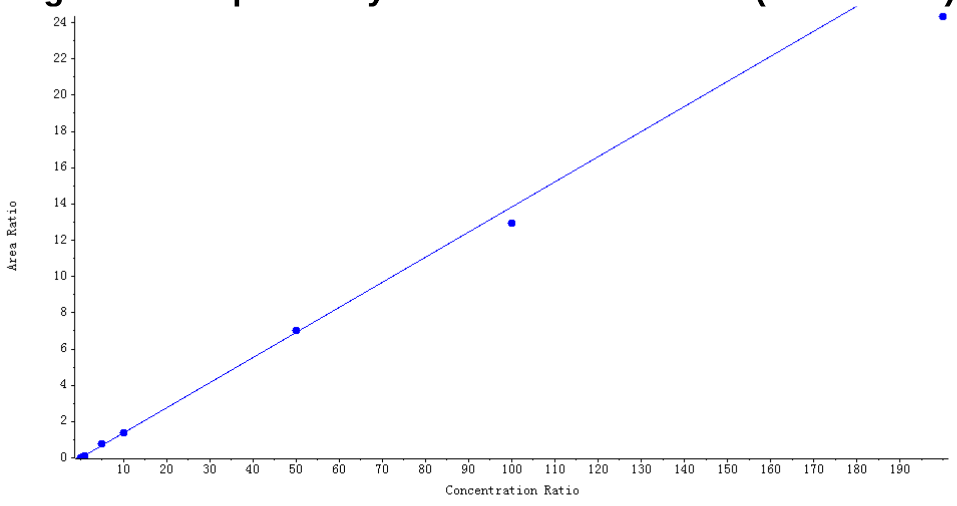

Peak Review

Blank
2MeScZR AREA:N/A S/N:N/A

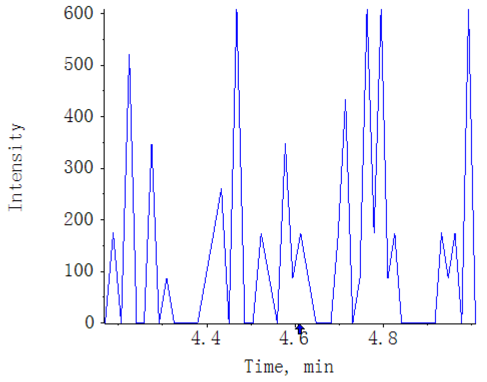

V3.0_MWMS_20240725_1
2MeScZR AREA:1.06e7 S/N:64.8

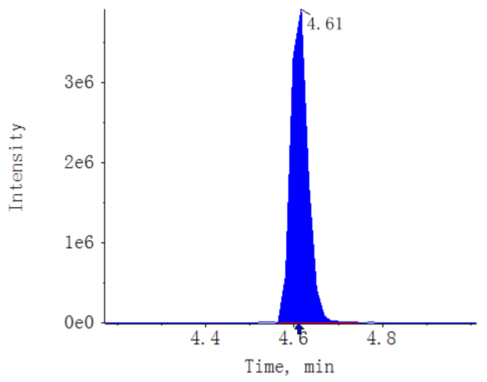

T24186682b_a
2MeScZR AREA:1.92e4 S/N:6.9

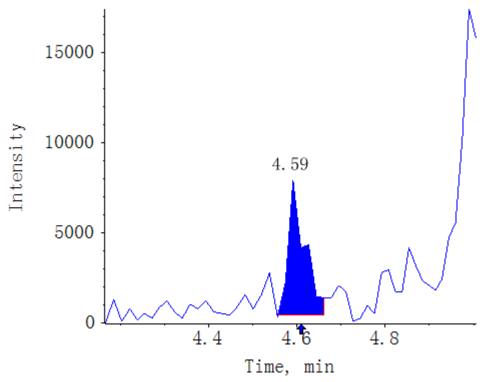

T24186682b_b
2MeScZR AREA:1.86e4 S/N:5.8

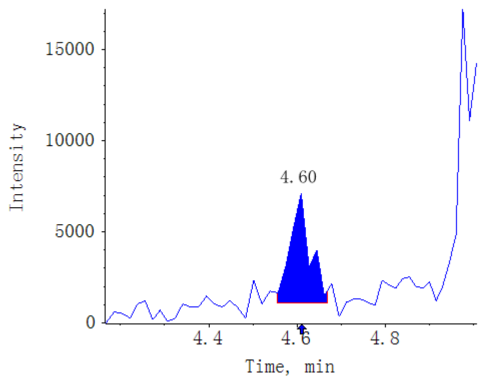

T24186682b_c
2MeScZR AREA:2.12e4 S/N:3.8

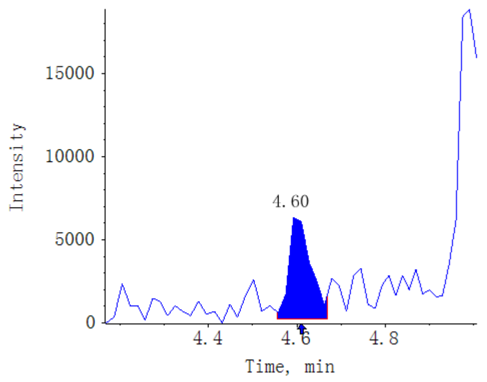

T24186683b_a
2MeScZR AREA:N/A S/N:N/A

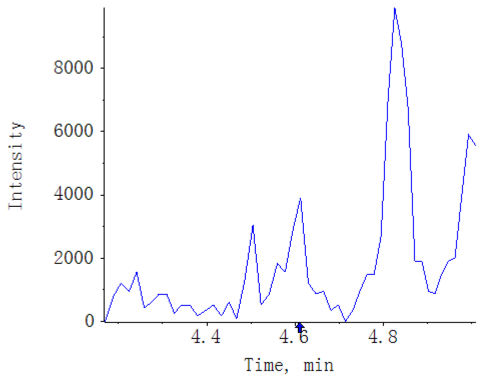

T24186683b_b
2MeScZR AREA:N/A S/N:N/A

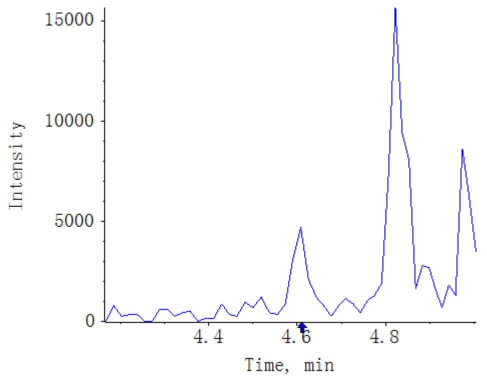

T24186683b_c
2MeScZR AREA:N/A S/N:N/A

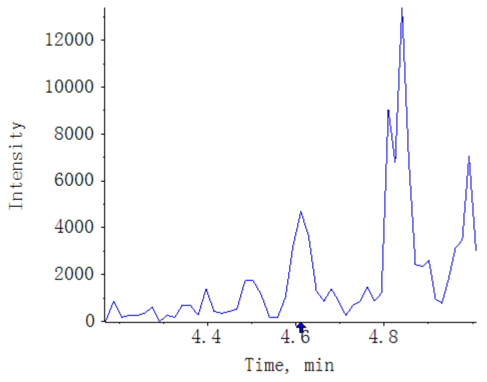

T24186684b_a
2MeScZR AREA:1.58e4 S/N:5.0

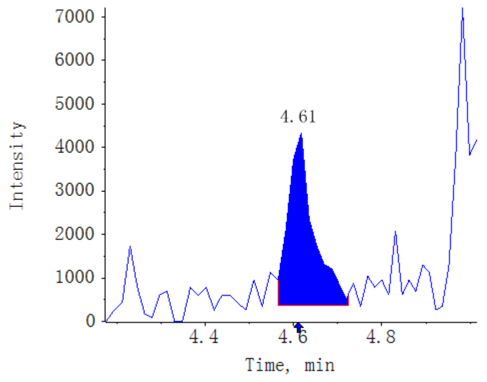

T24186684b_b
2MeScZR AREA:1.70e4 S/N:5.3

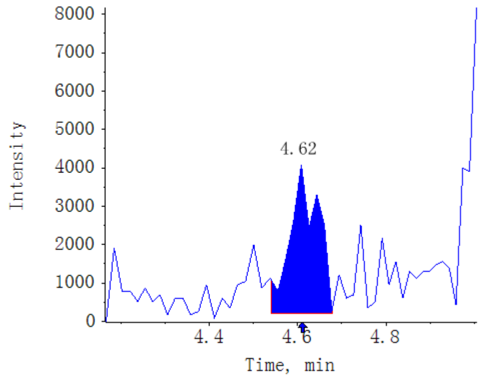

T24186684b_c
2MeScZR AREA:1.59e4 S/N:4.7

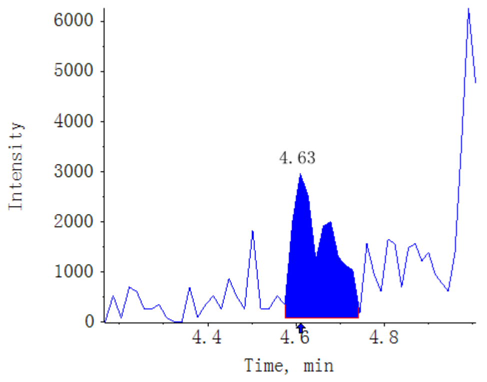

Result Table	MWXS-24-3064-a_9_WH6500-17_A20-3_V6.0_WSS_20240730	Algorithm Used	MQ4
Acquisition Method	ACC-PHs_V6.0_WH6500-17_CMY_20240521.dam	Instrument Name	Triple Quad 6500+ Low Mass
Project	N/A	Analytes QTY	109:50

Compound name: 2MeSiPR (382.1 / 250.2)

Sample Name	Sample Type	Area (cps)	Is Area (cps)	RT (min)	S/N	Target Conc	Calculated Conc.()
STD_0.01ppb	Standard	N/A	3.065e6	N/A	N/A	0.0100	N/A
STD_0.05ppb	Standard	7.83e3	3.030e6	5.79	15.2	0.0500	5.283102e-2
STD_0.1ppb	Standard	1.12e4	3.037e6	5.81	27.1	0.1000	8.607940e-2
STD_0.5ppb	Standard	5.25e4	2.659e6	5.80	44.3	0.5000	5.718470e-1
STD_1ppb	Standard	8.97e4	2.814e6	5.80	54.0	1.0000	9.381284e-1
STD_5ppb	Standard	5.29e5	2.801e6	5.82	45.8	5.0000	5.680949e0
STD_10ppb	Standard	9.32e5	2.618e6	5.80	50.7	10.0000	1.073337e1
STD_50ppb	Standard	4.20e6	2.412e6	5.80	72.4	50.0000	5.261050e1
STD_100ppb	Standard	6.80e6	2.237e6	5.81	48.0	100.0000	9.187307e1
STD_200ppb	Standard	1.24e7	2.096e6	5.81	70.3	200.0000	1.794024e2
STD_500ppb	Standard	2.73e7	1.785e6	5.80	41.8	500.0000	4.616419e2
V2.0_MW_RQC1_20240724	Quality Control	N/A	1.511e6	N/A	N/A	0.0000	N/A
Blank	Unknown	N/A	9.253e2	N/A	N/A	N/A	N/A
V3.0_MWMS_20240725_1	Unknown	5.73e5	4.177e6	5.78	69.4	N/A	4.123747e0
MWXS243064a_R1	Quality Control	N/A	1.316e6	N/A	N/A	0.0000	N/A
MWXS243064a_R2	Quality Control	N/A	1.226e6	N/A	N/A	0.0000	N/A
MWXS243064a_R3	Quality Control	N/A	1.274e6	N/A	N/A	0.0000	N/A
T24186682b_a	Unknown	N/A	1.337e6	N/A	N/A	N/A	N/A
T24186682b_b	Unknown	N/A	1.303e6	N/A	N/A	N/A	N/A
T24186682b_c	Unknown	N/A	1.287e6	N/A	N/A	N/A	N/A
T24186683b_a	Unknown	N/A	1.219e6	N/A	N/A	N/A	N/A
T24186683b_b	Unknown	N/A	1.264e6	N/A	N/A	N/A	N/A
T24186683b_c	Unknown	N/A	1.317e6	N/A	N/A	N/A	N/A
T24186684b_a	Unknown	N/A	1.242e6	N/A	N/A	N/A	N/A
T24186684b_b	Unknown	N/A	1.186e6	N/A	N/A	N/A	N/A
T24186684b_c	Unknown	N/A	1.175e6	N/A	N/A	N/A	N/A

Compound name: 2MeSiPR
Regression Equation: y = 0.03309 x + 8.34501e-4 (r = 0.99385) (weighting: 1 / x^2)

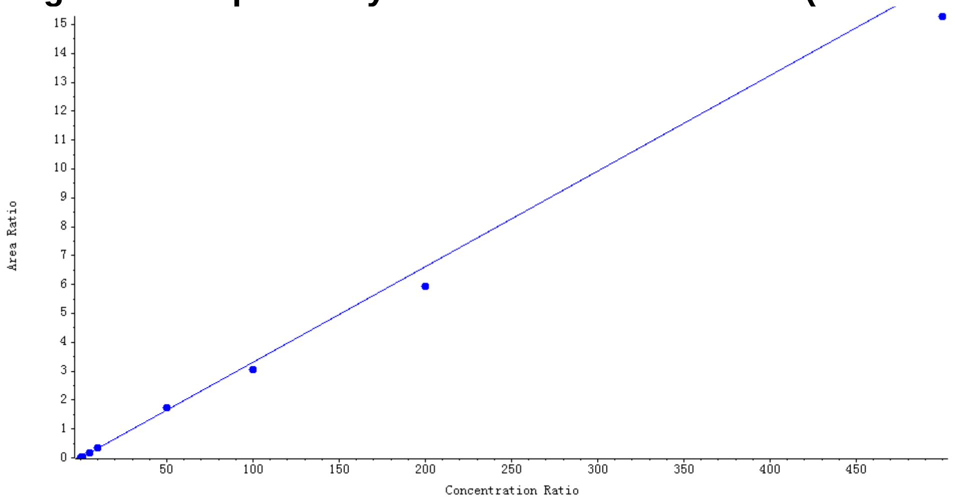

Peak Review

Blank
2MeSiPR AREA:N/A S/N:N/A

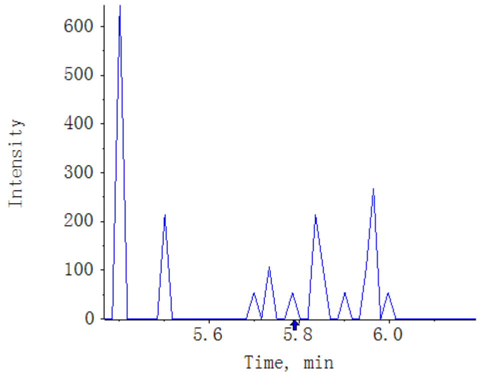

V3.0_MWMS_20240725_1
2MeSiPR AREA:5.73e5 S/N:69.4

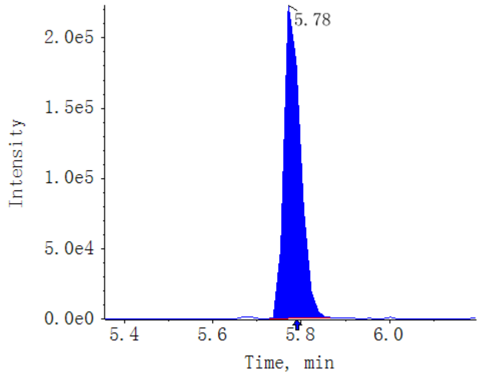

T24186682b_a
2MeSiPR AREA:N/A S/N:N/A

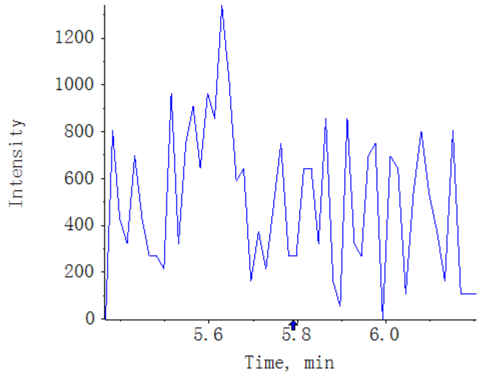

T24186682b_b
2MeSiPR AREA:N/A S/N:N/A

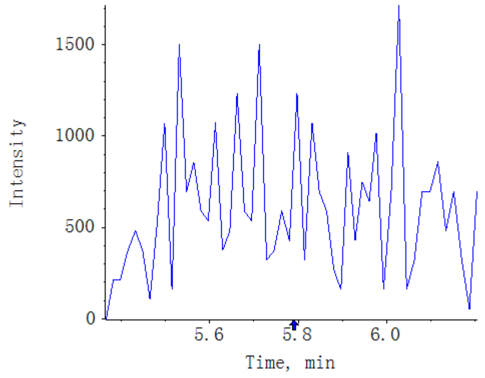

T24186682b_c
2MeSiPR AREA:N/A S/N:N/A

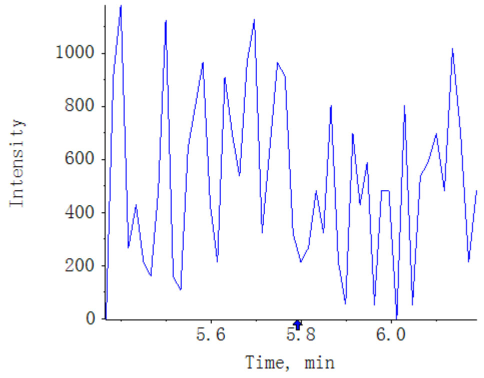

T24186683b_a
2MeSiPR AREA:N/A S/N:N/A

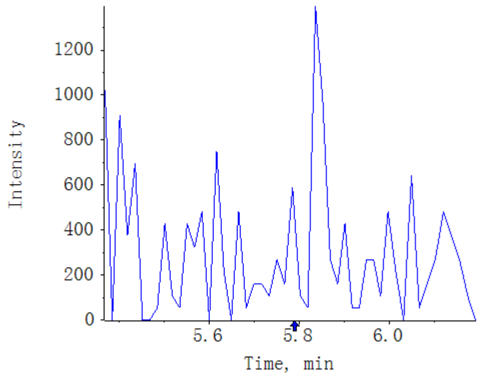

T24186683b_b
2MeSiPR AREA:N/A S/N:N/A

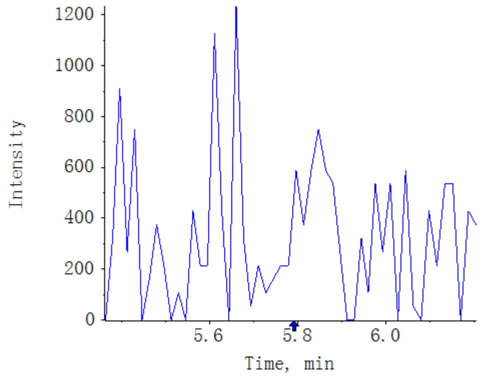

T24186683b_c
2MeSiPR AREA:N/A S/N:N/A

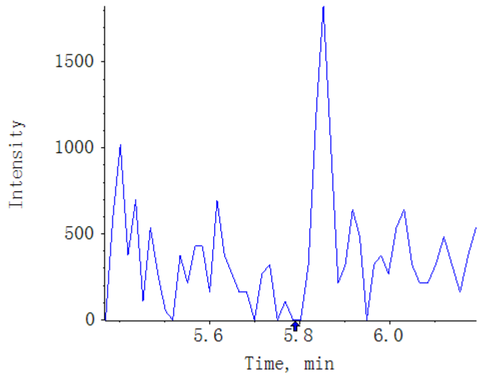

T24186684b_a
2MeSiPR AREA:N/A S/N:N/A

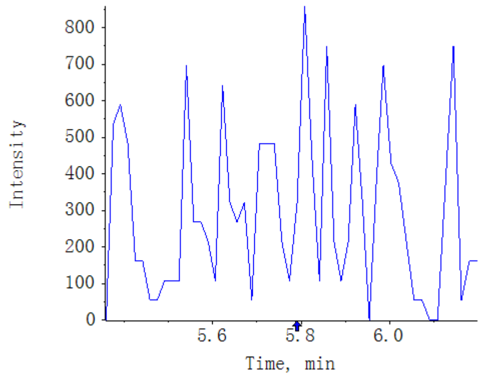

T24186684b_b
2MeSiPR AREA:N/A S/N:N/A

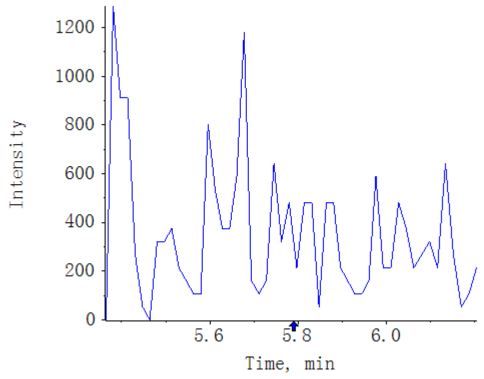

T24186684b_c
2MeSiPR AREA:N/A S/N:N/A

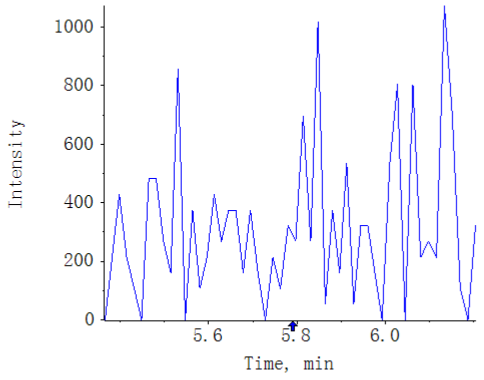

Result Table	MWXS-24-3064-a_9_WH6500-17_A20-3_V6.0_WSS_20240730	Algorithm Used	MQ4
Acquisition Method	ACC-PHs_V6.0_WH6500-17_CMY_20240521.dam	Instrument Name	Triple Quad 6500+ Low Mass
Project	N/A	Analytes QTY	109:51

Compound name: cZ (220.3 / 136.1)

Sample Name	Sample Type	Area (cps)	Is Area (cps)	RT (min)	S/N	Target Conc	Calculated Conc.()
STD_0.01ppb	Standard	N/A	6.534e6	N/A	N/A	0.0100	N/A
STD_0.05ppb	Standard	3.25e4	7.105e6	3.16	24.9	0.0500	5.067138e-2
STD_0.1ppb	Standard	5.67e4	6.945e6	3.17	33.5	0.1000	9.789621e-2
STD_0.5ppb	Standard	2.41e5	5.879e6	3.16	161.0	0.5000	5.288594e-1
STD_1ppb	Standard	3.75e5	5.871e6	3.15	203.2	1.0000	8.304321e-1
STD_5ppb	Standard	2.01e6	5.223e6	3.16	549.0	5.0000	5.040590e0
STD_10ppb	Standard	3.63e6	5.065e6	3.16	626.1	10.0000	9.424029e0
STD_50ppb	Standard	1.83e7	4.847e6	3.15	923.6	50.0000	4.970382e1
STD_100ppb	Standard	3.38e7	4.595e6	3.16	1020.3	100.0000	9.677947e1
STD_200ppb	Standard	6.69e7	4.227e6	3.15	1129.0	200.0000	2.080978e2
STD_500ppb	Standard	1.44e8	3.238e6	3.15	1063.6	500.0000	5.832893e2
V2.0_MW_RQC1_20240724	Quality Control	9.97e4	5.942e6	3.25	32.3	0.0000	2.112701e-1
Blank	Unknown	N/A	2.845e3	N/A	N/A	N/A	N/A
V3.0_MWMS_20240725_1	Unknown	4.57e6	1.146e7	3.25	1197.0	N/A	5.236750e0
MWXS243064a_R1	Quality Control	N/A	6.293e6	N/A	N/A	0.0000	N/A
MWXS243064a_R2	Quality Control	N/A	6.413e6	N/A	N/A	0.0000	N/A
MWXS243064a_R3	Quality Control	N/A	6.529e6	N/A	N/A	0.0000	N/A
T24186682b_a	Unknown	N/A	6.437e6	N/A	N/A	N/A	N/A
T24186682b_b	Unknown	N/A	6.323e6	N/A	N/A	N/A	N/A
T24186682b_c	Unknown	N/A	6.686e6	N/A	N/A	N/A	N/A
T24186683b_a	Unknown	N/A	6.369e6	N/A	N/A	N/A	N/A
T24186683b_b	Unknown	N/A	6.536e6	N/A	N/A	N/A	N/A
T24186683b_c	Unknown	N/A	6.791e6	N/A	N/A	N/A	N/A
T24186684b_a	Unknown	N/A	5.743e6	N/A	N/A	N/A	N/A
T24186684b_b	Unknown	N/A	6.364e6	N/A	N/A	N/A	N/A
T24186684b_c	Unknown	N/A	6.178e6	N/A	N/A	N/A	N/A

Compound name: cZ
Regression Equation: y = 0.07603 x + 7.17798e-4 (r = 0.99576) (weighting: 1 / x^2)

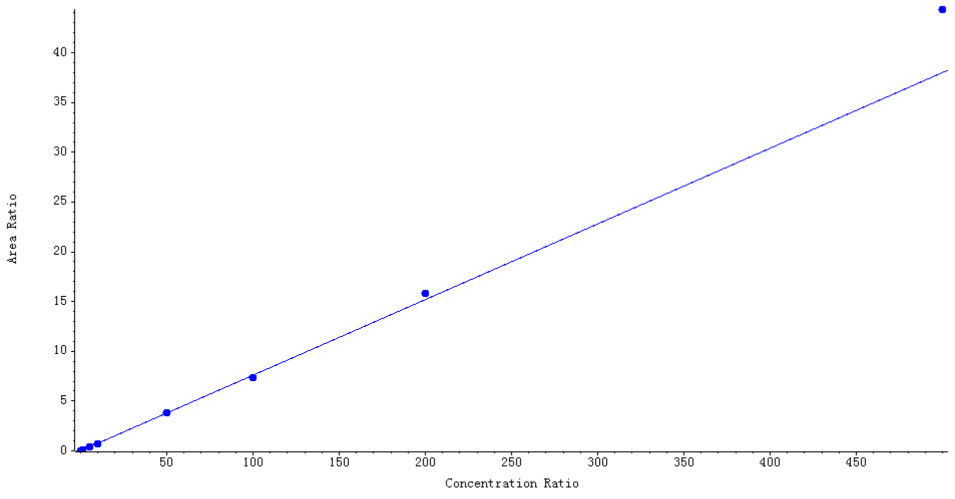

Peak Review

Blank
cZ AREA:N/A S/N:N/A

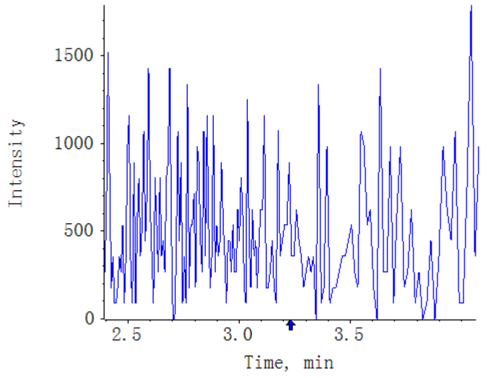

V3.0_MWMS_20240725_1
cZ AREA:4.57e6 S/N:1197.0

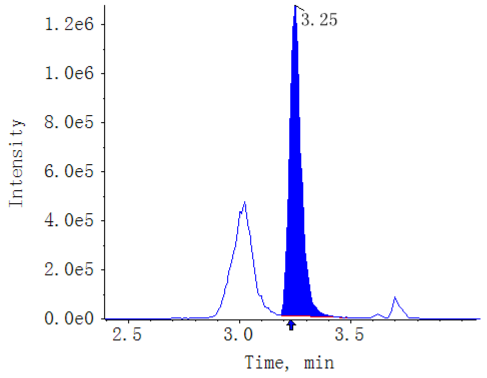

T24186682b_a
cZ AREA:N/A S/N:N/A

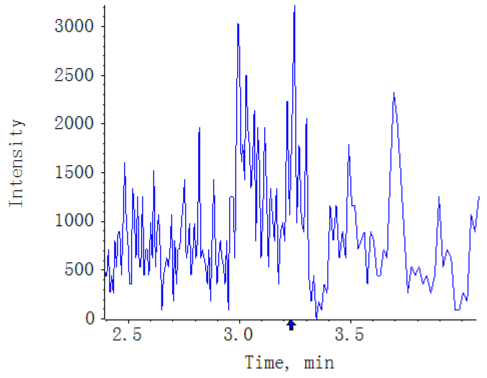

T24186682b_b
cZ AREA:N/A S/N:N/A

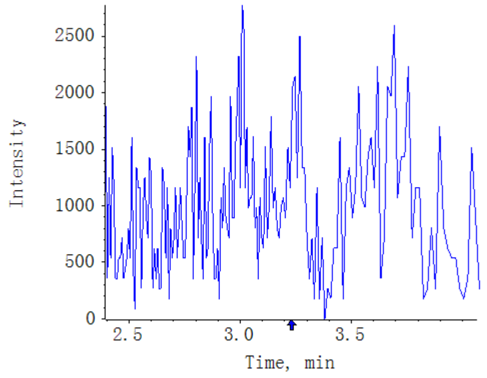

T24186682b_c
cZ AREA:N/A S/N:N/A

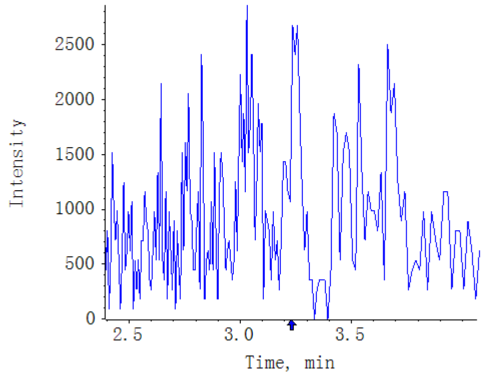

T24186683b_a
cZ AREA:N/A S/N:N/A

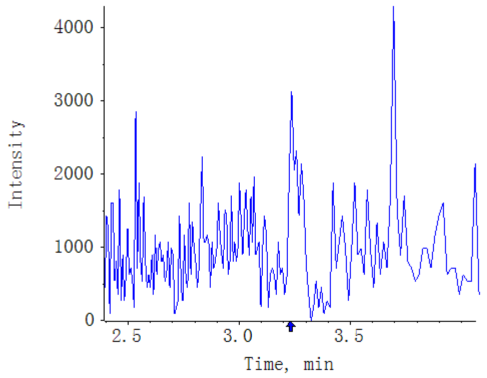

T24186683b_b
cZ AREA:N/A S/N:N/A

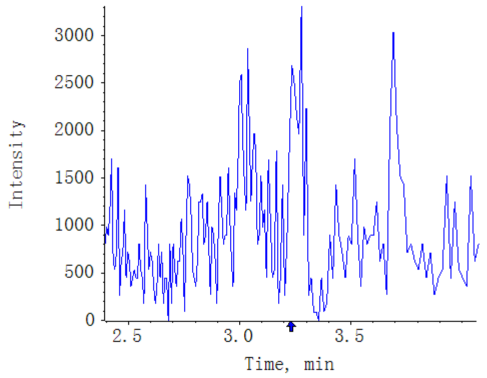

T24186683b_c
cZ AREA:N/A S/N:N/A

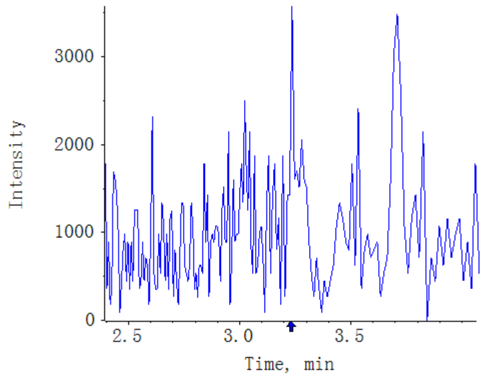

T24186684b_a
cZ AREA:N/A S/N:N/A

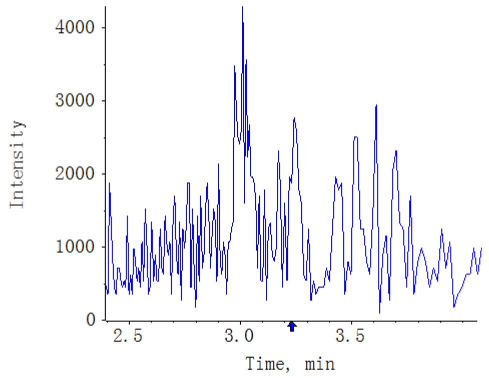

T24186684b_b
cZ AREA:N/A S/N:N/A

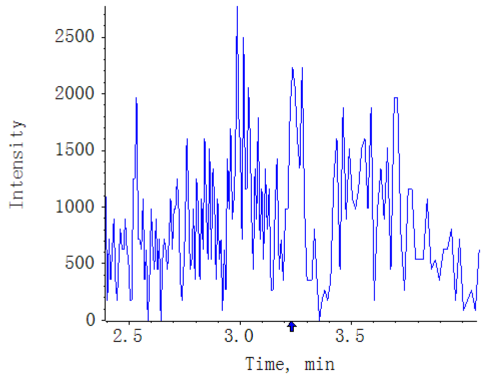

T24186684b_c
cZ AREA:N/A S/N:N/A

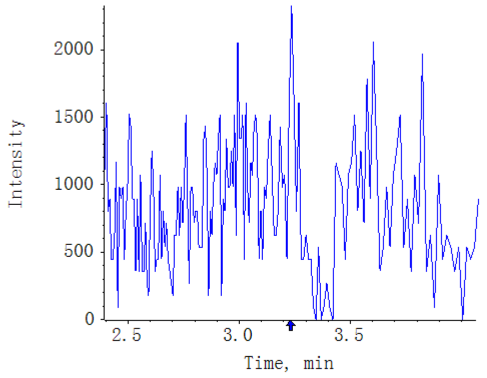

Result Table	MWXS-24-3064-a_9_WH6500-17_A20-3_V6.0_WSS_20240730	Algorithm Used	MQ4
Acquisition Method	ACC-PHs_V6.0_WH6500-17_CMY_20240521.dam	Instrument Name	Triple Quad 6500+ Low Mass
Project	N/A	Analytes QTY	109:52

Compound name: DHZ7G (384.2 / 222.1)

Sample Name	Sample Type	Area (cps)	Is Area (cps)	RT (min)	S/N	Target Conc	Calculated Conc.()
STD_0.01ppb	Standard	N/A	2.149e6	N/A	N/A	0.0100	N/A
STD_0.05ppb	Standard	5.20e4	2.298e6	3.10	49.0	0.0500	5.265106e-2
STD_0.1ppb	Standard	8.62e4	2.309e6	3.10	60.0	0.1000	8.953833e-2
STD_0.5ppb	Standard	4.08e5	1.950e6	3.10	272.9	0.5000	5.205551e-1
STD_1ppb	Standard	7.20e5	1.990e6	3.08	360.8	1.0000	9.026521e-1
STD_5ppb	Standard	3.47e6	1.697e6	3.10	1042.2	5.0000	5.120081e0
STD_10ppb	Standard	6.23e6	1.640e6	3.10	1432.5	10.0000	9.516752e0
STD_50ppb	Standard	3.51e7	1.686e6	3.09	2455.1	50.0000	5.219982e1
STD_100ppb	Standard	6.36e7	1.658e6	3.10	2415.8	100.0000	9.612501e1
STD_200ppb	Standard	1.36e8	1.623e6	3.08	2627.0	200.0000	2.101358e2
STD_500ppb	Standard	3.14e8	1.461e6	3.08	2852.1	500.0000	5.381082e2
V2.0_MW_RQC1_20240724	Quality Control	1.49e5	3.581e6	3.14	16.7	0.0000	1.003588e-1
Blank	Unknown	N/A	1.057e3	N/A	N/A	N/A	N/A
V3.0_MWMS_20240725_1	Unknown	6.08e6	2.638e6	3.19	1242.0	N/A	5.776798e0
MWXS243064a_R1	Quality Control	N/A	3.197e6	N/A	N/A	0.0000	N/A
MWXS243064a_R2	Quality Control	N/A	3.194e6	N/A	N/A	0.0000	N/A
MWXS243064a_R3	Quality Control	N/A	3.190e6	N/A	N/A	0.0000	N/A
T24186682b_a	Unknown	N/A	3.389e6	N/A	N/A	N/A	N/A
T24186682b_b	Unknown	N/A	3.111e6	N/A	N/A	N/A	N/A
T24186682b_c	Unknown	N/A	3.361e6	N/A	N/A	N/A	N/A
T24186683b_a	Unknown	N/A	3.077e6	N/A	N/A	N/A	N/A
T24186683b_b	Unknown	N/A	3.037e6	N/A	N/A	N/A	N/A
T24186683b_c	Unknown	N/A	3.008e6	N/A	N/A	N/A	N/A
T24186684b_a	Unknown	N/A	3.004e6	N/A	N/A	N/A	N/A
T24186684b_b	Unknown	N/A	3.134e6	N/A	N/A	N/A	N/A
T24186684b_c	Unknown	N/A	3.051e6	N/A	N/A	N/A	N/A

Compound name: DHZ7G
Regression Equation: y = 0.39895 x + 0.00163 (r = 0.99747) (weighting: 1 / x^2)

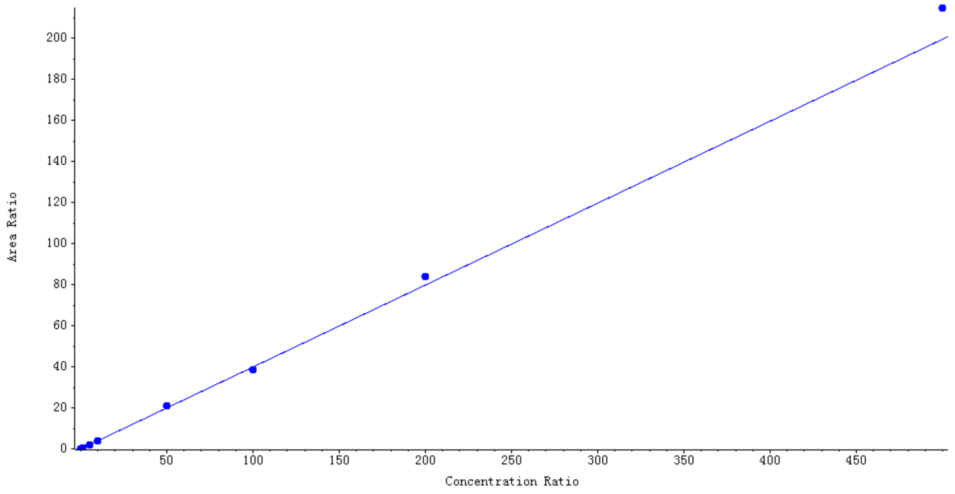

Peak Review

Blank
DHZ7G AREA:N/A S/N:N/A

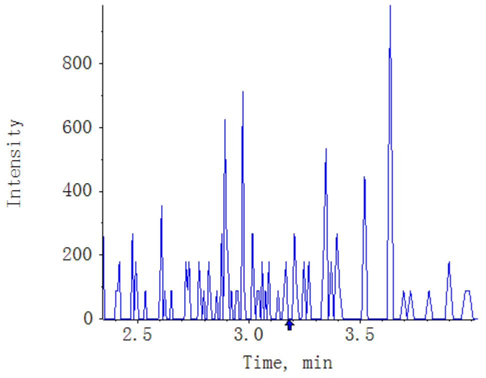

V3.0_MWMS_20240725_1
DHZ7G AREA:6.08e6 S/N:1242.0

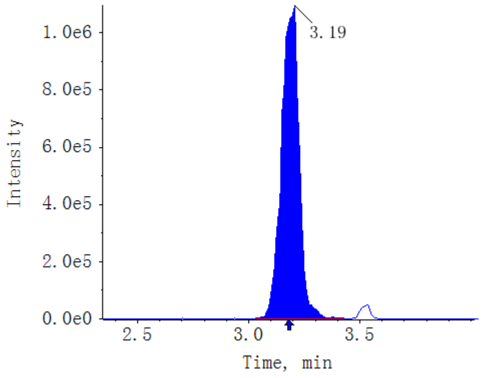

T24186682b_a
DHZ7G AREA:N/A S/N:N/A

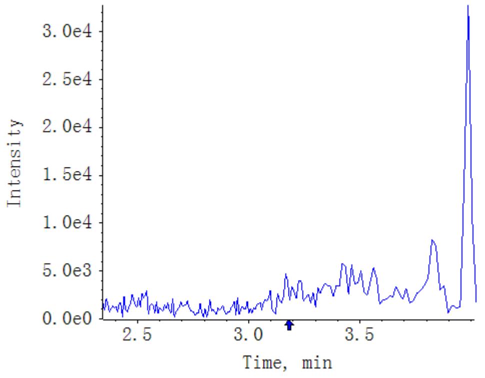

T24186682b_b
DHZ7G AREA:N/A S/N:N/A

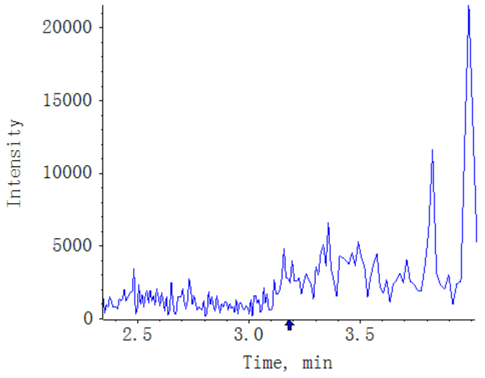

T24186682b_c
DHZ7G AREA:N/A S/N:N/A

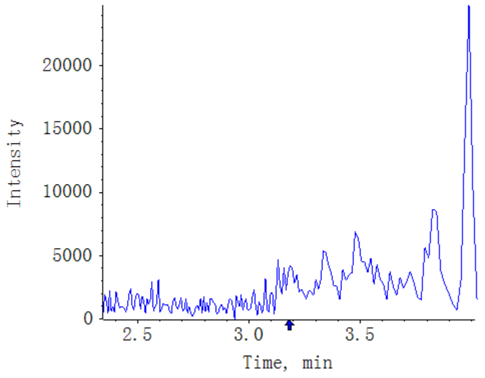

T24186683b_a
DHZ7G AREA:N/A S/N:N/A

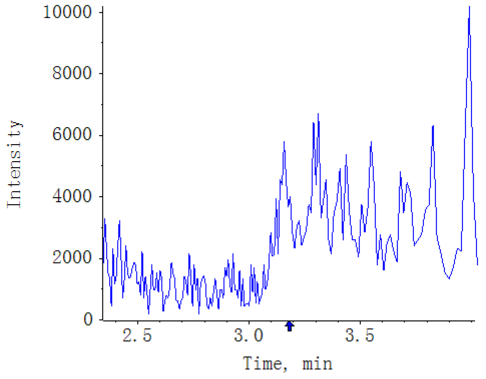

T24186683b_b
DHZ7G AREA:N/A S/N:N/A

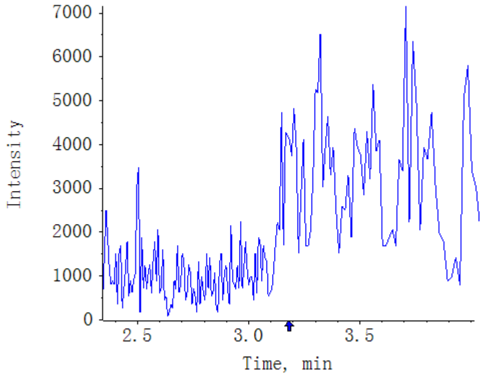

T24186683b_c
DHZ7G AREA:N/A S/N:N/A

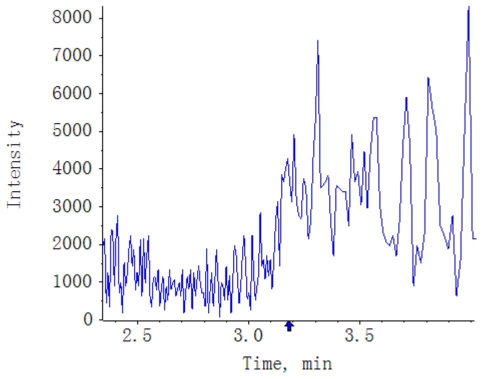

T24186684b_a
DHZ7G AREA:N/A S/N:N/A

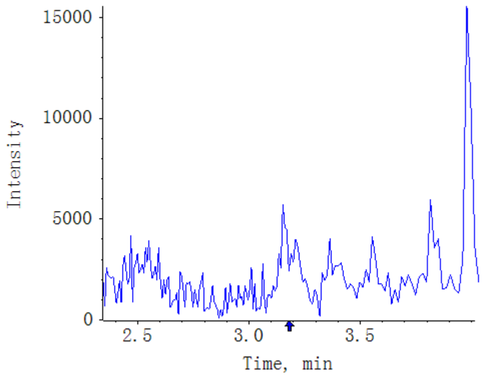

T24186684b_b
DHZ7G AREA:N/A S/N:N/A

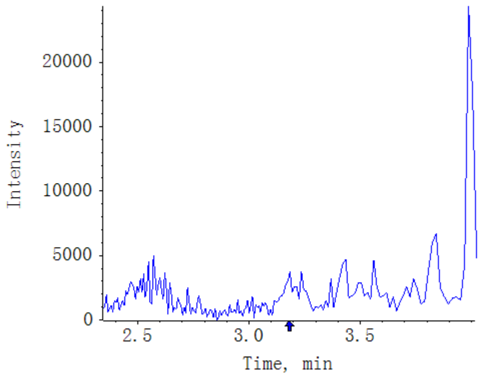

T24186684b_c
DHZ7G AREA:N/A S/N:N/A

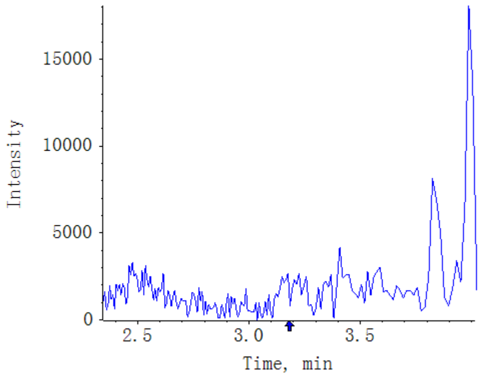

Result Table	MWXS-24-3064-a_9_WH6500-17_A20-3_V6.0_WSS_20240730	Algorithm Used	MQ4
Acquisition Method	ACC-PHs_V6.0_WH6500-17_CMY_20240521.dam	Instrument Name	Triple Quad 6500+ Low Mass
Project	N/A	Analytes QTY	109:53

Compound name: DZ (222.0 / 136.1)

Sample Name	Sample Type	Area (cps)	Is Area (cps)	RT (min)	S/N	Target Conc	Calculated Conc.()
STD_0.01ppb	Standard	N/A	7.184e6	N/A	N/A	0.0100	N/A
STD_0.05ppb	Standard	2.04e4	7.750e6	2.98	7.0	0.0500	6.388619e-2
STD_0.1ppb	Standard	3.07e4	7.338e6	3.01	13.5	0.1000	9.186025e-2
STD_0.5ppb	Standard	1.82e5	6.151e6	3.01	50.3	0.5000	5.490913e-1
STD_1ppb	Standard	2.87e5	6.214e6	2.99	78.9	1.0000	8.470968e-1
STD_5ppb	Standard	1.47e6	5.377e6	3.01	365.1	5.0000	4.939806e0
STD_10ppb	Standard	2.70e6	5.419e6	3.00	622.3	10.0000	8.972056e0
STD_50ppb	Standard	1.48e7	5.357e6	3.00	2030.8	50.0000	4.966470e1
STD_100ppb	Standard	2.72e7	5.173e6	3.00	2380.6	100.0000	9.448484e1
STD_200ppb	Standard	5.76e7	5.003e6	2.99	3085.6	200.0000	2.070040e2
STD_500ppb	Standard	1.23e8	4.410e6	2.98	3895.4	500.0000	5.000327e2
V2.0_MW_RQC1_20240724	Quality Control	N/A	6.808e6	N/A	N/A	0.0000	N/A
Blank	Unknown	N/A	1.451e3	N/A	N/A	N/A	N/A
V3.0_MWMS_20240725_1	Unknown	3.27e6	8.977e6	3.16	720.8	N/A	6.563762e0
MWXS243064a_R1	Quality Control	N/A	6.463e6	N/A	N/A	0.0000	N/A
MWXS243064a_R2	Quality Control	N/A	6.595e6	N/A	N/A	0.0000	N/A
MWXS243064a_R3	Quality Control	N/A	6.601e6	N/A	N/A	0.0000	N/A
T24186682b_a	Unknown	N/A	6.397e6	N/A	N/A	N/A	N/A
T24186682b_b	Unknown	N/A	6.305e6	N/A	N/A	N/A	N/A
T24186682b_c	Unknown	N/A	6.684e6	N/A	N/A	N/A	N/A
T24186683b_a	Unknown	N/A	6.187e6	N/A	N/A	N/A	N/A
T24186683b_b	Unknown	N/A	6.650e6	N/A	N/A	N/A	N/A
T24186683b_c	Unknown	N/A	6.592e6	N/A	N/A	N/A	N/A
T24186684b_a	Unknown	N/A	6.089e6	N/A	N/A	N/A	N/A
T24186684b_b	Unknown	N/A	6.654e6	N/A	N/A	N/A	N/A
T24186684b_c	Unknown	N/A	6.278e6	N/A	N/A	N/A	N/A

Compound name: DZ
Regression Equation: y = 0.05566 x + -9.26014e-4 (r = 0.99960) (weighting: 1 / x)

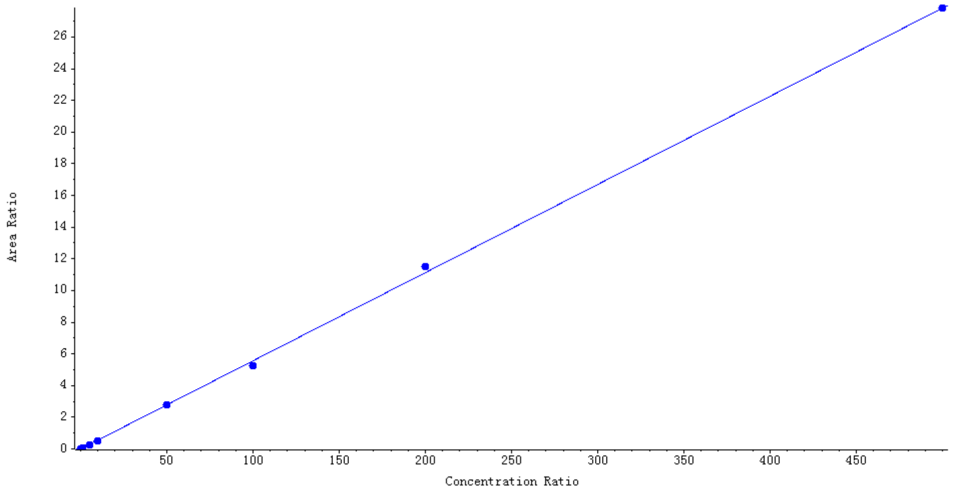

Peak Review

Blank
DZ AREA:N/A S/N:N/A

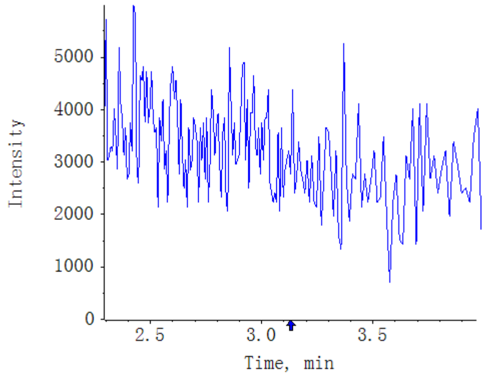

V3.0_MWMS_20240725_1
DZ AREA:3.27e6 S/N:720.8

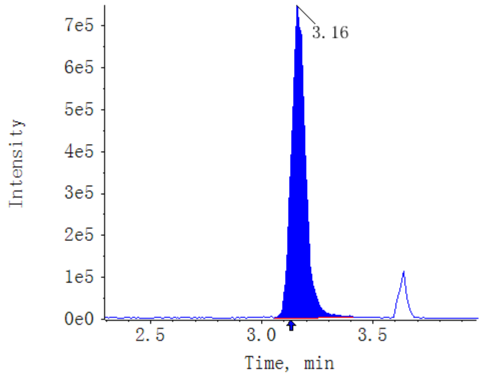

T24186682b_a
DZ AREA:N/A S/N:N/A

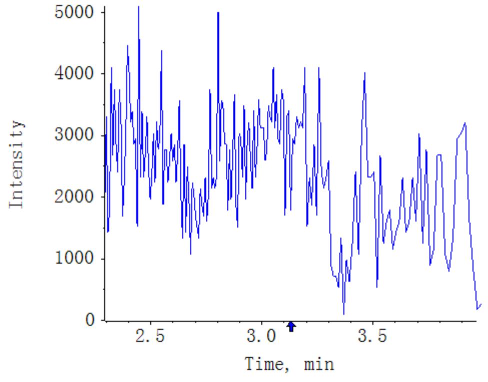

T24186682b_b
DZ AREA:N/A S/N:N/A

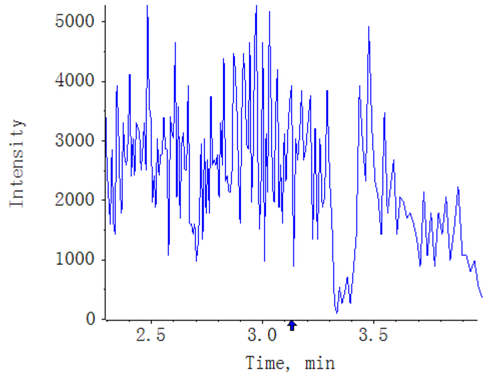

T24186682b_c
DZ AREA:N/A S/N:N/A

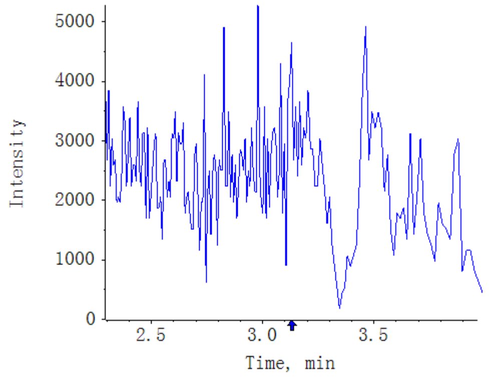

T24186683b_a
DZ AREA:N/A S/N:N/A

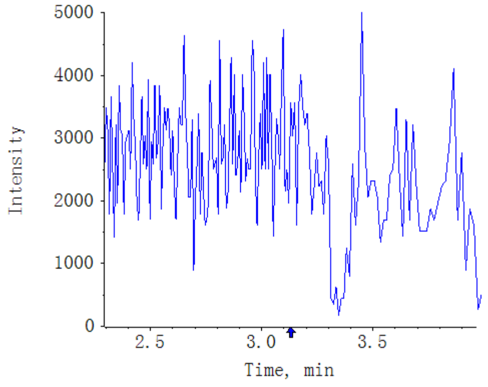

T24186683b_b
DZ AREA:N/A S/N:N/A

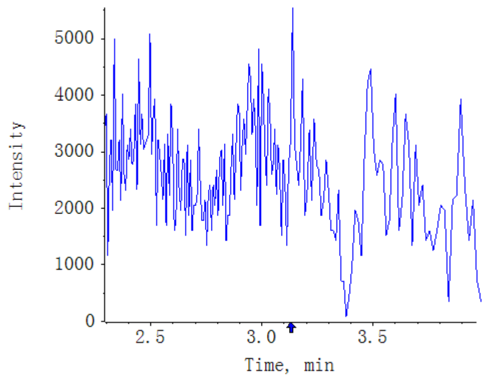

T24186683b_c
DZ AREA:N/A S/N:N/A

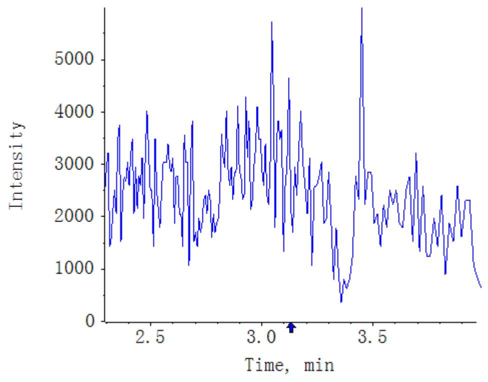

T24186684b_a
DZ AREA:N/A S/N:N/A

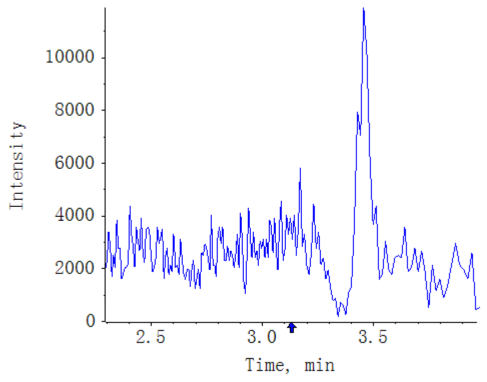

T24186684b_b
DZ AREA:N/A S/N:N/A

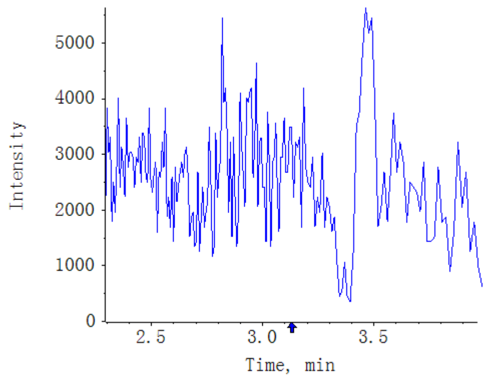

T24186684b_c
DZ AREA:N/A S/N:N/A

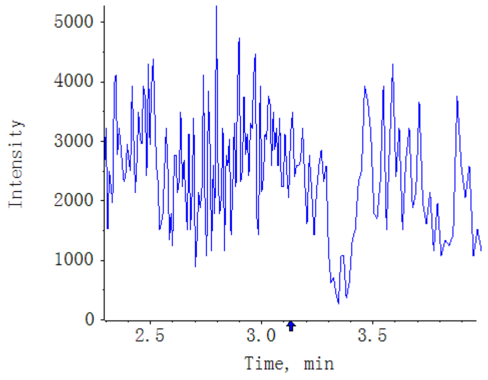

Result Table	MWXS-24-3064-a_9_WH6500-17_A20-3_V6.0_WSS_20240730	Algorithm Used	MQ4
Acquisition Method	ACC-PHs_V6.0_WH6500-17_CMY_20240521.dam	Instrument Name	Triple Quad 6500+ Low Mass
Project	N/A	Analytes QTY	109:54

Compound name: IP (204.3 / 136.0)

Sample Name	Sample Type	Area (cps)	Is Area (cps)	RT (min)	S/N	Target Conc	Calculated Conc.()
STD_0.01ppb	Standard	2.03e4	1.557e7	4.18	16.6	0.0100	9.945251e-3
STD_0.05ppb	Standard	5.36e4	1.530e7	4.18	28.7	0.0500	5.216786e-2
STD_0.1ppb	Standard	9.59e4	1.672e7	4.18	38.6	0.1000	9.510434e-2
STD_0.5ppb	Standard	4.22e5	1.486e7	4.17	49.6	0.5000	5.309768e-1
STD_1ppb	Standard	7.93e5	1.459e7	4.17	46.5	1.0000	1.029158e0
STD_5ppb	Standard	4.02e6	1.406e7	4.19	46.6	5.0000	5.476616e0
STD_10ppb	Standard	7.24e6	1.434e7	4.17	45.2	10.0000	9.676471e0
STD_50ppb	Standard	3.69e7	1.335e7	4.17	50.7	50.0000	5.300235e1
STD_100ppb	Standard	6.59e7	1.245e7	4.18	40.7	100.0000	1.016086e2
STD_200ppb	Standard	1.25e8	1.164e7	4.17	49.5	200.0000	2.058898e2
STD_500ppb	Standard	2.10e8	1.074e7	4.17	39.7	500.0000	3.757053e2
V2.0_MW_RQC1_20240724	Quality Control	1.65e4	6.875e6	4.17	11.2	0.0000	3.110311e-2
Blank	Unknown	N/A	1.497e4	N/A	N/A	N/A	N/A
V3.0_MWMS_20240725_1	Unknown	6.76e6	1.210e7	4.17	40.9	N/A	1.071095e1
MWXS243064a_R1	Quality Control	1.46e4	1.200e7	4.15	11.4	0.0000	8.230408e-3
MWXS243064a_R2	Quality Control	1.64e4	1.264e7	4.16	12.8	0.0000	9.775120e-3
MWXS243064a_R3	Quality Control	1.39e4	1.208e7	4.15	13.3	0.0000	6.986229e-3
T24186682b_a	Unknown	1.21e4	1.223e7	4.17	15.7	N/A	3.833511e-3
T24186682b_b	Unknown	1.60e4	1.220e7	4.16	11.2	N/A	1.010983e-2
T24186682b_c	Unknown	1.11e4	1.298e7	4.16	8.4	N/A	1.266655e-3
T24186683b_a	Unknown	1.34e4	1.222e7	4.16	8.2	N/A	5.901075e-3
T24186683b_b	Unknown	1.16e4	1.240e7	4.15	8.0	N/A	2.819194e-3
T24186683b_c	Unknown	1.13e4	1.286e7	4.16	7.4	N/A	1.797607e-3
T24186684b_a	Unknown	1.14e4	1.216e7	4.17	6.3	N/A	2.883833e-3
T24186684b_b	Unknown	1.14e4	1.270e7	4.18	10.4	N/A	2.078819e-3
T24186684b_c	Unknown	1.10e4	1.186e7	4.17	9.7	N/A	2.729195e-3

Compound name: IP
Regression Equation: y = 0.05205 x + 7.87791e-4 (r = 0.99543) (weighting: 1 / x^2)

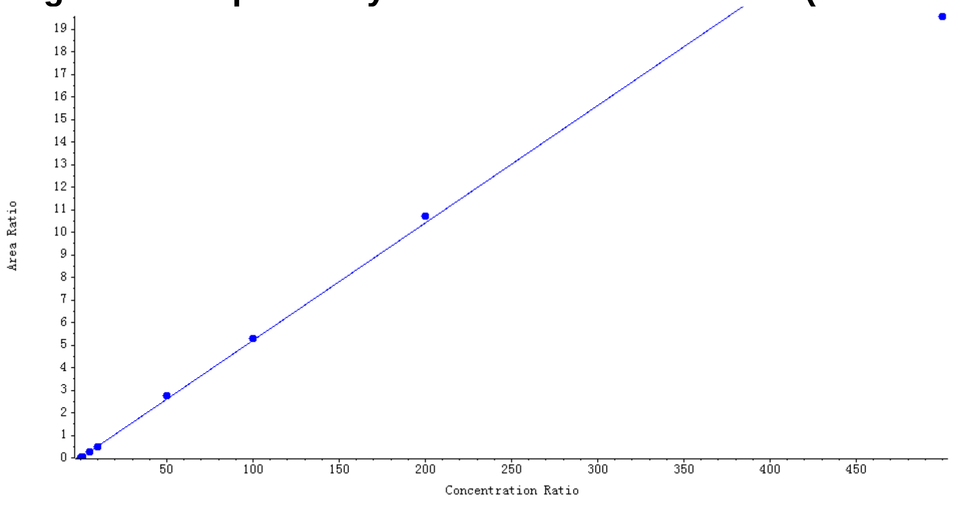

Peak Review

Blank
IP AREA:N/A S/N:N/A

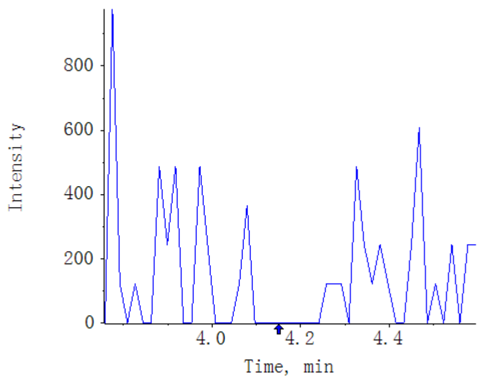

V3.0_MWMS_20240725_1
IP AREA:6.76e6 S/N:40.9

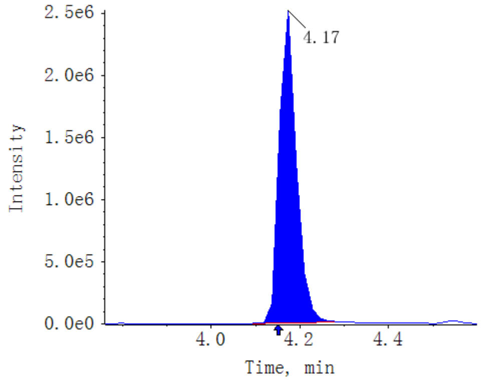

T24186682b_a
IP AREA:1.21e4 S/N:15.7

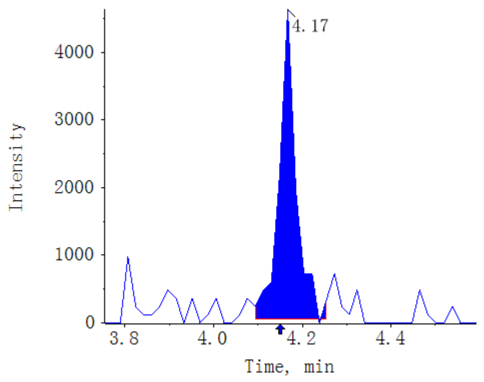

T24186682b_b
IP AREA:1.60e4 S/N:11.2

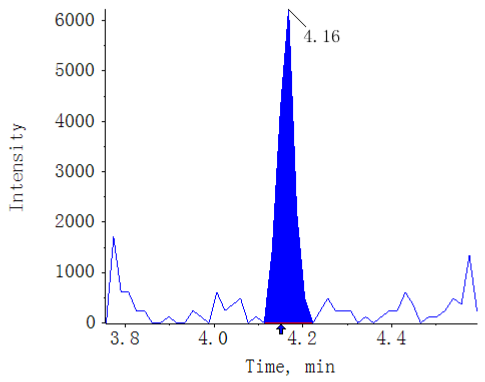

T24186682b_c
IP AREA:1.11e4 S/N:8.4

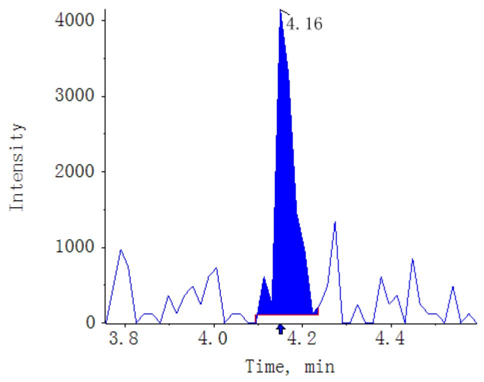

T24186683b_a
IP AREA:1.34e4 S/N:8.2

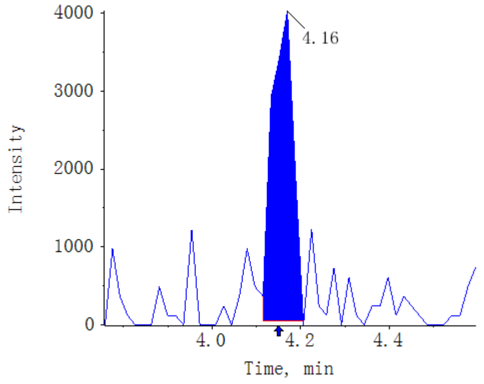

T24186683b_b
IP AREA:1.16e4 S/N:8.0

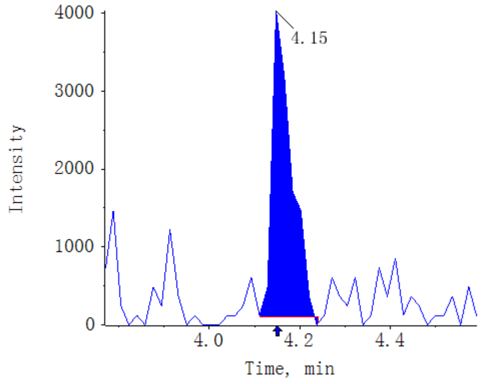

T24186683b_c
IP AREA:1.13e4 S/N:7.4

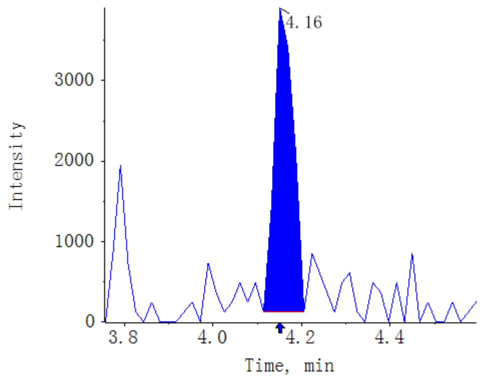

T24186684b_a
IP AREA:1.14e4 S/N:6.3

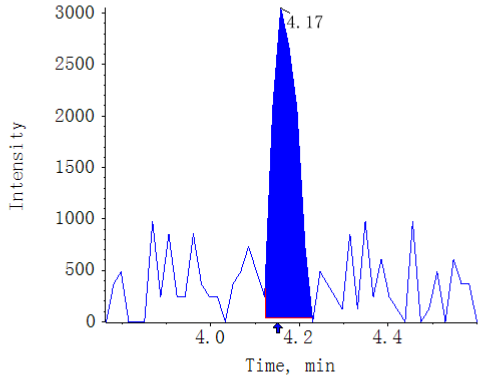

T24186684b_b
IP AREA:1.14e4 S/N:10.4

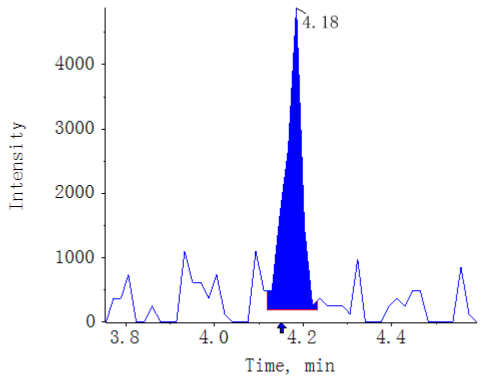

T24186684b_c
IP AREA:1.10e4 S/N:9.7

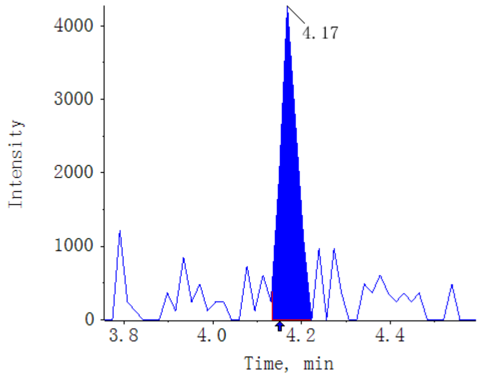

Result Table	MWXS-24-3064-a_9_WH6500-17_A20-3_V6.0_WSS_20240730	Algorithm Used	MQ4
Acquisition Method	ACC-PHs_V6.0_WH6500-17_CMY_20240521.dam	Instrument Name	Triple Quad 6500+ Low Mass
Project	N/A	Analytes QTY	109:55

Compound name: IPR (336.2 / 204.2)

Sample Name	Sample Type	Area (cps)	Is Area (cps)	RT (min)	S/N	Target Conc	Calculated Conc.()
STD_0.01ppb	Standard	2.10e4	1.320e7	4.55	23.0	0.0100	9.914739e-3
STD_0.05ppb	Standard	5.86e4	1.325e7	4.55	36.9	0.0500	5.046083e-2
STD_0.1ppb	Standard	1.06e5	1.314e7	4.56	54.7	0.1000	1.024805e-1
STD_0.5ppb	Standard	5.13e5	1.196e7	4.55	59.2	0.5000	6.009163e-1
STD_1ppb	Standard	8.37e5	1.194e7	4.55	54.7	1.0000	9.899807e-1
STD_5ppb	Standard	4.72e6	1.171e7	4.56	69.5	5.0000	5.756724e0
STD_10ppb	Standard	8.04e6	1.188e7	4.55	67.6	10.0000	9.665754e0
STD_50ppb	Standard	3.54e7	9.491e6	4.54	69.1	50.0000	5.336246e1
STD_100ppb	Standard	5.63e7	8.880e6	4.56	68.2	100.0000	9.061494e1
STD_200ppb	Standard	1.03e8	7.699e6	4.55	57.6	200.0000	1.906146e2
STD_500ppb	Standard	1.74e8	6.749e6	4.55	47.8	500.0000	3.691494e2
V2.0_MW_RQC1_20240724	Quality Control	1.19e4	5.338e6	4.53	7.2	0.0000	1.903280e-2
Blank	Unknown	N/A	1.226e3	N/A	N/A	N/A	N/A
V3.0_MWMS_20240725_1	Unknown	7.44e6	1.490e7	4.54	39.4	N/A	7.131406e0
MWXS243064a_R1	Quality Control	N/A	5.966e6	N/A	N/A	0.0000	N/A
MWXS243064a_R2	Quality Control	N/A	6.149e6	N/A	N/A	0.0000	N/A
MWXS243064a_R3	Quality Control	N/A	6.207e6	N/A	N/A	0.0000	N/A
T24186682b_a	Unknown	2.64e4	6.550e6	4.52	7.1	N/A	4.474863e-2
T24186682b_b	Unknown	2.69e4	6.203e6	4.53	4.8	N/A	4.920145e-2
T24186682b_c	Unknown	2.30e4	6.672e6	4.53	5.6	N/A	3.647542e-2
T24186683b_a	Unknown	3.10e4	7.190e6	4.51	6.1	N/A	4.881287e-2
T24186683b_b	Unknown	2.36e4	7.281e6	4.52	5.5	N/A	3.353888e-2
T24186683b_c	Unknown	1.38e4	7.030e6	4.53	2.4	N/A	1.527237e-2
T24186684b_a	Unknown	N/A	5.808e6	N/A	N/A	N/A	N/A
T24186684b_b	Unknown	N/A	5.958e6	N/A	N/A	N/A	N/A
T24186684b_c	Unknown	N/A	5.738e6	N/A	N/A	N/A	N/A

Compound name: IPR
Regression Equation: y = 0.06992 x + 8.99334e-4 (r = 0.99206) (weighting: 1 / x^2)

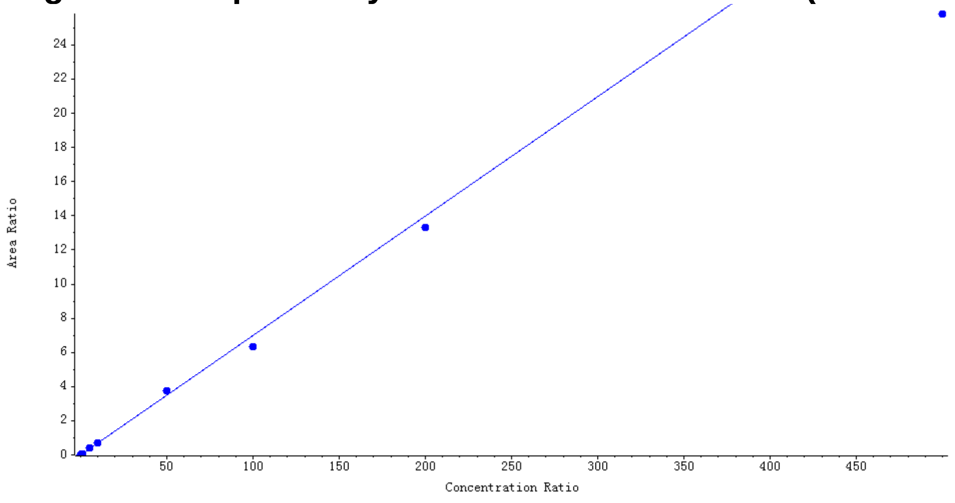

Peak Review

Blank
IPR AREA:N/A S/N:N/A

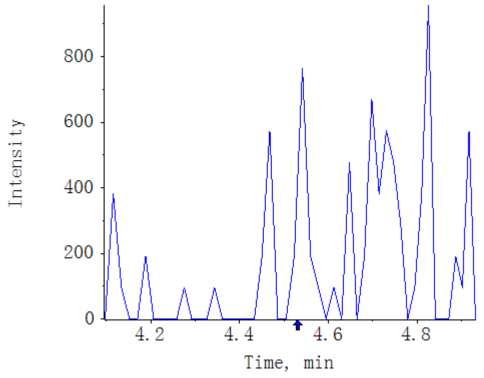

V3.0_MWMS_20240725_1
IPR AREA:7.44e6 S/N:39.4

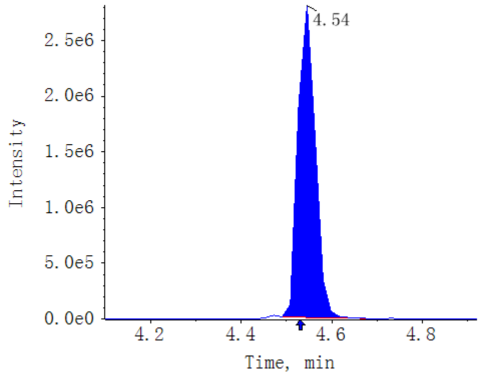

T24186682b_a
IPR AREA:2.64e4 S/N:7.1

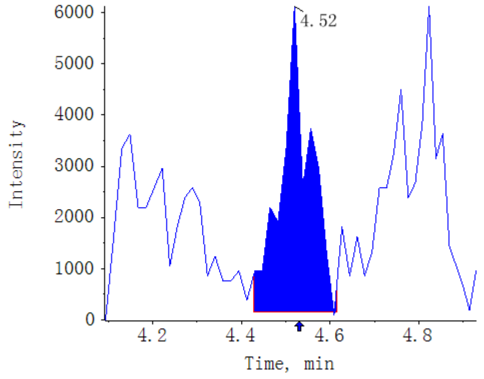

T24186682b_b
IPR AREA:2.69e4 S/N:4.8

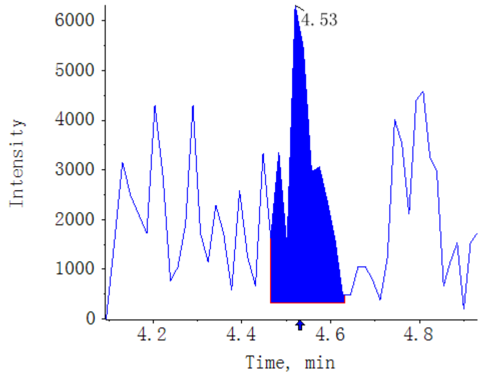

T24186682b_c
IPR AREA:2.30e4 S/N:5.6

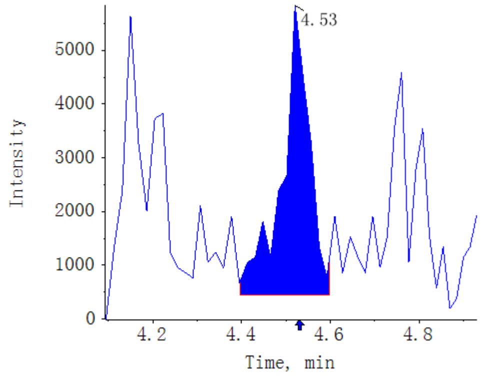

T24186683b_a
IPR AREA:3.10e4 S/N:6.1

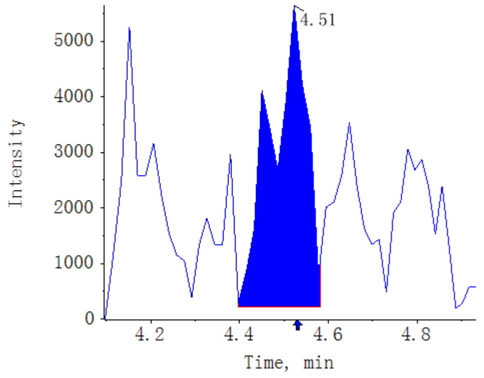

T24186683b_b
IPR AREA:2.36e4 S/N:5.5

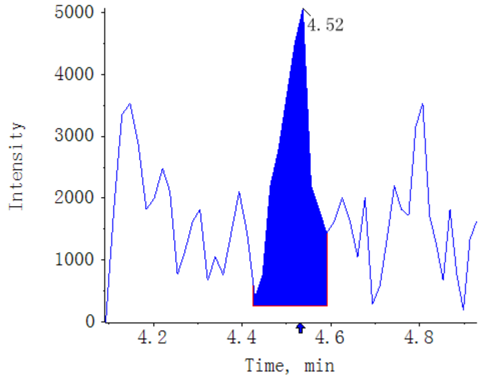

T24186683b_c
IPR AREA:1.38e4 S/N:2.4

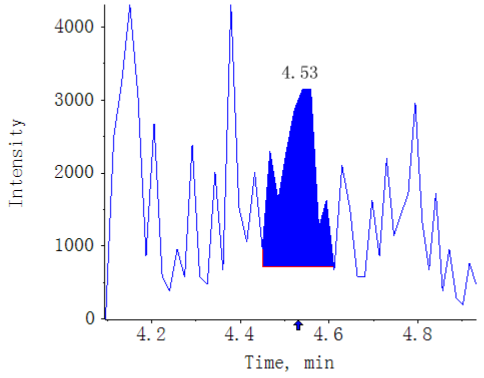

T24186684b_a
IPR AREA:N/A S/N:N/A

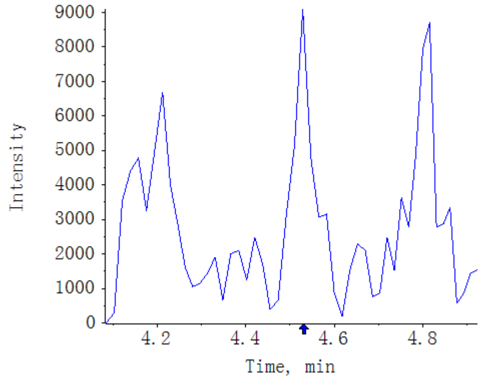

T24186684b_b
IPR AREA:N/A S/N:N/A

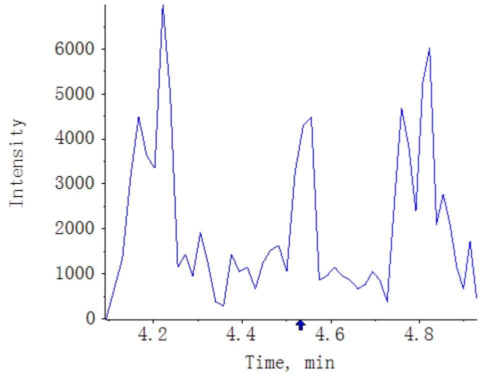

T24186684b_c
IPR AREA:N/A S/N:N/A

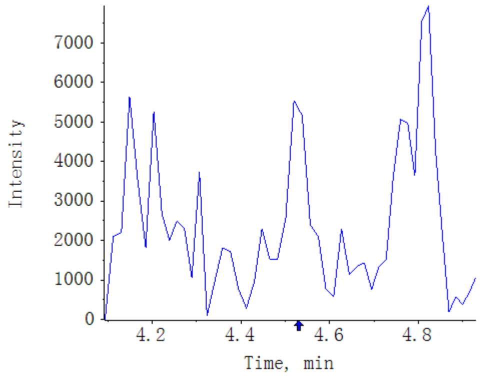

Result Table	MWXS-24-3064-a_9_WH6500-17_A20-3_V6.0_WSS_20240730	Algorithm Used	MQ4
Acquisition Method	ACC-PHs_V6.0_WH6500-17_CMY_20240521.dam	Instrument Name	Triple Quad 6500+ Low Mass
Project	N/A	Analytes QTY	109:56

Compound name: tZ (220.2 / 136.1)

Sample Name	Sample Type	Area (cps)	Is Area (cps)	RT (min)	S/N	Target Conc	Calculated Conc.()
STD_0.01ppb	Standard	N/A	2.440e6	N/A	N/A	0.0100	N/A
STD_0.05ppb	Standard	2.18e4	2.593e6	2.69	12.3	0.0500	5.151376e-2
STD_0.1ppb	Standard	3.88e4	2.495e6	2.72	16.2	0.1000	9.448302e-2
STD_0.5ppb	Standard	1.77e5	2.073e6	2.71	56.9	0.5000	5.127900e-1
STD_1ppb	Standard	3.12e5	2.115e6	2.69	99.0	1.0000	8.841082e-1
STD_5ppb	Standard	1.56e6	1.775e6	2.71	273.6	5.0000	5.268984e0
STD_10ppb	Standard	2.80e6	1.678e6	2.70	295.3	10.0000	9.984995e0
STD_50ppb	Standard	1.55e7	1.824e6	2.69	484.3	50.0000	5.093120e1
STD_100ppb	Standard	2.89e7	1.835e6	2.70	583.4	100.0000	9.428832e1
STD_200ppb	Standard	6.08e7	1.650e6	2.69	659.3	200.0000	2.207001e2
STD_500ppb	Standard	1.41e8	1.698e6	2.69	786.3	500.0000	4.989513e2
V2.0_MW_RQC1_20240724	Quality Control	3.67e4	3.188e6	3.09	9.6	0.0000	7.015519e-2
Blank	Unknown	N/A	3.390e3	N/A	N/A	N/A	N/A
V3.0_MWMS_20240725_1	Unknown	3.67e6	3.932e6	3.02	483.6	N/A	5.586893e0
MWXS243064a_R1	Quality Control	N/A	2.990e6	N/A	N/A	0.0000	N/A
MWXS243064a_R2	Quality Control	N/A	3.099e6	N/A	N/A	0.0000	N/A
MWXS243064a_R3	Quality Control	N/A	3.088e6	N/A	N/A	0.0000	N/A
T24186682b_a	Unknown	N/A	3.106e6	N/A	N/A	N/A	N/A
T24186682b_b	Unknown	N/A	3.036e6	N/A	N/A	N/A	N/A
T24186682b_c	Unknown	N/A	3.135e6	N/A	N/A	N/A	N/A
T24186683b_a	Unknown	N/A	2.973e6	N/A	N/A	N/A	N/A
T24186683b_b	Unknown	N/A	2.971e6	N/A	N/A	N/A	N/A
T24186683b_c	Unknown	N/A	3.009e6	N/A	N/A	N/A	N/A
T24186684b_a	Unknown	N/A	2.782e6	N/A	N/A	N/A	N/A
T24186684b_b	Unknown	N/A	3.028e6	N/A	N/A	N/A	N/A
T24186684b_c	Unknown	N/A	2.906e6	N/A	N/A	N/A	N/A

Compound name: tZ
Regression Equation: y = 0.16692 x + -2.07723e-4 (r = 0.99775) (weighting: 1 / x^2)

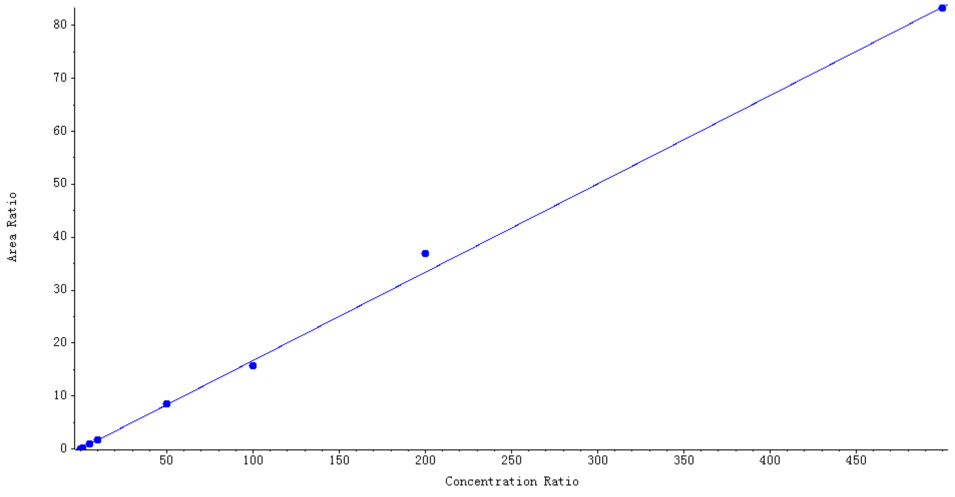

Peak Review

Blank
tZ AREA:N/A S/N:N/A

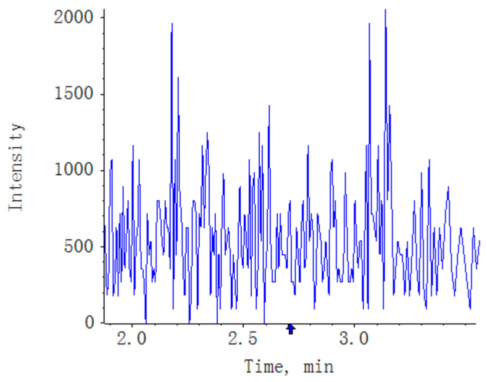

V3.0_MWMS_20240725_1
tZ AREA:3.67e6 S/N:483.6

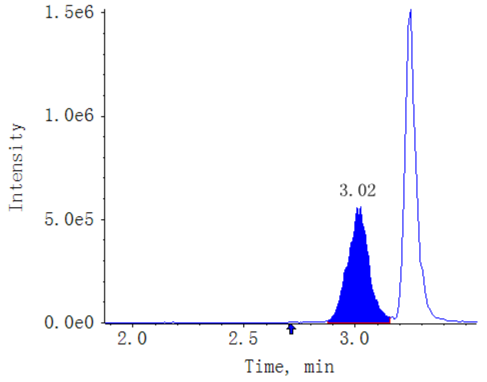

T24186682b_a
tZ AREA:N/A S/N:N/A

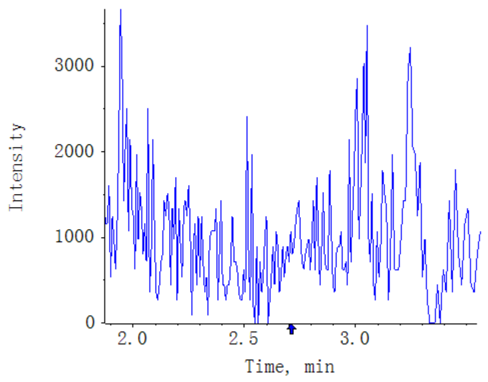

T24186682b_b
tZ AREA:N/A S/N:N/A

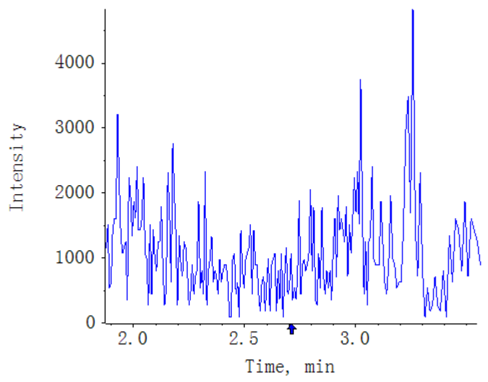

T24186682b_c
tZ AREA:N/A S/N:N/A

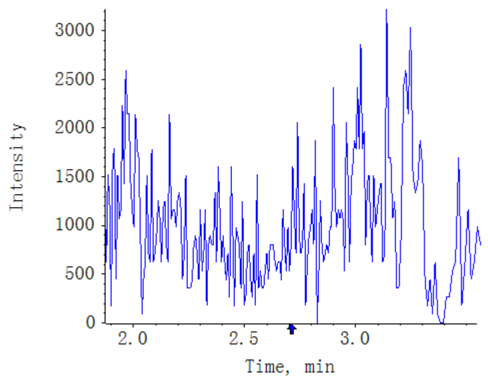

T24186683b_a
tZ AREA:N/A S/N:N/A

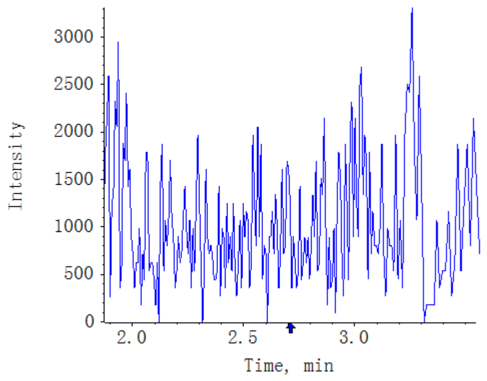

T24186683b_b
tZ AREA:N/A S/N:N/A

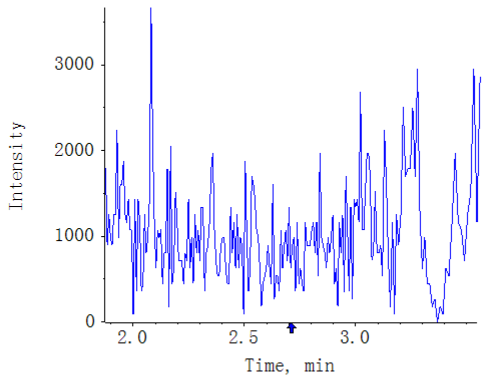

T24186683b_c
tZ AREA:N/A S/N:N/A

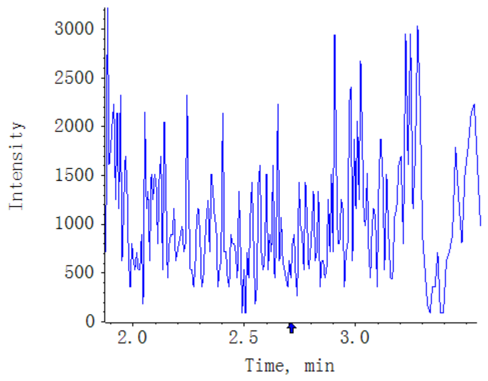

T24186684b_a
tZ AREA:N/A S/N:N/A

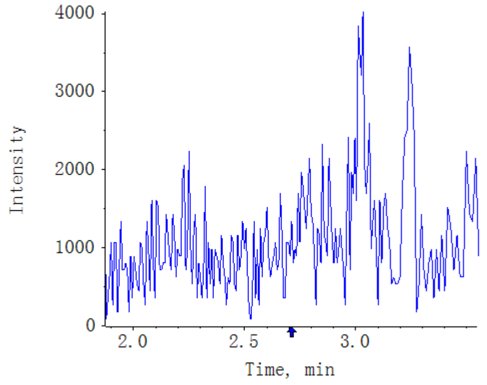

T24186684b_b
tZ AREA:N/A S/N:N/A

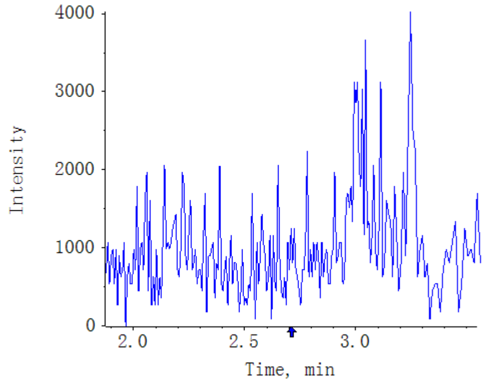

T24186684b_c
tZ AREA:N/A S/N:N/A

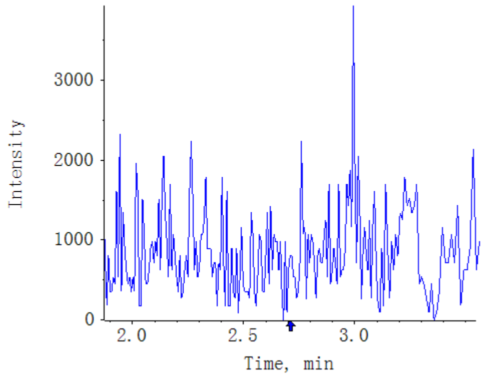

Result Table	MWXS-24-3064-a_9_WH6500-17_A20-3_V6.0_WSS_20240730	Algorithm Used	MQ4
Acquisition Method	ACC-PHs_V6.0_WH6500-17_CMY_20240521.dam	Instrument Name	Triple Quad 6500+ Low Mass
Project	N/A	Analytes QTY	109:57

Compound name: tZR (352.2 / 219.9)

Sample Name	Sample Type	Area (cps)	Is Area (cps)	RT (min)	S/N	Target Conc	Calculated Conc.()
STD_0.01ppb	Standard	1.02e4	2.440e6	3.62	6.4	0.0100	9.872663e-3
STD_0.05ppb	Standard	3.44e4	2.593e6	3.62	7.5	0.0500	5.491765e-2
STD_0.1ppb	Standard	5.08e4	2.495e6	3.63	8.5	0.1000	9.016066e-2
STD_0.5ppb	Standard	2.45e5	2.073e6	3.62	9.1	0.5000	5.755401e-1
STD_1ppb	Standard	4.08e5	2.115e6	3.62	10.2	1.0000	9.462382e-1
STD_5ppb	Standard	2.11e6	1.775e6	3.63	10.8	5.0000	5.885662e0
STD_10ppb	Standard	3.71e6	1.678e6	3.61	7.6	10.0000	1.095779e1
STD_50ppb	Standard	1.80e7	1.824e6	3.61	10.0	50.0000	4.907351e1
STD_100ppb	Standard	2.89e7	1.835e6	3.62	6.9	100.0000	7.820327e1
STD_200ppb	Standard	5.84e7	1.650e6	3.61	10.7	200.0000	1.758083e2
STD_500ppb	Standard	N/A	1.698e6	N/A	N/A	500.0000	N/A
V2.0_MW_RQC1_20240724	Quality Control	N/A	3.188e6	N/A	N/A	0.0000	N/A
Blank	Unknown	N/A	3.390e3	N/A	N/A	N/A	N/A
V3.0_MWMS_20240725_1	Unknown	4.82e6	3.932e6	3.62	8.5	N/A	6.071232e0
MWXS243064a_R1	Quality Control	N/A	2.990e6	N/A	N/A	0.0000	N/A
MWXS243064a_R2	Quality Control	N/A	3.099e6	N/A	N/A	0.0000	N/A
MWXS243064a_R3	Quality Control	N/A	3.088e6	N/A	N/A	0.0000	N/A
T24186682b_a	Unknown	N/A	3.106e6	N/A	N/A	N/A	N/A
T24186682b_b	Unknown	N/A	3.036e6	N/A	N/A	N/A	N/A
T24186682b_c	Unknown	N/A	3.135e6	N/A	N/A	N/A	N/A
T24186683b_a	Unknown	N/A	2.973e6	N/A	N/A	N/A	N/A
T24186683b_b	Unknown	N/A	2.971e6	N/A	N/A	N/A	N/A
T24186683b_c	Unknown	N/A	3.009e6	N/A	N/A	N/A	N/A
T24186684b_a	Unknown	3.51e4	2.782e6	3.61	7.7	N/A	5.175148e-2
T24186684b_b	Unknown	3.44e4	3.028e6	3.62	8.2	N/A	4.543523e-2
T24186684b_c	Unknown	2.69e4	2.906e6	3.62	5.4	N/A	3.493051e-2

Compound name: tZR
Regression Equation: y = 0.20140 x + 0.00220 (r = 0.99119) (weighting: 1 / x^2)

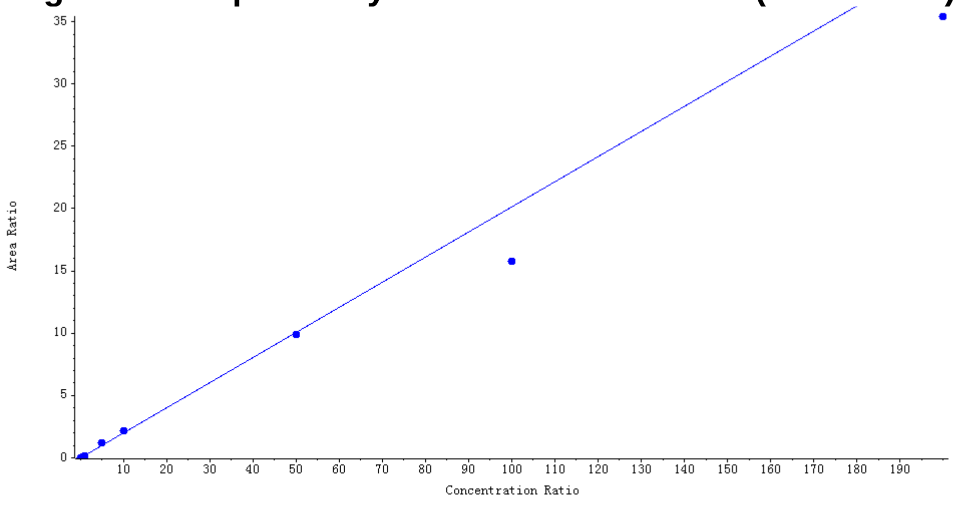

Peak Review

Blank
tZR AREA:N/A S/N:N/A

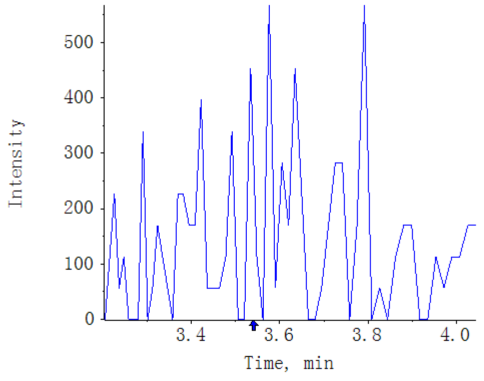

V3.0_MWMS_20240725_1
tZR AREA:4.82e6 S/N:8.5

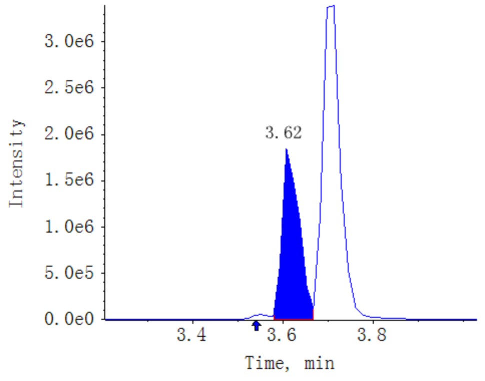

T24186682b_a
tZR AREA:N/A S/N:N/A

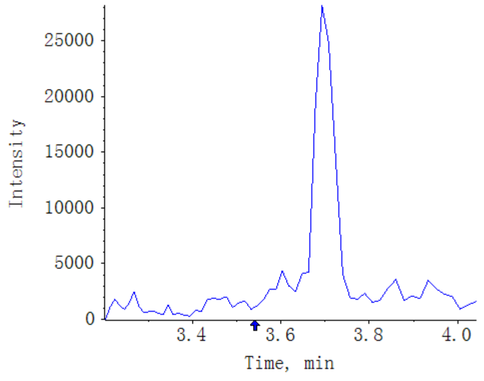

T24186682b_b
tZR AREA:N/A S/N:N/A

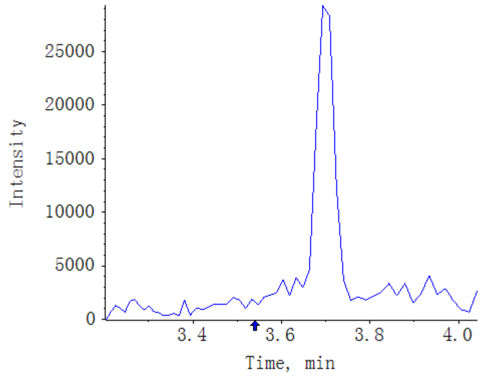

T24186682b_c
tZR AREA:N/A S/N:N/A

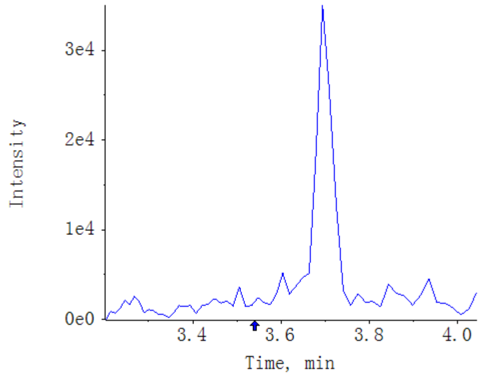

T24186683b_a
tZR AREA:N/A S/N:N/A

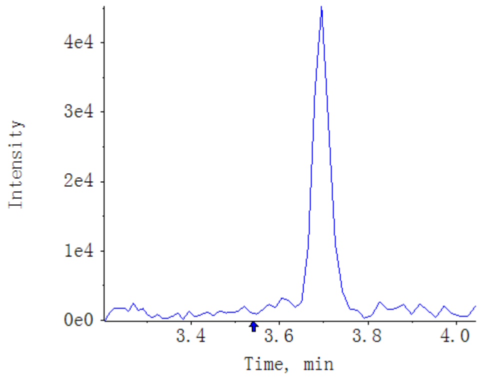

T24186683b_b
tZR AREA:N/A S/N:N/A

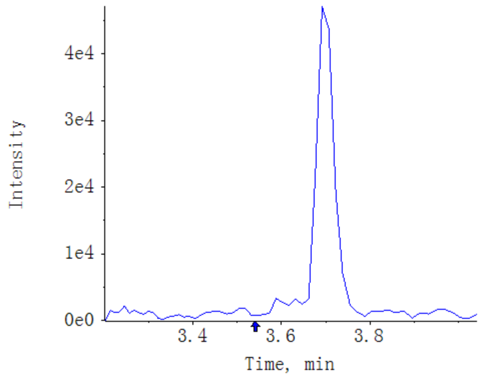

T24186683b_c
tZR AREA:N/A S/N:N/A

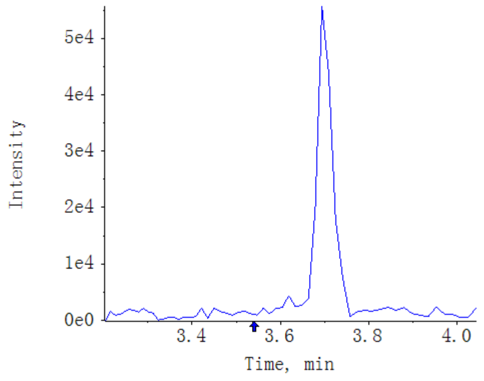

T24186684b_a
tZR AREA:3.51e4 S/N:7.7

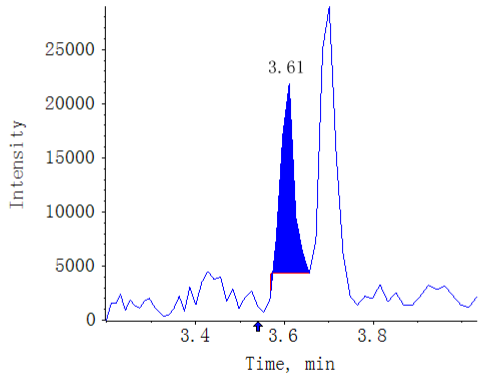

T24186684b_b
tZR AREA:3.44e4 S/N:8.2

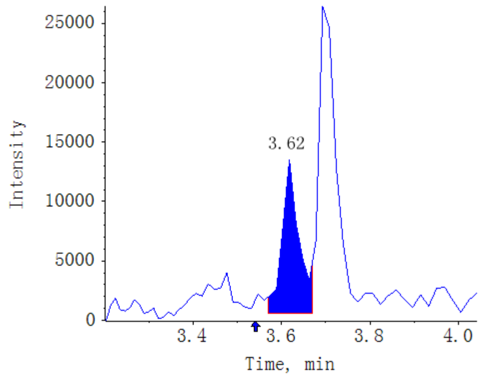

T24186684b_c
tZR AREA:2.69e4 S/N:5.4

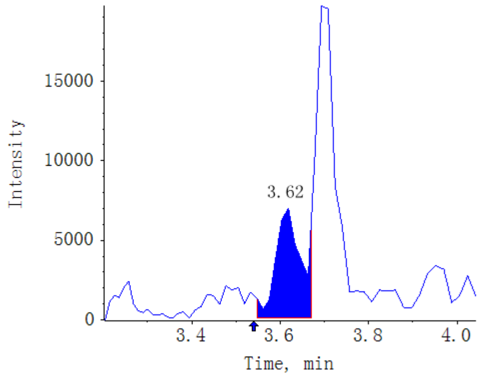

Result Table	MWXS-24-3064-a_9_WH6500-17_A20-3_V6.0_WSS_20240730	Algorithm Used	MQ4
Acquisition Method	ACC-PHs_V6.0_WH6500-17_CMY_20240521.dam	Instrument Name	Triple Quad 6500+ Low Mass
Project	N/A	Analytes QTY	109:58

Compound name: cZRMP (432.0 / 220.2)

Sample Name	Sample Type	Area (cps)	Is Area (cps)	RT (min)	S/N	Target Conc	Calculated Conc.()
STD_0.01ppb	Standard	N/A	3.065e6	N/A	N/A	0.0100	N/A
STD_0.05ppb	Standard	N/A	3.030e6	N/A	N/A	0.0500	N/A
STD_0.1ppb	Standard	7.74e3	3.037e6	2.35	18.6	0.1000	9.958478e-2
STD_0.5ppb	Standard	3.85e4	2.659e6	2.37	40.2	0.5000	5.339661e-1
STD_1ppb	Standard	7.11e4	2.814e6	2.35	54.3	1.0000	9.272589e-1
STD_5ppb	Standard	3.56e5	2.801e6	2.36	116.3	5.0000	4.638779e0
STD_10ppb	Standard	6.48e5	2.618e6	2.36	127.7	10.0000	9.028057e0
STD_50ppb	Standard	3.51e6	2.412e6	2.35	287.0	50.0000	5.300091e1
STD_100ppb	Standard	6.57e6	2.237e6	2.36	346.1	100.0000	1.070436e2
STD_200ppb	Standard	1.37e7	2.096e6	2.36	419.0	200.0000	2.377989e2
STD_500ppb	Standard	2.10e7	1.785e6	2.38	239.0	500.0000	4.294752e2
V2.0_MW_RQC1_20240724	Quality Control	N/A	1.511e6	N/A	N/A	0.0000	N/A
Blank	Unknown	N/A	9.253e2	N/A	N/A	N/A	N/A
V3.0_MWMS_20240725_1	Unknown	3.82e5	4.177e6	2.75	112.0	N/A	3.338583e0
MWXS243064a_R1	Quality Control	1.92e4	1.316e6	2.66	9.6	0.0000	5.375202e-1
MWXS243064a_R2	Quality Control	1.88e4	1.226e6	2.64	8.2	0.0000	5.668634e-1
MWXS243064a_R3	Quality Control	1.82e4	1.274e6	2.61	11.8	0.0000	5.271155e-1
T24186682b_a	Unknown	1.93e4	1.337e6	2.68	8.2	N/A	5.331364e-1
T24186682b_b	Unknown	2.42e4	1.303e6	2.70	6.4	N/A	6.823055e-1
T24186682b_c	Unknown	1.98e4	1.287e6	2.70	7.9	N/A	5.680857e-1
T24186683b_a	Unknown	2.14e4	1.219e6	2.69	10.0	N/A	6.472771e-1
T24186683b_b	Unknown	1.67e4	1.264e6	2.71	9.6	N/A	4.875561e-1
T24186683b_c	Unknown	2.11e4	1.317e6	2.70	13.1	N/A	5.905780e-1
T24186684b_a	Unknown	1.50e4	1.242e6	2.65	5.4	N/A	4.458251e-1
T24186684b_b	Unknown	1.54e4	1.186e6	2.65	7.7	N/A	4.795825e-1
T24186684b_c	Unknown	1.39e4	1.175e6	2.67	9.2	N/A	4.362298e-1

Compound name: cZRMP
Regression Equation: y = 0.02744 x + -1.83322e-4 (r = 0.99398) (weighting: 1 / x^2)

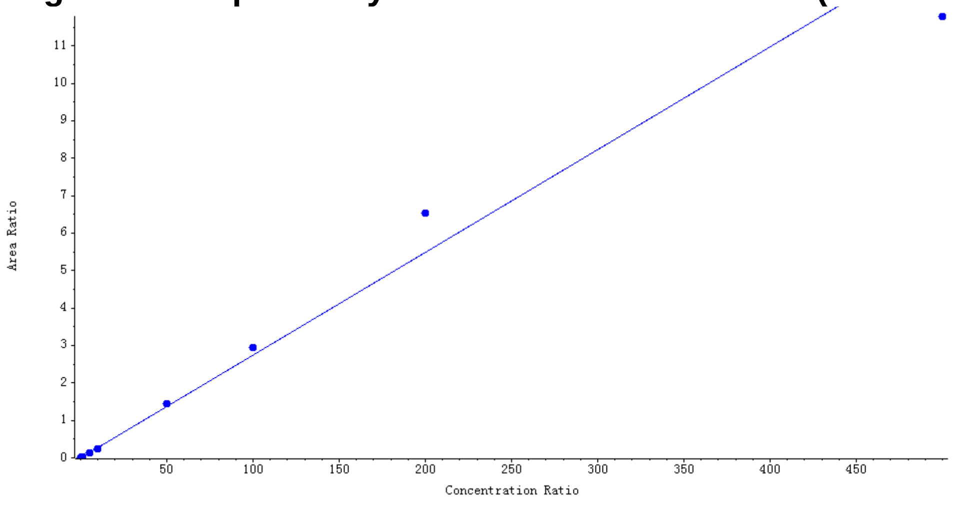

Peak Review

Blank
cZRMP AREA:N/A S/N:N/A

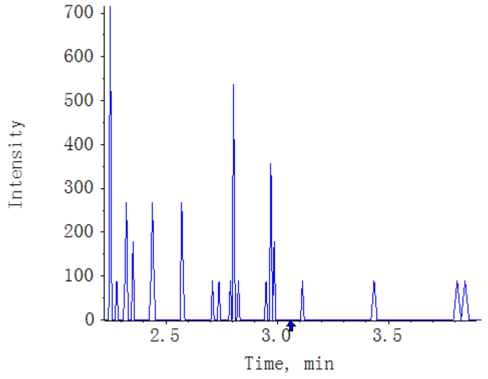

V3.0_MWMS_20240725_1
cZRMP AREA:3.82e5 S/N:112.0

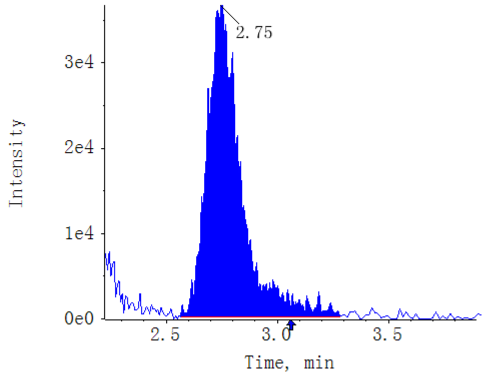

T24186682b_a
cZRMP AREA:1.93e4 S/N:8.2

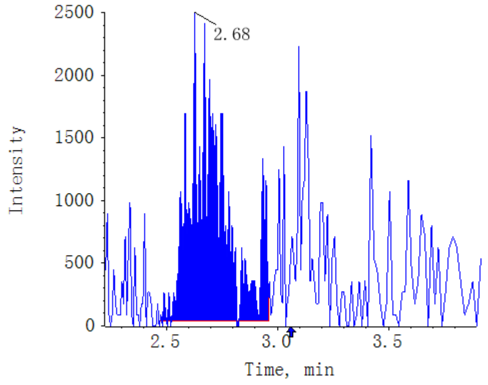

T24186682b_b
cZRMP AREA:2.42e4 S/N:6.4

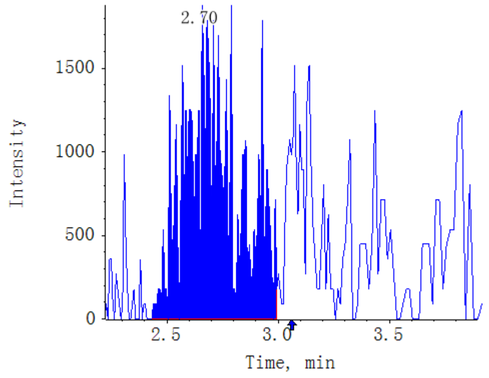

T24186682b_c
cZRMP AREA:1.98e4 S/N:7.9

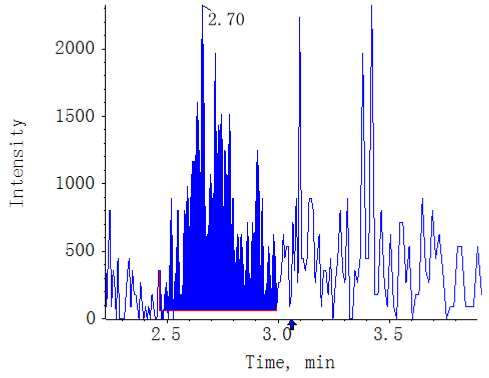

T24186683b_a
cZRMP AREA:2.14e4 S/N:10.0

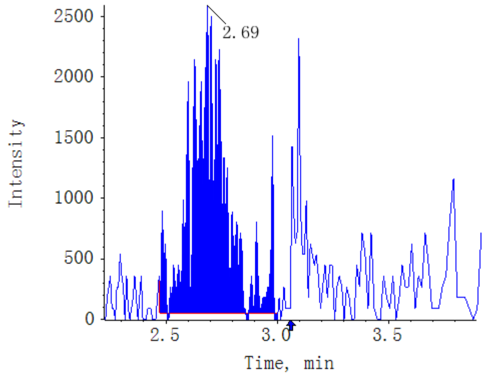

T24186683b_b
cZRMP AREA:1.67e4 S/N:9.6

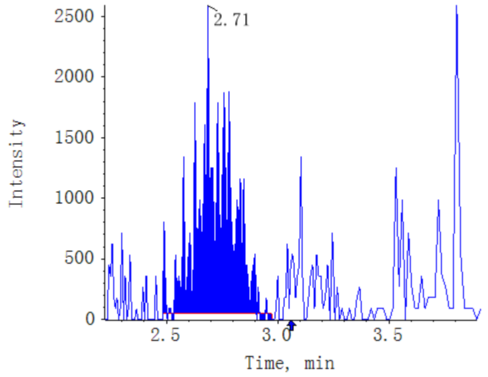

T24186683b_c
cZRMP AREA:2.11e4 S/N:13.1

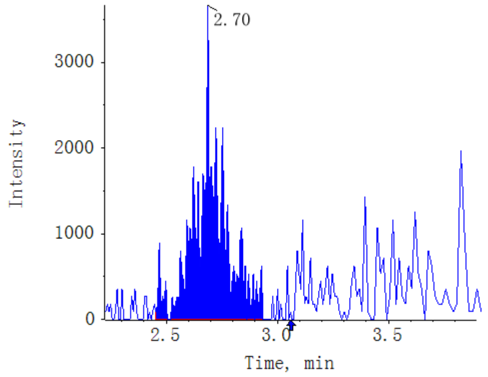

T24186684b_a
cZRMP AREA:1.50e4 S/N:5.4

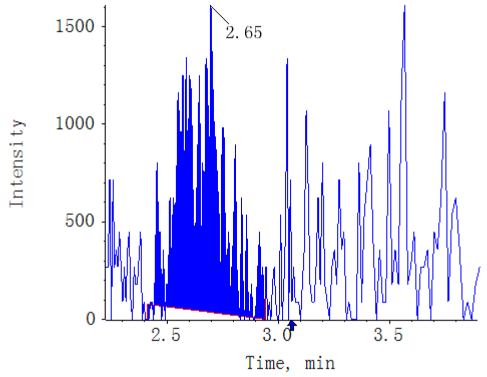

T24186684b_b
cZRMP AREA:1.54e4 S/N:7.7

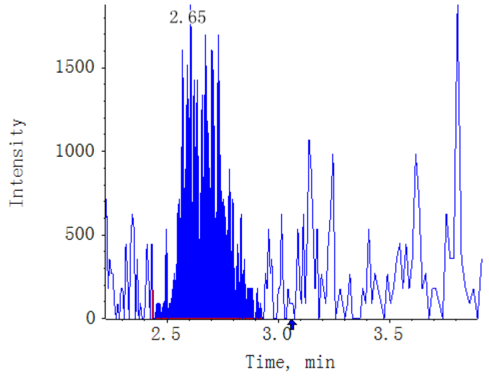

T24186684b_c
cZRMP AREA:1.39e4 S/N:9.2

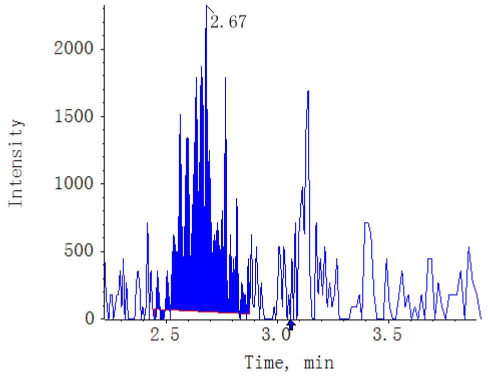

Result Table	MWXS-24-3064-a_9_WH6500-17_A20-3_V6.0_WSS_20240730	Algorithm Used	MQ4
Acquisition Method	ACC-PHs_V6.0_WH6500-17_CMY_20240521.dam	Instrument Name	Triple Quad 6500+ Low Mass
Project	N/A	Analytes QTY	109:59

Compound name: iPRMP (416.0 / 204.1)

Sample Name	Sample Type	Area (cps)	Is Area (cps)	RT (min)	S/N	Target Conc	Calculated Conc.()
STD_0.01ppb	Standard	6.38e3	3.065e6	3.79	12.0	0.0100	1.016038e-2
STD_0.05ppb	Standard	1.64e4	3.030e6	3.79	14.4	0.0500	4.808613e-2
STD_0.1ppb	Standard	2.86e4	3.037e6	3.80	21.8	0.1000	9.398196e-2
STD_0.5ppb	Standard	1.23e5	2.659e6	3.79	33.2	0.5000	5.159141e-1
STD_1ppb	Standard	1.83e5	2.814e6	3.79	37.1	1.0000	7.299370e-1
STD_5ppb	Standard	1.07e6	2.801e6	3.80	52.7	5.0000	4.363172e0
STD_10ppb	Standard	2.11e6	2.618e6	3.79	49.5	10.0000	9.175812e0
STD_50ppb	Standard	1.13e7	2.412e6	3.78	49.0	50.0000	5.353939e1
STD_100ppb	Standard	2.13e7	2.237e6	3.80	33.7	100.0000	1.086070e2
STD_200ppb	Standard	4.51e7	2.096e6	3.79	51.3	200.0000	2.456374e2
STD_500ppb	Standard	8.96e7	1.785e6	3.80	37.1	500.0000	5.726972e2
V2.0_MW_RQC1_20240724	Quality Control	3.37e4	1.511e6	3.79	15.6	0.0000	2.409481e-1
Blank	Unknown	N/A	9.253e2	N/A	N/A	N/A	N/A
V3.0_MWMS_20240725_1	Unknown	1.19e6	4.177e6	3.80	52.5	N/A	3.242843e0
MWXS243064a_R1	Quality Control	1.42e5	1.316e6	3.77	44.1	0.0000	1.220556e0
MWXS243064a_R2	Quality Control	1.63e5	1.226e6	3.79	43.5	0.0000	1.502374e0
MWXS243064a_R3	Quality Control	1.70e5	1.274e6	3.77	39.2	0.0000	1.506984e0
T24186682b_a	Unknown	1.73e5	1.337e6	3.78	47.2	N/A	1.462851e0
T24186682b_b	Unknown	1.59e5	1.303e6	3.78	42.0	N/A	1.381976e0
T24186682b_c	Unknown	1.74e5	1.287e6	3.78	37.3	N/A	1.527250e0
T24186683b_a	Unknown	1.49e5	1.219e6	3.78	42.1	N/A	1.378408e0
T24186683b_b	Unknown	1.34e5	1.264e6	3.78	32.8	N/A	1.195306e0
T24186683b_c	Unknown	1.28e5	1.317e6	3.79	23.9	N/A	1.092494e0
T24186684b_a	Unknown	1.86e5	1.242e6	3.79	44.0	N/A	1.691852e0
T24186684b_b	Unknown	1.49e5	1.186e6	3.79	34.8	N/A	1.416151e0
T24186684b_c	Unknown	1.58e5	1.175e6	3.79	30.4	N/A	1.519092e0

Compound name: iPRMP
Regression Equation: y = 0.08766 x + 0.00119 (r = 0.99005) (weighting: 1 / x^2)

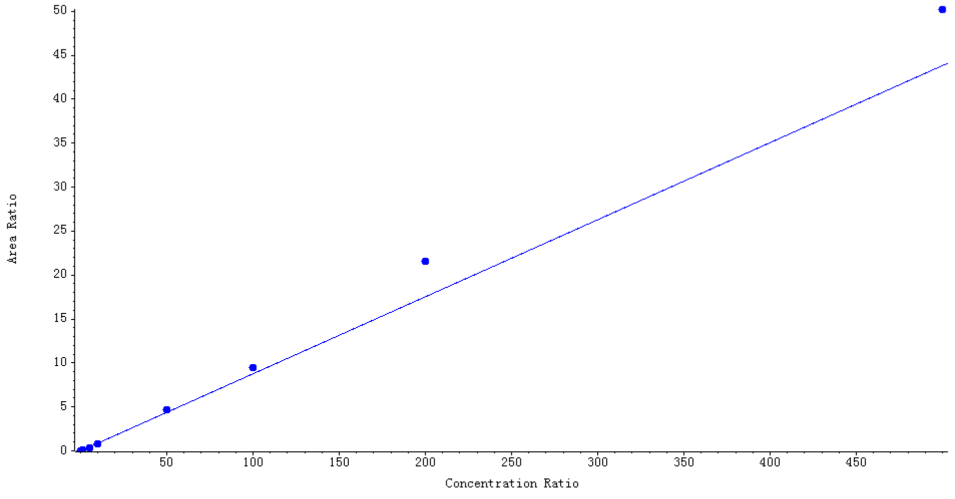

Peak Review

Blank
iPRMP AREA:N/A S/N:N/A

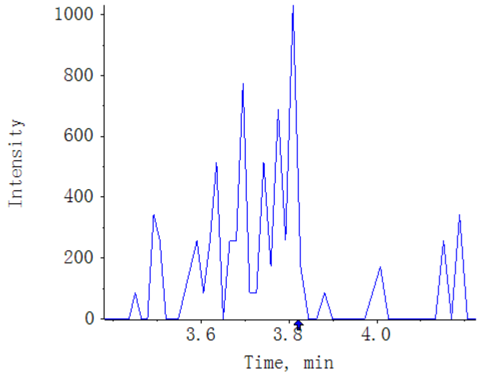

V3.0_MWMS_20240725_1
iPRMP AREA:1.19e6 S/N:52.5

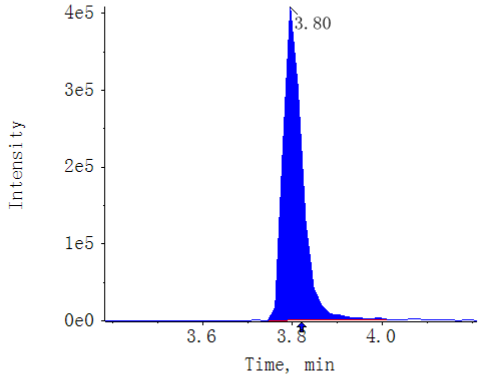

T24186682b_a
iPRMP AREA:1.73e5 S/N:47.2

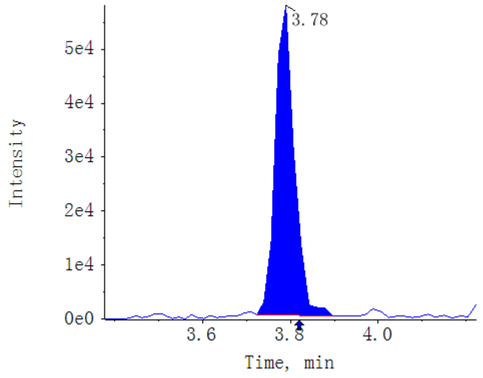

T24186682b_b
iPRMP AREA:1.59e5 S/N:42.0

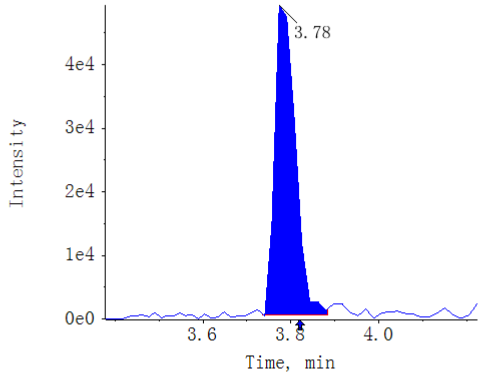

T24186682b_c
iPRMP AREA:1.74e5 S/N:37.3

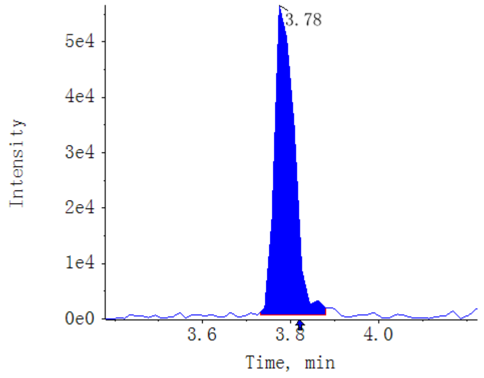

T24186683b_a
iPRMP AREA:1.49e5 S/N:42.1

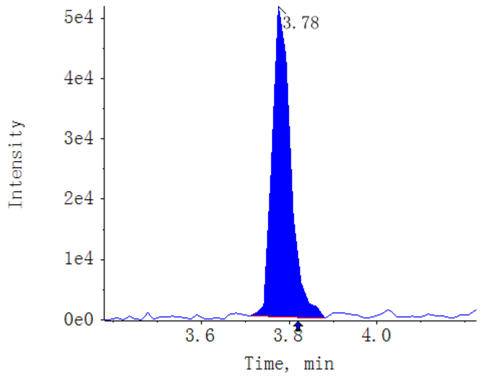

T24186683b_b
iPRMP AREA:1.34e5 S/N:32.8

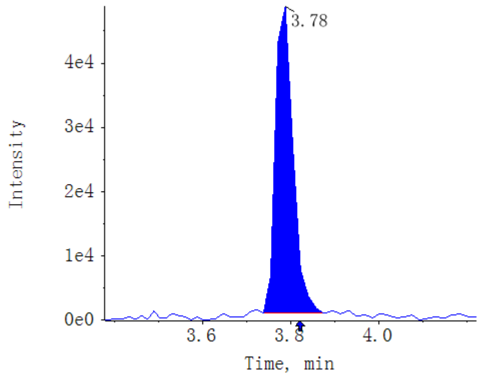

T24186683b_c
iPRMP AREA:1.28e5 S/N:23.9

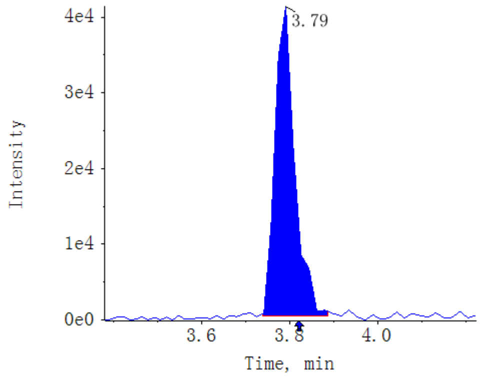

T24186684b_a
iPRMP AREA:1.86e5 S/N:44.0

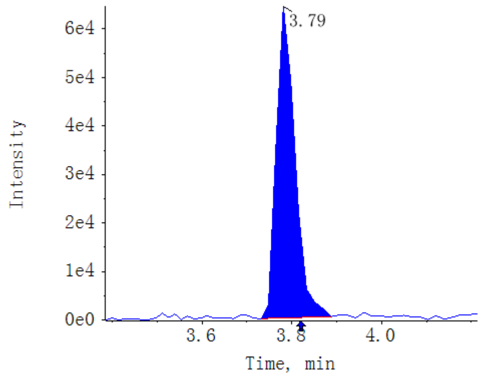

T24186684b_b
iPRMP AREA:1.49e5 S/N:34.8

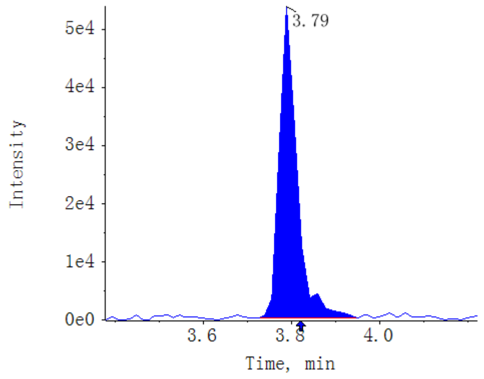

T24186684b_c
iPRMP AREA:1.58e5 S/N:30.4

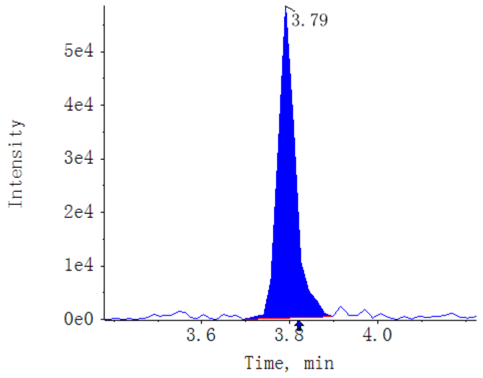

Result Table	MWXS-24-3064-a_9_WH6500-17_A20-3_V6.0_WSS_20240730	Algorithm Used	MQ4
Acquisition Method	ACC-PHs_V6.0_WH6500-17_CMY_20240521.dam	Instrument Name	Triple Quad 6500+ Low Mass
Project	N/A	Analytes QTY	109:60

Compound name: tZ9G (382.0 / 220.1)

Sample Name	Sample Type	Area (cps)	Is Area (cps)	RT (min)	S/N	Target Conc	Calculated Conc.()
STD_0.01ppb	Standard	N/A	1.546e6	N/A	N/A	0.0100	N/A
STD_0.05ppb	Standard	N/A	1.581e6	N/A	N/A	0.0500	N/A
STD_0.1ppb	Standard	1.26e4	1.618e6	2.97	10.6	0.1000	9.780917e-2
STD_0.5ppb	Standard	5.07e4	1.352e6	2.98	29.2	0.5000	5.681746e-1
STD_1ppb	Standard	8.32e4	1.385e6	2.96	39.8	1.0000	9.252990e-1
STD_5ppb	Standard	4.33e5	1.227e6	2.98	57.8	5.0000	5.556920e0
STD_10ppb	Standard	7.50e5	1.204e6	2.97	80.3	10.0000	9.837617e0
STD_50ppb	Standard	4.18e6	1.243e6	2.96	92.2	50.0000	5.324048e1
STD_100ppb	Standard	7.55e6	1.246e6	2.97	108.5	100.0000	9.591982e1
STD_200ppb	Standard	1.53e7	1.273e6	2.96	131.5	200.0000	1.908014e2
STD_500ppb	Standard	3.40e7	1.212e6	2.95	136.6	500.0000	4.435498e2
V2.0_MW_RQC1_20240724	Quality Control	3.88e6	1.973e6	3.10	583.9	0.0000	3.107544e1
Blank	Unknown	N/A	1.128e3	N/A	N/A	N/A	N/A
V3.0_MWMS_20240725_1	Unknown	1.02e6	1.465e6	3.14	134.6	N/A	1.094413e1
MWXS243064a_R1	Quality Control	N/A	1.869e6	N/A	N/A	0.0000	N/A
MWXS243064a_R2	Quality Control	N/A	1.799e6	N/A	N/A	0.0000	N/A
MWXS243064a_R3	Quality Control	N/A	1.915e6	N/A	N/A	0.0000	N/A
T24186682b_a	Unknown	N/A	1.983e6	N/A	N/A	N/A	N/A
T24186682b_b	Unknown	N/A	1.875e6	N/A	N/A	N/A	N/A
T24186682b_c	Unknown	N/A	1.962e6	N/A	N/A	N/A	N/A
T24186683b_a	Unknown	N/A	1.671e6	N/A	N/A	N/A	N/A
T24186683b_b	Unknown	N/A	1.677e6	N/A	N/A	N/A	N/A
T24186683b_c	Unknown	N/A	1.667e6	N/A	N/A	N/A	N/A
T24186684b_a	Unknown	N/A	1.717e6	N/A	N/A	N/A	N/A
T24186684b_b	Unknown	N/A	1.710e6	N/A	N/A	N/A	N/A
T24186684b_c	Unknown	N/A	1.760e6	N/A	N/A	N/A	N/A

Compound name: tZ9G
Regression Equation: y = 0.06319 x + 0.00161 (r = 0.99605) (weighting: 1 / x^2)

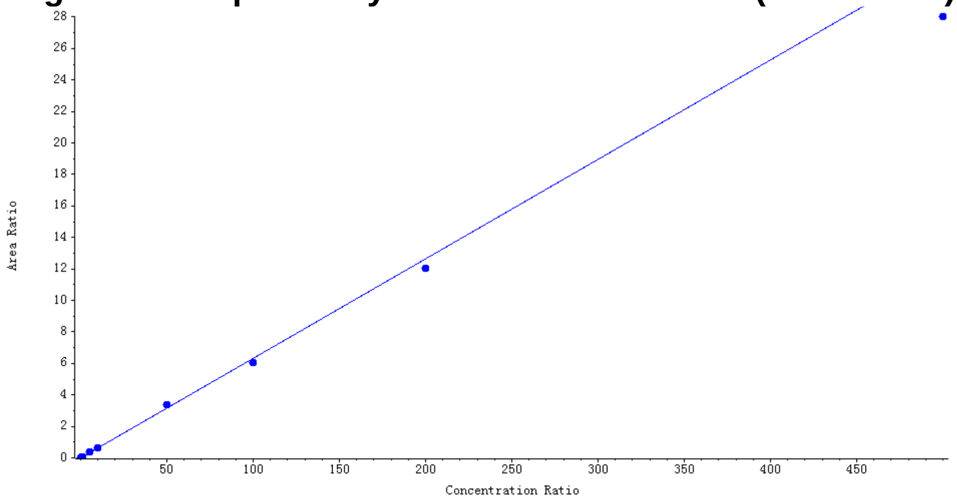

Peak Review

Blank

tZ9G AREA:N/A S/N:N/A

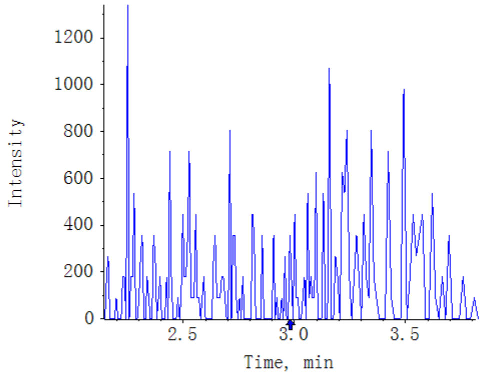

V3.0_MWMS_20240725_1

tZ9G AREA:1.02e6 S/N:134.6

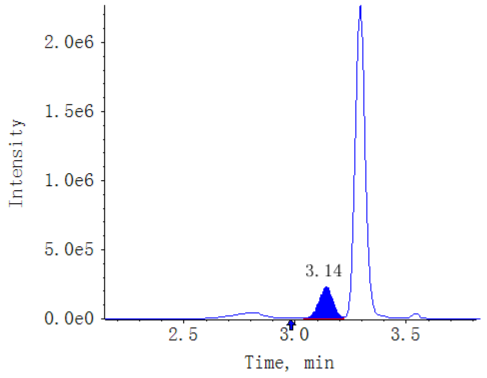

T24186682b_a

tZ9G AREA:N/A S/N:N/A

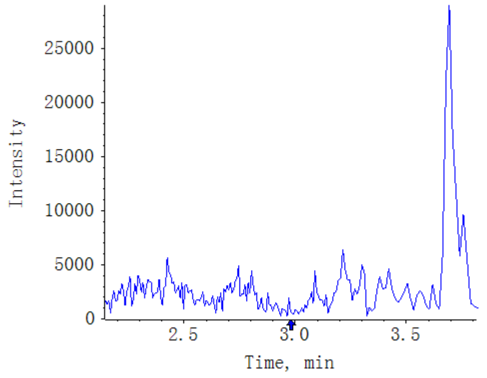

T24186682b_b

tZ9G AREA:N/A S/N:N/A

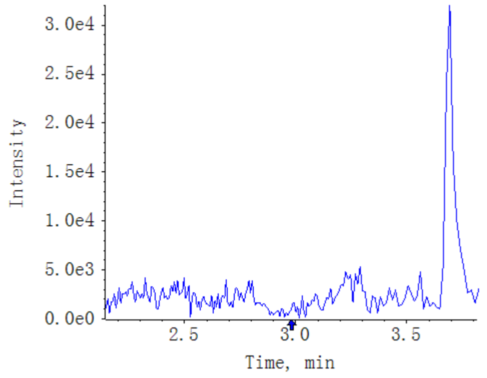

T24186682b_c

tZ9G AREA:N/A S/N:N/A

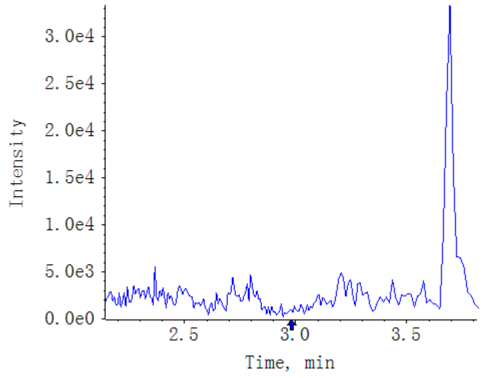

T24186683b_a

tZ9G AREA:N/A S/N:N/A

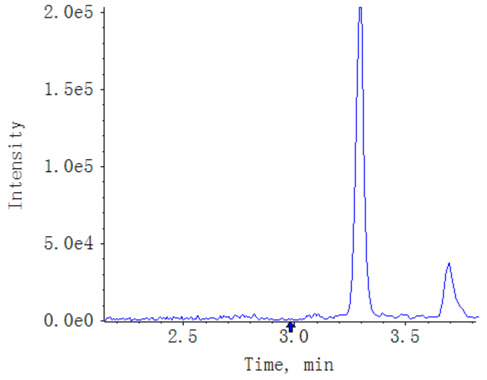

T24186683b_b

tZ9G AREA:N/A S/N:N/A

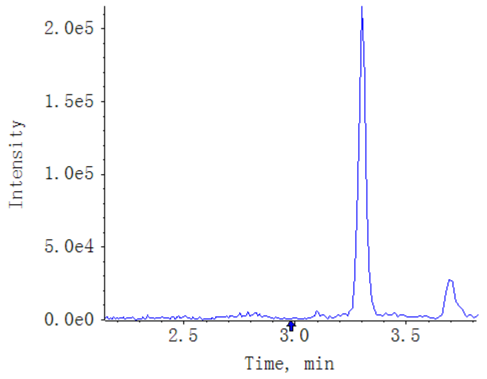

T24186683b_c

tZ9G AREA:N/A S/N:N/A

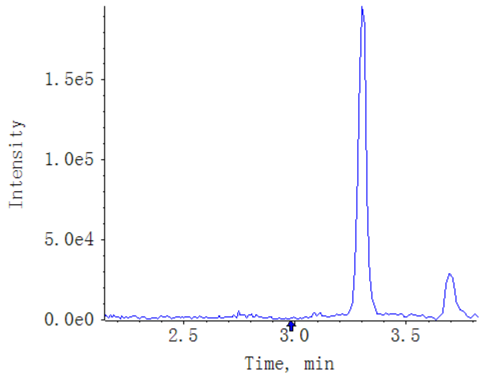

T24186684b_a

tZ9G AREA:N/A S/N:N/A

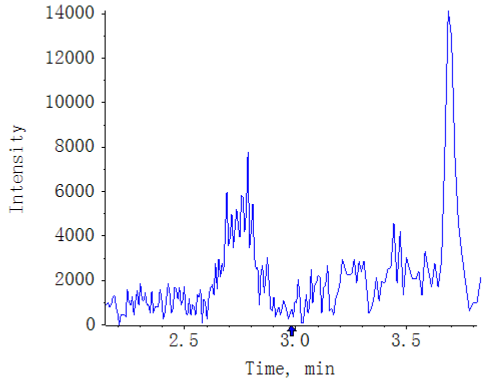

T24186684b_b

tZ9G AREA:N/A S/N:N/A

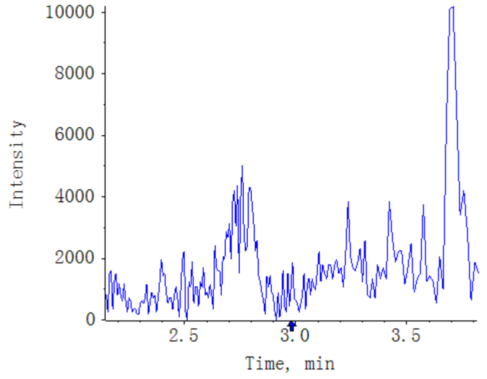

T24186684b_c

tZ9G AREA:N/A S/N:N/A

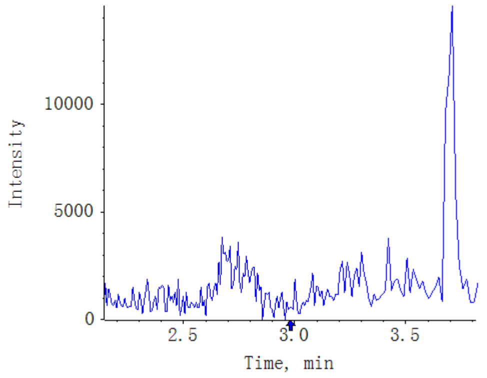

Result Table	MWXS-24-3064-a_9_WH6500-17_A20-3_V6.0_WSS_20240730	Algorithm Used	MQ4
Acquisition Method	ACC-PHs_V6.0_WH6500-17_CMY_20240521.dam	Instrument Name	Triple Quad 6500+ Low Mass
Project	N/A	Analytes QTY	109:61

Compound name: tZRMP (432.0 / 220.1)

Sample Name	Sample Type	Area (cps)	Is Area (cps)	RT (min)	S/N	Target Conc	Calculated Conc.()
STD_0.01ppb	Standard	N/A	3.065e6	N/A	N/A	0.0100	N/A
STD_0.05ppb	Standard	N/A	3.030e6	N/A	N/A	0.0500	N/A
STD_0.1ppb	Standard	N/A	3.037e6	N/A	N/A	0.1000	N/A
STD_0.5ppb	Standard	2.28e4	2.659e6	1.87	28.3	0.5000	5.489522e-1
STD_1ppb	Standard	3.70e4	2.814e6	1.85	31.0	1.0000	8.335897e-1
STD_5ppb	Standard	1.99e5	2.801e6	1.87	68.5	5.0000	4.437321e0
STD_10ppb	Standard	3.79e5	2.618e6	1.87	98.4	10.0000	9.034483e0
STD_50ppb	Standard	2.08e6	2.412e6	1.86	164.3	50.0000	5.370359e1
STD_100ppb	Standard	3.78e6	2.237e6	1.88	201.6	100.0000	1.052967e2
STD_200ppb	Standard	7.75e6	2.096e6	1.87	250.5	200.0000	2.301110e2
STD_500ppb	Standard	N/A	1.785e6	N/A	N/A	500.0000	N/A
V2.0_MW_RQC1_20240724	Quality Control	N/A	1.511e6	N/A	N/A	0.0000	N/A
Blank	Unknown	N/A	9.253e2	N/A	N/A	N/A	N/A
V3.0_MWMS_20240725_1	Unknown	2.03e5	4.177e6	2.14	69.8	N/A	3.043221e0
MWXS243064a_R1	Quality Control	1.27e4	1.316e6	2.07	9.1	0.0000	6.174619e-1
MWXS243064a_R2	Quality Control	1.32e4	1.226e6	2.08	8.8	0.0000	6.848359e-1
MWXS243064a_R3	Quality Control	1.38e4	1.274e6	2.07	6.6	0.0000	6.872018e-1
T24186682b_a	Unknown	1.21e4	1.337e6	2.07	7.0	N/A	5.763948e-1
T24186682b_b	Unknown	1.31e4	1.303e6	2.10	7.0	N/A	6.399842e-1
T24186682b_c	Unknown	1.63e4	1.287e6	2.11	5.6	N/A	8.049671e-1
T24186683b_a	Unknown	N/A	1.219e6	N/A	N/A	N/A	N/A
T24186683b_b	Unknown	N/A	1.264e6	N/A	N/A	N/A	N/A
T24186683b_c	Unknown	N/A	1.317e6	N/A	N/A	N/A	N/A
T24186684b_a	Unknown	2.85e4	1.242e6	2.09	10.3	N/A	1.443546e0
T24186684b_b	Unknown	1.58e4	1.186e6	2.11	8.2	N/A	8.439620e-1
T24186684b_c	Unknown	1.34e4	1.175e6	2.03	8.5	N/A	7.241188e-1

Compound name: tZRMP
Regression Equation: y = 0.01606 x + -2.37232e-4 (r = 0.99073) (weighting: 1 / x^2)

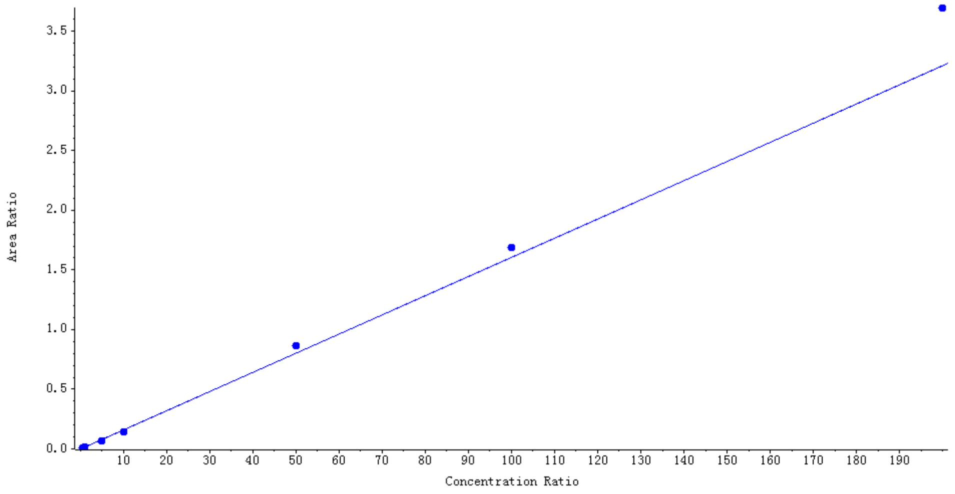

Peak Review

Blank
tZRMP AREA:N/A S/N:N/A

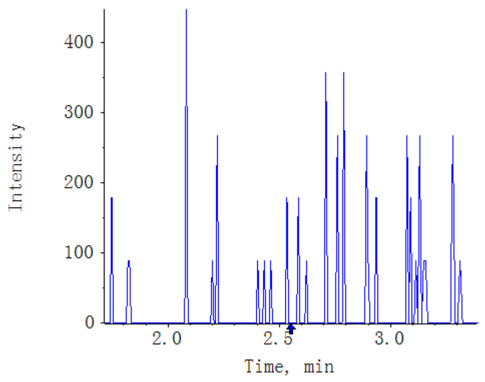

V3.0_MWMS_20240725_1
tZRMP AREA:2.03e5 S/N:69.8

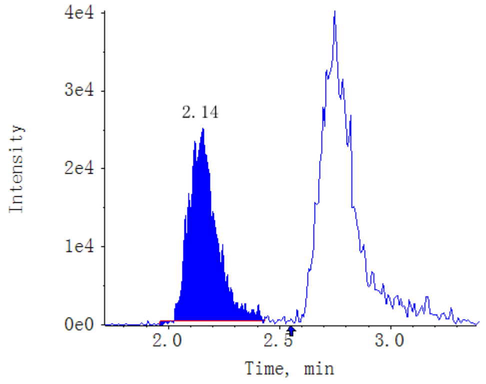

T24186682b_a
tZRMP AREA:1.21e4 S/N:7.0

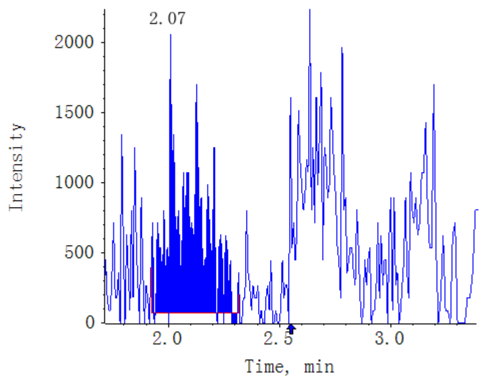

T24186682b_b
tZRMP AREA:1.31e4 S/N:7.0

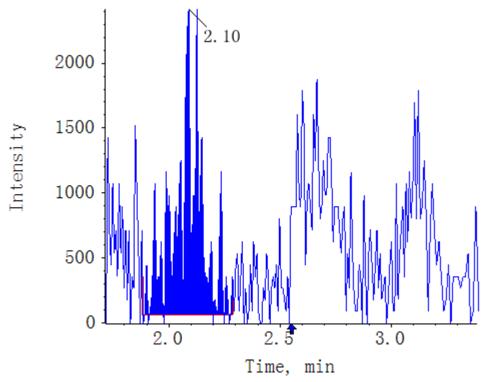

T24186682b_c
tZRMP AREA:1.63e4 S/N:5.6

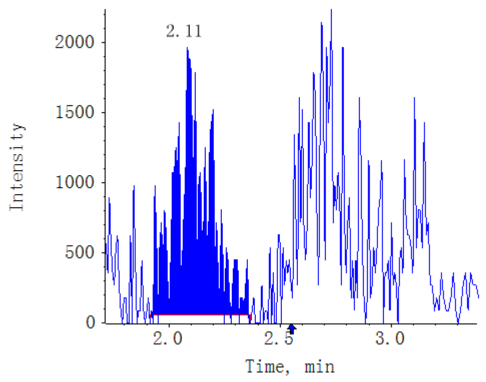

T24186683b_a
tZRMP AREA:N/A S/N:N/A

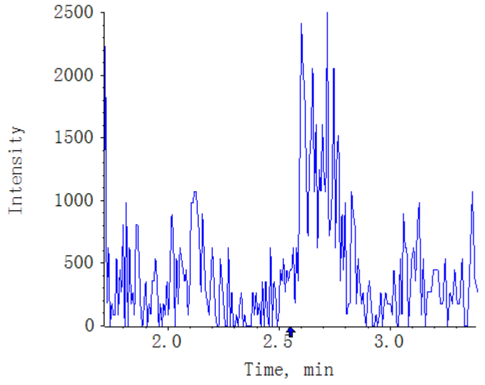

T24186683b_b
tZRMP AREA:N/A S/N:N/A

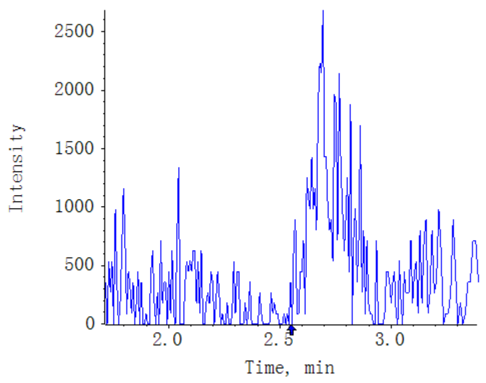

T24186683b_c
tZRMP AREA:N/A S/N:N/A

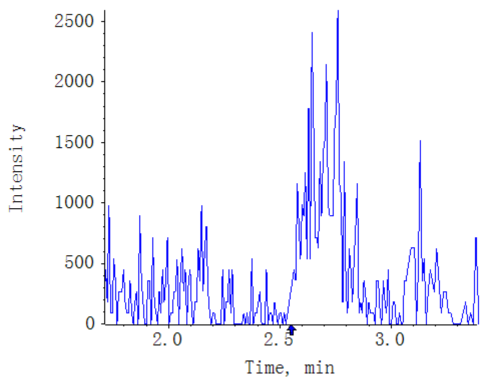

T24186684b_a
tZRMP AREA:2.85e4 S/N:10.3

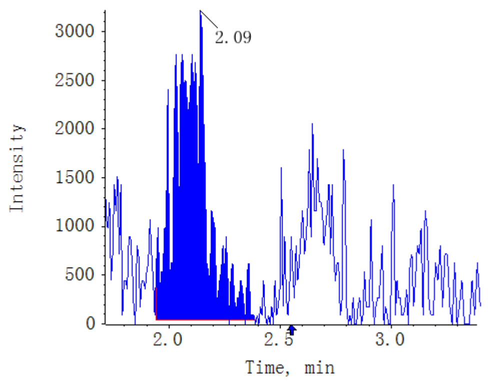

T24186684b_b
tZRMP AREA:1.58e4 S/N:8.2

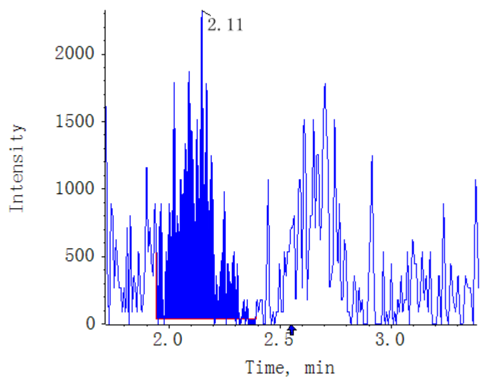

T24186684b_c
tZRMP AREA:1.34e4 S/N:8.5

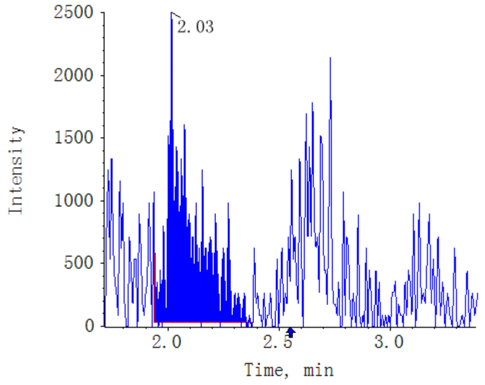

Result Table	MWXS-24-3064-a_9_WH6500-17_A20-3_V6.0_WSS_20240730	Algorithm Used	MQ4
Acquisition Method	ACC-PHs_V6.0_WH6500-17_CMY_20240521.dam	Instrument Name	Triple Quad 6500+ Low Mass
Project	N/A	Analytes QTY	109:62

Compound name: JA-Phe (358.2 / 120.2)

Sample Name	Sample Type	Area (cps)	Is Area (cps)	RT (min)	S/N	Target Conc	Calculated Conc.()
STD_0.01ppb	Standard	2.45e3	7.502e5	6.40	6.8	0.0100	9.797176e-3
STD_0.05ppb	Standard	1.43e4	7.505e5	6.40	23.7	0.0500	5.658189e-2
STD_0.1ppb	Standard	2.41e4	7.760e5	6.41	31.0	0.1000	9.180742e-2
STD_0.5ppb	Standard	1.21e5	6.580e5	6.40	44.0	0.5000	5.450079e-1
STD_1ppb	Standard	2.17e5	6.418e5	6.40	40.2	1.0000	9.968520e-1
STD_5ppb	Standard	1.51e6	7.441e5	6.42	61.6	5.0000	5.972648e0
STD_10ppb	Standard	2.62e6	7.645e5	6.40	42.2	10.0000	1.012793e1
STD_50ppb	Standard	1.36e7	8.212e5	6.40	47.5	50.0000	4.896105e1
STD_100ppb	Standard	2.51e7	8.032e5	6.41	40.3	100.0000	9.209530e1
STD_200ppb	Standard	4.93e7	9.369e5	6.41	41.8	200.0000	1.552412e2
STD_500ppb	Standard	N/A	8.441e5	N/A	N/A	500.0000	N/A
V2.0_MW_RQC1_20240724	Quality Control	N/A	3.905e5	N/A	N/A	0.0000	N/A
Blank	Unknown	N/A	5.961e3	N/A	N/A	N/A	N/A
V3.0_MWMS_20240725_1	Unknown	1.12e6	1.232e6	6.38	48.0	N/A	2.677002e0
MWXS243064a_R1	Quality Control	N/A	3.925e5	N/A	N/A	0.0000	N/A
MWXS243064a_R2	Quality Control	N/A	3.390e5	N/A	N/A	0.0000	N/A
MWXS243064a_R3	Quality Control	N/A	3.379e5	N/A	N/A	0.0000	N/A
T24186682b_a	Unknown	N/A	3.024e5	N/A	N/A	N/A	N/A
T24186682b_b	Unknown	N/A	2.893e5	N/A	N/A	N/A	N/A
T24186682b_c	Unknown	N/A	2.806e5	N/A	N/A	N/A	N/A
T24186683b_a	Unknown	N/A	2.778e5	N/A	N/A	N/A	N/A
T24186683b_b	Unknown	N/A	3.603e5	N/A	N/A	N/A	N/A
T24186683b_c	Unknown	N/A	3.184e5	N/A	N/A	N/A	N/A
T24186684b_a	Unknown	N/A	3.496e5	N/A	N/A	N/A	N/A
T24186684b_b	Unknown	N/A	3.022e5	N/A	N/A	N/A	N/A
T24186684b_c	Unknown	N/A	2.908e5	N/A	N/A	N/A	N/A

Compound name: JA-Phe
Regression Equation: y = 0.33884 x + -5.62064e-5 (r = 0.99242) (weighting: 1 / x^2)

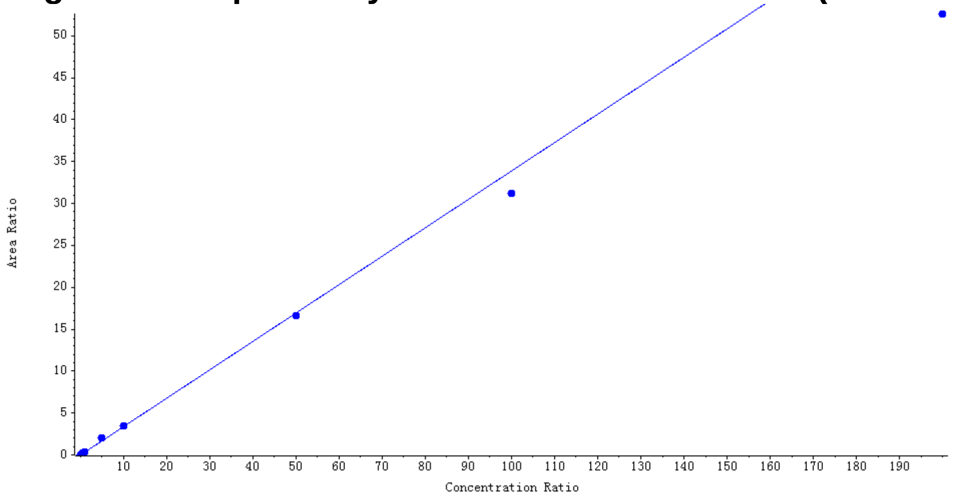

Peak Review

Blank
JA-Phe AREA:N/A S/N:N/A

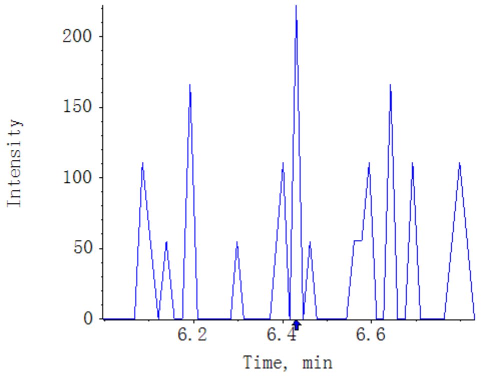

V3.0_MWMS_20240725_1
JA-Phe AREA:1.12e6 S/N:48.0

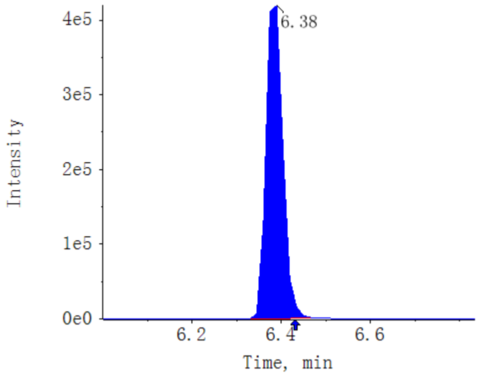

T24186682b_a
JA-Phe AREA:N/A S/N:N/A

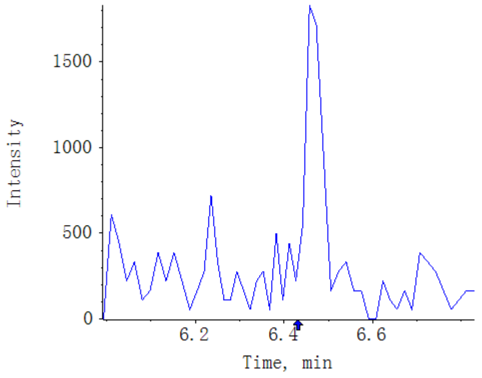

T24186682b_b
JA-Phe AREA:N/A S/N:N/A

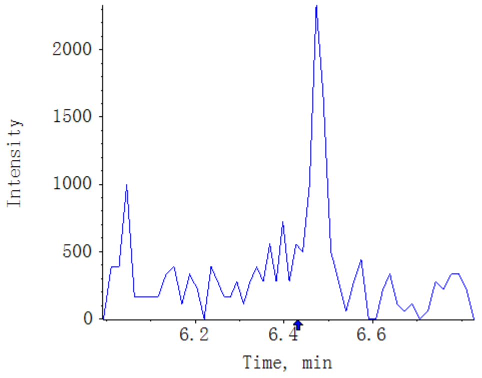

T24186682b_c
JA-Phe AREA:N/A S/N:N/A

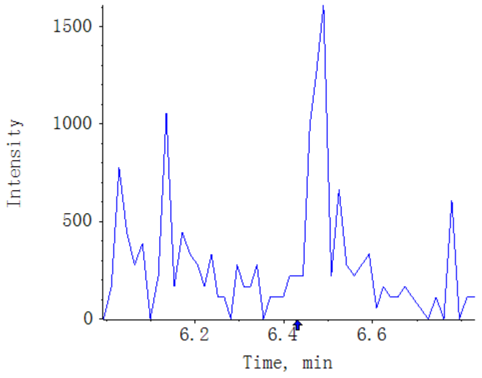

T24186683b_a
JA-Phe AREA:N/A S/N:N/A

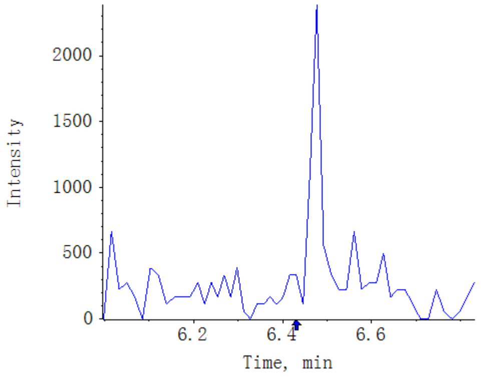

T24186683b_b
JA-Phe AREA:N/A S/N:N/A

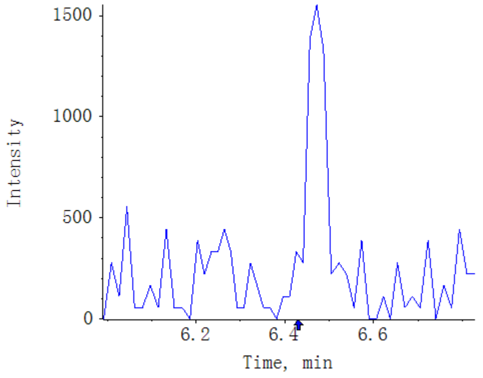

T24186683b_c
JA-Phe AREA:N/A S/N:N/A

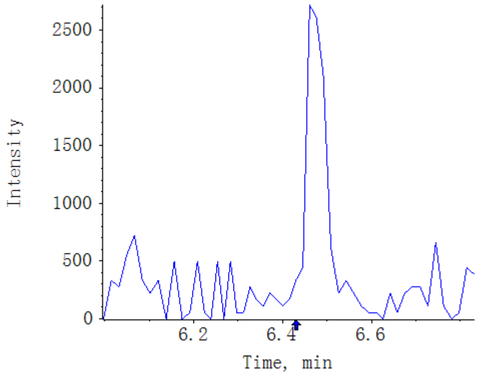

T24186684b_a
JA-Phe AREA:N/A S/N:N/A

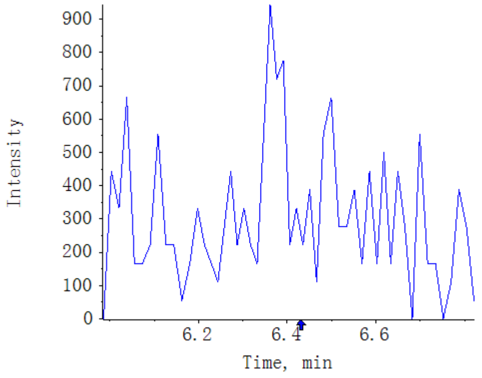

T24186684b_b
JA-Phe AREA:N/A S/N:N/A

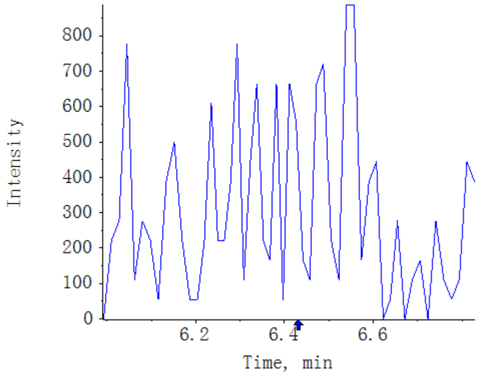

T24186684b_c
JA-Phe AREA:N/A S/N:N/A

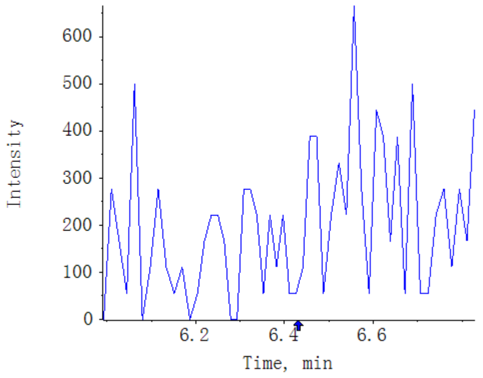

Result Table	MWXS-24-3064-a_9_WH6500-17_A20-3_V6.0_WSS_20240730	Algorithm Used	MQ4
Acquisition Method	ACC-PHs_V6.0_WH6500-17_CMY_20240521.dam	Instrument Name	Triple Quad 6500+ Low Mass
Project	N/A	Analytes QTY	109:63

Compound name: OPC-4 (239.1 / 221.3)

Sample Name	Sample Type	Area (cps)	Is Area (cps)	RT (min)	S/N	Target Conc	Calculated Conc.()
STD_0.01ppb	Standard	N/A	7.502e5	N/A	N/A	0.0100	N/A
STD_0.05ppb	Standard	N/A	7.505e5	N/A	N/A	0.0500	N/A
STD_0.1ppb	Standard	N/A	7.760e5	N/A	N/A	0.1000	N/A
STD_0.5ppb	Standard	1.74e4	6.580e5	6.41	8.0	0.5000	5.178477e-1
STD_1ppb	Standard	2.69e4	6.418e5	6.41	11.9	1.0000	8.908255e-1
STD_5ppb	Standard	1.82e5	7.441e5	6.43	26.8	5.0000	5.812467e0
STD_10ppb	Standard	3.42e5	7.645e5	6.41	28.8	10.0000	1.072050e1
STD_50ppb	Standard	1.65e6	8.212e5	6.41	41.2	50.0000	4.845365e1
STD_100ppb	Standard	2.89e6	8.032e5	6.42	35.0	100.0000	8.698630e1
STD_200ppb	Standard	N/A	9.369e5	N/A	N/A	200.0000	N/A
STD_500ppb	Standard	N/A	8.441e5	N/A	N/A	500.0000	N/A
V2.0_MW_RQC1_20240724	Quality Control	1.75e5	3.905e5	6.38	6.9	0.0000	1.076073e1
Blank	Unknown	N/A	5.961e3	N/A	N/A	N/A	N/A
V3.0_MWMS_20240725_1	Unknown	2.56e5	1.232e6	6.39	23.7	N/A	4.904749e0
MWXS243064a_R1	Quality Control	N/A	3.925e5	N/A	N/A	0.0000	N/A
MWXS243064a_R2	Quality Control	N/A	3.390e5	N/A	N/A	0.0000	N/A
MWXS243064a_R3	Quality Control	N/A	3.379e5	N/A	N/A	0.0000	N/A
T24186682b_a	Unknown	N/A	3.024e5	N/A	N/A	N/A	N/A
T24186682b_b	Unknown	N/A	2.893e5	N/A	N/A	N/A	N/A
T24186682b_c	Unknown	N/A	2.806e5	N/A	N/A	N/A	N/A
T24186683b_a	Unknown	N/A	2.778e5	N/A	N/A	N/A	N/A
T24186683b_b	Unknown	N/A	3.603e5	N/A	N/A	N/A	N/A
T24186683b_c	Unknown	N/A	3.184e5	N/A	N/A	N/A	N/A
T24186684b_a	Unknown	N/A	3.496e5	N/A	N/A	N/A	N/A
T24186684b_b	Unknown	N/A	3.022e5	N/A	N/A	N/A	N/A
T24186684b_c	Unknown	N/A	2.908e5	N/A	N/A	N/A	N/A

Compound name: OPC-4
Regression Equation: y = 0.04125 x + 0.00510 (r = 0.99186) (weighting: 1 / x^2)

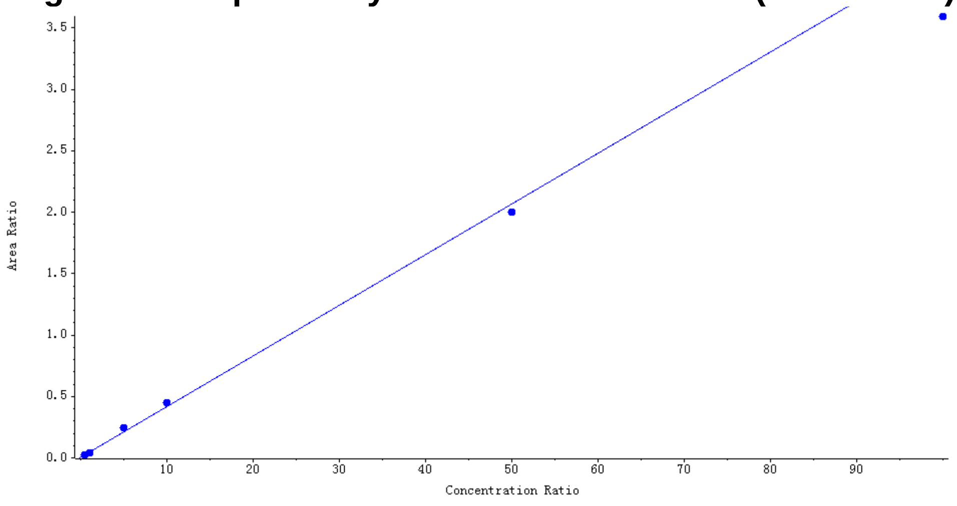

Peak Review

Blank
OPC-4 AREA:N/A S/N:N/A

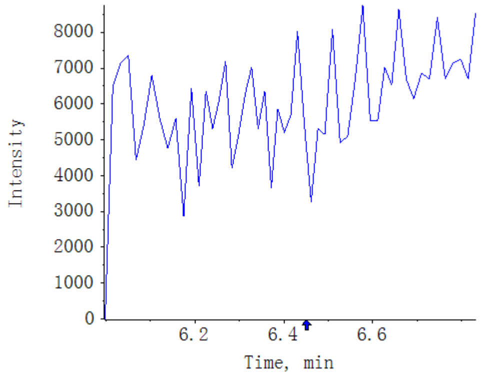

V3.0_MWMS_20240725_1
OPC-4 AREA:2.56e5 S/N:23.7

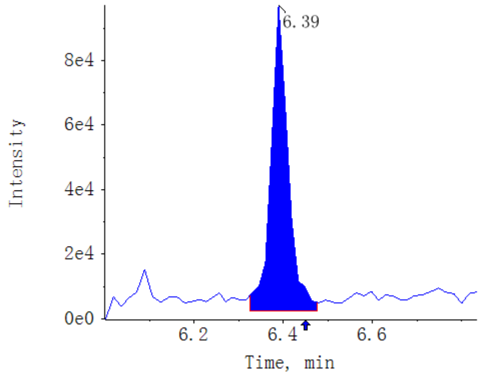

T24186682b_a
OPC-4 AREA:N/A S/N:N/A

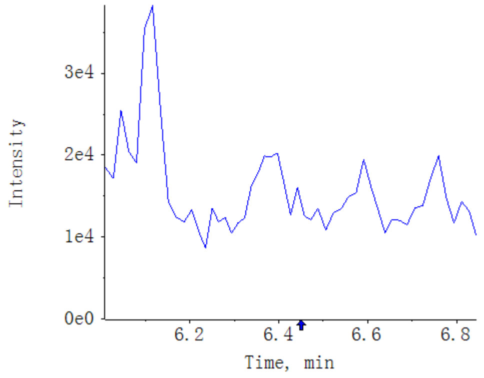

T24186682b_b
OPC-4 AREA:N/A S/N:N/A

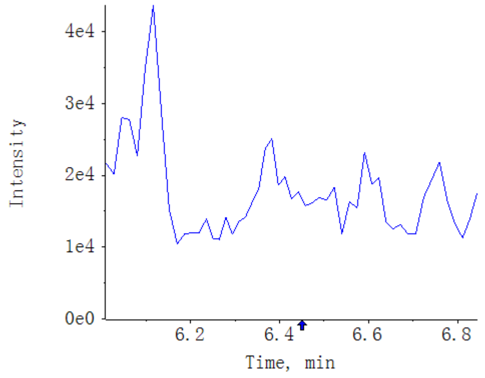

T24186682b_c
OPC-4 AREA:N/A S/N:N/A

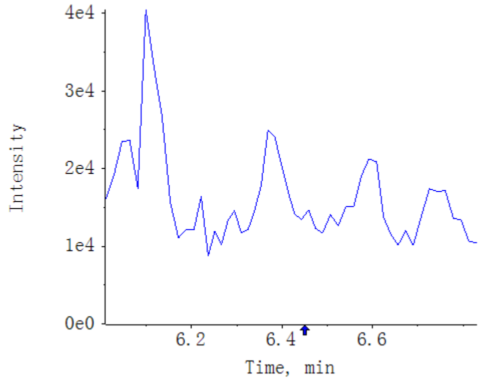

T24186683b_a
OPC-4 AREA:N/A S/N:N/A

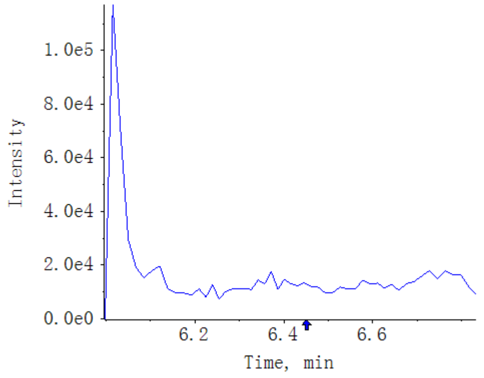

T24186683b_b
OPC-4 AREA:N/A S/N:N/A

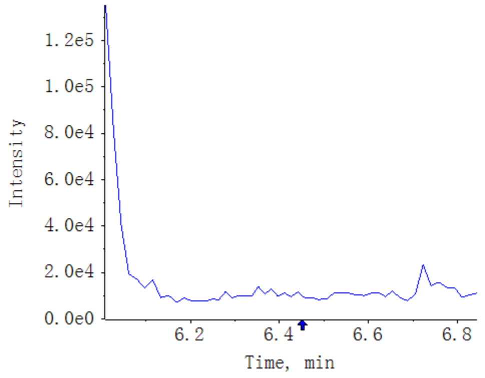

T24186683b_c
OPC-4 AREA:N/A S/N:N/A

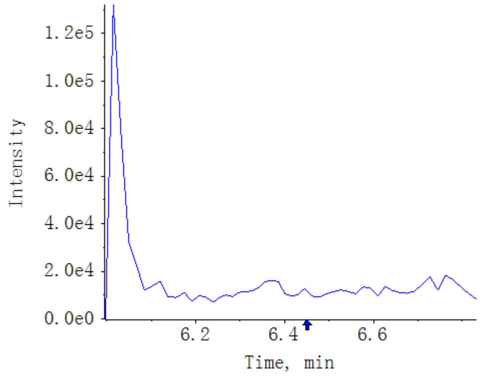

T24186684b_a
OPC-4 AREA:N/A S/N:N/A

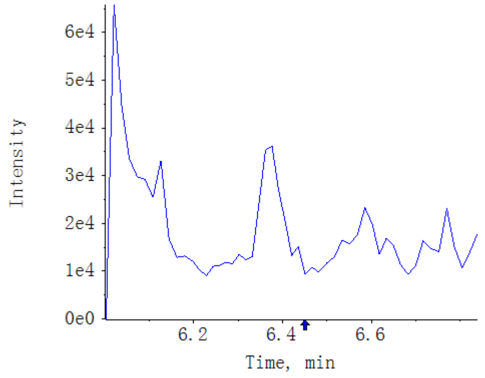

T24186684b_b
OPC-4 AREA:N/A S/N:N/A

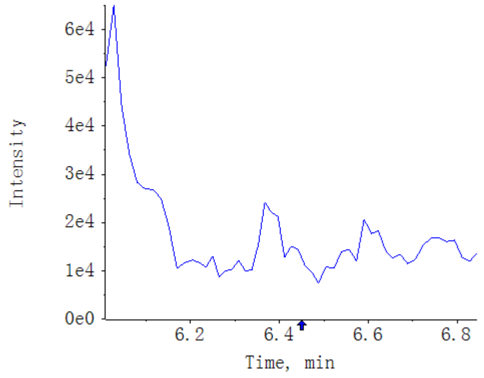

T24186684b_c
OPC-4 AREA:N/A S/N:N/A

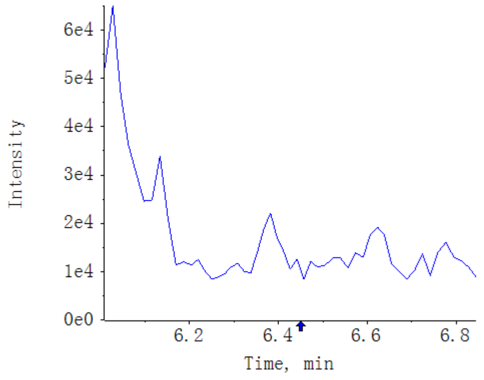

Result Table	MWXS-24-3064-a_9_WH6500-17_A20-3_V6.0_WSS_20240730	Algorithm Used	MQ4
Acquisition Method	ACC-PHs_V6.0_WH6500-17_CMY_20240521.dam	Instrument Name	Triple Quad 6500+ Low Mass
Project	N/A	Analytes QTY	109:64

Compound name: OPC-6 (267.2 / 249.2)

Sample Name	Sample Type	Area (cps)	Is Area (cps)	RT (min)	S/N	Target Conc	Calculated Conc.()
STD_0.01ppb	Standard	N/A	7.502e5	N/A	N/A	0.0100	N/A
STD_0.05ppb	Standard	N/A	7.505e5	N/A	N/A	0.0500	N/A
STD_0.1ppb	Standard	N/A	7.760e5	N/A	N/A	0.1000	N/A
STD_0.5ppb	Standard	2.46e4	6.580e5	7.02	8.2	0.5000	3.913178e-1
STD_1ppb	Standard	4.75e4	6.418e5	7.02	12.5	1.0000	9.437314e-1
STD_5ppb	Standard	2.98e5	7.441e5	7.03	38.5	5.0000	5.888467e0
STD_10ppb	Standard	5.38e5	7.645e5	7.02	43.9	10.0000	1.047172e1
STD_50ppb	Standard	2.93e6	8.212e5	7.02	47.7	50.0000	5.379234e1
STD_100ppb	Standard	5.41e6	8.032e5	7.03	47.4	100.0000	1.017206e2
STD_200ppb	Standard	1.18e7	9.369e5	7.02	58.7	200.0000	1.897103e2
STD_500ppb	Standard	2.81e7	8.441e5	7.02	58.7	500.0000	5.035815e2
V2.0_MW_RQC1_20240724	Quality Control	N/A	3.905e5	N/A	N/A	0.0000	N/A
Blank	Unknown	N/A	5.961e3	N/A	N/A	N/A	N/A
V3.0_MWMS_20240725_1	Unknown	3.25e5	1.232e6	7.01	26.0	N/A	3.808257e0
MWXS243064a_R1	Quality Control	N/A	3.925e5	N/A	N/A	0.0000	N/A
MWXS243064a_R2	Quality Control	N/A	3.390e5	N/A	N/A	0.0000	N/A
MWXS243064a_R3	Quality Control	N/A	3.379e5	N/A	N/A	0.0000	N/A
T24186682b_a	Unknown	2.45e5	3.024e5	7.02	8.2	N/A	1.208056e1
T24186682b_b	Unknown	2.36e5	2.893e5	7.03	8.4	N/A	1.214083e1
T24186682b_c	Unknown	1.91e5	2.806e5	7.02	5.9	N/A	1.010239e1
T24186683b_a	Unknown	N/A	2.778e5	N/A	N/A	N/A	N/A
T24186683b_b	Unknown	N/A	3.603e5	N/A	N/A	N/A	N/A
T24186683b_c	Unknown	N/A	3.184e5	N/A	N/A	N/A	N/A
T24186684b_a	Unknown	N/A	3.496e5	N/A	N/A	N/A	N/A
T24186684b_b	Unknown	N/A	3.022e5	N/A	N/A	N/A	N/A
T24186684b_c	Unknown	N/A	2.908e5	N/A	N/A	N/A	N/A

Compound name: OPC-6
Regression Equation: y = 0.06611 x + 0.01156 (r = 0.99936) (weighting: 1 / x)

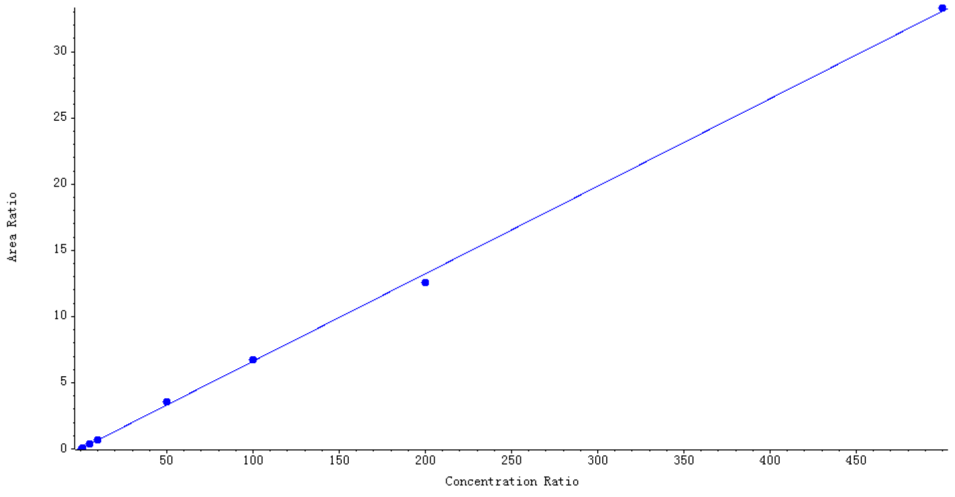

Peak Review

Blank
OPC-6 AREA:N/A S/N:N/A

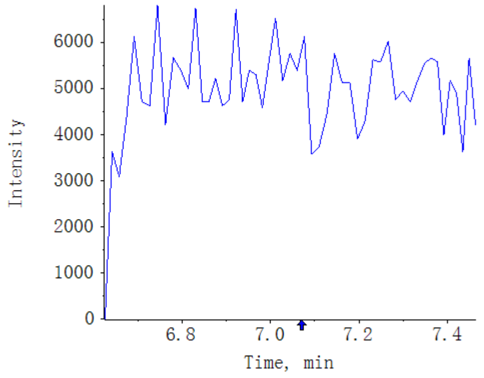

V3.0_MWMS_20240725_1
OPC-6 AREA:3.25e5 S/N:26.0

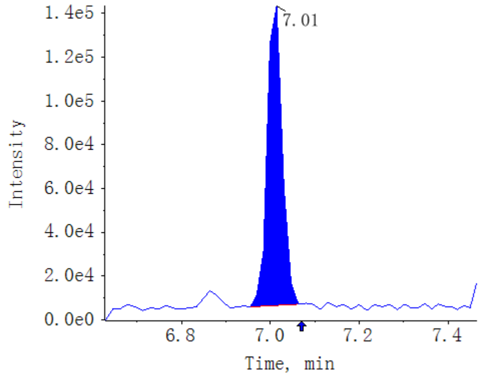

T24186682b_a
OPC-6 AREA:2.45e5 S/N:8.2

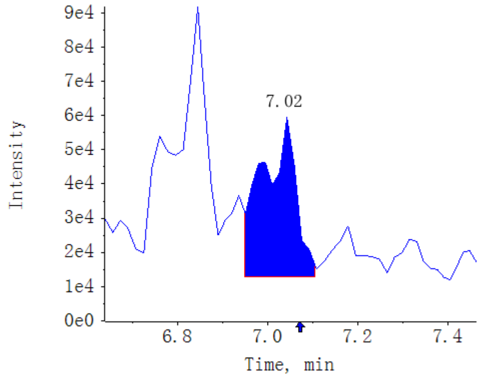

T24186682b_b
OPC-6 AREA:2.36e5 S/N:8.4

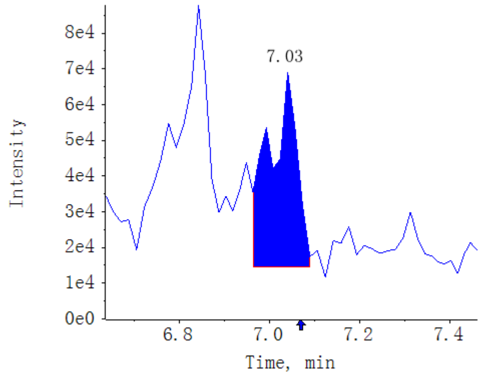

T24186682b_c
OPC-6 AREA:1.91e5 S/N:5.9

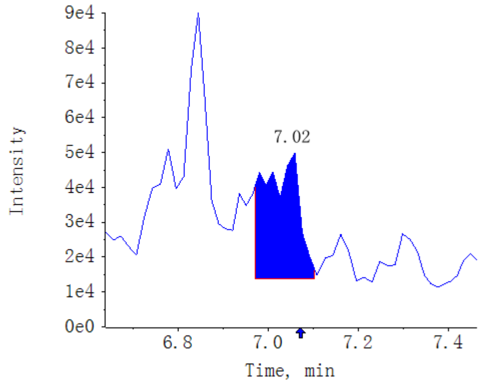

T24186683b_a
OPC-6 AREA:N/A S/N:N/A

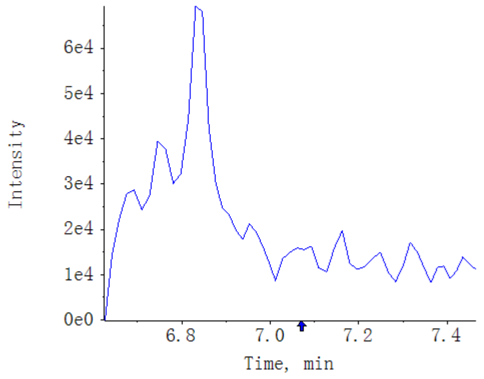

T24186683b_b
OPC-6 AREA:N/A S/N:N/A

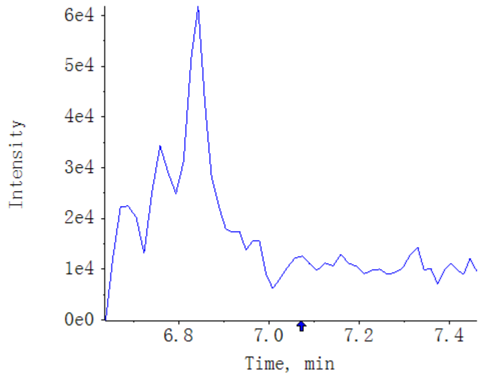

T24186683b_c
OPC-6 AREA:N/A S/N:N/A

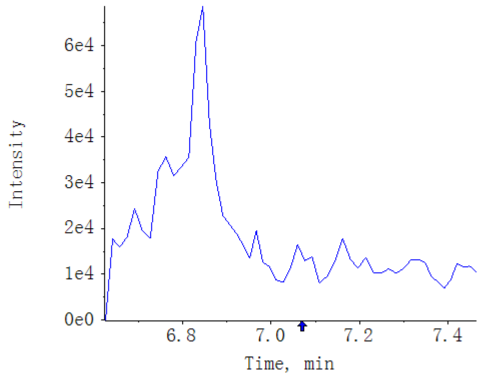

T24186684b_a
OPC-6 AREA:N/A S/N:N/A

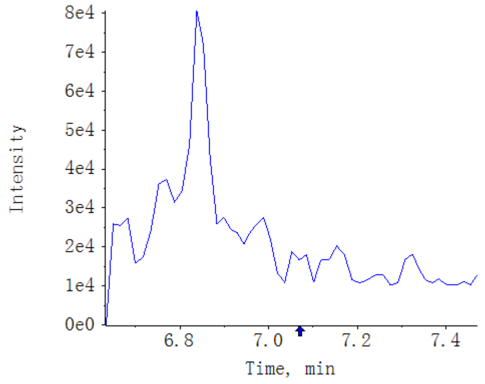

T24186684b_b
OPC-6 AREA:N/A S/N:N/A

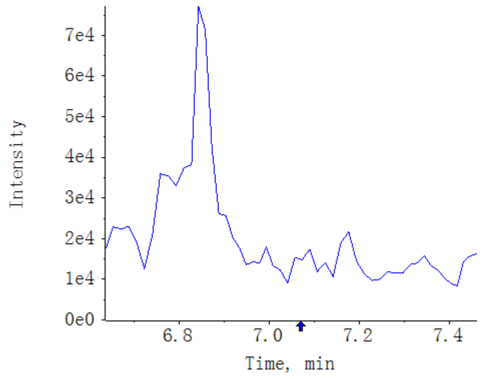

T24186684b_c
OPC-6 AREA:N/A S/N:N/A

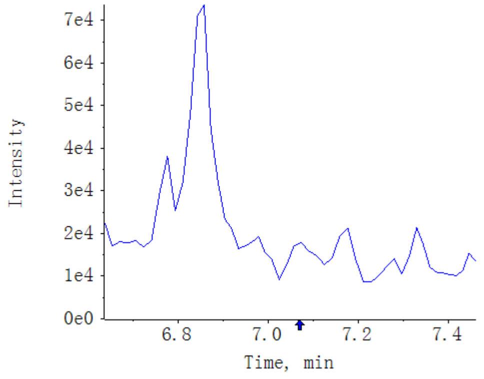

Result Table	MWXS-24-3064-a_9_WH6500-17_A20-3_V6.0_WSS_20240730	Algorithm Used	MQ4
Acquisition Method	ACC-PHs_V6.0_WH6500-17_CMY_20240521.dam	Instrument Name	Triple Quad 6500+ Low Mass
Project	N/A	Analytes QTY	109:65

Compound name: MEJA (225.2 / 151.2)

Sample Name	Sample Type	Area (cps)	Is Area (cps)	RT (min)	S/N	Target Conc	Calculated Conc.()
STD_0.01ppb	Standard	N/A	2.309e5	N/A	N/A	0.0100	N/A
STD_0.05ppb	Standard	N/A	2.305e5	N/A	N/A	0.0500	N/A
STD_0.1ppb	Standard	9.96e3	2.353e5	6.93	7.1	0.1000	1.372316e-1
STD_0.5ppb	Standard	4.08e4	2.276e5	6.92	25.7	0.5000	5.021242e-1
STD_1ppb	Standard	6.66e4	2.419e5	6.92	25.6	1.0000	7.578073e-1
STD_5ppb	Standard	4.55e5	2.490e5	6.93	31.7	5.0000	4.887456e0
STD_10ppb	Standard	8.25e5	2.424e5	6.92	40.1	10.0000	9.080093e0
STD_50ppb	Standard	4.35e6	2.297e5	6.91	46.7	50.0000	5.041809e1
STD_100ppb	Standard	8.12e6	2.311e5	6.92	34.1	100.0000	9.353596e1
STD_200ppb	Standard	1.68e7	2.163e5	6.92	42.7	200.0000	2.072812e2
STD_500ppb	Standard	N/A	1.818e5	N/A	N/A	500.0000	N/A
V2.0_MW_RQC1_20240724	Quality Control	3.73e5	8.369e4	6.86	34.0	0.0000	1.188084e1
Blank	Unknown	N/A	6.913e2	N/A	N/A	N/A	N/A
V3.0_MWMS_20240725_1	Unknown	4.70e5	1.949e5	6.90	39.3	N/A	6.443970e0
MWXS243064a_R1	Quality Control	N/A	7.628e4	N/A	N/A	0.0000	N/A
MWXS243064a_R2	Quality Control	N/A	8.083e4	N/A	N/A	0.0000	N/A
MWXS243064a_R3	Quality Control	N/A	8.154e4	N/A	N/A	0.0000	N/A
T24186682b_a	Unknown	2.23e4	6.346e4	6.89	5.2	N/A	9.578204e-1
T24186682b_b	Unknown	2.17e4	6.319e4	6.90	7.8	N/A	9.371572e-1
T24186682b_c	Unknown	1.44e4	6.814e4	6.89	5.0	N/A	5.854630e-1
T24186683b_a	Unknown	N/A	7.158e4	N/A	N/A	N/A	N/A
T24186683b_b	Unknown	N/A	7.473e4	N/A	N/A	N/A	N/A
T24186683b_c	Unknown	N/A	7.565e4	N/A	N/A	N/A	N/A
T24186684b_a	Unknown	2.60e4	7.124e4	6.87	6.1	N/A	9.976420e-1
T24186684b_b	Unknown	2.63e4	8.095e4	6.89	6.4	N/A	8.883228e-1
T24186684b_c	Unknown	2.24e4	7.778e4	6.89	7.5	N/A	7.896203e-1

Compound name: MEJA
Regression Equation: y = 0.37569 x + -0.00923 (r = 0.99883) (weighting: 1 / x)

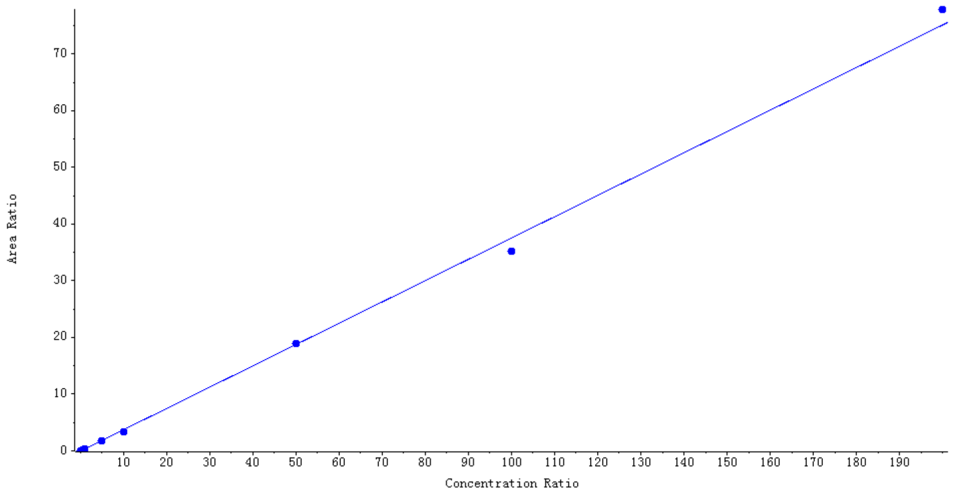

Peak Review

Blank
MEJA AREA:N/A S/N:N/A

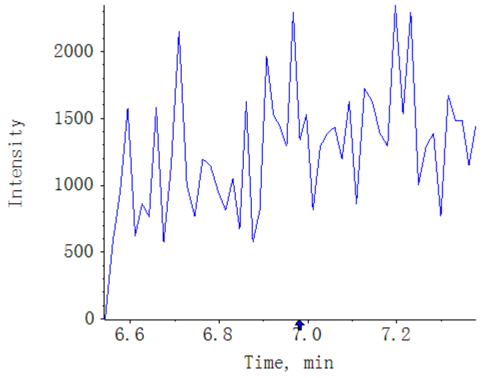

V3.0_MWMS_20240725_1
MEJA AREA:4.70e5 S/N:39.3

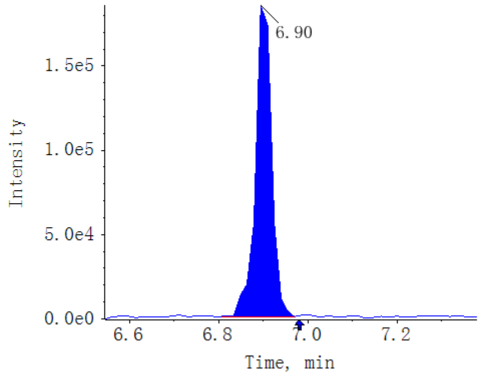

T24186682b_a
MEJA AREA:2.23e4 S/N:5.2

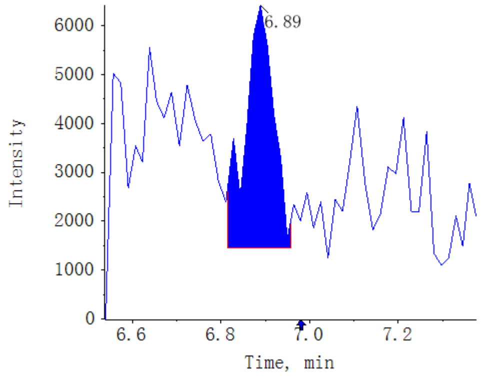

T24186682b_b
MEJA AREA:2.17e4 S/N:7.8

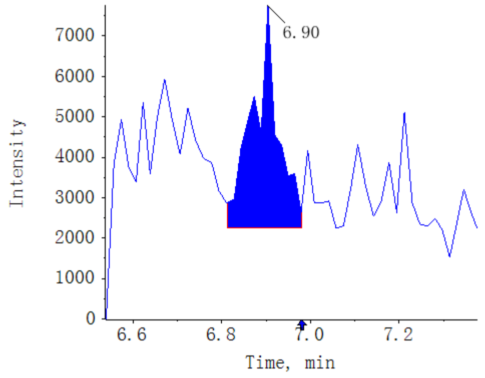

T24186682b_c
MEJA AREA:1.44e4 S/N:5.0

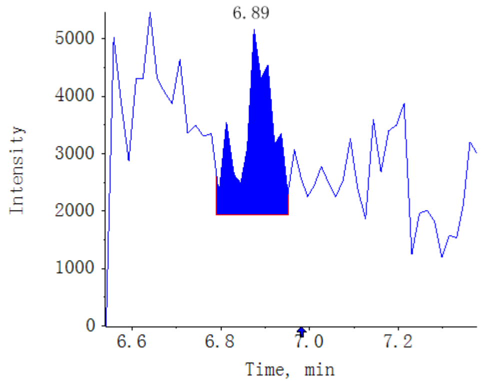

T24186683b_a
MEJA AREA:N/A S/N:N/A

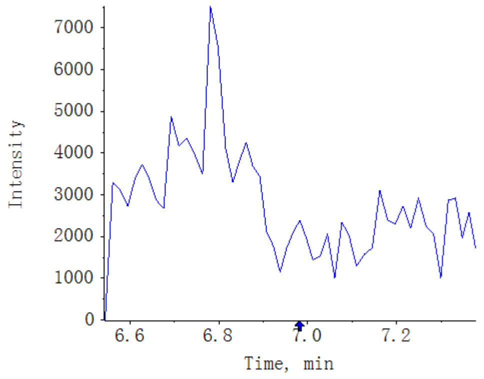

T24186683b_b
MEJA AREA:N/A S/N:N/A

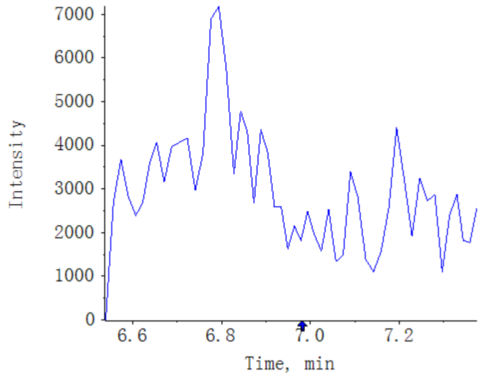

T24186683b_c
MEJA AREA:N/A S/N:N/A

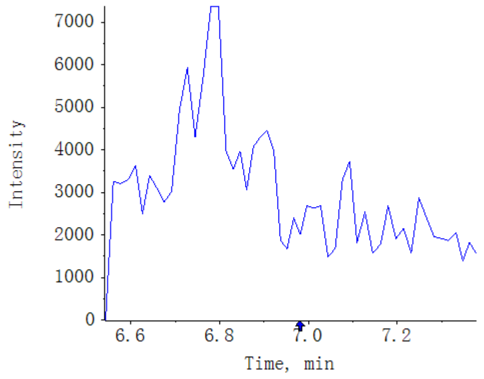

T24186684b_a
MEJA AREA:2.60e4 S/N:6.1

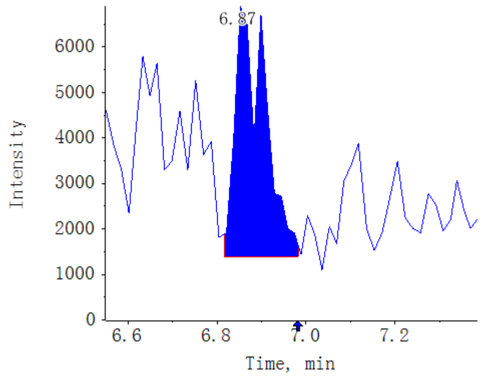

T24186684b_b
MEJA AREA:2.63e4 S/N:6.4

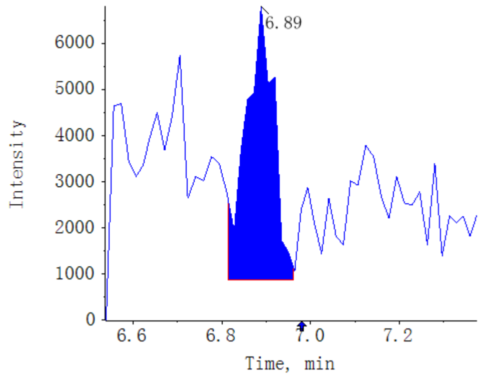

T24186684b_c
MEJA AREA:2.24e4 S/N:7.5

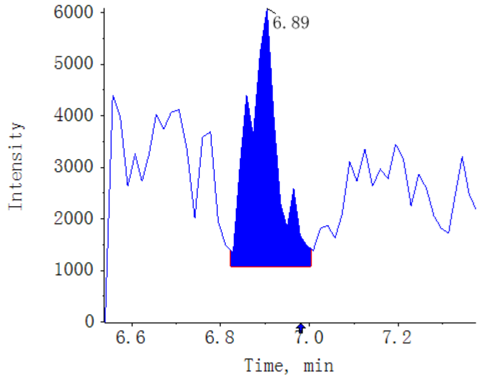

Result Table	MWXS-24-3064-a_9_WH6500-17_A20-3_V6.0_WSS_20240730	Algorithm Used	MQ4
Acquisition Method	ACC-PHs_V6.0_WH6500-17_CMY_20240521.dam	Instrument Name	Triple Quad 6500+ Low Mass
Project	N/A	Analytes QTY	109:66

Compound name: OPDA (293.1 / 275.2)

Sample Name	Sample Type	Area (cps)	Is Area (cps)	RT (min)	S/N	Target Conc	Calculated Conc.()
STD_0.01ppb	Standard	N/A	7.502e5	N/A	N/A	0.0100	N/A
STD_0.05ppb	Standard	4.74e4	7.505e5	7.26	14.4	0.0500	4.872466e-2
STD_0.1ppb	Standard	9.48e4	7.760e5	7.27	22.2	0.1000	1.019762e-1
STD_0.5ppb	Standard	4.23e5	6.580e5	7.26	35.1	0.5000	5.716418e-1
STD_1ppb	Standard	7.22e5	6.418e5	7.26	56.3	1.0000	1.007501e0
STD_5ppb	Standard	4.66e6	7.441e5	7.27	80.6	5.0000	5.646525e0
STD_10ppb	Standard	8.08e6	7.645e5	7.26	80.1	10.0000	9.535606e0
STD_50ppb	Standard	4.33e7	8.212e5	7.26	107.2	50.0000	4.755190e1
STD_100ppb	Standard	7.30e7	8.032e5	7.27	103.6	100.0000	8.210568e1
STD_200ppb	Standard	N/A	9.369e5	N/A	N/A	200.0000	N/A
STD_500ppb	Standard	N/A	8.441e5	N/A	N/A	500.0000	N/A
V2.0_MW_RQC1_20240724	Quality Control	2.99e6	3.905e5	7.26	11.2	0.0000	6.910401e0
Blank	Unknown	N/A	5.961e3	N/A	N/A	N/A	N/A
V3.0_MWMS_20240725_1	Unknown	3.60e6	1.232e6	7.25	86.5	N/A	2.631772e0
MWXS243064a_R1	Quality Control	1.25e7	3.925e5	7.24	41.3	0.0000	2.878660e1
MWXS243064a_R2	Quality Control	1.14e7	3.390e5	7.25	37.1	0.0000	3.030599e1
MWXS243064a_R3	Quality Control	1.21e7	3.379e5	7.23	43.1	0.0000	3.234521e1
T24186682b_a	Unknown	2.06e7	3.024e5	7.25	25.4	N/A	6.143743e1
T24186682b_b	Unknown	1.88e7	2.893e5	7.25	25.9	N/A	5.851212e1
T24186682b_c	Unknown	1.83e7	2.806e5	7.25	25.2	N/A	5.894515e1
T24186683b_a	Unknown	1.23e7	2.778e5	7.24	72.1	N/A	4.004090e1
T24186683b_b	Unknown	1.24e7	3.603e5	7.24	54.3	N/A	3.113114e1
T24186683b_c	Unknown	1.43e7	3.184e5	7.24	55.2	N/A	4.056042e1
T24186684b_a	Unknown	2.07e7	3.496e5	7.25	35.6	N/A	5.350397e1
T24186684b_b	Unknown	1.21e7	3.022e5	7.25	37.1	N/A	3.624104e1
T24186684b_c	Unknown	1.48e7	2.908e5	7.25	43.6	N/A	4.595250e1

Compound name: OPDA
Regression Equation: y = 1.10738 x + 0.00926 (r = 0.99360) (weighting: 1 / x^2)

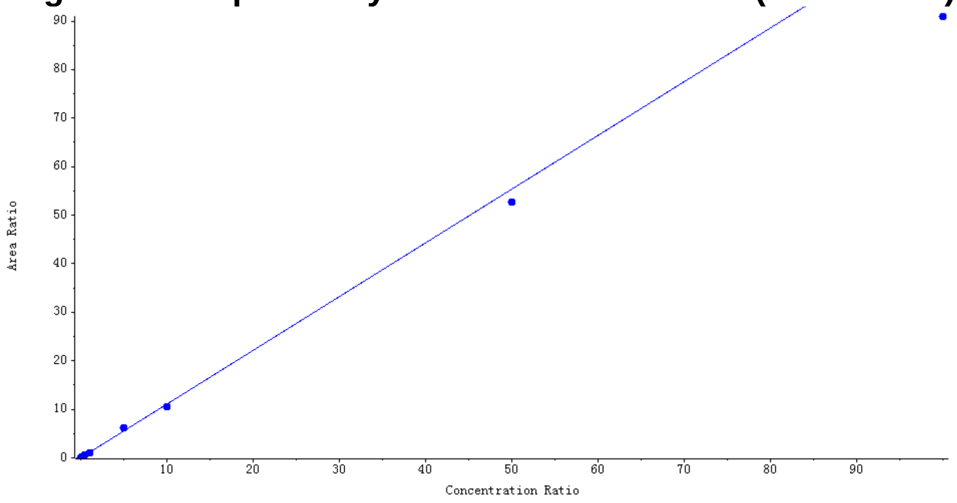

Peak Review

Blank
OPDA AREA:N/A S/N:N/A

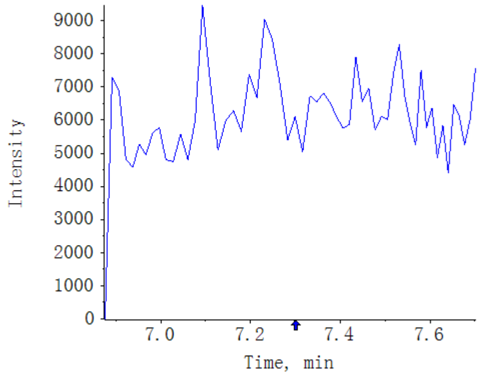

V3.0_MWMS_20240725_1
OPDA AREA:3.60e6 S/N:86.5

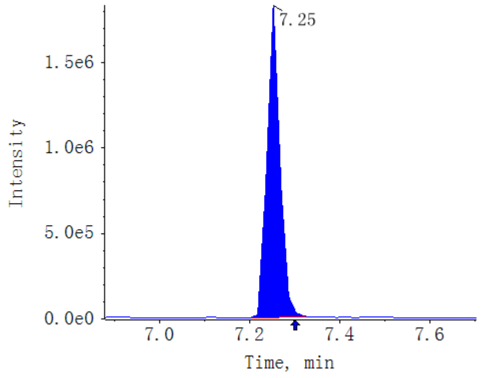

T24186682b_a
OPDA AREA:2.06e7 S/N:25.4

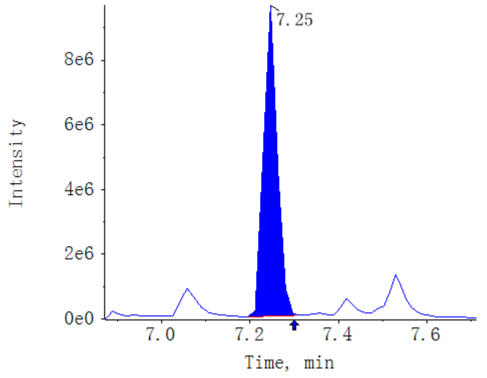

T24186682b_b
OPDA AREA:1.88e7 S/N:25.9

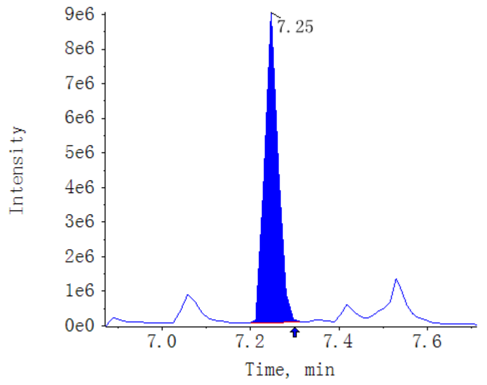

T24186682b_c
OPDA AREA:1.83e7 S/N:25.2

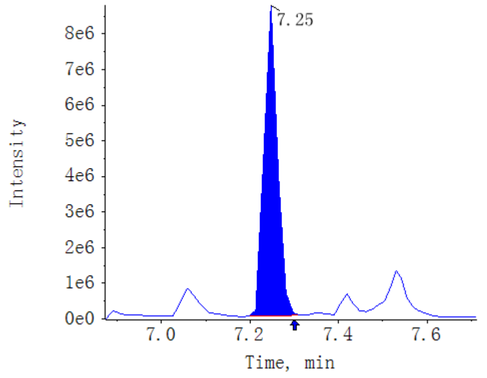

T24186683b_a
OPDA AREA:1.23e7 S/N:72.1

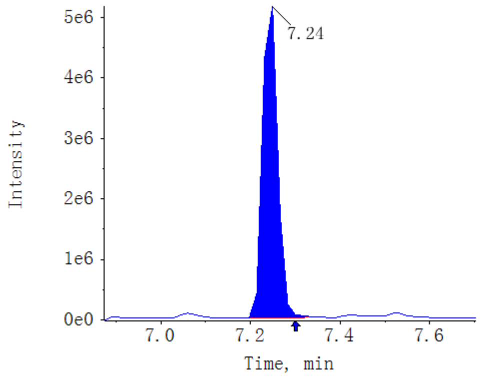

T24186683b_b
OPDA AREA:1.24e7 S/N:54.3

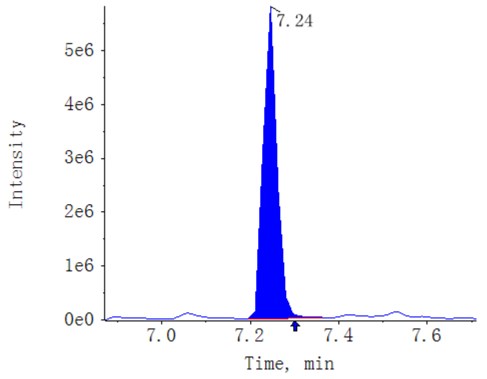

T24186683b_c
OPDA AREA:1.43e7 S/N:55.2

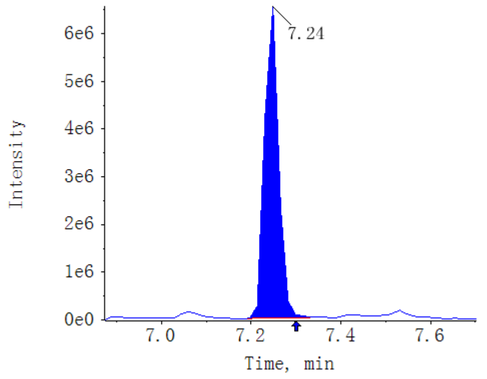

T24186684b_a
OPDA AREA:2.07e7 S/N:35.6

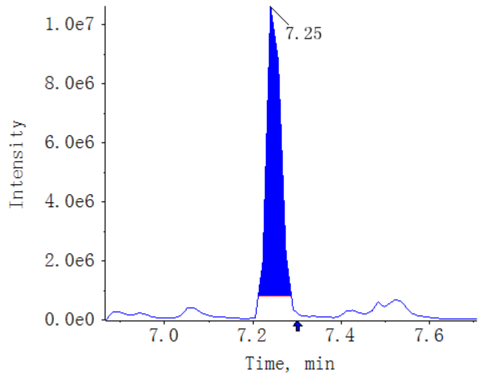

T24186684b_b
OPDA AREA:1.21e7 S/N:37.1

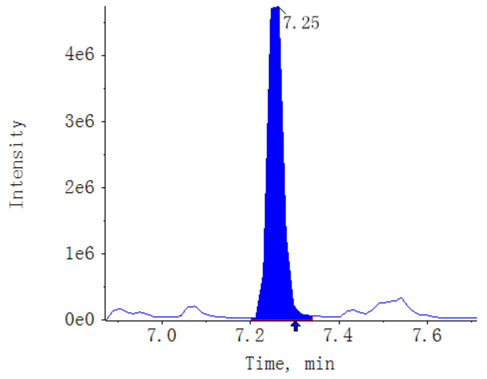

T24186684b_c
OPDA AREA:1.48e7 S/N:43.6

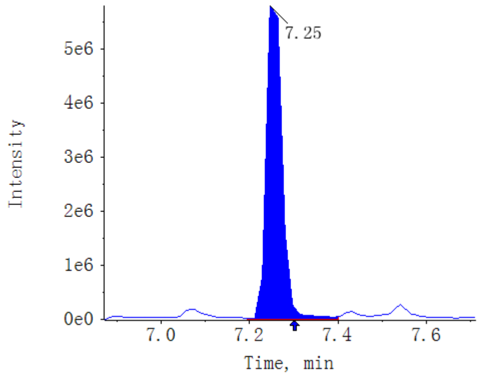

Result Table	MWXS-24-3064-a_9_WH6500-17_A20-3_V6.0_WSS_20240730	Algorithm Used	MQ4
Acquisition Method	ACC-PHs_V6.0_WH6500-17_CMY_20240521.dam	Instrument Name	Triple Quad 6500+ Low Mass
Project	N/A	Analytes QTY	109:67

Compound name: 12-OH-JA (209.2 / 191.2)

Sample Name	Sample Type	Area (cps)	Is Area (cps)	RT (min)	S/N	Target Conc	Calculated Conc.()
STD_0.01ppb	Standard	N/A	7.502e5	N/A	N/A	0.0100	N/A
STD_0.05ppb	Standard	N/A	7.505e5	N/A	N/A	0.0500	N/A
STD_0.1ppb	Standard	8.46e4	7.760e5	4.81	9.3	0.1000	9.892545e-2
STD_0.5ppb	Standard	8.78e4	6.580e5	4.80	9.2	0.5000	4.607752e-1
STD_1ppb	Standard	1.20e5	6.418e5	4.80	10.9	1.0000	1.249369e0
STD_5ppb	Standard	3.43e5	7.441e5	4.80	22.4	5.0000	5.326402e0
STD_10ppb	Standard	6.15e5	7.645e5	4.79	33.0	10.0000	1.044156e1
STD_50ppb	Standard	2.70e6	8.212e5	4.78	53.9	50.0000	4.734978e1
STD_100ppb	Standard	5.11e6	8.032e5	4.80	45.2	100.0000	9.312681e1
STD_200ppb	Standard	1.08e7	9.369e5	4.79	53.3	200.0000	1.704252e2
STD_500ppb	Standard	N/A	8.441e5	N/A	N/A	500.0000	N/A
V2.0_MW_RQC1_20240724	Quality Control	N/A	3.905e5	N/A	N/A	0.0000	N/A
Blank	Unknown	N/A	5.961e3	N/A	N/A	N/A	N/A
V3.0_MWMS_20240725_1	Unknown	4.30e5	1.232e6	4.78	18.0	N/A	3.668487e0
MWXS243064a_R1	Quality Control	N/A	3.925e5	N/A	N/A	0.0000	N/A
MWXS243064a_R2	Quality Control	N/A	3.390e5	N/A	N/A	0.0000	N/A
MWXS243064a_R3	Quality Control	N/A	3.379e5	N/A	N/A	0.0000	N/A
T24186682b_a	Unknown	N/A	3.024e5	N/A	N/A	N/A	N/A
T24186682b_b	Unknown	N/A	2.893e5	N/A	N/A	N/A	N/A
T24186682b_c	Unknown	N/A	2.806e5	N/A	N/A	N/A	N/A
T24186683b_a	Unknown	N/A	2.778e5	N/A	N/A	N/A	N/A
T24186683b_b	Unknown	N/A	3.603e5	N/A	N/A	N/A	N/A
T24186683b_c	Unknown	N/A	3.184e5	N/A	N/A	N/A	N/A
T24186684b_a	Unknown	N/A	3.496e5	N/A	N/A	N/A	N/A
T24186684b_b	Unknown	N/A	3.022e5	N/A	N/A	N/A	N/A
T24186684b_c	Unknown	N/A	2.908e5	N/A	N/A	N/A	N/A

Compound name: 12-OH-JA
Regression Equation: y = 0.06726 x + 0.10239 (r = 0.99185) (weighting: 1 / x^2)

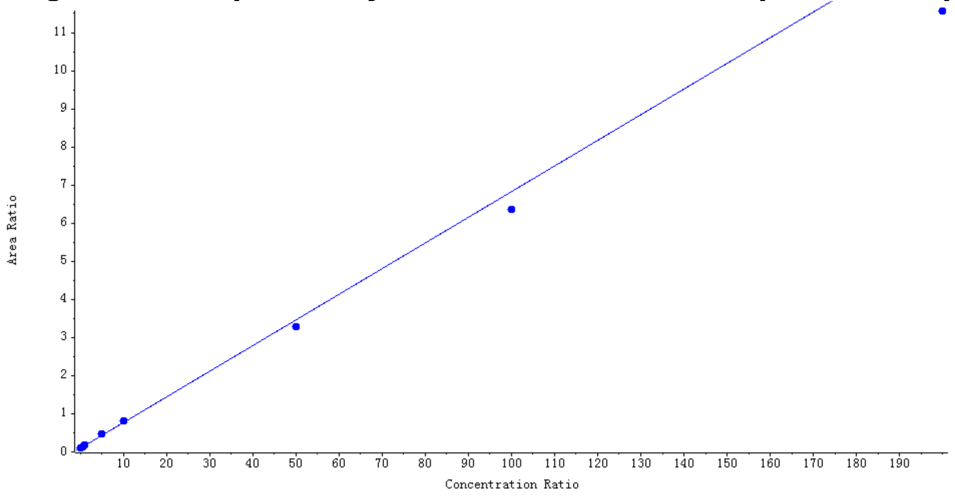

Peak Review

Blank
12-OH-JA AREA:N/A S/N:N/A

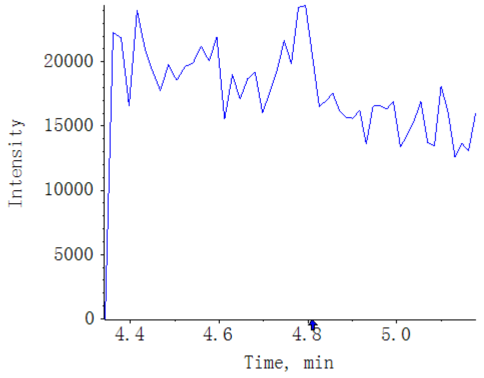

V3.0_MWMS_20240725_1
12-OH-JA AREA:4.30e5 S/N:18.0

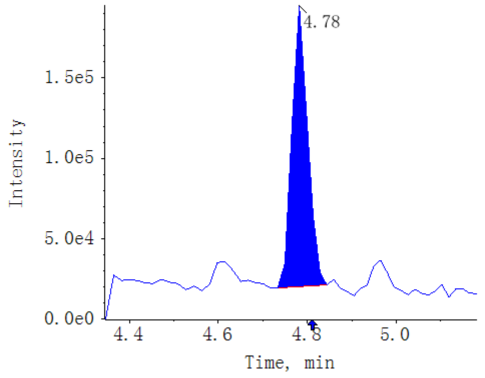

T24186682b_a
12-OH-JA AREA:N/A S/N:N/A

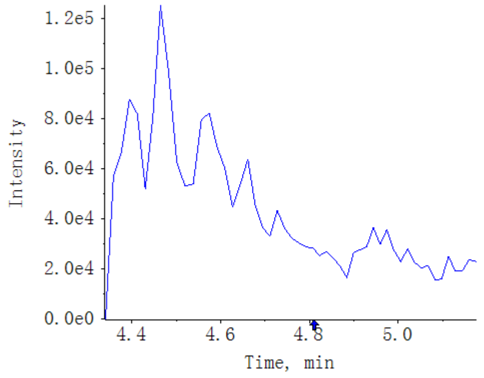

T24186682b_b
12-OH-JA AREA:N/A S/N:N/A

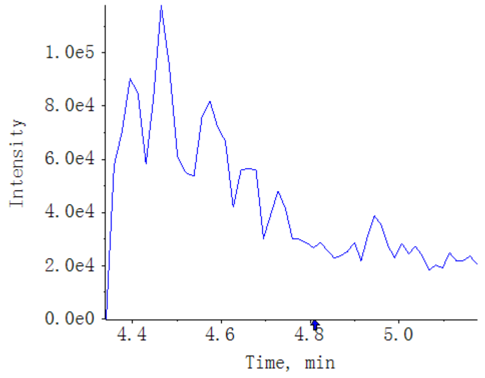

T24186682b_c
12-OH-JA AREA:N/A S/N:N/A

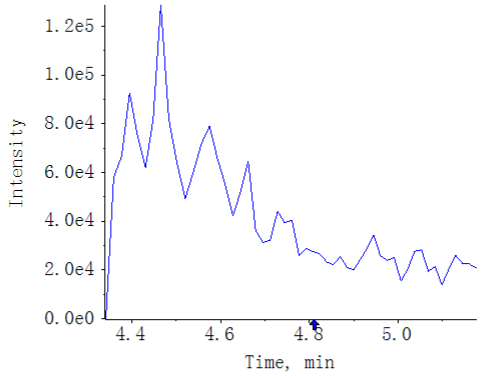

T24186683b_a
12-OH-JA AREA:N/A S/N:N/A

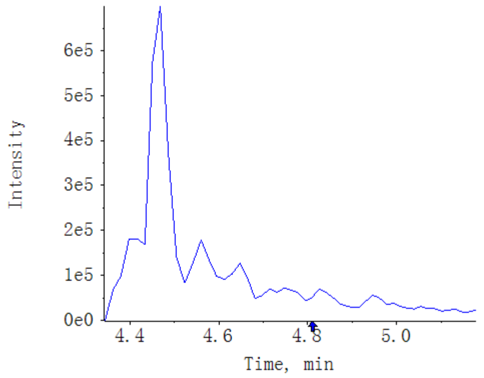

T24186683b_b
12-OH-JA AREA:N/A S/N:N/A

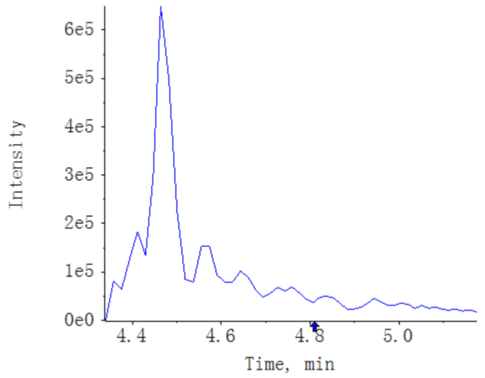

T24186683b_c
12-OH-JA AREA:N/A S/N:N/A

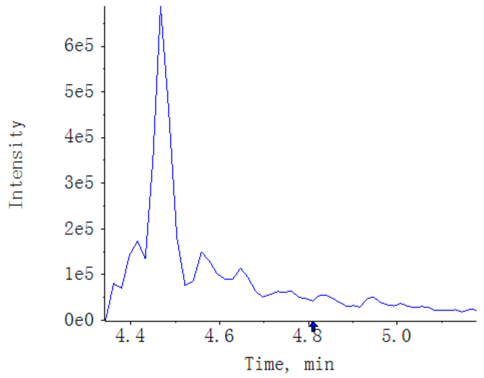

T24186684b_a
12-OH-JA AREA:N/A S/N:N/A

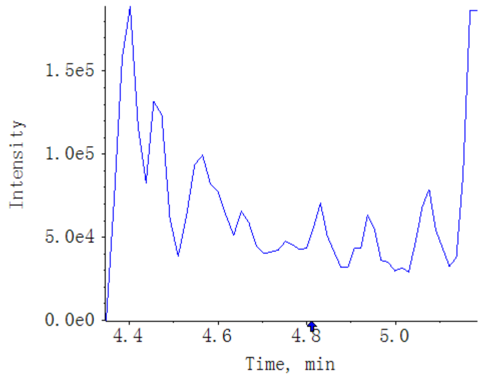

T24186684b_b
12-OH-JA AREA:N/A S/N:N/A

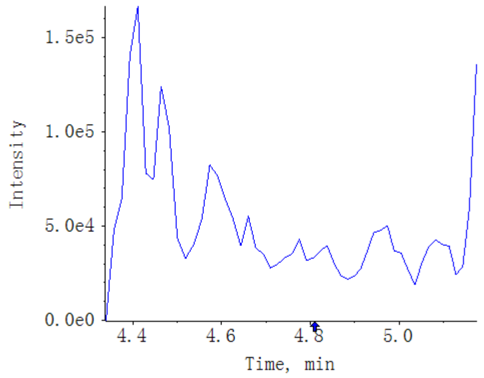

T24186684b_c
12-OH-JA AREA:N/A S/N:N/A

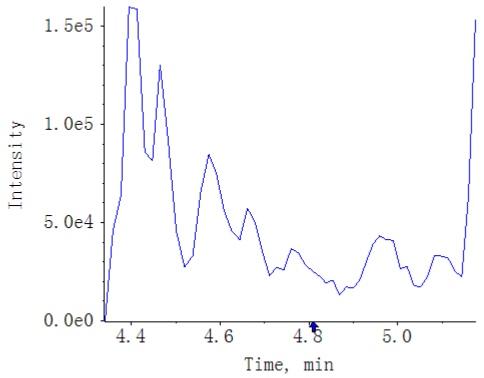

Result Table	MWXS-24-3064-a_9_WH6500-17_A20-3_V6.0_WSS_20240730	Algorithm Used	MQ4
Acquisition Method	ACC-PHs_V6.0_WH6500-17_CMY_20240521.dam	Instrument Name	Triple Quad 6500+ Low Mass
Project	N/A	Analytes QTY	109:68

Compound name: JA-ACC (294.1 / 151.1)

Sample Name	Sample Type	Area (cps)	Is Area (cps)	RT (min)	S/N	Target Conc	Calculated Conc.()
STD_0.01ppb	Standard	N/A	7.502e5	N/A	N/A	0.0100	N/A
STD_0.05ppb	Standard	7.49e3	7.505e5	5.36	15.7	0.0500	5.421944e-2
STD_0.1ppb	Standard	1.03e4	7.760e5	5.37	18.6	0.1000	7.991254e-2
STD_0.5ppb	Standard	5.26e4	6.580e5	5.36	38.2	0.5000	5.919634e-1
STD_1ppb	Standard	8.02e4	6.418e5	5.37	33.3	1.0000	9.384245e-1
STD_5ppb	Standard	5.37e5	7.441e5	5.38	35.2	5.0000	5.525494e0
STD_10ppb	Standard	9.33e5	7.645e5	5.37	57.2	10.0000	9.368647e0
STD_50ppb	Standard	5.42e6	8.212e5	5.36	36.5	50.0000	5.078408e1
STD_100ppb	Standard	1.04e7	8.032e5	5.37	59.8	100.0000	9.975050e1
STD_200ppb	Standard	2.29e7	9.369e5	5.37	40.3	200.0000	1.877969e2
STD_500ppb	Standard	N/A	8.441e5	N/A	N/A	500.0000	N/A
V2.0_MW_RQC1_20240724	Quality Control	1.48e4	3.905e5	5.42	7.2	0.0000	2.690411e-1
Blank	Unknown	N/A	5.961e3	N/A	N/A	N/A	N/A
V3.0_MWMS_20240725_1	Unknown	5.07e5	1.232e6	5.35	36.5	N/A	3.141595e0
MWXS243064a_R1	Quality Control	N/A	3.925e5	N/A	N/A	0.0000	N/A
MWXS243064a_R2	Quality Control	N/A	3.390e5	N/A	N/A	0.0000	N/A
MWXS243064a_R3	Quality Control	N/A	3.379e5	N/A	N/A	0.0000	N/A
T24186682b_a	Unknown	N/A	3.024e5	N/A	N/A	N/A	N/A
T24186682b_b	Unknown	N/A	2.893e5	N/A	N/A	N/A	N/A
T24186682b_c	Unknown	N/A	2.806e5	N/A	N/A	N/A	N/A
T24186683b_a	Unknown	N/A	2.778e5	N/A	N/A	N/A	N/A
T24186683b_b	Unknown	N/A	3.603e5	N/A	N/A	N/A	N/A
T24186683b_c	Unknown	N/A	3.184e5	N/A	N/A	N/A	N/A
T24186684b_a	Unknown	N/A	3.496e5	N/A	N/A	N/A	N/A
T24186684b_b	Unknown	N/A	3.022e5	N/A	N/A	N/A	N/A
T24186684b_c	Unknown	N/A	2.908e5	N/A	N/A	N/A	N/A

Compound name: JA-ACC
Regression Equation: y = 0.12997 x + 0.00294 (r = 0.99243) (weighting: 1 / x^2)

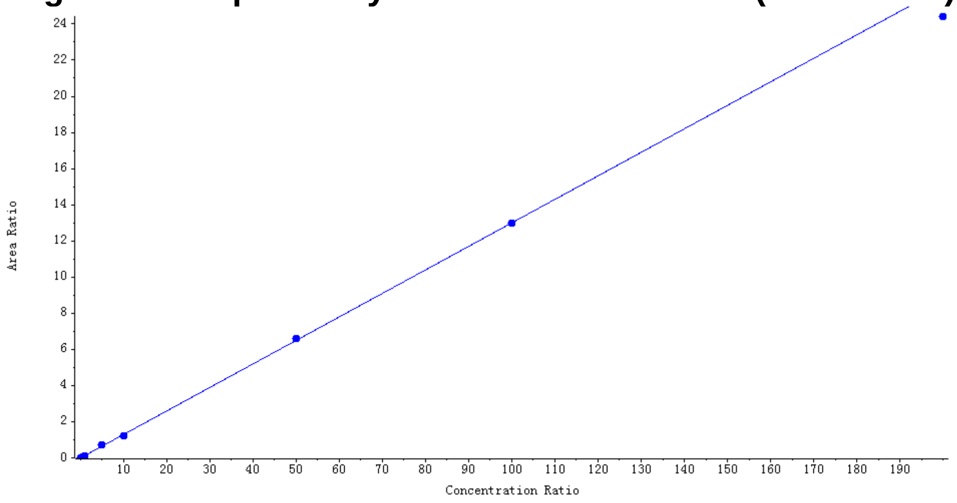

Peak Review

Blank
JA-ACC AREA:N/A S/N:N/A

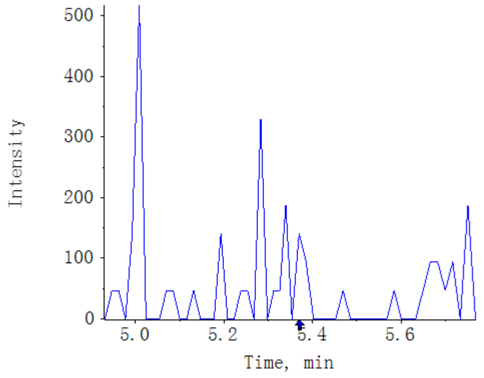

V3.0_MWMS_20240725_1
JA-ACC AREA:5.07e5 S/N:36.5

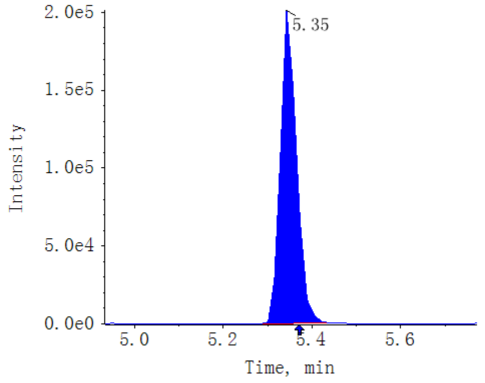

T24186682b_a
JA-ACC AREA:N/A S/N:N/A

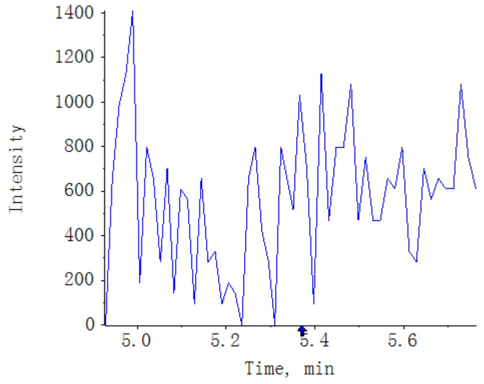

T24186682b_b
JA-ACC AREA:N/A S/N:N/A

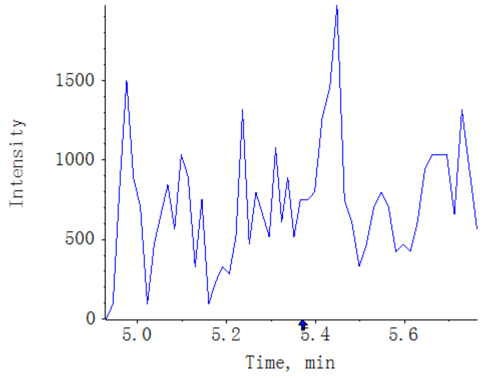

T24186682b_c
JA-ACC AREA:N/A S/N:N/A

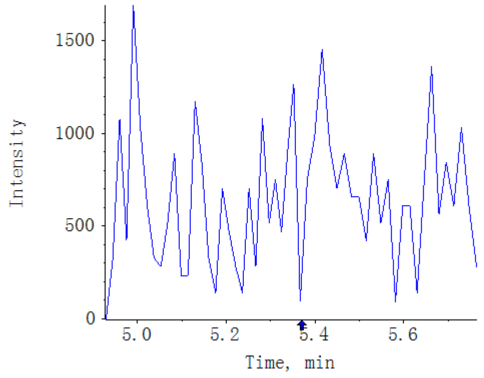

T24186683b_a
JA-ACC AREA:N/A S/N:N/A

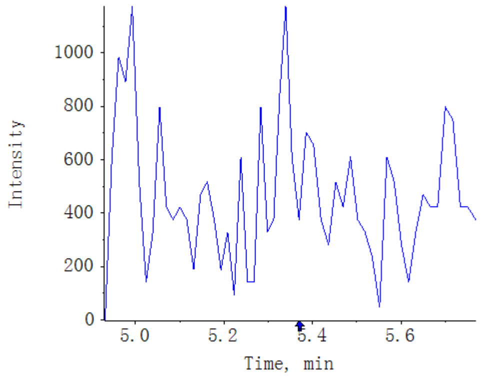

T24186683b_b
JA-ACC AREA:N/A S/N:N/A

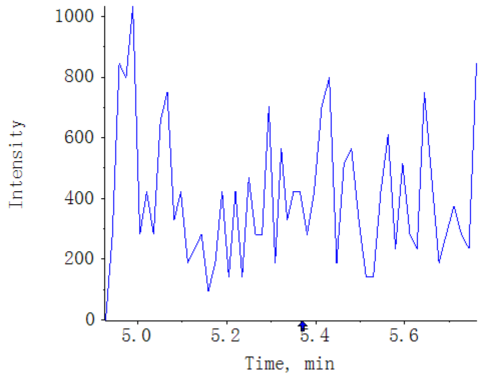

T24186683b_c
JA-ACC AREA:N/A S/N:N/A

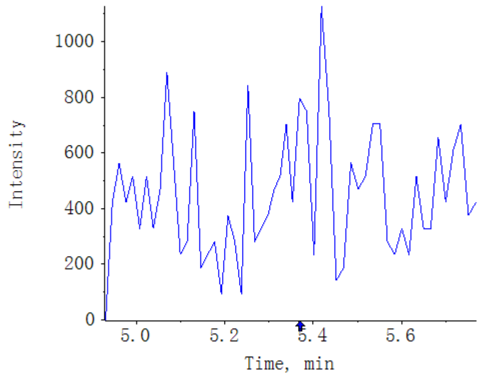

T24186684b_a
JA-ACC AREA:N/A S/N:N/A

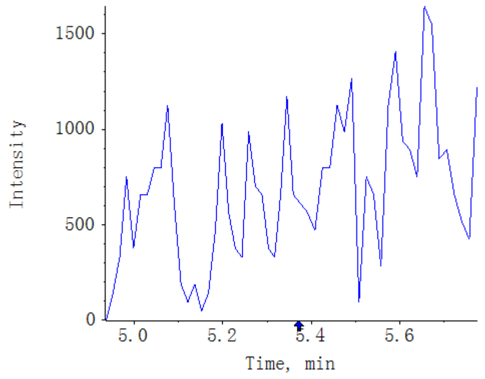

T24186684b_b
JA-ACC AREA:N/A S/N:N/A

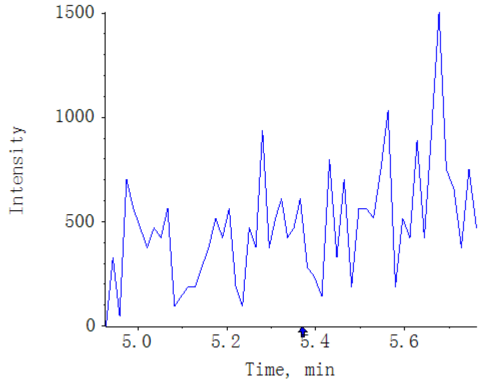

T24186684b_c
JA-ACC AREA:N/A S/N:N/A

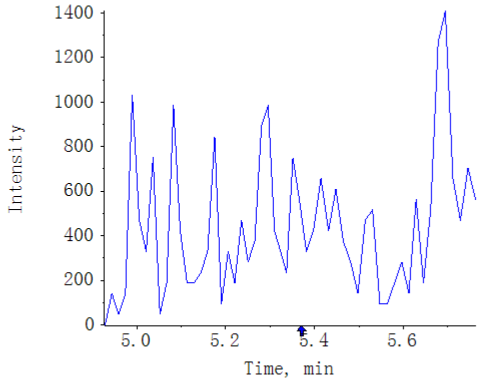

Result Table	MWXS-24-3064-a_9_WH6500-17_A20-3_V6.0_WSS_20240730	Algorithm Used	MQ4
Acquisition Method	ACC-PHs_V6.0_WH6500-17_CMY_20240521.dam	Instrument Name	Triple Quad 6500+ Low Mass
Project	N/A	Analytes QTY	109:69

Compound name: MLT (233.1 / 174.1)

Sample Name	Sample Type	Area (cps)	Is Area (cps)	RT (min)	S/N	Target Conc	Calculated Conc.()
STD_0.01ppb	Standard	1.43e4	N/A	4.91	8.0	0.0100	9.732919e-3
STD_0.05ppb	Standard	6.77e4	N/A	4.91	26.3	0.0500	5.370759e-2
STD_0.1ppb	Standard	1.35e5	N/A	4.92	50.2	0.1000	1.093283e-1
STD_0.5ppb	Standard	7.00e5	N/A	4.91	51.2	0.5000	5.734595e-1
STD_1ppb	Standard	1.17e6	N/A	4.91	48.2	1.0000	9.565710e-1
STD_5ppb	Standard	6.57e6	N/A	4.92	52.6	5.0000	5.400245e0
STD_10ppb	Standard	1.14e7	N/A	4.91	60.8	10.0000	9.376930e0
STD_50ppb	Standard	4.49e7	N/A	4.90	35.4	50.0000	3.690208e1
STD_100ppb	Standard	N/A	N/A	N/A	N/A	100.0000	N/A
STD_200ppb	Standard	N/A	N/A	N/A	N/A	200.0000	N/A
STD_500ppb	Standard	N/A	N/A	N/A	N/A	500.0000	N/A
V2.0_MW_RQC1_20240724	Quality Control	N/A	N/A	N/A	N/A	0.0000	N/A
Blank	Unknown	N/A	N/A	N/A	N/A	N/A	N/A
V3.0_MWMS_20240725_1	Unknown	7.59e6	N/A	4.89	40.7	N/A	6.237234e0
MWXS243064a_R1	Quality Control	N/A	N/A	N/A	N/A	0.0000	N/A
MWXS243064a_R2	Quality Control	N/A	N/A	N/A	N/A	0.0000	N/A
MWXS243064a_R3	Quality Control	N/A	N/A	N/A	N/A	0.0000	N/A
T24186682b_a	Unknown	N/A	N/A	N/A	N/A	N/A	N/A
T24186682b_b	Unknown	N/A	N/A	N/A	N/A	N/A	N/A
T24186682b_c	Unknown	N/A	N/A	N/A	N/A	N/A	N/A
T24186683b_a	Unknown	N/A	N/A	N/A	N/A	N/A	N/A
T24186683b_b	Unknown	N/A	N/A	N/A	N/A	N/A	N/A
T24186683b_c	Unknown	N/A	N/A	N/A	N/A	N/A	N/A
T24186684b_a	Unknown	N/A	N/A	N/A	N/A	N/A	N/A
T24186684b_b	Unknown	N/A	N/A	N/A	N/A	N/A	N/A
T24186684b_c	Unknown	N/A	N/A	N/A	N/A	N/A	N/A

Compound name: MLT
Regression Equation: y = 1.21590e6 x + 2416.61941 (r = 0.99083) (weighting: 1 / x^2)

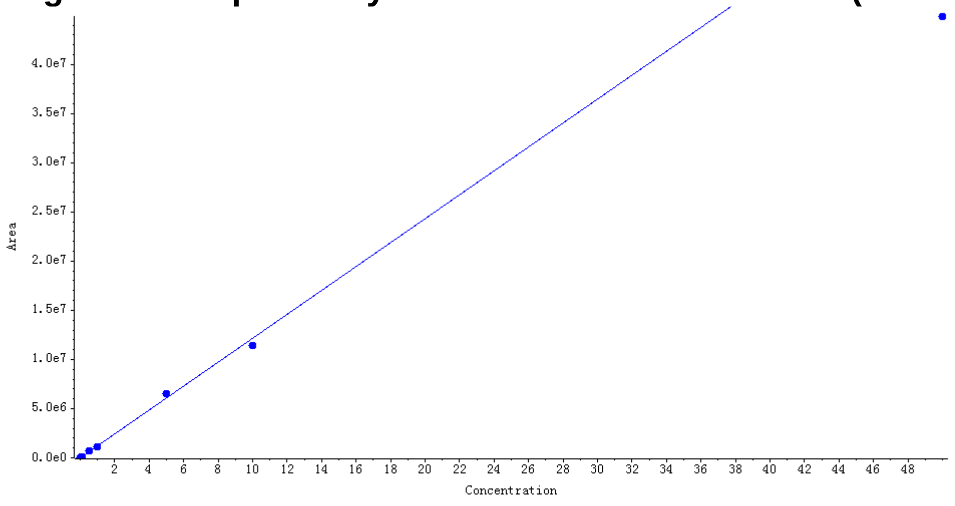

Peak Review

Blank

MLT AREA:N/A S/N:N/A

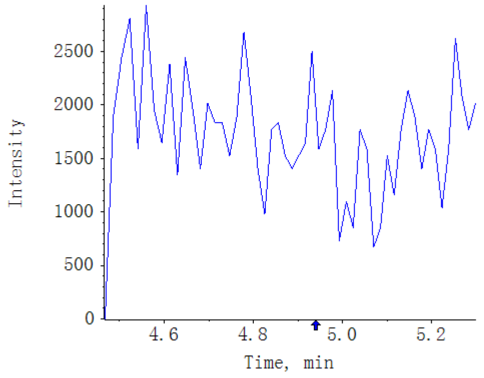

V3.0_MWMS_20240725_1

MLT AREA:7.59e6 S/N:40.7

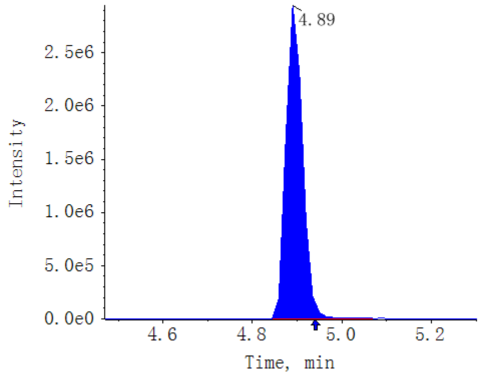

T24186682b_a

MLT AREA:N/A S/N:N/A

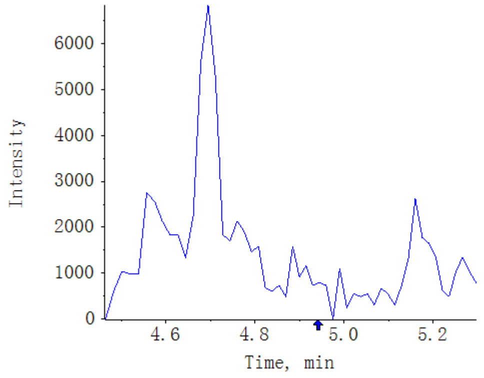

T24186682b_b

MLT AREA:N/A S/N:N/A

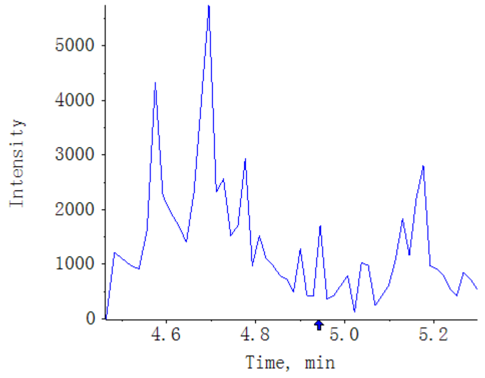

T24186682b_c

MLT AREA:N/A S/N:N/A

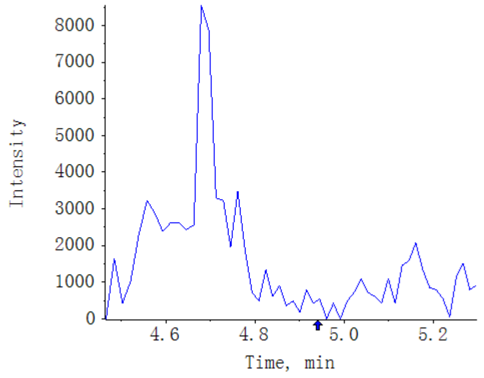

T24186683b_a

MLT AREA:N/A S/N:N/A

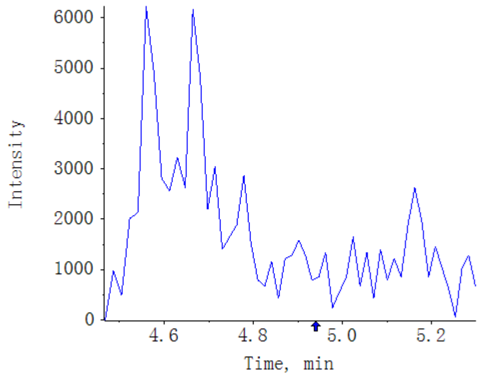

T24186683b_b

MLT AREA:N/A S/N:N/A

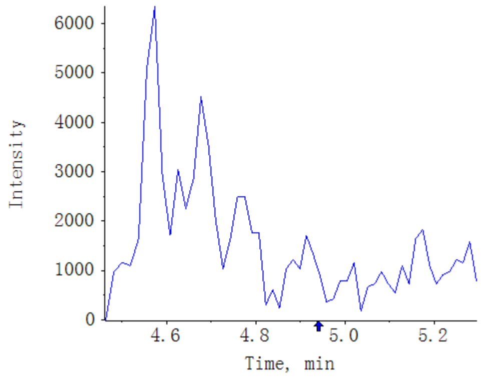

T24186683b_c

MLT AREA:N/A S/N:N/A

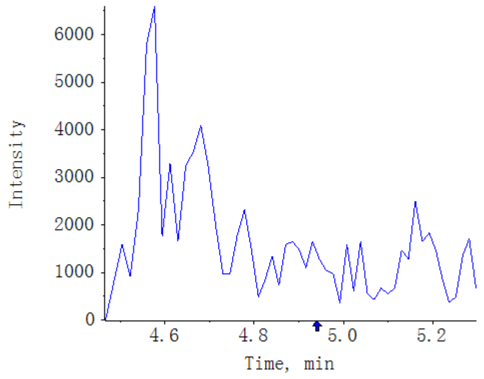

T24186684b_a

MLT AREA:N/A S/N:N/A

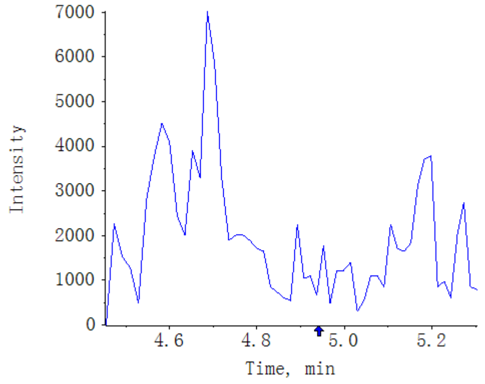

T24186684b_b

MLT AREA:N/A S/N:N/A

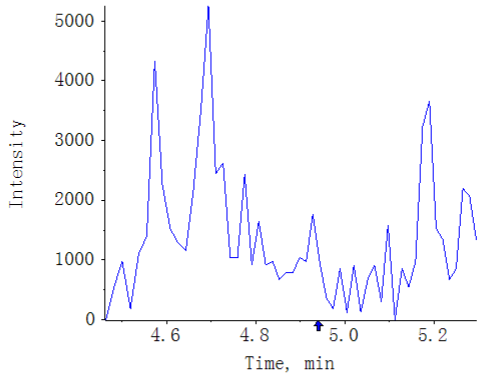

T24186684b_c

MLT AREA:N/A S/N:N/A

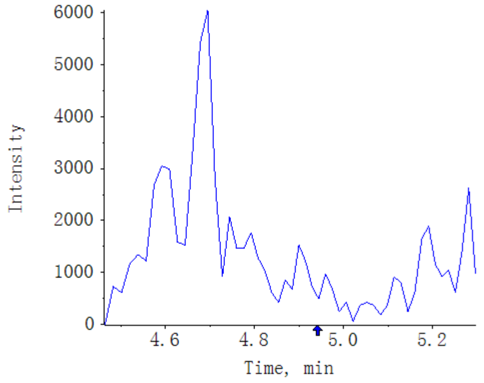

Result Table	MWXS-24-3064-a_9_WH6500-17_A20-3_V6.0_WSS_20240730	Algorithm Used	MQ4
Acquisition Method	ACC-PHs_V6.0_WH6500-17_CMY_20240521.dam	Instrument Name	Triple Quad 6500+ Low Mass
Project	N/A	Analytes QTY	109:70

Compound name: Phe_1 (166.1 / 149.1)

Sample Name	Sample Type	Area (cps)	Is Area (cps)	RT (min)	S/N	Target Conc	Calculated Conc.()
STD_0.01ppb	Standard	N/A	N/A	N/A	N/A	0.3000	N/A
STD_0.05ppb	Standard	N/A	N/A	N/A	N/A	1.5000	N/A
STD_0.1ppb	Standard	N/A	N/A	N/A	N/A	3.0000	N/A
STD_0.5ppb	Standard	9.87e3	N/A	1.80	10.0	15.0000	1.532516e1
STD_1ppb	Standard	1.71e4	N/A	1.79	15.6	30.0000	2.810019e1
STD_5ppb	Standard	9.49e4	N/A	1.80	30.4	150.0000	1.654811e2
STD_10ppb	Standard	1.68e5	N/A	1.80	35.8	300.0000	2.943717e2
STD_50ppb	Standard	9.40e5	N/A	1.79	54.2	1500.0000	1.657378e3
STD_100ppb	Standard	1.65e6	N/A	1.80	60.1	3000.0000	2.908203e3
STD_200ppb	Standard	3.00e6	N/A	1.79	66.6	6000.0000	5.297302e3
STD_500ppb	Standard	N/A	N/A	N/A	N/A	15000.0000	N/A
V2.0_MW_RQC1_20240724	Quality Control	1.36e6	N/A	1.91	151.6	0.0000	2.405695e3
Blank	Unknown	N/A	N/A	N/A	N/A	N/A	N/A
V3.0_MWMS_20240725_1	Unknown	1.65e5	N/A	1.91	78.5	N/A	2.890388e2
MWXS243064a_R1	Quality Control	2.49e6	N/A	1.88	401.5	0.0000	4.396085e3
MWXS243064a_R2	Quality Control	2.52e6	N/A	1.89	384.8	0.0000	4.455056e3
MWXS243064a_R3	Quality Control	2.61e6	N/A	1.88	379.5	0.0000	4.612795e3
T24186682b_a	Unknown	1.59e6	N/A	1.90	306.8	N/A	2.800852e3
T24186682b_b	Unknown	1.69e6	N/A	1.90	309.7	N/A	2.985844e3
T24186682b_c	Unknown	1.68e6	N/A	1.90	297.6	N/A	2.956409e3
T24186683b_a	Unknown	2.05e6	N/A	1.89	298.5	N/A	3.608802e3
T24186683b_b	Unknown	1.98e6	N/A	1.91	288.3	N/A	3.498495e3
T24186683b_c	Unknown	2.03e6	N/A	1.90	316.8	N/A	3.580758e3
T24186684b_a	Unknown	3.88e6	N/A	1.90	471.1	N/A	6.849428e3
T24186684b_b	Unknown	3.60e6	N/A	1.91	485.6	N/A	6.362075e3
T24186684b_c	Unknown	3.86e6	N/A	1.90	530.9	N/A	6.818239e3

Compound name: Phe_1
Regression Equation: y = 566.43857 x + 1186.99772 (r = 0.99574) (weighting: 1 / x^2)

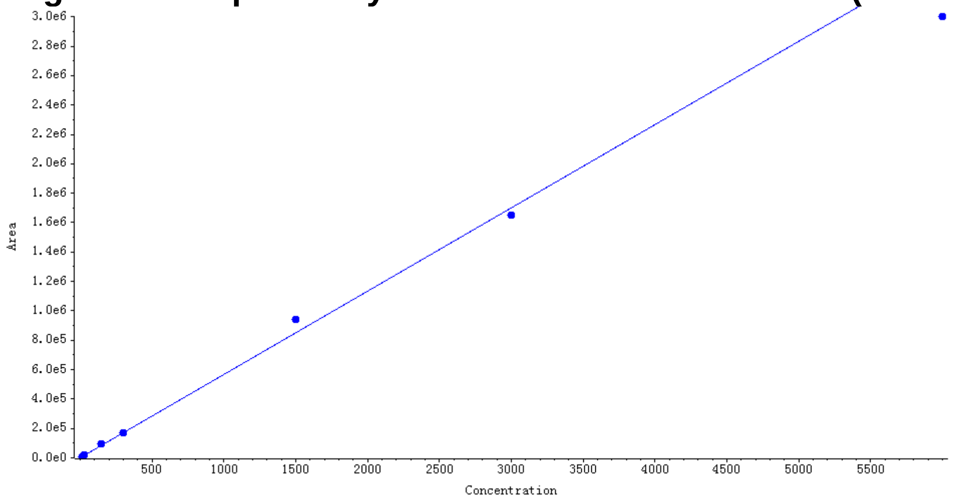

Peak Review

Blank

Phe_1 AREA:N/A S/N:N/A

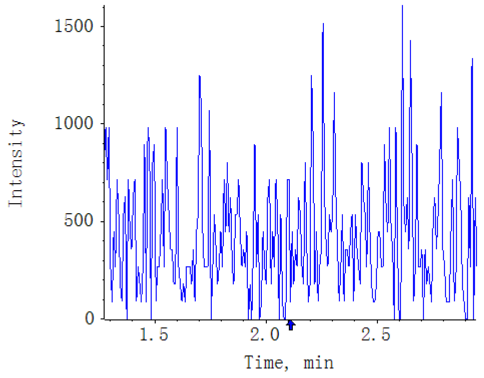

V3.0_MWMS_20240725_1

Phe_1 AREA:1.65e5 S/N:78.5

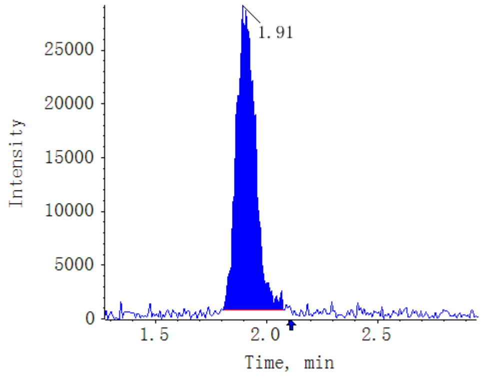

T24186682b_a

Phe_1 AREA:1.59e6 S/N:306.8

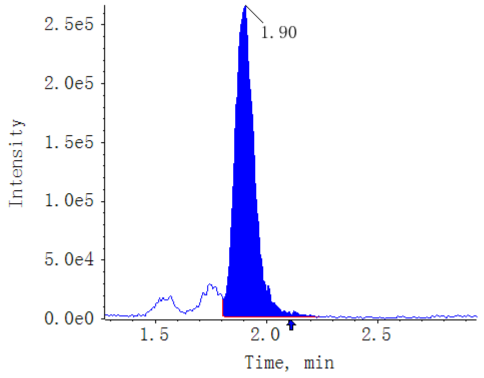

T24186682b_b

Phe_1 AREA:1.69e6 S/N:309.7

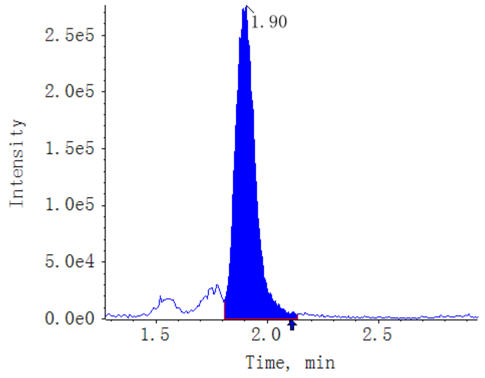

T24186682b_c

Phe_1 AREA:1.68e6 S/N:297.6

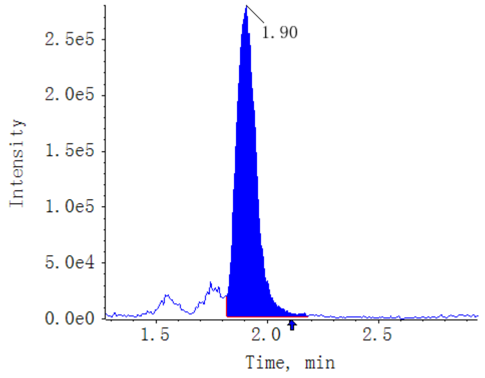

T24186683b_a

Phe_1 AREA:2.05e6 S/N:298.5

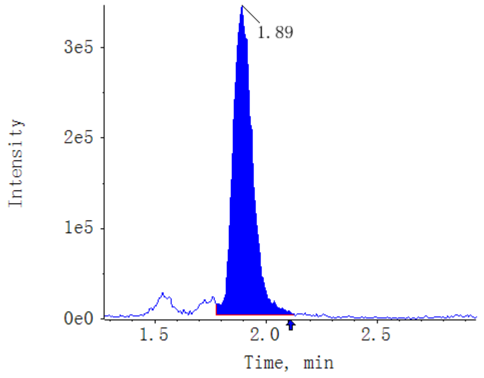

T24186683b_b

Phe_1 AREA:1.98e6 S/N:288.3

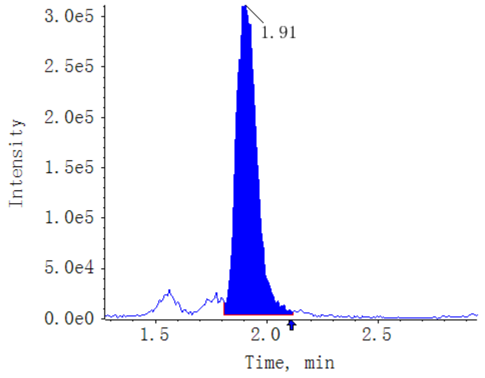

T24186683b_c

Phe_1 AREA:2.03e6 S/N:316.8

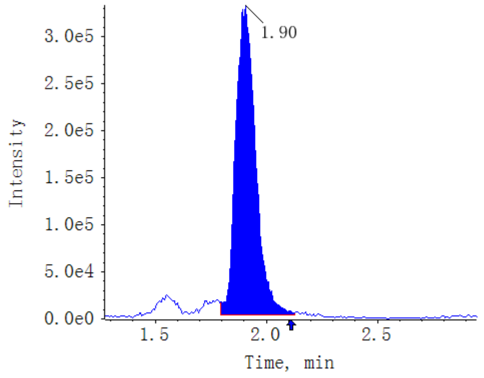

T24186684b_a

Phe_1 AREA:3.88e6 S/N:471.1

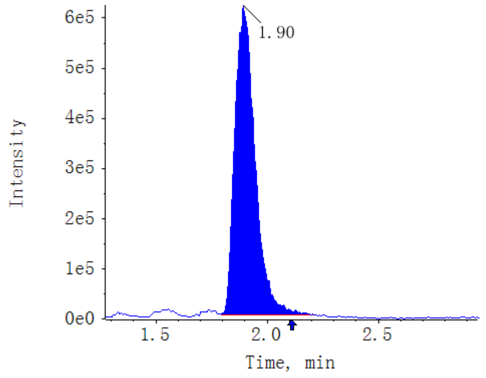

T24186684b_b

Phe_1 AREA:3.60e6 S/N:485.6

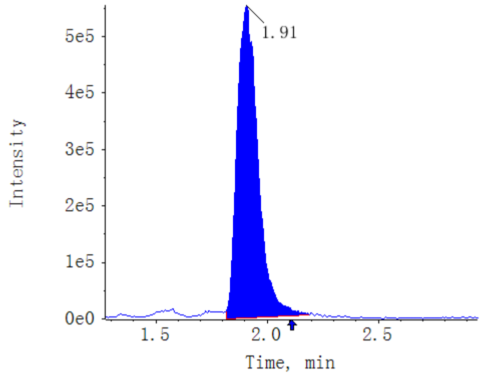

T24186684b_c

Phe_1 AREA:3.86e6 S/N:530.9

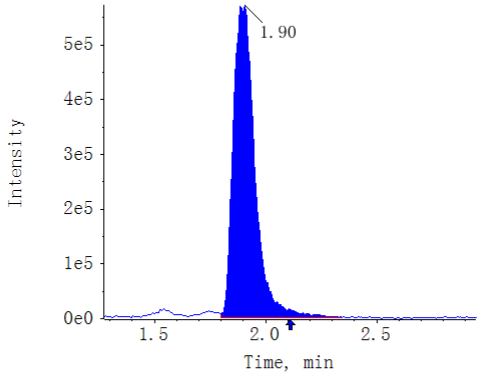

Result Table	MWXS-24-3064-a_9_WH6500-17_A20-3_V6.0_WSS_20240730	Algorithm Used	MQ4
Acquisition Method	ACC-PHs_V6.0_WH6500-17_CMY_20240521.dam	Instrument Name	Triple Quad 6500+ Low Mass
Project	N/A	Analytes QTY	109:71

Compound name: MeSAG (337.1 / 337.1)

Sample Name	Sample Type	Area (cps)	Is Area (cps)	RT (min)	S/N	Target Conc	Calculated Conc.()
STD_0.01ppb	Standard	N/A	N/A	N/A	N/A	0.0100	N/A
STD_0.05ppb	Standard	N/A	N/A	N/A	N/A	0.0500	N/A
STD_0.1ppb	Standard	N/A	N/A	N/A	N/A	0.1000	N/A
STD_0.5ppb	Standard	N/A	N/A	N/A	N/A	0.5000	N/A
STD_1ppb	Standard	2.27e4	N/A	4.17	3.7	1.0000	9.781323e-1
STD_5ppb	Standard	3.68e4	N/A	4.17	5.9	5.0000	5.221067e0
STD_10ppb	Standard	5.69e4	N/A	4.15	9.6	10.0000	1.128462e1
STD_50ppb	Standard	1.99e5	N/A	4.15	8.5	50.0000	5.419257e1
STD_100ppb	Standard	3.21e5	N/A	4.16	13.1	100.0000	9.060871e1
STD_200ppb	Standard	6.23e5	N/A	4.15	11.8	200.0000	1.814926e2
STD_500ppb	Standard	1.60e6	N/A	4.15	10.9	500.0000	4.758954e2
V2.0_MW_RQC1_20240724	Quality Control	N/A	N/A	N/A	N/A	0.0000	N/A
Blank	Unknown	N/A	N/A	N/A	N/A	N/A	N/A
V3.0_MWMS_20240725_1	Unknown	1.98e5	N/A	4.15	10.8	N/A	5.388002e1
MWXS243064a_R1	Quality Control	N/A	N/A	N/A	N/A	0.0000	N/A
MWXS243064a_R2	Quality Control	N/A	N/A	N/A	N/A	0.0000	N/A
MWXS243064a_R3	Quality Control	N/A	N/A	N/A	N/A	0.0000	N/A
T24186682b_a	Unknown	N/A	N/A	N/A	N/A	N/A	N/A
T24186682b_b	Unknown	N/A	N/A	N/A	N/A	N/A	N/A
T24186682b_c	Unknown	N/A	N/A	N/A	N/A	N/A	N/A
T24186683b_a	Unknown	N/A	N/A	N/A	N/A	N/A	N/A
T24186683b_b	Unknown	N/A	N/A	N/A	N/A	N/A	N/A
T24186683b_c	Unknown	N/A	N/A	N/A	N/A	N/A	N/A
T24186684b_a	Unknown	N/A	N/A	N/A	N/A	N/A	N/A
T24186684b_b	Unknown	N/A	N/A	N/A	N/A	N/A	N/A
T24186684b_c	Unknown	N/A	N/A	N/A	N/A	N/A	N/A

Compound name: MeSAG
Regression Equation: y = 3323.08561 x + 19406.02257 (r = 0.99572) (weighting: 1 / x^2)

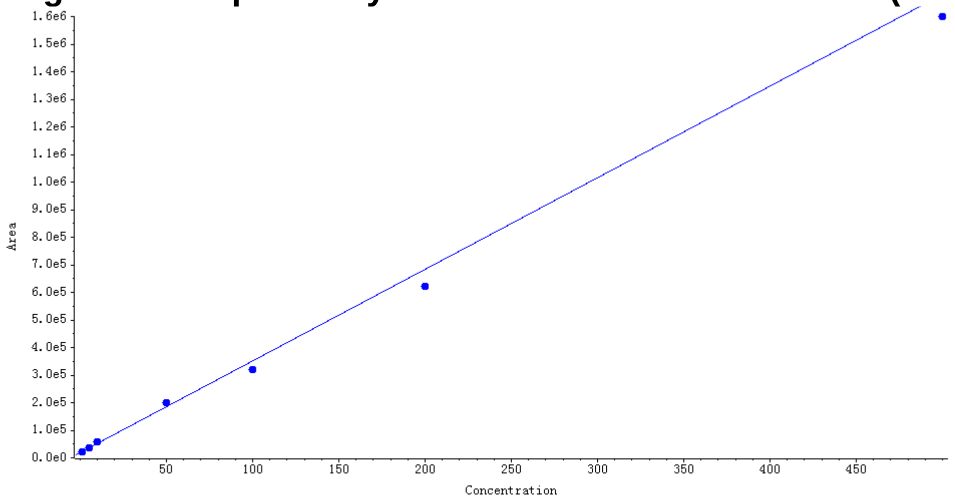

Peak Review

Blank
MeSAG AREA:N/A S/N:N/A

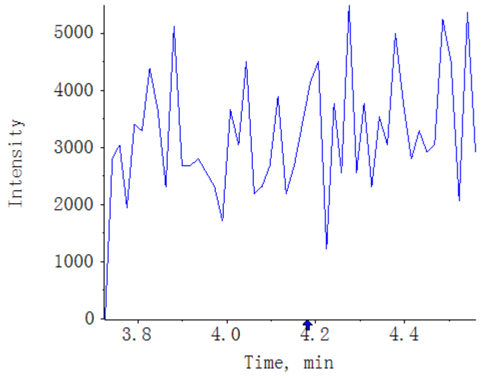

V3.0_MWMS_20240725_1
MeSAG AREA:1.98e5 S/N:10.8

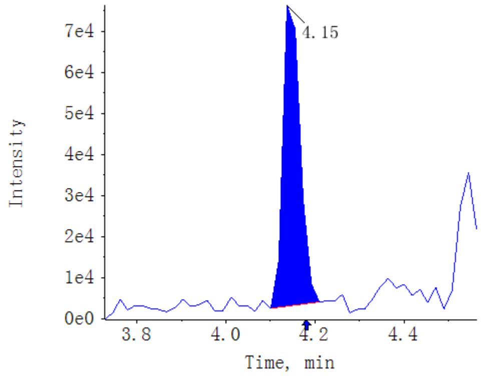

T24186682b_a
MeSAG AREA:N/A S/N:N/A

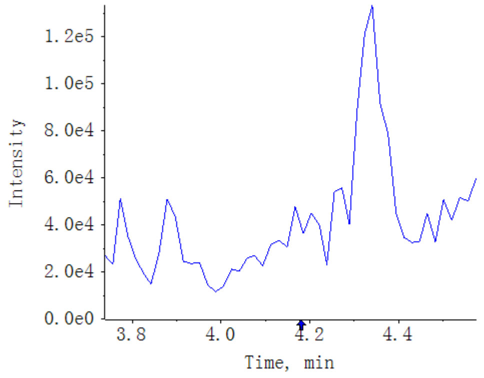

T24186682b_b
MeSAG AREA:N/A S/N:N/A

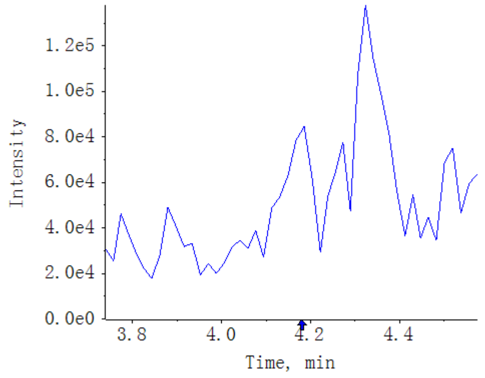

T24186682b_c
MeSAG AREA:N/A S/N:N/A

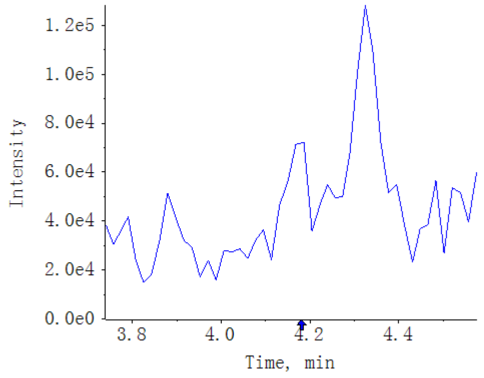

T24186683b_a
MeSAG AREA:N/A S/N:N/A

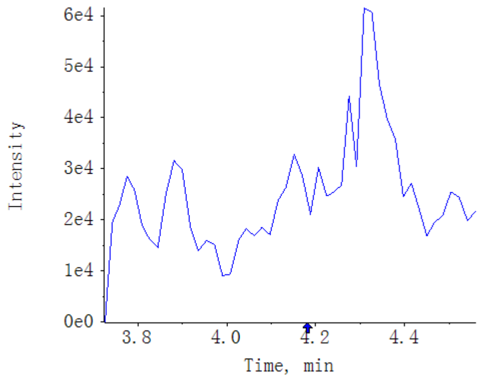

T24186683b_b
MeSAG AREA:N/A S/N:N/A

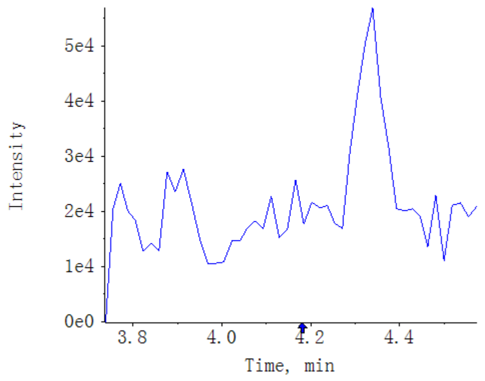

T24186683b_c
MeSAG AREA:N/A S/N:N/A

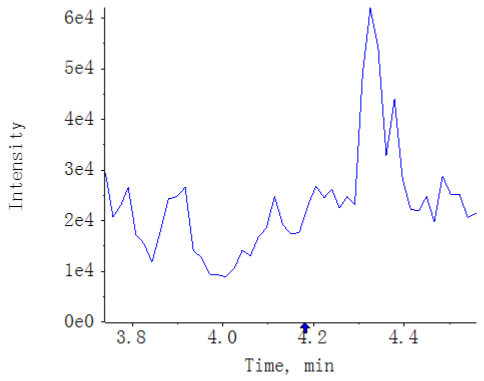

T24186684b_a
MeSAG AREA:N/A S/N:N/A

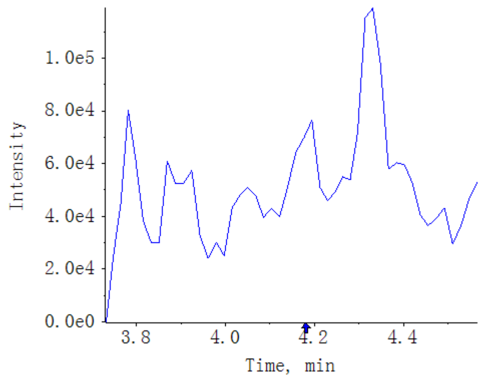

T24186684b_b
MeSAG AREA:N/A S/N:N/A

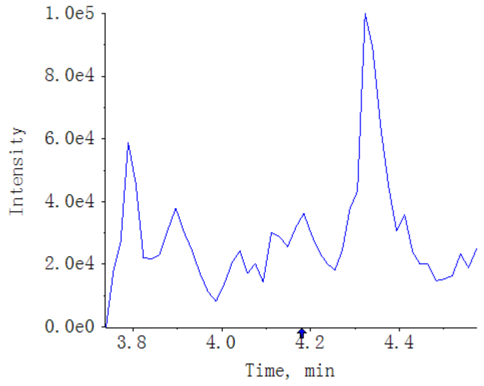

T24186684b_c
MeSAG AREA:N/A S/N:N/A

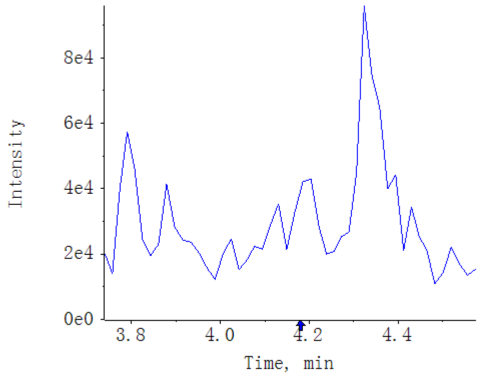

Result Table	MWXS-24-3064-a_9_WH6500-17_A20-3_V6.0_WSS_20240730	Algorithm Used	MQ4
Acquisition Method	ACC-PHs_V6.0_WH6500-17_CMY_20240521.dam	Instrument Name	Triple Quad 6500+ Low Mass
Project	N/A	Analytes QTY	109:72

Compound name: 5DS_1 (331.1 / 217.2)

Sample Name	Sample Type	Area (cps)	Is Area (cps)	RT (min)	S/N	Target Conc	Calculated Conc.()
STD_0.01ppb	Standard	N/A	N/A	N/A	N/A	0.2000	N/A
STD_0.05ppb	Standard	N/A	N/A	N/A	N/A	1.0000	N/A
STD_0.1ppb	Standard	2.35e4	N/A	7.50	55.8	2.0000	1.580810e0
STD_0.5ppb	Standard	7.96e4	N/A	7.49	91.7	10.0000	9.319834e0
STD_1ppb	Standard	1.19e5	N/A	7.49	67.6	20.0000	1.479013e1
STD_5ppb	Standard	1.09e6	N/A	7.50	74.5	100.0000	1.481195e2
STD_10ppb	Standard	1.57e6	N/A	7.49	72.7	200.0000	2.147361e2
STD_50ppb	Standard	7.44e6	N/A	7.49	93.5	1000.0000	1.023006e3
STD_100ppb	Standard	1.40e7	N/A	7.50	100.9	2000.0000	1.920447e3
STD_200ppb	Standard	N/A	N/A	N/A	N/A	4000.0000	N/A
STD_500ppb	Standard	N/A	N/A	N/A	N/A	10000.0000	N/A
V2.0_MW_RQC1_20240724	Quality Control	N/A	N/A	N/A	N/A	0.0000	N/A
Blank	Unknown	N/A	N/A	N/A	N/A	N/A	N/A
V3.0_MWMS_20240725_1	Unknown	8.83e5	N/A	7.48	61.8	N/A	1.199800e2
MWXS243064a_R1	Quality Control	N/A	N/A	N/A	N/A	0.0000	N/A
MWXS243064a_R2	Quality Control	N/A	N/A	N/A	N/A	0.0000	N/A
MWXS243064a_R3	Quality Control	N/A	N/A	N/A	N/A	0.0000	N/A
T24186682b_a	Unknown	N/A	N/A	N/A	N/A	N/A	N/A
T24186682b_b	Unknown	N/A	N/A	N/A	N/A	N/A	N/A
T24186682b_c	Unknown	N/A	N/A	N/A	N/A	N/A	N/A
T24186683b_a	Unknown	N/A	N/A	N/A	N/A	N/A	N/A
T24186683b_b	Unknown	N/A	N/A	N/A	N/A	N/A	N/A
T24186683b_c	Unknown	N/A	N/A	N/A	N/A	N/A	N/A
T24186684b_a	Unknown	N/A	N/A	N/A	N/A	N/A	N/A
T24186684b_b	Unknown	N/A	N/A	N/A	N/A	N/A	N/A
T24186684b_c	Unknown	N/A	N/A	N/A	N/A	N/A	N/A

Compound name: 5DS_1
Regression Equation: y = 7258.96213 x + 11982.69667 (r = 0.99552) (weighting: 1 / x)

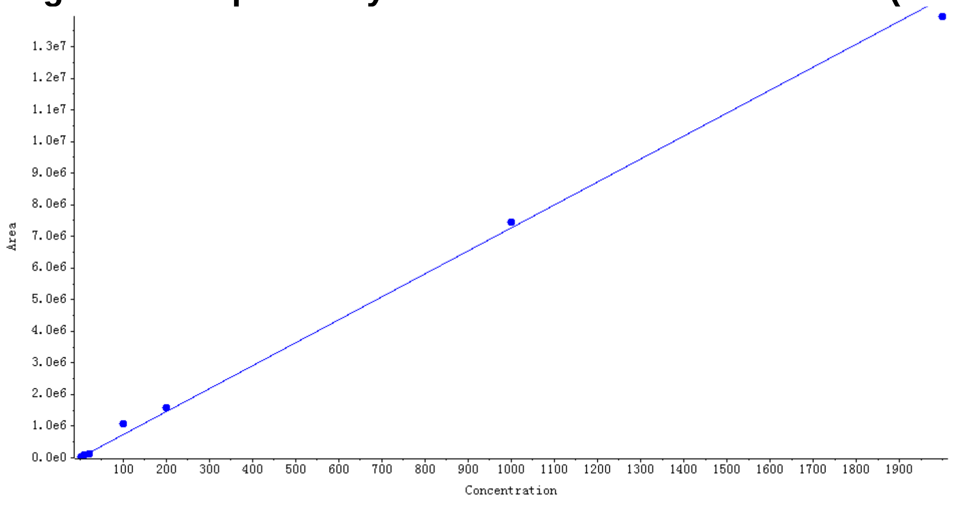

Peak Review

Blank
5DS_1 AREA:N/A S/N:N/A

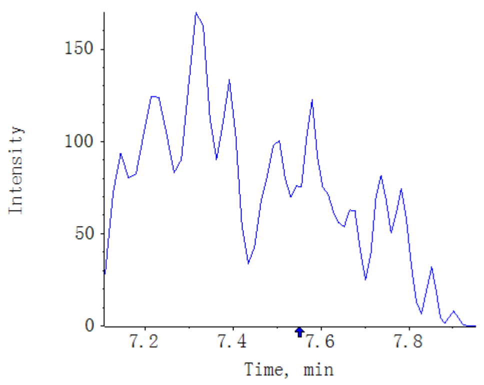

V3.0_MWMS_20240725_1
5DS_1 AREA:8.83e5 S/N:61.8

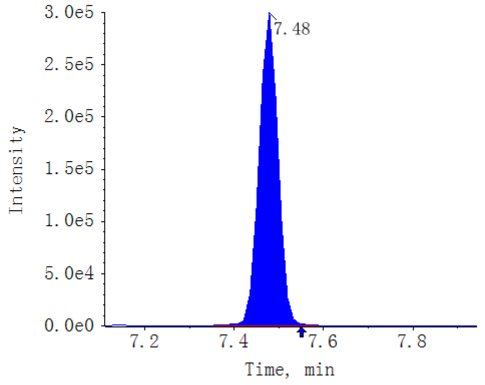

T24186682b_a
5DS_1 AREA:N/A S/N:N/A

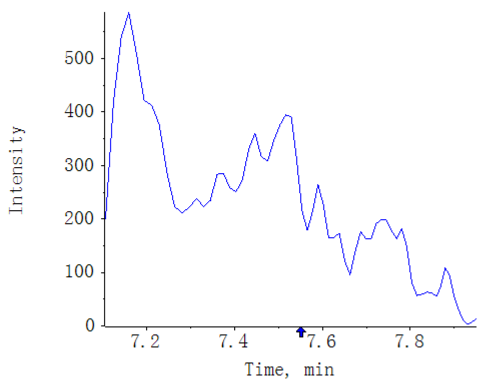

T24186682b_b
5DS_1 AREA:N/A S/N:N/A

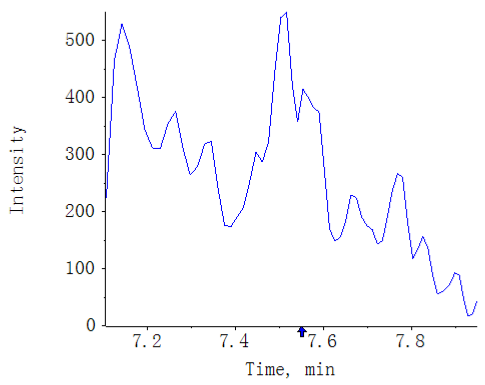

T24186682b_c
5DS_1 AREA:N/A S/N:N/A

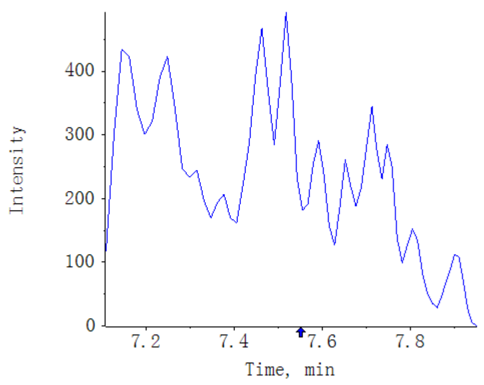

T24186683b_a
5DS_1 AREA:N/A S/N:N/A

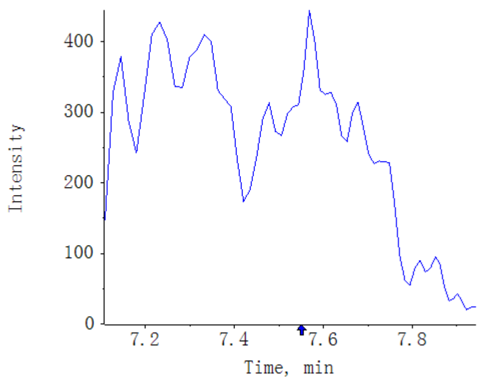

T24186683b_b
5DS_1 AREA:N/A S/N:N/A

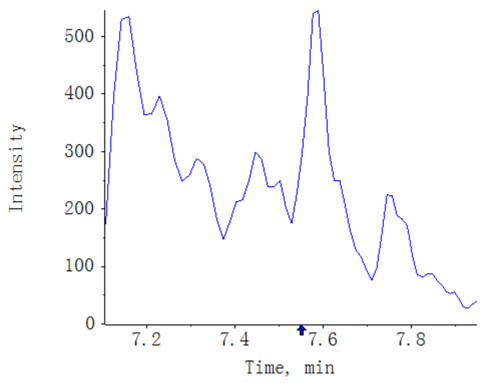

T24186683b_c
5DS_1 AREA:N/A S/N:N/A

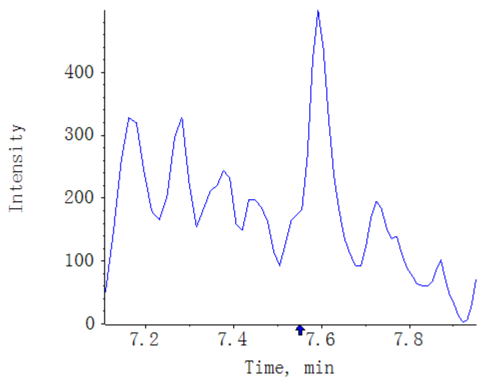

T24186684b_a
5DS_1 AREA:N/A S/N:N/A

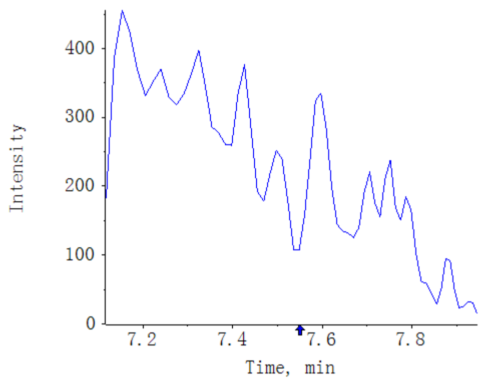

T24186684b_b
5DS_1 AREA:N/A S/N:N/A

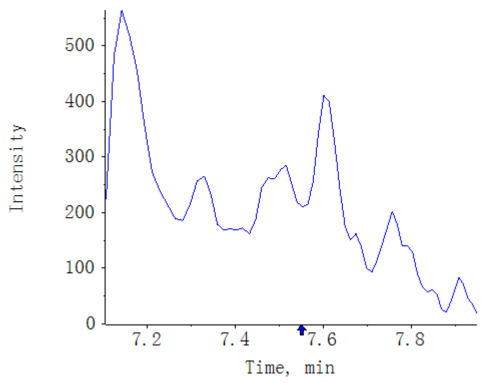

T24186684b_c
5DS_1 AREA:N/A S/N:N/A

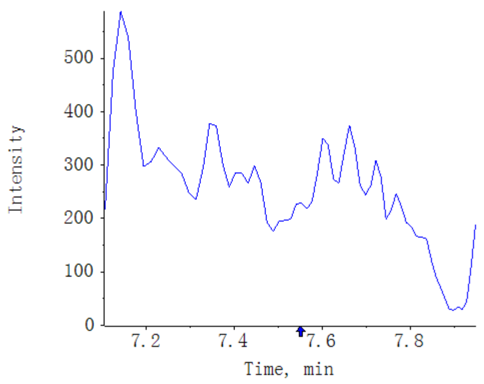

Result Table	MWXS-24-3064-a_9_WH6500-17_A20-3_V6.0_WSS_20240730	Algorithm Used	MQ4
Acquisition Method	ACC-PHs_V6.0_WH6500-17_CMY_20240521.dam	Instrument Name	Triple Quad 6500+ Low Mass
Project	N/A	Analytes QTY	109:73

Compound name: ST (347.4 / 96.9)

Sample Name	Sample Type	Area (cps)	Is Area (cps)	RT (min)	S/N	Target Conc	Calculated Conc.()
STD_0.01ppb	Standard	N/A	N/A	N/A	N/A	0.2000	N/A
STD_0.05ppb	Standard	N/A	N/A	N/A	N/A	1.0000	N/A
STD_0.1ppb	Standard	N/A	N/A	N/A	N/A	2.0000	N/A
STD_0.5ppb	Standard	6.00e3	N/A	6.39	3.6	10.0000	6.597100e0
STD_1ppb	Standard	1.15e4	N/A	6.40	5.5	20.0000	1.390959e1
STD_5ppb	Standard	9.16e4	N/A	6.41	5.9	100.0000	1.204520e2
STD_10ppb	Standard	1.64e5	N/A	6.39	6.5	200.0000	2.171215e2
STD_50ppb	Standard	9.04e5	N/A	6.39	7.9	1000.0000	1.199818e3
STD_100ppb	Standard	1.71e6	N/A	6.40	5.9	2000.0000	2.277835e3
STD_200ppb	Standard	3.34e6	N/A	6.40	7.0	4000.0000	4.443467e3
STD_500ppb	Standard	6.81e6	N/A	6.40	5.0	10000.0000	9.050799e3
V2.0_MW_RQC1_20240724	Quality Control	N/A	N/A	N/A	N/A	0.0000	N/A
Blank	Unknown	N/A	N/A	N/A	N/A	N/A	N/A
V3.0_MWMS_20240725_1	Unknown	8.21e4	N/A	6.37	8.2	N/A	1.078113e2
MWXS243064a_R1	Quality Control	N/A	N/A	N/A	N/A	0.0000	N/A
MWXS243064a_R2	Quality Control	N/A	N/A	N/A	N/A	0.0000	N/A
MWXS243064a_R3	Quality Control	N/A	N/A	N/A	N/A	0.0000	N/A
T24186682b_a	Unknown	N/A	N/A	N/A	N/A	N/A	N/A
T24186682b_b	Unknown	N/A	N/A	N/A	N/A	N/A	N/A
T24186682b_c	Unknown	N/A	N/A	N/A	N/A	N/A	N/A
T24186683b_a	Unknown	N/A	N/A	N/A	N/A	N/A	N/A
T24186683b_b	Unknown	N/A	N/A	N/A	N/A	N/A	N/A
T24186683b_c	Unknown	N/A	N/A	N/A	N/A	N/A	N/A
T24186684b_a	Unknown	N/A	N/A	N/A	N/A	N/A	N/A
T24186684b_b	Unknown	N/A	N/A	N/A	N/A	N/A	N/A
T24186684b_c	Unknown	N/A	N/A	N/A	N/A	N/A	N/A

Compound name: ST
Regression Equation: y = 752.22847 x + 1033.58992 (r = 0.99338) (weighting: 1 / x)

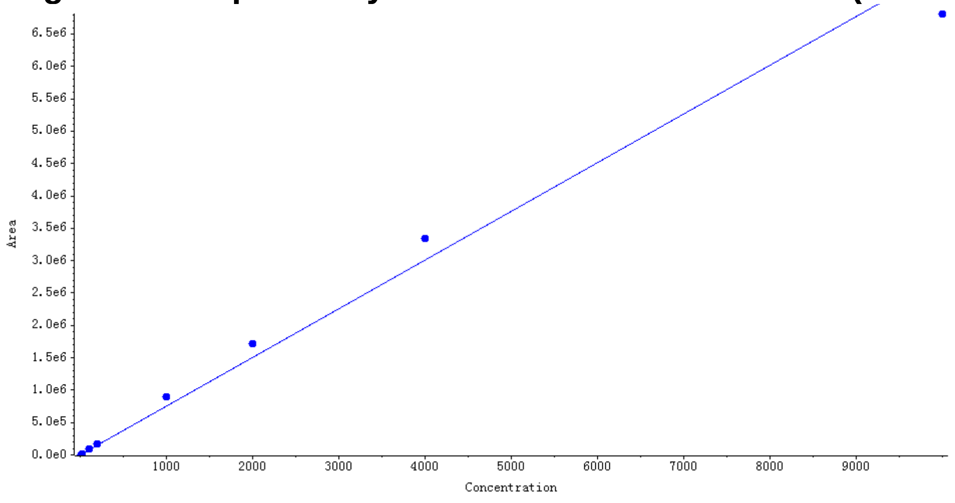

Peak Review

Blank
ST AREA:N/A S/N:N/A

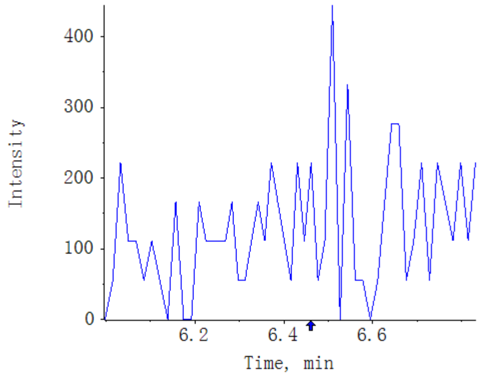

V3.0_MWMS_20240725_1
ST AREA:8.21e4 S/N:8.2

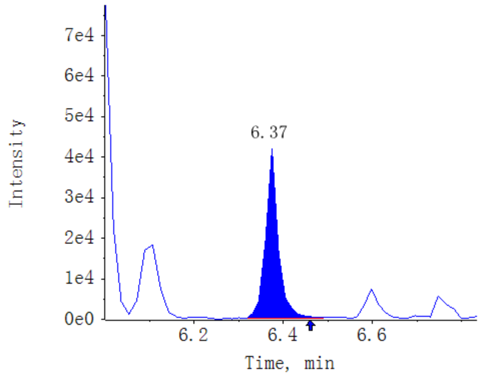

T24186682b_a
ST AREA:N/A S/N:N/A

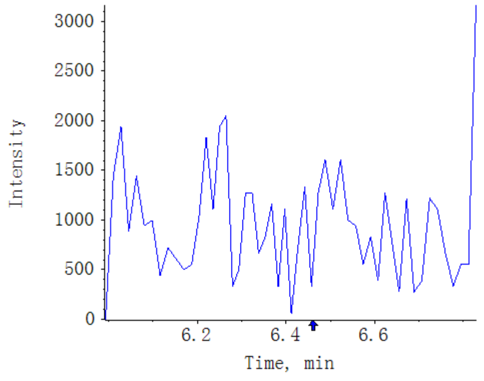

T24186682b_b
ST AREA:N/A S/N:N/A

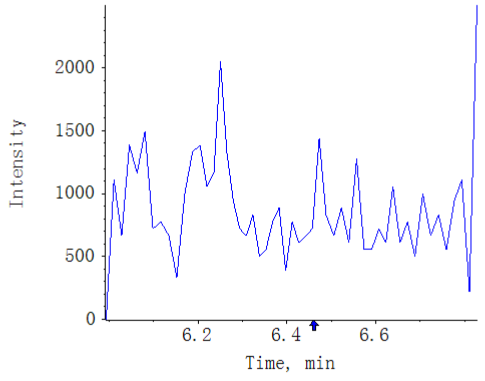

T24186682b_c
ST AREA:N/A S/N:N/A

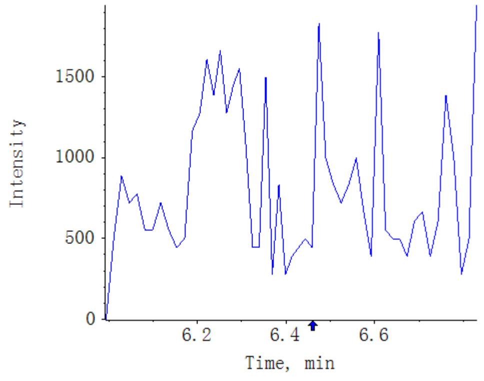

T24186683b_a
ST AREA:N/A S/N:N/A

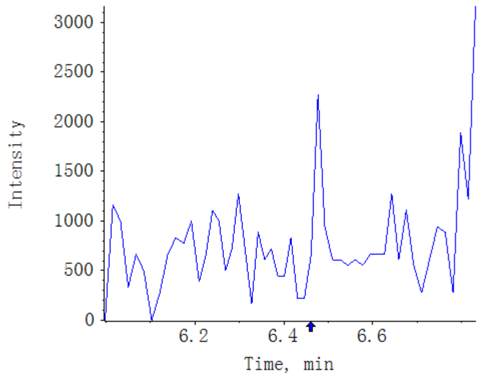

T24186683b_b
ST AREA:N/A S/N:N/A

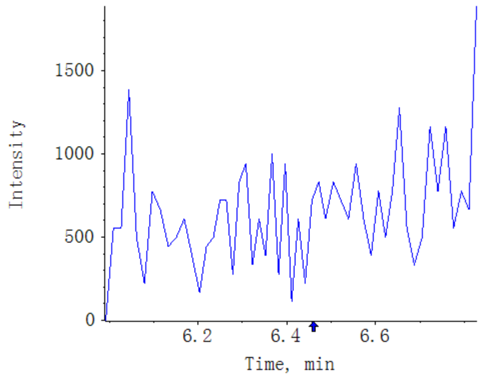

T24186683b_c
ST AREA:N/A S/N:N/A

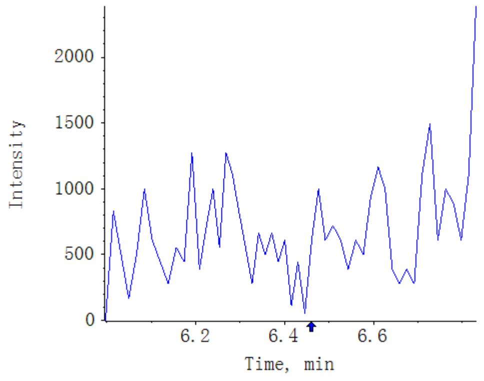

T24186684b_a
ST AREA:N/A S/N:N/A

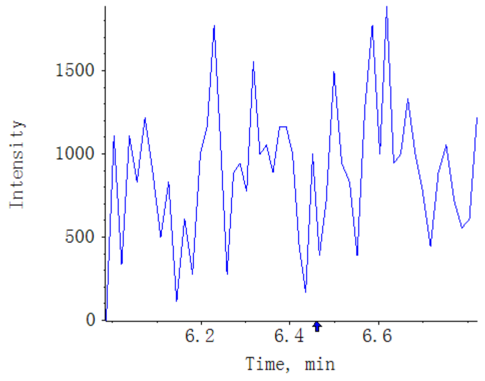

T24186684b_b
ST AREA:N/A S/N:N/A

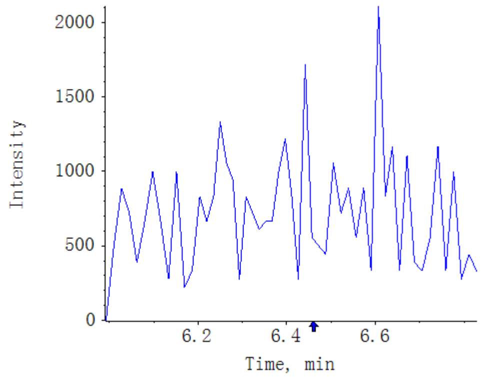

T24186684b_c
ST AREA:N/A S/N:N/A

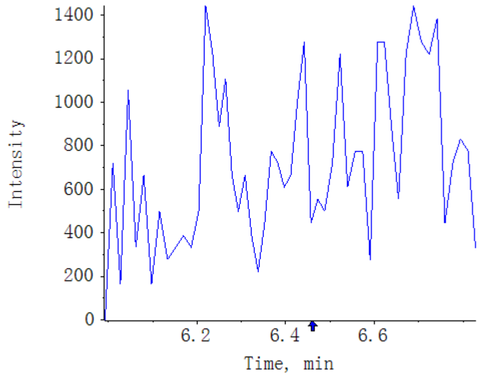

Result Table	MWXS-24-3064-a_9_WH6500-17_A20-3_V6.0_WSS_20240730	Algorithm Used	MQ4
Acquisition Method	ACC-PHs_V6.0_WH6500-17_CMY_20240521.dam	Instrument Name	Triple Quad 6500+ Low Mass
Project	N/A	Analytes QTY	109:74

Compound name: ACC (102.2 / 56.0)

Sample Name	Sample Type	Area (cps)	Is Area (cps)	RT (min)	S/N	Target Conc	Calculated Conc.()
STD_0.01ppb	Standard	N/A	N/A	N/A	N/A	0.0100	N/A
STD_0.05ppb	Standard	N/A	N/A	N/A	N/A	0.0500	N/A
STD_0.1ppb	Standard	N/A	N/A	N/A	N/A	0.1000	N/A
STD_0.5ppb	Standard	1.43e4	N/A	0.71	18.6	0.5000	3.820352e-1
STD_1ppb	Standard	1.90e4	N/A	0.71	26.4	1.0000	6.908279e-1
STD_5ppb	Standard	1.01e5	N/A	0.73	64.7	5.0000	6.108006e0
STD_10ppb	Standard	1.85e5	N/A	0.71	80.9	10.0000	1.168885e1
STD_50ppb	Standard	9.47e5	N/A	0.71	136.3	50.0000	6.236438e1
STD_100ppb	Standard	1.46e6	N/A	0.72	115.1	100.0000	9.619973e1
STD_200ppb	Standard	2.85e6	N/A	0.71	171.2	200.0000	1.890662e2
STD_500ppb	Standard	N/A	N/A	N/A	N/A	500.0000	N/A
V2.0_MW_RQC1_20240724	Quality Control	5.42e5	N/A	0.77	20.2	0.0000	3.544204e1
Blank	Unknown	N/A	N/A	N/A	N/A	N/A	N/A
V3.0_MWMS_20240725_1	Unknown	1.50e5	N/A	0.73	48.2	N/A	9.369486e0
MWXS243064a_R1	Quality Control	N/A	N/A	N/A	N/A	0.0000	N/A
MWXS243064a_R2	Quality Control	N/A	N/A	N/A	N/A	0.0000	N/A
MWXS243064a_R3	Quality Control	N/A	N/A	N/A	N/A	0.0000	N/A
T24186682b_a	Unknown	N/A	N/A	N/A	N/A	N/A	N/A
T24186682b_b	Unknown	N/A	N/A	N/A	N/A	N/A	N/A
T24186682b_c	Unknown	N/A	N/A	N/A	N/A	N/A	N/A
T24186683b_a	Unknown	N/A	N/A	N/A	N/A	N/A	N/A
T24186683b_b	Unknown	N/A	N/A	N/A	N/A	N/A	N/A
T24186683b_c	Unknown	N/A	N/A	N/A	N/A	N/A	N/A
T24186684b_a	Unknown	N/A	N/A	N/A	N/A	N/A	N/A
T24186684b_b	Unknown	N/A	N/A	N/A	N/A	N/A	N/A
T24186684b_c	Unknown	N/A	N/A	N/A	N/A	N/A	N/A

Compound name: ACC
Regression Equation: y = 15051.77405 x + 8590.95274 (r = 0.99373) (weighting: 1 / x)

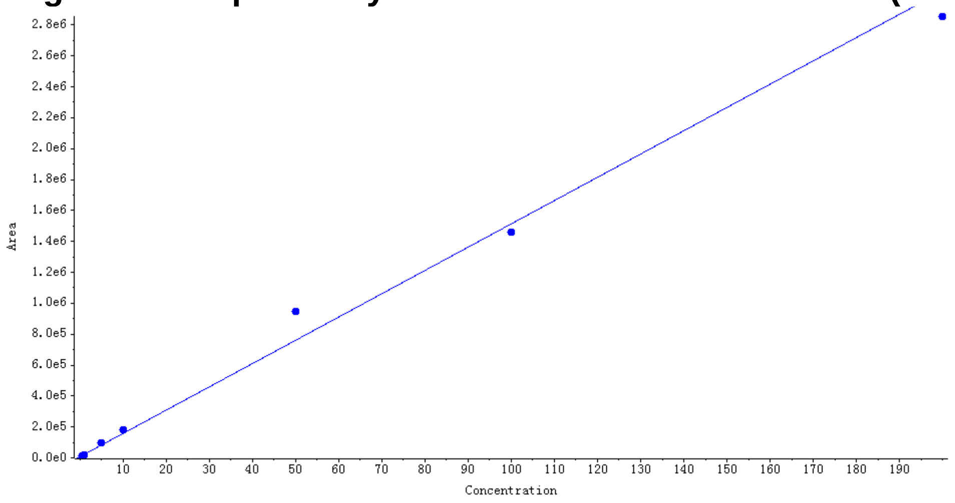

Peak Review

Blank

ACC AREA:N/A S/N:N/A

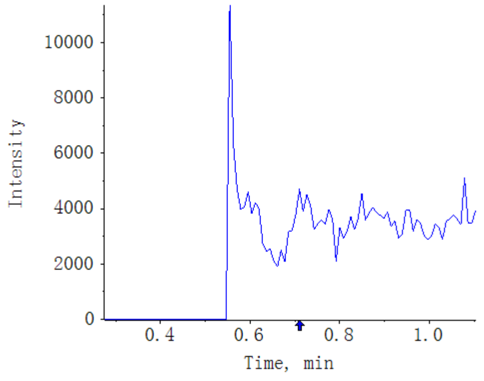

V3.0_MWMS_20240725_1

ACC AREA:1.50e5 S/N:48.2

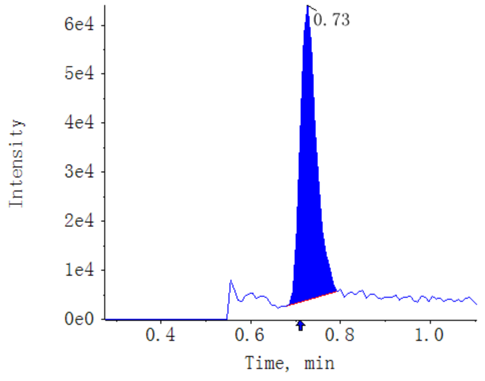

T24186682b_a

ACC AREA:N/A S/N:N/A

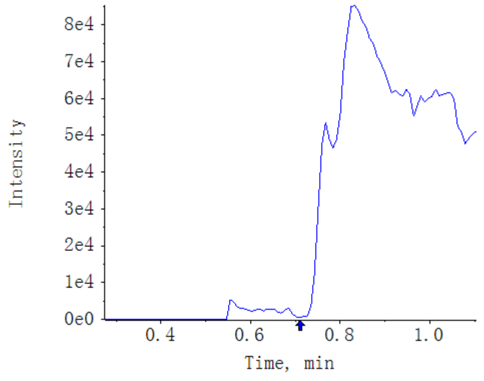

T24186682b_b

ACC AREA:N/A S/N:N/A

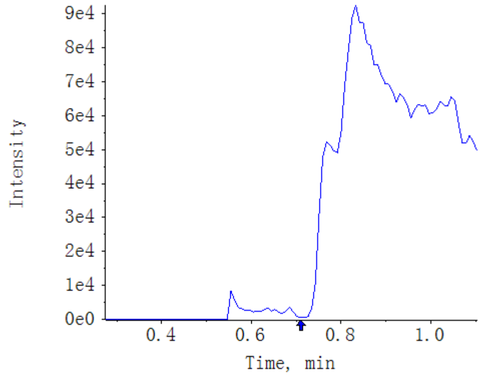

T24186682b_c

ACC AREA:N/A S/N:N/A

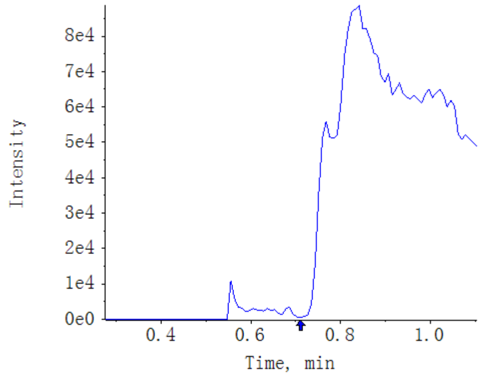

T24186683b_a

ACC AREA:N/A S/N:N/A

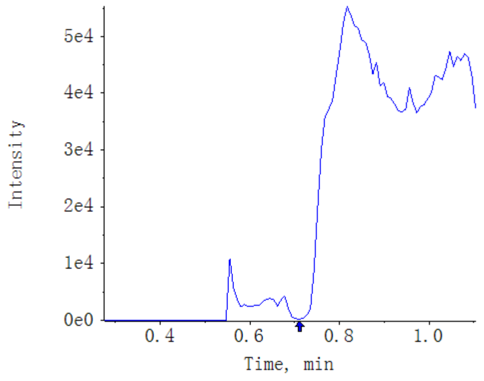

T24186683b_b

ACC AREA:N/A S/N:N/A

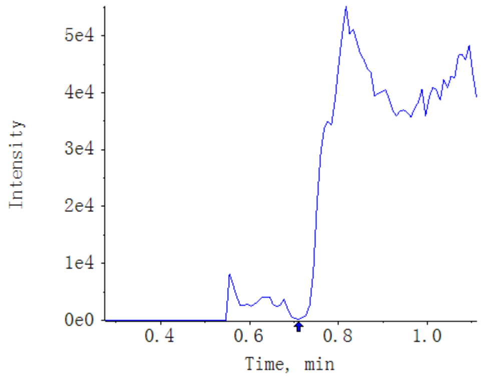

T24186683b_c

ACC AREA:N/A S/N:N/A

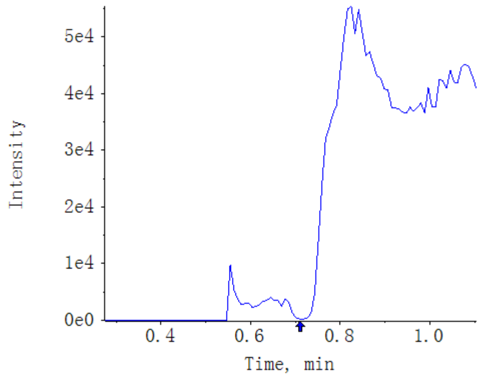

T24186684b_a

ACC AREA:N/A S/N:N/A

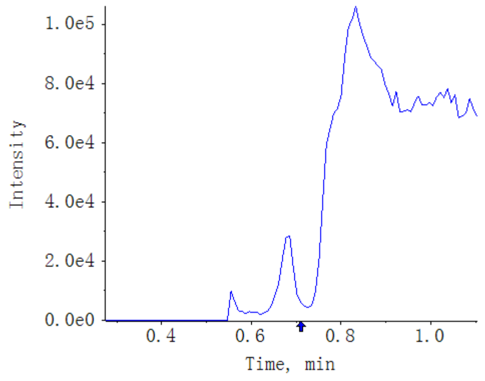

T24186684b_b

ACC AREA:N/A S/N:N/A

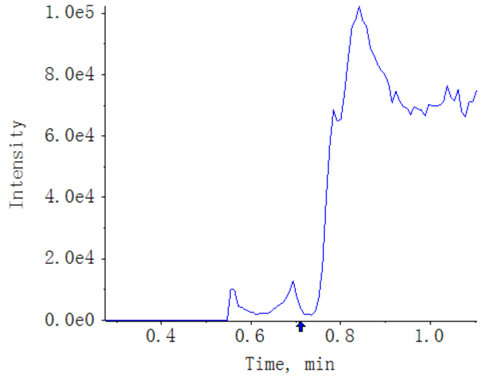

T24186684b_c

ACC AREA:N/A S/N:N/A

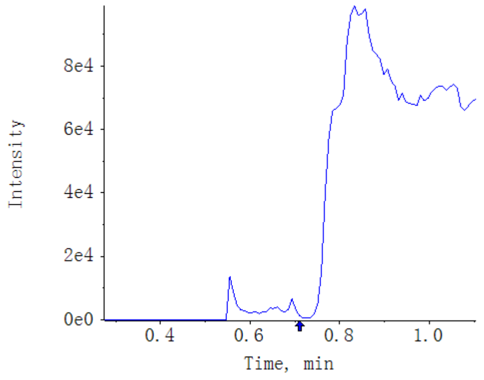

Result Table	MWXS-24-3064-a_9_WH6500-17_A20-3_V6.0_WSS_20240730	Algorithm Used	MQ4
Acquisition Method	ACC-PHs_V6.0_WH6500-17_CMY_20240521.dam	Instrument Name	Triple Quad 6500+ Low Mass
Project	N/A	Analytes QTY	109:75

Compound name: ABA (263.1 / 153.1)

Sample Name	Sample Type	Area (cps)	Is Area (cps)	RT (min)	S/N	Target Conc	Calculated Conc.()
STD_0.01ppb	Standard	N/A	1.776e6	N/A	N/A	0.0100	N/A
STD_0.05ppb	Standard	6.48e3	1.714e6	5.32	28.5	0.0500	7.392064e-2
STD_0.1ppb	Standard	1.02e4	1.713e6	5.32	24.5	0.1000	1.153874e-1
STD_0.5ppb	Standard	3.82e4	1.581e6	5.31	30.0	0.5000	4.585427e-1
STD_1ppb	Standard	5.73e4	1.620e6	5.31	32.4	1.0000	6.693404e-1
STD_5ppb	Standard	4.46e5	1.820e6	5.32	54.0	5.0000	4.627936e0
STD_10ppb	Standard	7.12e5	1.674e6	5.31	71.2	10.0000	8.020029e0
STD_50ppb	Standard	4.71e6	1.688e6	5.31	40.2	50.0000	5.268484e1
STD_100ppb	Standard	N/A	1.498e6	N/A	N/A	100.0000	N/A
STD_200ppb	Standard	N/A	1.443e6	N/A	N/A	200.0000	N/A
STD_500ppb	Standard	N/A	1.136e6	N/A	N/A	500.0000	N/A
V2.0_MW_RQC1_20240724	Quality Control	1.72e5	3.207e5	5.29	23.0	0.0000	1.011069e1
Blank	Unknown	N/A	4.110e2	N/A	N/A	N/A	N/A
V3.0_MWMS_20240725_1	Unknown	4.62e5	1.002e6	5.29	44.8	N/A	8.695969e0
MWXS243064a_R1	Quality Control	2.93e5	4.433e5	5.27	73.7	0.0000	1.245619e1
MWXS243064a_R2	Quality Control	2.79e5	4.254e5	5.28	66.1	0.0000	1.236517e1
MWXS243064a_R3	Quality Control	2.78e5	4.264e5	5.27	70.4	0.0000	1.230229e1
T24186682b_a	Unknown	3.31e5	3.571e5	5.28	56.7	N/A	1.748177e1
T24186682b_b	Unknown	3.18e5	3.443e5	5.28	52.0	N/A	1.744639e1
T24186682b_c	Unknown	3.19e5	3.247e5	5.28	50.0	N/A	1.853592e1
T24186683b_a	Unknown	1.36e5	5.544e5	5.28	41.3	N/A	4.617798e0
T24186683b_b	Unknown	1.34e5	5.030e5	5.28	44.0	N/A	5.045544e0
T24186683b_c	Unknown	1.37e5	5.433e5	5.28	53.4	N/A	4.767014e0
T24186684b_a	Unknown	4.01e5	4.203e5	5.28	47.7	N/A	1.801438e1
T24186684b_b	Unknown	3.51e5	4.614e5	5.29	57.0	N/A	1.433910e1
T24186684b_c	Unknown	3.23e5	4.338e5	5.29	60.2	N/A	1.405214e1

Compound name: ABA
Regression Equation: y = 0.05300 x + -1.41068e-4 (r = 0.99475) (weighting: 1 / x)

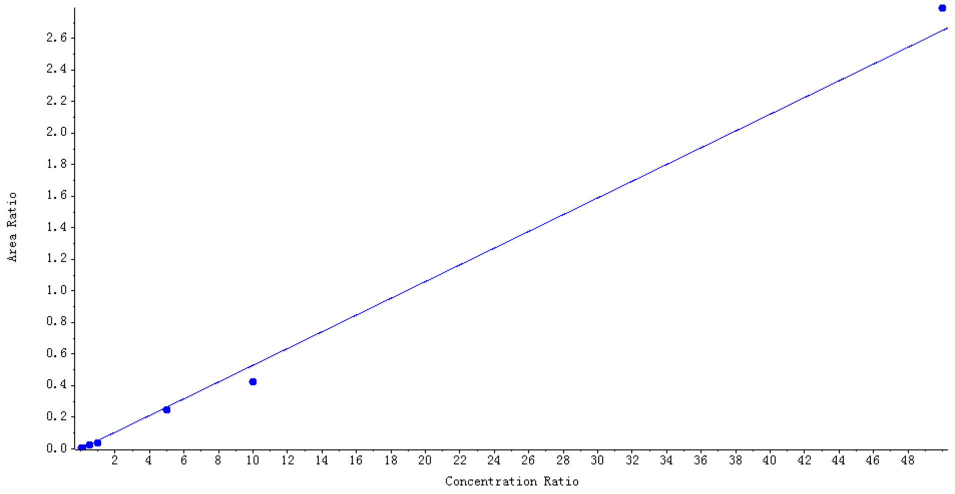

Peak Review

Blank
ABA AREA:N/A S/N:N/A

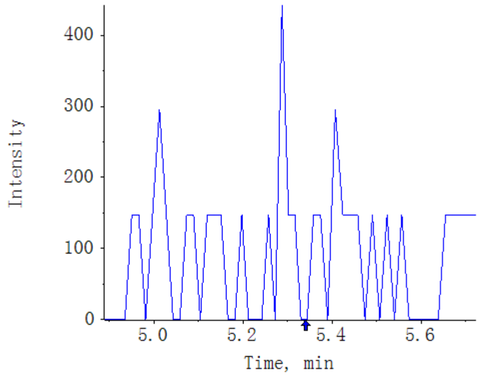

V3.0_MWMS_20240725_1
ABA AREA:4.62e5 S/N:44.8

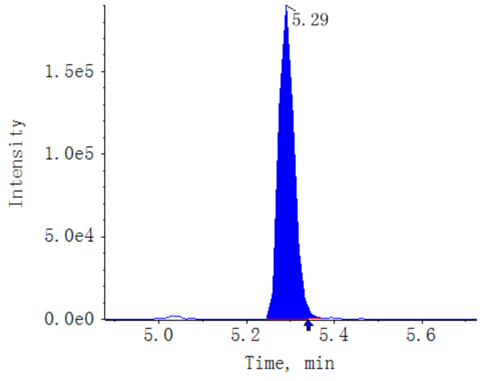

T24186682b_a
ABA AREA:3.31e5 S/N:56.7

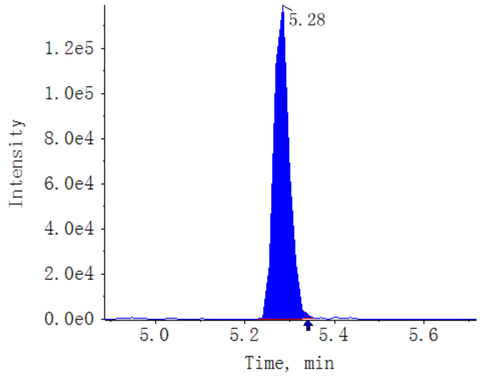

T24186682b_b
ABA AREA:3.18e5 S/N:52.0

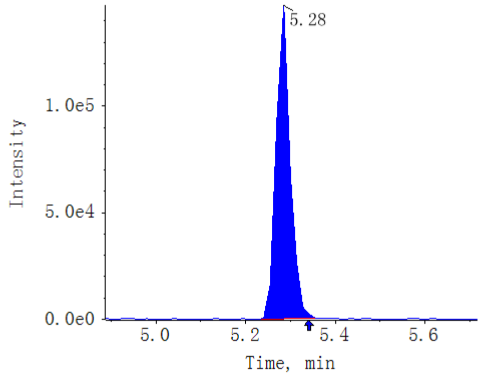

T24186682b_c
ABA AREA:3.19e5 S/N:50.0

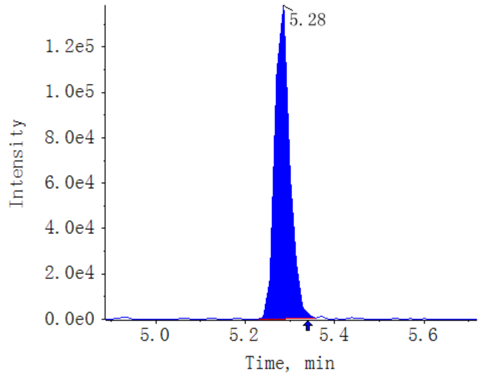

T24186683b_a
ABA AREA:1.36e5 S/N:41.3

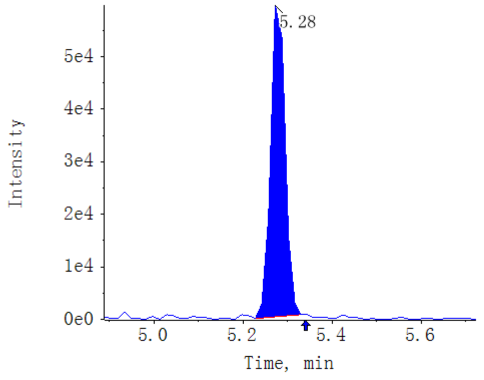

T24186683b_b
ABA AREA:1.34e5 S/N:44.0

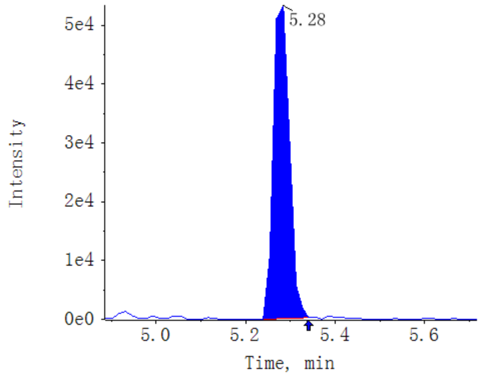

T24186683b_c
ABA AREA:1.37e5 S/N:53.4

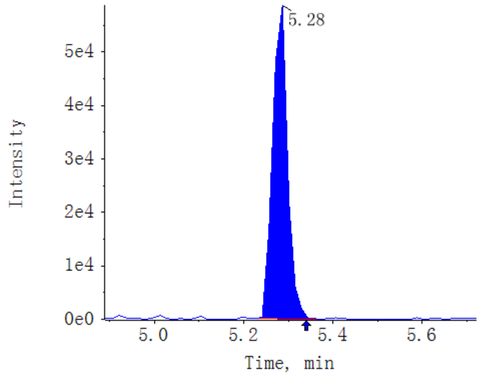

T24186684b_a
ABA AREA:4.01e5 S/N:47.7

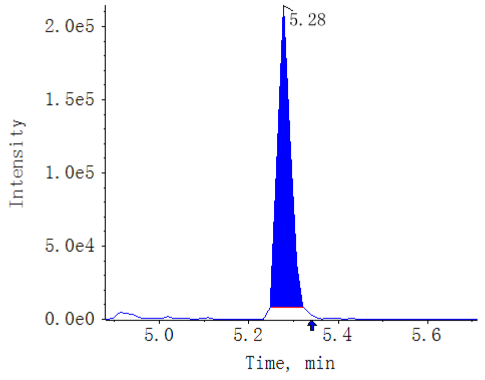

T24186684b_b
ABA AREA:3.51e5 S/N:57.0

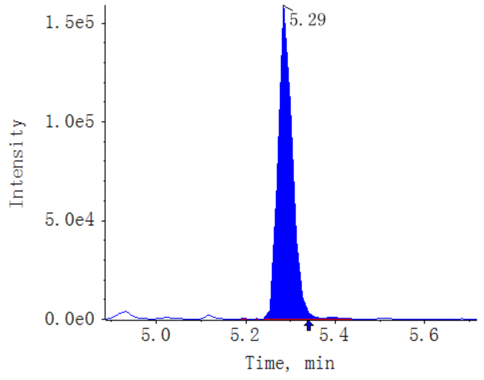

T24186684b_c
ABA AREA:3.23e5 S/N:60.2

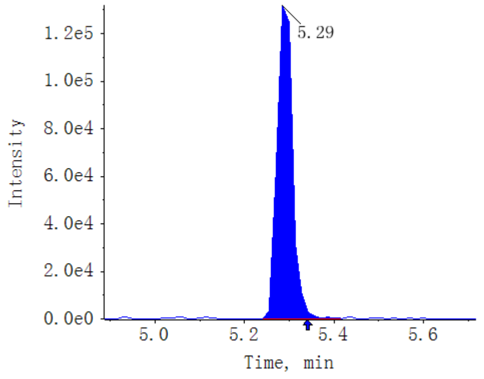

Result Table	MWXS-24-3064-a_9_WH6500-17_A20-3_V6.0_WSS_20240730	Algorithm Used	MQ4
Acquisition Method	ACC-PHs_V6.0_WH6500-17_CMY_20240521.dam	Instrument Name	Triple Quad 6500+ Low Mass
Project	N/A	Analytes QTY	109:76

Compound name: IAA-Glu (303.0 / 145.9)

Sample Name	Sample Type	Area (cps)	Is Area (cps)	RT (min)	S/N	Target Conc	Calculated Conc.()
STD_0.01ppb	Standard	8.17e2	4.457e5	4.40	8.9	0.0100	9.683887e-3
STD_0.05ppb	Standard	3.94e3	4.759e5	4.40	14.9	0.0500	5.910302e-2
STD_0.1ppb	Standard	6.21e3	4.571e5	4.41	33.6	0.1000	9.986273e-2
STD_0.5ppb	Standard	2.76e4	4.822e5	4.40	52.3	0.5000	4.348341e-1
STD_1ppb	Standard	4.64e4	4.362e5	4.41	44.0	1.0000	8.111185e-1
STD_5ppb	Standard	2.75e5	4.553e5	4.42	70.6	5.0000	4.621552e0
STD_10ppb	Standard	5.15e5	4.191e5	4.40	62.0	10.0000	9.410108e0
STD_50ppb	Standard	2.90e6	3.976e5	4.40	62.4	50.0000	5.593339e1
STD_100ppb	Standard	5.74e6	3.709e5	4.41	42.0	100.0000	1.186148e2
STD_200ppb	Standard	N/A	3.446e5	N/A	N/A	200.0000	N/A
STD_500ppb	Standard	N/A	2.675e5	N/A	N/A	500.0000	N/A
V2.0_MW_RQC1_20240724	Quality Control	N/A	1.104e5	N/A	N/A	0.0000	N/A
Blank	Unknown	N/A	3.683e3	N/A	N/A	N/A	N/A
V3.0_MWMS_20240725_1	Unknown	3.56e5	5.371e5	4.40	34.8	N/A	5.084638e0
MWXS243064a_R1	Quality Control	5.29e3	2.172e5	4.37	7.2	0.0000	1.823184e-1
MWXS243064a_R2	Quality Control	3.23e3	2.207e5	4.37	5.4	0.0000	1.078406e-1
MWXS243064a_R3	Quality Control	5.67e3	2.224e5	4.37	7.5	0.0000	1.911572e-1
T24186682b_a	Unknown	4.61e3	2.103e5	4.37	4.5	N/A	1.635454e-1
T24186682b_b	Unknown	3.12e3	1.670e5	4.38	5.1	N/A	1.388620e-1
T24186682b_c	Unknown	5.63e3	1.830e5	4.37	9.9	N/A	2.312903e-1
T24186683b_a	Unknown	3.95e3	2.452e5	4.37	6.7	N/A	1.190372e-1
T24186683b_b	Unknown	3.02e3	2.437e5	4.40	5.8	N/A	9.057372e-2
T24186683b_c	Unknown	3.60e3	2.504e5	4.38	5.7	N/A	1.059180e-1
T24186684b_a	Unknown	4.80e5	2.130e5	4.38	32.2	N/A	1.727355e1
T24186684b_b	Unknown	7.71e4	2.404e5	4.39	28.1	N/A	2.453565e0
T24186684b_c	Unknown	2.20e3	2.153e5	4.38	5.4	N/A	7.395379e-2

Compound name: IAA-Glu
Regression Equation: y = 0.13041 x + 5.70974e-4 (r = 0.99024) (weighting: 1 / x^2)

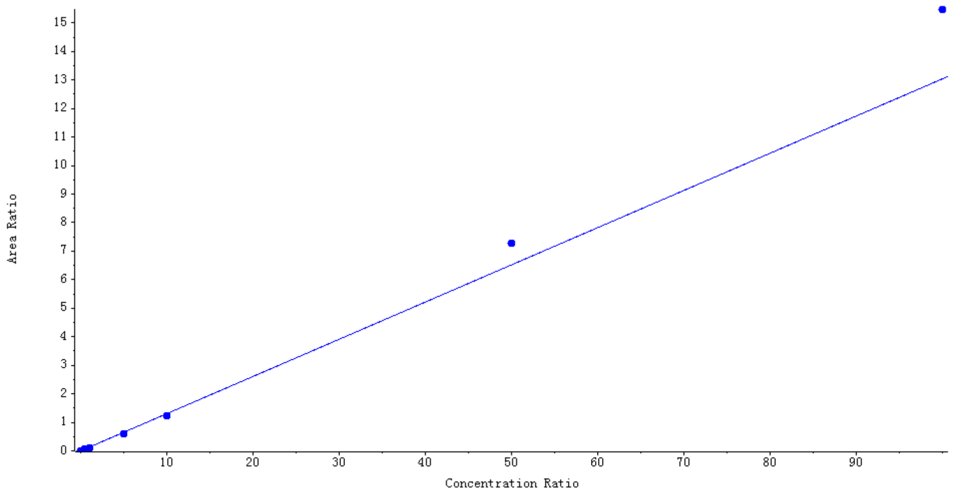

Peak Review

Blank
IAA-Glu AREA:N/A S/N:N/A

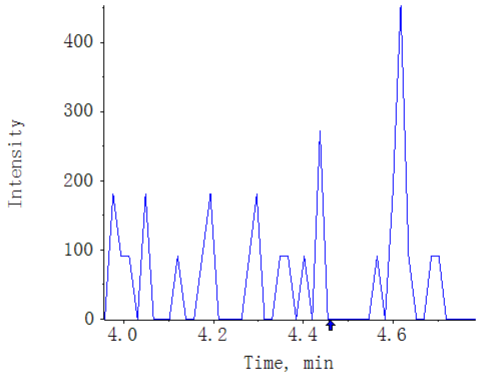

V3.0_MWMS_20240725_1
IAA-Glu AREA:3.56e5 S/N:34.8

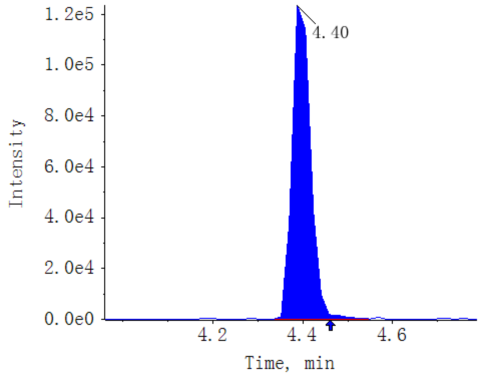

T24186682b_a
IAA-Glu AREA:4.61e3 S/N:4.5

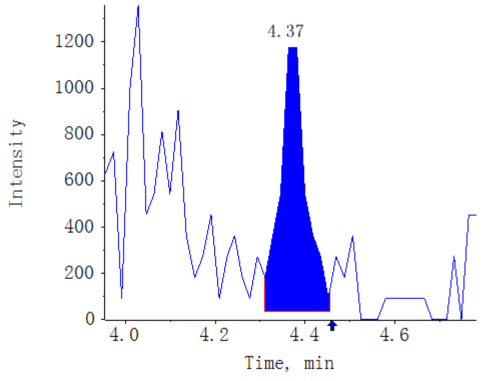

T24186682b_b
IAA-Glu AREA:3.12e3 S/N:5.1

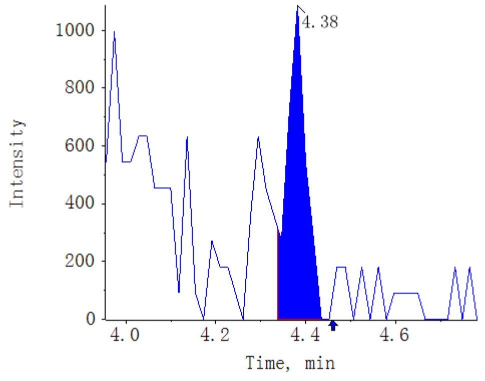

T24186682b_c
IAA-Glu AREA:5.63e3 S/N:9.9

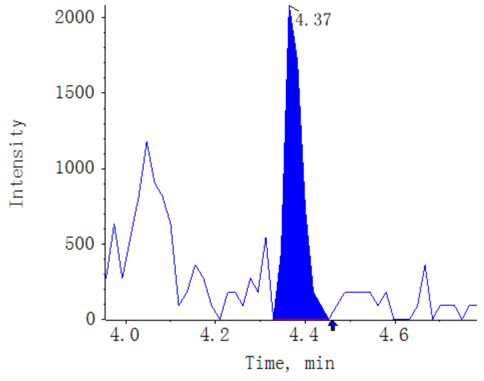

T24186683b_a
IAA-Glu AREA:3.95e3 S/N:6.7

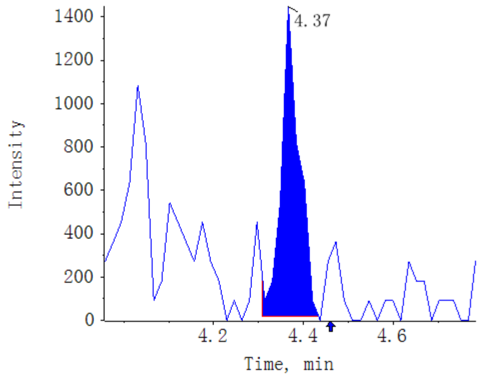

T24186683b_b
IAA-Glu AREA:3.02e3 S/N:5.8

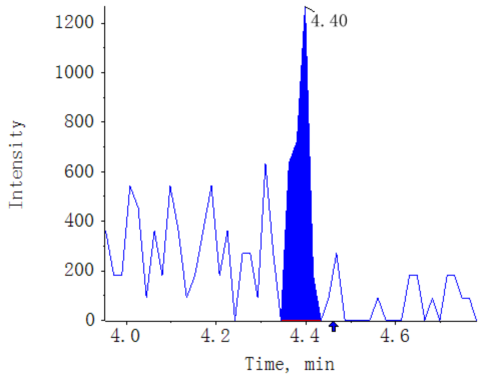

T24186683b_c
IAA-Glu AREA:3.60e3 S/N:5.7

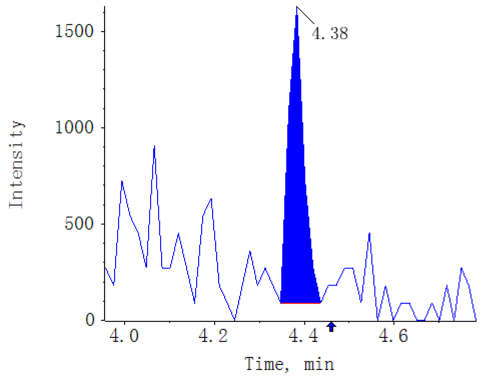

T24186684b_a
IAA-Glu AREA:4.80e5 S/N:32.2

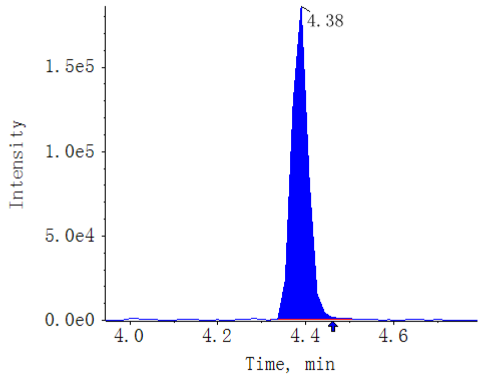

T24186684b_b
IAA-Glu AREA:7.71e4 S/N:28.1

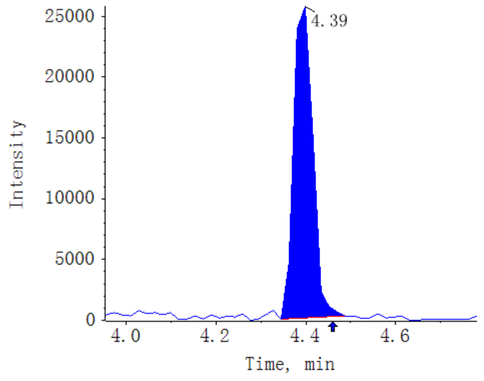

T24186684b_c
IAA-Glu AREA:2.20e3 S/N:5.4

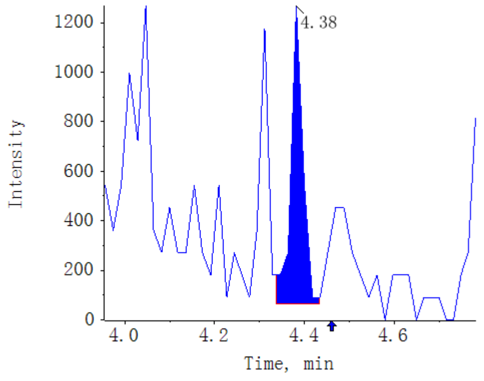

Result Table	MWXS-24-3064-a_9_WH6500-17_A20-3_V6.0_WSS_20240730	Algorithm Used	MQ4
Acquisition Method	ACC-PHs_V6.0_WH6500-17_CMY_20240521.dam	Instrument Name	Triple Quad 6500+ Low Mass
Project	N/A	Analytes QTY	109:77

Compound name: IAA-Glu-diMe (331.0 / 115.8)

Sample Name	Sample Type	Area (cps)	Is Area (cps)	RT (min)	S/N	Target Conc	Calculated Conc.()
STD_0.01ppb	Standard	N/A	4.457e5	N/A	N/A	0.0100	N/A
STD_0.05ppb	Standard	1.47e3	4.759e5	5.57	14.3	0.0500	4.968202e-2
STD_0.1ppb	Standard	2.51e3	4.571e5	5.59	13.9	0.1000	1.048477e-1
STD_0.5ppb	Standard	9.62e3	4.822e5	5.58	19.8	0.5000	4.377105e-1
STD_1ppb	Standard	1.67e4	4.362e5	5.59	38.5	1.0000	8.624362e-1
STD_5ppb	Standard	1.06e5	4.553e5	5.59	51.4	5.0000	5.357044e0
STD_10ppb	Standard	2.09e5	4.191e5	5.58	56.3	10.0000	1.148617e1
STD_50ppb	Standard	N/A	3.976e5	N/A	N/A	50.0000	N/A
STD_100ppb	Standard	N/A	3.709e5	N/A	N/A	100.0000	N/A
STD_200ppb	Standard	N/A	3.446e5	N/A	N/A	200.0000	N/A
STD_500ppb	Standard	N/A	2.675e5	N/A	N/A	500.0000	N/A
V2.0_MW_RQC1_20240724	Quality Control	N/A	1.104e5	N/A	N/A	0.0000	N/A
Blank	Unknown	N/A	3.683e3	N/A	N/A	N/A	N/A
V3.0_MWMS_20240725_1	Unknown	1.14e5	5.371e5	5.55	40.6	N/A	4.885417e0
MWXS243064a_R1	Quality Control	N/A	2.172e5	N/A	N/A	0.0000	N/A
MWXS243064a_R2	Quality Control	N/A	2.207e5	N/A	N/A	0.0000	N/A
MWXS243064a_R3	Quality Control	N/A	2.224e5	N/A	N/A	0.0000	N/A
T24186682b_a	Unknown	N/A	2.103e5	N/A	N/A	N/A	N/A
T24186682b_b	Unknown	N/A	1.670e5	N/A	N/A	N/A	N/A
T24186682b_c	Unknown	N/A	1.830e5	N/A	N/A	N/A	N/A
T24186683b_a	Unknown	N/A	2.452e5	N/A	N/A	N/A	N/A
T24186683b_b	Unknown	N/A	2.437e5	N/A	N/A	N/A	N/A
T24186683b_c	Unknown	N/A	2.504e5	N/A	N/A	N/A	N/A
T24186684b_a	Unknown	N/A	2.130e5	N/A	N/A	N/A	N/A
T24186684b_b	Unknown	N/A	2.404e5	N/A	N/A	N/A	N/A
T24186684b_c	Unknown	N/A	2.153e5	N/A	N/A	N/A	N/A

Compound name: IAA-Glu-diMe
Regression Equation: y = 0.04344 x + 9.33845e-4 (r = 0.99169) (weighting: 1 / x^2)

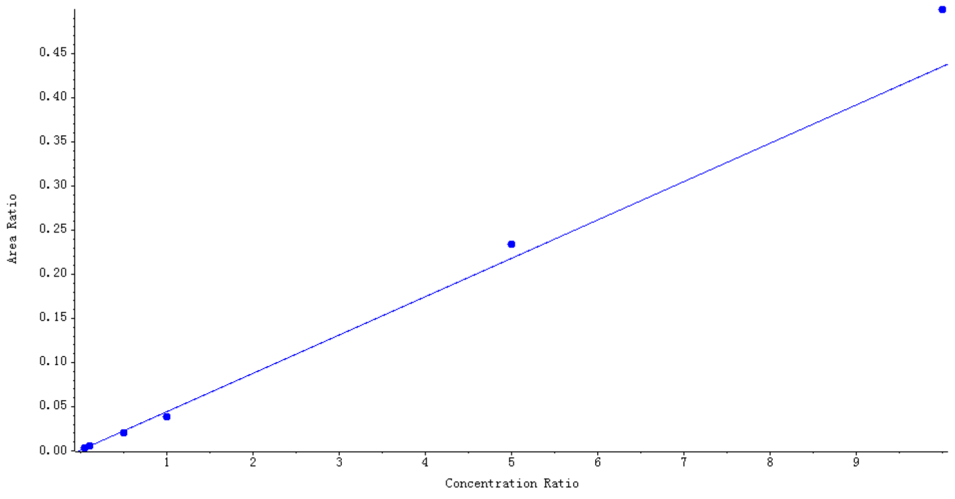

Peak Review

Blank
IAA-Glu-diMe AREA:N/A S/N:N/A

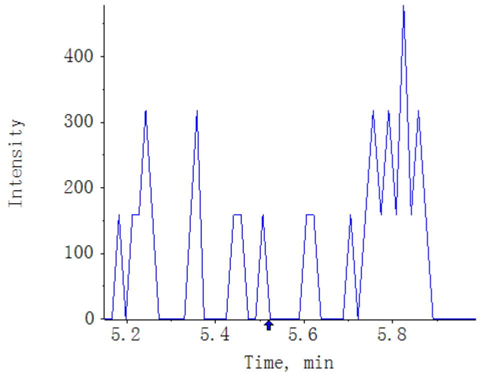

V3.0_MWMS_20240725_1
IAA-Glu-diMe AREA:1.14e5 S/N:40.6

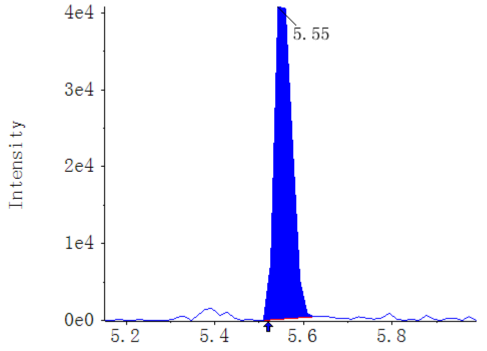

T24186682b_a
IAA-Glu-diMe AREA:N/A S/N:N/A

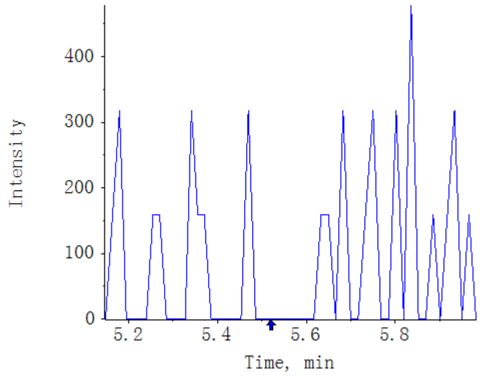

T24186682b_b
IAA-Glu-diMe AREA:N/A S/N:N/A

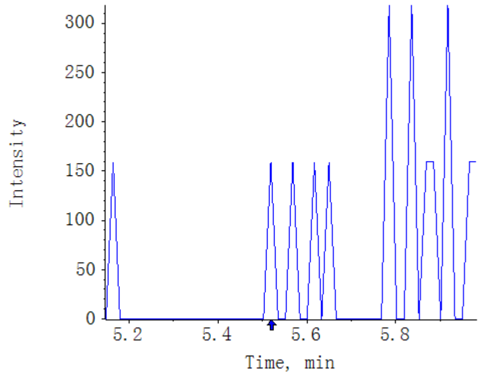

T24186682b_c
IAA-Glu-diMe AREA:N/A S/N:N/A

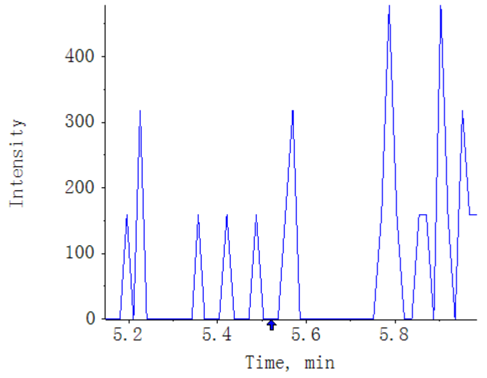

T24186683b_a
IAA-Glu-diMe AREA:N/A S/N:N/A

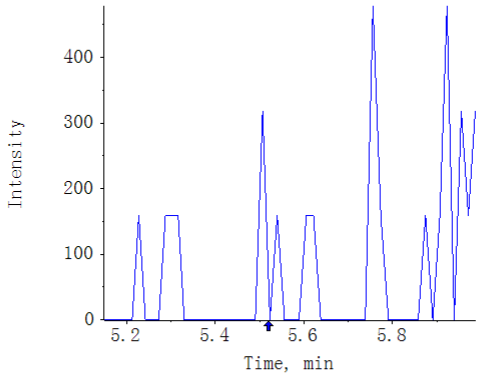

T24186683b_b
IAA-Glu-diMe AREA:N/A S/N:N/A

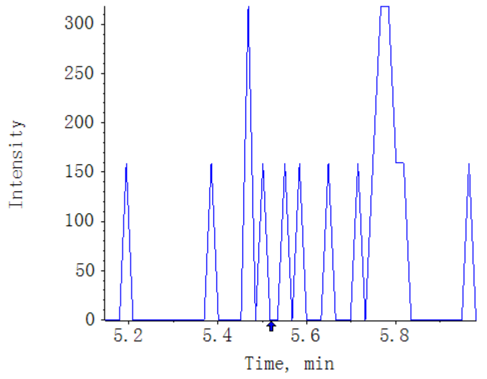

T24186683b_c
IAA-Glu-diMe AREA:N/A S/N:N/A

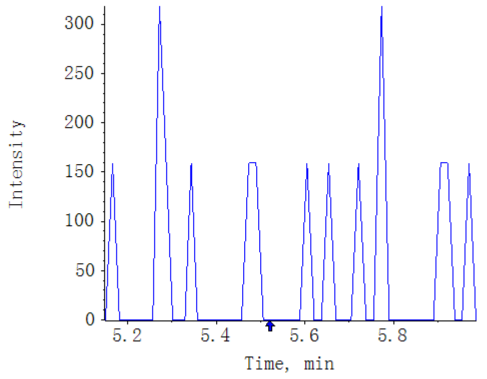

T24186684b_a
IAA-Glu-diMe AREA:N/A S/N:N/A

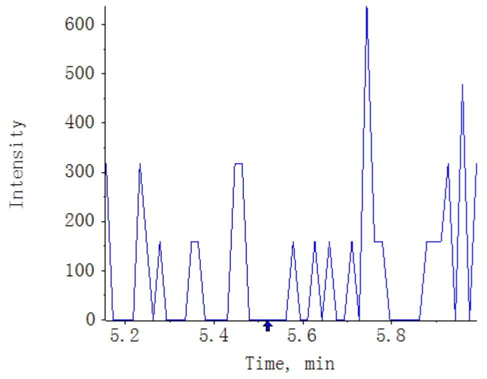

T24186684b_b
IAA-Glu-diMe AREA:N/A S/N:N/A

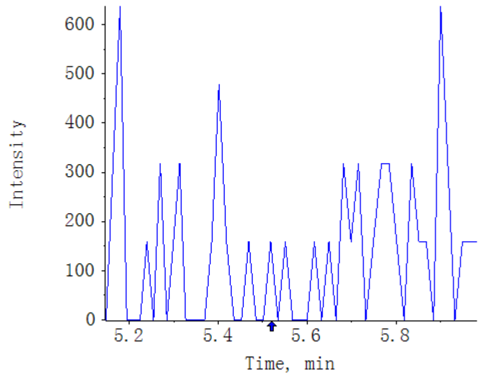

T24186684b_c
IAA-Glu-diMe AREA:N/A S/N:N/A

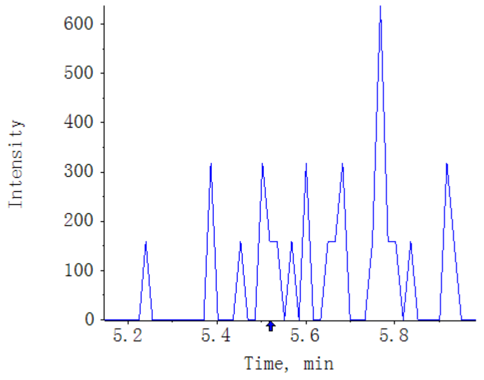

Result Table	MWXS-24-3064-a_9_WH6500-17_A20-3_V6.0_WSS_20240730	Algorithm Used	MQ4
Acquisition Method	ACC-PHs_V6.0_WH6500-17_CMY_20240521.dam	Instrument Name	Triple Quad 6500+ Low Mass
Project	N/A	Analytes QTY	109:78

Compound name: IAA-Asp (288.9 / 131.9)

Sample Name	Sample Type	Area (cps)	Is Area (cps)	RT (min)	S/N	Target Conc	Calculated Conc.()
STD_0.01ppb	Standard	N/A	2.428e6	N/A	N/A	0.0100	N/A
STD_0.05ppb	Standard	N/A	2.591e6	N/A	N/A	0.0500	N/A
STD_0.1ppb	Standard	3.17e3	2.431e6	4.29	26.0	0.1000	1.024039e-1
STD_0.5ppb	Standard	1.95e4	2.438e6	4.28	37.4	0.5000	4.762539e-1
STD_1ppb	Standard	3.65e4	2.460e6	4.28	36.0	1.0000	8.587116e-1
STD_5ppb	Standard	2.06e5	2.300e6	4.29	41.5	5.0000	5.036034e0
STD_10ppb	Standard	3.62e5	2.197e6	4.28	49.3	10.0000	9.240600e0
STD_50ppb	Standard	2.06e6	2.190e6	4.28	38.1	50.0000	5.258814e1
STD_100ppb	Standard	4.12e6	2.261e6	4.29	54.2	100.0000	1.019180e2
STD_200ppb	Standard	9.67e6	2.322e6	4.28	34.6	200.0000	2.325065e2
STD_500ppb	Standard	N/A	2.806e6	N/A	N/A	500.0000	N/A
V2.0_MW_RQC1_20240724	Quality Control	8.35e3	1.125e6	4.27	13.7	0.0000	4.439667e-1
Blank	Unknown	N/A	9.166e2	N/A	N/A	N/A	N/A
V3.0_MWMS_20240725_1	Unknown	2.20e5	1.991e6	4.27	26.1	N/A	6.214958e0
MWXS243064a_R1	Quality Control	2.85e4	2.449e6	4.25	12.8	0.0000	6.793146e-1
MWXS243064a_R2	Quality Control	2.73e4	2.428e6	4.26	13.3	0.0000	6.573695e-1
MWXS243064a_R3	Quality Control	2.74e4	2.131e6	4.25	15.7	0.0000	7.489312e-1
T24186682b_a	Unknown	1.42e4	2.686e6	4.26	7.8	N/A	3.242647e-1
T24186682b_b	Unknown	1.13e4	2.457e6	4.26	8.6	N/A	2.873865e-1
T24186682b_c	Unknown	1.52e4	2.706e6	4.26	12.2	N/A	3.422192e-1
T24186683b_a	Unknown	1.45e4	2.328e6	4.25	10.8	N/A	3.779726e-1
T24186683b_b	Unknown	1.20e4	2.370e6	4.26	8.7	N/A	3.124527e-1
T24186683b_c	Unknown	1.65e4	2.503e6	4.26	12.4	N/A	3.987029e-1
T24186684b_a	Unknown	2.47e6	2.515e6	4.26	25.0	N/A	5.485484e1
T24186684b_b	Unknown	5.15e5	2.330e6	4.27	23.0	N/A	1.237996e1
T24186684b_c	Unknown	5.60e3	2.293e6	4.27	6.1	N/A	1.659635e-1

Compound name: IAA-Asp
Regression Equation: y = 0.01791 x + -5.28172e-4 (r = 0.99543) (weighting: 1 / x^2)

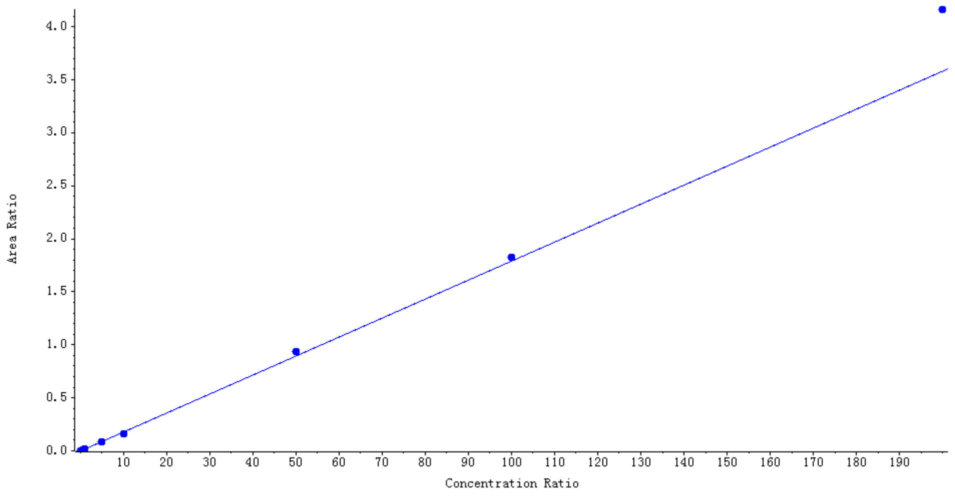

Peak Review

Blank
IAA-Asp AREA:N/A S/N:N/A

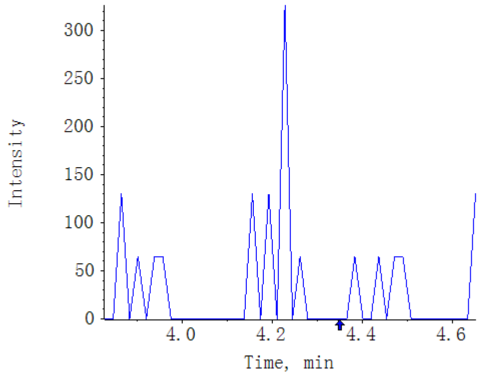

V3.0_MWMS_20240725_1
IAA-Asp AREA:2.20e5 S/N:26.1

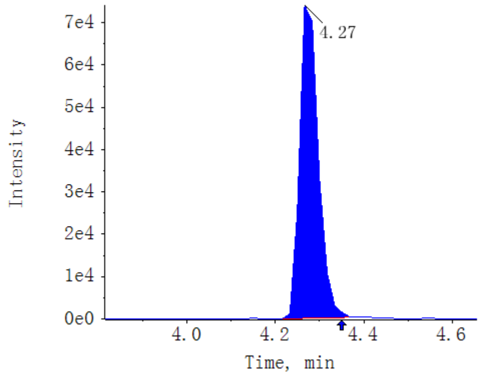

T24186682b_a
IAA-Asp AREA:1.42e4 S/N:7.8

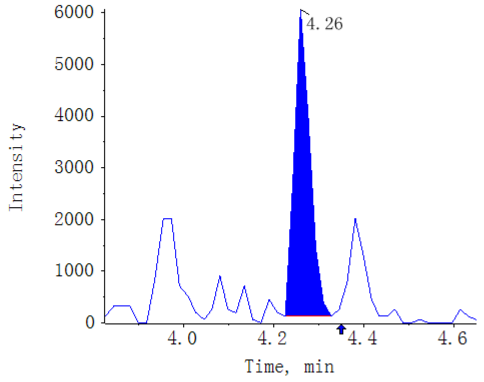

T24186682b_b
IAA-Asp AREA:1.13e4 S/N:8.6

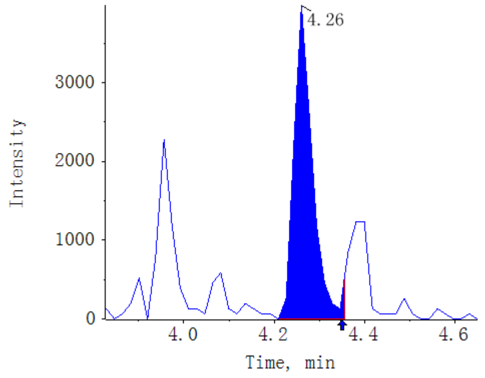

T24186682b_c
IAA-Asp AREA:1.52e4 S/N:12.2

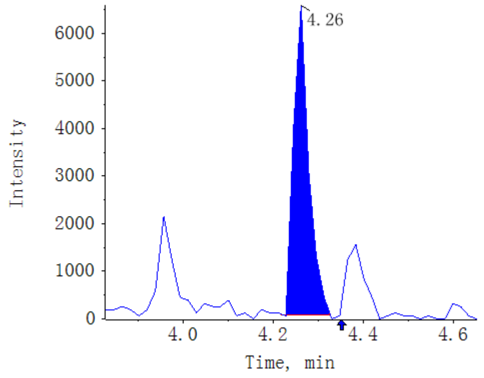

T24186683b_a
IAA-Asp AREA:1.45e4 S/N:10.8

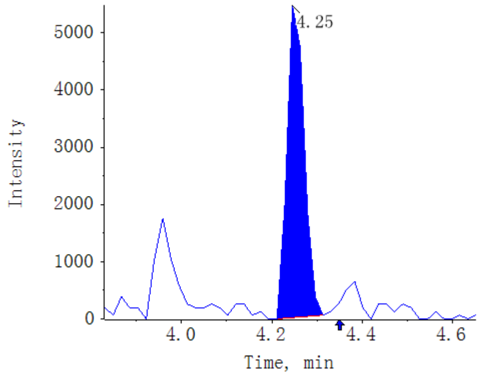

T24186683b_b
IAA-Asp AREA:1.20e4 S/N:8.7

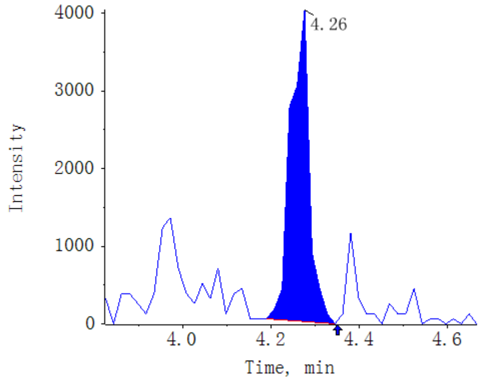

T24186683b_c
IAA-Asp AREA:1.65e4 S/N:12.4

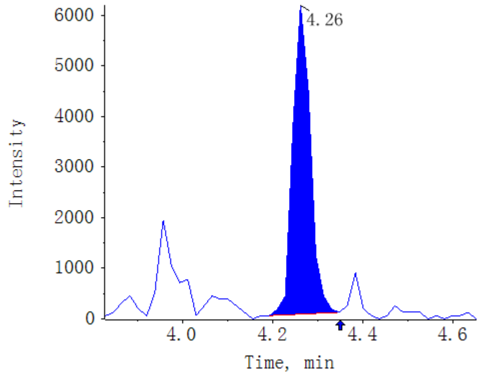

T24186684b_a
IAA-Asp AREA:2.47e6 S/N:25.0

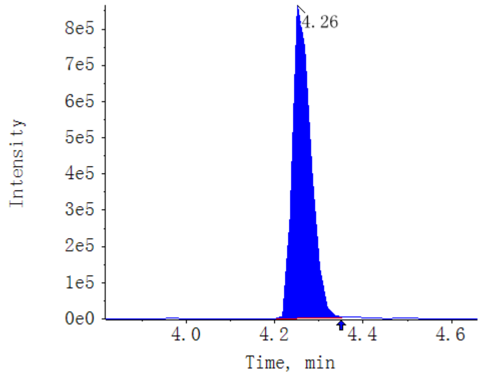

T24186684b_b
IAA-Asp AREA:5.15e5 S/N:23.0

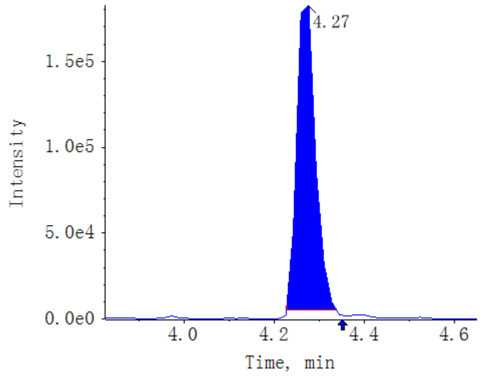

T24186684b_c
IAA-Asp AREA:5.60e3 S/N:6.1

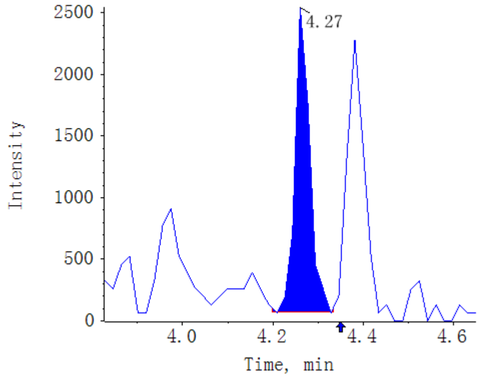

Result Table	MWXS-24-3064-a_9_WH6500-17_A20-3_V6.0_WSS_20240730	Algorithm Used	MQ4
Acquisition Method	ACC-PHs_V6.0_WH6500-17_CMY_20240521.dam	Instrument Name	Triple Quad 6500+ Low Mass
Project	N/A	Analytes QTY	109:79

Compound name: IAA-Trp (360.0 / 203.0)

Sample Name	Sample Type	Area (cps)	Is Area (cps)	RT (min)	S/N	Target Conc	Calculated Conc.()
STD_0.01ppb	Standard	N/A	4.457e5	N/A	N/A	0.0100	N/A
STD_0.05ppb	Standard	1.01e4	4.759e5	5.75	38.7	0.0500	5.343236e-2
STD_0.1ppb	Standard	1.48e4	4.571e5	5.76	36.1	0.1000	8.790636e-2
STD_0.5ppb	Standard	7.88e4	4.822e5	5.76	48.7	0.5000	4.867303e-1
STD_1ppb	Standard	1.28e5	4.362e5	5.76	70.5	1.0000	8.842459e-1
STD_5ppb	Standard	7.64e5	4.553e5	5.77	65.4	5.0000	5.098259e0
STD_10ppb	Standard	1.34e6	4.191e5	5.76	58.1	10.0000	9.728108e0
STD_50ppb	Standard	7.85e6	3.976e5	5.76	76.0	50.0000	6.010601e1
STD_100ppb	Standard	N/A	3.709e5	N/A	N/A	100.0000	N/A
STD_200ppb	Standard	N/A	3.446e5	N/A	N/A	200.0000	N/A
STD_500ppb	Standard	N/A	2.675e5	N/A	N/A	500.0000	N/A
V2.0_MW_RQC1_20240724	Quality Control	N/A	1.104e5	N/A	N/A	0.0000	N/A
Blank	Unknown	N/A	3.683e3	N/A	N/A	N/A	N/A
V3.0_MWMS_20240725_1	Unknown	9.53e5	5.371e5	5.74	85.0	N/A	5.391107e0
MWXS243064a_R1	Quality Control	N/A	2.172e5	N/A	N/A	0.0000	N/A
MWXS243064a_R2	Quality Control	N/A	2.207e5	N/A	N/A	0.0000	N/A
MWXS243064a_R3	Quality Control	N/A	2.224e5	N/A	N/A	0.0000	N/A
T24186682b_a	Unknown	N/A	2.103e5	N/A	N/A	N/A	N/A
T24186682b_b	Unknown	N/A	1.670e5	N/A	N/A	N/A	N/A
T24186682b_c	Unknown	N/A	1.830e5	N/A	N/A	N/A	N/A
T24186683b_a	Unknown	N/A	2.452e5	N/A	N/A	N/A	N/A
T24186683b_b	Unknown	N/A	2.437e5	N/A	N/A	N/A	N/A
T24186683b_c	Unknown	N/A	2.504e5	N/A	N/A	N/A	N/A
T24186684b_a	Unknown	N/A	2.130e5	N/A	N/A	N/A	N/A
T24186684b_b	Unknown	N/A	2.404e5	N/A	N/A	N/A	N/A
T24186684b_c	Unknown	N/A	2.153e5	N/A	N/A	N/A	N/A

Compound name: IAA-Trp
Regression Equation: y = 0.32842 x + 0.00361 (r = 0.99224) (weighting: 1 / x^2)

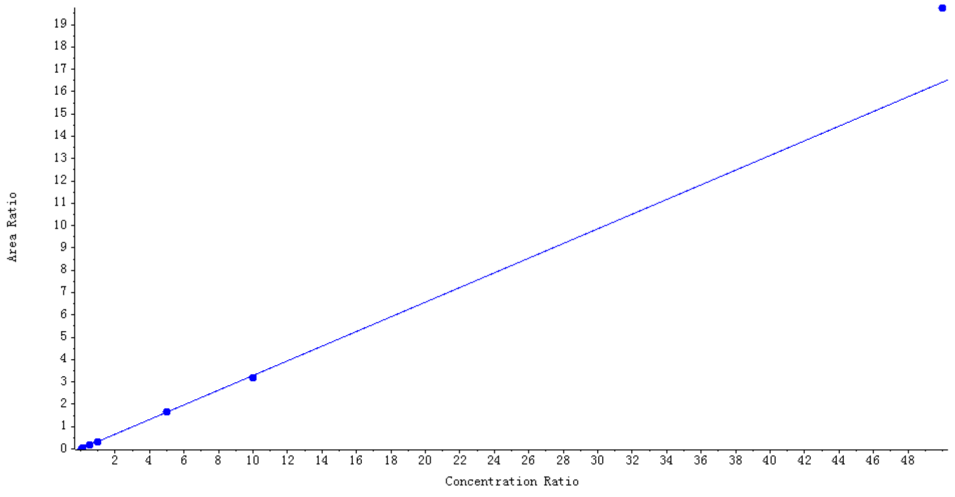

Peak Review

Blank

IAA-Trp AREA:N/A S/N:N/A

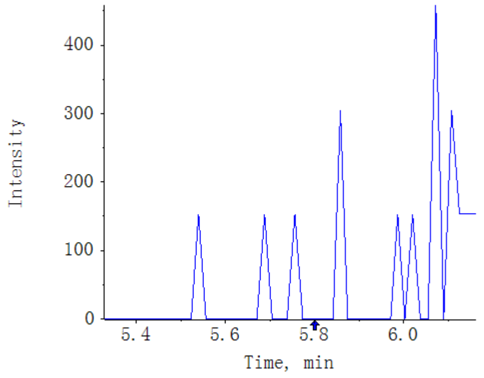

V3.0_MWMS_20240725_1

IAA-Trp AREA:9.53e5 S/N:85.0

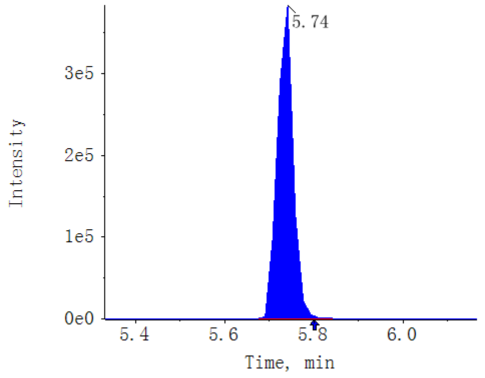

T24186682b_a

IAA-Trp AREA:N/A S/N:N/A

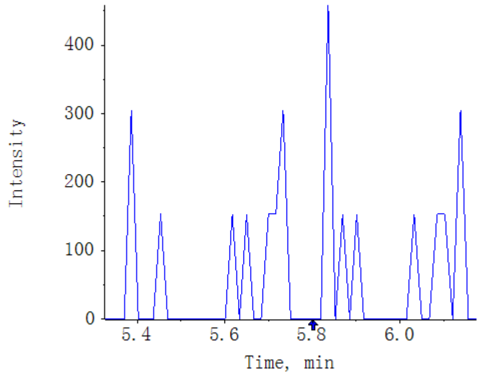

T24186682b_b

IAA-Trp AREA:N/A S/N:N/A

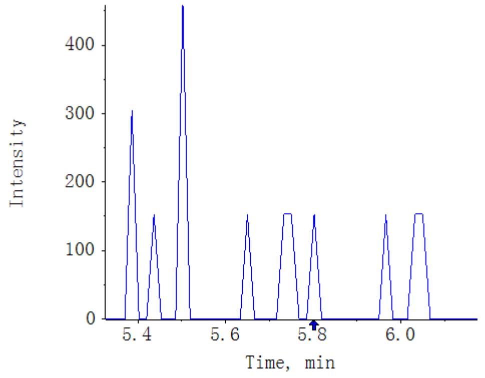

T24186682b_c

IAA-Trp AREA:N/A S/N:N/A

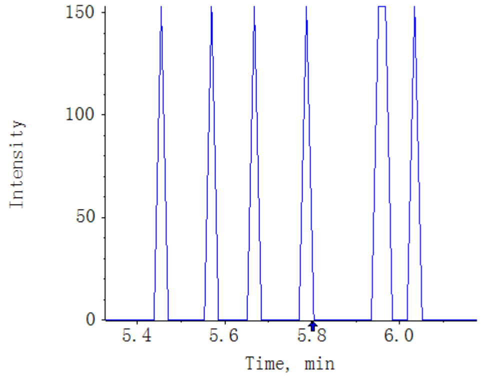

T24186683b_a

IAA-Trp AREA:N/A S/N:N/A

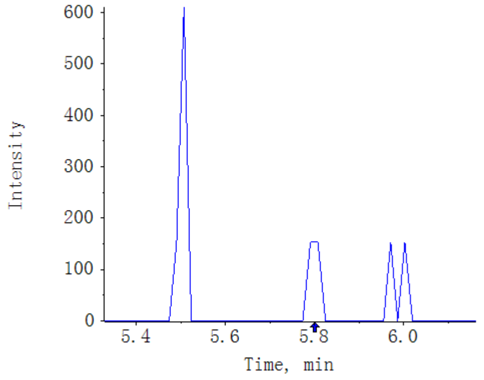

T24186683b_b

IAA-Trp AREA:N/A S/N:N/A

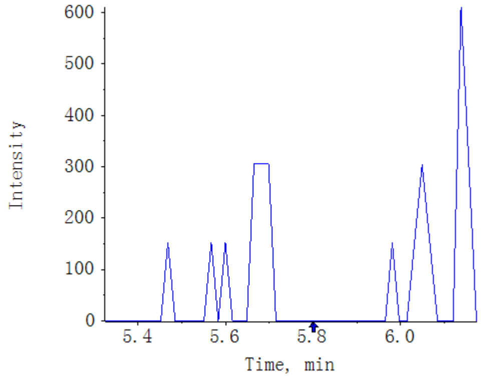

T24186683b_c

IAA-Trp AREA:N/A S/N:N/A

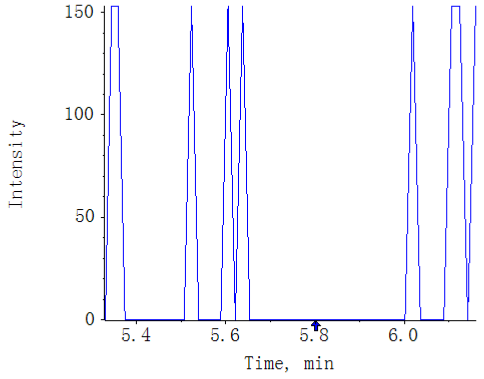

T24186684b_a

IAA-Trp AREA:N/A S/N:N/A

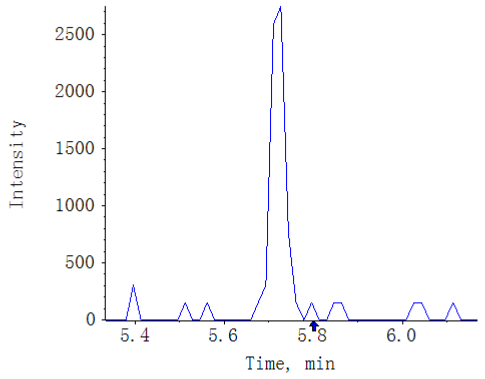

T24186684b_b

IAA-Trp AREA:N/A S/N:N/A

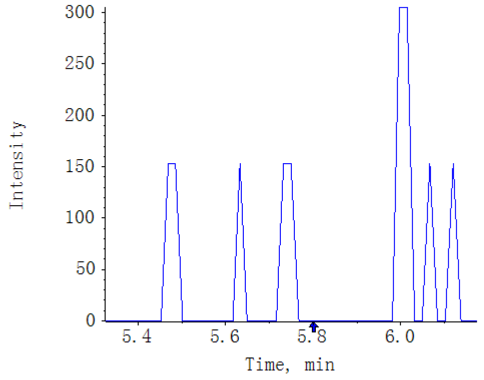

T24186684b_c

IAA-Trp AREA:N/A S/N:N/A

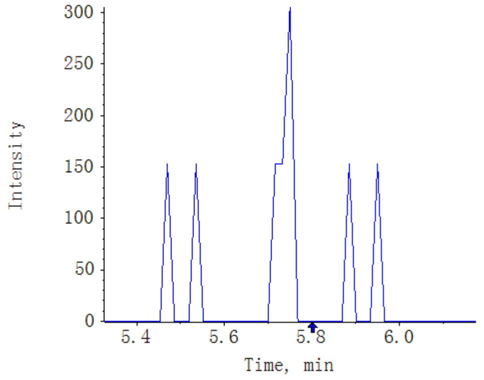

Result Table	MWXS-24-3064-a_9_WH6500-17_A20-3_V6.0_WSS_20240730	Algorithm Used	MQ4
Acquisition Method	ACC-PHs_V6.0_WH6500-17_CMY_20240521.dam	Instrument Name	Triple Quad 6500+ Low Mass
Project	N/A	Analytes QTY	109:80

Compound name: ILA (204.0 / 158.0)

Sample Name	Sample Type	Area (cps)	Is Area (cps)	RT (min)	S/N	Target Conc	Calculated Conc.()
STD_0.01ppb	Standard	N/A	3.406e6	N/A	N/A	0.0100	N/A
STD_0.05ppb	Standard	N/A	3.492e6	N/A	N/A	0.0500	N/A
STD_0.1ppb	Standard	N/A	3.412e6	N/A	N/A	0.1000	N/A
STD_0.5ppb	Standard	2.38e4	3.293e6	4.64	8.4	0.5000	4.799943e-1
STD_1ppb	Standard	5.22e4	3.317e6	4.64	16.8	1.0000	1.102201e0
STD_5ppb	Standard	2.05e5	3.124e6	4.65	34.1	5.0000	4.746535e0
STD_10ppb	Standard	3.68e5	3.024e6	4.64	47.6	10.0000	8.838894e0
STD_50ppb	Standard	2.10e6	3.140e6	4.63	50.2	50.0000	4.884443e1
STD_100ppb	Standard	4.03e6	3.127e6	4.65	54.0	100.0000	9.420314e1
STD_200ppb	Standard	8.58e6	2.901e6	4.64	50.0	200.0000	2.161136e2
STD_500ppb	Standard	2.01e7	2.664e6	4.64	47.0	500.0000	5.525629e2
V2.0_MW_RQC1_20240724	Quality Control	N/A	4.708e5	N/A	N/A	0.0000	N/A
Blank	Unknown	N/A	4.261e2	N/A	N/A	N/A	N/A
V3.0_MWMS_20240725_1	Unknown	2.63e5	2.572e6	4.63	12.7	N/A	7.420651e0
MWXS243064a_R1	Quality Control	N/A	9.151e5	N/A	N/A	0.0000	N/A
MWXS243064a_R2	Quality Control	N/A	8.986e5	N/A	N/A	0.0000	N/A
MWXS243064a_R3	Quality Control	N/A	8.285e5	N/A	N/A	0.0000	N/A
T24186682b_a	Unknown	N/A	7.372e5	N/A	N/A	N/A	N/A
T24186682b_b	Unknown	N/A	6.936e5	N/A	N/A	N/A	N/A
T24186682b_c	Unknown	N/A	7.477e5	N/A	N/A	N/A	N/A
T24186683b_a	Unknown	N/A	9.471e5	N/A	N/A	N/A	N/A
T24186683b_b	Unknown	N/A	1.031e6	N/A	N/A	N/A	N/A
T24186683b_c	Unknown	N/A	1.006e6	N/A	N/A	N/A	N/A
T24186684b_a	Unknown	N/A	1.019e6	N/A	N/A	N/A	N/A
T24186684b_b	Unknown	N/A	1.192e6	N/A	N/A	N/A	N/A
T24186684b_c	Unknown	N/A	1.198e6	N/A	N/A	N/A	N/A

Compound name: ILA
Regression Equation: y = 0.01368 x + 6.51735e-4 (r = 0.99575) (weighting: 1 / x^2)

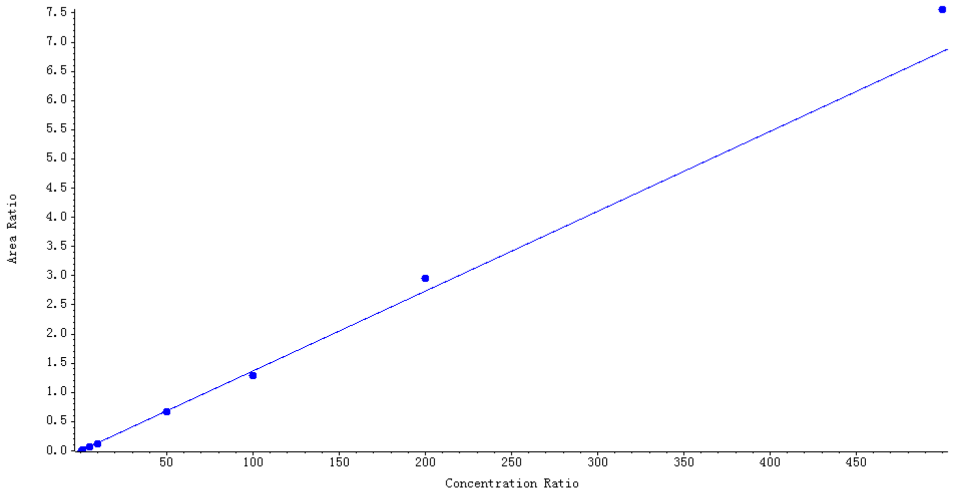

Peak Review

Blank

ILA AREA:N/A S/N:N/A

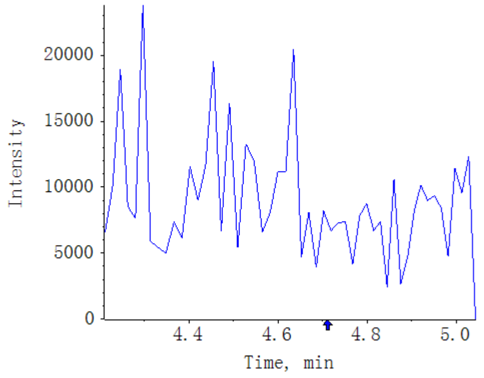

V3.0_MWMS_20240725_1

ILA AREA:2.63e5 S/N:12.7

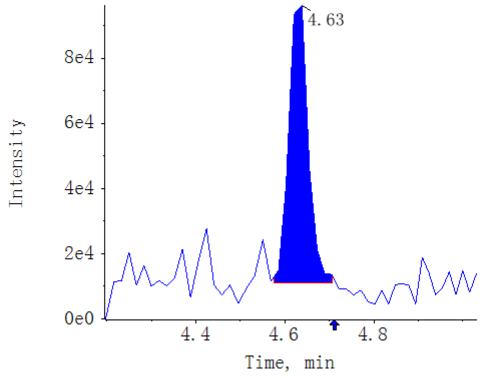

T24186682b_a

ILA AREA:N/A S/N:N/A

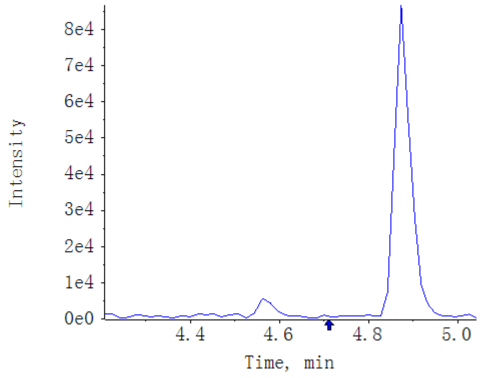

T24186682b_b

ILA AREA:N/A S/N:N/A

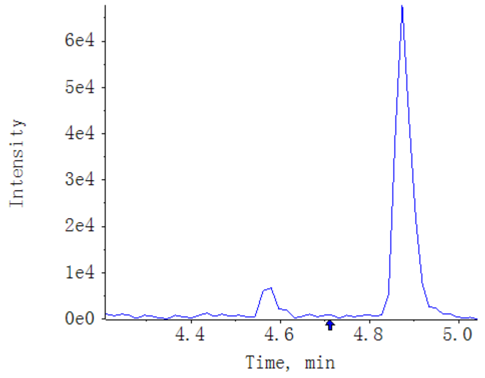

T24186682b_c

ILA AREA:N/A S/N:N/A

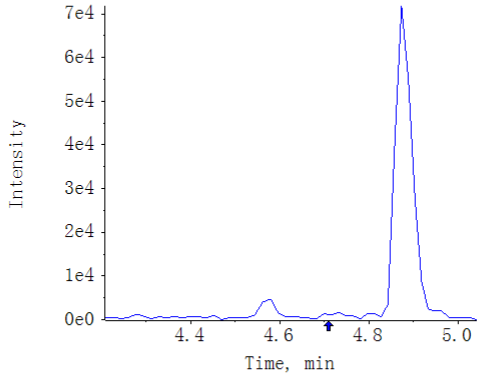

T24186683b_a

ILA AREA:N/A S/N:N/A

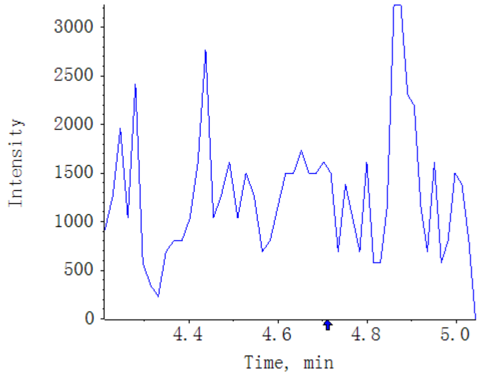

T24186683b_b

ILA AREA:N/A S/N:N/A

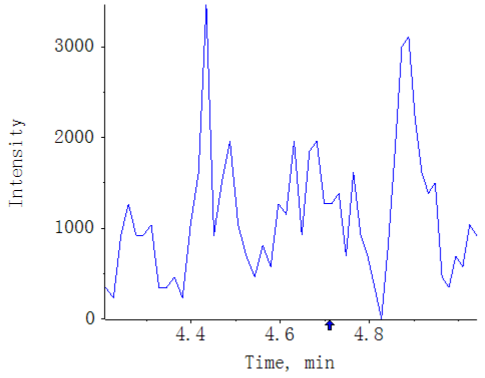

T24186683b_c

ILA AREA:N/A S/N:N/A

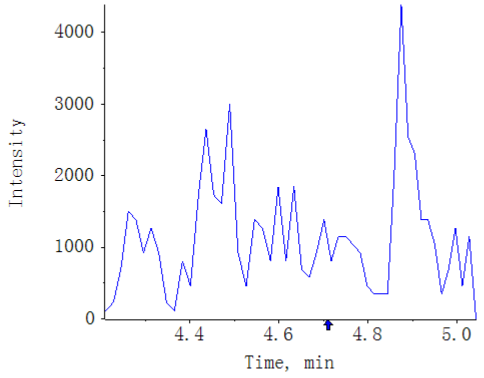

T24186684b_a

ILA AREA:N/A S/N:N/A

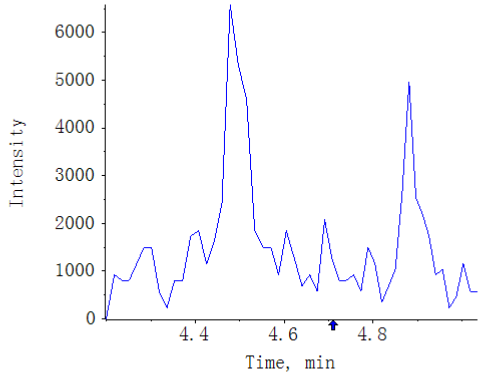

T24186684b_b

ILA AREA:N/A S/N:N/A

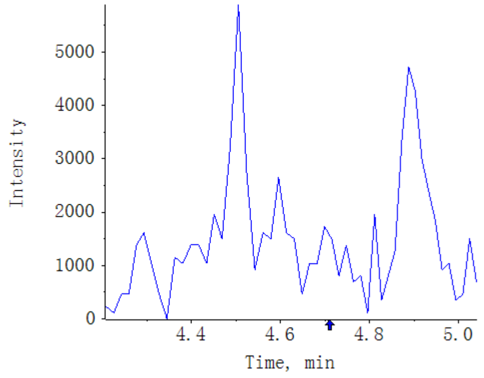

T24186684b_c

ILA AREA:N/A S/N:N/A

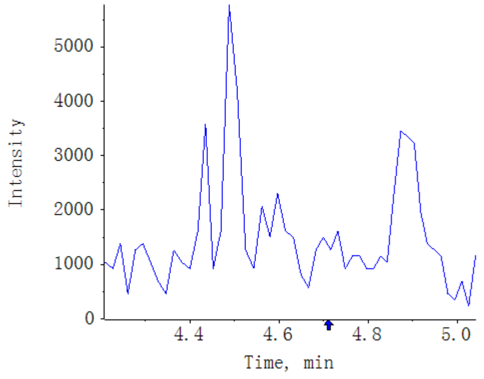

Result Table	MWXS-24-3064-a_9_WH6500-17_A20-3_V6.0_WSS_20240730	Algorithm Used	MQ4
Acquisition Method	ACC-PHs_V6.0_WH6500-17_CMY_20240521.dam	Instrument Name	Triple Quad 6500+ Low Mass
Project	N/A	Analytes QTY	109:81

Compound name: IAA-Phe (321.0 / 164.1)

Sample Name	Sample Type	Area (cps)	Is Area (cps)	RT (min)	S/N	Target Conc	Calculated Conc.()
STD_0.01ppb	Standard	2.37e3	4.457e5	5.87	21.1	0.0100	1.012667e-2
STD_0.05ppb	Standard	1.31e4	4.759e5	5.86	30.8	0.0500	4.971590e-2
STD_0.1ppb	Standard	2.33e4	4.571e5	5.88	42.1	0.1000	9.149596e-2
STD_0.5ppb	Standard	1.26e5	4.822e5	5.86	55.8	0.5000	4.659193e-1
STD_1ppb	Standard	2.04e5	4.362e5	5.87	71.5	1.0000	8.372142e-1
STD_5ppb	Standard	1.22e6	4.553e5	5.88	55.3	5.0000	4.785049e0
STD_10ppb	Standard	2.35e6	4.191e5	5.87	68.5	10.0000	9.989966e0
STD_50ppb	Standard	1.30e7	3.976e5	5.86	55.6	50.0000	5.830277e1
STD_100ppb	Standard	2.47e7	3.709e5	5.87	67.6	100.0000	1.186941e2
STD_200ppb	Standard	N/A	3.446e5	N/A	N/A	200.0000	N/A
STD_500ppb	Standard	N/A	2.675e5	N/A	N/A	500.0000	N/A
V2.0_MW_RQC1_20240724	Quality Control	2.53e3	1.104e5	5.93	6.7	0.0000	4.151816e-2
Blank	Unknown	N/A	3.683e3	N/A	N/A	N/A	N/A
V3.0_MWMS_20240725_1	Unknown	1.57e6	5.371e5	5.84	64.3	N/A	5.223200e0
MWXS243064a_R1	Quality Control	N/A	2.172e5	N/A	N/A	0.0000	N/A
MWXS243064a_R2	Quality Control	N/A	2.207e5	N/A	N/A	0.0000	N/A
MWXS243064a_R3	Quality Control	N/A	2.224e5	N/A	N/A	0.0000	N/A
T24186682b_a	Unknown	N/A	2.103e5	N/A	N/A	N/A	N/A
T24186682b_b	Unknown	N/A	1.670e5	N/A	N/A	N/A	N/A
T24186682b_c	Unknown	N/A	1.830e5	N/A	N/A	N/A	N/A
T24186683b_a	Unknown	N/A	2.452e5	N/A	N/A	N/A	N/A
T24186683b_b	Unknown	N/A	2.437e5	N/A	N/A	N/A	N/A
T24186683b_c	Unknown	N/A	2.504e5	N/A	N/A	N/A	N/A
T24186684b_a	Unknown	N/A	2.130e5	N/A	N/A	N/A	N/A
T24186684b_b	Unknown	N/A	2.404e5	N/A	N/A	N/A	N/A
T24186684b_c	Unknown	N/A	2.153e5	N/A	N/A	N/A	N/A

Compound name: IAA-Phe
Regression Equation: y = 0.56030 x + -3.46678e-4 (r = 0.99303) (weighting: 1 / x^2)

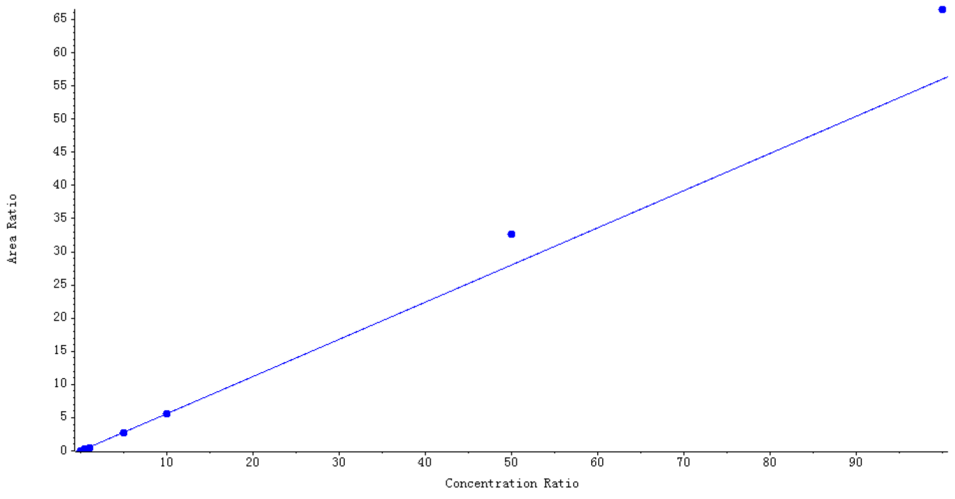

Peak Review

Blank

IAA-Phe AREA:N/A S/N:N/A

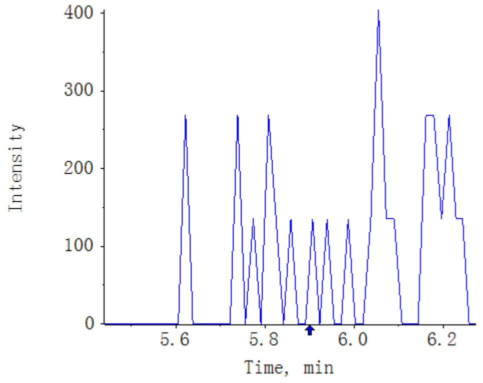

V3.0_MWMS_20240725_1

IAA-Phe AREA:1.57e6 S/N:64.3

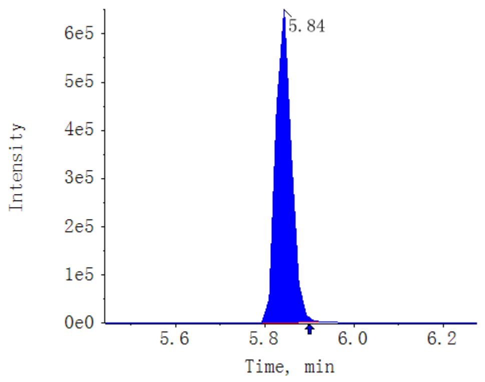

T24186682b_a

IAA-Phe AREA:N/A S/N:N/A

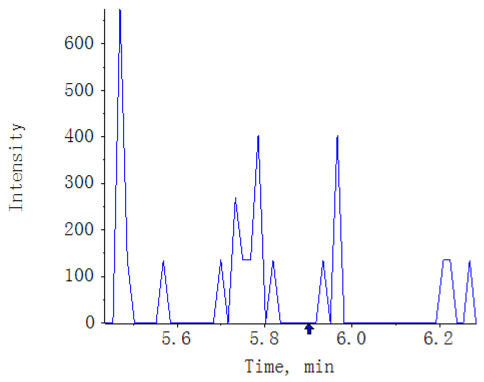

T24186682b_b

IAA-Phe AREA:N/A S/N:N/A

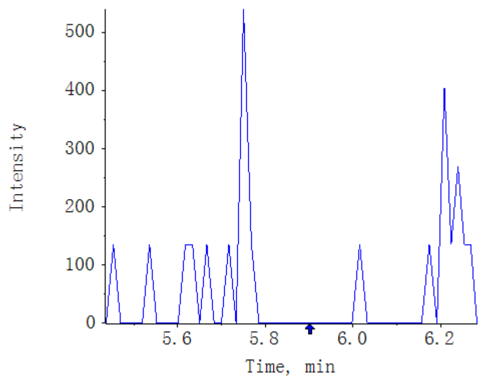

T24186682b_c

IAA-Phe AREA:N/A S/N:N/A

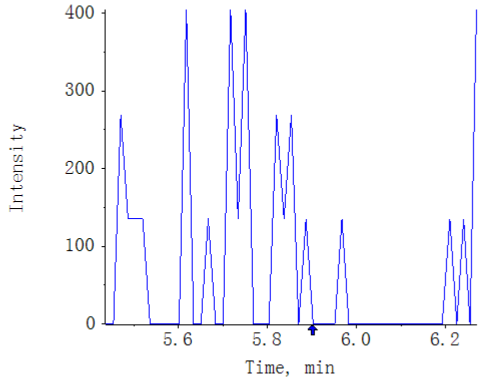

T24186683b_a

IAA-Phe AREA:N/A S/N:N/A

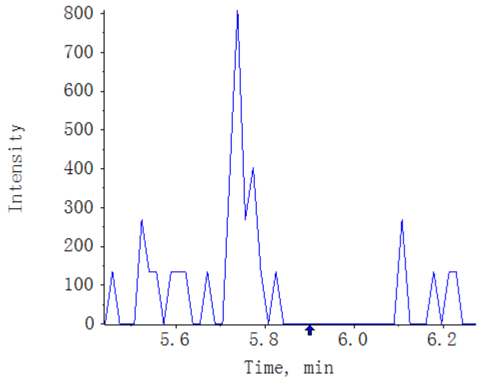

T24186683b_b

IAA-Phe AREA:N/A S/N:N/A

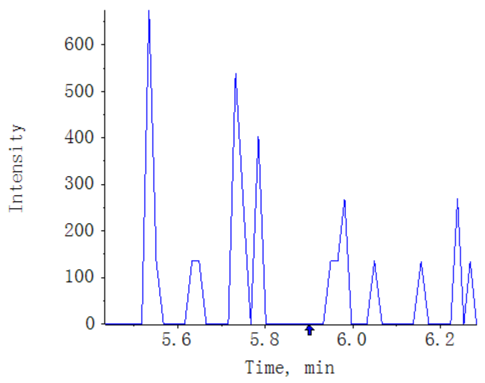

T24186683b_c

IAA-Phe AREA:N/A S/N:N/A

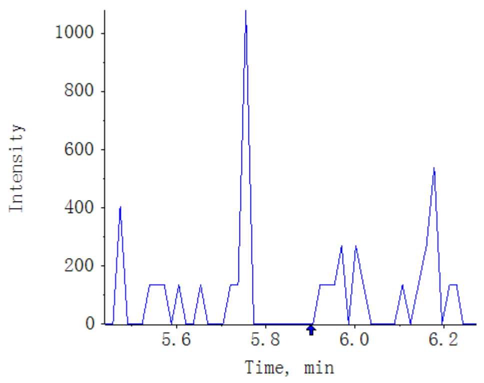

T24186684b_a

IAA-Phe AREA:N/A S/N:N/A

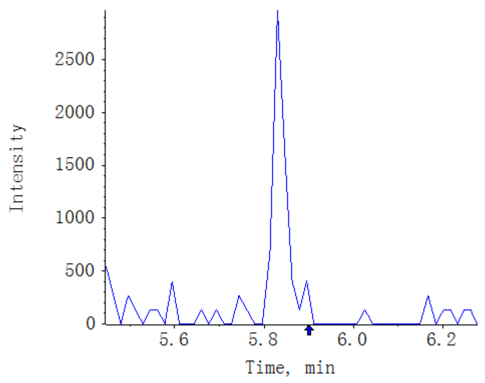

T24186684b_b

IAA-Phe AREA:N/A S/N:N/A

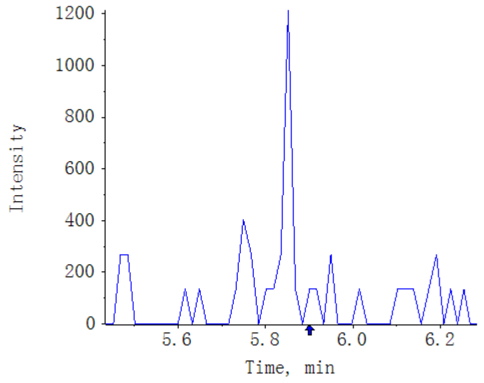

T24186684b_c

IAA-Phe AREA:N/A S/N:N/A

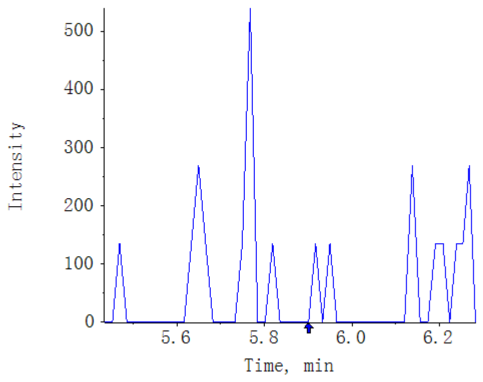

Result Table	MWXS-24-3064-a_9_WH6500-17_A20-3_V6.0_WSS_20240730	Algorithm Used	MQ4
Acquisition Method	ACC-PHs_V6.0_WH6500-17_CMY_20240521.dam	Instrument Name	Triple Quad 6500+ Low Mass
Project	N/A	Analytes QTY	109:82

Compound name: ICA (160.0 / 116.1)

Sample Name	Sample Type	Area (cps)	Is Area (cps)	RT (min)	S/N	Target Conc	Calculated Conc.()
STD_0.01ppb	Standard	N/A	1.235e7	N/A	N/A	0.0100	N/A
STD_0.05ppb	Standard	N/A	1.260e7	N/A	N/A	0.0500	N/A
STD_0.1ppb	Standard	1.02e4	1.243e7	4.78	7.9	0.1000	9.887568e-2
STD_0.5ppb	Standard	4.43e4	1.090e7	4.77	16.2	0.5000	5.752471e-1
STD_1ppb	Standard	6.71e4	1.166e7	4.77	24.9	1.0000	8.239146e-1
STD_5ppb	Standard	3.93e5	1.172e7	4.79	42.6	5.0000	4.903181e0
STD_10ppb	Standard	6.89e5	1.121e7	4.77	45.6	10.0000	9.013861e0
STD_50ppb	Standard	4.15e6	1.170e7	4.77	68.7	50.0000	5.207647e1
STD_100ppb	Standard	7.41e6	1.119e7	4.78	52.1	100.0000	9.731387e1
STD_200ppb	Standard	1.62e7	1.041e7	4.77	79.3	200.0000	2.280288e2
STD_500ppb	Standard	N/A	9.535e6	N/A	N/A	500.0000	N/A
V2.0_MW_RQC1_20240724	Quality Control	1.07e5	1.495e6	4.75	36.9	0.0000	1.050675e1
Blank	Unknown	N/A	1.791e4	N/A	N/A	N/A	N/A
V3.0_MWMS_20240725_1	Unknown	5.26e5	1.171e7	4.76	53.7	N/A	6.585121e0
MWXS243064a_R1	Quality Control	N/A	3.677e6	N/A	N/A	0.0000	N/A
MWXS243064a_R2	Quality Control	N/A	3.585e6	N/A	N/A	0.0000	N/A
MWXS243064a_R3	Quality Control	N/A	3.593e6	N/A	N/A	0.0000	N/A
T24186682b_a	Unknown	N/A	3.191e6	N/A	N/A	N/A	N/A
T24186682b_b	Unknown	N/A	3.006e6	N/A	N/A	N/A	N/A
T24186682b_c	Unknown	N/A	3.032e6	N/A	N/A	N/A	N/A
T24186683b_a	Unknown	N/A	3.634e6	N/A	N/A	N/A	N/A
T24186683b_b	Unknown	N/A	3.896e6	N/A	N/A	N/A	N/A
T24186683b_c	Unknown	N/A	3.346e6	N/A	N/A	N/A	N/A
T24186684b_a	Unknown	N/A	4.404e6	N/A	N/A	N/A	N/A
T24186684b_b	Unknown	N/A	4.701e6	N/A	N/A	N/A	N/A
T24186684b_c	Unknown	N/A	4.822e6	N/A	N/A	N/A	N/A

Compound name: ICA
Regression Equation: y = 0.00680 x + 1.46429e-4 (r = 0.99325) (weighting: 1 / x^2)

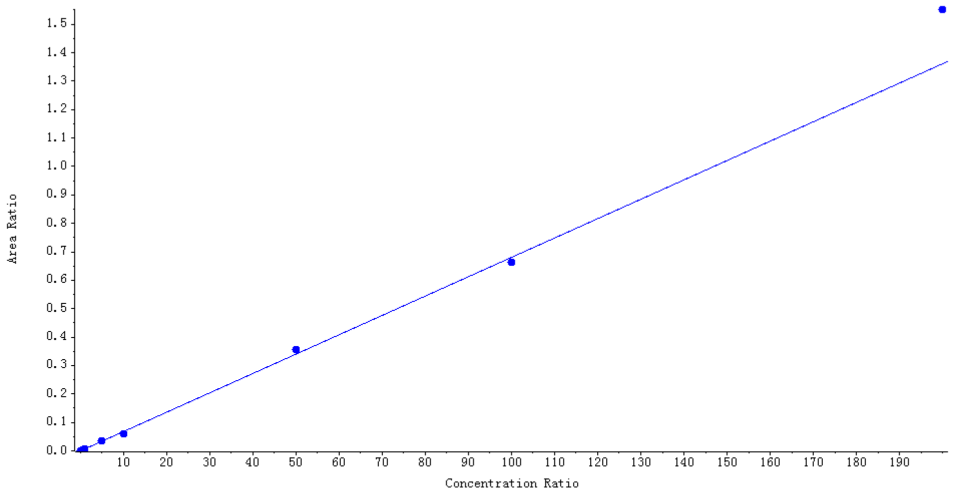

Peak Review

Blank

ICA AREA:N/A S/N:N/A

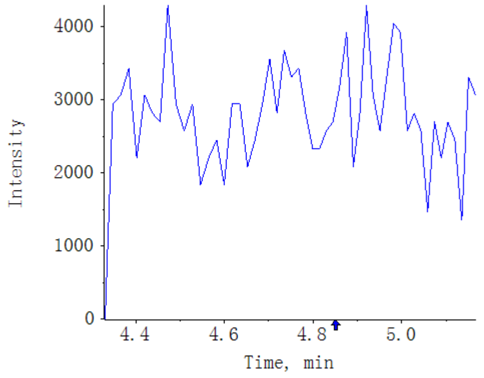

V3.0_MWMS_20240725_1

ICA AREA:5.26e5 S/N:53.7

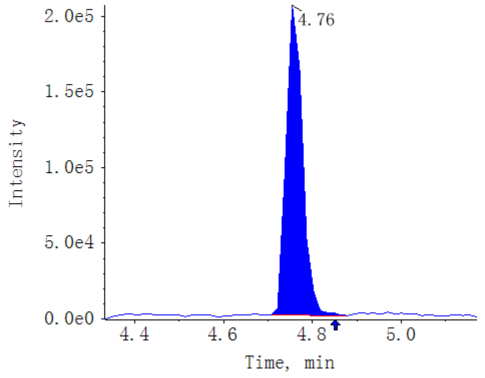

T24186682b_a

ICA AREA:N/A S/N:N/A

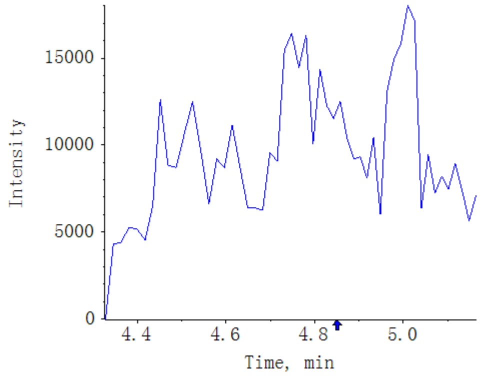

T24186682b_b

ICA AREA:N/A S/N:N/A

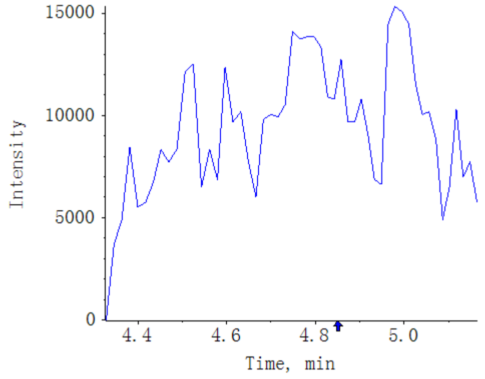

T24186682b_c

ICA AREA:N/A S/N:N/A

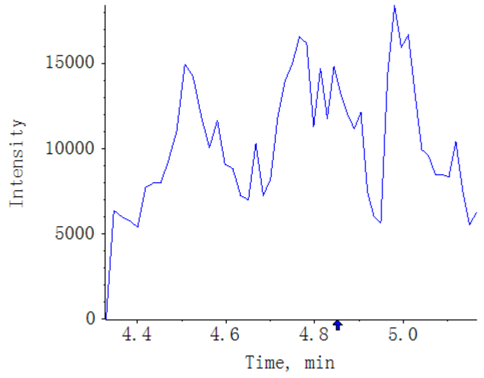

T24186683b_a

ICA AREA:N/A S/N:N/A

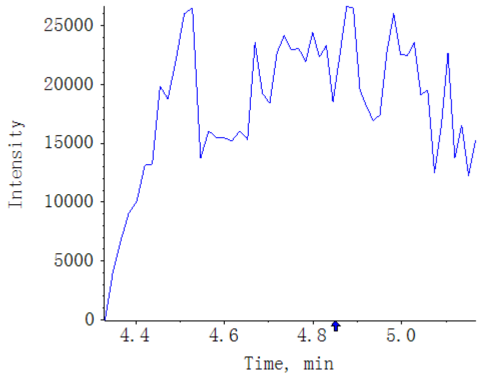

T24186683b_b

ICA AREA:N/A S/N:N/A

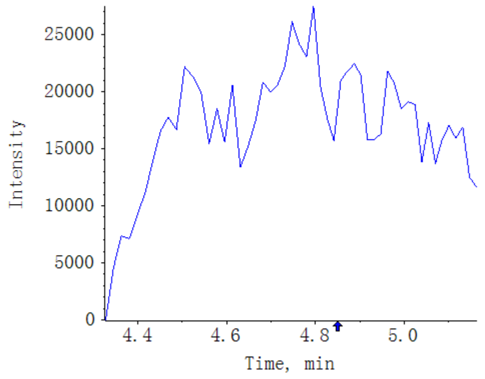

T24186683b_c

ICA AREA:N/A S/N:N/A

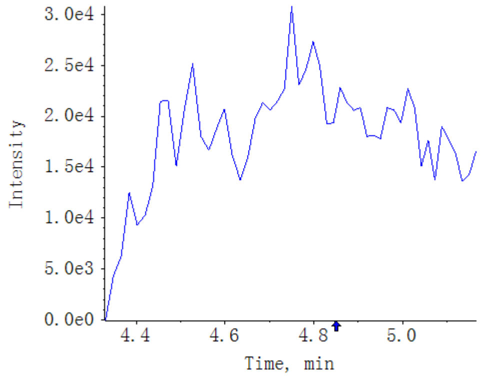

T24186684b_a

ICA AREA:N/A S/N:N/A

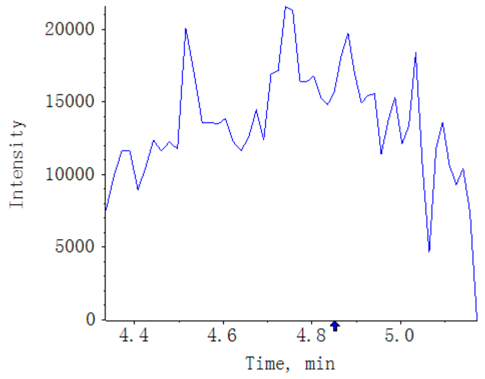

T24186684b_b

ICA AREA:N/A S/N:N/A

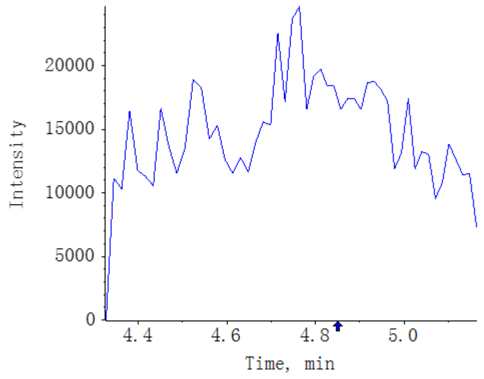

T24186684b_c

ICA AREA:N/A S/N:N/A

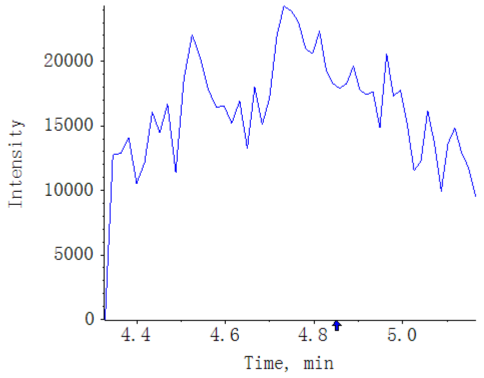

Result Table	MWXS-24-3064-a_9_WH6500-17_A20-3_V6.0_WSS_20240730	Algorithm Used	MQ4
Acquisition Method	ACC-PHs_V6.0_WH6500-17_CMY_20240521.dam	Instrument Name	Triple Quad 6500+ Low Mass
Project	N/A	Analytes QTY	109:83

Compound name: GA1 (347.0 / 259.0)

Sample Name	Sample Type	Area (cps)	Is Area (cps)	RT (min)	S/N	Target Conc	Calculated Conc.()
STD_0.01ppb	Standard	N/A	3.538e5	N/A	N/A	0.0100	N/A
STD_0.05ppb	Standard	N/A	3.397e5	N/A	N/A	0.0500	N/A
STD_0.1ppb	Standard	N/A	3.587e5	N/A	N/A	0.1000	N/A
STD_0.5ppb	Standard	6.07e3	3.275e5	4.50	5.8	0.5000	5.347708e-1
STD_1ppb	Standard	1.06e4	3.746e5	4.50	6.2	1.0000	8.363583e-1
STD_5ppb	Standard	5.55e4	3.209e5	4.51	19.3	5.0000	5.353633e0
STD_10ppb	Standard	1.13e5	3.165e5	4.49	30.3	10.0000	1.107829e1
STD_50ppb	Standard	5.90e5	3.582e5	4.49	43.3	50.0000	5.134689e1
STD_100ppb	Standard	1.08e6	3.626e5	4.50	35.9	100.0000	9.296893e1
STD_200ppb	Standard	2.28e6	3.701e5	4.50	43.7	200.0000	1.917835e2
STD_500ppb	Standard	N/A	4.777e5	N/A	N/A	500.0000	N/A
V2.0_MW_RQC1_20240724	Quality Control	N/A	1.299e5	N/A	N/A	0.0000	N/A
Blank	Unknown	N/A	5.220e2	N/A	N/A	N/A	N/A
V3.0_MWMS_20240725_1	Unknown	6.89e5	3.708e5	4.49	5.1	N/A	5.796418e1
MWXS243064a_R1	Quality Control	1.95e4	1.034e5	4.48	5.1	0.0000	5.855960e0
MWXS243064a_R2	Quality Control	1.45e4	1.066e5	4.49	5.3	0.0000	4.195696e0
MWXS243064a_R3	Quality Control	1.56e4	1.022e5	4.47	5.0	0.0000	4.719421e0
T24186682b_a	Unknown	N/A	8.442e4	N/A	N/A	N/A	N/A
T24186682b_b	Unknown	N/A	7.738e4	N/A	N/A	N/A	N/A
T24186682b_c	Unknown	N/A	9.288e4	N/A	N/A	N/A	N/A
T24186683b_a	Unknown	1.66e4	1.648e5	4.48	5.0	N/A	3.091343e0
T24186683b_b	Unknown	1.68e4	1.807e5	4.48	4.4	N/A	2.865208e0
T24186683b_c	Unknown	1.91e4	1.771e5	4.48	6.6	N/A	3.327064e0
T24186684b_a	Unknown	4.55e4	9.244e4	4.49	12.0	N/A	1.530411e1
T24186684b_b	Unknown	5.24e4	1.062e5	4.50	14.3	N/A	1.533954e1
T24186684b_c	Unknown	3.88e4	1.130e5	4.49	12.6	N/A	1.065917e1

Compound name: GA1
Regression Equation: y = 0.03205 x + 0.00140 (r = 0.99426) (weighting: 1 / x^2)

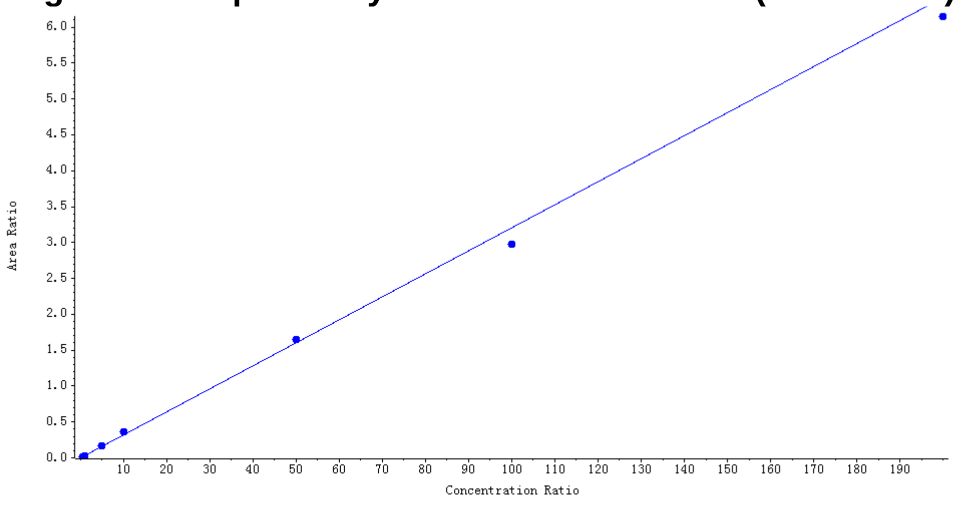

Peak Review

Blank
GA1 AREA:N/A S/N:N/A

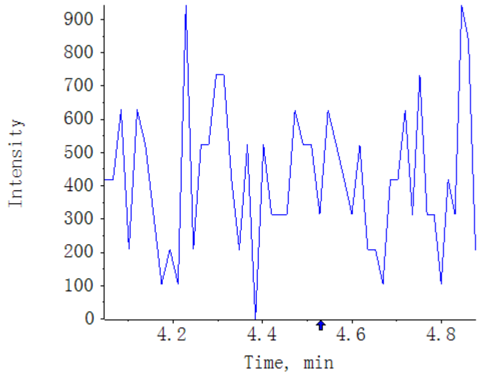

V3.0_MWMS_20240725_1
GA1 AREA:6.89e5 S/N:5.1

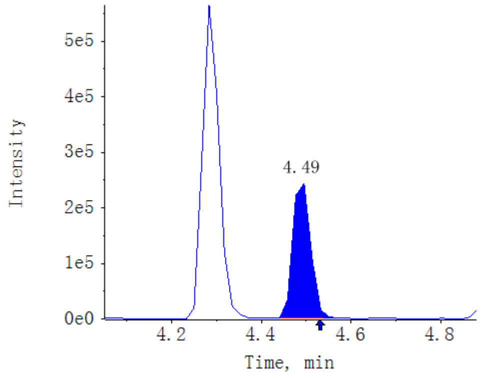

T24186682b_a
GA1 AREA:N/A S/N:N/A

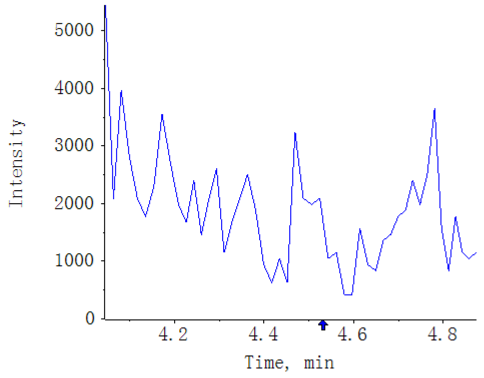

T24186682b_b
GA1 AREA:N/A S/N:N/A

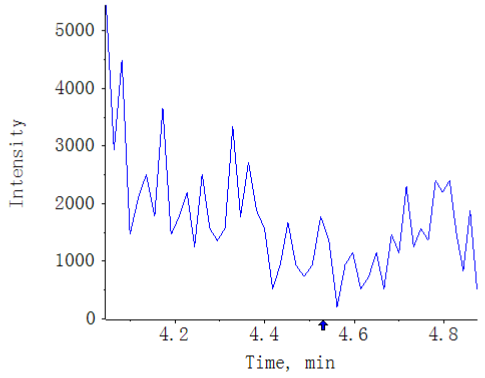

T24186682b_c
GA1 AREA:N/A S/N:N/A

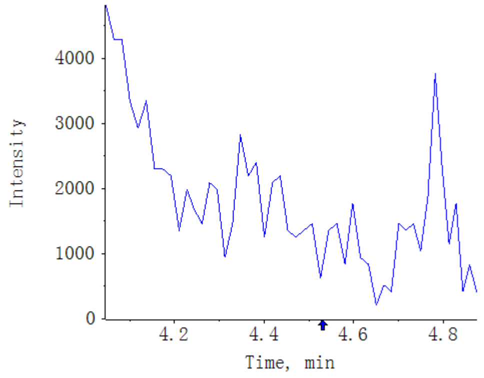

T24186683b_a
GA1 AREA:1.66e4 S/N:5.0

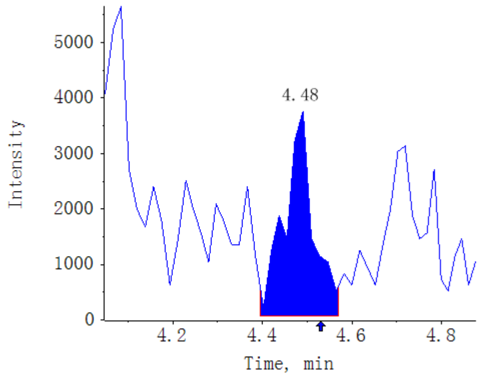

T24186683b_b
GA1 AREA:1.68e4 S/N:4.4

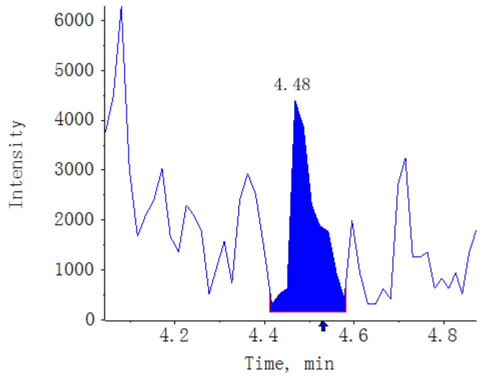

T24186683b_c
GA1 AREA:1.91e4 S/N:6.6

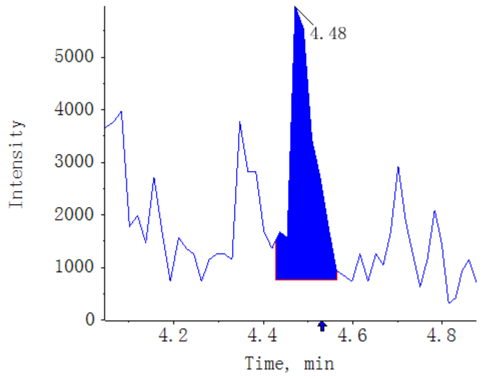

T24186684b_a
GA1 AREA:4.55e4 S/N:12.0

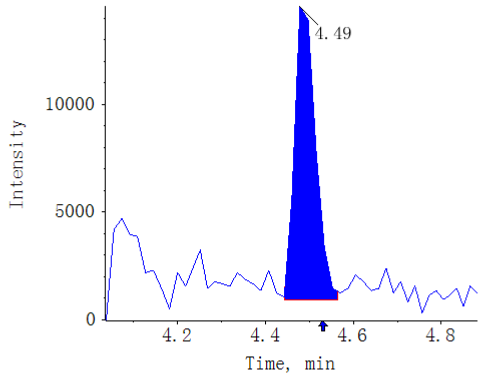

T24186684b_b
GA1 AREA:5.24e4 S/N:14.3

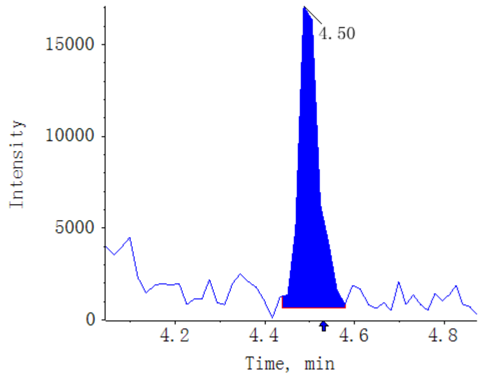

T24186684b_c
GA1 AREA:3.88e4 S/N:12.6

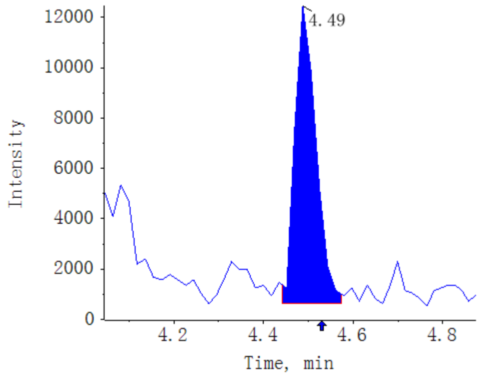

Result Table	MWXS-24-3064-a_9_WH6500-17_A20-3_V6.0_WSS_20240730	Algorithm Used	MQ4
Acquisition Method	ACC-PHs_V6.0_WH6500-17_CMY_20240521.dam	Instrument Name	Triple Quad 6500+ Low Mass
Project	N/A	Analytes QTY	109:84

Compound name: GA15 (329.1 / 131.0)

Sample Name	Sample Type	Area (cps)	Is Area (cps)	RT (min)	S/N	Target Conc	Calculated Conc.()
STD_0.01ppb	Standard	N/A	3.538e5	N/A	N/A	0.0100	N/A
STD_0.05ppb	Standard	N/A	3.397e5	N/A	N/A	0.0500	N/A
STD_0.1ppb	Standard	N/A	3.587e5	N/A	N/A	0.1000	N/A
STD_0.5ppb	Standard	5.67e3	3.275e5	6.87	20.4	0.5000	5.344209e-1
STD_1ppb	Standard	9.91e3	3.746e5	6.88	30.4	1.0000	8.284467e-1
STD_5ppb	Standard	5.72e4	3.209e5	6.89	52.5	5.0000	5.714549e0
STD_10ppb	Standard	1.04e5	3.165e5	6.88	66.7	10.0000	1.054199e1
STD_50ppb	Standard	5.82e5	3.582e5	6.88	68.0	50.0000	5.223997e1
STD_100ppb	Standard	1.05e6	3.626e5	6.88	61.4	100.0000	9.336034e1
STD_200ppb	Standard	2.13e6	3.701e5	6.88	73.9	200.0000	1.854400e2
STD_500ppb	Standard	N/A	4.777e5	N/A	N/A	500.0000	N/A
V2.0_MW_RQC1_20240724	Quality Control	N/A	1.299e5	N/A	N/A	0.0000	N/A
Blank	Unknown	N/A	5.220e2	N/A	N/A	N/A	N/A
V3.0_MWMS_20240725_1	Unknown	4.80e5	3.708e5	6.86	73.2	N/A	4.163497e1
MWXS243064a_R1	Quality Control	5.41e4	1.034e5	6.85	74.5	0.0000	1.682856e1
MWXS243064a_R2	Quality Control	5.58e4	1.066e5	6.86	61.1	0.0000	1.683362e1
MWXS243064a_R3	Quality Control	4.56e4	1.022e5	6.84	61.6	0.0000	1.435183e1
T24186682b_a	Unknown	3.39e4	8.442e4	6.85	51.1	N/A	1.289456e1
T24186682b_b	Unknown	3.34e4	7.738e4	6.85	57.1	N/A	1.388184e1
T24186682b_c	Unknown	3.24e4	9.288e4	6.85	59.3	N/A	1.121308e1
T24186683b_a	Unknown	4.40e4	1.648e5	6.85	59.3	N/A	8.562888e0
T24186683b_b	Unknown	4.31e4	1.807e5	6.85	52.2	N/A	7.658683e0
T24186683b_c	Unknown	3.66e4	1.771e5	6.86	58.5	N/A	6.632774e0
T24186684b_a	Unknown	8.15e4	9.244e4	6.85	72.5	N/A	2.835233e1
T24186684b_b	Unknown	7.57e4	1.062e5	6.86	62.3	N/A	2.291284e1
T24186684b_c	Unknown	7.54e4	1.130e5	6.86	69.9	N/A	2.144351e1

Compound name: GA15
Regression Equation: y = 0.03108 x + 7.16329e-4 (r = 0.99286) (weighting: 1 / x^2)

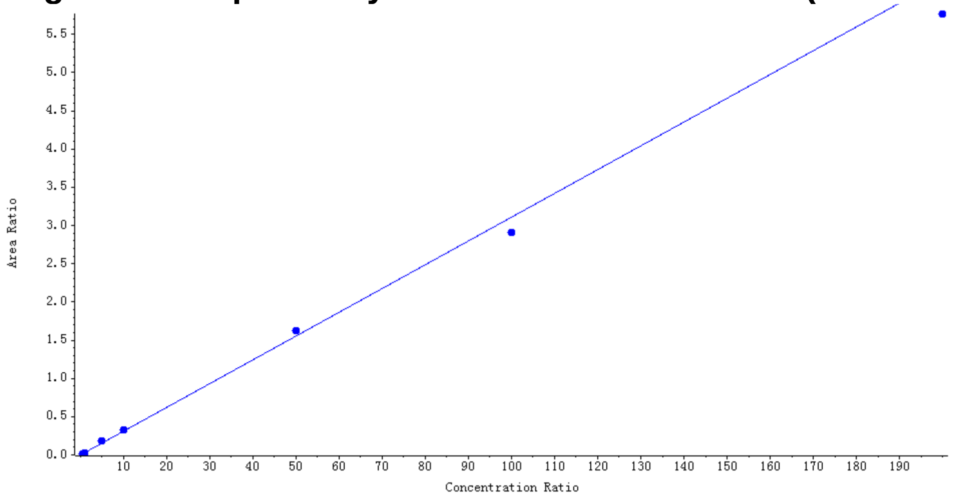

Peak Review

Blank
GA15 AREA:N/A S/N:N/A

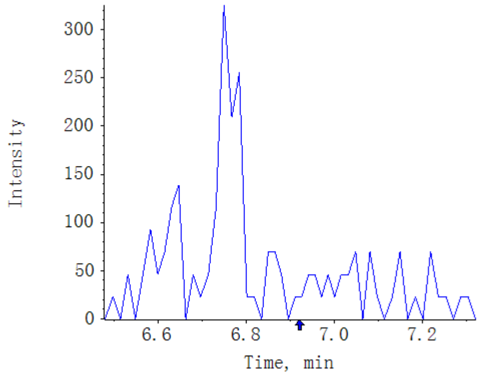

V3.0_MWMS_20240725_1
GA15 AREA:4.80e5 S/N:73.2

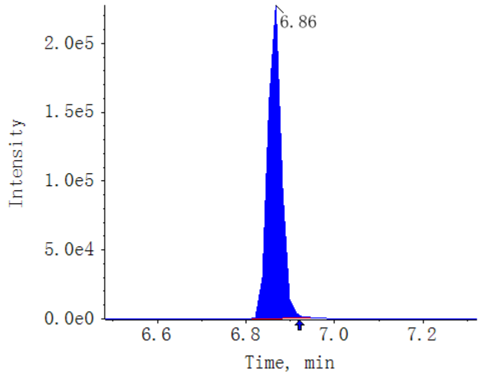

T24186682b_a
GA15 AREA:3.39e4 S/N:51.1

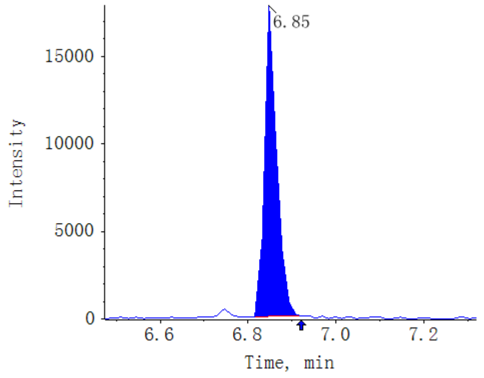

T24186682b_b
GA15 AREA:3.34e4 S/N:57.1

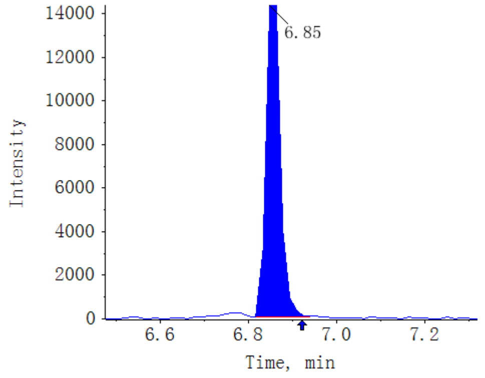

T24186682b_c
GA15 AREA:3.24e4 S/N:59.3

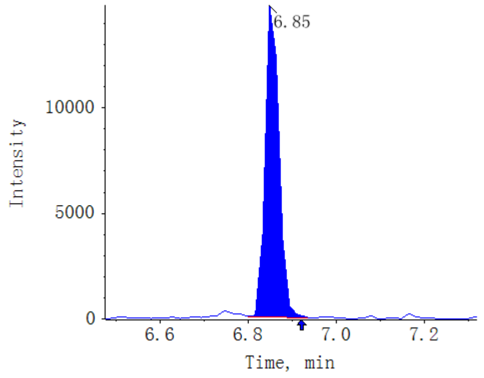

T24186683b_a
GA15 AREA:4.40e4 S/N:59.3

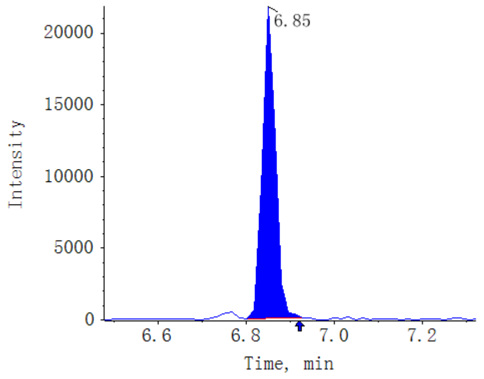

T24186683b_b
GA15 AREA:4.31e4 S/N:52.2

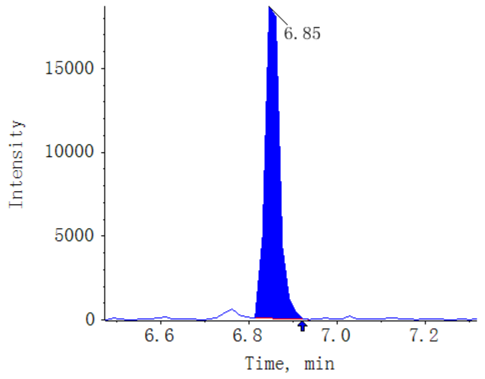

T24186683b_c
GA15 AREA:3.66e4 S/N:58.5

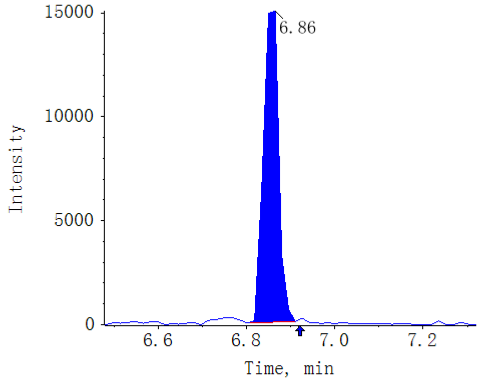

T24186684b_a
GA15 AREA:8.15e4 S/N:72.5

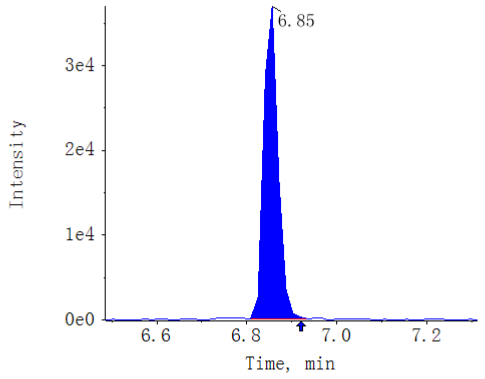

T24186684b_b
GA15 AREA:7.57e4 S/N:62.3

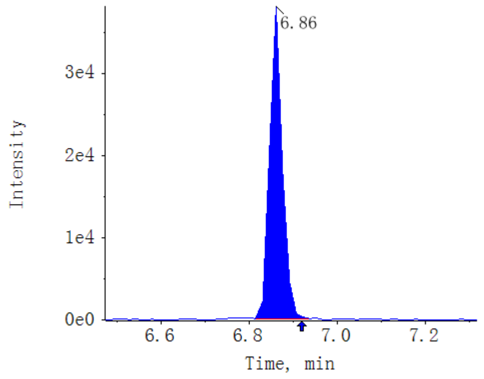

T24186684b_c
GA15 AREA:7.54e4 S/N:69.9

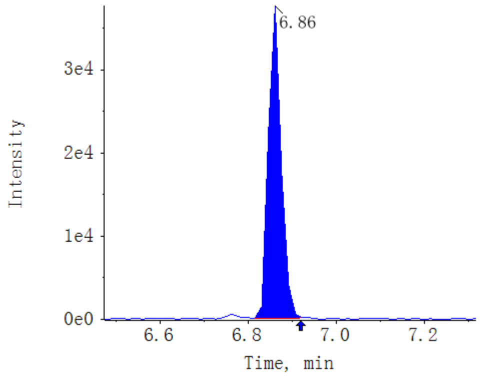

Result Table	MWXS-24-3064-a_9_WH6500-17_A20-3_V6.0_WSS_20240730	Algorithm Used	MQ4
Acquisition Method	ACC-PHs_V6.0_WH6500-17_CMY_20240521.dam	Instrument Name	Triple Quad 6500+ Low Mass
Project	N/A	Analytes QTY	109:85

Compound name: GA19 (361.2 / 273.2)

Sample Name	Sample Type	Area (cps)	Is Area (cps)	RT (min)	S/N	Target Conc	Calculated Conc.()
STD_0.01ppb	Standard	N/A	5.588e5	N/A	N/A	0.0100	N/A
STD_0.05ppb	Standard	N/A	5.433e5	N/A	N/A	0.0500	N/A
STD_0.1ppb	Standard	3.38e3	5.311e5	5.19	8.6	0.1000	1.044319e-1
STD_0.5ppb	Standard	7.24e3	5.473e5	5.18	13.5	0.5000	3.936478e-1
STD_1ppb	Standard	1.53e4	5.693e5	5.19	24.9	1.0000	9.666228e-1
STD_5ppb	Standard	7.04e4	5.361e5	5.20	42.7	5.0000	5.364494e0
STD_10ppb	Standard	1.28e5	5.314e5	5.19	50.4	10.0000	9.946402e0
STD_50ppb	Standard	6.89e5	5.532e5	5.18	57.8	50.0000	5.226965e1
STD_100ppb	Standard	1.31e6	5.266e5	5.19	63.7	100.0000	1.048653e2
STD_200ppb	Standard	2.66e6	5.463e5	5.19	47.7	200.0000	2.050440e2
STD_500ppb	Standard	6.69e6	5.550e5	5.19	45.1	500.0000	5.074787e2
V2.0_MW_RQC1_20240724	Quality Control	1.90e4	3.046e5	5.17	12.0	0.0000	2.461125e0
Blank	Unknown	N/A	4.720e2	N/A	N/A	N/A	N/A
V3.0_MWMS_20240725_1	Unknown	5.96e5	5.723e5	5.17	55.9	N/A	4.367900e1
MWXS243064a_R1	Quality Control	N/A	4.360e5	N/A	N/A	0.0000	N/A
MWXS243064a_R2	Quality Control	N/A	4.352e5	N/A	N/A	0.0000	N/A
MWXS243064a_R3	Quality Control	N/A	4.171e5	N/A	N/A	0.0000	N/A
T24186682b_a	Unknown	N/A	4.334e5	N/A	N/A	N/A	N/A
T24186682b_b	Unknown	N/A	3.891e5	N/A	N/A	N/A	N/A
T24186682b_c	Unknown	N/A	4.163e5	N/A	N/A	N/A	N/A
T24186683b_a	Unknown	N/A	4.886e5	N/A	N/A	N/A	N/A
T24186683b_b	Unknown	N/A	4.862e5	N/A	N/A	N/A	N/A
T24186683b_c	Unknown	N/A	4.836e5	N/A	N/A	N/A	N/A
T24186684b_a	Unknown	N/A	3.790e5	N/A	N/A	N/A	N/A
T24186684b_b	Unknown	N/A	3.903e5	N/A	N/A	N/A	N/A
T24186684b_c	Unknown	N/A	4.229e5	N/A	N/A	N/A	N/A

Compound name: GA19
Regression Equation: y = 0.02376 x + 0.00389 (r = 0.99599) (weighting: 1 / x^2)

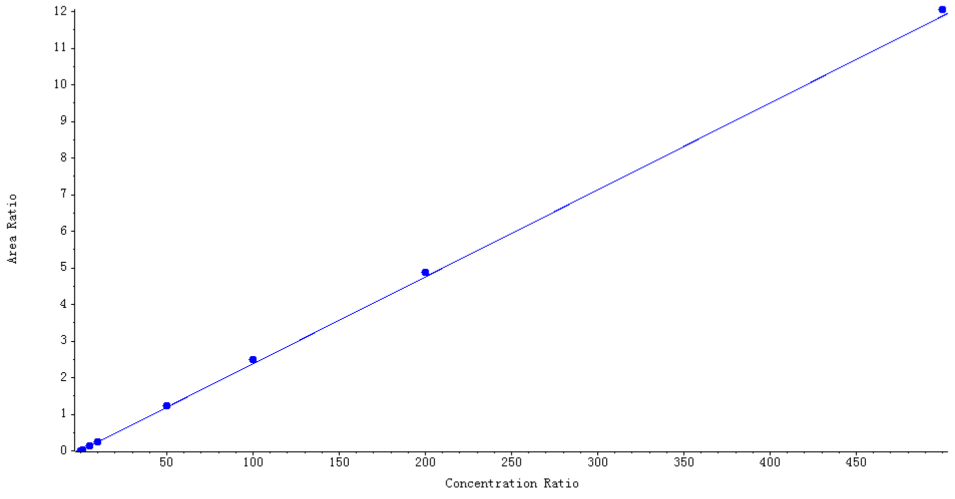

Peak Review

Blank
GA19 AREA:N/A S/N:N/A

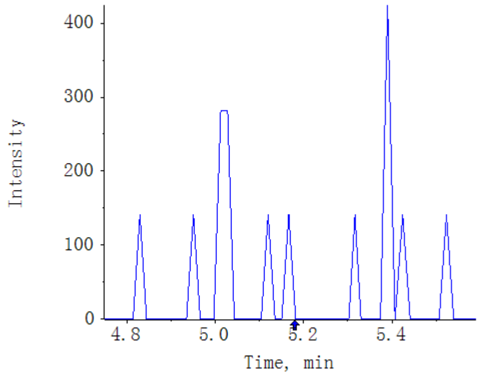

V3.0_MWMS_20240725_1
GA19 AREA:5.96e5 S/N:55.9

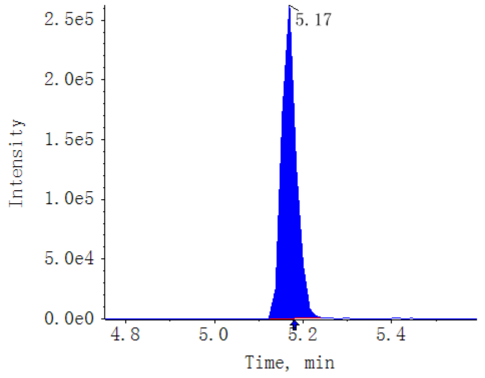

T24186682b_a
GA19 AREA:N/A S/N:N/A

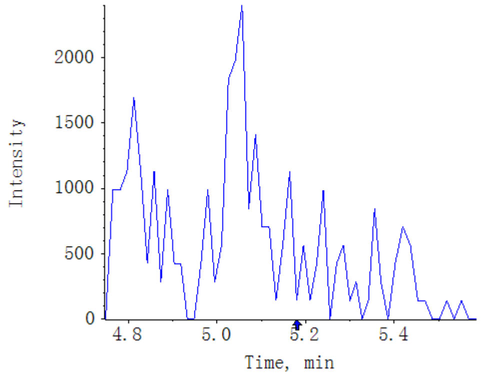

T24186682b_b
GA19 AREA:N/A S/N:N/A

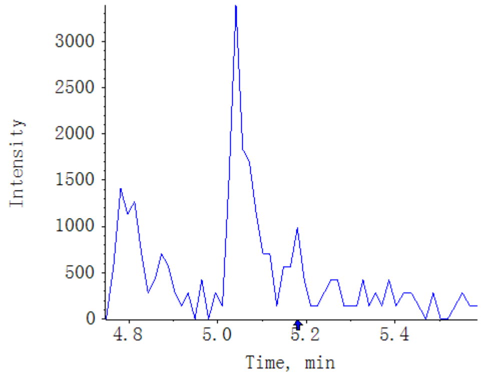

T24186682b_c
GA19 AREA:N/A S/N:N/A

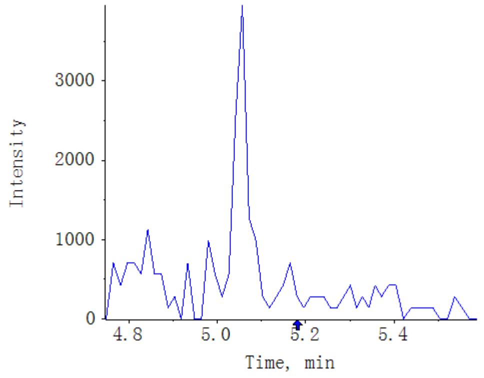

T24186683b_a
GA19 AREA:N/A S/N:N/A

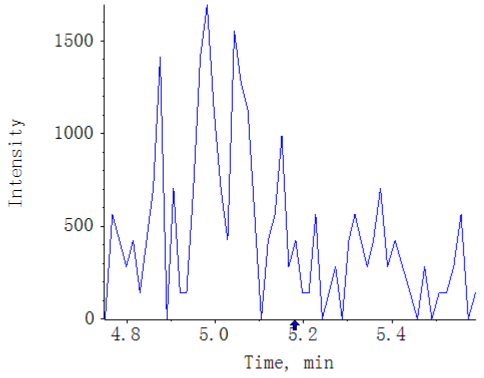

T24186683b_b
GA19 AREA:N/A S/N:N/A

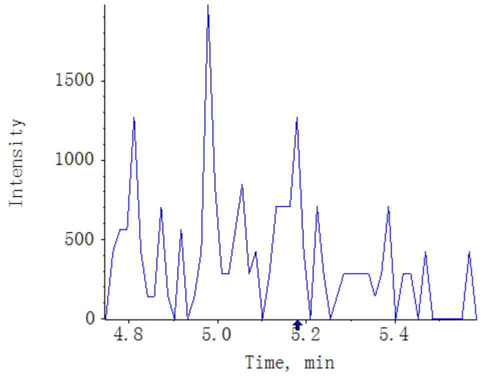

T24186683b_c
GA19 AREA:N/A S/N:N/A

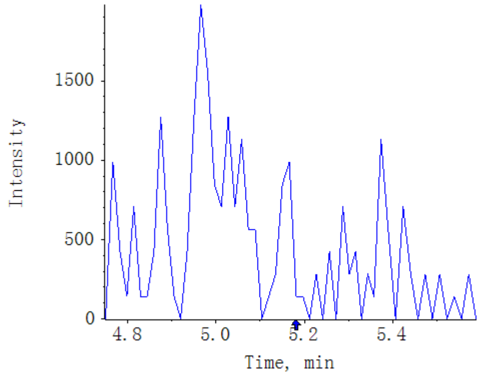

T24186684b_a
GA19 AREA:N/A S/N:N/A

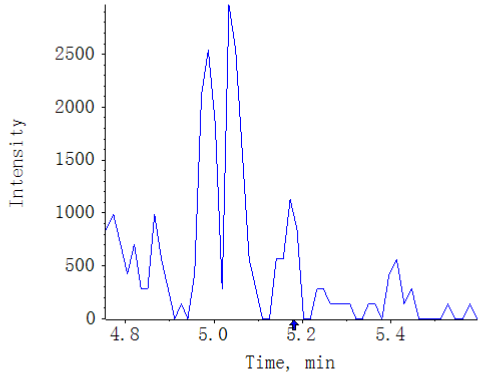

T24186684b_b
GA19 AREA:N/A S/N:N/A

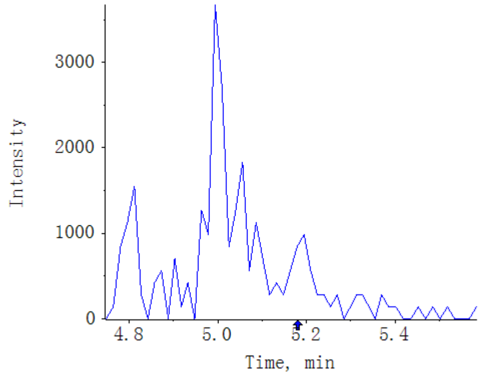

T24186684b_c
GA19 AREA:N/A S/N:N/A

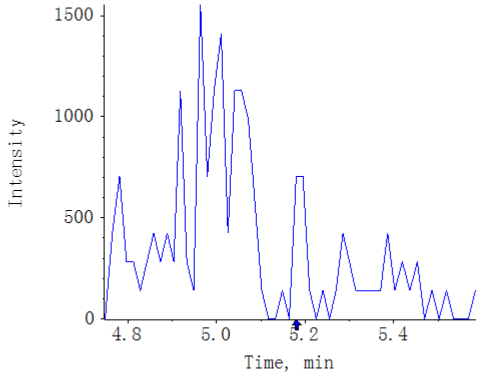

Result Table	MWXS-24-3064-a_9_WH6500-17_A20-3_V6.0_WSS_20240730	Algorithm Used	MQ4
Acquisition Method	ACC-PHs_V6.0_WH6500-17_CMY_20240521.dam	Instrument Name	Triple Quad 6500+ Low Mass
Project	N/A	Analytes QTY	109:86

Compound name: GA20 (331.3 / 287.0)

Sample Name	Sample Type	Area (cps)	Is Area (cps)	RT (min)	S/N	Target Conc	Calculated Conc.()
STD_0.01ppb	Standard	N/A	1.468e6	N/A	N/A	0.0100	N/A
STD_0.05ppb	Standard	N/A	1.535e6	N/A	N/A	0.0500	N/A
STD_0.1ppb	Standard	5.10e3	1.516e6	5.38	5.6	0.1000	8.155950e-2
STD_0.5ppb	Standard	1.41e4	1.366e6	5.37	16.3	0.5000	4.830423e-1
STD_1ppb	Standard	2.36e4	1.409e6	5.37	20.7	1.0000	8.529789e-1
STD_5ppb	Standard	1.38e5	1.392e6	5.38	22.8	5.0000	5.624280e0
STD_10ppb	Standard	2.43e5	1.333e6	5.37	22.1	10.0000	1.040493e1
STD_50ppb	Standard	1.31e6	1.321e6	5.37	24.1	50.0000	5.709042e1
STD_100ppb	Standard	2.41e6	1.314e6	5.38	21.1	100.0000	1.056905e2
STD_200ppb	Standard	4.59e6	1.263e6	5.37	27.1	200.0000	2.095117e2
STD_500ppb	Standard	9.03e6	1.093e6	5.37	19.7	500.0000	4.768606e2
V2.0_MW_RQC1_20240724	Quality Control	8.16e4	4.832e5	5.29	7.6	0.0000	9.635136e0
Blank	Unknown	N/A	8.350e2	N/A	N/A	N/A	N/A
V3.0_MWMS_20240725_1	Unknown	1.39e6	1.809e6	5.35	15.2	N/A	4.419306e1
MWXS243064a_R1	Quality Control	N/A	8.458e5	N/A	N/A	0.0000	N/A
MWXS243064a_R2	Quality Control	N/A	8.906e5	N/A	N/A	0.0000	N/A
MWXS243064a_R3	Quality Control	N/A	8.093e5	N/A	N/A	0.0000	N/A
T24186682b_a	Unknown	2.16e4	9.467e5	5.43	8.0	N/A	1.205830e0
T24186682b_b	Unknown	1.37e4	7.728e5	5.43	5.6	N/A	9.098541e-1
T24186682b_c	Unknown	1.44e4	8.075e5	5.43	9.5	N/A	9.138669e-1
T24186683b_a	Unknown	N/A	9.485e5	N/A	N/A	N/A	N/A
T24186683b_b	Unknown	N/A	8.888e5	N/A	N/A	N/A	N/A
T24186683b_c	Unknown	N/A	9.185e5	N/A	N/A	N/A	N/A
T24186684b_a	Unknown	N/A	8.781e5	N/A	N/A	N/A	N/A
T24186684b_b	Unknown	N/A	9.928e5	N/A	N/A	N/A	N/A
T24186684b_c	Unknown	N/A	7.991e5	N/A	N/A	N/A	N/A

Compound name: GA20
Regression Equation: y = 0.01733 x + 0.00195 (r = 0.99828) (weighting: 1 / x)

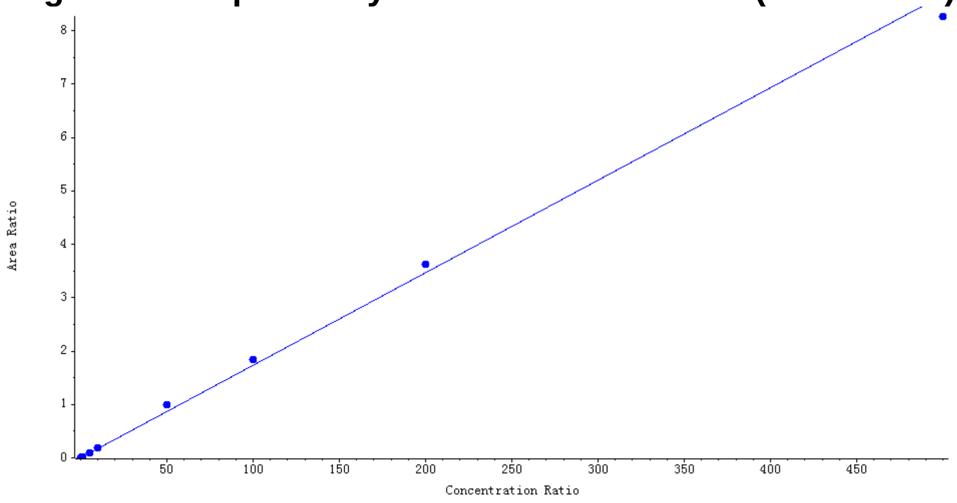

Peak Review

Blank
GA20 AREA:N/A S/N:N/A

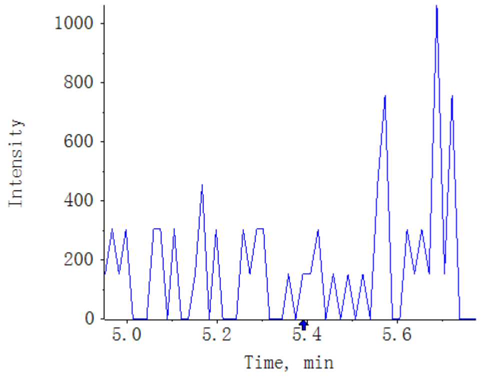

V3.0_MWMS_20240725_1
GA20 AREA:1.39e6 S/N:15.2

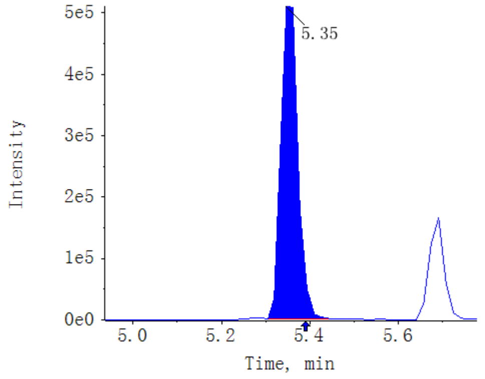

T24186682b_a
GA20 AREA:2.16e4 S/N:8.0

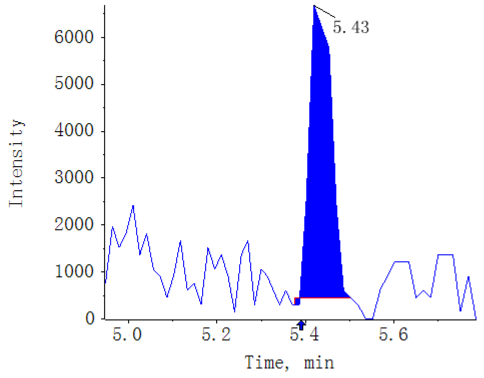

T24186682b_b
GA20 AREA:1.37e4 S/N:5.6

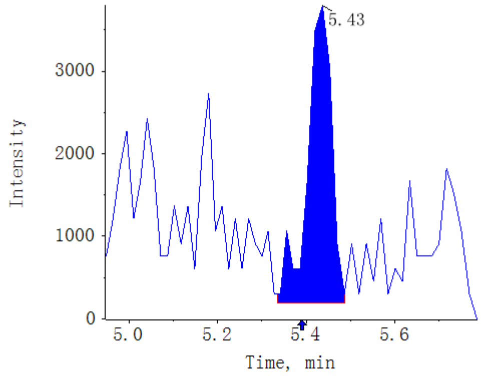

T24186682b_c
GA20 AREA:1.44e4 S/N:9.5

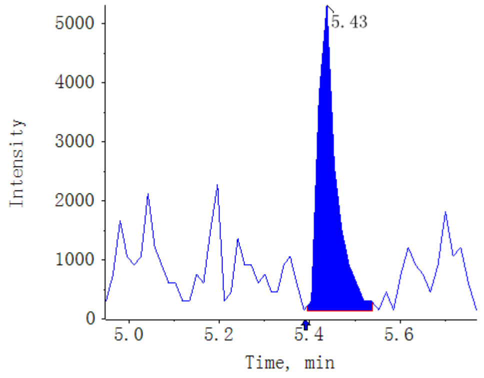

T24186683b_a
GA20 AREA:N/A S/N:N/A

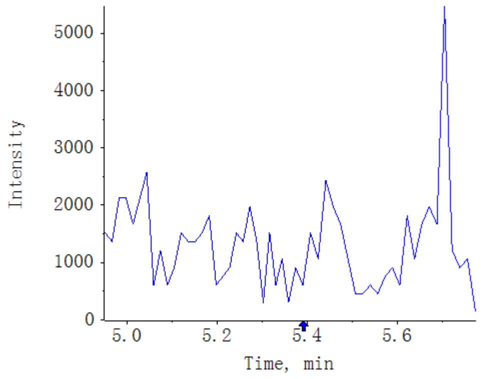

T24186683b_b
GA20 AREA:N/A S/N:N/A

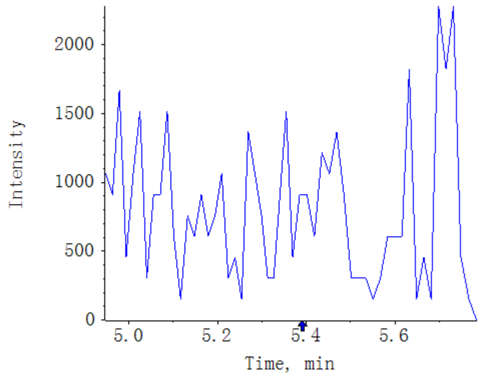

T24186683b_c
GA20 AREA:N/A S/N:N/A

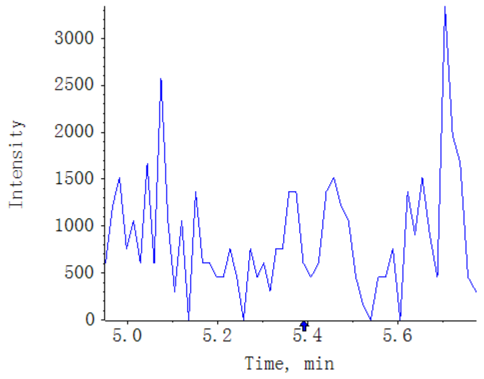

T24186684b_a
GA20 AREA:N/A S/N:N/A

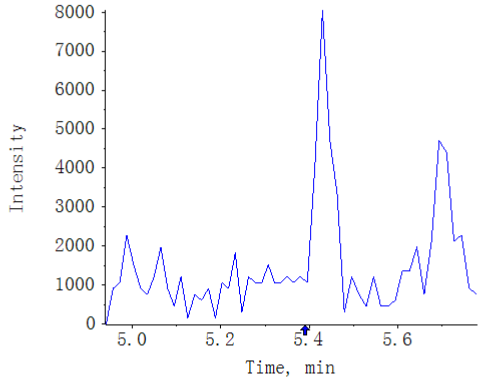

T24186684b_b
GA20 AREA:N/A S/N:N/A

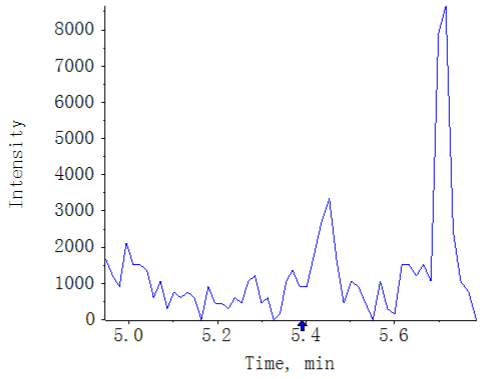

T24186684b_c
GA20 AREA:N/A S/N:N/A

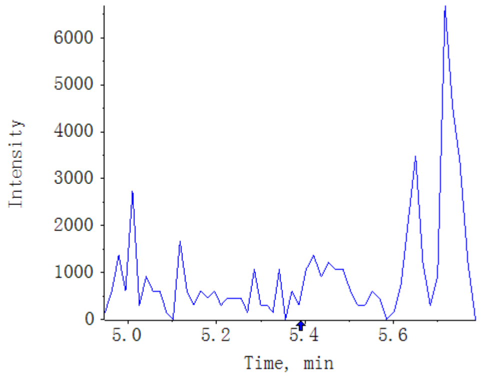

Result Table	MWXS-24-3064-a_9_WH6500-17_A20-3_V6.0_WSS_20240730	Algorithm Used	MQ4
Acquisition Method	ACC-PHs_V6.0_WH6500-17_CMY_20240521.dam	Instrument Name	Triple Quad 6500+ Low Mass
Project	N/A	Analytes QTY	109:87

Compound name: GA24 (345.1 / 257.1)

Sample Name	Sample Type	Area (cps)	Is Area (cps)	RT (min)	S/N	Target Conc	Calculated Conc.()
STD_0.01ppb	Standard	N/A	3.538e5	N/A	N/A	0.0100	N/A
STD_0.05ppb	Standard	N/A	3.397e5	N/A	N/A	0.0500	N/A
STD_0.1ppb	Standard	1.71e3	3.587e5	6.38	5.1	0.1000	1.024931e-1
STD_0.5ppb	Standard	9.52e3	3.275e5	6.36	20.5	0.5000	5.126449e-1
STD_1ppb	Standard	1.53e4	3.746e5	6.36	23.1	1.0000	7.101133e-1
STD_5ppb	Standard	8.91e4	3.209e5	6.37	30.8	5.0000	4.706418e0
STD_10ppb	Standard	1.84e5	3.165e5	6.36	36.4	10.0000	9.848069e0
STD_50ppb	Standard	1.15e6	3.582e5	6.36	56.4	50.0000	5.439842e1
STD_100ppb	Standard	2.31e6	3.626e5	6.37	46.7	100.0000	1.074117e2
STD_200ppb	Standard	5.05e6	3.701e5	6.36	63.7	200.0000	2.302980e2
STD_500ppb	Standard	N/A	4.777e5	N/A	N/A	500.0000	N/A
V2.0_MW_RQC1_20240724	Quality Control	2.14e4	1.299e5	6.32	10.0	0.0000	2.802933e0
Blank	Unknown	N/A	5.220e2	N/A	N/A	N/A	N/A
V3.0_MWMS_20240725_1	Unknown	1.37e6	3.708e5	6.34	60.5	N/A	6.227852e1
MWXS243064a_R1	Quality Control	8.94e3	1.034e5	6.32	13.4	0.0000	1.480541e0
MWXS243064a_R2	Quality Control	8.20e3	1.066e5	6.33	11.0	0.0000	1.320873e0
MWXS243064a_R3	Quality Control	7.59e3	1.022e5	6.32	12.5	0.0000	1.274641e0
T24186682b_a	Unknown	4.13e3	8.442e4	6.33	7.8	N/A	8.478635e-1
T24186682b_b	Unknown	4.51e3	7.738e4	6.34	5.7	N/A	1.004167e0
T24186682b_c	Unknown	5.91e3	9.288e4	6.33	9.8	N/A	1.094793e0
T24186683b_a	Unknown	6.50e3	1.648e5	6.32	9.6	N/A	6.874492e-1
T24186683b_b	Unknown	6.13e3	1.807e5	6.33	10.0	N/A	5.946780e-1
T24186683b_c	Unknown	6.56e3	1.771e5	6.33	6.3	N/A	6.466756e-1
T24186684b_a	Unknown	1.13e4	9.244e4	6.33	15.7	N/A	2.091670e0
T24186684b_b	Unknown	1.30e4	1.062e5	6.34	18.7	N/A	2.080501e0
T24186684b_c	Unknown	1.56e4	1.130e5	6.34	19.4	N/A	2.343491e0

Compound name: GA24
Regression Equation: y = 0.05928 x + -0.00130 (r = 0.99022) (weighting: 1 / x^2)

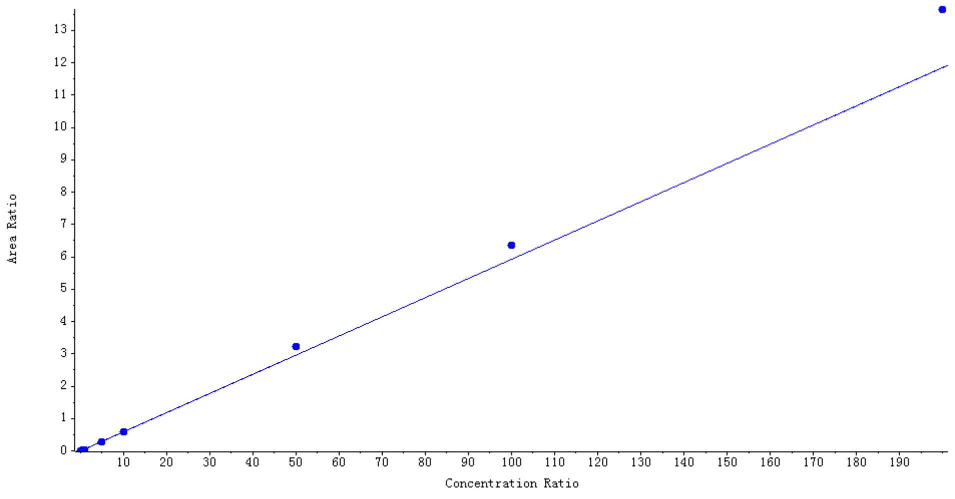

Peak Review

Blank

GA24 AREA:N/A S/N:N/A

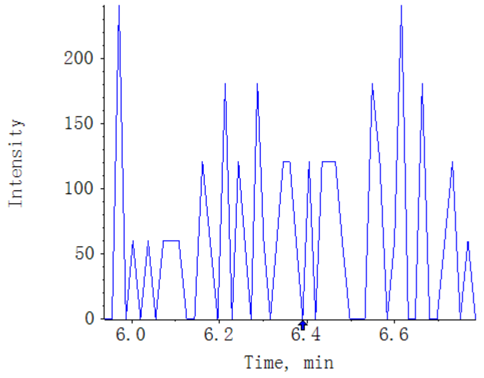

V3.0_MWMS_20240725_1

GA24 AREA:1.37e6 S/N:60.5

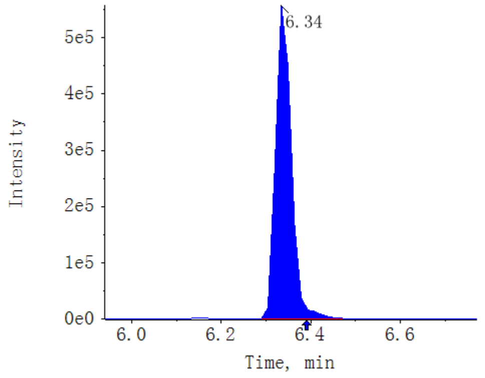

T24186682b_a

GA24 AREA:4.13e3 S/N:7.8

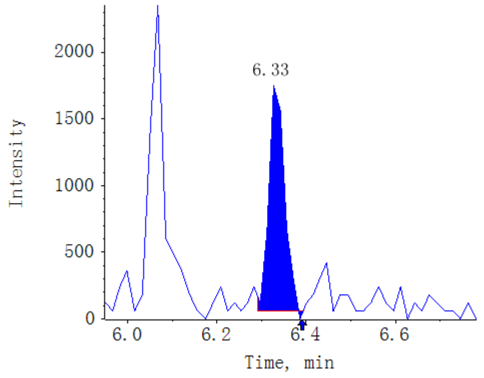

T24186682b_b

GA24 AREA:4.51e3 S/N:5.7

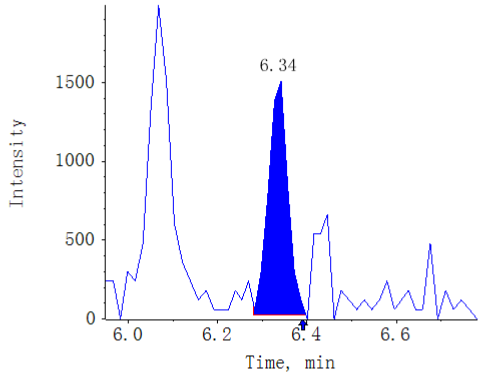

T24186682b_c

GA24 AREA:5.91e3 S/N:9.8

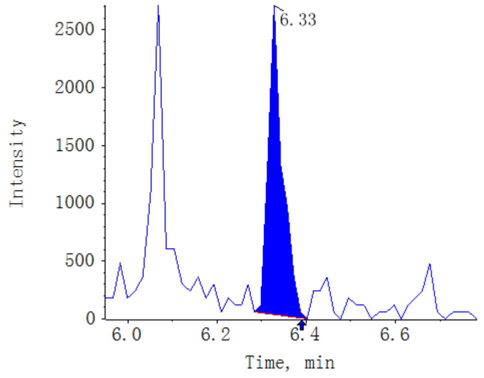

T24186683b_a

GA24 AREA:6.50e3 S/N:9.6

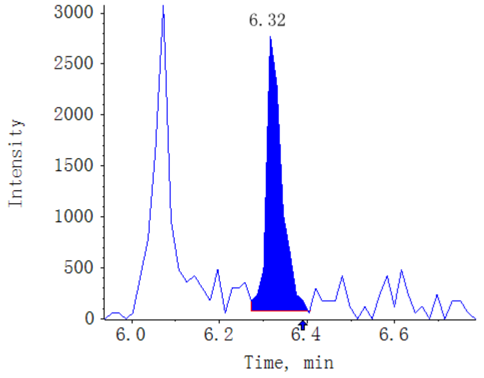

T24186683b_b

GA24 AREA:6.13e3 S/N:10.0

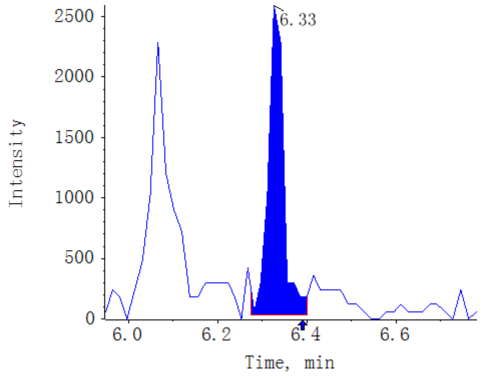

T24186683b_c

GA24 AREA:6.56e3 S/N:6.3

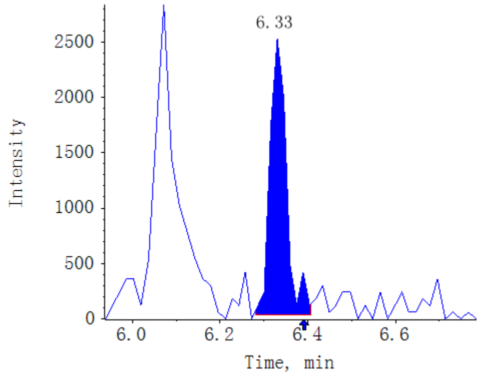

T24186684b_a

GA24 AREA:1.13e4 S/N:15.7

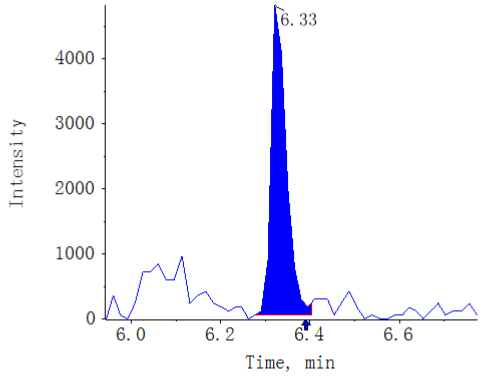

T24186684b_b

GA24 AREA:1.30e4 S/N:18.7

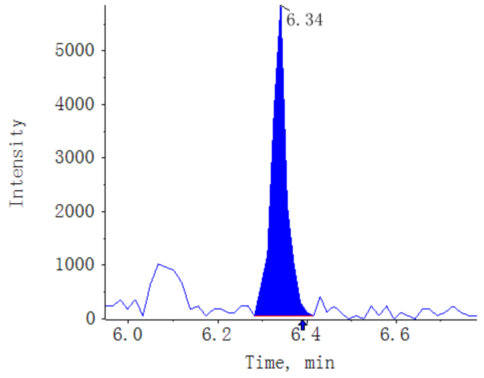

T24186684b_c

GA24 AREA:1.56e4 S/N:19.4

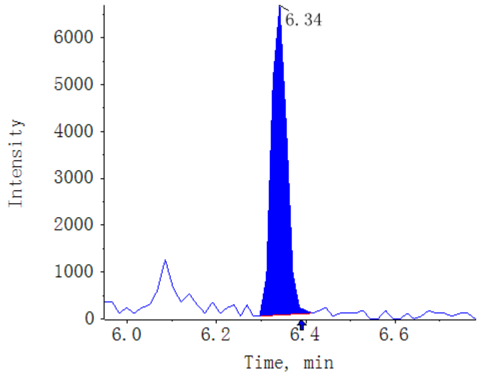

Result Table	MWXS-24-3064-a_9_WH6500-17_A20-3_V6.0_WSS_20240730	Algorithm Used	MQ4
Acquisition Method	ACC-PHs_V6.0_WH6500-17_CMY_20240521.dam	Instrument Name	Triple Quad 6500+ Low Mass
Project	N/A	Analytes QTY	109:88

Compound name: GA3 (345.0 / 239.0)

Sample Name	Sample Type	Area (cps)	Is Area (cps)	RT (min)	S/N	Target Conc	Calculated Conc.()
STD_0.01ppb	Standard	N/A	3.538e5	N/A	N/A	0.0100	N/A
STD_0.05ppb	Standard	4.06e3	3.397e5	4.46	10.3	0.0500	5.430110e-2
STD_0.1ppb	Standard	5.43e3	3.587e5	4.47	9.7	0.1000	8.473325e-2
STD_0.5ppb	Standard	1.81e4	3.275e5	4.46	27.6	0.5000	4.696493e-1
STD_1ppb	Standard	3.69e4	3.746e5	4.46	40.9	1.0000	8.842105e-1
STD_5ppb	Standard	1.94e5	3.209e5	4.47	66.7	5.0000	5.736692e0
STD_10ppb	Standard	3.75e5	3.165e5	4.46	59.5	10.0000	1.130141e1
STD_50ppb	Standard	2.05e6	3.582e5	4.45	53.7	50.0000	5.479005e1
STD_100ppb	Standard	3.54e6	3.626e5	4.47	61.8	100.0000	9.357884e1
STD_200ppb	Standard	7.21e6	3.701e5	4.46	46.0	200.0000	1.868135e2
STD_500ppb	Standard	N/A	4.777e5	N/A	N/A	500.0000	N/A
V2.0_MW_RQC1_20240724	Quality Control	N/A	1.299e5	N/A	N/A	0.0000	N/A
Blank	Unknown	N/A	5.220e2	N/A	N/A	N/A	N/A
V3.0_MWMS_20240725_1	Unknown	2.22e6	3.708e5	4.45	33.8	N/A	5.736660e1
MWXS243064a_R1	Quality Control	5.12e4	1.034e5	4.45	15.9	0.0000	4.699121e0
MWXS243064a_R2	Quality Control	6.12e4	1.066e5	4.46	19.0	0.0000	5.452719e0
MWXS243064a_R3	Quality Control	5.64e4	1.022e5	4.45	22.8	0.0000	5.234502e0
T24186682b_a	Unknown	3.50e4	8.442e4	4.46	19.1	N/A	3.923704e0
T24186682b_b	Unknown	3.89e4	7.738e4	4.46	20.5	N/A	4.769761e0
T24186682b_c	Unknown	4.13e4	9.288e4	4.46	17.2	N/A	4.210955e0
T24186683b_a	Unknown	1.70e4	1.648e5	4.45	13.8	N/A	9.297482e-1
T24186683b_b	Unknown	8.97e3	1.807e5	4.47	7.0	N/A	4.160056e-1
T24186683b_c	Unknown	1.21e4	1.771e5	4.46	7.1	N/A	5.958406e-1
T24186684b_a	Unknown	4.48e4	9.244e4	4.46	11.9	N/A	4.591707e0
T24186684b_b	Unknown	3.60e4	1.062e5	4.46	13.8	N/A	3.195241e0
T24186684b_c	Unknown	4.36e4	1.130e5	4.46	12.8	N/A	3.640184e0

Compound name: GA3
Regression Equation: y = 0.10418 x + 0.00630 (r = 0.99243) (weighting: 1 / x^2)

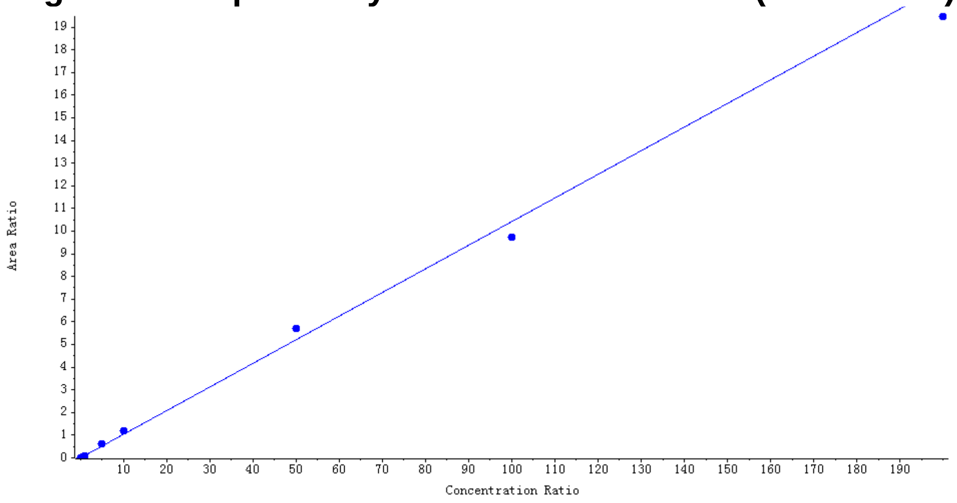

Peak Review

Blank

GA3 AREA:N/A S/N:N/A

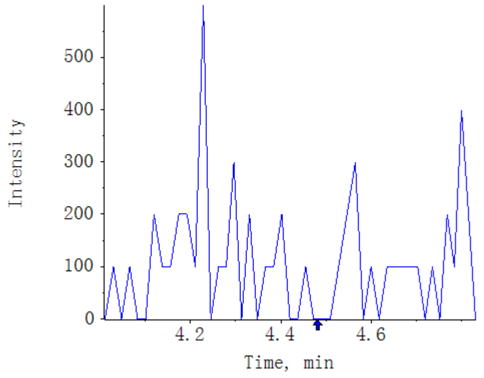

V3.0_MWMS_20240725_1

GA3 AREA:2.22e6 S/N:33.8

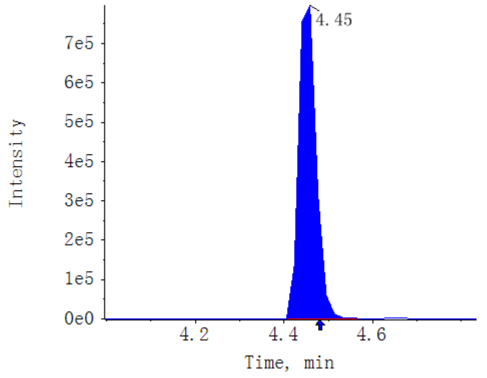

T24186682b_a

GA3 AREA:3.50e4 S/N:19.1

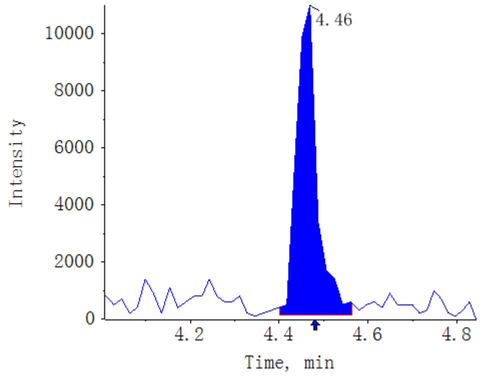

T24186682b_b

GA3 AREA:3.89e4 S/N:20.5

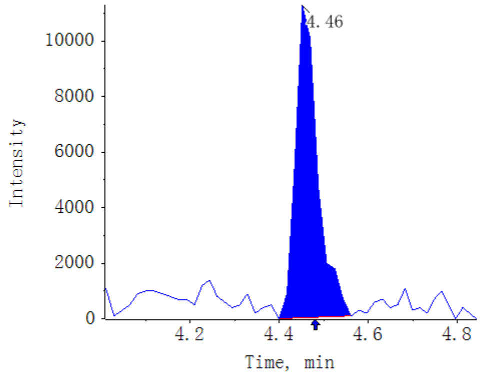

T24186682b_c

GA3 AREA:4.13e4 S/N:17.2

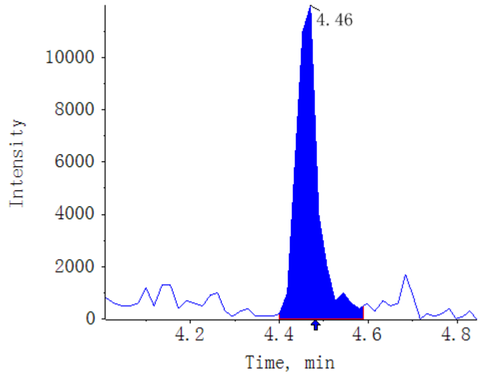

T24186683b_a

GA3 AREA:1.70e4 S/N:13.8

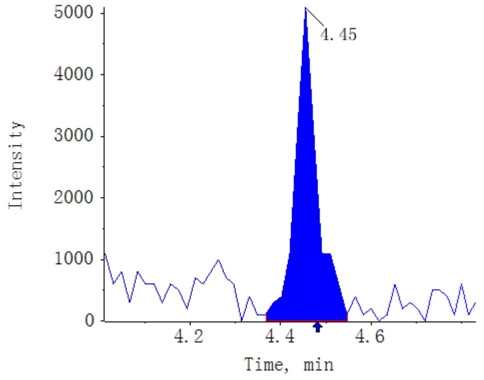

T24186683b_b

GA3 AREA:8.97e3 S/N:7.0

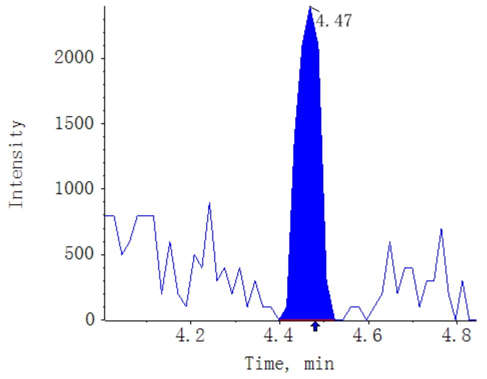

T24186683b_c

GA3 AREA:1.21e4 S/N:7.1

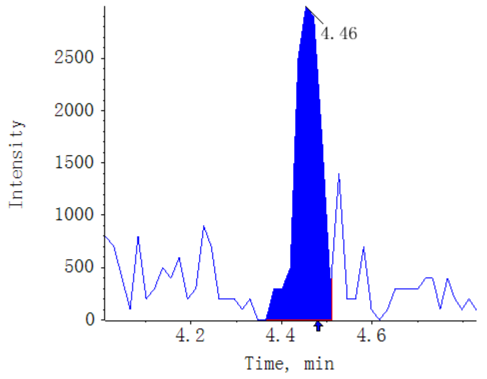

T24186684b_a

GA3 AREA:4.48e4 S/N:11.9

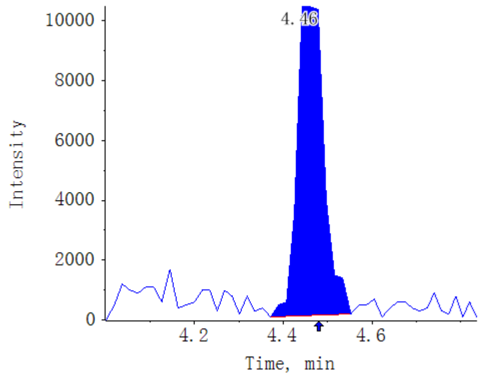

T24186684b_b

GA3 AREA:3.60e4 S/N:13.8

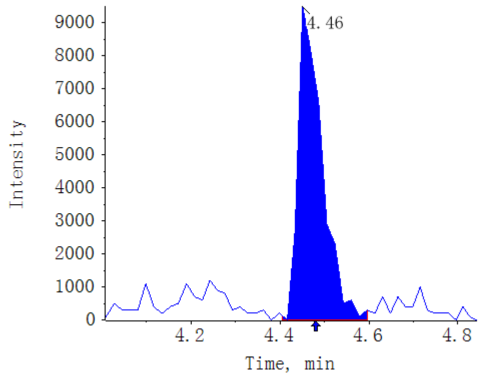

T24186684b_c

GA3 AREA:4.36e4 S/N:12.8

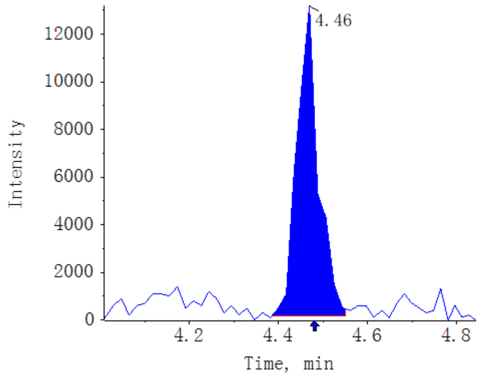

Result Table	MWXS-24-3064-a_9_WH6500-17_A20-3_V6.0_WSS_20240730	Algorithm Used	MQ4
Acquisition Method	ACC-PHs_V6.0_WH6500-17_CMY_20240521.dam	Instrument Name	Triple Quad 6500+ Low Mass
Project	N/A	Analytes QTY	109:89

Compound name: GA4 (331.0 / 212.8)

Sample Name	Sample Type	Area (cps)	Is Area (cps)	RT (min)	S/N	Target Conc	Calculated Conc.()
STD_0.01ppb	Standard	N/A	3.538e5	N/A	N/A	0.0100	N/A
STD_0.05ppb	Standard	N/A	3.397e5	N/A	N/A	0.0500	N/A
STD_0.1ppb	Standard	N/A	3.587e5	N/A	N/A	0.1000	N/A
STD_0.5ppb	Standard	1.08e4	3.275e5	6.18	10.2	0.5000	5.372462e-1
STD_1ppb	Standard	1.82e4	3.746e5	6.18	12.1	1.0000	8.340795e-1
STD_5ppb	Standard	9.08e4	3.209e5	6.19	64.2	5.0000	5.281121e0
STD_10ppb	Standard	1.75e5	3.165e5	6.17	54.9	10.0000	1.038410e1
STD_50ppb	Standard	1.02e6	3.582e5	6.17	71.7	50.0000	5.377408e1
STD_100ppb	Standard	1.96e6	3.626e5	6.18	61.7	100.0000	1.022177e2
STD_200ppb	Standard	4.32e6	3.701e5	6.18	67.9	200.0000	2.213361e2
STD_500ppb	Standard	9.98e6	4.777e5	6.18	69.8	500.0000	3.962273e2
V2.0_MW_RQC1_20240724	Quality Control	5.92e3	1.299e5	6.14	5.6	0.0000	7.782622e-1
Blank	Unknown	N/A	5.220e2	N/A	N/A	N/A	N/A
V3.0_MWMS_20240725_1	Unknown	8.88e5	3.708e5	6.15	66.9	N/A	4.538247e1
MWXS243064a_R1	Quality Control	N/A	1.034e5	N/A	N/A	0.0000	N/A
MWXS243064a_R2	Quality Control	N/A	1.066e5	N/A	N/A	0.0000	N/A
MWXS243064a_R3	Quality Control	N/A	1.022e5	N/A	N/A	0.0000	N/A
T24186682b_a	Unknown	N/A	8.442e4	N/A	N/A	N/A	N/A
T24186682b_b	Unknown	N/A	7.738e4	N/A	N/A	N/A	N/A
T24186682b_c	Unknown	N/A	9.288e4	N/A	N/A	N/A	N/A
T24186683b_a	Unknown	N/A	1.648e5	N/A	N/A	N/A	N/A
T24186683b_b	Unknown	N/A	1.807e5	N/A	N/A	N/A	N/A
T24186683b_c	Unknown	N/A	1.771e5	N/A	N/A	N/A	N/A
T24186684b_a	Unknown	N/A	9.244e4	N/A	N/A	N/A	N/A
T24186684b_b	Unknown	N/A	1.062e5	N/A	N/A	N/A	N/A
T24186684b_c	Unknown	N/A	1.130e5	N/A	N/A	N/A	N/A

Compound name: GA4
Regression Equation: y = 0.05270 x + 0.00459 (r = 0.99162) (weighting: 1 / x^2)

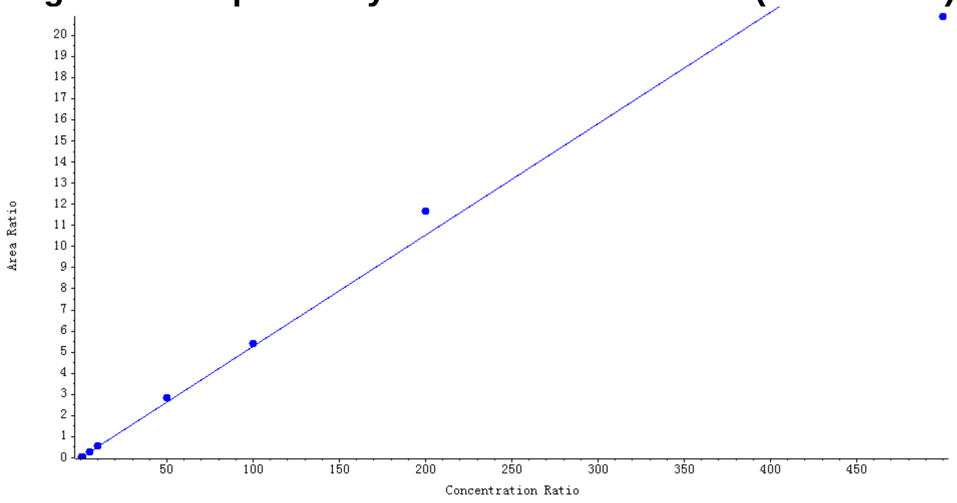

Peak Review

Blank
GA4 AREA:N/A S/N:N/A

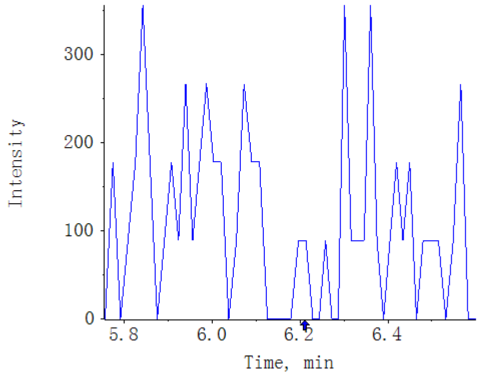

V3.0_MWMS_20240725_1
GA4 AREA:8.88e5 S/N:66.9

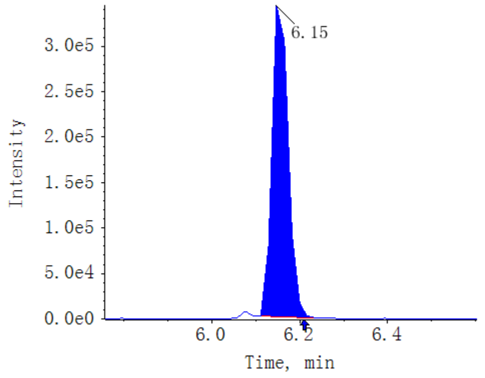

T24186682b_a
GA4 AREA:N/A S/N:N/A

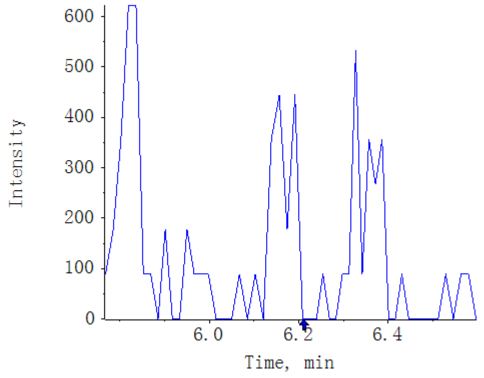

T24186682b_b
GA4 AREA:N/A S/N:N/A

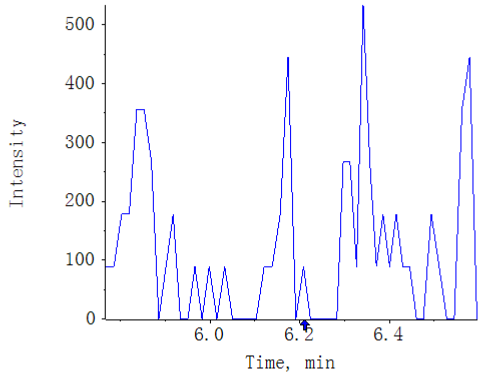

T24186682b_c
GA4 AREA:N/A S/N:N/A

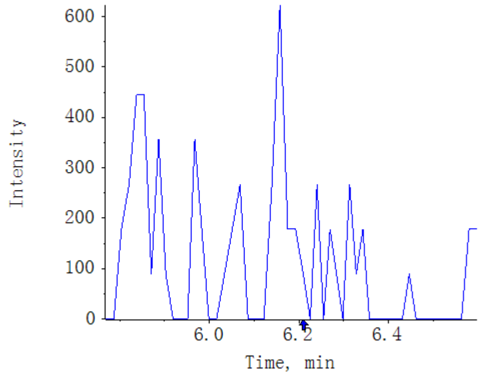

T24186683b_a
GA4 AREA:N/A S/N:N/A

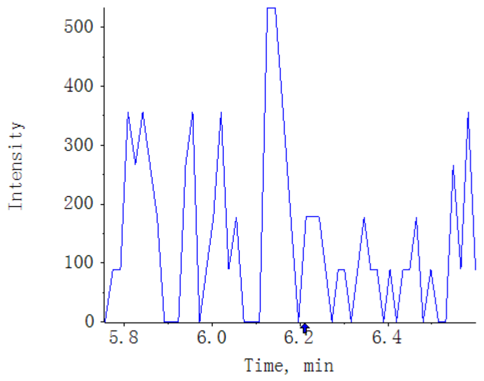

T24186683b_b
GA4 AREA:N/A S/N:N/A

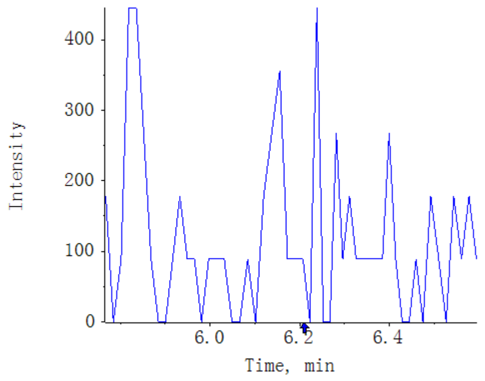

T24186683b_c
GA4 AREA:N/A S/N:N/A

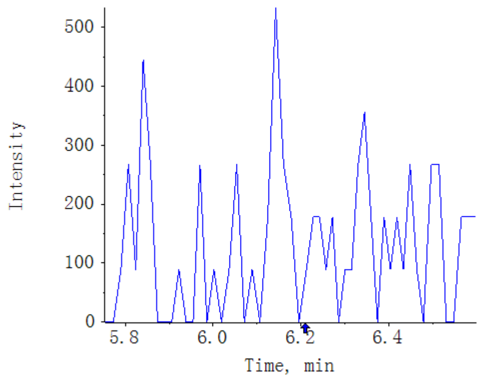

T24186684b_a
GA4 AREA:N/A S/N:N/A

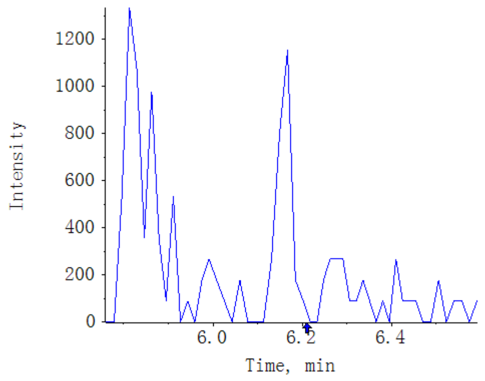

T24186684b_b
GA4 AREA:N/A S/N:N/A

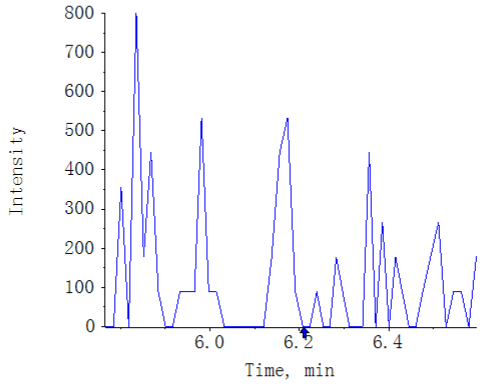

T24186684b_c
GA4 AREA:N/A S/N:N/A

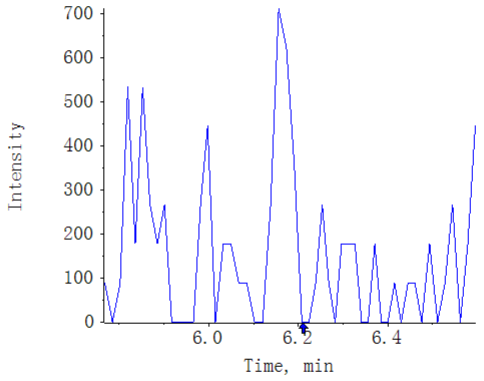

Result Table	MWXS-24-3064-a_9_WH6500-17_A20-3_V6.0_WSS_20240730	Algorithm Used	MQ4
Acquisition Method	ACC-PHs_V6.0_WH6500-17_CMY_20240521.dam	Instrument Name	Triple Quad 6500+ Low Mass
Project	N/A	Analytes QTY	109:90

Compound name: GA53 (347.1 / 303.1)

Sample Name	Sample Type	Area (cps)	Is Area (cps)	RT (min)	S/N	Target Conc	Calculated Conc.()
STD_0.01ppb	Standard	N/A	2.861e5	N/A	N/A	0.0100	N/A
STD_0.05ppb	Standard	N/A	2.595e5	N/A	N/A	0.0500	N/A
STD_0.1ppb	Standard	N/A	2.773e5	N/A	N/A	0.1000	N/A
STD_0.5ppb	Standard	8.26e3	2.740e5	5.76	17.2	0.5000	4.534986e-1
STD_1ppb	Standard	1.95e4	2.736e5	5.77	24.6	1.0000	1.169578e0
STD_5ppb	Standard	8.62e4	2.727e5	5.78	36.6	5.0000	5.408396e0
STD_10ppb	Standard	1.53e5	2.579e5	5.77	44.2	10.0000	1.020926e1
STD_50ppb	Standard	7.99e5	2.825e5	5.77	50.2	50.0000	4.895524e1
STD_100ppb	Standard	1.50e6	3.087e5	5.78	78.3	100.0000	8.417146e1
STD_200ppb	Standard	N/A	3.833e5	N/A	N/A	200.0000	N/A
STD_500ppb	Standard	N/A	4.987e5	N/A	N/A	500.0000	N/A
V2.0_MW_RQC1_20240724	Quality Control	1.18e4	1.766e5	5.75	7.8	0.0000	1.088780e0
Blank	Unknown	N/A	6.192e2	N/A	N/A	N/A	N/A
V3.0_MWMS_20240725_1	Unknown	7.03e5	3.199e5	5.75	61.0	N/A	3.801105e1
MWXS243064a_R1	Quality Control	N/A	2.017e5	N/A	N/A	0.0000	N/A
MWXS243064a_R2	Quality Control	N/A	2.213e5	N/A	N/A	0.0000	N/A
MWXS243064a_R3	Quality Control	N/A	2.075e5	N/A	N/A	0.0000	N/A
T24186682b_a	Unknown	N/A	2.225e5	N/A	N/A	N/A	N/A
T24186682b_b	Unknown	N/A	2.077e5	N/A	N/A	N/A	N/A
T24186682b_c	Unknown	N/A	1.990e5	N/A	N/A	N/A	N/A
T24186683b_a	Unknown	6.17e3	2.008e5	5.78	5.0	N/A	4.639996e-1
T24186683b_b	Unknown	8.32e3	2.109e5	5.77	8.2	N/A	6.146986e-1
T24186683b_c	Unknown	7.55e3	2.133e5	5.78	8.8	N/A	5.440513e-1
T24186684b_a	Unknown	N/A	1.861e5	N/A	N/A	N/A	N/A
T24186684b_b	Unknown	N/A	1.949e5	N/A	N/A	N/A	N/A
T24186684b_c	Unknown	N/A	2.203e5	N/A	N/A	N/A	N/A

Compound name: GA53
Regression Equation: y = 0.05769 x + 0.00398 (r = 0.99092) (weighting: 1 / x^2)

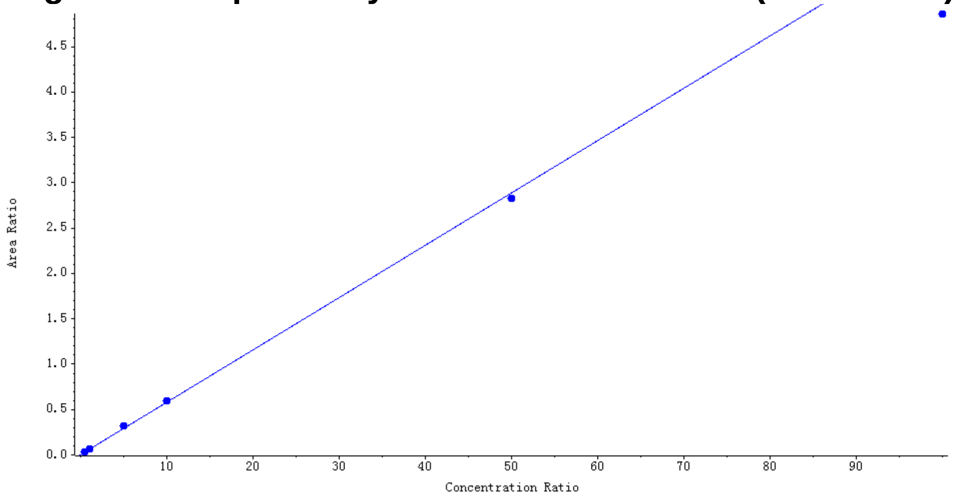

Peak Review

Blank
GA53 AREA:N/A S/N:N/A

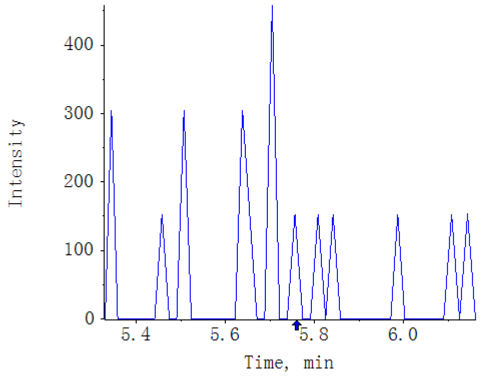

V3.0_MWMS_20240725_1
GA53 AREA:7.03e5 S/N:61.0

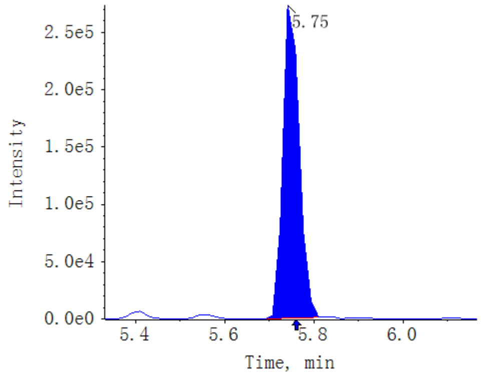

T24186682b_a
GA53 AREA:N/A S/N:N/A

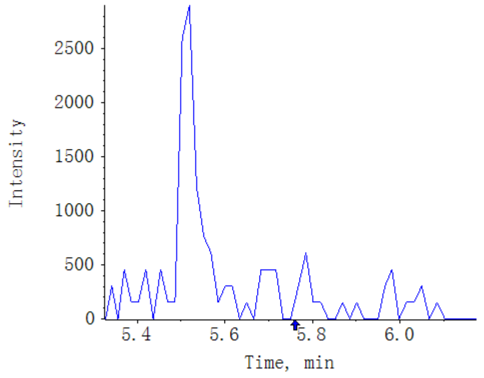

T24186682b_b
GA53 AREA:N/A S/N:N/A

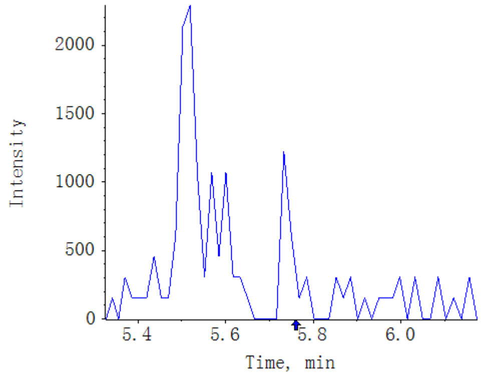

T24186682b_c
GA53 AREA:N/A S/N:N/A

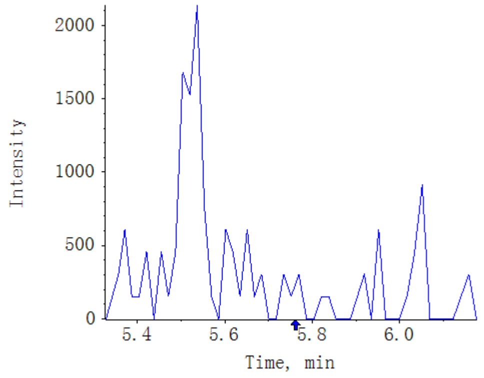

T24186683b_a
GA53 AREA:6.17e3 S/N:5.0

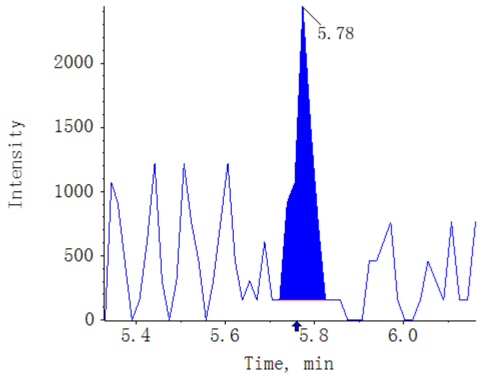

T24186683b_b
GA53 AREA:8.32e3 S/N:8.2

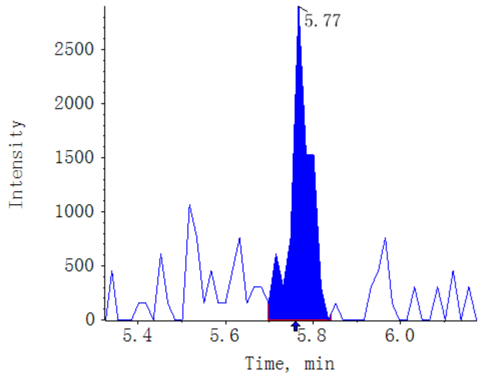

T24186683b_c
GA53 AREA:7.55e3 S/N:8.8

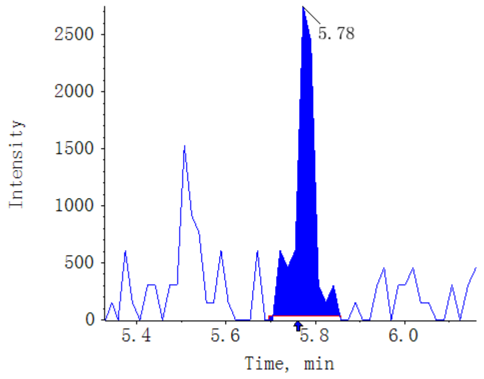

T24186684b_a
GA53 AREA:N/A S/N:N/A

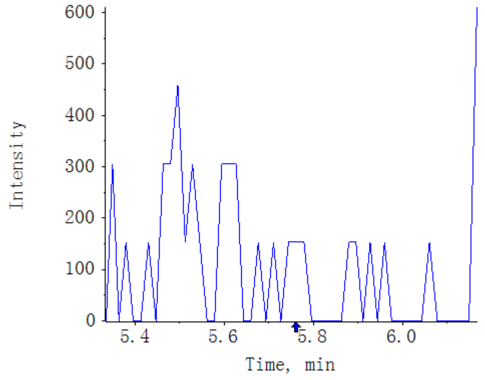

T24186684b_b
GA53 AREA:N/A S/N:N/A

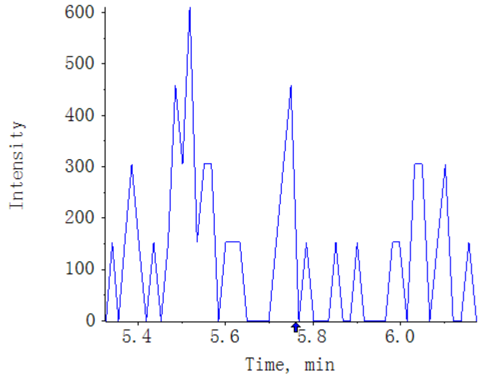

T24186684b_c
GA53 AREA:N/A S/N:N/A

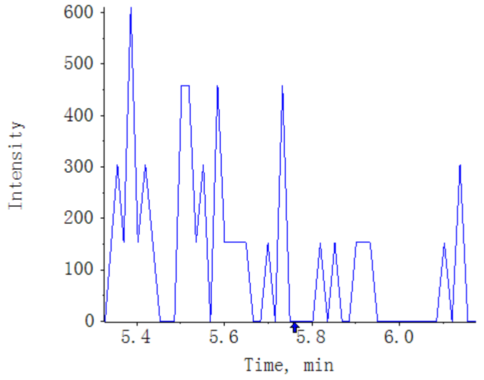

Result Table	MWXS-24-3064-a_9_WH6500-17_A20-3_V6.0_WSS_20240730	Algorithm Used	MQ4
Acquisition Method	ACC-PHs_V6.0_WH6500-17_CMY_20240521.dam	Instrument Name	Triple Quad 6500+ Low Mass
Project	N/A	Analytes QTY	109:91

Compound name: GA7 (329.2 / 223.0)

Sample Name	Sample Type	Area (cps)	Is Area (cps)	RT (min)	S/N	Target Conc	Calculated Conc.()
STD_0.01ppb	Standard	1.65e3	3.538e5	6.11	6.2	0.0100	9.442285e-3
STD_0.05ppb	Standard	1.12e4	3.397e5	6.10	18.2	0.0500	6.225082e-2
STD_0.1ppb	Standard	2.05e4	3.587e5	6.12	25.3	0.1000	1.079981e-1
STD_0.5ppb	Standard	8.99e4	3.275e5	6.10	64.7	0.5000	5.158586e-1
STD_1ppb	Standard	1.57e5	3.746e5	6.11	63.1	1.0000	7.879884e-1
STD_5ppb	Standard	9.63e5	3.209e5	6.12	66.8	5.0000	5.627776e0
STD_10ppb	Standard	1.73e6	3.165e5	6.10	67.3	10.0000	1.025644e1
STD_50ppb	Standard	9.36e6	3.582e5	6.10	81.3	50.0000	4.898366e1
STD_100ppb	Standard	1.71e7	3.626e5	6.11	67.2	100.0000	8.841785e1
STD_200ppb	Standard	3.54e7	3.701e5	6.11	81.8	200.0000	1.792035e2
STD_500ppb	Standard	N/A	4.777e5	N/A	N/A	500.0000	N/A
V2.0_MW_RQC1_20240724	Quality Control	N/A	1.299e5	N/A	N/A	0.0000	N/A
Blank	Unknown	N/A	5.220e2	N/A	N/A	N/A	N/A
V3.0_MWMS_20240725_1	Unknown	7.19e6	3.708e5	6.08	102.0	N/A	3.636864e1
MWXS243064a_R1	Quality Control	N/A	1.034e5	N/A	N/A	0.0000	N/A
MWXS243064a_R2	Quality Control	N/A	1.066e5	N/A	N/A	0.0000	N/A
MWXS243064a_R3	Quality Control	N/A	1.022e5	N/A	N/A	0.0000	N/A
T24186682b_a	Unknown	N/A	8.442e4	N/A	N/A	N/A	N/A
T24186682b_b	Unknown	N/A	7.738e4	N/A	N/A	N/A	N/A
T24186682b_c	Unknown	N/A	9.288e4	N/A	N/A	N/A	N/A
T24186683b_a	Unknown	N/A	1.648e5	N/A	N/A	N/A	N/A
T24186683b_b	Unknown	N/A	1.807e5	N/A	N/A	N/A	N/A
T24186683b_c	Unknown	N/A	1.771e5	N/A	N/A	N/A	N/A
T24186684b_a	Unknown	N/A	9.244e4	N/A	N/A	N/A	N/A
T24186684b_b	Unknown	N/A	1.062e5	N/A	N/A	N/A	N/A
T24186684b_c	Unknown	N/A	1.130e5	N/A	N/A	N/A	N/A

Compound name: GA7
Regression Equation: y = 0.53315 x + -3.56507e-4 (r = 0.99071) (weighting: 1 / x^2)

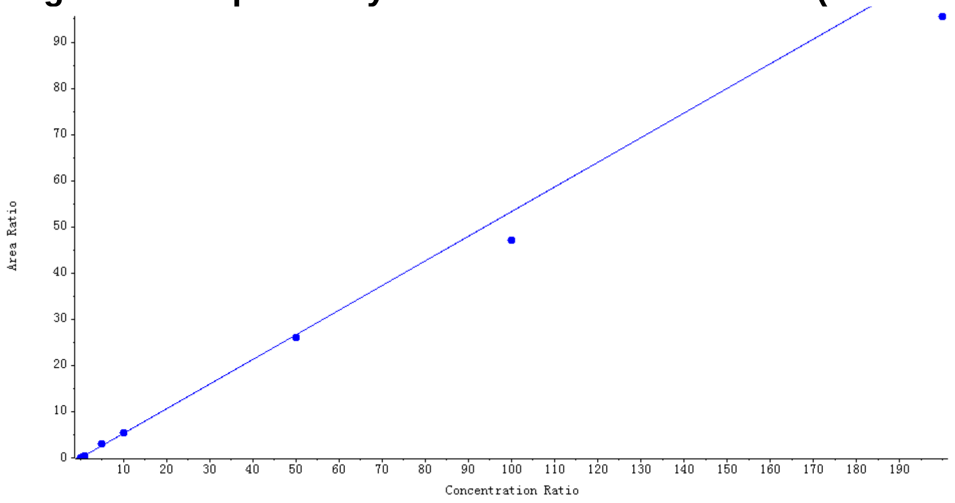

Peak Review

Blank
GA7 AREA:N/A S/N:N/A

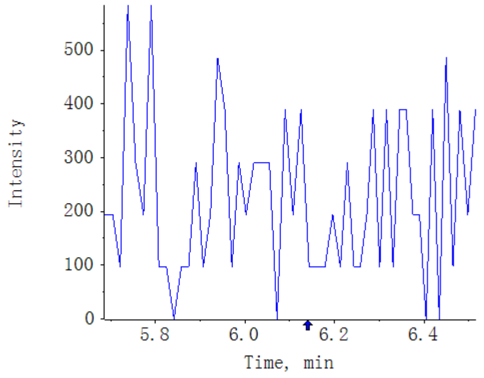

V3.0_MWMS_20240725_1
GA7 AREA:7.19e6 S/N:102.0

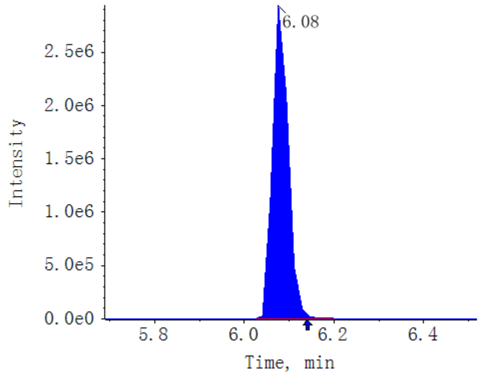

T24186682b_a
GA7 AREA:N/A S/N:N/A

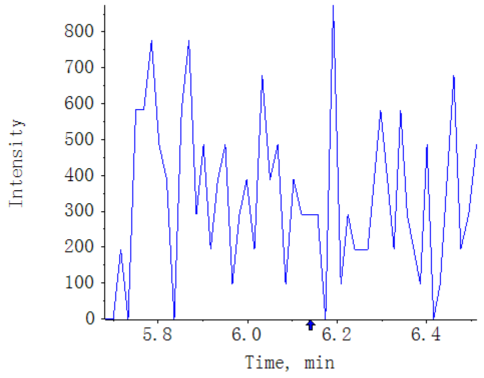

T24186682b_b
GA7 AREA:N/A S/N:N/A

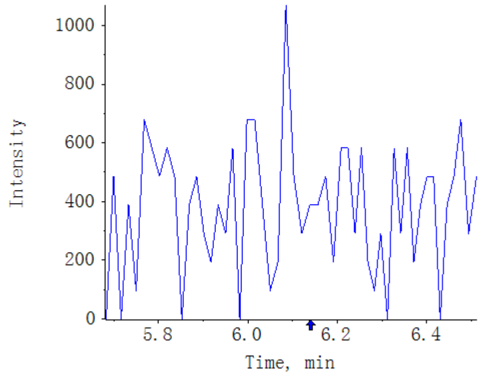

T24186682b_c
GA7 AREA:N/A S/N:N/A

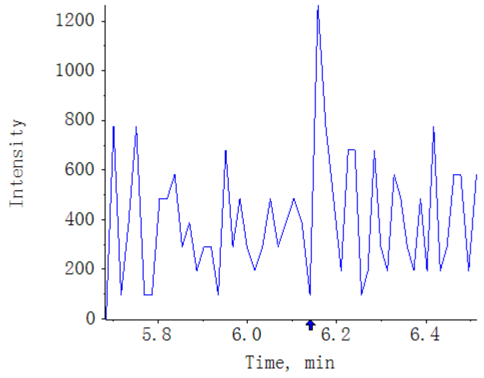

T24186683b_a
GA7 AREA:N/A S/N:N/A

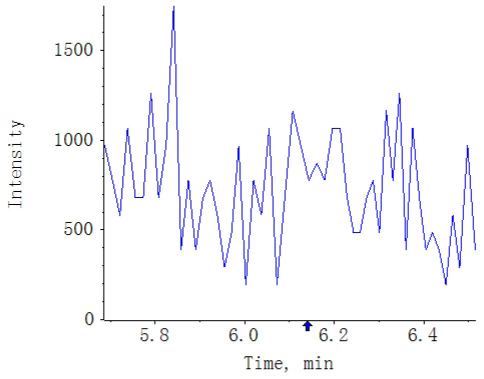

T24186683b_b
GA7 AREA:N/A S/N:N/A

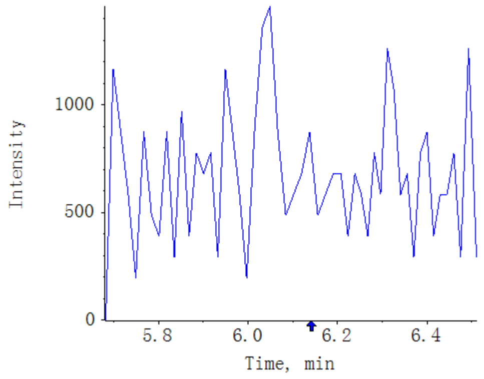

T24186683b_c
GA7 AREA:N/A S/N:N/A

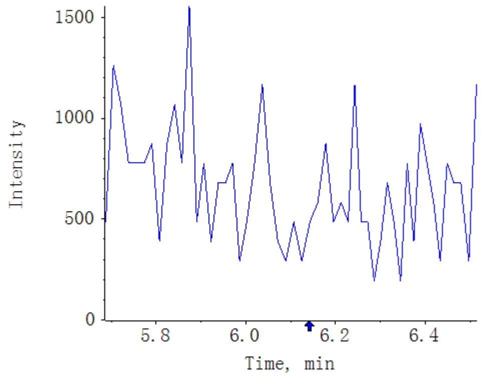

T24186684b_a
GA7 AREA:N/A S/N:N/A

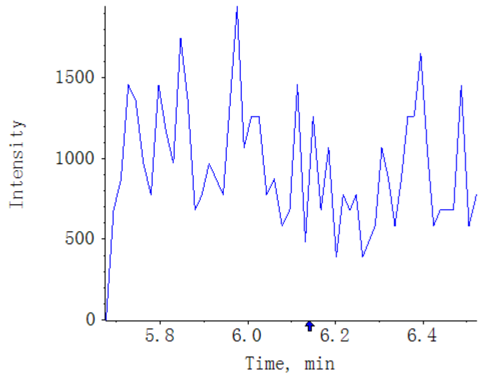

T24186684b_b
GA7 AREA:N/A S/N:N/A

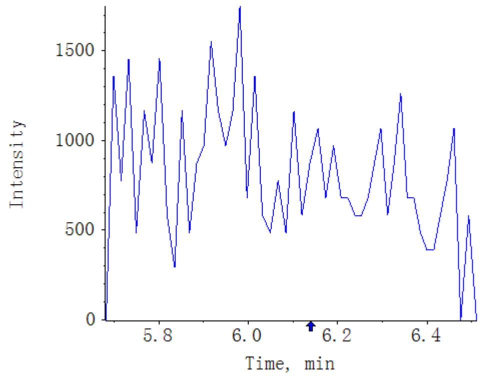

T24186684b_c
GA7 AREA:N/A S/N:N/A

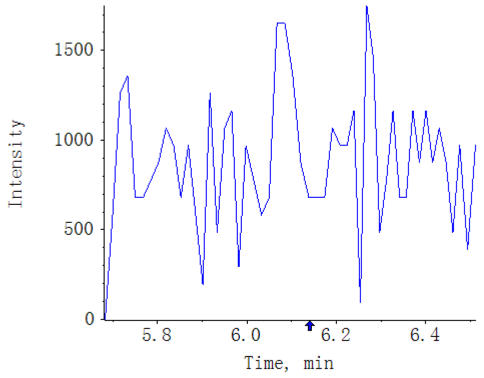

Result Table	MWXS-24-3064-a_9_WH6500-17_A20-3_V6.0_WSS_20240730	Algorithm Used	MQ4
Acquisition Method	ACC-PHs_V6.0_WH6500-17_CMY_20240521.dam	Instrument Name	Triple Quad 6500+ Low Mass
Project	N/A	Analytes QTY	109:92

Compound name: GA9 (315.1 / 270.9)

Sample Name	Sample Type	Area (cps)	Is Area (cps)	RT (min)	S/N	Target Conc	Calculated Conc.()
STD_0.01ppb	Standard	N/A	4.523e5	N/A	N/A	0.0100	N/A
STD_0.05ppb	Standard	N/A	4.667e5	N/A	N/A	0.0500	N/A
STD_0.1ppb	Standard	3.97e3	4.509e5	6.89	6.8	0.1000	9.708936e-2
STD_0.5ppb	Standard	1.86e4	4.650e5	6.88	20.1	0.5000	5.819583e-1
STD_1ppb	Standard	3.00e4	4.767e5	6.88	21.0	1.0000	9.379783e-1
STD_5ppb	Standard	1.78e5	4.788e5	6.89	55.2	5.0000	5.739618e0
STD_10ppb	Standard	3.13e5	5.039e5	6.88	52.7	10.0000	9.612460e0
STD_50ppb	Standard	1.69e6	4.980e5	6.88	67.8	50.0000	5.267353e1
STD_100ppb	Standard	3.01e6	5.035e5	6.89	53.0	100.0000	9.301388e1
STD_200ppb	Standard	6.01e6	5.601e5	6.88	57.8	200.0000	1.668865e2
STD_500ppb	Standard	N/A	6.640e5	N/A	N/A	500.0000	N/A
V2.0_MW_RQC1_20240724	Quality Control	N/A	2.635e5	N/A	N/A	0.0000	N/A
Blank	Unknown	N/A	1.869e4	N/A	N/A	N/A	N/A
V3.0_MWMS_20240725_1	Unknown	1.89e6	5.318e5	6.87	62.1	N/A	5.533031e1
MWXS243064a_R1	Quality Control	N/A	2.704e5	N/A	N/A	0.0000	N/A
MWXS243064a_R2	Quality Control	N/A	2.550e5	N/A	N/A	0.0000	N/A
MWXS243064a_R3	Quality Control	N/A	2.590e5	N/A	N/A	0.0000	N/A
T24186682b_a	Unknown	N/A	2.533e5	N/A	N/A	N/A	N/A
T24186682b_b	Unknown	N/A	2.649e5	N/A	N/A	N/A	N/A
T24186682b_c	Unknown	N/A	2.571e5	N/A	N/A	N/A	N/A
T24186683b_a	Unknown	5.96e3	3.090e5	6.84	5.8	N/A	2.601024e-1
T24186683b_b	Unknown	6.54e3	3.048e5	6.85	6.8	N/A	2.938920e-1
T24186683b_c	Unknown	5.12e3	3.009e5	6.85	5.8	N/A	2.247728e-1
T24186684b_a	Unknown	N/A	2.526e5	N/A	N/A	N/A	N/A
T24186684b_b	Unknown	N/A	2.695e5	N/A	N/A	N/A	N/A
T24186684b_c	Unknown	N/A	2.775e5	N/A	N/A	N/A	N/A

Compound name: GA9
Regression Equation: y = 0.06427 x + 0.00257 (r = 0.99293) (weighting: 1 / x^2)

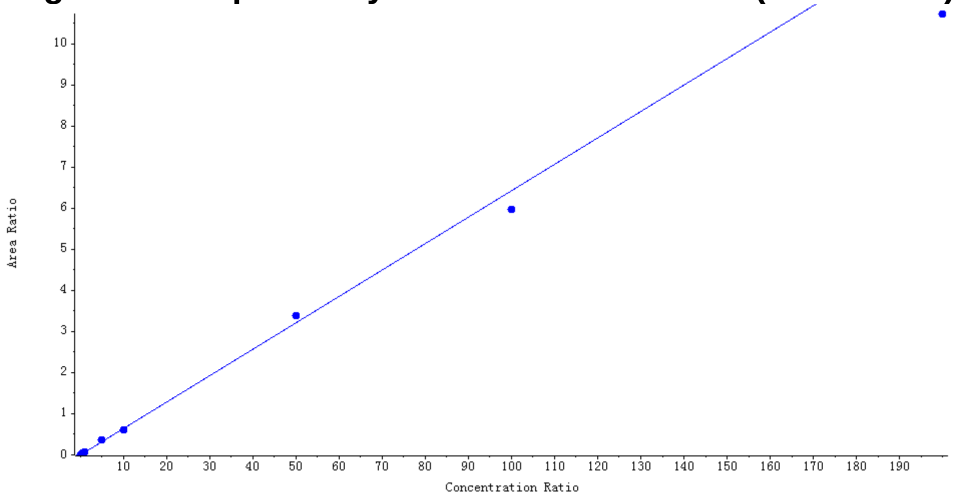

Peak Review

Blank

GA9 AREA:N/A S/N:N/A

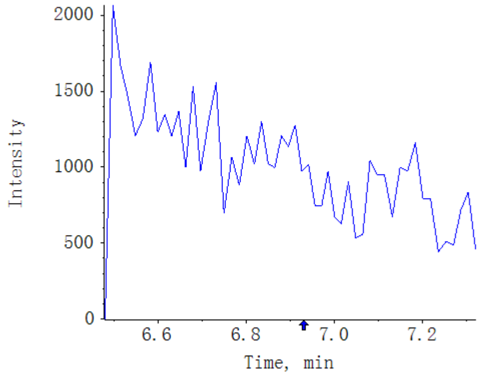

V3.0_MWMS_20240725_1

GA9 AREA:1.89e6 S/N:62.1

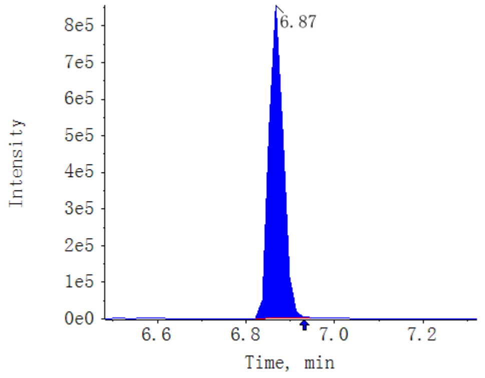

T24186682b_a

GA9 AREA:N/A S/N:N/A

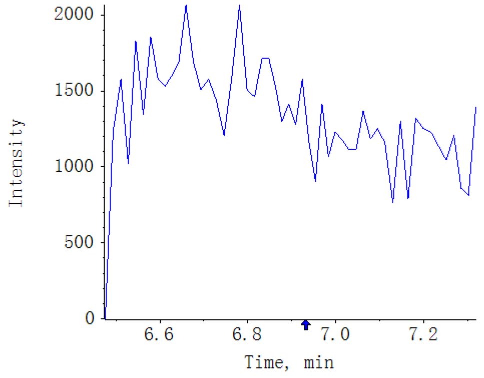

T24186682b_b

GA9 AREA:N/A S/N:N/A

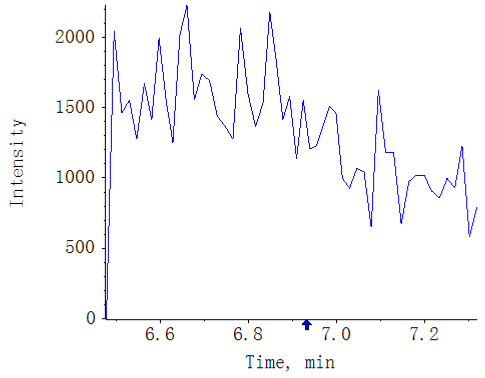

T24186682b_c

GA9 AREA:N/A S/N:N/A

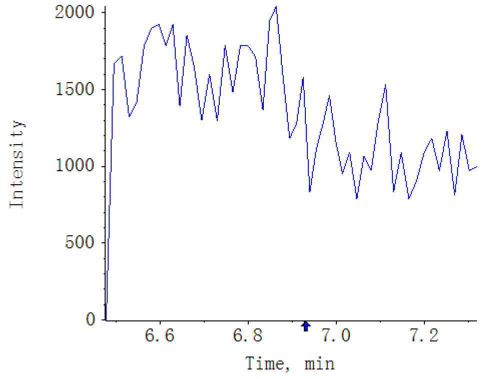

T24186683b_a

GA9 AREA:5.96e3 S/N:5.8

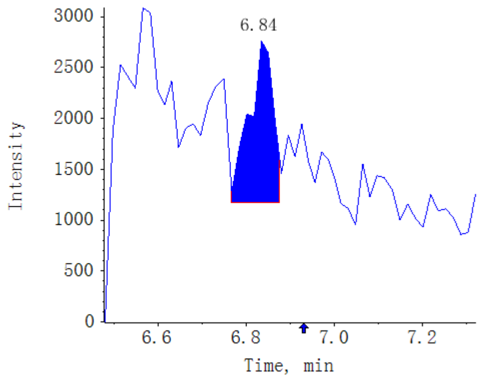

T24186683b_b

GA9 AREA:6.54e3 S/N:6.8

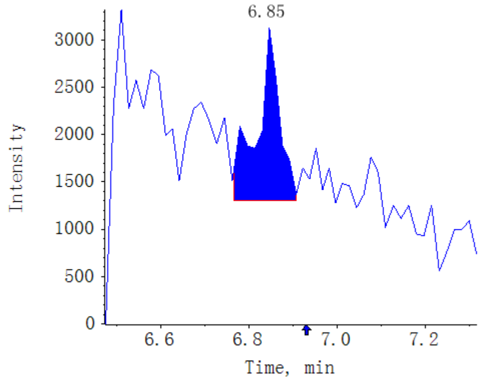

T24186683b_c

GA9 AREA:5.12e3 S/N:5.8

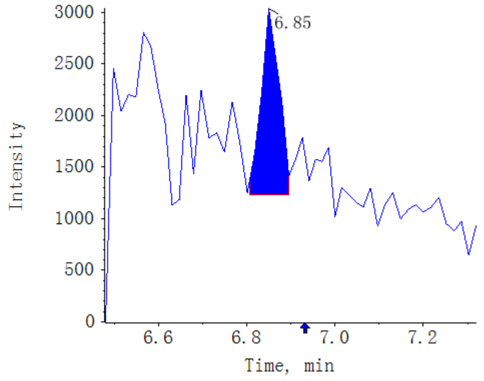

T24186684b_a

GA9 AREA:N/A S/N:N/A

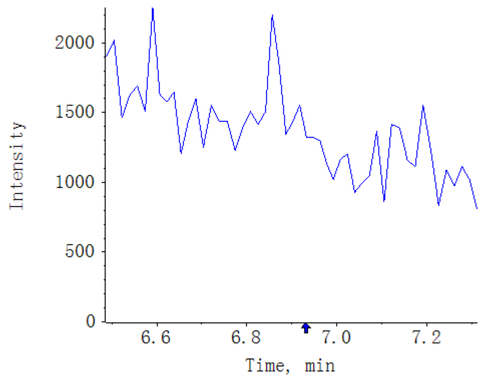

T24186684b_b

GA9 AREA:N/A S/N:N/A

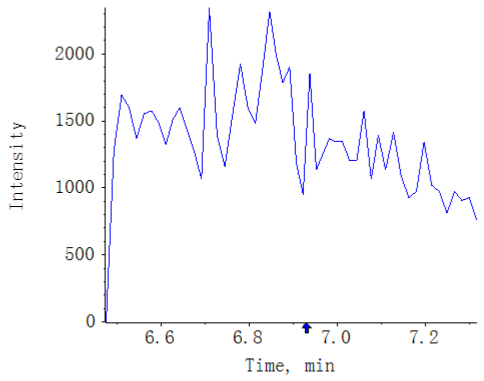

T24186684b_c

GA9 AREA:N/A S/N:N/A

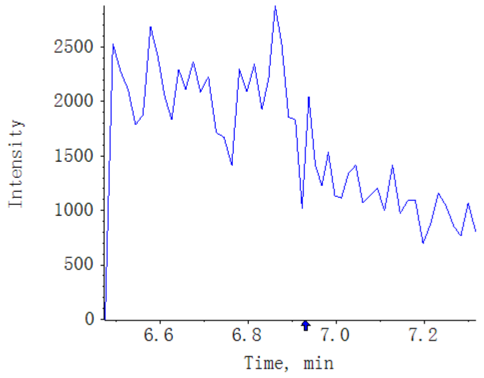

Result Table	MWXS-24-3064-a_9_WH6500-17_A20-3_V6.0_WSS_20240730	Algorithm Used	MQ4
Acquisition Method	ACC-PHs_V6.0_WH6500-17_CMY_20240521.dam	Instrument Name	Triple Quad 6500+ Low Mass
Project	N/A	Analytes QTY	109:93

Compound name: GA44 (345.1 / 301.2)

Sample Name	Sample Type	Area (cps)	Is Area (cps)	RT (min)	S/N	Target Conc	Calculated Conc.()
STD_0.01ppb	Standard	N/A	3.538e5	N/A	N/A	0.0100	N/A
STD_0.05ppb	Standard	N/A	3.397e5	N/A	N/A	0.0500	N/A
STD_0.1ppb	Standard	N/A	3.587e5	N/A	N/A	0.1000	N/A
STD_0.5ppb	Standard	5.77e3	3.275e5	5.42	7.7	0.5000	4.653307e-1
STD_1ppb	Standard	1.47e4	3.746e5	5.42	16.9	1.0000	1.112224e0
STD_5ppb	Standard	6.07e4	3.209e5	5.43	31.2	5.0000	5.562460e0
STD_10ppb	Standard	1.14e5	3.165e5	5.42	56.8	10.0000	1.064428e1
STD_50ppb	Standard	5.86e5	3.582e5	5.41	50.3	50.0000	4.855074e1
STD_100ppb	Standard	9.88e5	3.626e5	5.42	44.5	100.0000	8.091802e1
STD_200ppb	Standard	N/A	3.701e5	N/A	N/A	200.0000	N/A
STD_500ppb	Standard	N/A	4.777e5	N/A	N/A	500.0000	N/A
V2.0_MW_RQC1_20240724	Quality Control	N/A	1.299e5	N/A	N/A	0.0000	N/A
Blank	Unknown	N/A	5.220e2	N/A	N/A	N/A	N/A
V3.0_MWMS_20240725_1	Unknown	6.29e5	3.708e5	5.40	69.4	N/A	5.033214e1
MWXS243064a_R1	Quality Control	N/A	1.034e5	N/A	N/A	0.0000	N/A
MWXS243064a_R2	Quality Control	N/A	1.066e5	N/A	N/A	0.0000	N/A
MWXS243064a_R3	Quality Control	N/A	1.022e5	N/A	N/A	0.0000	N/A
T24186682b_a	Unknown	N/A	8.442e4	N/A	N/A	N/A	N/A
T24186682b_b	Unknown	N/A	7.738e4	N/A	N/A	N/A	N/A
T24186682b_c	Unknown	N/A	9.288e4	N/A	N/A	N/A	N/A
T24186683b_a	Unknown	N/A	1.648e5	N/A	N/A	N/A	N/A
T24186683b_b	Unknown	N/A	1.807e5	N/A	N/A	N/A	N/A
T24186683b_c	Unknown	N/A	1.771e5	N/A	N/A	N/A	N/A
T24186684b_a	Unknown	N/A	9.244e4	N/A	N/A	N/A	N/A
T24186684b_b	Unknown	N/A	1.062e5	N/A	N/A	N/A	N/A
T24186684b_c	Unknown	N/A	1.130e5	N/A	N/A	N/A	N/A

Compound name: GA44
Regression Equation: y = 0.03365 x + 0.00195 (r = 0.99074) (weighting: 1 / x^2)

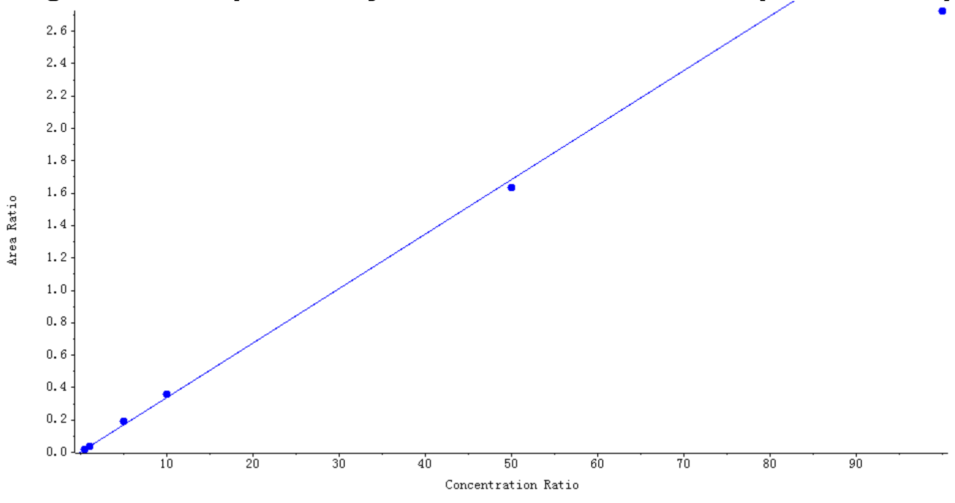

Peak Review

Blank
GA44 AREA:N/A S/N:N/A

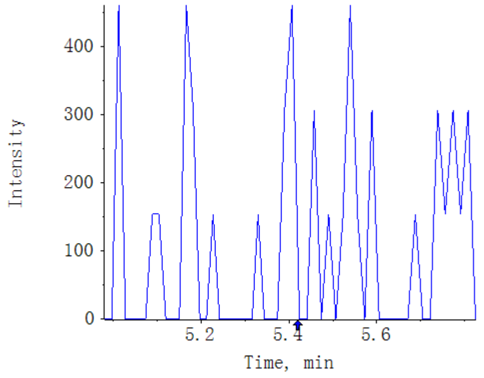

V3.0_MWMS_20240725_1
GA44 AREA:6.29e5 S/N:69.4

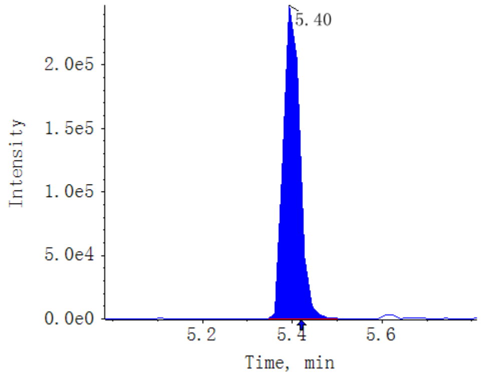

T24186682b_a
GA44 AREA:N/A S/N:N/A

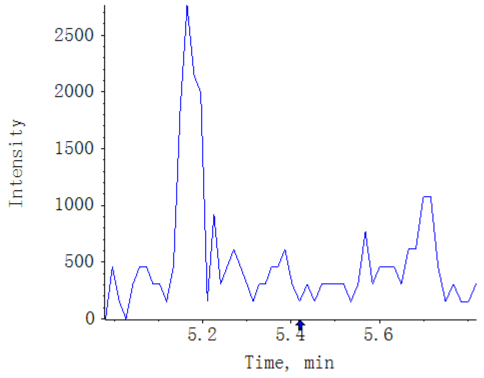

T24186682b_b
GA44 AREA:N/A S/N:N/A

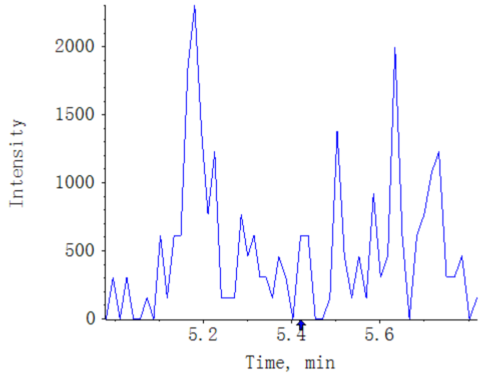

T24186682b_c
GA44 AREA:N/A S/N:N/A

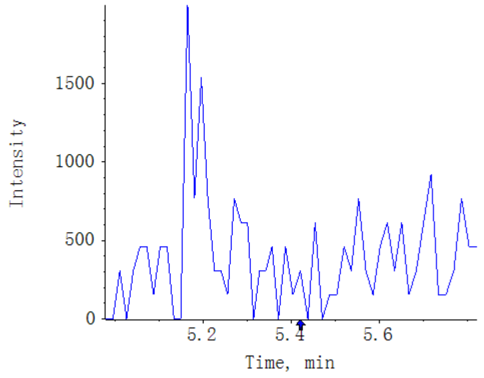

T24186683b_a
GA44 AREA:N/A S/N:N/A

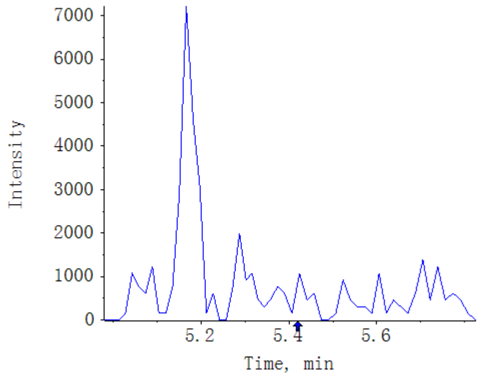

T24186683b_b
GA44 AREA:N/A S/N:N/A

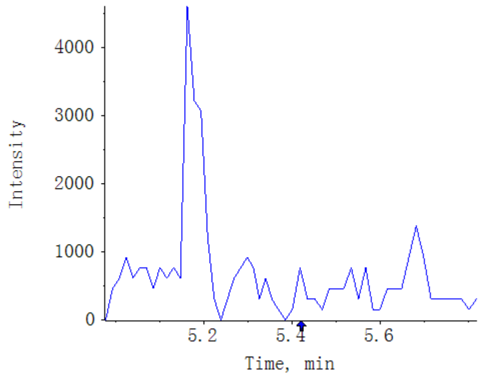

T24186683b_c
GA44 AREA:N/A S/N:N/A

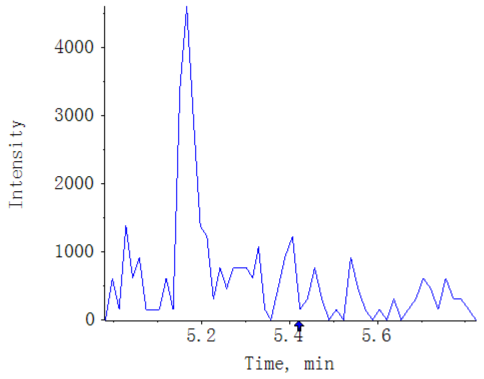

T24186684b_a
GA44 AREA:N/A S/N:N/A

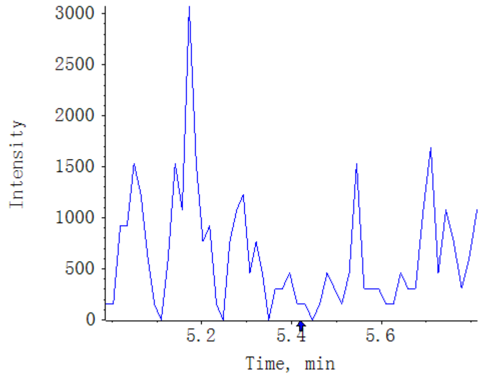

T24186684b_b
GA44 AREA:N/A S/N:N/A

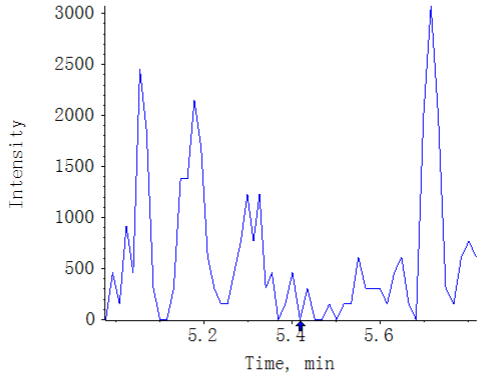

T24186684b_c
GA44 AREA:N/A S/N:N/A

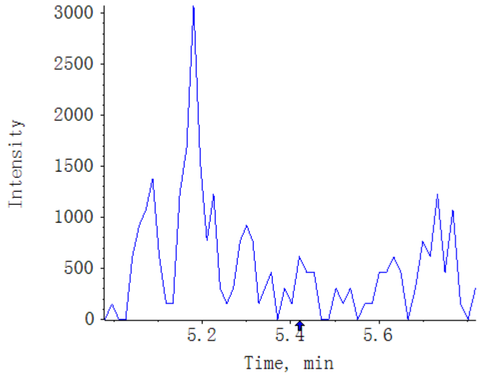

Result Table	MWXS-24-3064-a_9_WH6500-17_A20-3_V6.0_WSS_20240730	Algorithm Used	MQ4
Acquisition Method	ACC-PHs_V6.0_WH6500-17_CMY_20240521.dam	Instrument Name	Triple Quad 6500+ Low Mass
Project	N/A	Analytes QTY	109:94

Compound name: GA8 (363.1 / 275.2)

Sample Name	Sample Type	Area (cps)	Is Area (cps)	RT (min)	S/N	Target Conc	Calculated Conc.()
STD_0.01ppb	Standard	N/A	3.538e5	N/A	N/A	0.0100	N/A
STD_0.05ppb	Standard	2.93e3	3.397e5	3.81	8.0	0.0500	5.004120e-2
STD_0.1ppb	Standard	5.01e3	3.587e5	3.82	14.2	0.1000	9.764978e-2
STD_0.5ppb	Standard	2.26e4	3.275e5	3.81	45.7	0.5000	5.884068e-1
STD_1ppb	Standard	3.60e4	3.746e5	3.81	50.5	1.0000	8.318408e-1
STD_5ppb	Standard	2.09e5	3.209e5	3.82	43.4	5.0000	5.801408e0
STD_10ppb	Standard	3.64e5	3.165e5	3.81	45.9	10.0000	1.024544e1
STD_50ppb	Standard	1.98e6	3.582e5	3.81	64.6	50.0000	4.928965e1
STD_100ppb	Standard	3.76e6	3.626e5	3.82	44.0	100.0000	9.262650e1
STD_200ppb	Standard	7.60e6	3.701e5	3.81	53.9	200.0000	1.834280e2
STD_500ppb	Standard	N/A	4.777e5	N/A	N/A	500.0000	N/A
V2.0_MW_RQC1_20240724	Quality Control	N/A	1.299e5	N/A	N/A	0.0000	N/A
Blank	Unknown	N/A	5.220e2	N/A	N/A	N/A	N/A
V3.0_MWMS_20240725_1	Unknown	2.29e6	3.708e5	3.82	51.2	N/A	5.524531e1
MWXS243064a_R1	Quality Control	N/A	1.034e5	N/A	N/A	0.0000	N/A
MWXS243064a_R2	Quality Control	N/A	1.066e5	N/A	N/A	0.0000	N/A
MWXS243064a_R3	Quality Control	N/A	1.022e5	N/A	N/A	0.0000	N/A
T24186682b_a	Unknown	N/A	8.442e4	N/A	N/A	N/A	N/A
T24186682b_b	Unknown	N/A	7.738e4	N/A	N/A	N/A	N/A
T24186682b_c	Unknown	N/A	9.288e4	N/A	N/A	N/A	N/A
T24186683b_a	Unknown	N/A	1.648e5	N/A	N/A	N/A	N/A
T24186683b_b	Unknown	N/A	1.807e5	N/A	N/A	N/A	N/A
T24186683b_c	Unknown	N/A	1.771e5	N/A	N/A	N/A	N/A
T24186684b_a	Unknown	N/A	9.244e4	N/A	N/A	N/A	N/A
T24186684b_b	Unknown	N/A	1.062e5	N/A	N/A	N/A	N/A
T24186684b_c	Unknown	N/A	1.130e5	N/A	N/A	N/A	N/A

Compound name: GA8
Regression Equation: y = 0.11196 x + 0.00302 (r = 0.99281) (weighting: 1 / x^2)

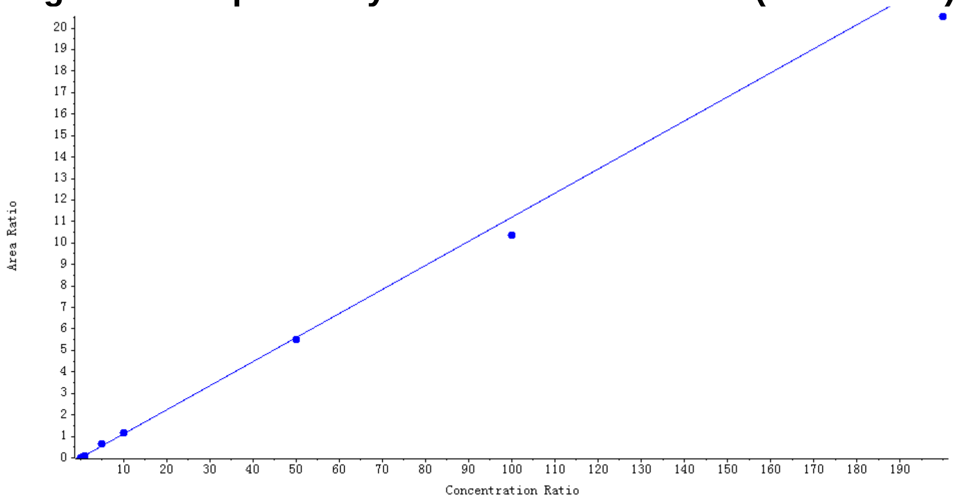

Peak Review

Blank

GA8 AREA:N/A S/N:N/A

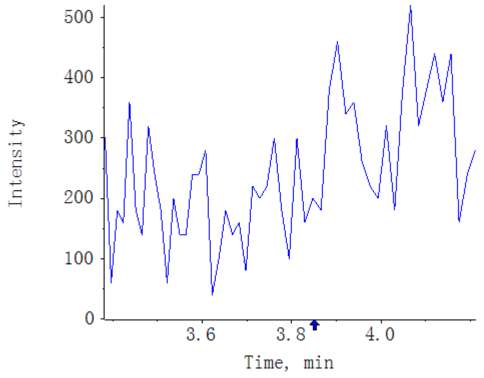

V3.0_MWMS_20240725_1

GA8 AREA:2.29e6 S/N:51.2

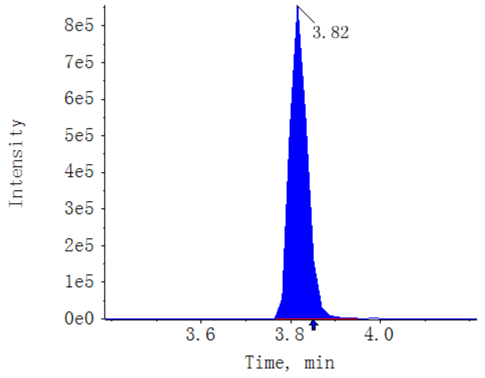

T24186682b_a

GA8 AREA:N/A S/N:N/A

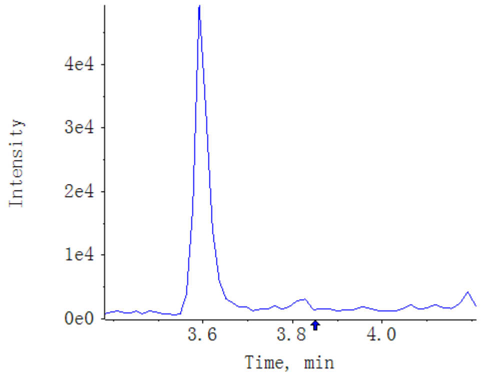

T24186682b_b

GA8 AREA:N/A S/N:N/A

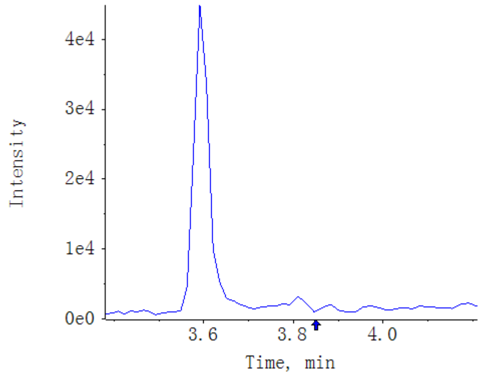

T24186682b_c

GA8 AREA:N/A S/N:N/A

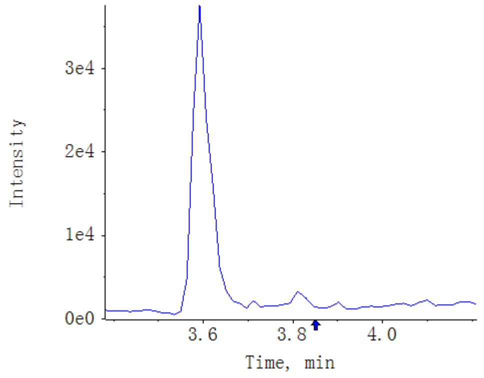

T24186683b_a

GA8 AREA:N/A S/N:N/A

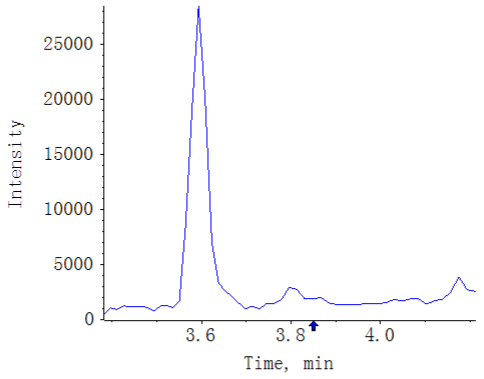

T24186683b_b

GA8 AREA:N/A S/N:N/A

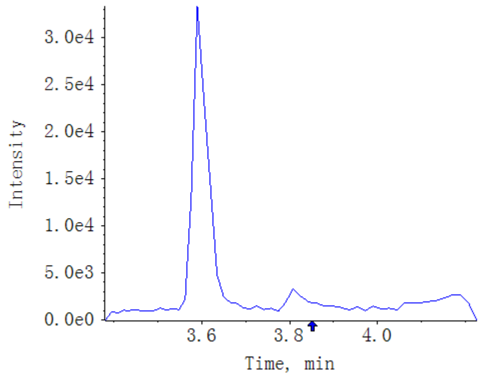

T24186683b_c

GA8 AREA:N/A S/N:N/A

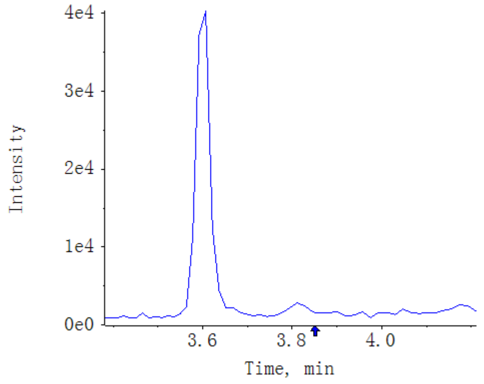

T24186684b_a

GA8 AREA:N/A S/N:N/A

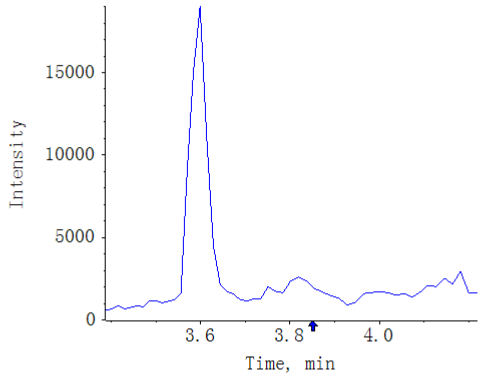

T24186684b_b

GA8 AREA:N/A S/N:N/A

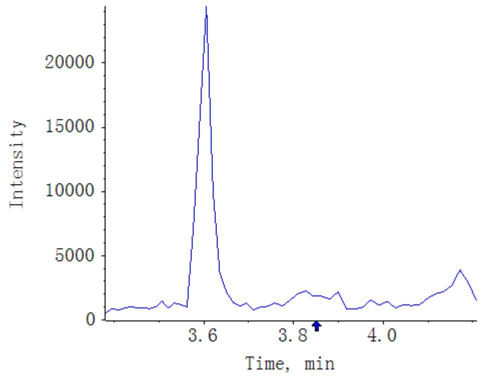

T24186684b_c

GA8 AREA:N/A S/N:N/A

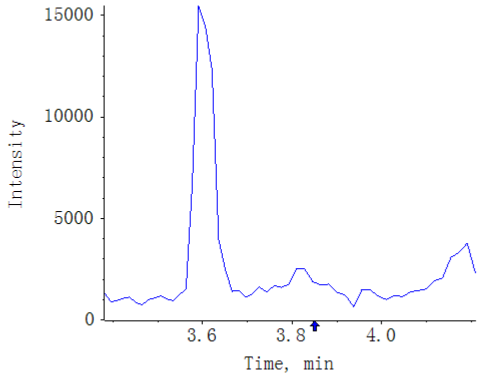

Result Table	MWXS-24-3064-a_9_WH6500-17_A20-3_V6.0_WSS_20240730	Algorithm Used	MQ4
Acquisition Method	ACC-PHs_V6.0_WH6500-17_CMY_20240521.dam	Instrument Name	Triple Quad 6500+ Low Mass
Project	N/A	Analytes QTY	109:95

Compound name: GA5 (329.1 / 145.0)

Sample Name	Sample Type	Area (cps)	Is Area (cps)	RT (min)	S/N	Target Conc	Calculated Conc.()
STD_0.01ppb	Standard	N/A	3.538e5	N/A	N/A	0.0100	N/A
STD_0.05ppb	Standard	N/A	3.397e5	N/A	N/A	0.0500	N/A
STD_0.1ppb	Standard	N/A	3.587e5	N/A	N/A	0.1000	N/A
STD_0.5ppb	Standard	1.04e4	3.275e5	5.31	26.9	0.5000	5.545207e-1
STD_1ppb	Standard	1.51e4	3.746e5	5.31	22.9	1.0000	7.570723e-1
STD_5ppb	Standard	7.71e4	3.209e5	5.32	31.2	5.0000	5.504255e0
STD_10ppb	Standard	1.41e5	3.165e5	5.31	38.7	10.0000	1.040274e1
STD_50ppb	Standard	8.01e5	3.582e5	5.30	42.4	50.0000	5.282813e1
STD_100ppb	Standard	1.48e6	3.626e5	5.31	52.6	100.0000	9.670091e1
STD_200ppb	Standard	3.03e6	3.701e5	5.31	41.2	200.0000	1.938380e2
STD_500ppb	Standard	N/A	4.777e5	N/A	N/A	500.0000	N/A
V2.0_MW_RQC1_20240724	Quality Control	N/A	1.299e5	N/A	N/A	0.0000	N/A
Blank	Unknown	N/A	5.220e2	N/A	N/A	N/A	N/A
V3.0_MWMS_20240725_1	Unknown	8.95e5	3.708e5	5.28	43.9	N/A	5.701867e1
MWXS243064a_R1	Quality Control	N/A	1.034e5	N/A	N/A	0.0000	N/A
MWXS243064a_R2	Quality Control	N/A	1.066e5	N/A	N/A	0.0000	N/A
MWXS243064a_R3	Quality Control	N/A	1.022e5	N/A	N/A	0.0000	N/A
T24186682b_a	Unknown	N/A	8.442e4	N/A	N/A	N/A	N/A
T24186682b_b	Unknown	N/A	7.738e4	N/A	N/A	N/A	N/A
T24186682b_c	Unknown	N/A	9.288e4	N/A	N/A	N/A	N/A
T24186683b_a	Unknown	N/A	1.648e5	N/A	N/A	N/A	N/A
T24186683b_b	Unknown	N/A	1.807e5	N/A	N/A	N/A	N/A
T24186683b_c	Unknown	N/A	1.771e5	N/A	N/A	N/A	N/A
T24186684b_a	Unknown	N/A	9.244e4	N/A	N/A	N/A	N/A
T24186684b_b	Unknown	N/A	1.062e5	N/A	N/A	N/A	N/A
T24186684b_c	Unknown	N/A	1.130e5	N/A	N/A	N/A	N/A

Compound name: GA5
Regression Equation: y = 0.04217 x + 0.00831 (r = 0.99096) (weighting: 1 / x^2)

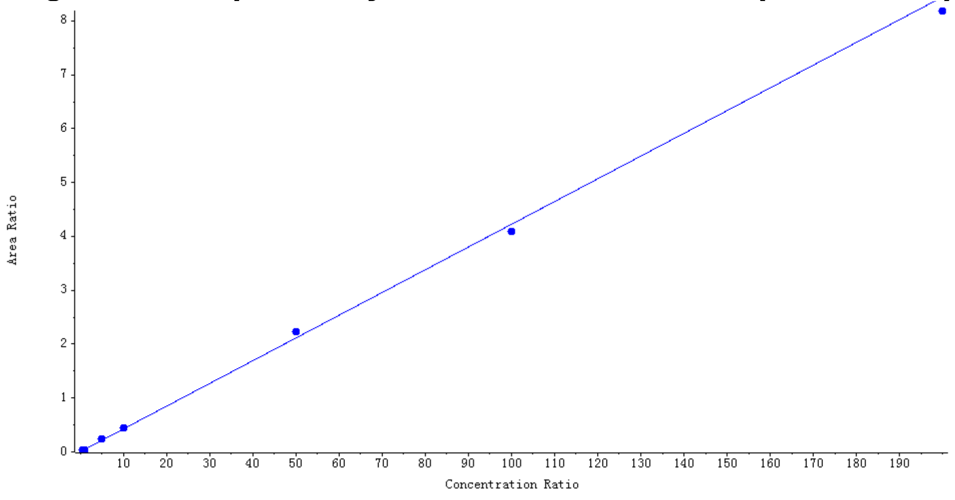

Peak Review

Blank
GA5 AREA:N/A S/N:N/A

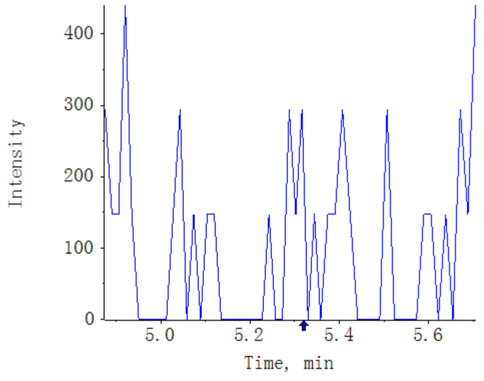

V3.0_MWMS_20240725_1
GA5 AREA:8.95e5 S/N:43.9

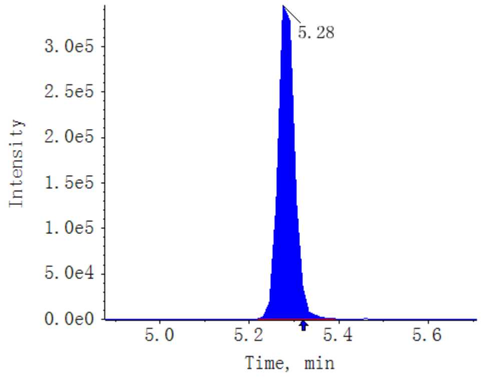

T24186682b_a
GA5 AREA:N/A S/N:N/A

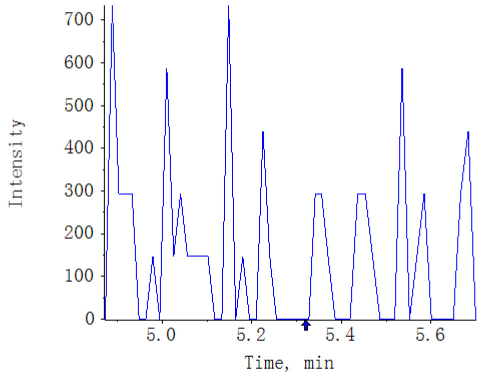

T24186682b_b
GA5 AREA:N/A S/N:N/A

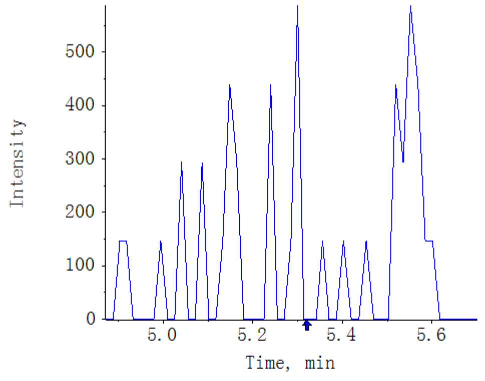

T24186682b_c
GA5 AREA:N/A S/N:N/A

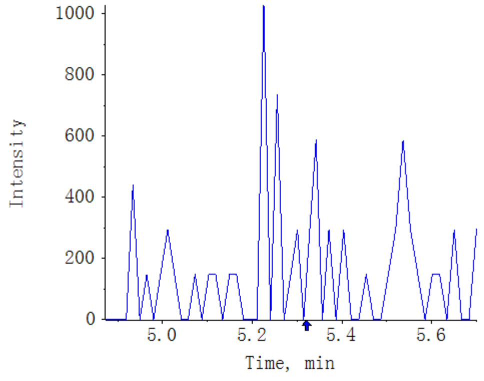

T24186683b_a
GA5 AREA:N/A S/N:N/A

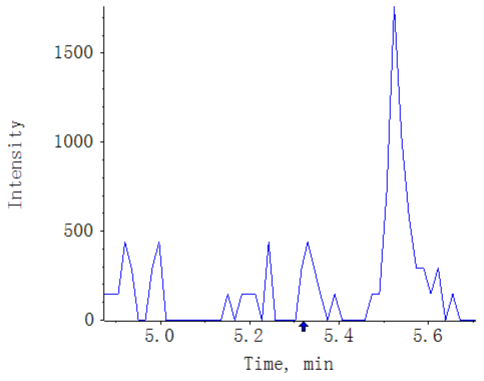

T24186683b_b
GA5 AREA:N/A S/N:N/A

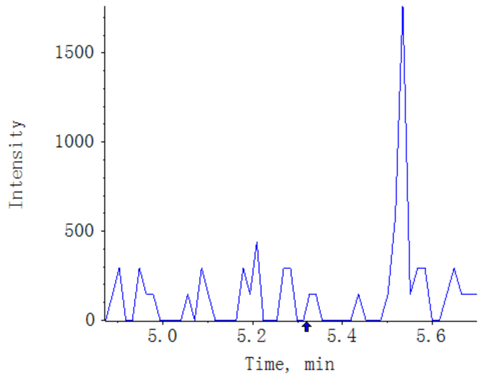

T24186683b_c
GA5 AREA:N/A S/N:N/A

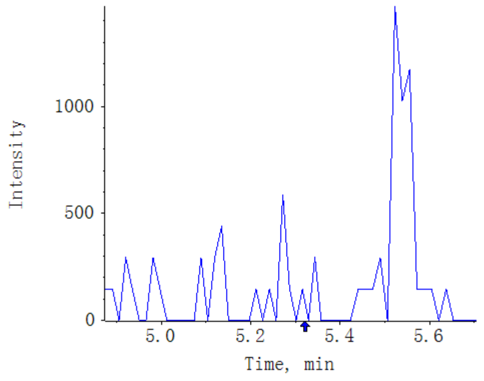

T24186684b_a
GA5 AREA:N/A S/N:N/A

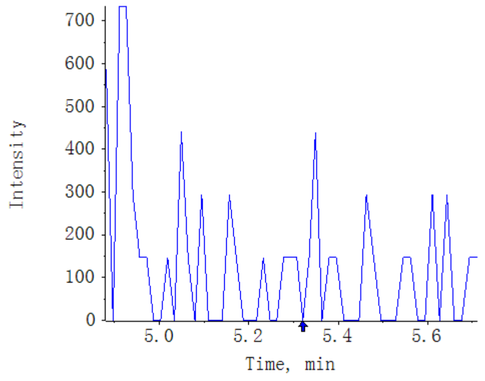

T24186684b_b
GA5 AREA:N/A S/N:N/A

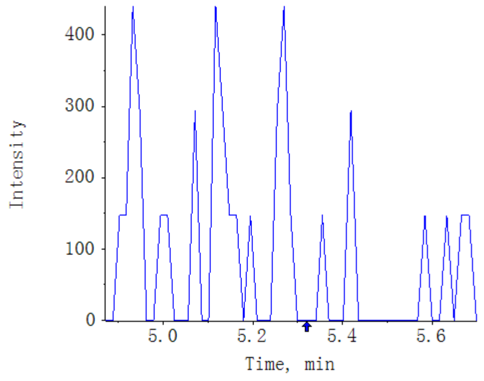

T24186684b_c
GA5 AREA:N/A S/N:N/A

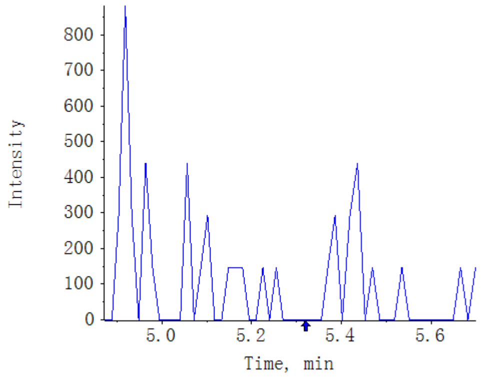

Result Table	MWXS-24-3064-a_9_WH6500-17_A20-3_V6.0_WSS_20240730	Algorithm Used	MQ4
Acquisition Method	ACC-PHs_V6.0_WH6500-17_CMY_20240521.dam	Instrument Name	Triple Quad 6500+ Low Mass
Project	N/A	Analytes QTY	109:96

Compound name: GA6 (345.2 / 257.1)

Sample Name	Sample Type	Area (cps)	Is Area (cps)	RT (min)	S/N	Target Conc	Calculated Conc.()
STD_0.01ppb	Standard	N/A	3.538e5	N/A	N/A	0.0100	N/A
STD_0.05ppb	Standard	N/A	3.397e5	N/A	N/A	0.0500	N/A
STD_0.1ppb	Standard	6.08e3	3.587e5	4.92	7.2	0.1000	9.858712e-2
STD_0.5ppb	Standard	2.61e4	3.275e5	4.90	23.2	0.5000	5.715736e-1
STD_1ppb	Standard	4.22e4	3.746e5	4.91	37.5	1.0000	8.186963e-1
STD_5ppb	Standard	2.49e5	3.209e5	4.92	38.0	5.0000	5.817554e0
STD_10ppb	Standard	4.43e5	3.165e5	4.90	40.6	10.0000	1.049242e1
STD_50ppb	Standard	2.36e6	3.582e5	4.90	58.3	50.0000	4.950765e1
STD_100ppb	Standard	4.46e6	3.626e5	4.91	41.6	100.0000	9.242657e1
STD_200ppb	Standard	9.10e6	3.701e5	4.91	61.9	200.0000	1.850228e2
STD_500ppb	Standard	N/A	4.777e5	N/A	N/A	500.0000	N/A
V2.0_MW_RQC1_20240724	Quality Control	N/A	1.299e5	N/A	N/A	0.0000	N/A
Blank	Unknown	N/A	5.220e2	N/A	N/A	N/A	N/A
V3.0_MWMS_20240725_1	Unknown	2.49e6	3.708e5	4.89	57.2	N/A	5.050442e1
MWXS243064a_R1	Quality Control	N/A	1.034e5	N/A	N/A	0.0000	N/A
MWXS243064a_R2	Quality Control	N/A	1.066e5	N/A	N/A	0.0000	N/A
MWXS243064a_R3	Quality Control	N/A	1.022e5	N/A	N/A	0.0000	N/A
T24186682b_a	Unknown	N/A	8.442e4	N/A	N/A	N/A	N/A
T24186682b_b	Unknown	N/A	7.738e4	N/A	N/A	N/A	N/A
T24186682b_c	Unknown	N/A	9.288e4	N/A	N/A	N/A	N/A
T24186683b_a	Unknown	N/A	1.648e5	N/A	N/A	N/A	N/A
T24186683b_b	Unknown	N/A	1.807e5	N/A	N/A	N/A	N/A
T24186683b_c	Unknown	N/A	1.771e5	N/A	N/A	N/A	N/A
T24186684b_a	Unknown	N/A	9.244e4	N/A	N/A	N/A	N/A
T24186684b_b	Unknown	N/A	1.062e5	N/A	N/A	N/A	N/A
T24186684b_c	Unknown	N/A	1.130e5	N/A	N/A	N/A	N/A

Compound name: GA6
Regression Equation: y = 0.13290 x + 0.00385 (r = 0.99262) (weighting: 1 / x^2)

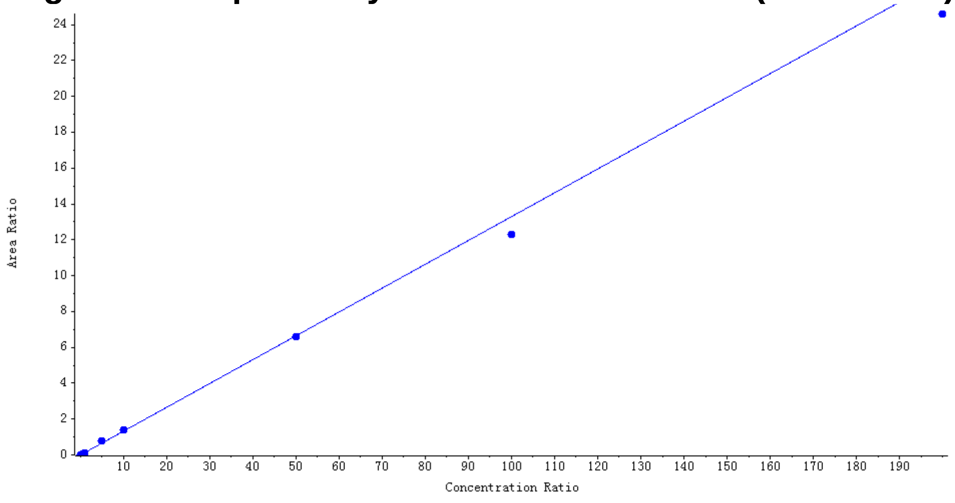

Peak Review

Blank

GA6 AREA:N/A S/N:N/A

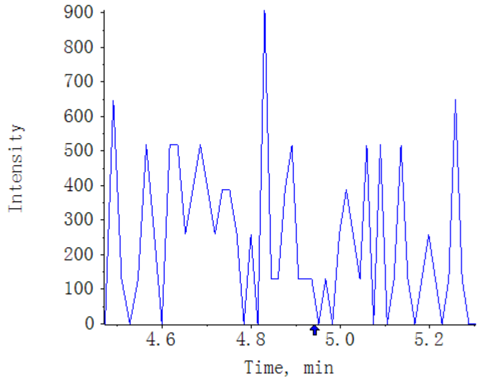

V3.0_MWMS_20240725_1

GA6 AREA:2.49e6 S/N:57.2

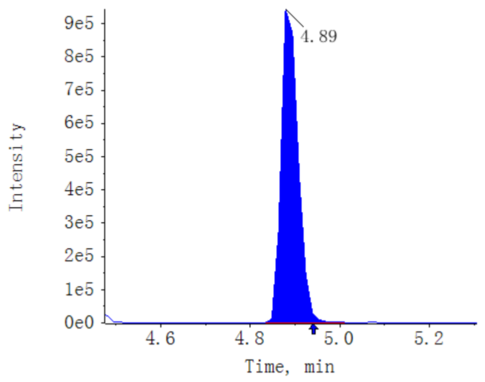

T24186682b_a

GA6 AREA:N/A S/N:N/A

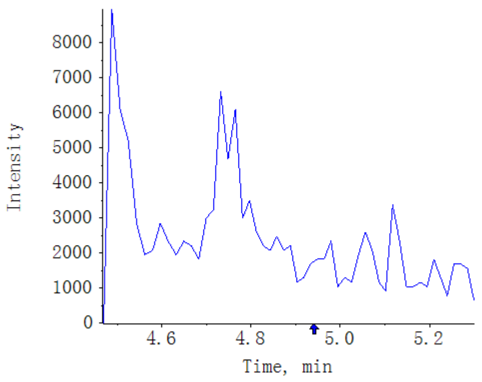

T24186682b_b

GA6 AREA:N/A S/N:N/A

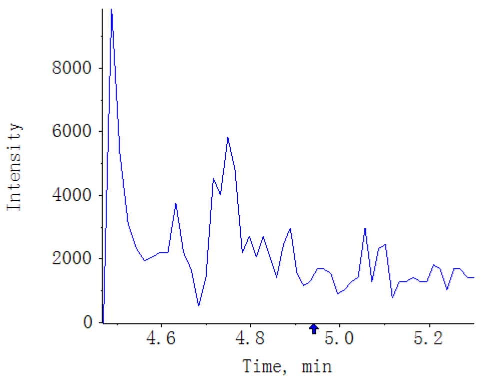

T24186682b_c

GA6 AREA:N/A S/N:N/A

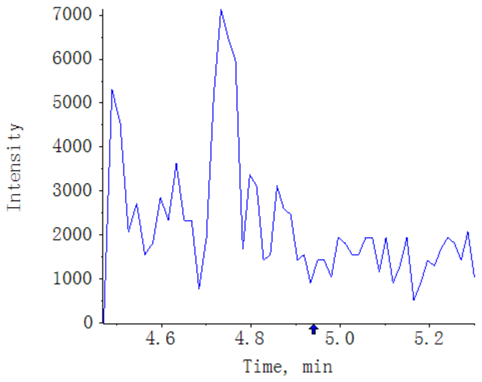

T24186683b_a

GA6 AREA:N/A S/N:N/A

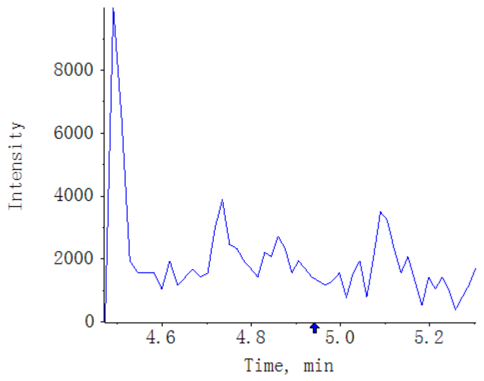

T24186683b_b

GA6 AREA:N/A S/N:N/A

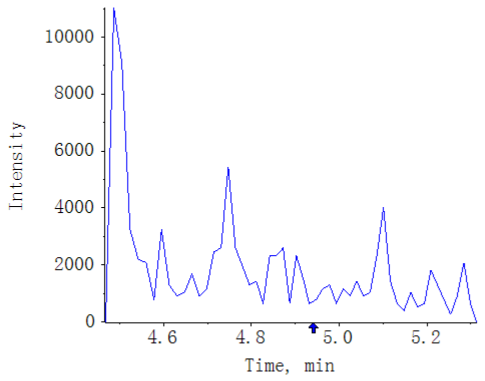

T24186683b_c

GA6 AREA:N/A S/N:N/A

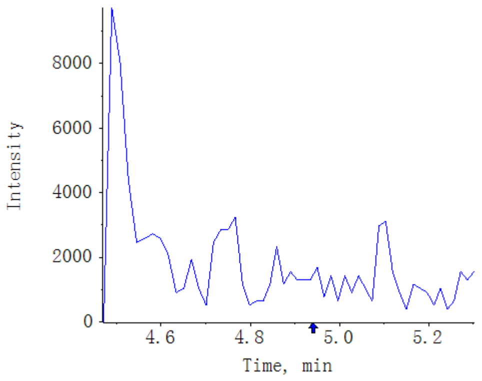

T24186684b_a

GA6 AREA:N/A S/N:N/A

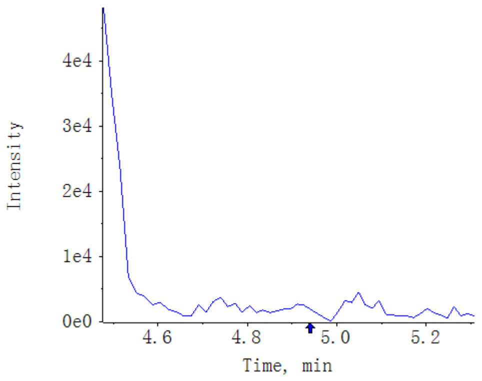

T24186684b_b

GA6 AREA:N/A S/N:N/A

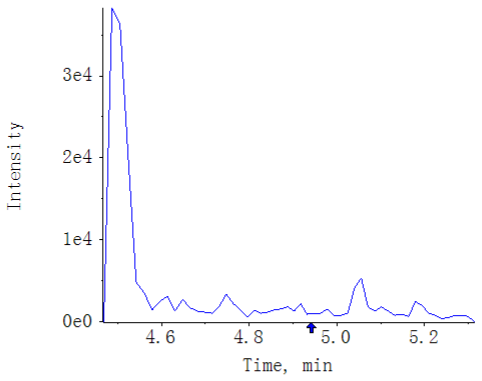

T24186684b_c

GA6 AREA:N/A S/N:N/A

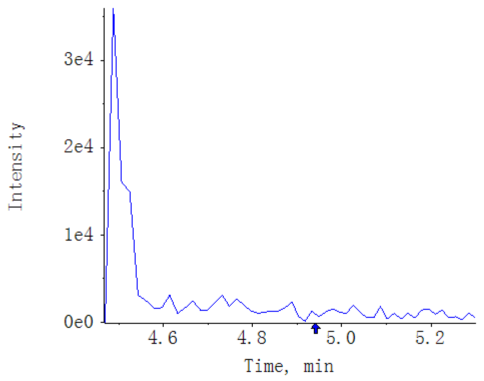

Result Table	MWXS-24-3064-a_9_WH6500-17_A20-3_V6.0_WSS_20240730	Algorithm Used	MQ4
Acquisition Method	ACC-PHs_V6.0_WH6500-17_CMY_20240521.dam	Instrument Name	Triple Quad 6500+ Low Mass
Project	N/A	Analytes QTY	109:97

Compound name: GA29 (347.2 / 259.1)

Sample Name	Sample Type	Area (cps)	Is Area (cps)	RT (min)	S/N	Target Conc	Calculated Conc.()
STD_0.01ppb	Standard	N/A	3.538e5	N/A	N/A	0.0100	N/A
STD_0.05ppb	Standard	N/A	3.397e5	N/A	N/A	0.0500	N/A
STD_0.1ppb	Standard	N/A	3.587e5	N/A	N/A	0.1000	N/A
STD_0.5ppb	Standard	6.97e2	3.275e5	3.98	6.0	0.5000	4.996675e-1
STD_1ppb	Standard	1.70e3	3.746e5	3.98	4.3	1.0000	9.657945e-1
STD_5ppb	Standard	1.00e4	3.209e5	4.00	17.8	5.0000	6.090990e0
STD_10ppb	Standard	1.51e4	3.165e5	3.98	23.7	10.0000	9.287781e0
STD_50ppb	Standard	9.20e4	3.582e5	3.98	64.5	50.0000	4.954352e1
STD_100ppb	Standard	1.77e5	3.626e5	3.99	53.4	100.0000	9.431248e1
STD_200ppb	Standard	3.67e5	3.701e5	3.98	58.2	200.0000	1.907798e2
STD_500ppb	Standard	N/A	4.777e5	N/A	N/A	500.0000	N/A
V2.0_MW_RQC1_20240724	Quality Control	N/A	1.299e5	N/A	N/A	0.0000	N/A
Blank	Unknown	N/A	5.220e2	N/A	N/A	N/A	N/A
V3.0_MWMS_20240725_1	Unknown	7.67e4	3.708e5	3.98	2.4	N/A	3.992009e1
MWXS243064a_R1	Quality Control	1.24e4	1.034e5	4.06	7.9	0.0000	2.325961e1
MWXS243064a_R2	Quality Control	1.19e4	1.066e5	4.08	6.0	0.0000	2.166180e1
MWXS243064a_R3	Quality Control	1.13e4	1.022e5	4.05	9.0	0.0000	2.141080e1
T24186682b_a	Unknown	8.66e3	8.442e4	4.05	4.4	N/A	1.984886e1
T24186682b_b	Unknown	8.15e3	7.738e4	4.05	5.8	N/A	2.037570e1
T24186682b_c	Unknown	9.23e3	9.288e4	4.03	6.6	N/A	1.922574e1
T24186683b_a	Unknown	1.61e4	1.648e5	4.07	9.8	N/A	1.883644e1
T24186683b_b	Unknown	1.49e4	1.807e5	4.05	7.9	N/A	1.596664e1
T24186683b_c	Unknown	1.45e4	1.771e5	4.08	10.3	N/A	1.582517e1
T24186684b_a	Unknown	1.30e4	9.244e4	4.05	7.7	N/A	2.711506e1
T24186684b_b	Unknown	1.40e4	1.062e5	4.08	6.9	N/A	2.545711e1
T24186684b_c	Unknown	1.65e4	1.130e5	4.08	8.2	N/A	2.812034e1

Compound name: GA29
Regression Equation: y = 0.00519 x + -4.66344e-4 (r = 0.99388) (weighting: 1 / x^2)

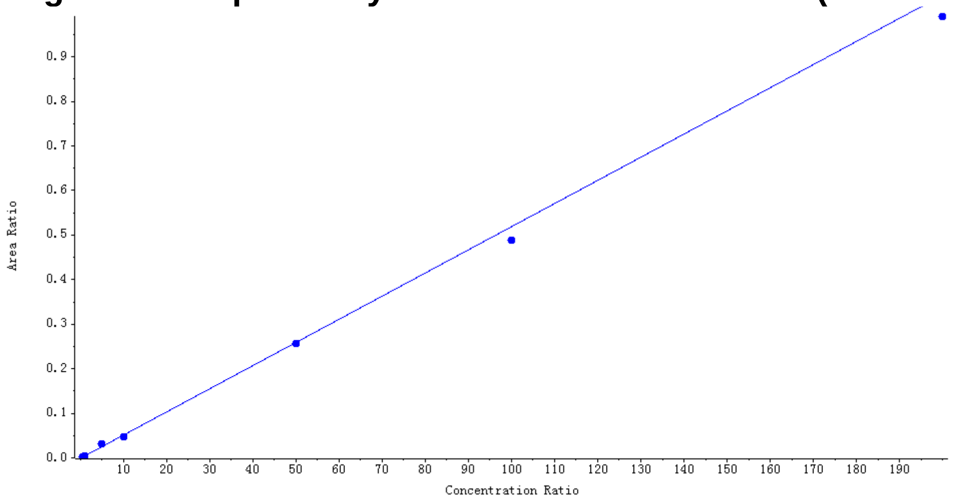

Peak Review

Blank
GA29 AREA:N/A S/N:N/A

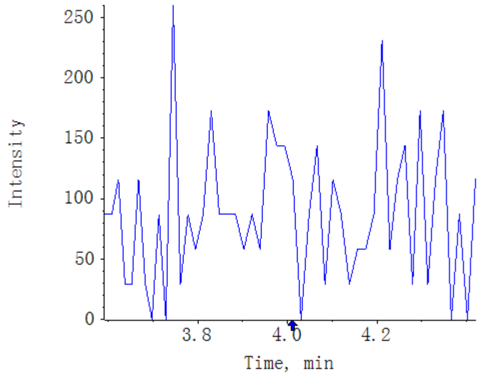

V3.0_MWMS_20240725_1
GA29 AREA:7.67e4 S/N:2.4

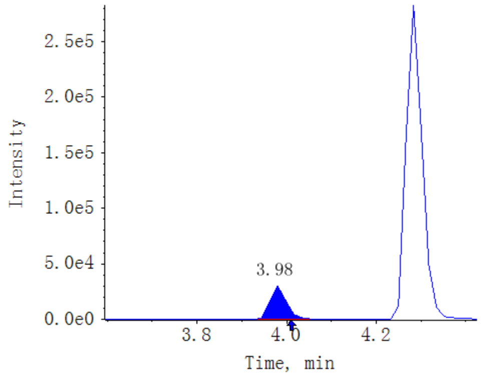

T24186682b_a
GA29 AREA:8.66e3 S/N:4.4

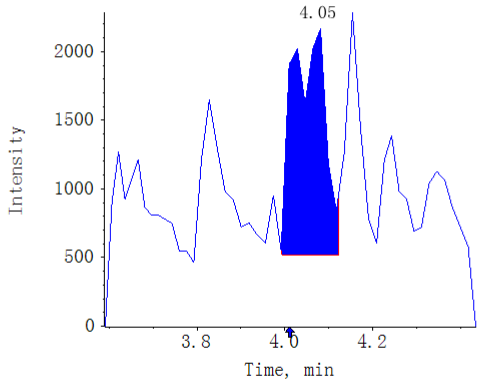

T24186682b_b
GA29 AREA:8.15e3 S/N:5.8

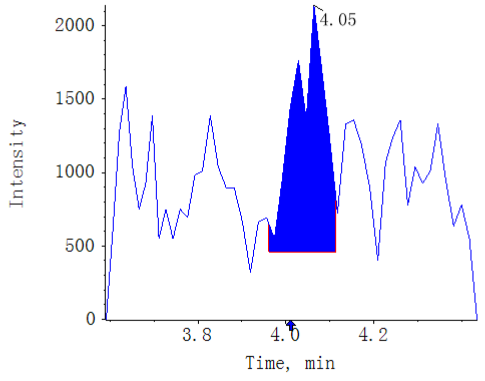

T24186682b_c
GA29 AREA:9.23e3 S/N:6.6

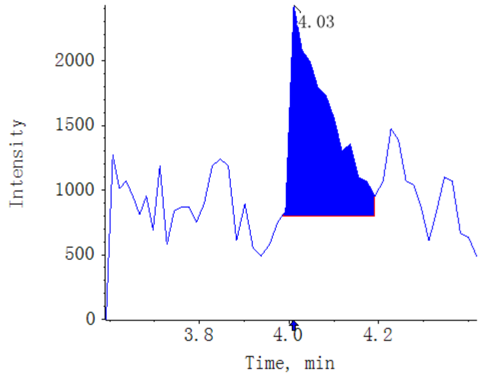

T24186683b_a
GA29 AREA:1.61e4 S/N:9.8

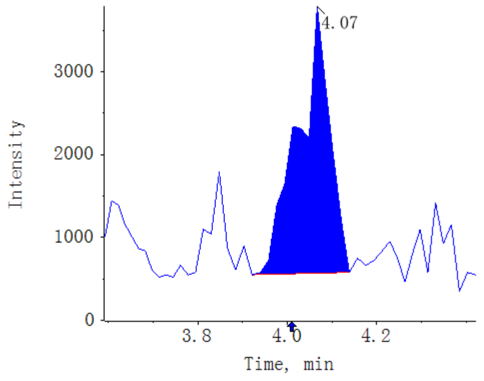

T24186683b_b
GA29 AREA:1.49e4 S/N:7.9

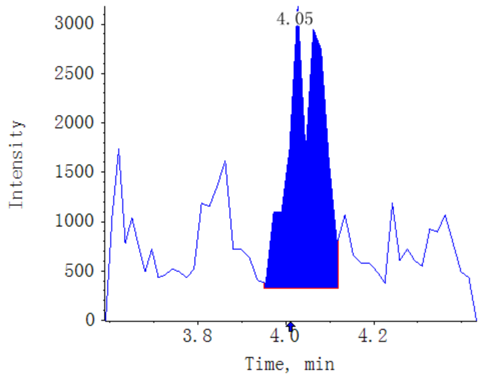

T24186683b_c
GA29 AREA:1.45e4 S/N:10.3

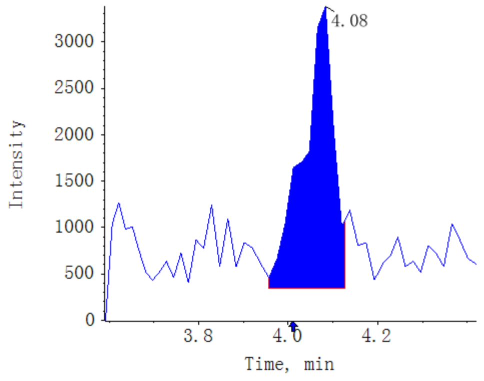

T24186684b_a
GA29 AREA:1.30e4 S/N:7.7

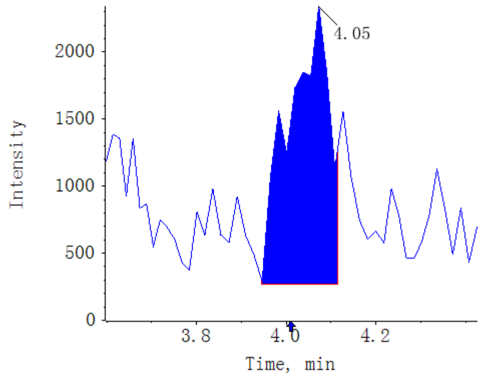

T24186684b_b
GA29 AREA:1.40e4 S/N:6.9

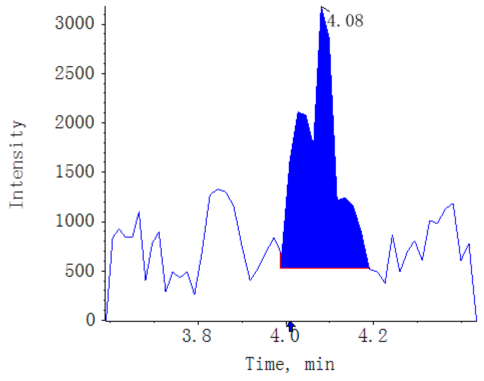

T24186684b_c
GA29 AREA:1.65e4 S/N:8.2

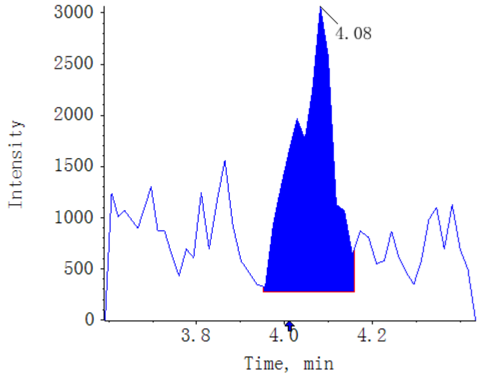

Result Table	MWXS-24-3064-a_9_WH6500-17_A20-3_V6.0_WSS_20240730	Algorithm Used	MQ4
Acquisition Method	ACC-PHs_V6.0_WH6500-17_CMY_20240521.dam	Instrument Name	Triple Quad 6500+ Low Mass
Project	N/A	Analytes QTY	109:98

Compound name: GA34 (347.2 / 241.3)

Sample Name	Sample Type	Area (cps)	Is Area (cps)	RT (min)	S/N	Target Conc	Calculated Conc.()
STD_0.01ppb	Standard	N/A	3.538e5	N/A	N/A	0.0100	N/A
STD_0.05ppb	Standard	1.91e3	3.397e5	5.58	5.9	0.0500	4.460079e-2
STD_0.1ppb	Standard	6.33e3	3.587e5	5.59	14.3	0.1000	1.221900e-1
STD_0.5ppb	Standard	2.64e4	3.275e5	5.58	20.3	0.5000	5.278342e-1
STD_1ppb	Standard	4.60e4	3.746e5	5.58	22.1	1.0000	8.003940e-1
STD_5ppb	Standard	2.80e5	3.209e5	5.59	32.8	5.0000	5.634130e0
STD_10ppb	Standard	5.13e5	3.165e5	5.58	40.2	10.0000	1.046837e1
STD_50ppb	Standard	2.77e6	3.582e5	5.57	34.9	50.0000	4.982618e1
STD_100ppb	Standard	5.25e6	3.626e5	5.59	39.9	100.0000	9.340925e1
STD_200ppb	Standard	1.06e7	3.701e5	5.58	31.7	200.0000	1.851484e2
STD_500ppb	Standard	N/A	4.777e5	N/A	N/A	500.0000	N/A
V2.0_MW_RQC1_20240724	Quality Control	N/A	1.299e5	N/A	N/A	0.0000	N/A
Blank	Unknown	N/A	5.220e2	N/A	N/A	N/A	N/A
V3.0_MWMS_20240725_1	Unknown	2.46e6	3.708e5	5.55	26.6	N/A	4.282719e1
MWXS243064a_R1	Quality Control	3.06e4	1.034e5	5.54	16.1	0.0000	1.917387e0
MWXS243064a_R2	Quality Control	3.79e4	1.066e5	5.55	19.3	0.0000	2.300744e0
MWXS243064a_R3	Quality Control	2.69e4	1.022e5	5.54	14.2	0.0000	1.705974e0
T24186682b_a	Unknown	1.75e4	8.442e4	5.56	8.6	N/A	1.342011e0
T24186682b_b	Unknown	1.79e4	7.738e4	5.57	10.9	N/A	1.500790e0
T24186682b_c	Unknown	1.70e4	9.288e4	5.57	9.9	N/A	1.186801e0
T24186683b_a	Unknown	5.41e4	1.648e5	5.54	19.1	N/A	2.126461e0
T24186683b_b	Unknown	5.18e4	1.807e5	5.54	19.1	N/A	1.858695e0
T24186683b_c	Unknown	4.91e4	1.771e5	5.55	17.5	N/A	1.796011e0
T24186684b_a	Unknown	3.20e4	9.244e4	5.55	25.9	N/A	2.240524e0
T24186684b_b	Unknown	3.06e4	1.062e5	5.56	24.6	N/A	1.868470e0
T24186684b_c	Unknown	2.73e4	1.130e5	5.55	31.0	N/A	1.566291e0

Compound name: GA34
Regression Equation: y = 0.15507 x + -0.00131 (r = 0.99043) (weighting: 1 / x^2)

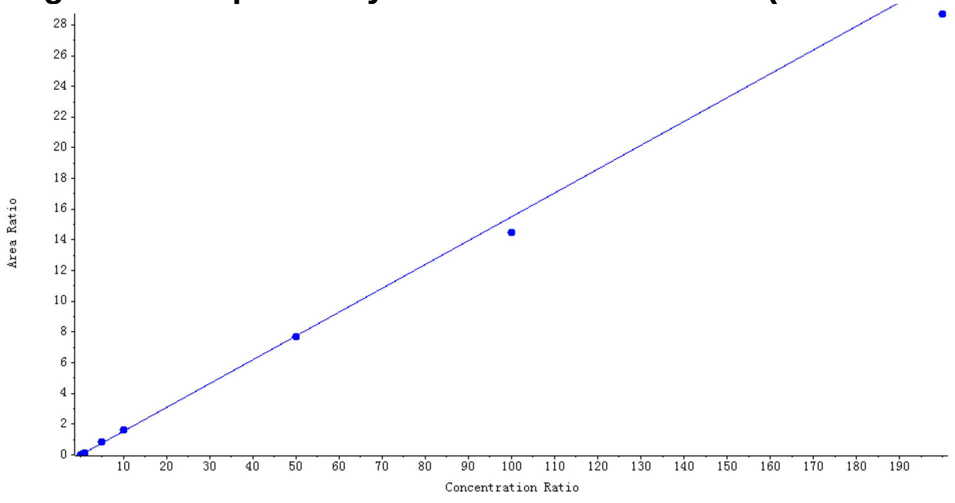

Peak Review

Blank
GA34 AREA:N/A S/N:N/A

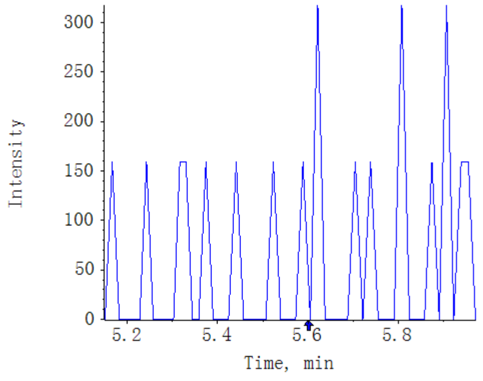

V3.0_MWMS_20240725_1
GA34 AREA:2.46e6 S/N:26.6

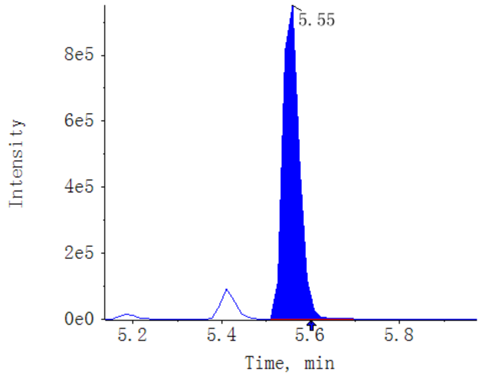

T24186682b_a
GA34 AREA:1.75e4 S/N:8.6

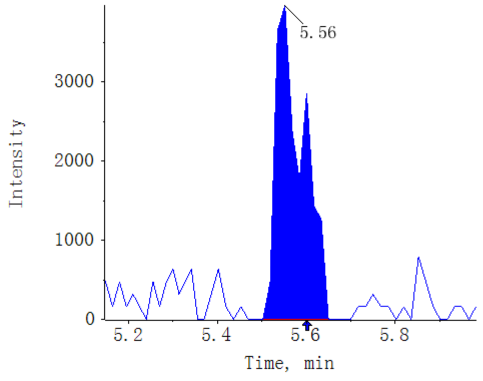

T24186682b_b
GA34 AREA:1.79e4 S/N:10.9

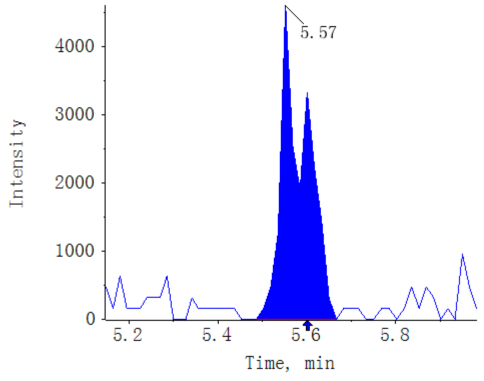

T24186682b_c
GA34 AREA:1.70e4 S/N:9.9

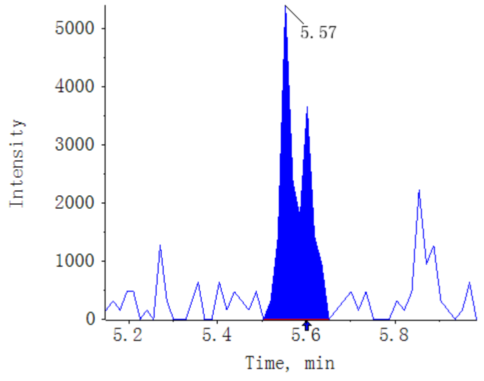

T24186683b_a
GA34 AREA:5.41e4 S/N:19.1

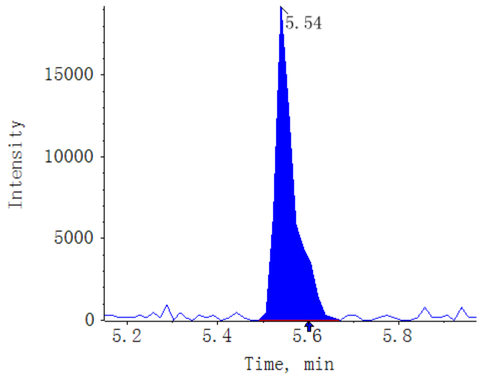

T24186683b_b
GA34 AREA:5.18e4 S/N:19.1

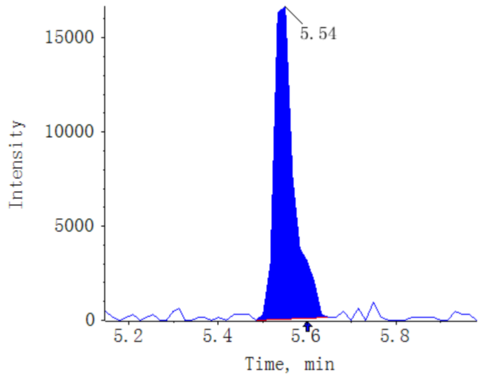

T24186683b_c
GA34 AREA:4.91e4 S/N:17.5

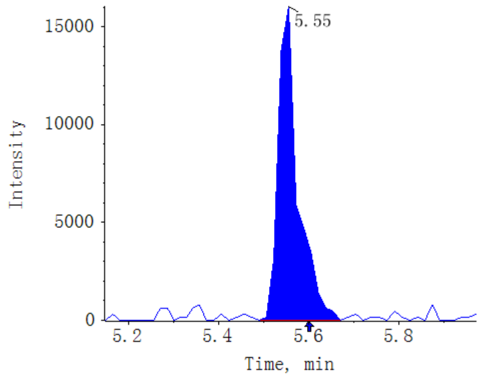

T24186684b_a
GA34 AREA:3.20e4 S/N:25.9

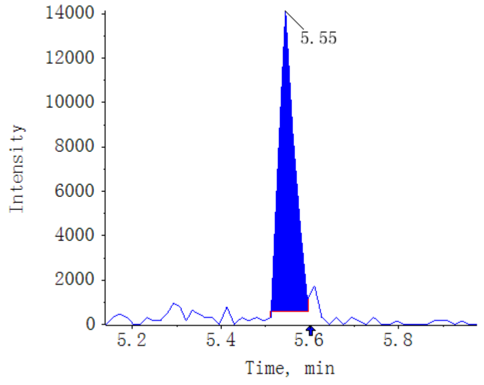

T24186684b_b
GA34 AREA:3.06e4 S/N:24.6

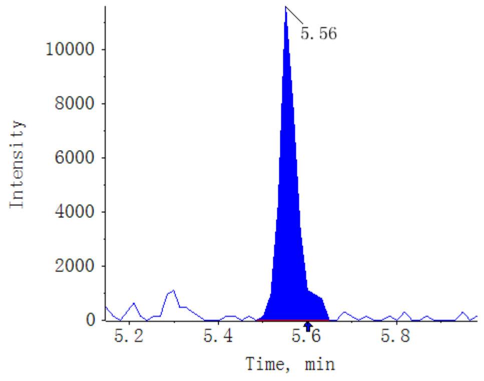

T24186684b_c
GA34 AREA:2.73e4 S/N:31.0

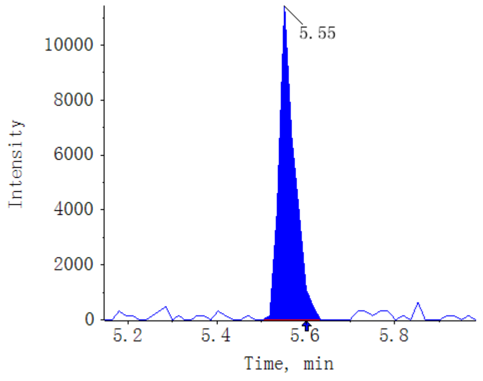

Result Table	MWXS-24-3064-a_9_WH6500-17_A20-3_V6.0_WSS_20240730	Algorithm Used	MQ4
Acquisition Method	ACC-PHs_V6.0_WH6500-17_CMY_20240521.dam	Instrument Name	Triple Quad 6500+ Low Mass
Project	N/A	Analytes QTY	109:99

Compound name: GA51 (331.1 / 243.2)

Sample Name	Sample Type	Area (cps)	Is Area (cps)	RT (min)	S/N	Target Conc	Calculated Conc.()
STD_0.01ppb	Standard	N/A	3.538e5	N/A	N/A	0.0100	N/A
STD_0.05ppb	Standard	N/A	3.397e5	N/A	N/A	0.0500	N/A
STD_0.1ppb	Standard	N/A	3.587e5	N/A	N/A	0.1000	N/A
STD_0.5ppb	Standard	N/A	3.275e5	N/A	N/A	0.5000	N/A
STD_1ppb	Standard	7.92e3	3.746e5	5.71	9.0	1.0000	9.715611e-1
STD_5ppb	Standard	3.39e4	3.209e5	5.72	7.8	5.0000	5.514031e0
STD_10ppb	Standard	6.35e4	3.165e5	5.71	10.9	10.0000	1.062052e1
STD_50ppb	Standard	3.64e5	3.582e5	5.70	11.8	50.0000	5.447592e1
STD_100ppb	Standard	7.04e5	3.626e5	5.72	12.9	100.0000	1.043119e2
STD_200ppb	Standard	1.37e6	3.701e5	5.71	10.3	200.0000	1.992695e2
STD_500ppb	Standard	3.26e6	4.777e5	5.71	11.6	500.0000	3.672982e2
V2.0_MW_RQC1_20240724	Quality Control	N/A	1.299e5	N/A	N/A	0.0000	N/A
Blank	Unknown	N/A	5.220e2	N/A	N/A	N/A	N/A
V3.0_MWMS_20240725_1	Unknown	6.03e5	3.708e5	5.68	11.9	N/A	8.733001e1
MWXS243064a_R1	Quality Control	N/A	1.034e5	N/A	N/A	0.0000	N/A
MWXS243064a_R2	Quality Control	N/A	1.066e5	N/A	N/A	0.0000	N/A
MWXS243064a_R3	Quality Control	N/A	1.022e5	N/A	N/A	0.0000	N/A
T24186682b_a	Unknown	N/A	8.442e4	N/A	N/A	N/A	N/A
T24186682b_b	Unknown	N/A	7.738e4	N/A	N/A	N/A	N/A
T24186682b_c	Unknown	N/A	9.288e4	N/A	N/A	N/A	N/A
T24186683b_a	Unknown	N/A	1.648e5	N/A	N/A	N/A	N/A
T24186683b_b	Unknown	N/A	1.807e5	N/A	N/A	N/A	N/A
T24186683b_c	Unknown	N/A	1.771e5	N/A	N/A	N/A	N/A
T24186684b_a	Unknown	N/A	9.244e4	N/A	N/A	N/A	N/A
T24186684b_b	Unknown	N/A	1.062e5	N/A	N/A	N/A	N/A
T24186684b_c	Unknown	N/A	1.130e5	N/A	N/A	N/A	N/A

Compound name: GA51
Regression Equation: y = 0.01860 x + 0.00306 (r = 0.99110) (weighting: 1 / x^2)

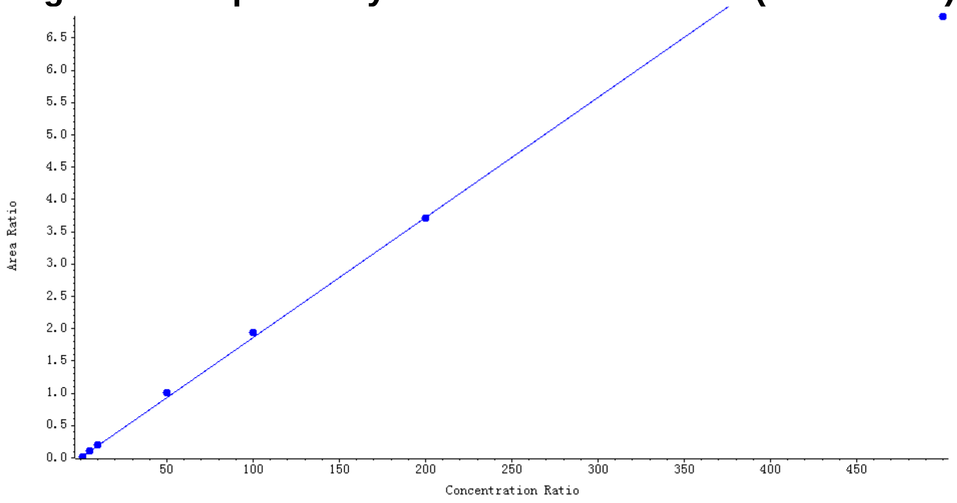

Peak Review

Blank
GA51 AREA:N/A S/N:N/A

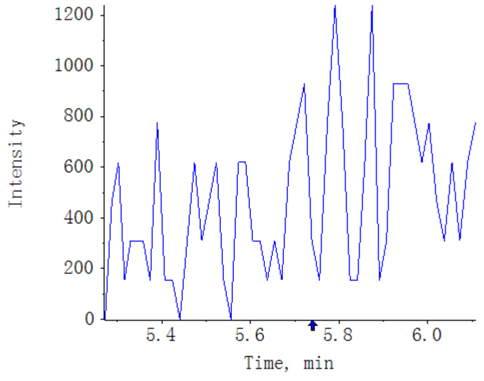

V3.0_MWMS_20240725_1
GA51 AREA:6.03e5 S/N:11.9

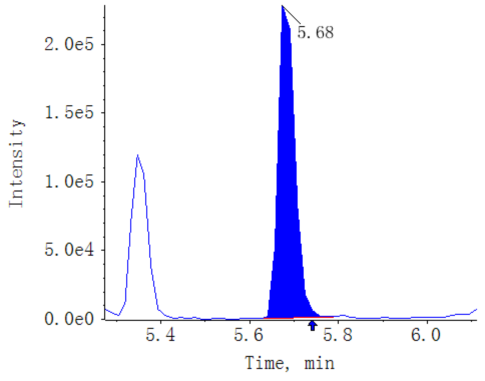

T24186682b_a
GA51 AREA:N/A S/N:N/A

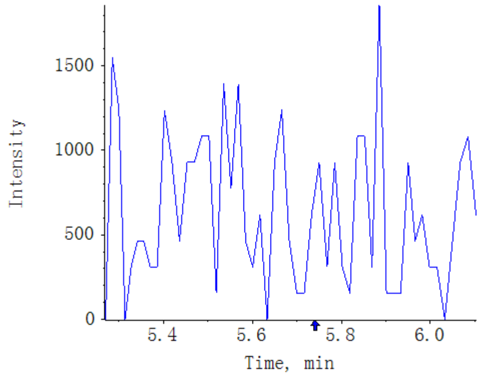

T24186682b_b
GA51 AREA:N/A S/N:N/A

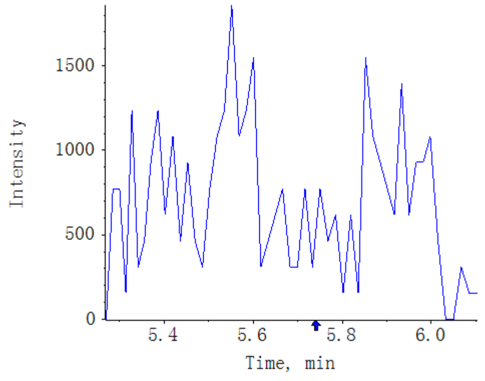

T24186682b_c
GA51 AREA:N/A S/N:N/A

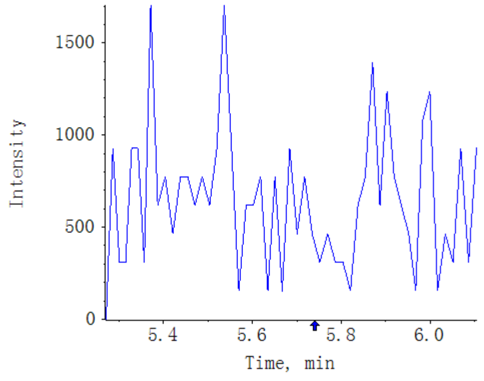

T24186683b_a
GA51 AREA:N/A S/N:N/A

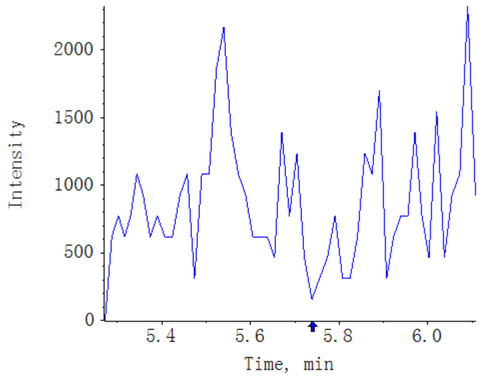

T24186683b_b
GA51 AREA:N/A S/N:N/A

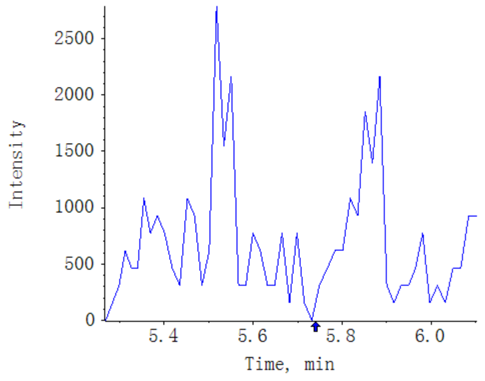

T24186683b_c
GA51 AREA:N/A S/N:N/A

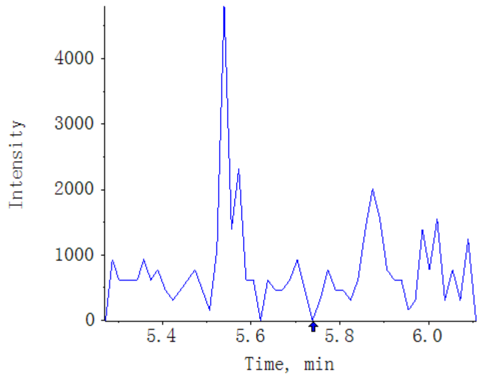

T24186684b_a
GA51 AREA:N/A S/N:N/A

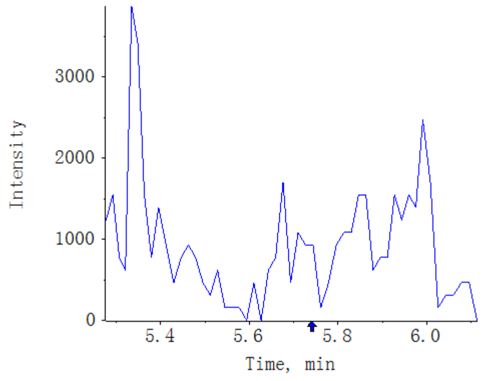

T24186684b_b
GA51 AREA:N/A S/N:N/A

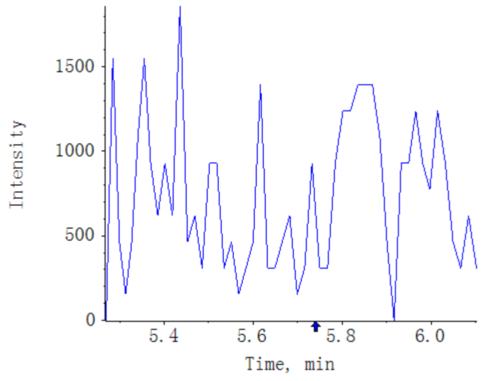

T24186684b_c
GA51 AREA:N/A S/N:N/A

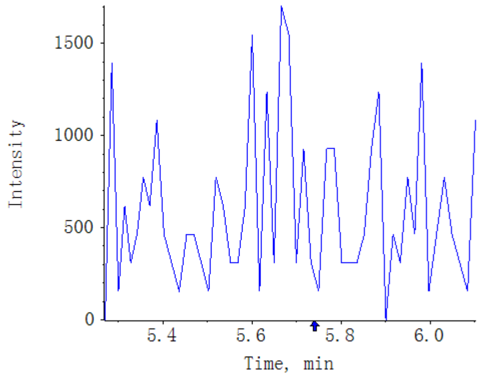

Result Table	MWXS-24-3064-a_9_WH6500-17_A20-3_V6.0_WSS_20240730	Algorithm Used	MQ4
Acquisition Method	ACC-PHs_V6.0_WH6500-17_CMY_20240521.dam	Instrument Name	Triple Quad 6500+ Low Mass
Project	N/A	Analytes QTY	109:100

Compound name: GA12-ald (315.1 / 271.1)

Sample Name	Sample Type	Area (cps)	Is Area (cps)	RT (min)	S/N	Target Conc	Calculated Conc.()
STD_0.01ppb	Standard	N/A	3.538e5	N/A	N/A	0.0100	N/A
STD_0.05ppb	Standard	3.93e2	3.397e5	7.70	7.2	0.0500	4.814542e-2
STD_0.1ppb	Standard	9.22e2	3.587e5	7.71	8.8	0.1000	1.112710e-1
STD_0.5ppb	Standard	3.44e3	3.275e5	7.70	40.1	0.5000	4.648504e-1
STD_1ppb	Standard	6.19e3	3.746e5	7.70	49.2	1.0000	7.336483e-1
STD_5ppb	Standard	4.07e4	3.209e5	7.72	155.1	5.0000	5.657186e0
STD_10ppb	Standard	6.63e4	3.165e5	7.70	136.3	10.0000	9.342544e0
STD_50ppb	Standard	4.23e5	3.582e5	7.70	176.7	50.0000	5.262031e1
STD_100ppb	Standard	8.46e5	3.626e5	7.71	162.5	100.0000	1.040849e2
STD_200ppb	Standard	1.83e6	3.701e5	7.71	251.8	200.0000	2.204171e2
STD_500ppb	Standard	N/A	4.777e5	N/A	N/A	500.0000	N/A
V2.0_MW_RQC1_20240724	Quality Control	N/A	1.299e5	N/A	N/A	0.0000	N/A
Blank	Unknown	N/A	5.220e2	N/A	N/A	N/A	N/A
V3.0_MWMS_20240725_1	Unknown	4.19e4	3.708e5	7.69	178.7	N/A	5.039555e0
MWXS243064a_R1	Quality Control	N/A	1.034e5	N/A	N/A	0.0000	N/A
MWXS243064a_R2	Quality Control	N/A	1.066e5	N/A	N/A	0.0000	N/A
MWXS243064a_R3	Quality Control	N/A	1.022e5	N/A	N/A	0.0000	N/A
T24186682b_a	Unknown	N/A	8.442e4	N/A	N/A	N/A	N/A
T24186682b_b	Unknown	N/A	7.738e4	N/A	N/A	N/A	N/A
T24186682b_c	Unknown	N/A	9.288e4	N/A	N/A	N/A	N/A
T24186683b_a	Unknown	N/A	1.648e5	N/A	N/A	N/A	N/A
T24186683b_b	Unknown	N/A	1.807e5	N/A	N/A	N/A	N/A
T24186683b_c	Unknown	N/A	1.771e5	N/A	N/A	N/A	N/A
T24186684b_a	Unknown	N/A	9.244e4	N/A	N/A	N/A	N/A
T24186684b_b	Unknown	N/A	1.062e5	N/A	N/A	N/A	N/A
T24186684b_c	Unknown	N/A	1.130e5	N/A	N/A	N/A	N/A

Compound name: GA12-ald
Regression Equation: y = 0.02242 x + 7.67665e-5 (r = 0.99083) (weighting: 1 / x^2)

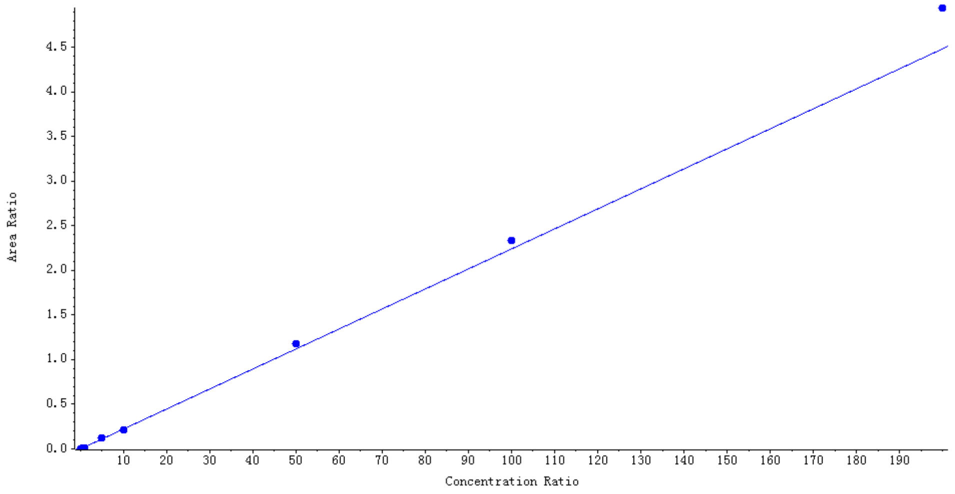

Peak Review

Blank

GA12-ald AREA:N/A S/N:N/A

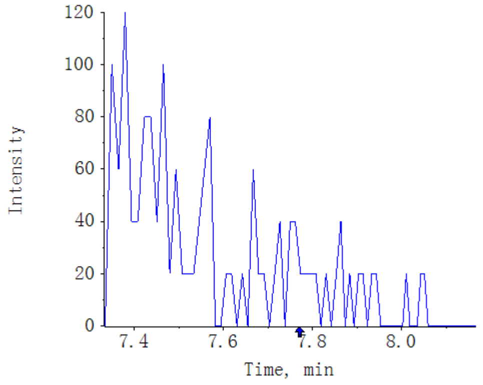

V3.0_MWMS_20240725_1

GA12-ald AREA:4.19e4 S/N:178.7

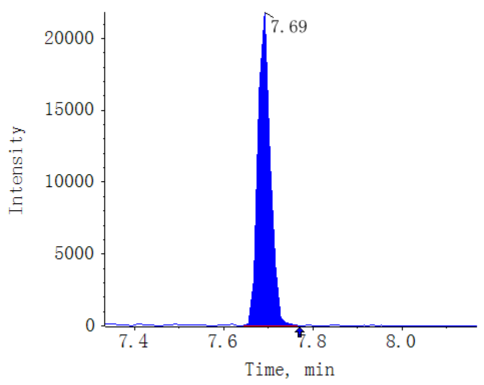

T24186682b_a

GA12-ald AREA:N/A S/N:N/A

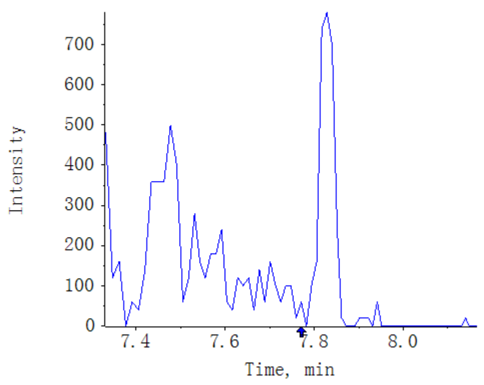

T24186682b_b

GA12-ald AREA:N/A S/N:N/A

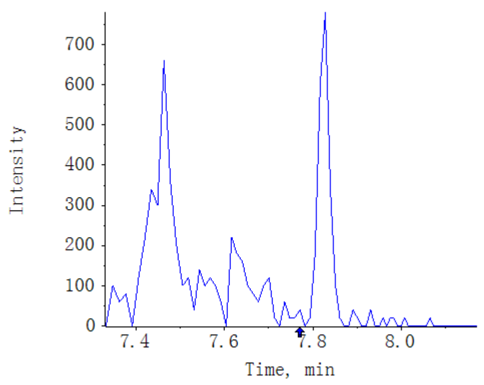

T24186682b_c

GA12-ald AREA:N/A S/N:N/A

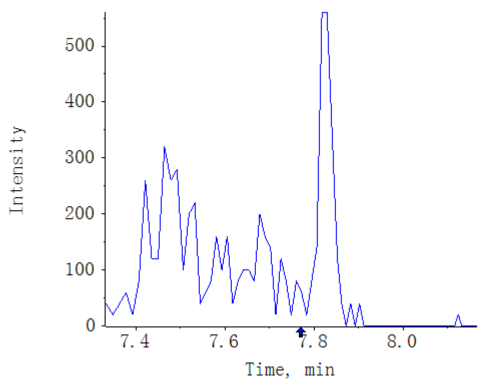

T24186683b_a

GA12-ald AREA:N/A S/N:N/A

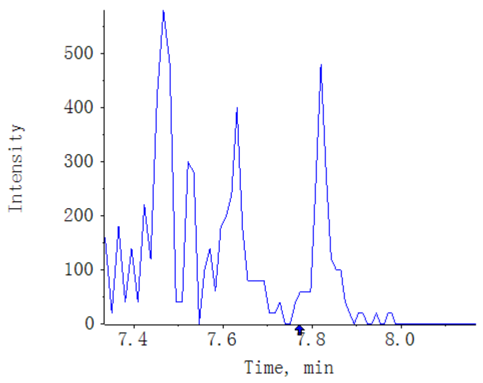

T24186683b_b

GA12-ald AREA:N/A S/N:N/A

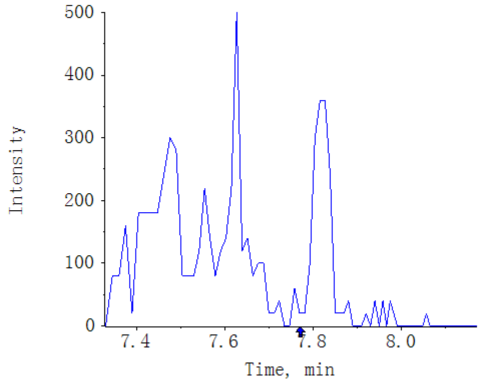

T24186683b_c

GA12-ald AREA:N/A S/N:N/A

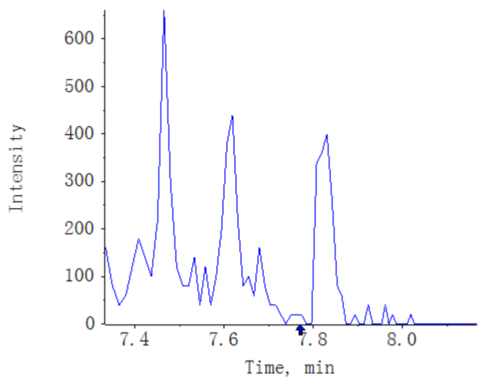

T24186684b_a

GA12-ald AREA:N/A S/N:N/A

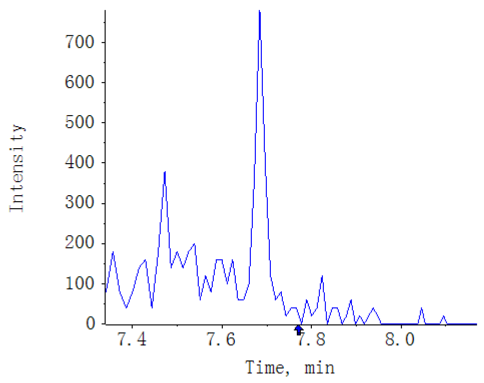

T24186684b_b

GA12-ald AREA:N/A S/N:N/A

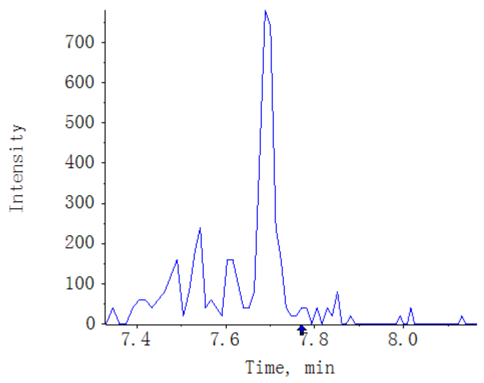

T24186684b_c

GA12-ald AREA:N/A S/N:N/A

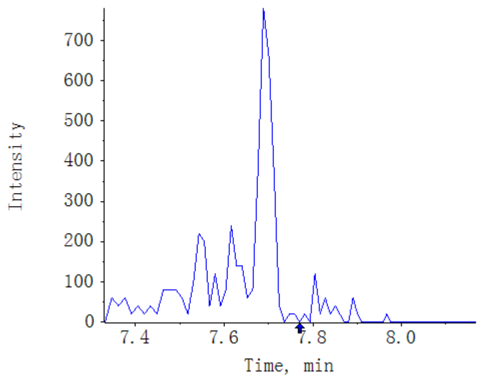

**Result Table**	MWXS-24-3064-a_9_WH6500-17_A20-3_V6.0_WSS_20240730	**Algorithm Used**	MQ4
**Acquisition Method**	ACC-PHs_V6.0_WH6500-17_CMY_20240521.dam	**Instrument Name**	Triple Quad 6500+ Low Mass
**Project**	N/A	**Analytes QTY**	109:101

Compound name: JA-Val (308.0 / 116.0)

Sample Name	Sample Type	Area (cps)	Is Area (cps)	RT (min)	S/N	Target Conc	Calculated Conc.()
STD_0.01ppb	Standard	6.44e3	7.502e5	6.01	5.3	0.0100	1.010583e-2
STD_0.05ppb	Standard	2.65e4	7.505e5	6.01	20.9	0.0500	4.857154e-2
STD_0.1ppb	Standard	4.96e4	7.760e5	6.03	35.6	0.1000	8.986950e-2
STD_0.5ppb	Standard	2.80e5	6.580e5	6.01	58.7	0.5000	6.118082e-1
STD_1ppb	Standard	4.69e5	6.418e5	6.02	51.9	1.0000	1.051344e0
STD_5ppb	Standard	2.99e6	7.441e5	6.03	55.9	5.0000	5.781938e0
STD_10ppb	Standard	5.22e6	7.645e5	6.02	68.3	10.0000	9.844487e0
STD_50ppb	Standard	2.81e7	8.212e5	6.01	46.4	50.0000	4.930811e1
STD_100ppb	Standard	5.07e7	8.032e5	6.03	71.9	100.0000	9.096719e1
STD_200ppb	Standard	1.05e8	9.369e5	6.02	43.0	200.0000	1.615321e2
STD_500ppb	Standard	N/A	8.441e5	N/A	N/A	500.0000	N/A
V2.0_MW_RQC1_20240724	Quality Control	2.02e5	3.905e5	6.03	32.1	0.0000	7.429434e-1
Blank	Unknown	N/A	5.961e3	N/A	N/A	N/A	N/A
V3.0_MWMS_20240725_1	Unknown	2.88e6	1.232e6	6.00	68.4	N/A	3.366980e0
MWXS243064a_R1	Quality Control	N/A	3.925e5	N/A	N/A	0.0000	N/A
MWXS243064a_R2	Quality Control	N/A	3.390e5	N/A	N/A	0.0000	N/A
MWXS243064a_R3	Quality Control	N/A	3.379e5	N/A	N/A	0.0000	N/A
T24186682b_a	Unknown	1.99e3	3.024e5	6.02	9.4	N/A	7.212696e-3
T24186682b_b	Unknown	3.56e3	2.893e5	6.01	8.1	N/A	1.548720e-2
T24186682b_c	Unknown	2.01e3	2.806e5	6.02	6.3	N/A	8.046473e-3
T24186683b_a	Unknown	N/A	2.778e5	N/A	N/A	N/A	N/A
T24186683b_b	Unknown	N/A	3.603e5	N/A	N/A	N/A	N/A
T24186683b_c	Unknown	N/A	3.184e5	N/A	N/A	N/A	N/A
T24186684b_a	Unknown	7.14e3	3.496e5	5.99	13.3	N/A	2.718008e-2
T24186684b_b	Unknown	4.09e3	3.022e5	6.02	8.6	N/A	1.724998e-2
T24186684b_c	Unknown	3.58e3	2.908e5	6.01	10.7	N/A	1.545786e-2

Compound name: JA-Val
Regression Equation: y = 0.69361 x + 0.00158 (r = 0.99204) (weighting: 1 / x^2)

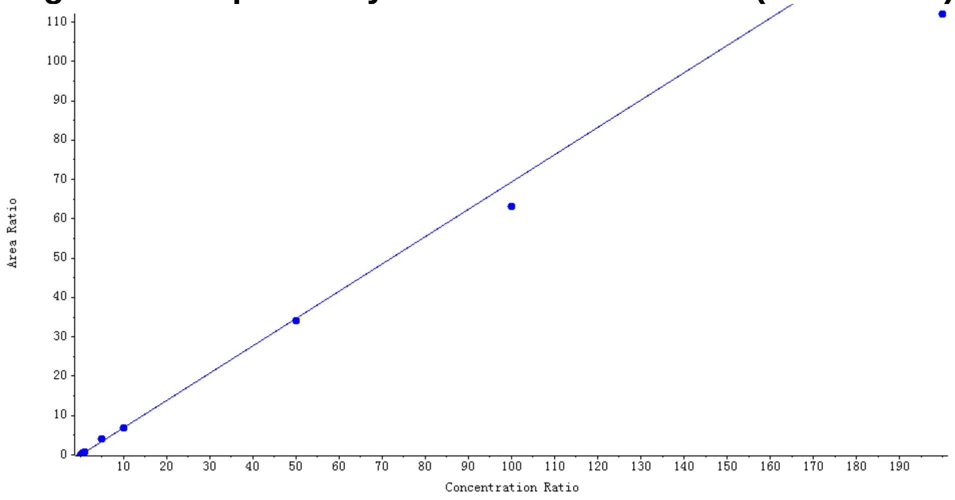

Peak Review

Blank
JA-Val AREA:N/A S/N:N/A

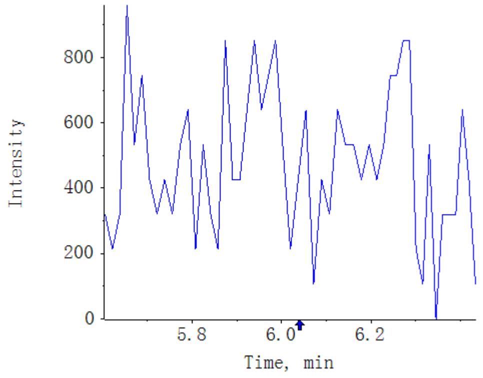

V3.0_MWMS_20240725_1
JA-Val AREA:2.88e6 S/N:68.4

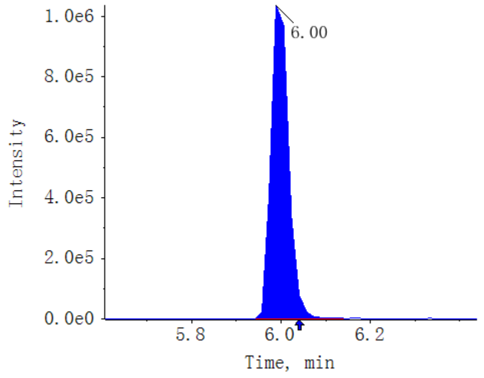

T24186682b_a
JA-Val AREA:1.99e3 S/N:9.4

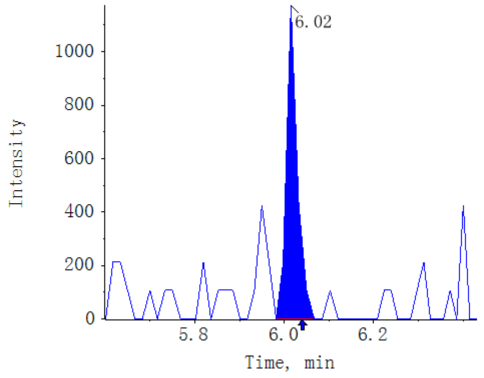

T24186682b_b
JA-Val AREA:3.56e3 S/N:8.1

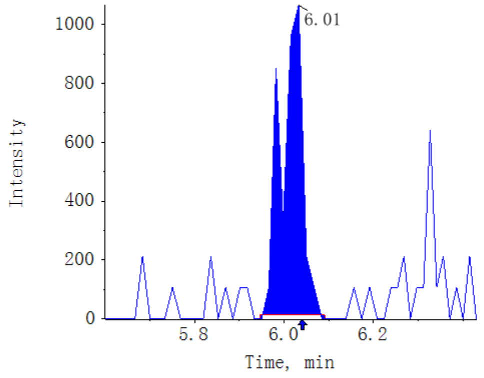

T24186682b_c
JA-Val AREA:2.01e3 S/N:6.3

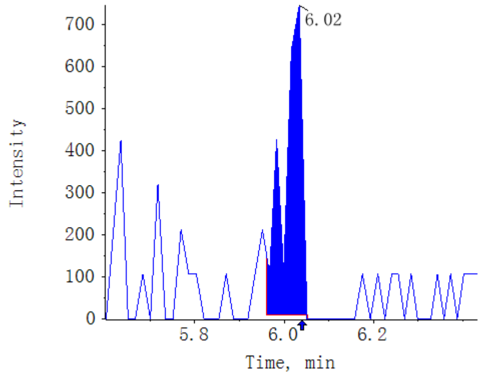

T24186683b_a
JA-Val AREA:N/A S/N:N/A

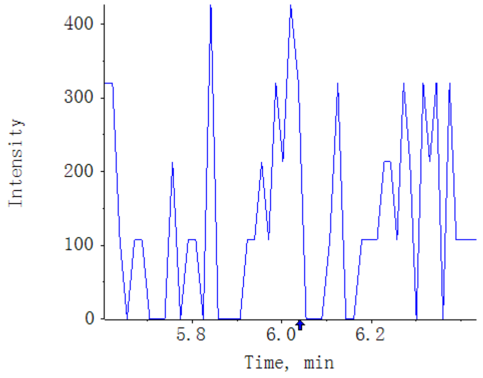

T24186683b_b
JA-Val AREA:N/A S/N:N/A

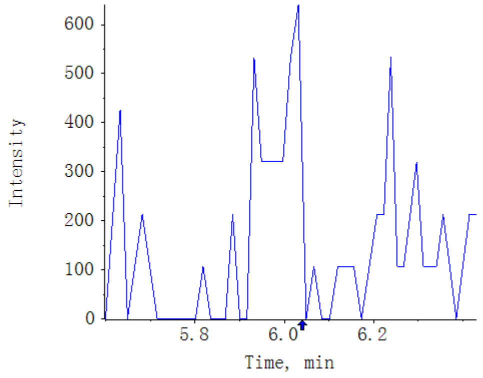

T24186683b_c
JA-Val AREA:N/A S/N:N/A

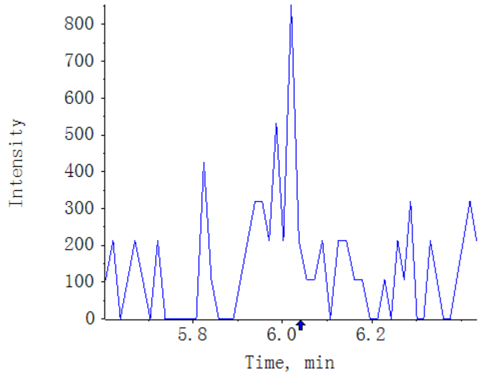

T24186684b_a
JA-Val AREA:7.14e3 S/N:13.3

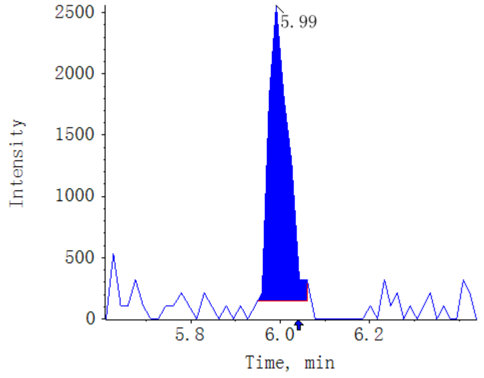

T24186684b_b
JA-Val AREA:4.09e3 S/N:8.6

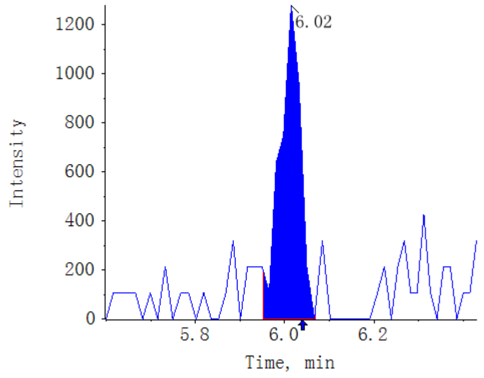

T24186684b_c
JA-Val AREA:3.58e3 S/N:10.7

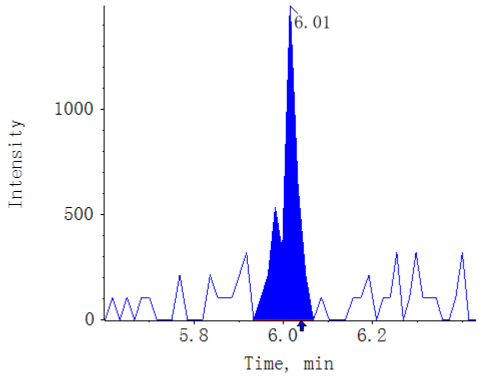

Result Table	MWXS-24-3064-a_9_WH6500-17_A20-3_V6.0_WSS_20240730	Algorithm Used	MQ4
Acquisition Method	ACC-PHs_V6.0_WH6500-17_CMY_20240521.dam	Instrument Name	Triple Quad 6500+ Low Mass
Project	N/A	Analytes QTY	109:102

Compound name: H2JA (211.1 / 59.0)

Sample Name	Sample Type	Area (cps)	Is Area (cps)	RT (min)	S/N	Target Conc	Calculated Conc.()
STD_0.01ppb	Standard	6.29e3	7.502e5	6.17	22.1	0.2000	1.986419e-1
STD_0.05ppb	Standard	3.65e4	7.505e5	6.17	47.0	1.0000	1.020763e0
STD_0.1ppb	Standard	7.51e4	7.760e5	6.18	66.0	2.0000	2.000786e0
STD_0.5ppb	Standard	3.64e5	6.580e5	6.17	53.2	10.0000	1.130699e1
STD_1ppb	Standard	6.17e5	6.418e5	6.17	78.3	20.0000	1.962190e1
STD_5ppb	Standard	4.07e6	7.441e5	6.19	52.8	100.0000	1.115643e2
STD_10ppb	Standard	7.26e6	7.645e5	6.17	62.0	200.0000	1.937996e2
STD_50ppb	Standard	3.81e7	8.212e5	6.17	68.6	1000.0000	9.461896e2
STD_100ppb	Standard	6.64e7	8.032e5	6.18	50.6	2000.0000	1.686021e3
STD_200ppb	Standard	N/A	9.369e5	N/A	N/A	4000.0000	N/A
STD_500ppb	Standard	N/A	8.441e5	N/A	N/A	10000.0000	N/A
V2.0_MW_RQC1_20240724	Quality Control	4.00e3	3.905e5	6.15	5.2	0.0000	2.366826e-1
Blank	Unknown	N/A	5.961e3	N/A	N/A	N/A	N/A
V3.0_MWMS_20240725_1	Unknown	4.94e6	1.232e6	6.15	89.2	N/A	8.181598e1
MWXS243064a_R1	Quality Control	N/A	3.925e5	N/A	N/A	0.0000	N/A
MWXS243064a_R2	Quality Control	N/A	3.390e5	N/A	N/A	0.0000	N/A
MWXS243064a_R3	Quality Control	N/A	3.379e5	N/A	N/A	0.0000	N/A
T24186682b_a	Unknown	N/A	3.024e5	N/A	N/A	N/A	N/A
T24186682b_b	Unknown	N/A	2.893e5	N/A	N/A	N/A	N/A
T24186682b_c	Unknown	N/A	2.806e5	N/A	N/A	N/A	N/A
T24186683b_a	Unknown	N/A	2.778e5	N/A	N/A	N/A	N/A
T24186683b_b	Unknown	N/A	3.603e5	N/A	N/A	N/A	N/A
T24186683b_c	Unknown	N/A	3.184e5	N/A	N/A	N/A	N/A
T24186684b_a	Unknown	N/A	3.496e5	N/A	N/A	N/A	N/A
T24186684b_b	Unknown	N/A	3.022e5	N/A	N/A	N/A	N/A
T24186684b_c	Unknown	N/A	2.908e5	N/A	N/A	N/A	N/A

Compound name: H2JA
Regression Equation: y = 0.04904 x + -0.00136 (r = 0.99593) (weighting: 1 / x^2)

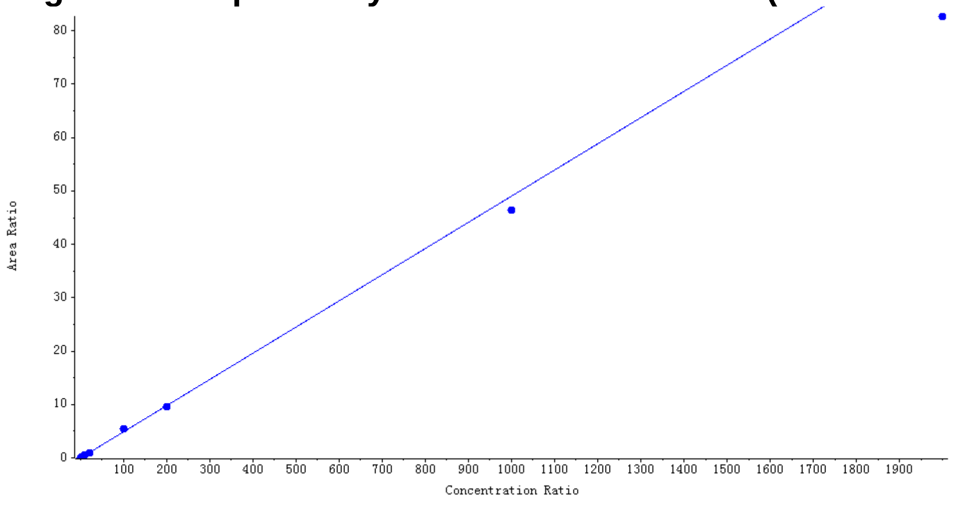

Peak Review

Blank
H2JA AREA:N/A S/N:N/A

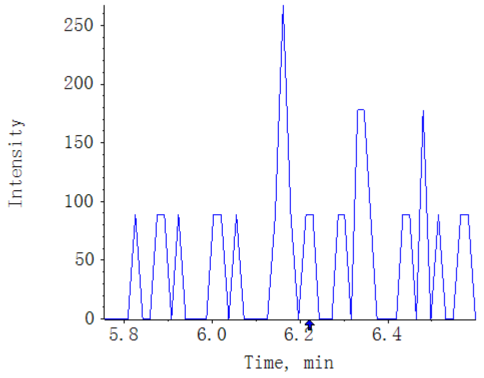

V3.0_MWMS_20240725_1
H2JA AREA:4.94e6 S/N:89.2

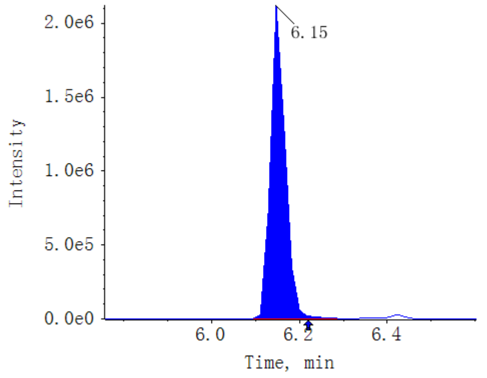

T24186682b_a
H2JA AREA:N/A S/N:N/A

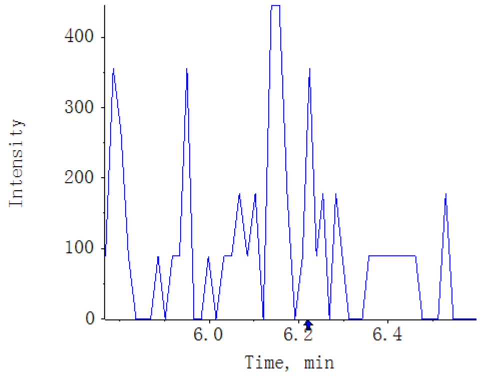

T24186682b_b
H2JA AREA:N/A S/N:N/A

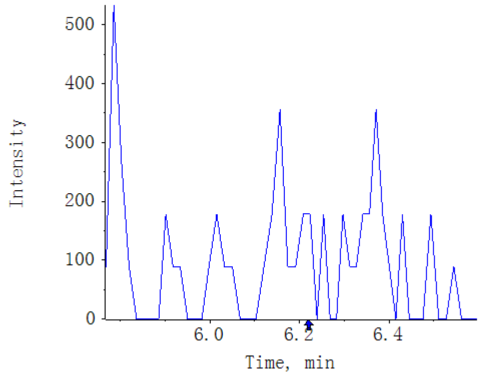

T24186682b_c
H2JA AREA:N/A S/N:N/A

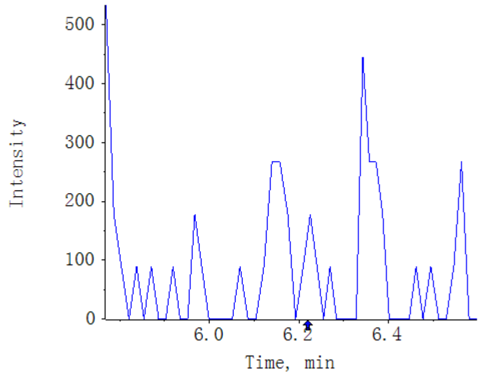

T24186683b_a
H2JA AREA:N/A S/N:N/A

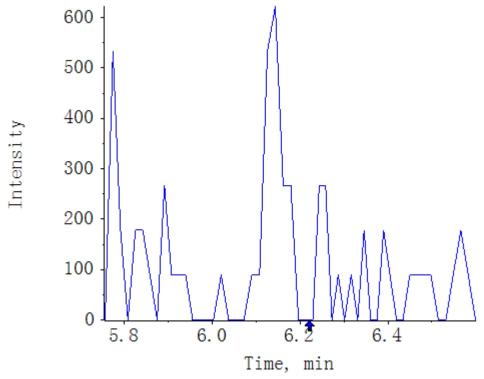

T24186683b_b
H2JA AREA:N/A S/N:N/A

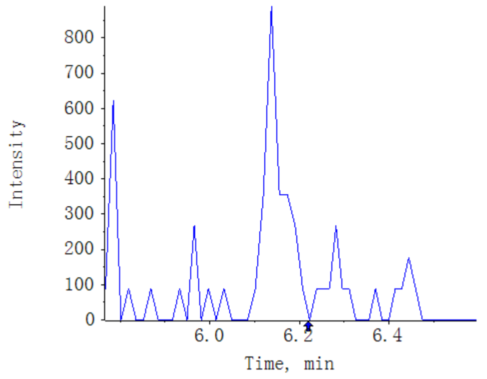

T24186683b_c
H2JA AREA:N/A S/N:N/A

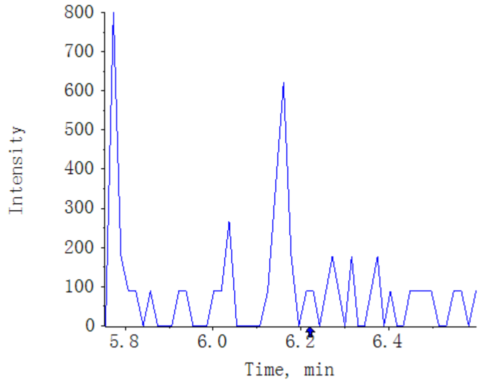

T24186684b_a
H2JA AREA:N/A S/N:N/A

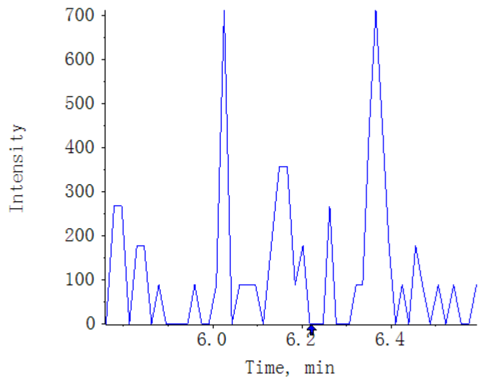

T24186684b_b
H2JA AREA:N/A S/N:N/A

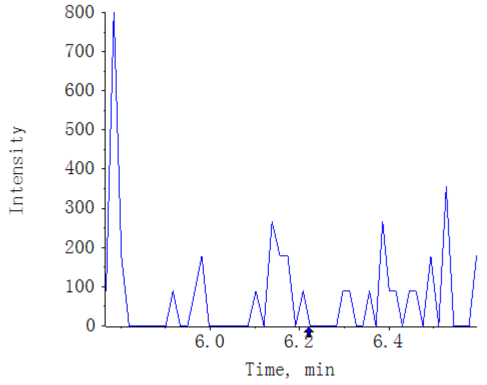

T24186684b_c
H2JA AREA:N/A S/N:N/A

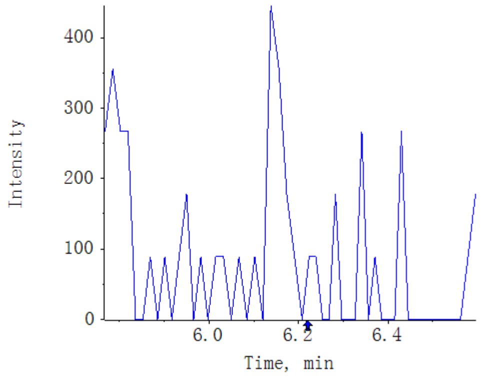

Result Table	MWXS-24-3064-a_9_WH6500-17_A20-3_V6.0_WSS_20240730	Algorithm Used	MQ4
Acquisition Method	ACC-PHs_V6.0_WH6500-17_CMY_20240521.dam	Instrument Name	Triple Quad 6500+ Low Mass
Project	N/A	Analytes QTY	109:103

Compound name: JA (209.1 / 58.8)

Sample Name	Sample Type	Area (cps)	Is Area (cps)	RT (min)	S/N	Target Conc	Calculated Conc.()
STD_0.01ppb	Standard	2.03e3	7.502e5	5.81	6.9	0.0100	7.656658e-3
STD_0.05ppb	Standard	3.51e3	7.505e5	5.81	10.0	0.0500	4.110584e-2
STD_0.1ppb	Standard	5.39e3	7.760e5	5.82	10.5	0.1000	7.962153e-2
STD_0.5ppb	Standard	2.68e4	6.580e5	5.81	38.7	0.5000	6.523618e-1
STD_1ppb	Standard	4.17e4	6.418e5	5.81	36.0	1.0000	1.064239e0
STD_5ppb	Standard	2.59e5	7.441e5	5.82	36.4	5.0000	5.863822e0
STD_10ppb	Standard	4.83e5	7.645e5	5.81	48.6	10.0000	1.068283e1
STD_50ppb	Standard	2.54e6	8.212e5	5.81	43.0	50.0000	5.233084e1
STD_100ppb	Standard	4.55e6	8.032e5	5.82	37.6	100.0000	9.593752e1
STD_200ppb	Standard	N/A	9.369e5	N/A	N/A	200.0000	N/A
STD_500ppb	Standard	N/A	8.441e5	N/A	N/A	500.0000	N/A
V2.0_MW_RQC1_20240724	Quality Control	1.40e6	3.905e5	5.79	70.8	0.0000	6.078485e1
Blank	Unknown	N/A	5.961e3	N/A	N/A	N/A	N/A
V3.0_MWMS_20240725_1	Unknown	3.02e5	1.232e6	5.79	28.6	N/A	4.112259e0
MWXS243064a_R1	Quality Control	4.51e4	3.925e5	5.77	42.1	0.0000	1.910231e0
MWXS243064a_R2	Quality Control	4.36e4	3.390e5	5.78	50.8	0.0000	2.142532e0
MWXS243064a_R3	Quality Control	4.19e4	3.379e5	5.77	42.9	0.0000	2.062379e0
T24186682b_a	Unknown	3.41e4	3.024e5	5.78	58.0	N/A	1.876408e0
T24186682b_b	Unknown	4.37e4	2.893e5	5.78	52.7	N/A	2.520651e0
T24186682b_c	Unknown	3.03e4	2.806e5	5.78	46.4	N/A	1.793595e0
T24186683b_a	Unknown	2.11e4	2.778e5	5.77	35.0	N/A	1.248903e0
T24186683b_b	Unknown	2.40e4	3.603e5	5.77	31.9	N/A	1.092736e0
T24186683b_c	Unknown	3.01e4	3.184e5	5.77	47.7	N/A	1.562879e0
T24186684b_a	Unknown	9.27e4	3.496e5	5.78	46.3	N/A	4.458139e0
T24186684b_b	Unknown	1.05e5	3.022e5	5.78	54.6	N/A	5.827671e0
T24186684b_c	Unknown	8.38e4	2.908e5	5.79	45.7	N/A	4.846977e0

Compound name: JA
Regression Equation: y = 0.05898 x + 0.00226 (r = 0.99842) (weighting: 1 / x)

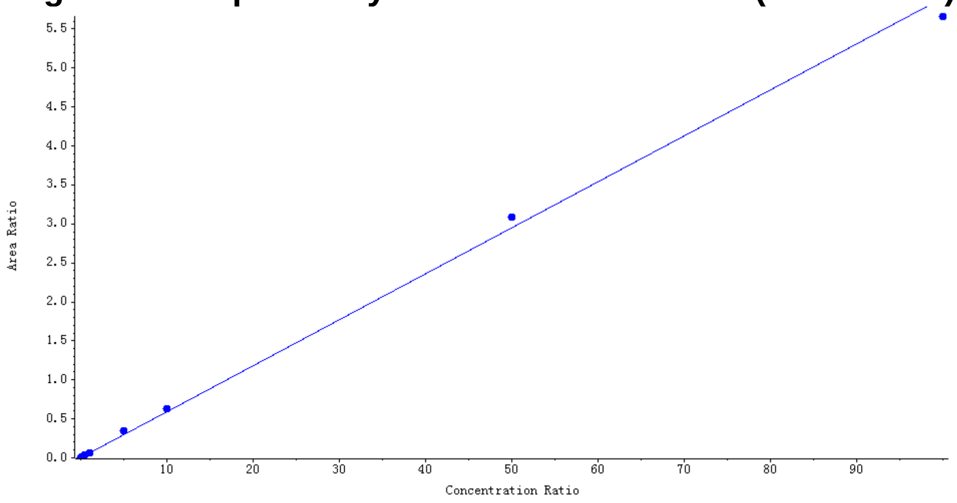

Peak Review

Blank
JA AREA:N/A S/N:N/A

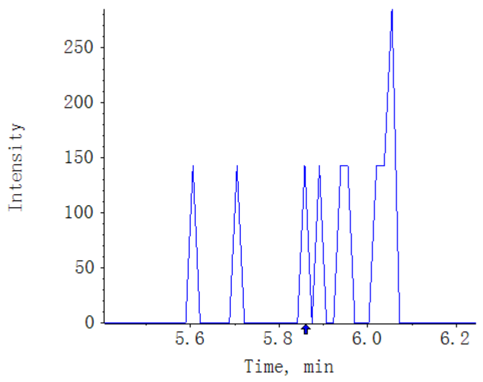

V3.0_MWMS_20240725_1
JA AREA:3.02e5 S/N:28.6

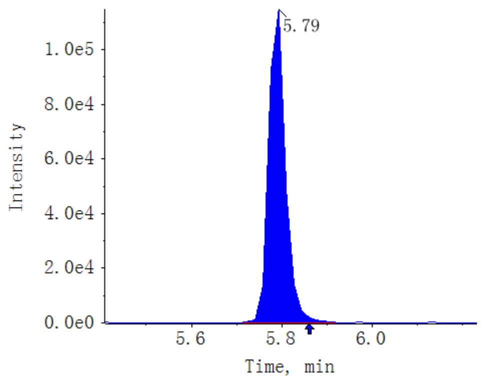

T24186682b_a
JA AREA:3.41e4 S/N:58.0

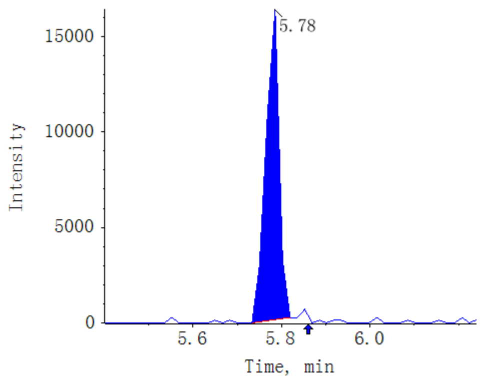

T24186682b_b
JA AREA:4.37e4 S/N:52.7

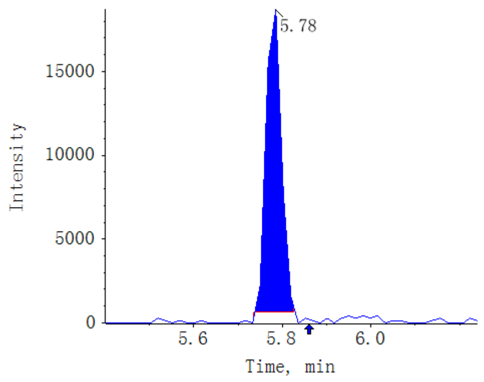

T24186682b_c
JA AREA:3.03e4 S/N:46.4

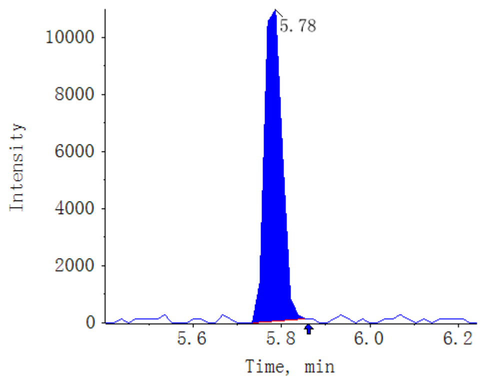

T24186683b_a
JA AREA:2.11e4 S/N:35.0

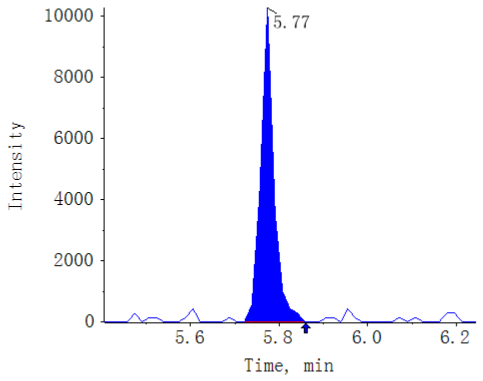

T24186683b_b
JA AREA:2.40e4 S/N:31.9

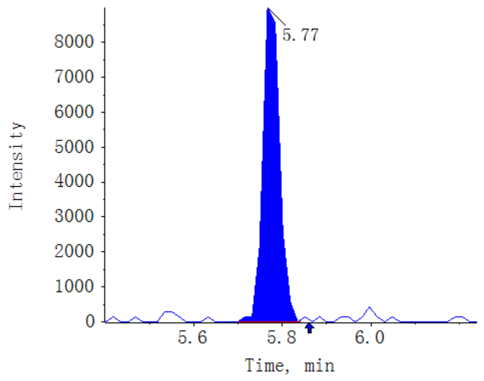

T24186683b_c
JA AREA:3.01e4 S/N:47.7

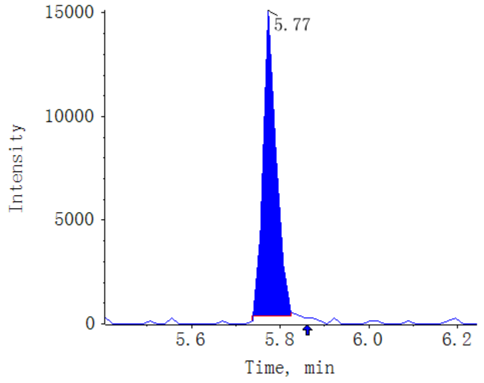

T24186684b_a
JA AREA:9.27e4 S/N:46.3

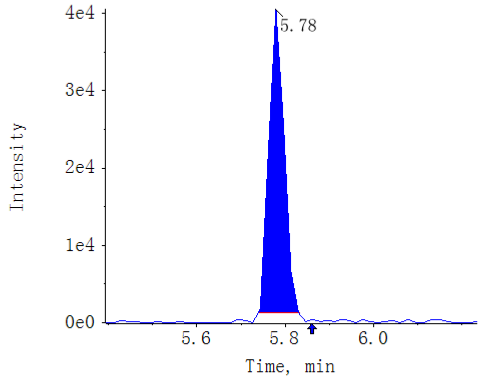

T24186684b_b
JA AREA:1.05e5 S/N:54.6

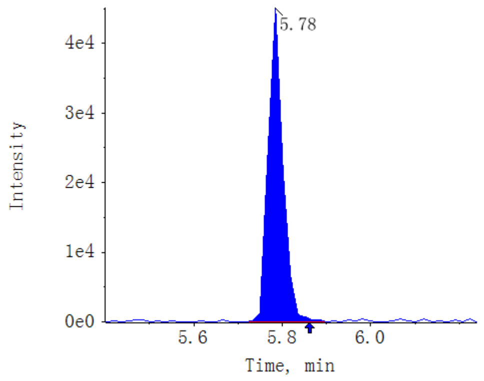

T24186684b_c
JA AREA:8.38e4 S/N:45.7

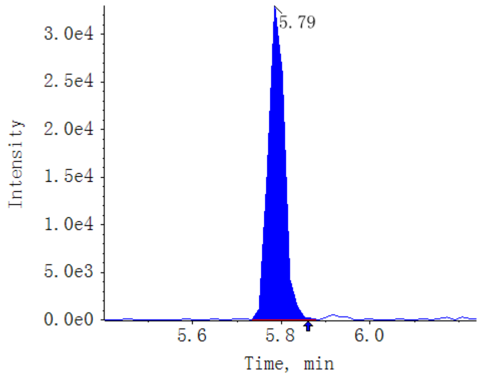

Result Table	MWXS-24-3064-a_9_WH6500-17_A20-3_V6.0_WSS_20240730	Algorithm Used	MQ4
Acquisition Method	ACC-PHs_V6.0_WH6500-17_CMY_20240521.dam	Instrument Name	Triple Quad 6500+ Low Mass
Project	N/A	Analytes QTY	109:104

Compound name: JA-ILE (322.4 / 129.7)

Sample Name	Sample Type	Area (cps)	Is Area (cps)	RT (min)	S/N	Target Conc	Calculated Conc.()
STD_0.01ppb	Standard	1.37e3	7.502e5	6.34	11.6	0.0100	9.691341e-3
STD_0.05ppb	Standard	7.78e3	7.505e5	6.35	25.8	0.0500	5.671625e-2
STD_0.1ppb	Standard	1.43e4	7.760e5	6.35	20.9	0.1000	1.010225e-1
STD_0.5ppb	Standard	6.84e4	6.580e5	6.34	34.7	0.5000	5.720598e-1
STD_1ppb	Standard	1.17e5	6.418e5	6.35	50.8	1.0000	1.003025e0
STD_5ppb	Standard	7.35e5	7.441e5	6.36	35.6	5.0000	5.436742e0
STD_10ppb	Standard	1.29e6	7.645e5	6.34	59.1	10.0000	9.281242e0
STD_50ppb	Standard	6.75e6	8.212e5	6.34	49.7	50.0000	4.527987e1
STD_100ppb	Standard	1.19e7	8.032e5	6.35	41.7	100.0000	8.181014e1
STD_200ppb	Standard	N/A	9.369e5	N/A	N/A	200.0000	N/A
STD_500ppb	Standard	N/A	8.441e5	N/A	N/A	500.0000	N/A
V2.0_MW_RQC1_20240724	Quality Control	1.63e6	3.905e5	6.35	26.9	0.0000	2.301620e1
Blank	Unknown	N/A	5.961e3	N/A	N/A	N/A	N/A
V3.0_MWMS_20240725_1	Unknown	6.87e5	1.232e6	6.33	67.6	N/A	3.071132e0
MWXS243064a_R1	Quality Control	1.38e4	3.925e5	6.33	24.9	0.0000	1.938907e-1
MWXS243064a_R2	Quality Control	1.54e4	3.390e5	6.34	27.7	0.0000	2.503988e-1
MWXS243064a_R3	Quality Control	1.54e4	3.379e5	6.33	17.6	0.0000	2.511029e-1
T24186682b_a	Unknown	2.79e4	3.024e5	6.34	21.9	N/A	5.075633e-1
T24186682b_b	Unknown	5.90e4	2.893e5	6.34	22.1	N/A	1.123493e0
T24186682b_c	Unknown	2.41e4	2.806e5	6.33	18.5	N/A	4.735918e-1
T24186683b_a	Unknown	5.91e3	2.778e5	6.33	16.6	N/A	1.167927e-1
T24186683b_b	Unknown	5.28e3	3.603e5	6.34	20.0	N/A	8.033165e-2
T24186683b_c	Unknown	9.30e3	3.184e5	6.34	11.0	N/A	1.605305e-1
T24186684b_a	Unknown	5.96e4	3.496e5	6.33	23.1	N/A	9.385015e-1
T24186684b_b	Unknown	4.85e4	3.022e5	6.34	26.6	N/A	8.842932e-1
T24186684b_c	Unknown	1.53e4	2.908e5	6.35	17.2	N/A	2.888238e-1

Compound name: JA-ILE
Regression Equation: y = 0.18156 x + 7.06750e-5 (r = 0.99358) (weighting: 1 / x^2)

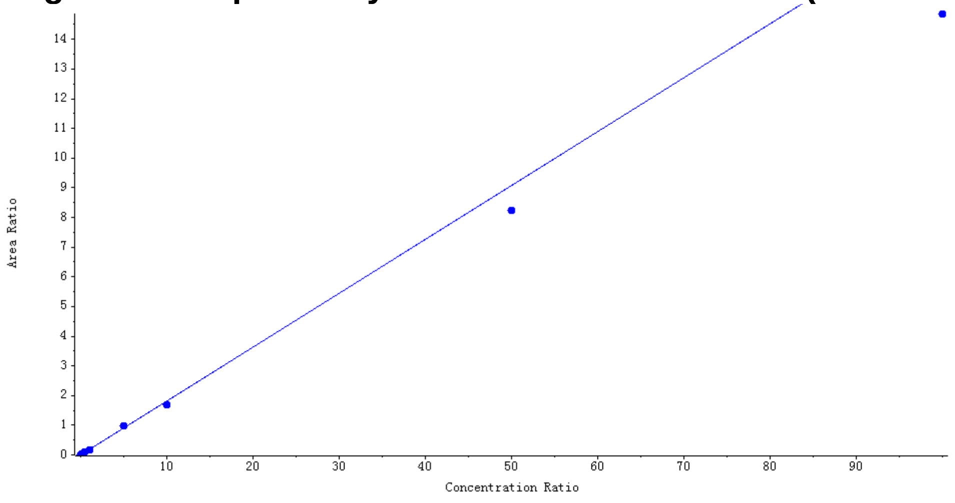

Peak Review

Blank
JA-ILE AREA:N/A S/N:N/A

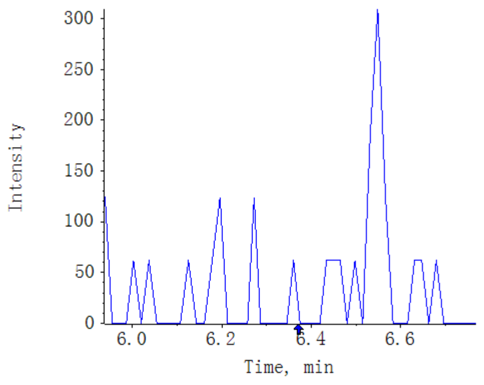

V3.0_MWMS_20240725_1
JA-ILE AREA:6.87e5 S/N:67.6

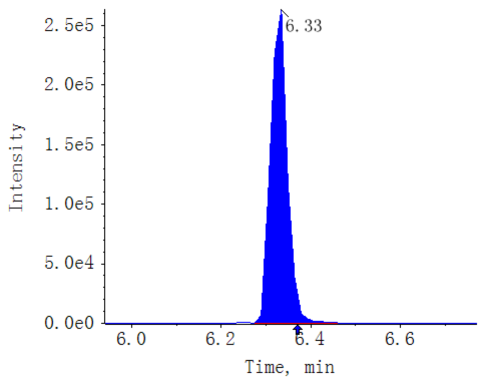

T24186682b_a
JA-ILE AREA:2.79e4 S/N:21.9

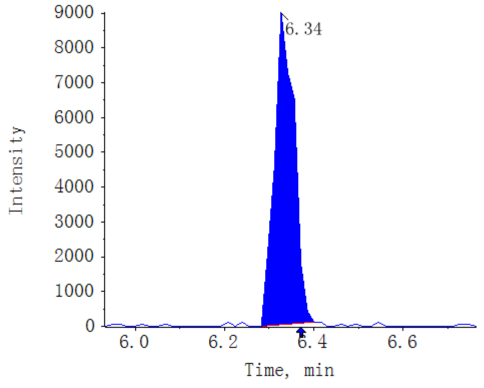

T24186682b_b
JA-ILE AREA:5.90e4 S/N:22.1

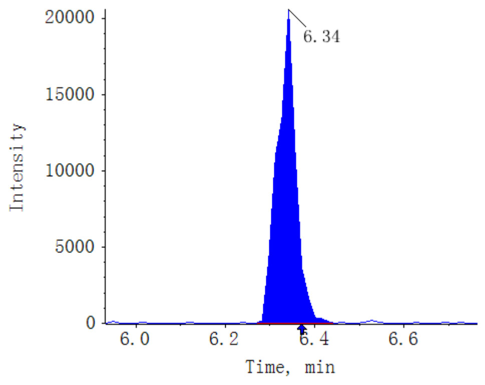

T24186682b_c
JA-ILE AREA:2.41e4 S/N:18.5

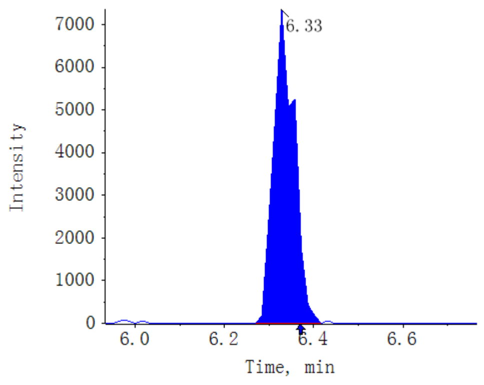

T24186683b_a
JA-ILE AREA:5.91e3 S/N:16.6

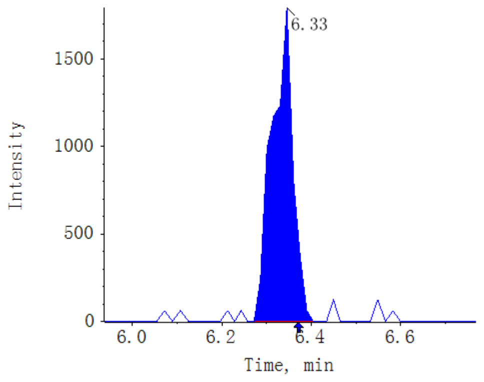

T24186683b_b
JA-ILE AREA:5.28e3 S/N:20.0

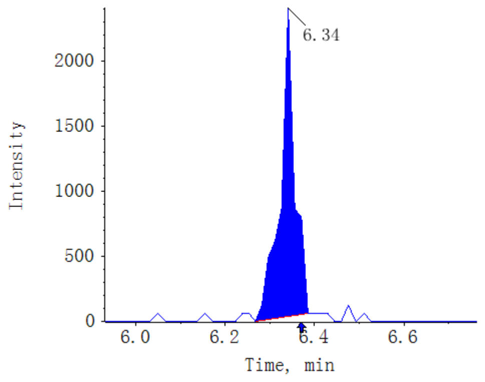

T24186683b_c
JA-ILE AREA:9.30e3 S/N:11.0

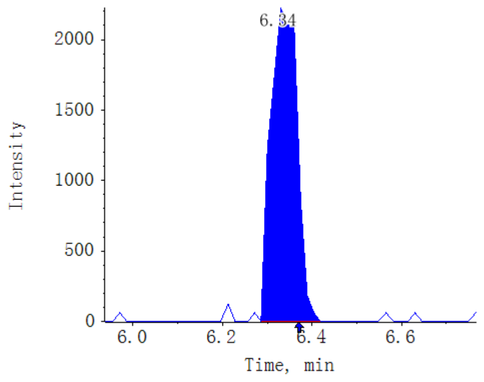

T24186684b_a
JA-ILE AREA:5.96e4 S/N:23.1

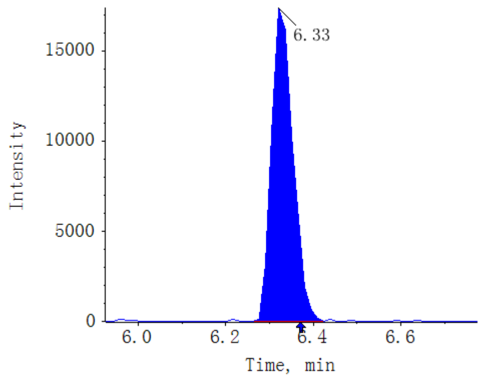

T24186684b_b
JA-ILE AREA:4.85e4 S/N:26.6

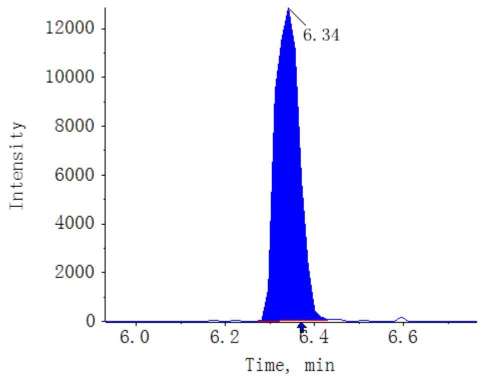

T24186684b_c
JA-ILE AREA:1.53e4 S/N:17.2

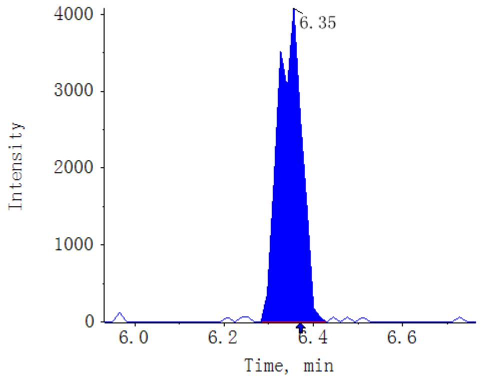

Result Table	MWXS-24-3064-a_9_WH6500-17_A20-3_V6.0_WSS_20240730	Algorithm Used	MQ4
Acquisition Method	ACC-PHs_V6.0_WH6500-17_CMY_20240521.dam	Instrument Name	Triple Quad 6500+ Low Mass
Project	N/A	Analytes QTY	109:105

Compound name: SA (137.0 / 93.0)

Sample Name	Sample Type	Area (cps)	Is Area (cps)	RT (min)	S/N	Target Conc	Calculated Conc.()
STD_0.01ppb	Standard	N/A	1.215e6	N/A	N/A	0.0100	N/A
STD_0.05ppb	Standard	N/A	1.255e6	N/A	N/A	0.0500	N/A
STD_0.1ppb	Standard	N/A	1.302e6	N/A	N/A	0.1000	N/A
STD_0.5ppb	Standard	2.95e4	1.189e6	5.04	9.9	0.5000	5.219903e-1
STD_1ppb	Standard	4.59e4	1.138e6	5.04	15.5	1.0000	9.252838e-1
STD_5ppb	Standard	2.30e5	1.175e6	5.05	33.3	5.0000	4.960717e0
STD_10ppb	Standard	3.87e5	1.159e6	5.04	43.7	10.0000	8.563547e0
STD_50ppb	Standard	2.45e6	1.169e6	5.04	40.3	50.0000	5.434204e1
STD_100ppb	Standard	4.97e6	1.176e6	5.05	57.5	100.0000	1.095397e2
STD_200ppb	Standard	N/A	1.115e6	N/A	N/A	200.0000	N/A
STD_500ppb	Standard	N/A	1.112e6	N/A	N/A	500.0000	N/A
V2.0_MW_RQC1_20240724	Quality Control	2.99e8	2.902e5	5.02	26.9	0.0000	2.675454e4
Blank	Unknown	N/A	3.284e3	N/A	N/A	N/A	N/A
V3.0_MWMS_20240725_1	Unknown	5.54e5	1.366e6	5.02	32.2	N/A	1.040672e1
MWXS243064a_R1	Quality Control	1.58e6	7.768e5	5.01	43.4	0.0000	5.278440e1
MWXS243064a_R2	Quality Control	1.59e6	7.851e5	5.01	38.1	0.0000	5.253247e1
MWXS243064a_R3	Quality Control	1.58e6	8.557e5	5.00	43.9	0.0000	4.797695e1
T24186682b_a	Unknown	1.49e6	6.900e5	5.02	48.1	N/A	5.599014e1
T24186682b_b	Unknown	1.44e6	6.962e5	5.02	51.5	N/A	5.360706e1
T24186682b_c	Unknown	1.30e6	7.585e5	5.02	41.8	N/A	4.457969e1
T24186683b_a	Unknown	1.68e6	7.918e5	5.01	39.9	N/A	5.515248e1
T24186683b_b	Unknown	2.09e6	9.104e5	5.01	49.0	N/A	5.941350e1
T24186683b_c	Unknown	1.63e6	8.317e5	5.01	39.3	N/A	5.075923e1
T24186684b_a	Unknown	1.36e6	7.399e5	5.01	34.5	N/A	4.758105e1
T24186684b_b	Unknown	1.29e6	7.622e5	5.02	42.6	N/A	4.378357e1
T24186684b_c	Unknown	1.44e6	6.150e5	5.02	43.3	N/A	6.090363e1

Compound name: SA
Regression Equation: y = 0.03848 x + 0.00471 (r = 0.99416) (weighting: 1 / x^2)

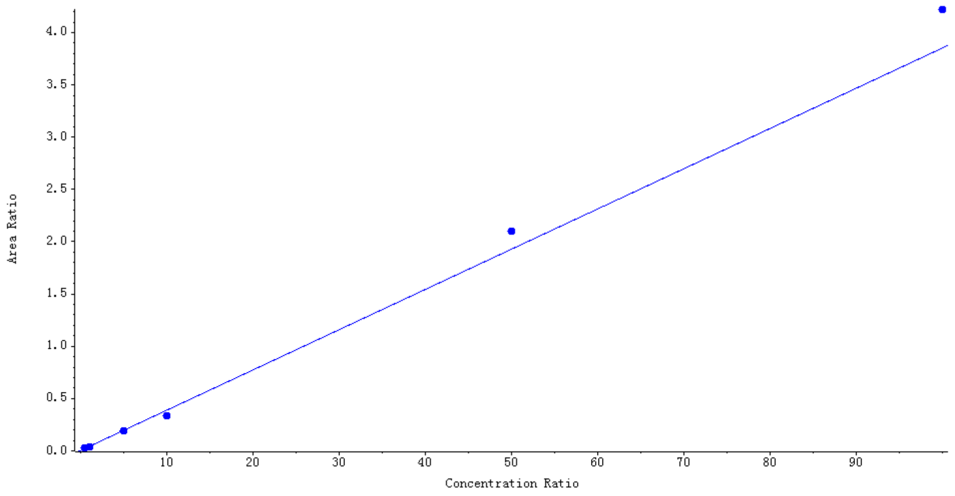

Peak Review

Blank
SA AREA:N/A S/N:N/A

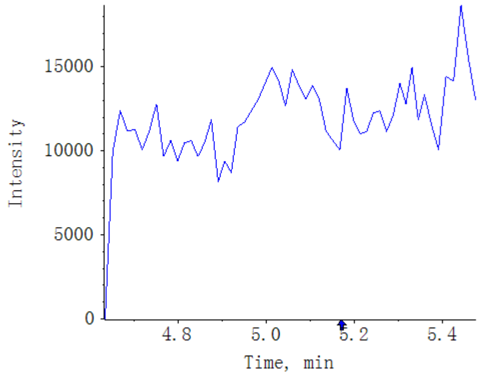

V3.0_MWMS_20240725_1
SA AREA:5.54e5 S/N:32.2

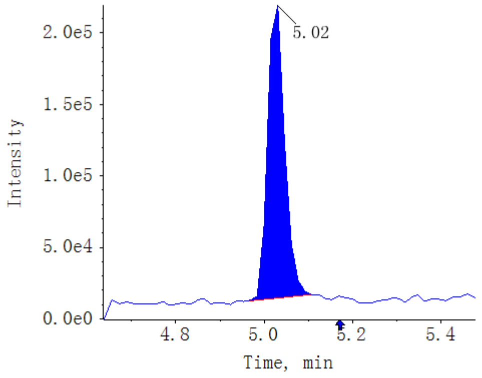

T24186682b_a
SA AREA:1.49e6 S/N:48.1

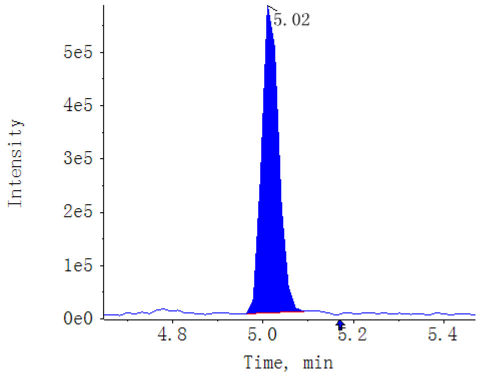

T24186682b_b
SA AREA:1.44e6 S/N:51.5

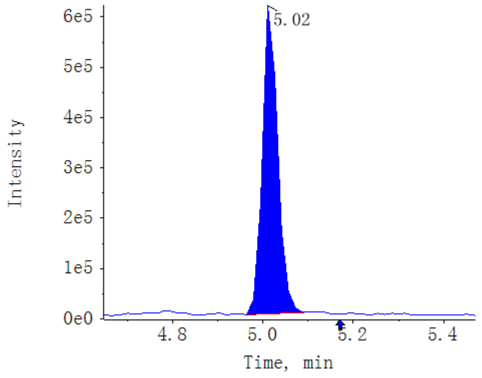

T24186682b_c
SA AREA:1.30e6 S/N:41.8

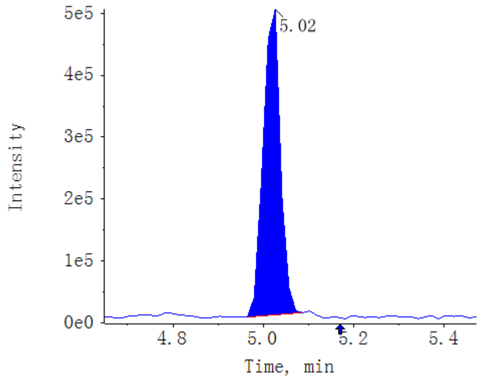

T24186683b_a
SA AREA:1.68e6 S/N:39.9

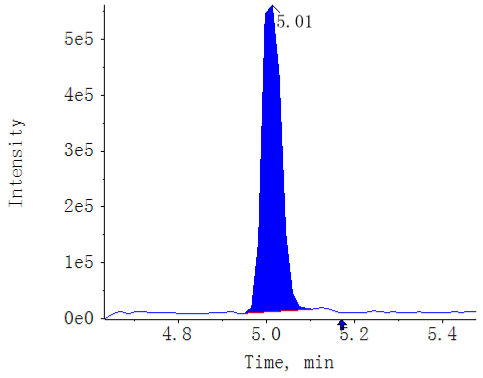

T24186683b_b
SA AREA:2.09e6 S/N:49.0

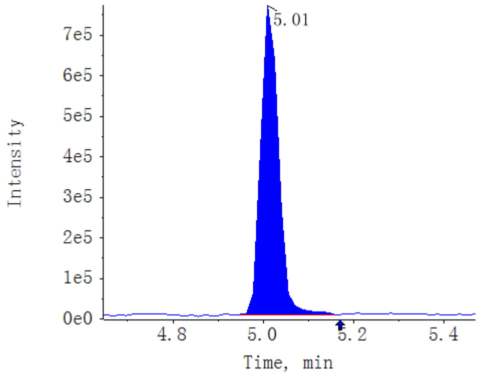

T24186683b_c
SA AREA:1.63e6 S/N:39.3

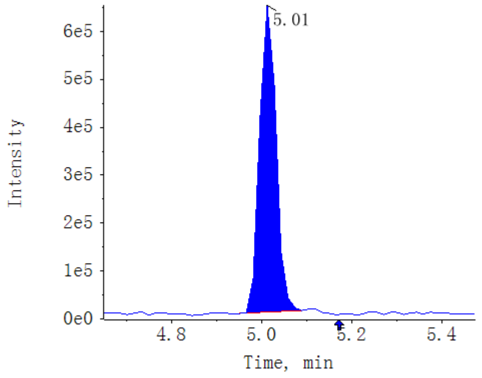

T24186684b_a
SA AREA:1.36e6 S/N:34.5

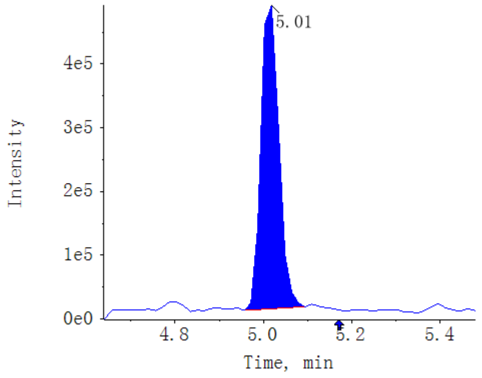

T24186684b_b
SA AREA:1.29e6 S/N:42.6

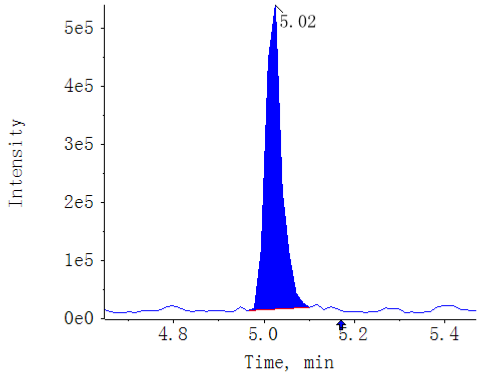

T24186684b_c
SA AREA:1.44e6 S/N:43.3

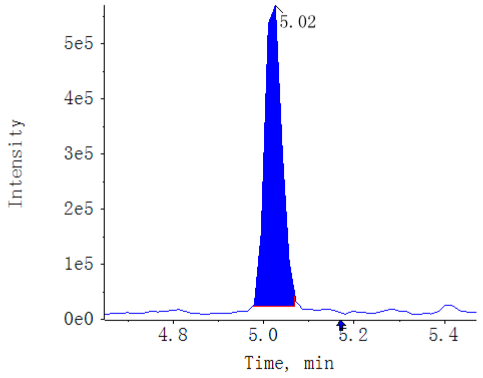

Result Table	MWXS-24-3064-a_9_WH6500-17_A20-3_V6.0_WSS_20240730	Algorithm Used	MQ4
Acquisition Method	ACC-PHs_V6.0_WH6500-17_CMY_20240521.dam	Instrument Name	Triple Quad 6500+ Low Mass
Project	N/A	Analytes QTY	109:106

Compound name: SAG (299.2 / 137.0)

Sample Name	Sample Type	Area (cps)	Is Area (cps)	RT (min)	S/N	Target Conc	Calculated Conc.()
STD_0.01ppb	Standard	1.46e4	1.215e6	3.54	37.0	0.2000	1.940918e-1
STD_0.05ppb	Standard	7.35e4	1.255e6	3.54	34.7	1.0000	1.130487e0
STD_0.1ppb	Standard	1.36e5	1.302e6	3.55	34.3	2.0000	2.050803e0
STD_0.5ppb	Standard	6.26e5	1.189e6	3.54	28.7	10.0000	1.053521e1
STD_1ppb	Standard	1.11e6	1.138e6	3.54	35.7	20.0000	1.963432e1
STD_5ppb	Standard	6.00e6	1.175e6	3.55	39.0	100.0000	1.025648e2
STD_10ppb	Standard	1.12e7	1.159e6	3.54	41.1	200.0000	1.936870e2
STD_50ppb	Standard	6.11e7	1.169e6	3.54	32.8	1000.0000	1.051660e3
STD_100ppb	Standard	1.08e8	1.176e6	3.55	41.1	2000.0000	1.845456e3
STD_200ppb	Standard	1.93e8	1.115e6	3.54	29.5	4000.0000	3.479783e3
STD_500ppb	Standard	N/A	1.112e6	N/A	N/A	10000.0000	N/A
V2.0_MW_RQC1_20240724	Quality Control	2.14e8	2.902e5	3.55	1829.3	0.0000	1.484601e4
Blank	Unknown	N/A	3.284e3	N/A	N/A	N/A	N/A
V3.0_MWMS_20240725_1	Unknown	6.58e6	1.366e6	3.56	4188.7	N/A	9.687523e1
MWXS243064a_R1	Quality Control	1.60e6	7.768e5	3.54	413.7	0.0000	4.137723e1
MWXS243064a_R2	Quality Control	1.59e6	7.851e5	3.55	828.2	0.0000	4.073862e1
MWXS243064a_R3	Quality Control	1.58e6	8.557e5	3.53	611.7	0.0000	3.706359e1
T24186682b_a	Unknown	1.50e6	6.900e5	3.54	625.7	N/A	4.369457e1
T24186682b_b	Unknown	1.42e6	6.962e5	3.54	381.7	N/A	4.091050e1
T24186682b_c	Unknown	1.68e6	7.585e5	3.54	691.7	N/A	4.451427e1
T24186683b_a	Unknown	2.53e6	7.918e5	3.54	1146.5	N/A	6.427403e1
T24186683b_b	Unknown	2.31e6	9.104e5	3.55	994.9	N/A	5.097953e1
T24186683b_c	Unknown	2.31e6	8.317e5	3.55	942.1	N/A	5.591831e1
T24186684b_a	Unknown	1.27e6	7.399e5	3.54	261.3	N/A	3.434277e1
T24186684b_b	Unknown	9.37e5	7.622e5	3.55	232.4	N/A	2.468558e1
T24186684b_c	Unknown	5.23e5	6.150e5	3.55	273.4	N/A	1.707212e1

Compound name: SAG
Regression Equation: y = 0.04972 x + 0.00235 (r = 0.99707) (weighting: 1 / x^2)

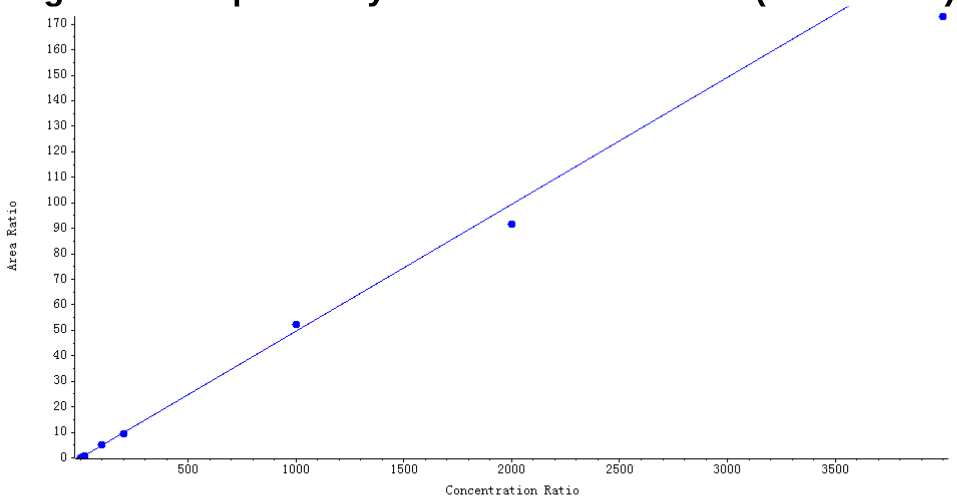

Peak Review

Blank
SAG AREA:N/A S/N:N/A

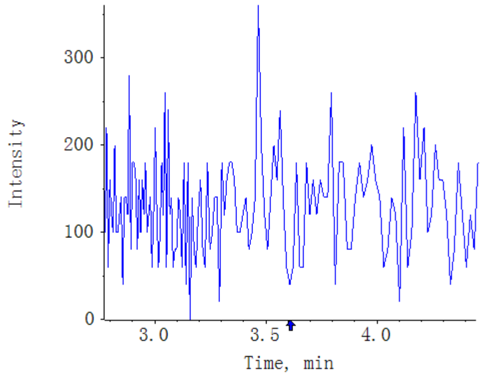

V3.0_MWMS_20240725_1
SAG AREA:6.58e6 S/N:4188.7

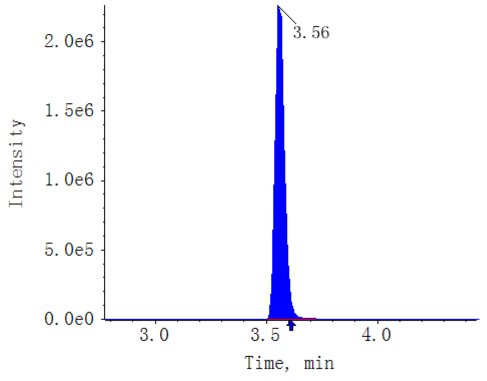

T24186682b_a
SAG AREA:1.50e6 S/N:625.7

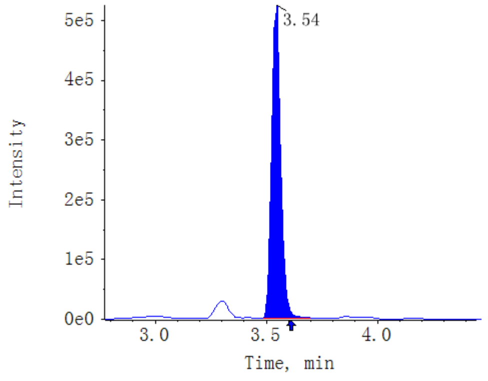

T24186682b_b
SAG AREA:1.42e6 S/N:381.7

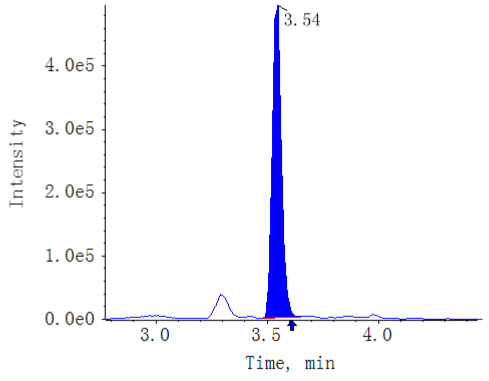

T24186682b_c
SAG AREA:1.68e6 S/N:691.7

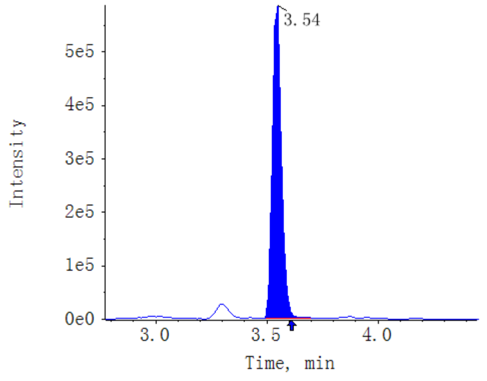

T24186683b_a
SAG AREA:2.53e6 S/N:1146.5

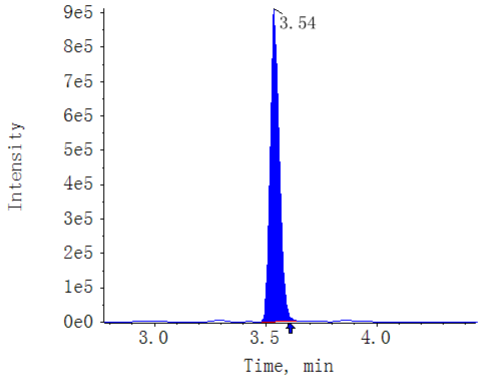

T24186683b_b
SAG AREA:2.31e6 S/N:994.9

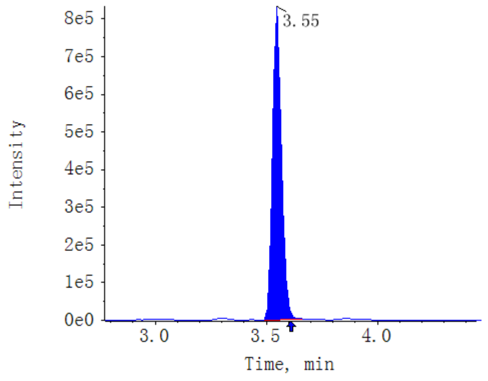

T24186683b_c
SAG AREA:2.31e6 S/N:942.1

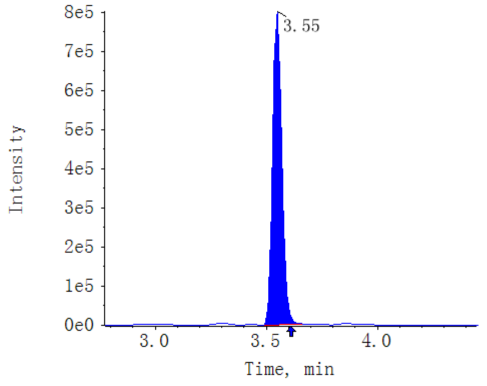

T24186684b_a
SAG AREA:1.27e6 S/N:261.3

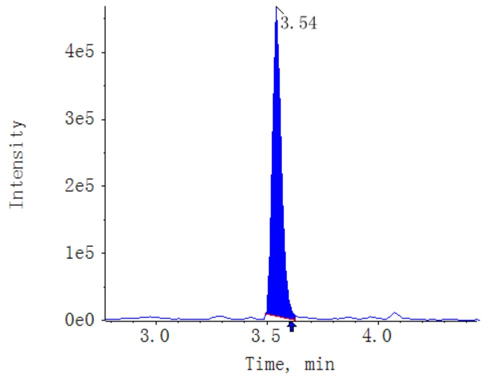

T24186684b_b
SAG AREA:9.37e5 S/N:232.4

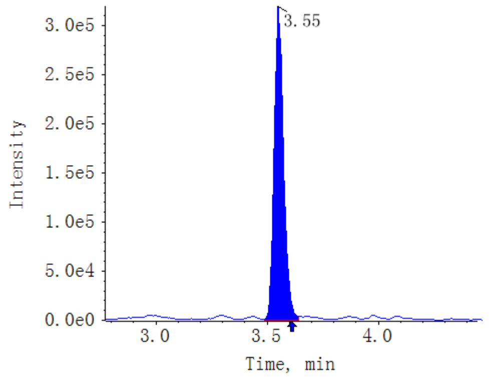

T24186684b_c
SAG AREA:5.23e5 S/N:273.4

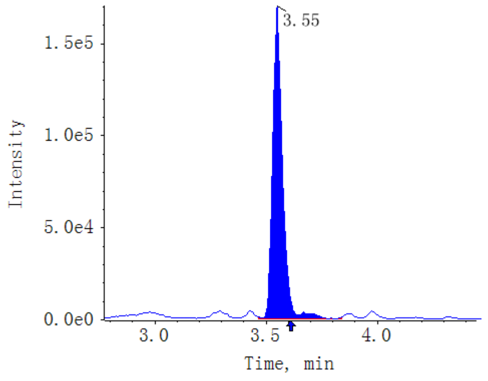

Result Table	MWXS-24-3064-a_9_WH6500-17_A20-3_V6.0_WSS_20240730	Algorithm Used	MQ4
Acquisition Method	ACC-PHs_V6.0_WH6500-17_CMY_20240521.dam	Instrument Name	Triple Quad 6500+ Low Mass
Project	N/A	Analytes QTY	109:107

Compound name: t-CA_1 (146.8 / 77.1)

Sample Name	Sample Type	Area (cps)	Is Area (cps)	RT (min)	S/N	Target Conc	Calculated Conc.()
STD_0.01ppb	Standard	N/A	N/A	N/A	N/A	0.2000	N/A
STD_0.05ppb	Standard	N/A	N/A	N/A	N/A	1.0000	N/A
STD_0.1ppb	Standard	N/A	N/A	N/A	N/A	2.0000	N/A
STD_0.5ppb	Standard	2.08e3	N/A	5.56	11.8	10.0000	1.382725e1
STD_1ppb	Standard	3.26e3	N/A	5.58	4.0	20.0000	1.916584e1
STD_5ppb	Standard	1.60e4	N/A	5.58	19.9	100.0000	7.677678e1
STD_10ppb	Standard	3.66e4	N/A	5.57	42.9	200.0000	1.700599e2
STD_50ppb	Standard	2.27e5	N/A	5.57	55.1	1000.0000	1.031662e3
STD_100ppb	Standard	4.46e5	N/A	5.58	69.8	2000.0000	2.018508e3
STD_200ppb	Standard	N/A	N/A	N/A	N/A	4000.0000	N/A
STD_500ppb	Standard	N/A	N/A	N/A	N/A	10000.0000	N/A
V2.0_MW_RQC1_20240724	Quality Control	N/A	N/A	N/A	N/A	0.0000	N/A
Blank	Unknown	N/A	N/A	N/A	N/A	N/A	N/A
V3.0_MWMS_20240725_1	Unknown	1.88e4	N/A	5.54	21.7	N/A	8.931093e1
MWXS243064a_R1	Quality Control	N/A	N/A	N/A	N/A	0.0000	N/A
MWXS243064a_R2	Quality Control	N/A	N/A	N/A	N/A	0.0000	N/A
MWXS243064a_R3	Quality Control	N/A	N/A	N/A	N/A	0.0000	N/A
T24186682b_a	Unknown	N/A	N/A	N/A	N/A	N/A	N/A
T24186682b_b	Unknown	N/A	N/A	N/A	N/A	N/A	N/A
T24186682b_c	Unknown	N/A	N/A	N/A	N/A	N/A	N/A
T24186683b_a	Unknown	N/A	N/A	N/A	N/A	N/A	N/A
T24186683b_b	Unknown	N/A	N/A	N/A	N/A	N/A	N/A
T24186683b_c	Unknown	N/A	N/A	N/A	N/A	N/A	N/A
T24186684b_a	Unknown	N/A	N/A	N/A	N/A	N/A	N/A
T24186684b_b	Unknown	N/A	N/A	N/A	N/A	N/A	N/A
T24186684b_c	Unknown	N/A	N/A	N/A	N/A	N/A	N/A

Compound name: t-CA_1
Regression Equation: y = 221.19928 x + -983.41848 (r = 0.99799) (weighting: 1 / x)

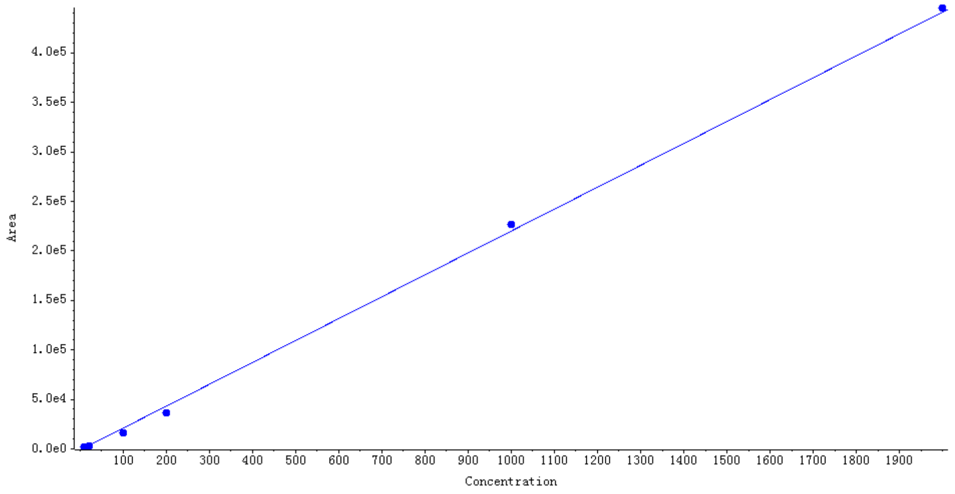

Peak Review

Blank
t-CA_1 AREA:N/A S/N:N/A

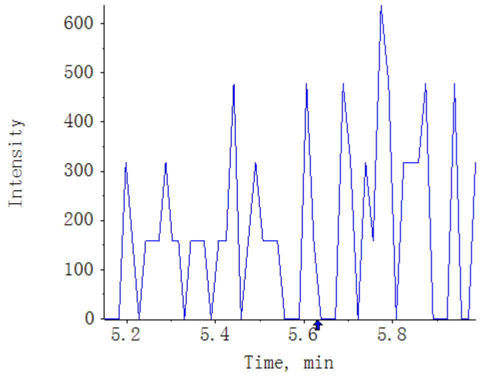

V3.0_MWMS_20240725_1
t-CA_1 AREA:1.88e4 S/N:21.7

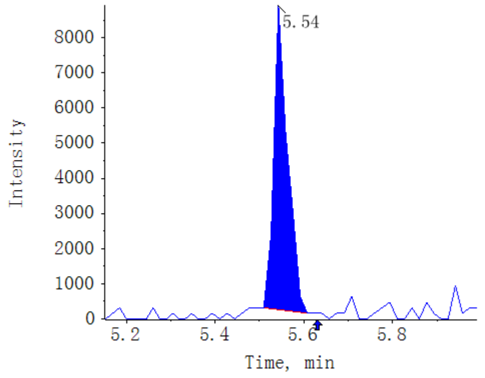

T24186682b_a
t-CA_1 AREA:N/A S/N:N/A

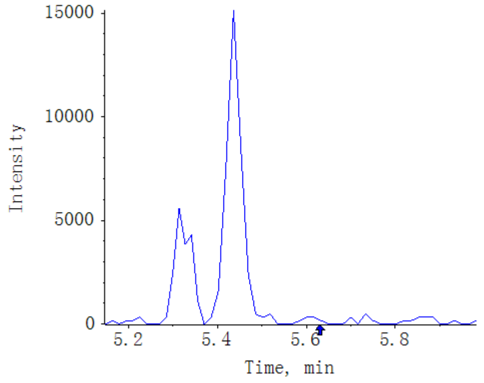

T24186682b_b
t-CA_1 AREA:N/A S/N:N/A

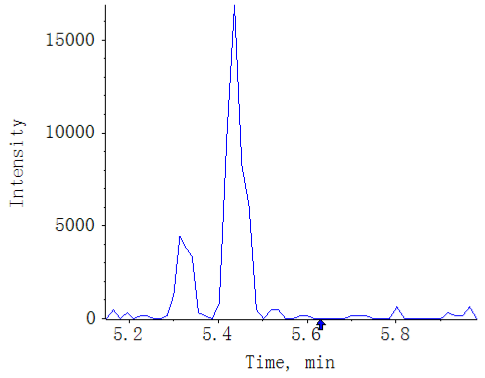

T24186682b_c
t-CA_1 AREA:N/A S/N:N/A

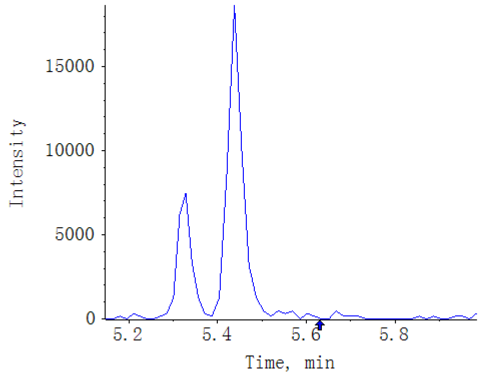

T24186683b_a
t-CA_1 AREA:N/A S/N:N/A

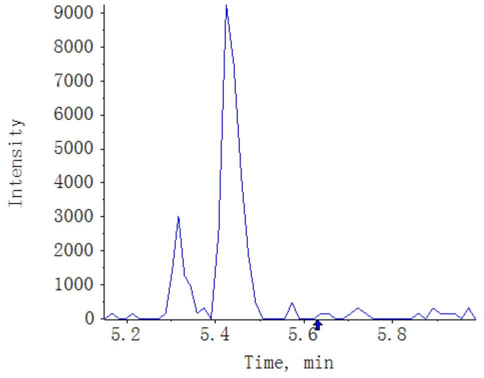

T24186683b_b
t-CA_1 AREA:N/A S/N:N/A

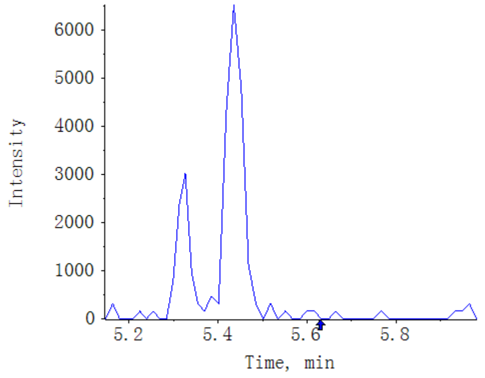

T24186683b_c
t-CA_1 AREA:N/A S/N:N/A

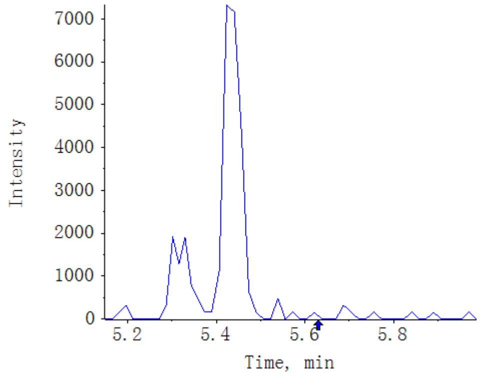

T24186684b_a
t-CA_1 AREA:N/A S/N:N/A

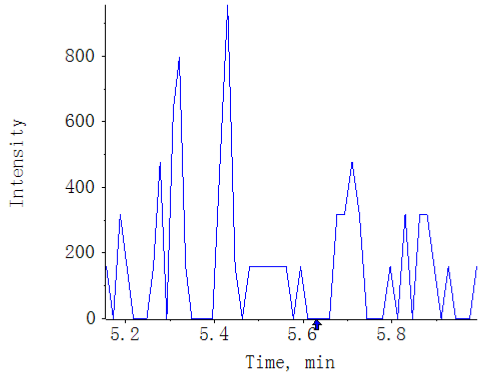

T24186684b_b
t-CA_1 AREA:N/A S/N:N/A

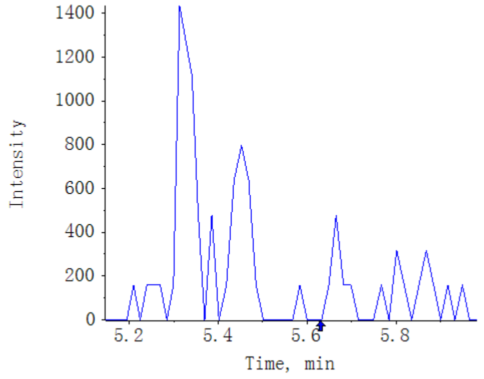

T24186684b_c
t-CA_1 AREA:N/A S/N:N/A

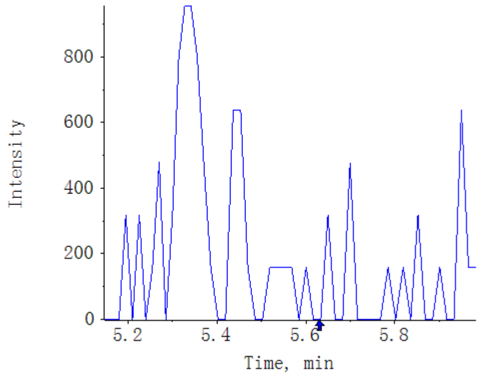

Result Table	MWXS-24-3064-a_9_WH6500-17_A20-3_V6.0_WSS_20240730	Algorithm Used	MQ4
Acquisition Method	ACC-PHs_V6.0_WH6500-17_CMY_20240521.dam	Instrument Name	Triple Quad 6500+ Low Mass
Project	N/A	Analytes QTY	109:108

Compound name: 2-Coumarate (162.8 / 119.0)

Sample Name	Sample Type	Area (cps)	Is Area (cps)	RT (min)	S/N	Target Conc	Calculated Conc.()
STD_0.01ppb	Standard	N/A	N/A	N/A	N/A	0.0100	N/A
STD_0.05ppb	Standard	N/A	N/A	N/A	N/A	0.0500	N/A
STD_0.1ppb	Standard	N/A	N/A	N/A	N/A	0.1000	N/A
STD_0.5ppb	Standard	N/A	N/A	N/A	N/A	0.5000	N/A
STD_1ppb	Standard	3.26e4	N/A	4.90	4.6	1.0000	1.036127e0
STD_5ppb	Standard	1.40e5	N/A	4.91	16.3	5.0000	4.359584e0
STD_10ppb	Standard	2.78e5	N/A	4.89	24.6	10.0000	8.607665e0
STD_50ppb	Standard	1.80e6	N/A	4.89	49.4	50.0000	5.550381e1
STD_100ppb	Standard	3.63e6	N/A	4.90	55.4	100.0000	1.121113e2
STD_200ppb	Standard	N/A	N/A	N/A	N/A	200.0000	N/A
STD_500ppb	Standard	N/A	N/A	N/A	N/A	500.0000	N/A
V2.0_MW_RQC1_20240724	Quality Control	N/A	N/A	N/A	N/A	0.0000	N/A
Blank	Unknown	N/A	N/A	N/A	N/A	N/A	N/A
V3.0_MWMS_20240725_1	Unknown	3.82e5	N/A	4.87	30.2	N/A	1.181867e1
MWXS243064a_R1	Quality Control	N/A	N/A	N/A	N/A	0.0000	N/A
MWXS243064a_R2	Quality Control	N/A	N/A	N/A	N/A	0.0000	N/A
MWXS243064a_R3	Quality Control	N/A	N/A	N/A	N/A	0.0000	N/A
T24186682b_a	Unknown	N/A	N/A	N/A	N/A	N/A	N/A
T24186682b_b	Unknown	N/A	N/A	N/A	N/A	N/A	N/A
T24186682b_c	Unknown	N/A	N/A	N/A	N/A	N/A	N/A
T24186683b_a	Unknown	N/A	N/A	N/A	N/A	N/A	N/A
T24186683b_b	Unknown	N/A	N/A	N/A	N/A	N/A	N/A
T24186683b_c	Unknown	N/A	N/A	N/A	N/A	N/A	N/A
T24186684b_a	Unknown	N/A	N/A	N/A	N/A	N/A	N/A
T24186684b_b	Unknown	N/A	N/A	N/A	N/A	N/A	N/A
T24186684b_c	Unknown	N/A	N/A	N/A	N/A	N/A	N/A

Compound name: 2-Coumarate
Regression Equation: y = 3.23619e4 x + -925.83117 (r = 0.99051) (weighting: 1 / x^2)

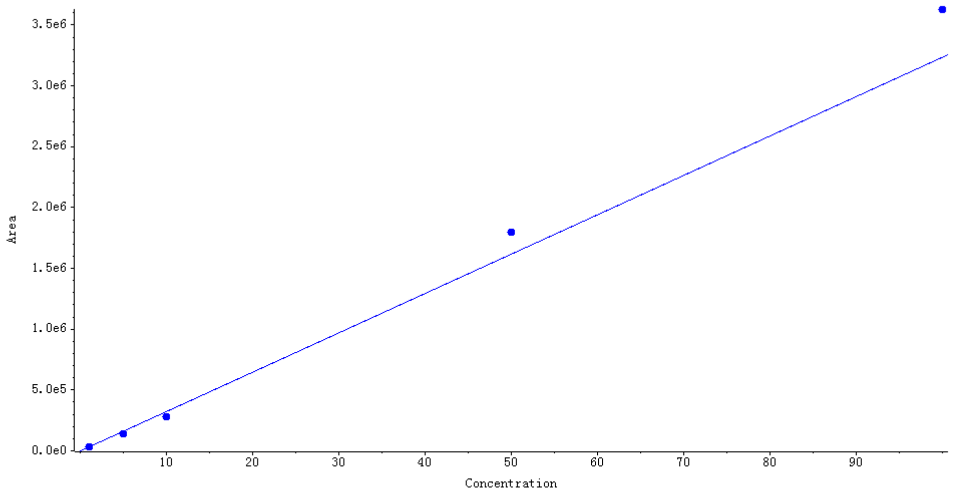

Peak Review

Blank
2-Coumarate AREA:N/A S/N:N/A

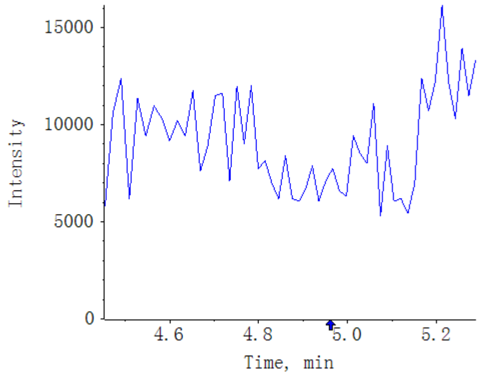

V3.0_MWMS_20240725_1
2-Coumarate AREA:3.82e5 S/N:30.2

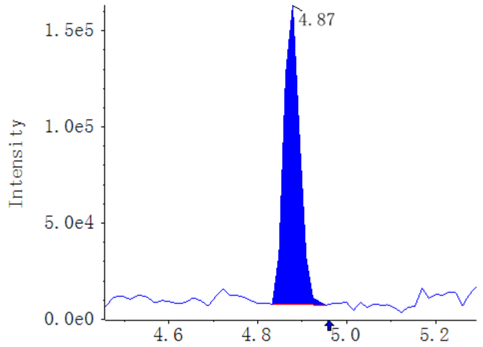

T24186682b_a
2-Coumarate AREA:N/A S/N:N/A

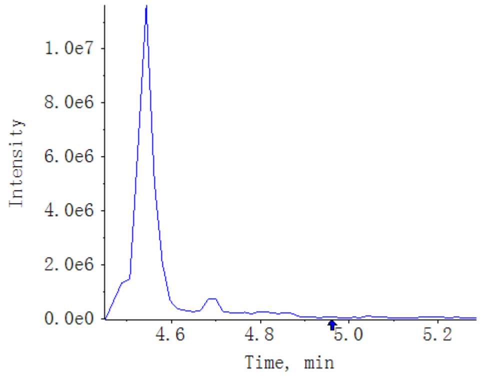

T24186682b_b
2-Coumarate AREA:N/A S/N:N/A

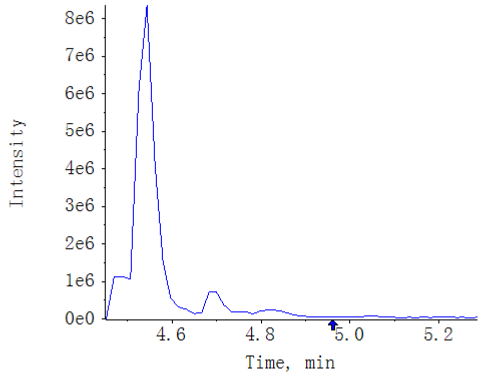

T24186682b_c
2-Coumarate AREA:N/A S/N:N/A

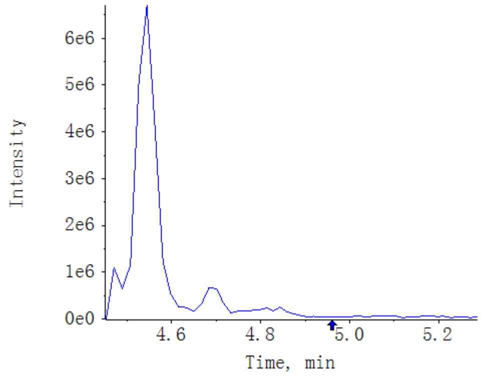

T24186683b_a
2-Coumarate AREA:N/A S/N:N/A

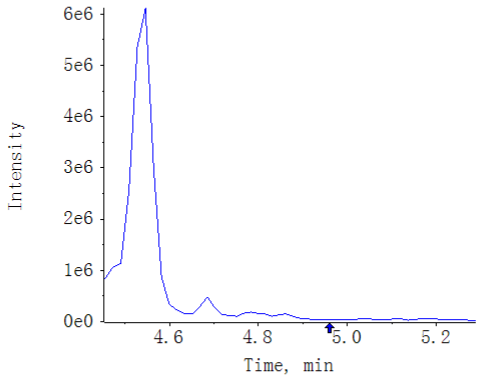

T24186683b_b
2-Coumarate AREA:N/A S/N:N/A

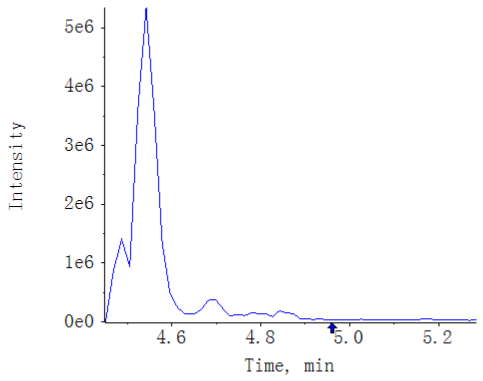

T24186683b_c
2-Coumarate AREA:N/A S/N:N/A

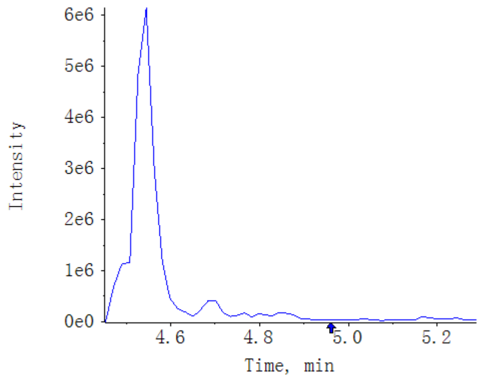

T24186684b_a
2-Coumarate AREA:N/A S/N:N/A

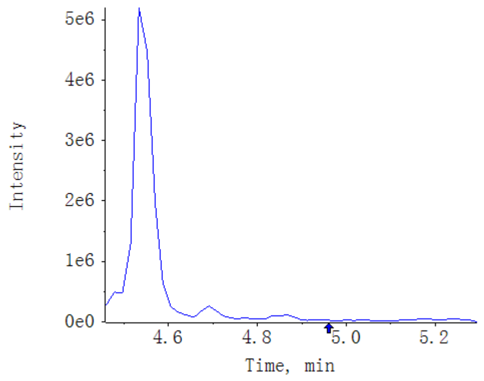

T24186684b_b
2-Coumarate AREA:N/A S/N:N/A

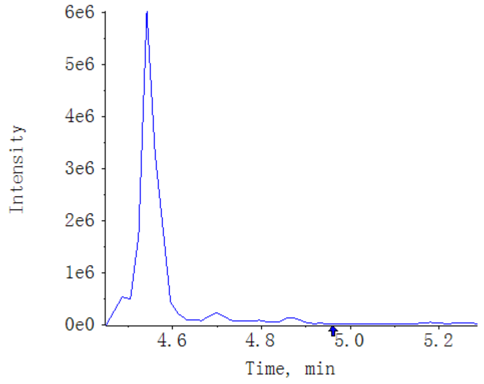

T24186684b_c
2-Coumarate AREA:N/A S/N:N/A

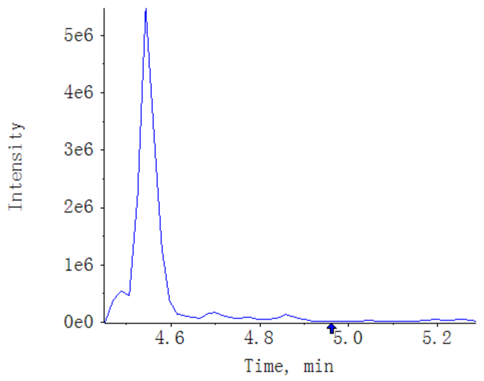

Supplement: Supplementary file 1 [file antioxidants-14-00862-s001.zip › Supplementary materials_2.pdf]
